# Na^+^/K^+^-ATPase Is Present in Scrapie-Associated Fibrils, Modulates PrP Misfolding *In Vitro* and Links PrP Function and Dysfunction

**DOI:** 10.1371/journal.pone.0026813

**Published:** 2011-11-02

**Authors:** James F. Graham, Dominic Kurian, Sonya Agarwal, Lorna Toovey, Lawrence Hunt, Louise Kirby, Teresa J. T. Pinheiro, Steven J. Banner, Andrew C. Gill

**Affiliations:** 1 The Roslin Institute and R(D)SVS, Neuropathogenesis Division, University of Edinburgh, Easter Bush, Roslin, Edinburgh, Midlothian, United Kingdom; 2 Institute for Animal Health, Compton, Newbury, Berkshire, United Kingdom; 3 School of Biological Sciences, University of Warwick, Coventry, United Kingdom; University of Melbourne, Australia

## Abstract

Transmissible spongiform encephalopathies are characterised by widespread deposition of fibrillar and/or plaque-like forms of the prion protein. These aggregated forms are produced by misfolding of the normal prion protein, PrP^C^, to the disease-associated form, PrP^Sc^, through mechanisms that remain elusive but which require either direct or indirect interaction between PrP^C^ and PrP^Sc^ isoforms. A wealth of evidence implicates other non-PrP molecules as active participants in the misfolding process, to catalyse and direct the conformational conversion of PrP^C^ or to provide a scaffold ensuring correct alignment of PrP^C^ and PrP^Sc^ during conversion. Such molecules may be specific to different scrapie strains to facilitate differential prion protein misfolding. Since molecular cofactors may become integrated into the growing protein fibril during prion conversion, we have investigated the proteins contained in prion disease-specific deposits by shotgun proteomics of scrapie-associated fibrils (SAF) from mice infected with 3 different strains of mouse-passaged scrapie. Concomitant use of negative control preparations allowed us to identify and discount proteins that are enriched non-specifically by the SAF isolation protocol. We found several proteins that co-purified specifically with SAF from infected brains but none of these were reproducibly and demonstrably specific for particular scrapie strains. The α-chain of Na^+^/K^+^-ATPase was common to SAF from all 3 strains and we tested the ability of this protein to modulate *in vitro* misfolding of recombinant PrP. Na^+^/K^+^-ATPase enhanced the efficiency of disease-specific conversion of recombinant PrP suggesting that it may act as a molecular cofactor. Consistent with previous results, the same protein inhibited fibrillisation kinetics of recombinant PrP. Since functional interactions between PrP^C^ and Na^+^/K^+^-ATPase have previously been reported in astrocytes, our data highlight this molecule as a key link between PrP function, dysfunction and misfolding.

## Introduction

Transmissible spongiform encephalopathies (TSEs) are a group of fatal neurodegenerative disorders affecting humans and animals. TSEs are characterised by the post-translational conversion of the host prion protein, PrP^C^, to a disease-associated isoform, PrP^Sc^. PrP^C^ is largely α-helical in structure, whilst PrP^Sc^ possesses dramatically increased levels of β-sheet; it is also partially protease resistant and accumulates in insoluble plaques and fibrils during disease. PrP^Sc^ is hypothesised to be the principle component of the TSE infectious agent but the molecular basis of the process by which PrP^C^ is converted to PrP^Sc^ is still unknown [1]. It is assumed that PrP^Sc^ acts catalytically by imposing its conformation onto nascent PrP^C^ causing the disease-associated structural changes. In this case, the information required to passage disease is believed to be encoded in the tertiary or quaternary structure of PrP^Sc^ [2]. A major challenge to this hypothesis is the existence of many different TSE strains that can be passaged, with high fidelity, in congenic hosts. This requires PrP^Sc^ to exist in many different stable isoforms, each capable of passing sufficient information to cause the differing pathology resulting from specific strains of disease [2], [3]. Accumulating evidence suggests that interactions between PrP^C^/PrP^Sc^ and molecular cofactors are necessary for the conversion process to take place [4]. Such molecules could act directly, as chaperones to assist in the misfolding of PrP^C^, or could act indirectly through the formation of a molecular scaffold [5] that allows for the correct spatial alignment between PrP^C^ and PrP^Sc^. Alternatively, accessory molecules may modulate PrP^C^ structure to ensure the correct folded form is available for conversion [6]. Many accessory molecules may exist *in vivo* and their involvement in prion protein misfolding may differ depending on TSE strain [7]. Defining the identity of molecular cofactors that aid prion misfolding *in vivo* is a key goal in TSE research.

In order for accessory molecules to be part of the conversion process they must come into contact with either PrP^C^ or PrP^Sc^. Cofactors may, therefore, be molecules with which the prion protein interacts during normal cell biology. Based on published studies, a range of candidates exists [8], [9], [10] and particular examples include neural cell adhesion molecule (N-CAM) [11] and the 37/67 kDa Laminin receptor [12]. PrP^C^ has also been reported to bind to copper ions [13], nucleic acids [14], lipids [15] and glycosaminoglycans [16]. In cell culture, pro-low density lipoprotein receptor 1 (LRP-1) [17] and glypican-1 [18] have recently been suggested to mediate PrP misfolding. In short, a range of different molecules covering almost all biomolecular subsets are candidates for prion conversion cofactors. However, Deleault *et al*. [19] published work demonstrating that misfolding of the prion protein into an infectious isoform *in vitro* could be achieved using only poly(A)RNA and molecules, including lipidic components, that co-purified with PrP^C^. Wang *et* al have also shown that recombinant PrP (recPrP), expressed by a prokaryotic system, can be induced to misfold into infectious isoforms in the presence of lipid and nucleic acid, strengthening the case for these molecules as *bona fide* prion protein conversion cofactors [20]. Both pieces of work, however, made use of misfolding assay conditions with periodic sonication, which may impart sufficient energy to overcome the conversion threshold that would normally be facilitated by cofactors *in vivo* [21]. Furthermore, recent results suggest that nucleic acid may not aid conversion in all mammalian systems and, in some species, cofactors may be localised to the plasma membrane of cells [22]. Together these observations demonstrate that the identities of the molecular species aiding prion conversion, particularly those molecules playing this role *in vivo*, are still largely unknown.

Accessory molecules that facilitate prion protein misfolding may become integrated into the growing protein fibril. Several researchers have reported the identification of proteins enriched in TSE-specific fibrils but have either used no negative control preparations [23], [24], or have used proteinase K (PK) in the purification procedure [25]. The PK step retains the protease-resistant core of PrP^Sc^ but may result in digestion of cofactors that are bound loosely in the fibril and are accessible to proteases. Even partial PK digestion of such proteins will hinder MS-based protein identification.

We have isolated scrapie-associated fibrils (SAF) from mouse brains by means of crude preparation procedures in the absence of protease treatment and have adopted a shotgun proteomics approach to the identification of proteins that co-purify with the SAF. SAF preparations were made from the brains of mice infected with 3 different strains of mouse-passaged scrapie, ME7, 79A and 22F. As controls, we also made equivalent preparations from uninfected wildtype mouse brains (WT) and from mice in which the *Prnp* gene has been knocked out (PrP^−/−^). We found several proteins in control preparations that have previously been reported [23], [24] to be integral to SAF. Additionally, several molecules were identified that appear specifically associated with SAF, including the α-chain of sodium/potassium transporting ATPase (Na^+^/K^+^-ATPase) and an isoform of the guanine nucleotide binding protein, but there was limited evidence for proteinaceous cofactors segregating specifically and reproducibly with SAF from any one of the TSE strains used. By use of two established *in vitro* misfolding assays, we found that Na^+^/K^+^-ATPase modulates the misfolding of recPrP, suggesting that it may act as a non-strain specific catalyst in prion misfolding *in vivo*. Coupled with reports of functional associations between PrP^C^ and Na^+^/K^+^-ATPase in cultures of astrocyte s [26], our novel findings provide a key molecular link between PrP function and misfolding and suggest that neurotoxicity associated with prion diseases may involve both loss and gain of function.

## Results and Discussion

### Multiple proteinaceous species copurify with PrP^Sc^ in SAF from three different mouse strains

In our laboratory, routinely we use a method for the preparation of scrapie associated fibrils from TSE-infected brain homogenate that was developed over two decades ago [27]. The procedure is relatively crude and involves tissue homogenisation followed by detergent extraction and differential centrifugation to enrich for detergent insoluble, aggregated species. Further extraction and processing is possible from this stage to produce preparations that retain TSE infectivity, but such processes often involve the use of harsh solvent conditions or protease treatment to remove PK-sensitive proteins [28]. Although clearly allowing enhanced purification of PrP^Sc^, these procedures may also remove cofactors that were important *in vivo* during the misfolding process. Such cofactors may be loosely associated with SAF and susceptible to protease treatment and we wanted to retain these proteins since they may be relevant to the misfolding process. Hence, for all analyses, SAF preparations were used directly without further processing.

**Figure 1:**
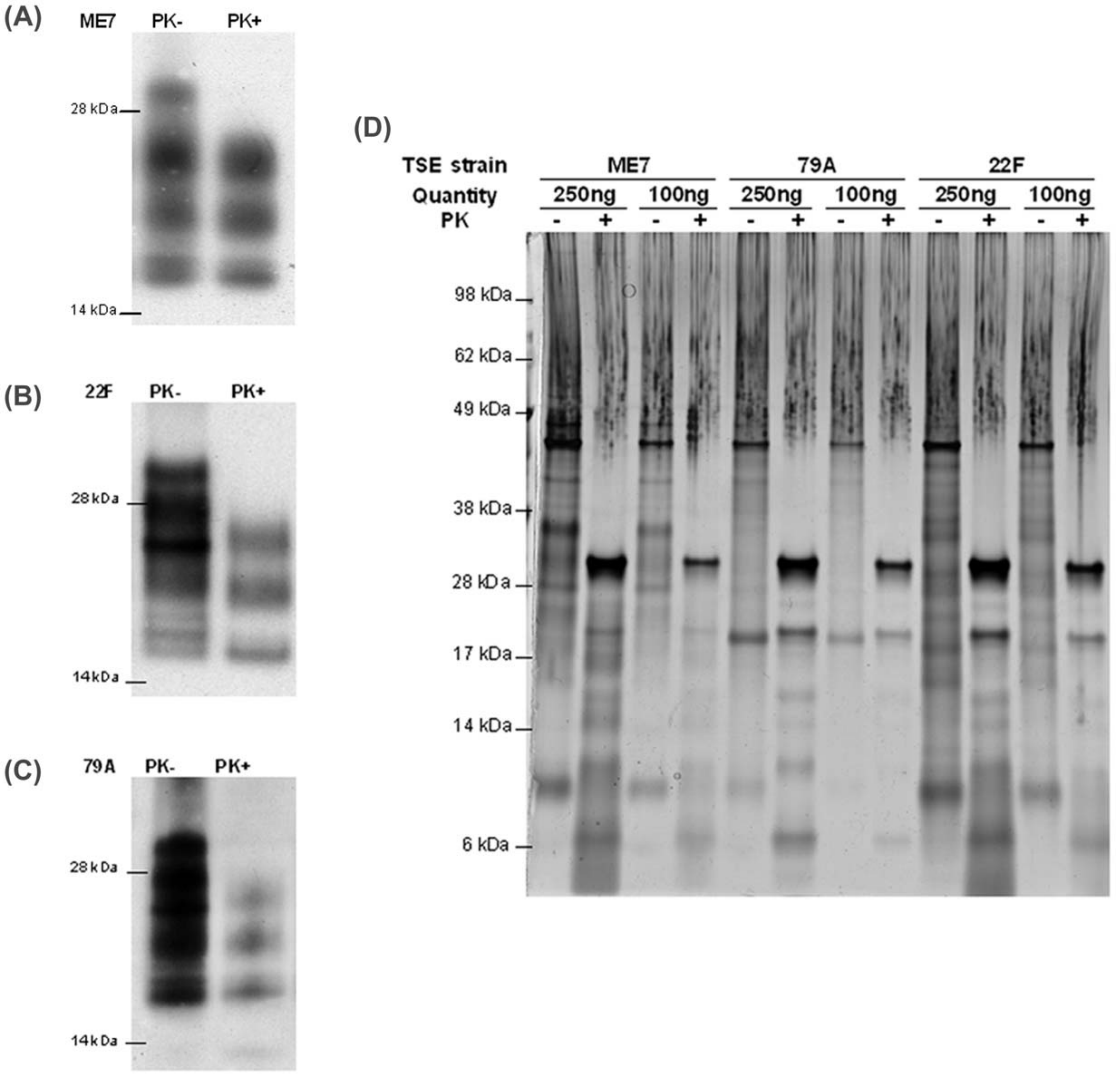
SAF preparations contain protease resistant PrP and a range of other proteins. Panels (A), (B) and (C) show representative results of Western blotting of SAF preparations from mice infected with ME7, 22F and 79A scrapie respectively, both with and without PK digestion. Blots were probed with the anti-PrP antibody 8H4. Quantities stated are estimates of the amount of PrP^Sc^ based on a yield of 10 µg PrP^Sc^ per brain. Panel (D) shows a silver stained SDS-PAGE gel of different SAF preparations. Two different dilutions were loaded for each SAF preparation, both with and without PK digestion. Quantities stated relate to PrP^Sc^ amounts loaded after balancing based on semi-quantitative Western blotting. Molecular weight markers are in kDa.

SAF were purified from the brains of mice terminally affected with the mouse-passaged scrapie strains ME7, 79A and 22F. After purification, SAF-specific proteins were separated by SDS-PAGE and the gels were either blotted to PVDF and the membrane probed with the anti-PrP antibody 8H4, or proteins were visualised by silver staining. Representative Western blots are shown in Figure 1(a)–(c), with and without proteinase K treatment. For each Western blot we loaded a nominal amount of 0.1 µg of SAF onto SDS-PAGE gels based on the estimate that 10 µg of SAF can be extracted from a single mouse brain. Prior to PK digestion, PrP resolves as a mixture of isoforms depending on the presence or absence of the two N-linked carbohydrates and the N-terminal region. After PK digestion, for all three strains PrP-immunoreactive bands separated into characteristic triplets between 17–27 kDa associated with the three different glycoforms of PrP: un-, mono- and di-glycosylated. Significant work has shown that ratios of the prevalence of each glycoform can be used as a diagnostic marker for different strains of TSE disease, at least when the total PrP^Sc^ from a brain sample is analysed [29]. The intensities of bands on our Western blots are comparable with glycoform ratios previously published [30], demonstrating the TSE strain-specific nature of the SAF preparations. The densitometric intensities of Western Blot bands were compared to a range of standards produced by Western blotting recPrP at different concentrations (not shown). This allowed us to estimate the actual amount of PrP^Sc^ present in each SAF preparation and allowed the standardisation of subsequent analyses for PrP^Sc^ content.

We used SDS-PAGE to attempt to determine the approximate number of proteins co-purifying alongside PrP^Sc^ in each strain and whether differences could be detected in the protein profile of SAF preparations from different mouse-passaged scrapie strains. Figure 1(d) shows a representative silver stained gel of SAF preparations at two different dilutions, as determined by Western blotting, and both with and without PK treatment. Prior to PK digestion, SAF from each strain contains a range of non-PrP proteins, as evidenced by a variety of discrete bands and poorly resolved smears on the gel (Figure 1(d)). A particularly intense band is evident around 45 kDa and is present in SAF preparations from all 3 strains. After PK digestion of SAF from each strain, there are also poorly resolved signals on the gel although most appear in the low mass region. The intense band at ∼30 kDa in PK positive lanes represents PK. Since SAF are poorly soluble and aggregated, the quality of SDS-PAGE separations cannot readily be improved and from our gels it was problematic to compare protein species present in SAF from different strains; in non-PK treated lanes, the proteins are too numerous to quantify and many of the bands are too diffuse to allow individual protein species to be identified or to allow either qualitative or quantitative comparisons between strains. However, the reduced number of protein signals after PK digestion justifies the use of purification methods that do not employ protease treatment to analyse SAF-associated proteins. To identify SAF-associated proteins, we adopted the method of shotgun proteomics [31], a technique that avoids SDS-PAGE analysis and that allows the analysis of proteins directly from in-solution tryptic digests.

### Control preparations contain proteins previously suggested to be integral to SAF

The SAF preparation protocol is believed to result primarily in the enrichment of amyloid fibrils. However, since our procedure is relatively crude and does not use a protease step, there will inevitably be contamination from other proteins that are not incorporated into SAF *in vivo*, but which share similar biochemical properties, such as insolubility and/or high molecular weight. In the absence of PK treatment or quantitative comparisons of SDS-PAGE protein profiles, there is no proteomic-based method to discriminate those proteins that are present in SAF preparations non-specifically from those that are genuinely SAF-associated. To allow us to identify and discard non-specifically co-purifying proteins from shotgun proteomic analyses, the SAF purification protocol was applied to uninfected WT and PrP^−/−^ mouse brains to generate control preparations. These preparations were applied to our proteomics workflow to produce a list of proteins that could be excluded from subsequent analysis of PrP^Sc^-containing SAF preparations.

After reduction, alkylation and tryptic digestion under denaturing conditions, online nanoLC-MS/MS of control preparations from uninfected WT mouse brains allowed the identification of several purified proteins. The identities of these proteins are reported in Table 1 and were assessed to be significant if there were a minimum of two peptides identified by LC-MS/MS with protein-level Mascot scores above the confidence threshold. Four proteins were identified in preparations from normal mouse brains: three different chains of calcium/calmodulin dependent kinase (CamKII) and a ferritin isoform. A control preparation from PrP^−/−^ mice had a vastly reduced number of identified peptides and the only significant hit was to CamKII α-chain. Other proteins were identified in SAF preparations from wildtype mice (see Supplementary Mascot Searches S1), but were below the level of confidence or had only a single peptide identified, hence are not reported here.

**Table 1:** LC-MS/MS identification of proteins associated with the mock-SAF preparations of uninfected mouse brains.

Gene and protein name	WT Mascot score(Peptide matches)	PrP^−/−^ Mascot score(Peptide matches)
CamK2a Calcium/calmodulin dependent kinase type II α-chain	1043 (8)	276 (4)
CamK2b Calcium/calmodulin dependent kinase type II β-chain	313 (10)	-
Camk2d Calcium/calmodulin dependent kinase type II δ-chain	198 (5)	-
Ftl1 Ferritin (light chain 1)	71 (2)	-

The SAF preparation procedure was carried out on both uninfected wildtype mouse brains (WT) or uninfected knockout mouse brains (PrP^−/−^). Four brains for each mouse strain were homogenised prior to detergent extraction and differential centrifugation to isolate protein species. Precipitated proteins were reduced and alkylated in 6 M guanidine hydrochloride then digested with typsin. Tryptic peptides were separated and detected by online LC-MS/MS and the data searched against the IPI murine database by use of the Mascot search algorithm. Proteins that were below the level of significance or for which only single peptides were identified are not reported.

Since the SAF purification procedure enriches for proteins that are high molecular weight and detergent insoluble, it is not surprising that CamKII and ferritin were present in control preparations. The native proteins exist as multimers of high molecular weight (500–600 kDa, and 450 kDa respectively) and ferritin is known to oligomerise, which will cause further increases in molecular weight [32]. Ferritin has previously been shown to contaminate SAF preparations [33] whilst CamKII, a serine/threonine protein kinase located in synaptic junctions, is highly abundant in brain and one may expect at least a proportion of the protein to be present within the detergent-insoluble fractions. Previous proteomic studies of SAF have identified CamKII and ferritin as co-purifying with TSE-specific SAF preparations. These studies, however, did not include shotgun MS analyses of control preparations [23], [24], although a more recent study by Moore *et al*, which did include this type of experiment, also identified these two proteins in control preparations [25]. Thus, our data reinforces the notion that these proteins are non-specific contaminants of SAF preparations and are not associated with PrP^Sc^. As a result of additional processing steps resulting in increased protein concentrations, Moore *et al.* also found additional proteins present in control preparations, including ribosomal and proteasomal subunits, versican core protein and myelin associated proteins. Our experiments, in conjunction with those of Moore *et al.*, provide a background list of proteins that appear to co-purify to the same fractions as SAF using the isolation techniques described, even in the absence of TSE disease in mice. This allows any identification of these proteins in analyses of SAF preparations from infected mice to be interpreted with care. We cannot rule out that proteins identified in control preparations may also associate with PrP^Sc^ and therefore become more highly enriched in preparations when comparing TSE-infected verses non-infected mouse brains. To prove this would necessitate carefully controlled quantitative proteomic work.

### Mass spectrometry of infected SAF preparations confirms the presence of specific proteins in addition to PrP^Sc^

To identify proteins that associate with PrP^Sc^, particularly those that may be strain specific, replicate SAF preparations were generated from the brains of mice inoculated with ME7, 22F and 79A scrapie. After reduction, alkylation and tryptic digestion, peptides were analysed by online LC-MS/MS and the resulting MS/MS spectra were submitted to Mascot for searching against the IPI mouse database. As previously, proteins were discounted if they had only a single peptide hit associated with them or if they were below the significance threshold determined by the Mascot algorithm. We used a confidence limit of 99% and this yielded peptide-level false-positive discovery rates that were all less than 2.5% and typically 0%. A compiled list of proteins identified by the above criteria is shown in Table 2, but full Mascot search results are also provided as Supplementary Mascot Searches S1. Some proteins identified in our experiments had previously been identified in the uninfected control preparations, including various chains of the CamKII protein and both light and heavy chains of ferritin. Additionally, some proteins detected in mock SAF preparations by Moore *et al* [25] were also identified in our infected SAF preparations and include various actin isoforms, versican core protein and myelin-associated oligodendrocyte basic protein. These proteins are not included in table 2 but a full, compiled list of proteomic identifications is available (Table S1).

**Table 2:** Proteins present in scrapie associated fibrils from ME7, 79A and 22F mouse-passaged scrapie.

TSE strain (code for supporting data)	ME7 (10881)	ME7 (10754)^c^	22F (10882)	22F (10753)^c^	79A (10883)	79A (10755)^c^
Gene and Protein Name	Mascot Score (peptide matches)	Mascot Score (peptide matches)	Mascot Score (peptide matches)	Mascot Score (peptide matches)	Mascot Score (peptide matches)	Mascot Score (peptide matches)
Tubb# Tubulin β-chains, various^a^	Max 259 (7)	Max 392 (11)	Max 415 (6)	Max 504 (8)	Max 246 (6)	Max 656 (12)
Gnao1 Isoform Alpha-2 of Guanine nucleotide-binding protein G(o) subunit alpha^a^	227 (3)	278 (4)	70 (3)	212 (3)	208 (2)	367 (7)
Apoe Apolipoprotein E	226 (9)	92 (6)	nd	nd	147 (5)	67 (4)
Atp1a3 Na+/K+ ATPase subunits α3/α2^a^	171 (3)	312 (5)	198 (4)	130 (3)	dbcl	397 (8)
Prnp Major prion protein	168 (4)	236 (5)	41 (2)	nd	70 (3)	282 (5)
Srrm2 Serine arginine repetitive matrix protein 2	134 (4)	nd	nd	nd	nd	nd
H1f0 Histone H1.0	102 (3)	nd	nd	53 (2)	nd	nd
Hspa8 Heat shock cognate 71 kDa protein	79 (3)	52 (2)	nd	nd	nd	136 (6)
Tbc1d10b TBC1 domain family, member 10b	76 (2)	nd	nd	nd	nd	nd
Nsf Vesicle-fusing ATPase	67 (2)	37 (2)	nd	nd	nd	dbcl
Ckb Creatine kinase B-type	56 (3)	341 (5)	160 (3)	dbcl	dbcl	170 (5)
Lrp1 Pro low-density lipoprotein receptor-related protein 1	37 (2)	nd	nd	nd	88 (3)	170 (3)
Tuba# Tubulin α-chains, various^a^	nd	246 (6)	288 (3)	341 (6)	nd	Max 427 (9)
LOC100039214; similar to Glyceraldehyde-3-phosphate dehydrogenase isoform 1	dbcl	186 (3)	78 (3)	99 (2)	dbcl	250 (7)
Dlg4 Isoform 2 of Disks large homolog 4	nd	nd	65 (2)	nd	dbcl	nd
Ubb;Ubiquitin	dbcl	dbcl	dbcl	171 (2)	nd	158 (3)
Cnp Isoform CNPII of 2′,3′-cyclic-nucleotide 3′-phosphodiesterase	nd	nd	nd	nd	dbcl	159 (3)
Syn1 Isoform Ib/Syn2 isoform IIb of Synapsin1	nd	nd	nd	nd	nd	94 (4)/47 (2)
Dnm1 Isoform 1 of Dynamin 1	nd	nd	nd	nd	nd	66 (2)
Eno1 Alpha Enolase	nd	nd	nd	nd	nd	60 (2)

SAF were purified from the brains of mice infected with ME7, 22F or 79A strains of mouse-passaged scrapie. Duplicate preparations were made and the 6 samples were reduced and alkylated in 6 M guanidine hydrochloride prior to tryptic digestion. Tryptic peptides were separated and detected by online LC-MS/MS and the data searched against the IPI murine database by use of the Mascot search algorithm. Proteins are listed in the table if they were identified conclusively in at least one SAF preparation. Protein hits were deemed to be conclusively identified if they were above the level of significance (as determined by Mascot scores) and more than one peptide matched to the sequence. Proteins identified in these experiments but that had previously been identified in control preparations from uninfected brains are not listed in this table, but full compiled results are available (Table S1). nd – not detected. dbcl – protein detected but below the level of confidence because only a single peptide was matched.

a – due to extensive sequence homology between different chains of these proteins it is not possible to tell whether only a single isoform or multiple isoforms are present.

We detected over 20 individual proteins in our SAF preparations. The prion protein was detected in 5/6 preparations, although was not detected in one of the replicate samples from 22F infected brains. The reason for this is not clear, but we noticed that over several repeat preparations of 22F fibrils, there was a reduction in protein yield and it is conceivable that trace levels of specific proteases co-purified with SAF from this strain and caused excessive proteolysis of the sample. Addition of protease inhibitors during SAF preparation is not compatible with subsequent tryptic digestion and MS analysis, hence we were unable to avoid excessive proteolysis. Furthermore, we also note that tryptic digestion of PrP yields only a handful of peptides that are immediately amenable to LC-MS/MS analysis and sequence coverage of PrP is limited at best. Against the limited detection efficacy of PrP by MS techniques, any reduction in yield of tryptic peptides during proteomic workflows would dramatically inhibit detection of prion protein.

The proteins with the highest Mascot scores were either chains of the CamKII protein, isoforms of β-tubulin or isoforms of actin. Tubulin is an integral component of microtubules – polymeric, high molecular weight assemblies that form part of the cytoskeleton and are abundant in the brain. As a result, tubulin isoforms may have been expected to occur in control preparations, since the generic nature of the techniques used will necessarily select for molecules with the same physical characteristics of PrP^Sc^. However, tubulin isoforms were not identified conclusively in control preparations and this strengthens the evidence for their specific association with PrP^Sc^ in fibrillar deposits. The sequence similarity between different β-tubulin isoforms is high and it was not possible unambiguously to determine the predominate isoform. Interestingly, different tubulin isoforms have been found to be associated with inclusions formed during other protein misfolding diseases [34], [35], [36], [37] and microtubules may function as a scaffold to bring together normally folded proteins with a growing nucleus of misfolded protein. Alternatively, protein aggregates may associate with microtubles and other cytoskeletal structures after the aggregates have formed, as a mechanism by which the cell localises misfolded protein. It is noteworthy that many protein misfolding diseases are characterised by deregulation of tubulin polymerisation, potentially suggesting a key role for cytoskeletal components in neurodegeneration. However, we also cannot rule out that a non-physiological association occurs between tubulin and PrP^Sc^, during the SAF isolation procedure.

In addition to cytoskeletal proteins, several plasma membrane bound proteins were also identified as present in SAF preparations from multiple scrapie strains. Proteins such as the α-chain of Na^+^/K^+^-ATPase (note that peptides matched to both α2- and α3-chains) and the G_O_ G-protein Gnao1 were detected in almost all SAF preparations, suggesting that they are not strain specific cofactors. Nevertheless, the plasma membrane has been hypothesised to be the site of prion conversion based on evidence derived from cell culture models [38] whilst recent data from our laboratory [39] and also that of others [22], [40], has indicated that plasma membrane preparations may contain factors that enhance conversion of PrP. The presence of plasma membrane specific proteins in our SAF preparations could, therefore, point to the cellular location of conversion. These proteins may be involved in normal PrP^C^ function, in misfolding or may simply be located proximal to the misfolding PrP^C^ molecule. As such, they may become integrated into the fibril in either active or passive processes. In addition to Na^+^/K^+^-ATPase and Gnao1, other plasma membrane associated proteins that were detected in SAF included apolipoprotein E (ApoE), isoform 2 of disks large homolog 4 and LRP-1, which has previously been suggested to play a role in PrP^C^ trafficking [17], [41].

The remainder of proteins detected in multiple SAF preparations were predominately cytoplasmic and included creatine kinase B, which was detected in preparations of SAF from all three strains and which has previously been shown to bind PrP^C^ [42], a GAPDH-like protein, a histone isoform as well as heat shock cognate 71 kDa protein and a protein with homology to ubiquitin. These latter two proteins are part of the unfolded protein response and they have been found to be associated with inclusions in other protein misfolding diseases. In single SAF preparations we also detected the TBC1 domain family member 10b, the vesicle fusing ATPase, Nsf, the serine-arginine repetitive matrix protein 2 (srrm2), CNPII, synapsin-1 and dynamin 1, amongst others. None of the proteins that we identified were present specifically and reproducibly in repeat SAF preparations from a single TSE strain suggesting that if strain specific cofactors exist they are non-proteinaceous, do not get integrated into the amyloid deposits or are present only in quantities too small to allow detection.

### Western blotting validates the presence of ApoE and Na^+^/K^+^-ATPase in SAF preparations

**Figure 2:**
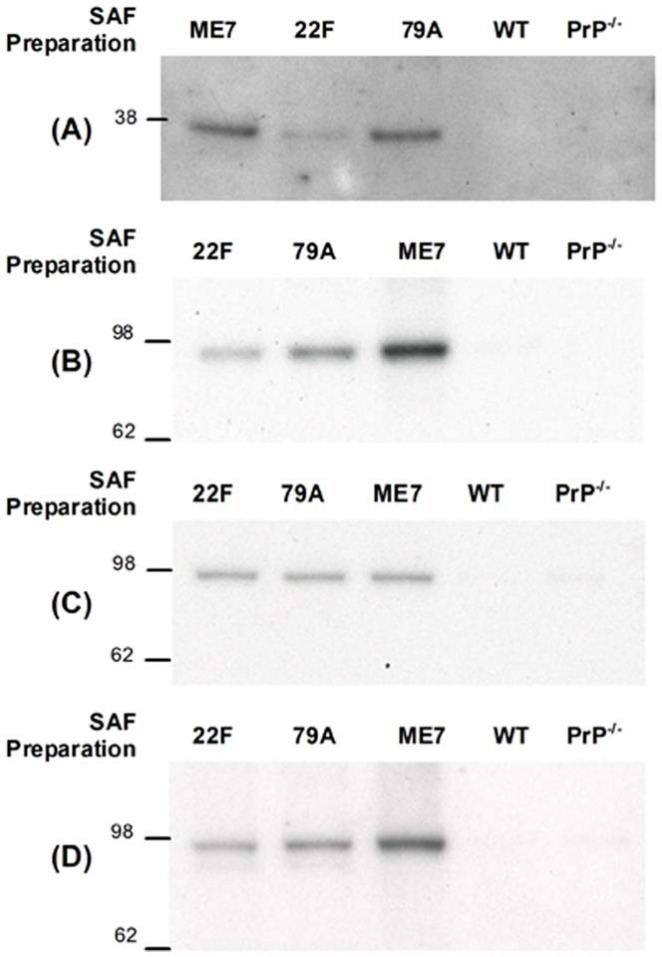
Western blotting confirms the presence of ApoE and Na^+^/K^+^-ATPase α-chains in SAF preparations. Based on an estimate of 10 µg PrP^Sc^ per brain, the equivalent of 0.25 µg/µl PrP^Sc^ from an ME7 (lane 1), 22F (lane 2) and 79A (lane 3) SAF preparation were resolved by SDS-PAGE, blotted onto PVDF membrane and probed with the primary antibody against (A) Apolipoprotein E (B) Total Na^+^/K^+^ ATPase α-chains using a pan α-chain antibody (C) Na^+^/K^+^ ATPase α2 isoform and (D) Na^+^/K^+^ ATPase α3 isoform. In all cases, in lanes 4 and 5 were loaded the equivalent of 0.25 µg/µl of control preparation material from uninfected WT and PrP^−/−^ mouse brains respectively. Molecular weight markers are in kDa.

In order to validate our proteomics studies, we selected two biologically interesting proteins for follow up studies, ApoE and Na^+^/K^+^-ATPase. Our decision was based on established links of both proteins to other neurodegenerative disorders in addition to the availability of antibodies. We have demonstrated by Western blotting that the SAF preparations from infected tissues contained protease-resistant PrP (Figure 1(a–c)) and we additionally performed Western blotting using an antibody against ApoE and several different antibodies that recognise various different α-chains of Na^+^/K^+^-ATPase. Representative Western blots for ApoE and Na^+^/K^+^-ATPase are shown in Figure 2(a–d). In each case we balanced the loading of SAF preparations based on semi-quantitative measures of PrP^Sc^ levels. We also included lanes containing our negative control preparations. As shown in Figure 2(a), immunoreactivity for ApoE was present as a discrete band at ∼36 kDa in all three lanes corresponding to infected SAF preparations, but not in the two mock SAF preparations from uninfected WT and PrP^−/−^ mice. The signal in the 22F SAF preparation was weak compared with ME7 and 79A preparations, but clearly present on longer exposure (data not shown). Western blotting using a pan α-chain polyclonal antibody demonstrated the presence of the Na^+^/K^+^-ATPase α-chain in all three SAF preparations from infected mice, but not in the two control preparations. Immunoreactivity was present as a single band at ∼100 kDa, consistent with previous reports and with the calculated molecular mass of Na^+^/K^+^-ATPase α-chains of ∼100–110 kDa. Since we could not distinguish whether α2- or α3-chains were selectively present from our proteomics results, we also probed blots with antibodies that specifically recognised α2- or α3-chains and typical results are shown in Figure 2(c) and (d) respectively. These data show clearly that both α2- and α3-chains are present in preparations of SAF from infected mouse brains, but not in control preparations. Combined, the results of our Western blots confirm the presence of three potentially interesting proteins in infected SAF preparations and validate our proteomics data.

### Na^+^/K^+^-ATPase enhances cell free conversion of murine recombinant PrP and acts as a potential conversion cofactor

Both α2- and α3-subunits of Na^+^/K^+^-ATPase were detected specifically in SAF preparations, by Western blotting. In brain, the α2-chain is expressed predominately in glia whilst the α3-chain is more highly enriched in neurons, however single subunits are not thought to be expressed exclusively on any particular cell type. Na^+^/K^+^-ATPase is a crucial plasma membrane bound enzyme that determines the cellular resting potential by pumping sodium ions out from and potassium ions in to cells, against their concentration gradients. Amongst other things, a stable resting potential inhibits the opening of voltage dependant calcium channels. In neurons, neurotransmitter binding causes a depolarisation of the membrane, thereby opening ion channels allowing calcium influx and activation of several important secondary functions. It is therefore perhaps unsurprising that mutations and defects in both α2- and α3-chains of Na^+^/K^+^-ATPase have been associated with neurodegenerative disorders [43]. Palladino *et al* observed that mutations in the *Drosophila* α-subunit of Na^+^/K^+^-ATPase resulted in severe neuronal damage and a marked reduction in lifespan [44]. Previous work by the same group had indicated that inhibition of the Na^+^/K^+^-ATPase caused spongiform vacuolation similar to that seen in TSE infections [45]. Furthermore, PrP^C^ has been shown to interact either directly or indirectly with Na^+^/K^+^-ATPase by a variety of techniques, including affinity chromatography [42], cross linking in tissue samples [9], [26], [42] and co-immunoprecipitation experiments [8].

Based on the above evidence, we reasoned that Na^+^/K^+^-ATPase may represent a prion protein conversion cofactor and we sourced Na^+^/K^+^-ATPase commercially to allow us to assess whether it modulated misfolding in two prion protein misfolding assays. Although commercially available Na^+^/K^+^-ATPase was isolated from pigs and our *in vitro* misfolding assays use murine recPrP, homology between porcine and murine Na^+^/K^+^-ATPase α-subunit isoforms is typically greater than 99% and we considered it unlikely that species differences would have a major impact on our results. Firstly, we used a well characterised cell free conversion assay (CFCA) [46], which uses SAF preparations as a source of PrP^Sc^ to convert recPrP into a proteinase K-resistant isoform. The newly formed PK resistant recPrP can be detected against a background of PrP^Sc^ by the prior incorporation of an antibody recognition epitope into recPrP, in this case the 3F4 monoclonal antibody epitope. The CFCA has previously been shown to replicate many aspects of TSE disease including species and polymorphism barriers [47]. The assay is also inhibited by anti-TSE drugs [46] and we have recently shown that conversion of recPrP in the assay can be enhanced by the inclusion of subcellular fractions enriched for plasma membrane [39].

**Figure 3:**
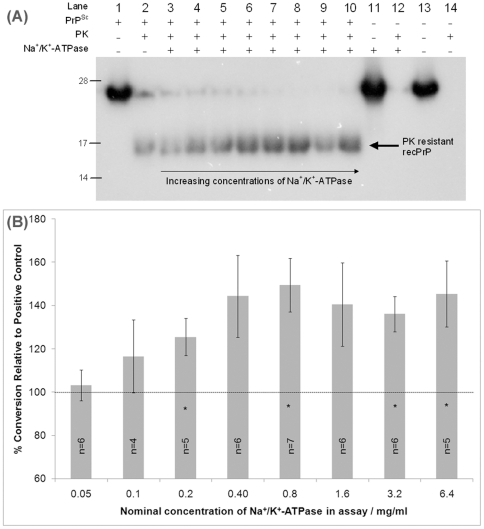
Na^+^/K^+^-ATPase enhances cell free conversion of recPrP. (A) Western blot of a typical cell free conversion assay seeded with ME7 fibrils in the presence (lanes 3–10) of Na^+^/K^+^-ATPase at increasing concentrations following protease treatment. Lanes 1 & 2 contain CFCA reactions in the absence of Na^+^/K^+^-ATPase, both without and with protease digestion, and represent the reaction to which data are normalised. Lanes 11–14 contain negative control reactions without and with protease to demonstrate that recombinant PrP does not convert spontaneously in the absence of PrP^Sc^, even in the presence of Na^+^/K^+^-ATPase. (B) Bar chart showing conversion efficiencies of recombinant PrP as a function of the amount of Na^+^/K^+^-ATPase added. Cell free conversion assays were repeated multiple times, as indicated, and percent conversion was normalised relative to control reactions containing no additive, the efficiency of which was set to 100% and indicated by the dotted line. Error bars show ± standard error of conversion efficiency for each concentration point. Bars marked with an asterisk are significantly different from controls. Nominal Na^+^/K^+^-ATPase concentrations are given based on weight of lyophilised powder and for details of the calculated Na^+^/K^+^-ATPase concentrations used see materials and methods.

Figure 3(a) shows results of a typical CFCA experiment seeded with ME7 scrapie and detected by SDS-PAGE and Western blotting. The assay contains both positive and negative control reactions as well as several reactions supplemented with increasing concentrations of Na^+^/K^+^-ATPase. In the presence of SAF but the absence of Na^+^/K^+^-ATPase, a PK resistant product is formed from recPrP (Figure 3(a) lanes 1 & 2). In the absence of SAF, no protease-resistant product is formed, even when Na^+^/K^+^-ATPase is included (lanes 11–14). In the presence of both PrP^Sc^ and Na^+^/K^+^-ATPase, the intensity of the band corresponding to protease-resistant recPrP conversion product increases with increasing amounts of Na^+^/K^+^-ATPase. We repeated the experiment multiple times and averaged the conversion efficiencies of recPrP in the presence of Na^+^/K^+^-ATPase, by setting the positive control reaction to 100% conversion. Figure 3(b) shows, graphically, the average conversion efficiencies as a function of Na^+^/K^+^-ATPase concentration and demonstrates clearly that Na^+^/K^+^-ATPase enhances production of protease resistant recPrP in our CFCA. The number of replicate experiments performed for each concentration of Na^+^/K^+^-ATPase is displayed on the bar chart. One-sample t-tests demonstrate increases in reactions incorporating 0.2, 0.8, 3.2 and 6.4 mg/ml Na^+^/K^+^-ATPase relative to control reactions are statistically significant, as indicated by asterisks on Figure 3(b). Although conversion efficiencies in reactions incorporating 0.4 and 1.6 mg/ml Na^+^/K^+^-ATPase were not statistically significant, the underlying trend of the data is towards increased conversion in reactions incorporating Na^+^/K^+^-ATPase. Moreover, statistically significant conversion efficiencies were measured at concentrations of Na^+^/K^+^-ATPase of only 0.2 mg/ml, which is ∼3 fold less than the concentration of recPrP in the assay in molar terms (see materials and methods for details), suggesting that Na^+^/K^+^-ATPase is capable of enhancing conversion even at sub-stoichiometric levels.

During commercial production, Na^+^/K^+^-ATPase is isolated by sodium iodide extraction of microsome preparations and prepared as a lyophilised powder in 90% sucrose. We assessed the effect of the sucrose carrier on cell free conversion of recPrP (Figure S1) and although calculated conversion efficiencies were variable, partly because high concentrations of sucrose cause loading problems for SDS-PAGE gels, the general trend of the data indicates that sucrose has no effect on conversion efficiency of recPrP in the CFCA. Thus, we concluded that Na^+^/K^+^-ATPase specifically enhances conversion of recPrP and may act as a prion protein conversion cofactor.

**Figure 4:**
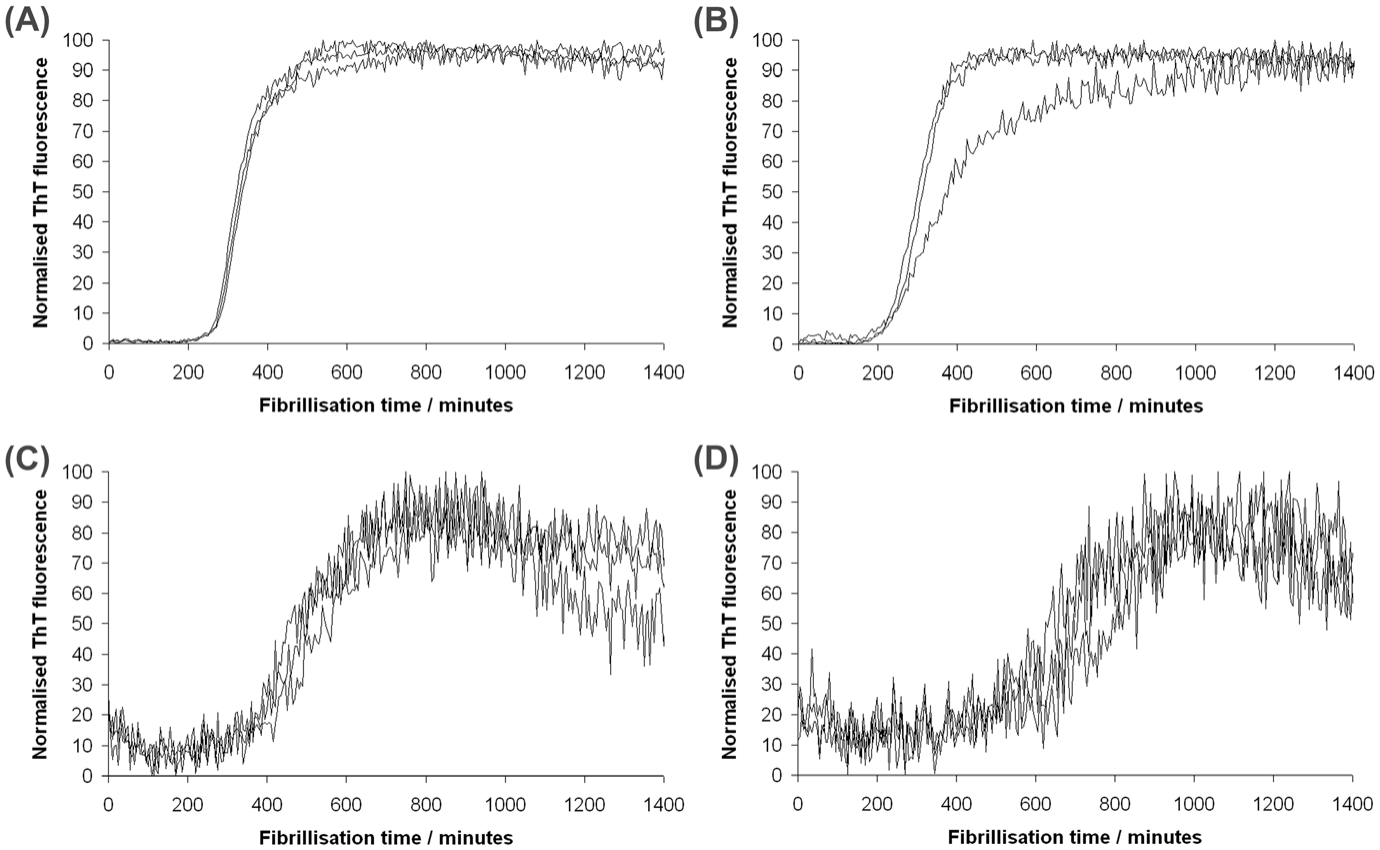
Fibrillisation of recPrP is inhibited by Na^+^/K^+^-ATPase at low stoichiometries. Murine recPrP was fibrillised in a buffer containing 2 M guandinium hydrochloride and different amounts of Na^+^/K^+^-ATPase. Reactions were performed in triplicate and fibrillisation was monitored by the increase in ThT fluorescence over time. After subtraction of background fluorescence from control wells, the resulting data were normalised to express the ThT fluorescence as a percentage of the maximum signal. (A) recPrP alone (B) final nominal concentration of 18 µg/ml Na^+^/K^+^-ATPase (C) final nominal concentration of 35 µg/ml Na^+^/K^+^-ATPase (D) final nominal concentration of 70 µg/ml Na^+^/K^+^-ATPase. For details of the calculated concentrations of Na^+^/K^+^-ATPase used see materials and methods.

We also tested the effect of Na^+^/K^+^-ATPase on the kinetics of *in vitro* fibrillisation of recombinant PrP, a misfolding pathway that is promoted by partially-denaturing concentrations of guanidine hydrochloride. Several recent findings have suggested that fibrillisation is a competing pathway to disease-specific conversion and results in structures that are of limited physiological relevance [48], [49]. Indeed, we have previously found that cellular factors promoting disease-specific misfolding also inhibit *in vitro* chaotrope-induced fibrillisation [6], [39]. We fibrillised murine recombinant PrP in the presence or absence of Na^+^/K^+^-ATPase, monitoring the extent of fibrillisation by the increase in fluorescence from thioflavin T (ThT), and typical results are shown in Figure 4. Panel (a) shows fibrillisation of wildtype murine recPrP alone. The lag time to onset of fibrillisation is ∼200 minutes and the subsequent elongation of fibrils occurs rapidly. Panels (b)–(d) show fibrillisation of recPrP in the presence of increasing amounts of Na^+^/K^+^-ATPase. We found only a modest increase in the lag time to fibrillisation when Na^+^/K^+^-ATPase was present when compared to control reactions. In previous work, we have found that dramatic changes in lag time can be achieved by subtle variations in solution conditions, but the incorporation of Na^+^/K^+^-ATPase altered lag times by only ∼100 minutes. Conversely, we found that the kinetics of the subsequent elongation phase was dramatically delayed in the presence of higher concentrations of Na^+^/K^+^-ATPase as compared to control reactions. It should be noted that the concentrations of Na^+^/K^+^-ATPase used are around 100 fold less than the molar concentration of recPrP in the fibrillisation assay indicating that inhibition occurs at exceptionally low PrP:Na^+^/K^+^-ATPase stoichiometry. For control reactions (panel (a)) it is possible to fit sigmoidal curves to the data, which allows calculations of lag times and rates of elongation. However, it was not possible accurately to fit sigmoidal curves to data obtained from reactions in the presence of Na^+^/K^+^-ATPase because the elongation phases are inhibited to such an extent, hence statistical comparisons of the effect of Na^+^/K^+^-ATPase cannot be made. We assessed the effect of sucrose, the carrier included in Na^+^/K^+^-ATPase preparations, on fibrillisation of recPrP and found that fibrillisation kinetics were not demonstrably different to controls (Figure S2). Thus, we find that Na^+^/K^+^-ATPase negatively impacts recombinant PrP fibrillisation predominately by inhibiting the elongation phase of fibrillisation, data which suggests that binding of recPrP to Na^+^/K^+^-ATPase may be a key factor mediating prion misfolding.

### Na+/K+-ATPase links prion protein function and misfolding

Combined, our findings provide one of the first links between normal PrP^C^ function and TSE disease-specific misfolding of the prion protein. The major function of PrP^C^ has remained enigmatic, despite evidence of roles in many biochemical processes, including metal ion binding and transport [50], protection against reactive oxygen species through an inherent superoxide dismutase activity [51], signal transduction [52], [53], neuroprotection [54] and regulation of calcium homeostasis [55]. However, in the absence of PrP^C^, knockout mice develop essentially normally [56] and the only major phenotypic abnormality is a neuropathy associated with demyelination of peripheral nerves, which manifests only late in life [57]. These observations contribute towards the case for PrP^C^ as a neuroprotective molecule that is important during times of acute stress, such as exposure of cells to reactive oxygen species [58], withdrawal of serum from cell cultures [59] or age of an individual [57]. Evidence to implicate PrP^C^ as a ‘specific’ neuroprotective molecule is limited [60], however, and available evidence points instead to a function for PrP^C^ in aiding the regulation of cellular homeostatic mechanisms. PrP^C^ may facilitate normal neuronal processes and a regulatory association with Na^+^/K^+^-ATPase may underlie this function, as previously suggested by Kleene *et al*. [26]. Under normal circumstances, Na^+^/K^+^-ATPase works at ∼30% of its peak pumping capacity. However, under certain conditions such as during cellular stress, the Na^+^/K^+^-ATPase needs to operate at a greater capacity since it maintains the electrical gradient required to drive other cellular processes. This includes maintaining neuronal calcium fluxes by the Na^+^/Ca^2+^ exchanger [61] or astrocytic removal of glutamate following synaptic transmissions [62]. Recent findings have also indicated a role for the Na^+^/K^+^-ATPase in signal transduction that is not linked to its enzymatic function [63], [64]. Kleene *et al* found that the lack of PrP^C^ in knockout mice results in lower efficiencies of Na^+^/K^+^-ATPase pumping without affecting resting membrane potential [26]. This suggests that the loss of PrP^C^ may preclude Na^+^/K^+^-ATPase working at full capacity when it is needed - e.g. during times of stress - resulting in many of the subtle defects in PrP-null mice [65], [66], [67], [68]. It is not yet known whether lack of PrP alters signal transduction through Na^+^/K^+^-ATPase.

In addition to a putative function role for a complex involving PrP^C^ and Na^+^/K^+^-ATPase, the presence of Na^+^/K^+^-ATPase in SAF preparations suggests that such a complex is important for PrP misfolding *in vivo*. This is evidenced by our demonstrations that Na^+^/K^+^-ATPase can modulate different misfolding pathways of PrP. Various groups have shown interactions between Na^+^/K^+^-ATPase and PrP^C^ [8], [9], [42] and there have been several suggestions that the two proteins associate to form a multiprotein complex with additional partners. One such partner is suggested to be 2′,3′-cyclic nucleotide 3′-phosphodiesterase (CNP1) [69] and interestingly this protein was identified in one of our SAF preparations and was also detected in another below the level of confidence. CNP1 is a membrane bound enzyme involved in the synthesis of the myelin sheath [70] and it has been reported that CNP1 may act as an anchor for tubulin [71]. Thus, the involvement of a PrP-ATPase-CNP1 complex in prion protein function and misfolding may explain a range of observations from our data and from that of other studies in the field.

A key question to determine the role of Na^+^/K^+^-ATPase in PrP misfolding *in vivo* is whether Na^+^/K^+^-ATPase knockout mice can sustain a prion infection, but homozygous knockout of either α2- or α3-chains result in lethality [72]. Hemizygous knockout mice (α2 or α3) exhibit a range of behavioural and physiological abnormalities [72] and, against this background, proving a direct role for Na^+^/K^+^-ATPase in prion misfolding *in vivo* may be challenging. Nevertheless, it is possible to develop a hypothetical model in which, under normal conditions, PrP^C^ participates in the regulation of the activity of Na^+^/K^+^-ATPase allowing it to increase activity on demand. During TSE disease, PrP^C^ is lost as it is converted to PrP^Sc^, thereby reducing the functional PrP^C^ available and compromising Na^+^/K^+^-ATPase enzymatic or signalling functions. In addition, Na^+^/K^+^-ATPase is sequestered from the surface of neurons and/or astrocytes into the growing fibril and this double loss of function, coupled with partial toxicity of extracellular, fibrillar deposits, may be sufficient to initiate a cascade of intracellular events that culminate in neuronal loss. Such a process could explain the requirement for PrP^C^ for neurotoxicity of oligomeric protein preparations [73] and there is significant evidence pointing to a role for electrophysiological disturbances in the toxicity of prion protein aggregates [74]. We are certainly not the first to suggest a key role for the loss of Na^+^/K^+^-ATPase function in TSE diseases [45], [75], [76] but our work is the first to demonstrate that Na^+^/K^+^-ATPase is present in SAF and that this protein can mediate misfolding of PrP.

The analysis of SAF from mouse scrapie strains by mass spectrometry has highlighted various proteins as being associated with PrP^Sc^ and raises intriguing questions over their potential roles in PrP conversion. Our finding that one of the copurifying proteins, Na^+^/K^+^-ATPase, enhances cell free conversion of recombinant PrP, suggests that this protein, potentially in association with other binding partners, may be integral to facilitating PrP conversion *in vivo*.

## Materials and Methods

### Ethics Statement and generation of infected tissue samples

All experimental protocols were approved by the ‘Institute for Animal Health Neuropathogenesis Division Ethical Review Process’ and experimentation was carried out under licences 14-79-0560 and 14-92-3745 granted by the UK Home Office. All experiments were performed accordance with the UK Home Office Regulations (Animals (Scientific Procedures) Act 1986). ME7, 22F and 79A scrapie strains were passaged in either C57bl/6 or CV mice, both of which have the *Prnp* ^a^ genotype. All mice were kept in contained conditions at The Roslin Institute mouse facility. Mice were culled when clinical signs became apparent according to approved clinical monitoring criteria, brains were immediately removed, snap frozen in liquid nitrogen and stored at −80°C until used.

### Purification of scrapie associated fibrils

Based on a method described by Hope *et al*. [27], SAF were isolated from the brains of terminally TSE infected animals. A 5% (w/v) homogenate was prepared from a pool of 4 mouse brains in a buffer containing 10% (w/v) *N*-lauroylsarcosine, 0.1 M sodium phosphate, pH 7.4. The homogenate was centrifuged at 22,000 *g* for 30 minutes at 10°C, the pellet was discarded and the supernatant centrifuged at 215,000 *g* for 150 minutes at 10°C. The pellet was re-suspended in 3 ml H_2_O followed by incubation at room temperature for 1 hour. To this, 9 ml of iodide solution (0.9 M potassium iodide, 15 mM sodium phosphate, 9 mM sodium thiosulphate, 1.5% (w/v) *N*-lauroylsarcosine, pH 8.5) was added. The suspension was centrifuged at 285,000 *g* for 90 minutes at 10°C through a cushion of 20% (w/v) sucrose in 0.6 M potassium iodide, 10 mM sodium phosphate, 6 mM sodium thiosulphate, 1% (w/v) *N*-lauroylsarcosine sodium salt, pH 8.5. The resultant pellet was washed in H_2_O and centrifuged at 13,000 *g* for 30 minutes at room temperature. The final SAF pellet was re-suspended in H_2_O to approximately 1 µg/µl by sonication, estimated based on a yield of 10 µg per mouse brain. Densitometric analysis of semi-quantitative Western blots incorporating serial dilutions of recombinant murine PrP gave a more accurate concentration and appropriate modifications were made to give 1 µg/µl final concentration.

### SDS-PAGE and Western blotting analysis of SAF preparations

For Western blots, the following primary antibodies were used: Na^+^/K^+^-ATPase pan α-chain, rabbit polyclonal (H-300, Santa Cruz Biotechnology Inc), Na^+^/K^+^-ATPase α2-chain, rabbit polyclonal (AB9094, Millipore), Na^+^/K^+^-ATPase α3-chain, goat polyclonal (Y-13, Santa Cruz Biotechnology Inc), ApoE, rabbit polyclonal (AMS Biotechnology), PrP, 8H4 and 3F4 mouse monoclonal (TSE Resource Centre, The Roslin Institute). Protein samples were separated by SDS-PAGE using 4–12% (w/v) NuPAGE Bis-Tris gradient gels (Invitrogen). Silver nitrate staining of SDS-PAGE gels was carried out according to the Plus One silver staining protocol (GE Healthcare). For Western blotting, gels were semi-dry blotted onto Immobilon-P PVDF membrane (Whatman). The membrane was probed with the appropriate primary antibody, and secondary antibody binding was detected by ECL Western blotting detection reagent (GE Healthcare). The membrane was dried and exposed to hyperfilm-MP (GE Healthcare), which was developed by a Compact Xograph X4 (Xograph Imaging Systems).

### Shotgun Liquid chromatography-tandem mass spectrometry (LC-MS/MS) of scrapie associated fibrils

SAF preparations were reduced and carboxymethylated in 6 M guanidine hydrochloride, 100 mM ammonium bicarbonate (pH 8.5) by use of 40 µM dithiothreitol and 200 µM iodoacetamide respectively, followed by precipitation at −20°C for 12 hours in 20 volumes of ethanol. The resultant pellet was resuspended in 4 M urea, 100 mM ammonium bicarbonate (pH 8.5) prior to tryptic digestion (1∶20 enzyme∶substrate ratio by weight) at 37°C for 8 hours. Tryptic peptides were concentrated using a 180 µm×20 mm, 5 µm Symmetry C18 trap (Waters) for 3 min at 10 µl/min, and resolved on a 1.7 µm, 100 µm×100 mm, BEH 130 C18 column (Waters) at 400 nl/min that was attached to a nano Acquity UPLC (Waters). Peptides were eluted over a linear gradient of 0–50% (v/v) acetonitrile, 0.1% (v/v) formic acid for 30 minutes with a flow rate of 400 nl/min. To complete the elution, the column was washed with 85% (v/v) acetonitrile, 0.1% (v/v) formic acid for 7 minutes. Ionised peptides were analysed by a quadrupole time of flight (Q-ToF) Premier mass spectrometer (Waters) in data-dependent acquisition mode where a MS survey scan was used to automatically select multicharged peptides for further MS/MS fragmentation. From each survey scan up to three peptides were selected for fragmentation. MS/MS collision energy was dependent on precursor ion mass and charge state. A reference spectrum was collected at every 30 seconds from Glu-fibrinopeptide B (785.8426 m/z), introduced *via* a reference sprayer. Raw MS/MS spectra were processed using ProteinLynx Global Server (Waters) and were searched against the IPI murine protein database by use of the Mascot search algorithm. Search parameters are given in Supplementary Materials and Methods.

### Cell free conversion assays

Details of our cell free conversion assay have previously been published [46]. Briefly, recombinant PrP incorporating a 3F4 antibody recognition epitope was produced by expression in bacteria. After bacterial lysis and inclusion body solubilisation, the protein was purified by sequential chromatographic steps of nickel ion affinity and cation exchange chromatography. Protein containing fractions were oxidized overnight, in the presence of copper ions, to form the single disulfide bond, and were extensively dialysed against 50 mM sodium acetate, pH 5.5. SAF were prepared as above from ME7 infected mouse brains and were mixed at a ratio of 5∶1 (SAF∶recPrP) with the 3F4-tagged recPrP substrate in an assay buffer supplemented with different concentrations of Na^+^/K^+^-ATPase. The final concentrations of PrP^Sc^ and recPrP used were 50 µg/ml and 10 µg/ml respectively. The mixture was incubated at 37°C for 24 hours and was subsequently treated with proteinase K, after prior removal of an aliquot equal to 1/10^th^ of the reaction mixture for use as a -PK control. All samples were separated by SDS-PAGE and PK-resistant recombinant PrP was detected by Western blotting using the 3F4 antibody, which does not detect wildtype murine PrP. Western blots were scanned by use of an ImageScanner III (GE Healthcare) and protein bands quantified by densitometric methods using ImageQuant software (GE Healthcare). We defined the control reaction, without Na^+^/K^+^-ATPase, as being 100% conversion and normalised data relative to this reaction.

### Fibrillisation of murine recombinant PrP in the presence of Na^+^/K^+^-ATPase

The methods for production of murine recombinant PrP and fibrillisation of this protein have previously been published [39] and follow those previously described by the group of Baskakov [77], [78]. Briefly, recombinant PrP was expressed by bacteria and protein was purified from inclusions bodies by immobilized metal ion affinity chromatography, size exclusion chromatography and reversed phase chromatography followed by lyophilisation. Protein, at a final concentration of 5 µM, was fibrillised in a guanidinium hydrochloride containing buffer (2 M guanidine HCl, 10 mM thiourea, 120 µg/ml recPrP, 50 mM 2-(N-morpholino)ethanesulfonic acid (MES) pH 6.0, 10 µM ThT) by shaking at 900 rpm in a 96 well plate. The extent of fibrillisation was monitored in real time by the increase in ThT fluorescence by use of a Fluoroskan Ascent (Thermo Scientific) plate reader with excitation at 444 nm and emission at 485 nm. Readings were taken every 5 minutes and the fibrillisation reaction was monitored for 24 hours.

### Addition of Na^+^/K^+^-ATPase to misfolding assays

Na^+^/K^+^-ATPase was purchased from Sigma. To determine the effect of Na^+^/K^+^-ATPase on cell free conversion of recPrP, doubling dilutions of Na^+^/K^+^-ATPase solution in conversion assay buffer were prepared from a starting concentration of 12.8 mg/ml. These were added 1∶1 to cell free conversion assays to yield nominal concentrations of 6.4 mg/ml–0.1 mg/ml Na^+^/K^+^-ATPase. However, Na^+^/K^+^-ATPase as purchased was only 10% by weight of the lyophilised powder (the balance was sucrose) hence effective concentrations of Na^+^/K^+^-ATPase were in the range 0.64–0.01 mg/ml. Assuming a combined molecular weight of the assembled Na^+^/K^+^-ATPase complex of ∼154 kDa (α-chain ∼111.5 kDa, β-chain ∼35 kDa, γ-chain ∼7.5 kDa), the final concentrations of Na^+^/K^+^-ATPase in CFCA ranged from 4 µM to 30 nM. By comparison, recPrP concentration in each CFCA was 430 nM. All cell free conversion assays were repeated at least 3 times.

To determine the affect of Na^+^/K^+^-ATPase on PrP fibrillisation, three different concentrations of enzyme were added to 3 replicate wells of a 96 well plate containing recombinant PrP in fibrillisation buffer. Using the above figures for nominal Na^+^/K^+^-ATPase concentrations and molecular weight, the final concentrations of Na^+^/K^+^-ATPase in fibrillisation reactions equated to 45.5, 22.5 and 11.5 nM. By comparison, recPrP concentration was ∼5 µM. The entire experiment was repeated three times to yield 9 independent replicates per ATPase concentration.

## Supporting Information

Figure S1 **Sucrose does not enhance cell free conversion of murine recPrP.** Cell free conversion assays used a SAF preparation from ME7 infected mice as seed and murine recombinant PrP and were performed according to the materials and methods section. Sucrose was added to the final concentrations shown. The Western blot was imaged and densitometry was used to calculate the percentage conversion of recombinant PrP for each reaction. The percent conversion of the positive control reaction was set to 100% and sucrose-containing reactions were normalised accordingly.
(DOC)

Figure S2 **Sucrose has no effect on in vitro fibrillisation of murine recPrP.** Thioflavin T fluorescent profiles during fibrillisation of murine recombinant PrP, in triplicate, in the presence of (A) no additive (B) 50 µg/ml Sucrose (C) 200 µg/ml sucrose (D) 800 µg/ml sucrose. After subtraction of background fluorescence, thioflavin t fluorescence was normalised to 0–100% for each reaction.
(DOC)

Table S1 **Full, compiled list of proteins identified in SAF preparations from infected mouse brains.** SAF were purified from the brains of mice infected with ME7, 22F or 79A strains of mouse-passaged scrapie. Duplicate preparations were made and the 6 samples were reduced and alkylated in 6 M guanidine hydrochloride prior to tryptic digestion. Tryptic peptides were separated and detected by online LC-MS/MS and the data searched against the IPI murine database by use of the Mascot search algorithm. Proteins are listed in the table if they were identified conclusively in at least one SAF preparation. Protein hits were deemed to be conclusively identified if they were above the level of significance (as determined by Mascot scores) and more than one peptide matched to the sequence. Those proteins in upper rows and italicised were found in control preparations from uninfected brains either in the current study or in Moore *et al.* nd – not detected. dbcl – protein detected but below the level of confidence because only a single peptide was matched. a – due to extensive sequence homology between different chains of these proteins it is not possible to tell whether only a single isoform or multiple isoforms are present.
(DOC)

Supplementary Mascot Searches S1 **Composite file of results from Mascot searches on LC-MS/MS data.** The file is composed of Mascot single searches of SAF derived from uninfected mice and PrP^−/−^ as well as SAF derived from 2 replicate preparations each of wild type mice infected with ME7, 22F and 79A strains of mouse passaged scrapie.
(PDF)
