# Supplementary material for: Na+/K+-ATPase Is Present in Scrapie-Associated Fibrils, Modulates PrP Misfolding In Vitro and Links PrP Function and Dysfunction
Source: PLoS One. 2011 Nov 2;6(11):e26813. doi: 10.1371/journal.pone.0026813 (PMC3206849; doi:10.1371/journal.pone.0026813)
Supplement: Figure S1 — Sucrose does not enhance cell free conversion of murine recPrP. Cell free conversion assays used a SAF preparation from ME7 infected mice as seed and murine recombinant PrP and were performed according to the materials and methods section. Sucrose was added to the final concentrations shown. The Western blot was imaged and densitometry was used to calculate the percentage conversion of recombinant PrP for each reaction. The percent conversion of the positive control reaction was set to 100% and sucrose-containing reactions were normalised accordingly. (DOC) [file pone.0026813.s001.doc]

**Na+/K+ ATPase as a prion misfolding cofactor, Graham *et al*.**

**Supplementary figure 1**

**Sucrose does not enhance cell free conversion of murine recPrP**

*Supplementary Figure 1 - Cell free conversion assays used a SAF preparation from ME7 infected mice as seed and murine recombinant PrP and were performed according to the materials and methods section. Sucrose was added to the final concentrations shown. The Western blot was imaged and densitometry was used to calculate the percentage conversion of recombinant PrP for each reaction. The percent conversion of the positive control reaction was set to 100% and sucrose-containing reactions were normalised accordingly.*
