# Supplementary material for: Na+/K+-ATPase Is Present in Scrapie-Associated Fibrils, Modulates PrP Misfolding In Vitro and Links PrP Function and Dysfunction
Source: PLoS One. 2011 Nov 2;6(11):e26813. doi: 10.1371/journal.pone.0026813 (PMC3206849; doi:10.1371/journal.pone.0026813)
Supplement: Figure S2 — Sucrose has no effect on in vitro fibrillisation of murine recPrP. Thioflavin T fluorescent profiles during fibrillisation of murine recombinant PrP, in triplicate, in the presence of (A) no additive (B) 50 µg/ml Sucrose (C) 200 µg/ml sucrose (D) 800 µg/ml sucrose. After subtraction of background fluorescence, thioflavin t fluorescence was normalised to 0–100% for each reaction. (DOC) [file pone.0026813.s002.doc]

**Na+/K+ ATPase as a prion misfolding cofactor, Graham *et al*.**

**Supplementary figure 2**

**Sucrose has no effect on *in vitro* fibrillisation of murine recPrP**


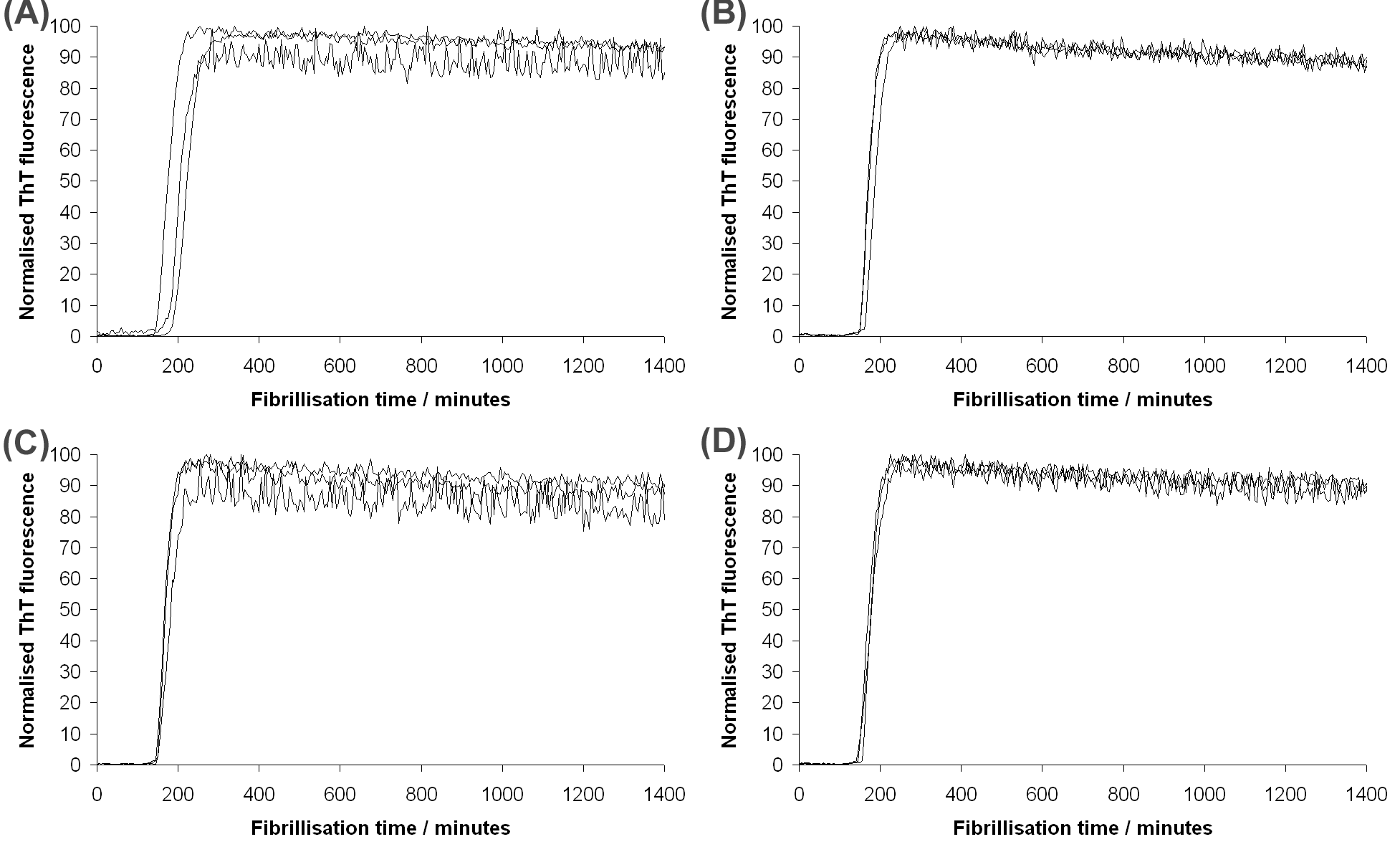


*Supplementary figure 2 – Thioflavin T fluorescent profiles during fibrillisation of murine recombinant PrP, in triplicate, in the presence of (A) no additive (B) 50 µg/ml Sucrose (C) 200 µg/ml sucrose (D) 800 µg/ml sucrose. After subtraction of background fluorescence, thioflavin t fluorescence was normalised to 0-100% for each reaction.*
