# Supplementary material for: Na+/K+-ATPase Is Present in Scrapie-Associated Fibrils, Modulates PrP Misfolding In Vitro and Links PrP Function and Dysfunction
Source: PLoS One. 2011 Nov 2;6(11):e26813. doi: 10.1371/journal.pone.0026813 (PMC3206849; doi:10.1371/journal.pone.0026813)
Supplement: Table S1 — Full, compiled list of proteins identified in SAF preparations from infected mouse brains. SAF were purified from the brains of mice infected with ME7, 22F or 79A strains of mouse-passaged scrapie. Duplicate preparations were made and the 6 samples were reduced and alkylated in 6 M guanidine hydrochloride prior to tryptic digestion. Tryptic peptides were separated and detected by online LC-MS/MS and the data searched against the IPI murine database by use of the Mascot search algorithm. Proteins are listed in the table if they were identified conclusively in at least one SAF preparation. Protein hits were deemed to be conclusively identified if they were above the level of significance (as determined by Mascot scores) and more than one peptide matched to the sequence. Those proteins in upper rows and italicised were found in control preparations from uninfected brains either in the current study or in Moore et al. nd – not detected. dbcl – protein detected but below the level of confidence because only a single peptide was matched. a – due to extensive sequence homology between different chains of these proteins it is not possible to tell whether only a single isoform or multiple isoforms are present. (DOC) [file pone.0026813.s003.doc]

| **TSE strain (code for supporting data)** | **ME7 (10881)** | **ME7 (10754) ^c^** | **22F (10882)** | **22F (10753) ^c^** | **79A (10883)** | **79A (10755) ^c^** |
| --- | --- | --- | --- | --- | --- | --- |
| **Gene and Protein Name** | **Mascot Score** (peptide matches) | **Mascot Score** (peptide matches) | **Mascot Score** (peptide matches) | **Mascot Score** (peptide matches) | **Mascot Score** (peptide matches) | **Mascot Score** (peptide matches) |
| *Camk2# Calcium/calmodulin-dependent protein kinase type II various chains ^a^* | *Max 1434 (15)* | *Max 883 (14)* | *Max 1121 (11)* | *Max 921 (14)* | *Max 3316 (16)* | *Max 371 (10)* |
| *Act# Actin, various isoforms ^a^* | *Max 175 (4)* | *Max 665 (10)* | *Max 157 (4)* | *Max 107 (6)* | *Max 234 (3)* | *Max 818 (13)* |
| *Ftl1; Ferritin light chain 1* | *nd* | *nd* | *247 (2)* | *452 (5)* | *nd* | *116 (2)* |
| *Fth1 Ferritin heavy chain* | *nd* | *nd* | *88 (2)* | *297 (3)* | *nd* | *62 (2)* |
| *Mobp Isoform 1 of Myelin-associated oligodendrocyte basic protein* | *nd* | *113 (5)* | *nd* | *71 (4)* | *nd* | *41 (2)* |
| *Vcan Isoform V0 of Versican core protein* | *nd* | *100 (3)* | *nd* | *123 (2)* | *dbcl* | *nd* |
| Tubb# Tubulin β-chains, various a | Max 259 (7) | Max 392 (11) | Max 415 (6) | Max 504 (8) | Max 246 (6) | Max 656 (12) |
| Gnao1 Isoform Alpha-2 of Guanine nucleotide-binding protein G(o) subunit alpha a | 227 (3) | 278 (4) | 70 (3) | 212 (3) | 208 (2) | 367 (7) |
| Apoe Apolipoprotein E | 226 (9) | 92 (6) | nd | nd | 147 (5) | 67 (4) |
| Atp1a3 Na+/K+ ATPase subunits α3 / α2 a | 171 (3) | 312 (5) | 198 (4) | 130 (3) | dbcl | 397 (8) |
| Prnp Major prion protein | 168 (4) | 236 (5) | 41 (2) | nd | 70 (3) | 282 (5) |
| Srrm2 Serine arginine repetitive matrix protein 2 | 134 (4) | nd | nd | nd | nd | nd |
| H1f0 Histone H1.0 | 102 (3) | nd | nd | 53 (2) | nd | nd |
| Hspa8 Heat shock cognate 71 kDa protein | 79 (3) | 52 (2) | nd | nd | nd | 136 (6) |
| Tbc1d10b TBC1 domain family, member 10b | 76 (2) | nd | nd | nd | nd | nd |
| Nsf Vesicle-fusing ATPase | 67 (2) | 37 (2) | nd | nd | nd | dbcl |
| Ckb Creatine kinase B-type | 56 (3) | 341 (5) | 160 (3) | dbcl | dbcl | 170 (5) |
| Lrp1 Pro low-density lipoprotein receptor-related protein 1 | 37 (2) | nd | nd | nd | 88 (3) | 170 (3) |
| Tuba# Tubulin α-chains, various a | nd | 246 (6) | 288 (3) | 341 (6) | nd | Max 427 (9) |
| LOC100039214; similar to Glyceraldehyde-3-phosphate dehydrogenase isoform 1 | dbcl | 186 (3) | 78 (3) | 99 (2) | dbcl | 250 (7) |
| Dlg4 Isoform 2 of Disks large homolog 4 | nd | nd | 65 (2) | nd | dbcl | nd |
| Ubb;Ubiquitin | dbcl | dbcl | dbcl | 171 (2) | nd | 158 (3) |
| Cnp Isoform CNPII of 2',3'-cyclic-nucleotide 3'-phosphodiesterase | nd | nd | nd | nd | dbcl | 159 (3) |
| Syn1 Isoform Ib / Syn2 isoform IIb of Synapsin1 | nd | nd | nd | nd | nd | 94 (4) / 47 (2) |
| Dnm1 Isoform 1 of Dynamin 1 | nd | nd | nd | nd | nd | 66 (2) |
| Eno1 Alpha Enolase | nd | nd | nd | nd | nd | 60 (2) |

Supplementary Table S1 - SAF were purified from the brains of mice infected with ME7, 22F or 79A strains of mouse-passaged scrapie. Duplicate preparations were made and the 6 samples were reduced and alkylated in 6 M guanidine hydrochloride prior to tryptic digestion. Tryptic peptides were separated and detected by online LC-MS/MS and the data searched against the IPI murine database by use of the Mascot search algorithm. Proteins are listed in the table if they were identified conclusively in at least one SAF preparation. Protein hits were deemed to be conclusively identified if they were above the level of significance (as determined by Mascot scores) and more than one peptide matched to the sequence. Those proteins in upper rows and italicised were found in control preparations from uninfected brains either in the current study or in Moore *et al.* nd – not detected. dbcl – protein detected but below the level of confidence because only a single peptide was matched. a – due to extensive sequence homology between different chains of these proteins it is not possible to tell whether only a single isoform or multiple isoforms are present.
