# Supplementary material for: Na+/K+-ATPase Is Present in Scrapie-Associated Fibrils, Modulates PrP Misfolding In Vitro and Links PrP Function and Dysfunction
Source: PLoS One. 2011 Nov 2;6(11):e26813. doi: 10.1371/journal.pone.0026813 (PMC3206849; doi:10.1371/journal.pone.0026813)

Mascot Search Results

```
User          : Dominic
Email         : dominic.kurian@bbsrc.ac.uk
Search title  : IPI Mouse_15-02-2010
MS data file  : D:\Peaklist\Dominic Kurian\QTofDK20010806 GATC P 0010884.pkl
Database      : IPI_mouse MOUSE_v3_79 (54943 sequences; 24917557 residues)
Timestamp     : 15 Feb 2011 at 15:26:16 GMT
Protein hits  : IPI00420725 Tax_Id=10090 Gene_Symbol=Camk2a Uncharacterized protein
                IPI00875723 Tax_Id=10090 Gene_Symbol=Camk2b 68 kDa protein
                IPI00112584 Tax_Id=10090 Gene_Symbol=Camk2d Isoform 1 of Calcium/calmodulin-dependent protein kinase type II subunit
                IPI00608020 Tax_Id=10090 Gene_Symbol=Ftl1 ferritin light chain 1
                IPI00347110 Tax_Id=10090 Gene_Symbol=Krt73 Keratin, type II cytoskeletal 73
                IPI00625729 Tax_Id=10090 Gene_Symbol=Krt1 Keratin, type II cytoskeletal 1
                IPI00222228 Tax_Id=10090 Gene_Symbol=4732456N10Rik hypothetical protein LOC239673
                IPI00138892 Tax_Id=10090 Gene_Symbol=Uba52 Ubiquitin-60S ribosomal protein L40
                IPI00874682 Tax_Id=10090 Gene_Symbol=- 36 kDa protein
                IPI00223714 Tax_Id=10090 Gene_Symbol=Hist1h1e Histone H1.4
```

	IPI_mouse	Decoy	False discovery rate
Peptide matches above identity threshold	65	0	0.00 %
Peptide matches above homology or identity threshold	75	0	0.00 %

Probability Based Mowse Score

Ions score is -10*Log(P), where P is the probability that the observed match is a random event.
Individual ions scores > 37 indicate identity or extensive homology (p<0.01).
Protein scores are derived from ions scores as a non-probabilistic basis for ranking protein hits.

Score Distribution

Peptide Summary Report

Format As: Peptide Summary   Help

Significance threshold p< 0.01   Max. number of hits AUTO

Standard scoring ○ MudPIT scoring ● Ions score or expect cut-off 0   Show sub-sets 0

Show pop-ups ● Suppress pop-ups ○ Sort unassigned Decreasing Score   Require bold red ☐

Select All | Select None | Search Selected | ☐ **Error tolerant** | Archive Report

1. IPI00420725 **Mass:** 55882 **Score:** 1043 **Queries matched:** 83 **emPAI:** 0.33
Tax_Id=10090 Gene_Symbol=Camk2a Uncharacterized protein
☐ Check to include this hit in error tolerant search or archive report

	Query	Observed	Mr(expt)	Mr(calc)	ppm	Miss	Score	Expect	Rank	Peptide
☑	105	463.2481	924.4816	924.4777	4.26	1	2	39	1	R.DHQKLER.E
	183	574.3185	1146.6224	1146.6431	-17.98	0	(0)	75	3	K.GAILTTMLATR.N
☑	184	574.3212	1146.6278	1146.6431	-13.27	0	(39)	0.0084	1	K.GAILTTMLATR.N
☑	190	574.3264	1146.6382	1146.6431	-4.20	0	(35)	0.022	1	K.GAILTTMLATR.N
☑	191	574.3266	1146.6386	1146.6431	-3.85	0	(36)	0.016	1	K.GAILTTMLATR.N
☑	192	574.3272	1146.6398	1146.6431	-2.81	0	(42)	0.0034	1	K.GAILTTMLATR.N
☑	193	574.3276	1146.6406	1146.6431	-2.11	0	(51)	0.00041	1	K.GAILTTMLATR.N
☑	195	574.3278	1146.6410	1146.6431	-1.76	0	(47)	0.0012	1	K.GAILTTMLATR.N
☑	197	574.3286	1146.6426	1146.6431	-0.36	0	(12)	3.8	1	K.GAILTTMLATR.N
☑	198	574.3288	1146.6430	1146.6431	-0.02	0	(42)	0.0031	1	K.GAILTTMLATR.N
☑	199	574.3290	1146.6434	1146.6431	0.33	0	(54)	0.0002	1	K.GAILTTMLATR.N
☑	200	574.3296	1146.6446	1146.6431	1.38	0	64	2.3e-005	1	K.GAILTTMLATR.N
☑	201	574.3298	1146.6450	1146.6431	1.73	0	(45)	0.0018	1	K.GAILTTMLATR.N
☑	202	574.3300	1146.6454	1146.6431	2.08	0	(64)	2.2e-005	1	K.GAILTTMLATR.N
☑	203	574.3303	1146.6460	1146.6431	2.60	0	(45)	0.0017	1	K.GAILTTMLATR.N
☑	204	574.3303	1146.6460	1146.6431	2.60	0	(25)	0.18	1	K.GAILTTMLATR.N
☑	205	574.3306	1146.6466	1146.6431	3.12	0	(39)	0.0056	1	K.GAILTTMLATR.N
☑	207	574.3308	1146.6470	1146.6431	3.47	0	(48)	0.00072	1	K.GAILTTMLATR.N
☑	208	574.3308	1146.6470	1146.6431	3.47	0	(50)	0.00047	1	K.GAILTTMLATR.N
☑	209	574.3309	1146.6472	1146.6431	3.65	0	(42)	0.0033	1	K.GAILTTMLATR.N
☑	212	574.3314	1146.6482	1146.6431	4.52	0	(35)	0.014	1	K.GAILTTMLATR.N
☑	213	574.3317	1146.6488	1146.6431	5.04	0	(31)	0.035	1	K.GAILTTMLATR.N
☑	214	574.3324	1146.6502	1146.6431	6.26	0	(26)	0.12	1	K.GAILTTMLATR.N
☑	242	631.2996	1260.5846	1260.5927	-6.42	0	(46)	0.0014	1	R.FYFENLWSR.N
☑	243	631.3040	1260.5934	1260.5927	0.56	0	(48)	0.001	1	R.FYFENLWSR.N
☑	244	631.3043	1260.5940	1260.5927	1.04	0	57	0.00013	1	R.FYFENLWSR.N

☑	246	631.3052	1260.5958	1260.5927	2.46	0	(23)	0.31	1	R.FYFENLWSR.N
☑	247	631.3057	1260.5968	1260.5927	3.26	0	(42)	0.0042	1	R.FYFENLWSR.N
☑	248	631.3059	1260.5972	1260.5927	3.57	0	(48)	0.001	1	R.FYFENLWSR.N
☑	249	631.3065	1260.5984	1260.5927	4.53	0	(4)	31	1	R.FYFENLWSR.N
☑	250	631.3067	1260.5988	1260.5927	4.84	0	(44)	0.0027	1	R.FYFENLWSR.N
☑	273	652.3435	1302.6724	1302.6932	-15.92	0	(8)	12	1	R.ITQYLDAGGIPR.T
☑	274	652.3566	1302.6986	1302.6932	4.19	0	(53)	0.00039	1	R.ITQYLDAGGIPR.T
☑	275	652.3569	1302.6992	1302.6932	4.65	0	(39)	0.0089	1	R.ITQYLDAGGIPR.T
☑	276	652.3579	1302.7012	1302.6932	6.18	0	59	8e-005	1	R.ITQYLDAGGIPR.T
☑	277	652.3600	1302.7054	1302.6932	9.41	0	(55)	0.0002	1	R.ITQYLDAGGIPR.T
	297	670.8989	1339.7832	1339.7711	9.09	0	2	19	6	R.DLKPENLLLASK.L
☑	491	816.8908	1631.7670	1631.7719	-2.96	0	(36)	0.014	1	R.FTEEYQLFEELGK.G
☑	492	816.8912	1631.7678	1631.7719	-2.47	0	(47)	0.0011	1	R.FTEEYQLFEELGK.G
☑	494	816.8917	1631.7688	1631.7719	-1.86	0	(52)	0.00042	1	R.FTEEYQLFEELGK.G
☑	495	816.8923	1631.7700	1631.7719	-1.12	0	(31)	0.046	1	R.FTEEYQLFEELGK.G
☑	496	816.8926	1631.7706	1631.7719	-0.76	0	(44)	0.0028	1	R.FTEEYQLFEELGK.G
☑	497	816.8933	1631.7720	1631.7719	0.10	0	(54)	0.00023	1	R.FTEEYQLFEELGK.G
☑	498	816.8933	1631.7720	1631.7719	0.10	0	(37)	0.011	1	R.FTEEYQLFEELGK.G
☑	499	816.8934	1631.7722	1631.7719	0.22	0	(54)	0.00027	1	R.FTEEYQLFEELGK.G
☑	500	816.8934	1631.7722	1631.7719	0.22	0	(27)	0.13	1	R.FTEEYQLFEELGK.G
☑	502	816.8936	1631.7726	1631.7719	0.47	0	(70)	5.6e-006	1	R.FTEEYQLFEELGK.G
☑	503	816.8936	1631.7726	1631.7719	0.47	0	(55)	0.00021	1	R.FTEEYQLFEELGK.G
☑	504	816.8936	1631.7726	1631.7719	0.47	0	(27)	0.12	1	R.FTEEYQLFEELGK.G
☑	505	816.8938	1631.7730	1631.7719	0.72	0	(73)	2.8e-006	1	R.FTEEYQLFEELGK.G
☑	506	816.8939	1631.7732	1631.7719	0.84	0	74	2.8e-006	1	R.FTEEYQLFEELGK.G
☑	507	816.8943	1631.7740	1631.7719	1.33	0	(59)	8.2e-005	1	R.FTEEYQLFEELGK.G
☑	509	816.8944	1631.7742	1631.7719	1.45	0	(54)	0.00027	1	R.FTEEYQLFEELGK.G
☑	510	816.8944	1631.7742	1631.7719	1.45	0	(56)	0.00014	1	R.FTEEYQLFEELGK.G
☑	511	816.8944	1631.7742	1631.7719	1.45	0	(63)	3.2e-005	1	R.FTEEYQLFEELGK.G
☑	512	816.8945	1631.7744	1631.7719	1.57	0	(62)	4.3e-005	1	R.FTEEYQLFEELGK.G
☑	513	816.8946	1631.7746	1631.7719	1.70	0	(63)	3.1e-005	1	R.FTEEYQLFEELGK.G
☑	515	816.8948	1631.7750	1631.7719	1.94	0	(51)	0.00047	1	R.FTEEYQLFEELGK.G
☑	516	816.8951	1631.7756	1631.7719	2.31	0	(48)	0.00096	1	R.FTEEYQLFEELGK.G
☑	517	816.8954	1631.7762	1631.7719	2.68	0	(71)	5.3e-006	1	R.FTEEYQLFEELGK.G
☑	518	816.8955	1631.7764	1631.7719	2.80	0	(37)	0.014	1	R.FTEEYQLFEELGK.G
☑	519	816.8956	1631.7766	1631.7719	2.92	0	(9)	8	1	R.FTEEYQLFEELGK.G
☑	520	816.8956	1631.7766	1631.7719	2.92	0	(22)	0.43	1	R.FTEEYQLFEELGK.G
☑	523	816.8964	1631.7782	1631.7719	3.90	0	(20)	0.61	1	R.FTEEYQLFEELGK.G
☑	524	816.8965	1631.7784	1631.7719	4.02	0	(48)	0.00099	1	R.FTEEYQLFEELGK.G
☑	525	816.8968	1631.7790	1631.7719	4.39	0	(50)	0.00064	1	R.FTEEYQLFEELGK.G
☑	526	816.8972	1631.7798	1631.7719	4.88	0	(3)	35	1	R.FTEEYQLFEELGK.G
☑	527	816.8974	1631.7802	1631.7719	5.13	0	(57)	0.00014	1	R.FTEEYQLFEELGK.G
☑	528	816.8977	1631.7808	1631.7719	5.50	0	(2)	44	1	R.FTEEYQLFEELGK.G
☑	529	816.8980	1631.7814	1631.7719	5.86	0	(41)	0.005	1	R.FTEEYQLFEELGK.G
☑	530	816.8983	1631.7820	1631.7719	6.23	0	(20)	0.63	1	R.FTEEYQLFEELGK.G
☑	531	816.8991	1631.7836	1631.7719	7.21	0	(63)	3.1e-005	1	R.FTEEYQLFEELGK.G
	532	816.8991	1631.7836	1631.7719	7.21	0	(1)	57	4	R.FTEEYQLFEELGK.G
☑	533	816.8996	1631.7846	1631.7719	7.82	0	(52)	0.00037	1	R.FTEEYQLFEELGK.G
☑	534	816.9014	1631.7882	1631.7719	10.0	0	(41)	0.0058	1	R.FTEEYQLFEELGK.G
☑	535	816.9016	1631.7886	1631.7719	10.3	0	(38)	0.011	1	R.FTEEYQLFEELGK.G
☑	598	1040.9820	2079.9494	2079.9426	3.32	0	32	0.027	1	K.AGAYDFPSPEWDTVTPEAK.D
☑	599	1040.9830	2079.9514	2079.9426	4.28	0	(9)	6	1	K.AGAYDFPSPEWDTVTPEAK.D
☑	602	1040.9895	2079.9644	2079.9426	10.5	0	(17)	1.1	1	K.AGAYDFPSPEWDTVTPEAK.D
☑	623	715.3424	2143.0054	2143.0321	-12.46	0	(14)	1.7	1	K.VTEQLIEAISNGDFESYTK.M
☑	625	715.3448	2143.0126	2143.0321	-9.10	0	(43)	0.0023	1	K.VTEQLIEAISNGDFESYTK.M
☑	626	715.3478	2143.0216	2143.0321	-4.90	0	63	2.4e-005	1	K.VTEQLIEAISNGDFESYTK.M
☑	629	1072.5308	2143.0470	2143.0321	6.99	0	(22)	0.35	1	K.VTEQLIEAISNGDFESYTK.M

Proteins matching the same set of peptides:
IPI00621806 **Mass:** 54651 **Score:** 1043 **Queries matched:** 83
Tax_Id=10090 Gene_Symbol=Camk2a Isoform Alpha CaMKII of Calcium/calmodulin-dependent protein kinase type II subunit alpha

---

2. IPI00875723 **Mass:** 68686 **Score:** 313 **Queries matched:** 44 **emPAI:** 0.32
Tax_Id=10090 Gene_Symbol=Camk2b 68 kDa protein
☐ Check to include this hit in error tolerant search or archive report

Query	Observed	Mr(expt)	Mr(calc)	ppm	Miss	Score	Expect	Rank	Peptide
105	463.2481	924.4816	924.4777	4.26	1	2	39	1	R.DHQKLER.E
183	574.3185	1146.6224	1146.6431	-17.98	0	(0)	75	3	K.GAILTTMLATR.N
184	574.3212	1146.6278	1146.6431	-13.27	0	(39)	0.0084	1	K.GAILTTMLATR.N
190	574.3264	1146.6382	1146.6431	-4.20	0	(35)	0.022	1	K.GAILTTMLATR.N
191	574.3266	1146.6386	1146.6431	-3.85	0	(36)	0.016	1	K.GAILTTMLATR.N
192	574.3272	1146.6398	1146.6431	-2.81	0	(42)	0.0034	1	K.GAILTTMLATR.N
193	574.3276	1146.6406	1146.6431	-2.11	0	(51)	0.00041	1	K.GAILTTMLATR.N
195	574.3278	1146.6410	1146.6431	-1.76	0	(47)	0.0012	1	K.GAILTTMLATR.N
197	574.3286	1146.6426	1146.6431	-0.36	0	(12)	3.8	1	K.GAILTTMLATR.N
198	574.3288	1146.6430	1146.6431	-0.02	0	(42)	0.0031	1	K.GAILTTMLATR.N
199	574.3290	1146.6434	1146.6431	0.33	0	(54)	0.0002	1	K.GAILTTMLATR.N
200	574.3296	1146.6446	1146.6431	1.38	0	64	2.3e-005	1	K.GAILTTMLATR.N
201	574.3298	1146.6450	1146.6431	1.73	0	(45)	0.0018	1	K.GAILTTMLATR.N
202	574.3300	1146.6454	1146.6431	2.08	0	(64)	2.2e-005	1	K.GAILTTMLATR.N
203	574.3303	1146.6460	1146.6431	2.60	0	(45)	0.0017	1	K.GAILTTMLATR.N
204	574.3303	1146.6460	1146.6431	2.60	0	(25)	0.18	1	K.GAILTTMLATR.N

	205	574.3306	1146.6466	1146.6431	3.12	0	(39)	0.0056	1	K.GAILTTMLATR.N
	207	574.3308	1146.6470	1146.6431	3.47	0	(48)	0.00072	1	K.GAILTTMLATR.N
	208	574.3308	1146.6470	1146.6431	3.47	0	(50)	0.00047	1	K.GAILTTMLATR.N
	209	574.3309	1146.6472	1146.6431	3.65	0	(42)	0.0033	1	K.GAILTTMLATR.N
	212	574.3314	1146.6482	1146.6431	4.52	0	(35)	0.014	1	K.GAILTTMLATR.N
	213	574.3317	1146.6488	1146.6431	5.04	0	(31)	0.035	1	K.GAILTTMLATR.N
	214	574.3324	1146.6502	1146.6431	6.26	0	(26)	0.12	1	K.GAILTTMLATR.N
☑	217	**579.2984**	**1156.5822**	**1156.6313**	**-42.37**	**1**	**14**	**3.3**	**1**	**R.VAAVPASGSRSR.R**
	297	670.8989	1339.7832	1339.7711	9.09	0	2	19	6	R.DLKPENLLLASK.C
☑	309	**458.5792**	**1372.7158**	**1372.7310**	**-11.12**	**1**	**38**	**0.011**	**1**	**K.KADGVKPQTNSTK.N**
☑	310	**458.5814**	**1372.7224**	**1372.7310**	**-6.31**	**1**	**(25)**	**0.25**	**1**	**K.KADGVKPQTNSTK.N**
☑	481	**810.8718**	**1619.7290**	**1619.7355**	**-3.99**	**0**	**(18)**	**0.69**	**1**	**R.FTDEYQLYEDIGK.G**
☑	482	**810.8729**	**1619.7312**	**1619.7355**	**-2.63**	**0**	**(41)**	**0.0039**	**1**	**R.FTDEYQLYEDIGK.G**
☑	483	**810.8740**	**1619.7334**	**1619.7355**	**-1.27**	**0**	**52**	**0.00033**	**1**	**R.FTDEYQLYEDIGK.G**
☑	485	**810.8782**	**1619.7418**	**1619.7355**	**3.92**	**0**	**(3)**	**24**	**1**	**R.FTDEYQLYEDIGK.G**
☑	487	**810.8815**	**1619.7484**	**1619.7355**	**7.99**	**0**	**(7)**	**10**	**1**	**R.FTDEYQLYEDIGK.G**
	571	**897.9096**	**1793.8046**	**1793.8652**	**-33.75**	**0**	**0**	**42**	**10**	**R.TPSPIATAMATTVTCTR.F + Oxidation (M)**
	598	1040.9820	2079.9494	2079.9426	3.32	0	32	0.027	1	K.AGAYDFPSPEWDTVTPEAK.N
	599	1040.9830	2079.9514	2079.9426	4.28	0	(9)	6	1	K.AGAYDFPSPEWDTVTPEAK.N
	602	1040.9895	2079.9644	2079.9426	10.5	0	(17)	1.1	1	K.AGAYDFPSPEWDTVTPEAK.N
☑	604	**704.9893**	**2111.9461**	**2112.0011**	**-26.05**	**0**	**(14)**	**1.4**	**1**	**K.TTEQLIEAVNNGDFEAYAK.I**
☑	607	**705.0047**	**2111.9923**	**2112.0011**	**-4.18**	**0**	**29**	**0.061**	**1**	**K.TTEQLIEAVNNGDFEAYAK.I**
	609	**1057.0087**	**2112.0028**	**2112.0011**	**0.83**	**0**	**(3)**	**22**	**3**	**K.TTEQLIEAVNNGDFEAYAK.I**
☑	674	**785.0440**	**2352.1102**	**2352.1301**	**-8.48**	**0**	**(66)**	**9.2e-006**	**1**	**R.QTTAPATMSTAASGTTMGLVEQAK.S**
☑	675	**785.0443**	**2352.1111**	**2352.1301**	**-8.09**	**0**	**85**	**1.4e-007**	**1**	**R.QTTAPATMSTAASGTTMGLVEQAK.S**
☑	677	**1177.0797**	**2352.1448**	**2352.1301**	**6.26**	**0**	**(30)**	**0.045**	**1**	**R.QTTAPATMSTAASGTTMGLVEQAK.S**
☑	678	**1177.0834**	**2352.1522**	**2352.1301**	**9.41**	**0**	**(45)**	**0.0013**	**1**	**R.QTTAPATMSTAASGTTMGLVEQAK.S**
	680	**1177.0856**	**2352.1566**	**2352.1301**	**11.3**	**0**	**(2)**	**28**	**3**	**R.QTTAPATMSTAASGTTMGLVEQAK.S**

---

3. IPI00112584 **Mass:** 56961 **Score:** 198 **Queries matched:** 30 **emPAI:** 0.12
Tax_Id=10090 Gene_Symbol=Camk2d Isoform 1 of Calcium/calmodulin-dependent protein kinase type II subunit delta
☐ Check to include this hit in error tolerant search or archive report

	Query	Observed	Mr(expt)	Mr(calc)	ppm	Miss	Score	Expect	Rank	Peptide
	105	463.2481	924.4816	924.4777	4.26	1	2	39	1	R.DHQKLER.E
	183	574.3185	1146.6224	1146.6431	-17.98	0	(0)	75	3	K.GAILTTMLATR.N
	184	574.3212	1146.6278	1146.6431	-13.27	0	(39)	0.0084	1	K.GAILTTMLATR.N
	190	574.3264	1146.6382	1146.6431	-4.20	0	(35)	0.022	1	K.GAILTTMLATR.N
	191	574.3266	1146.6386	1146.6431	-3.85	0	(36)	0.016	1	K.GAILTTMLATR.N
	192	574.3272	1146.6398	1146.6431	-2.81	0	(42)	0.0034	1	K.GAILTTMLATR.N
	193	574.3276	1146.6406	1146.6431	-2.11	0	(51)	0.00041	1	K.GAILTTMLATR.N
	195	574.3278	1146.6410	1146.6431	-1.76	0	(47)	0.0012	1	K.GAILTTMLATR.N
	197	574.3286	1146.6426	1146.6431	-0.36	0	(12)	3.8	1	K.GAILTTMLATR.N
	198	574.3288	1146.6430	1146.6431	-0.02	0	(42)	0.0031	1	K.GAILTTMLATR.N
	199	574.3290	1146.6434	1146.6431	0.33	0	(54)	0.0002	1	K.GAILTTMLATR.N
	200	574.3296	1146.6446	1146.6431	1.38	0	64	2.3e-005	1	K.GAILTTMLATR.N
	201	574.3298	1146.6450	1146.6431	1.73	0	(45)	0.0018	1	K.GAILTTMLATR.N
	202	574.3300	1146.6454	1146.6431	2.08	0	(64)	2.2e-005	1	K.GAILTTMLATR.N
	203	574.3303	1146.6460	1146.6431	2.60	0	(45)	0.0017	1	K.GAILTTMLATR.N
	204	574.3303	1146.6460	1146.6431	2.60	0	(25)	0.18	1	K.GAILTTMLATR.N
	205	574.3306	1146.6466	1146.6431	3.12	0	(39)	0.0056	1	K.GAILTTMLATR.N
	207	574.3308	1146.6470	1146.6431	3.47	0	(48)	0.00072	1	K.GAILTTMLATR.N
	208	574.3308	1146.6470	1146.6431	3.47	0	(50)	0.00047	1	K.GAILTTMLATR.N
	209	574.3309	1146.6472	1146.6431	3.65	0	(42)	0.0033	1	K.GAILTTMLATR.N
	212	574.3314	1146.6482	1146.6431	4.52	0	(35)	0.014	1	K.GAILTTMLATR.N
	213	574.3317	1146.6488	1146.6431	5.04	0	(31)	0.035	1	K.GAILTTMLATR.N
	214	574.3324	1146.6502	1146.6431	6.26	0	(26)	0.12	1	K.GAILTTMLATR.N
	297	670.8989	1339.7832	1339.7711	9.09	0	2	19	6	R.DLKPENLLLASK.S
☑	472	**809.8823**	**1617.7500**	**1617.7562**	**-3.83**	**0**	**(2)**	**34**	**1**	**R.FTDEYQLFEELGK.G**
☑	473	**809.8842**	**1617.7538**	**1617.7562**	**-1.48**	**0**	**37**	**0.0098**	**1**	**R.FTDEYQLFEELGK.G**
☑	475	**809.8906**	**1617.7666**	**1617.7562**	**6.43**	**0**	**(35)**	**0.021**	**1**	**R.FTDEYQLFEELGK.G**
	598	1040.9820	2079.9494	2079.9426	3.32	0	32	0.027	1	K.AGAYDFPSPEWDTVTPEAK.D
	599	1040.9830	2079.9514	2079.9426	4.28	0	(9)	6	1	K.AGAYDFPSPEWDTVTPEAK.D
	602	1040.9895	2079.9644	2079.9426	10.5	0	(17)	1.1	1	K.AGAYDFPSPEWDTVTPEAK.D

Proteins matching the same set of peptides:
IPI00406790 **Mass:** 60593 **Score:** 198 **Queries matched:** 30
Tax_Id=10090 Gene_Symbol=Camk2d Isoform 4 of Calcium/calmodulin-dependent protein kinase type II subunit delta
IPI00475044 **Mass:** 55899 **Score:** 198 **Queries matched:** 30
Tax_Id=10090 Gene_Symbol=Camk2d Isoform 2 of Calcium/calmodulin-dependent protein kinase type II subunit delta
IPI00828919 **Mass:** 58492 **Score:** 198 **Queries matched:** 30
Tax_Id=10090 Gene_Symbol=Camk2d Isoform 3 of Calcium/calmodulin-dependent protein kinase type II subunit delta
IPI00857865 **Mass:** 54664 **Score:** 198 **Queries matched:** 30
Tax_Id=10090 Gene_Symbol=Camk2d Putative uncharacterized protein
IPI00858128 **Mass:** 58283 **Score:** 198 **Queries matched:** 30
Tax_Id=10090 Gene_Symbol=Camk2d calcium/calmodulin-dependent protein kinase type II subunit delta isoform 1
IPI00858144 **Mass:** 55828 **Score:** 198 **Queries matched:** 30
Tax_Id=10090 Gene_Symbol=Camk2d 55 kDa protein

---

4. IPI00608020 **Mass:** 20817 **Score:** 71 **Queries matched:** 5 **emPAI:** 0.16
Tax_Id=10090 Gene_Symbol=Ftl1 ferritin light chain 1
☐ Check to include this hit in error tolerant search or archive report

	Query	Observed	Mr(expt)	Mr(calc)	ppm	Miss	Score	Expect	Rank	Peptide
☑	133	**517.7672**	**1033.5198**	**1033.5192**	**0.59**	**0**	**(6)**	**24**	**1**	**R.LLEFQNDR.G**
☑	134	**517.7681**	**1033.5216**	**1033.5192**	**2.33**	**0**	**17**	**2.1**	**1**	**R.LLEFQNDR.G**

☑	135	517.7722	1033.5298	1033.5192	10.3	0	(9)	11	1	R.LLEFQNDR.G
☑	682	791.7310	2372.1712	2372.1761	-2.07	0	(40)	0.0043	1	R.VAGPQPAQTGAPQGSLGEYLFER.L
☑	683	791.7352	2372.1838	2372.1761	3.25	0	54	0.00015	1	R.VAGPQPAQTGAPQGSLGEYLFER.L

Proteins matching the same set of peptides:
IPI00625129 **Mass:** 20783 **Score:** 71 **Queries matched:** 5
Tax_Id=10090 Gene_Symbol=Ftl2 ferritin light chain 2
IPI00762203 **Mass:** 20847 **Score:** 71 **Queries matched:** 5
Tax_Id=10090 Gene_Symbol=Ftl1 Ferritin light chain 1

---

5. IPI00347110 **Mass:** 59502 **Score:** 69 **Queries matched:** 4 **emPAI:** 0.11
Tax_Id=10090 Gene_Symbol=Krt73 Keratin, type II cytoskeletal 73
☐ Check to include this hit in error tolerant search or archive report

	Query	Observed	Mr(expt)	Mr(calc)	ppm	Miss	Score	Expect	Rank	Peptide
☑	263	639.3532	1276.6918	1276.7027	-8.47	0	(33)	0.036	1	K.LALDIEIATYR.K
☑	264	639.3586	1276.7026	1276.7027	-0.01	0	40	0.0068	1	K.LALDIEIATYR.K
☑	354	738.3995	1474.7844	1474.7780	4.38	0	(22)	0.4	1	R.FLEQQNQVLQTK.W
☑	355	738.4022	1474.7898	1474.7780	8.04	0	55	0.0002	1	R.FLEQQNQVLQTK.W

---

6. IPI00625729 **Mass:** 66079 **Score:** 55 **Queries matched:** 3 **emPAI:** 0.05
Tax_Id=10090 Gene_Symbol=Krt1 Keratin, type II cytoskeletal 1
☐ Check to include this hit in error tolerant search or archive report

	Query	Observed	Mr(expt)	Mr(calc)	ppm	Miss	Score	Expect	Rank	Peptide
☑	258	633.3261	1264.6376	1264.6299	6.12	0	11	5.7	1	R.TNAENEFVTIK.K
	354	738.3995	1474.7844	1474.7780	4.38	0	(22)	0.4	1	R.FLEQQNQVLQTK.W
	355	738.4022	1474.7898	1474.7780	8.04	0	55	0.0002	1	R.FLEQQNQVLQTK.W

---

7. IPI00222228 **Mass:** 58587 **Score:** 45 **Queries matched:** 3 **emPAI:** 0.06
Tax_Id=10090 Gene_Symbol=4732456N10Rik hypothetical protein LOC239673
☐ Check to include this hit in error tolerant search or archive report

	Query	Observed	Mr(expt)	Mr(calc)	ppm	Miss	Score	Expect	Rank	Peptide
	263	639.3532	1276.6918	1276.7027	-8.47	0	(33)	0.036	1	K.LALDIEIATYR.K
	264	639.3586	1276.7026	1276.7027	-0.01	0	40	0.0068	1	K.LALDIEIATYR.K
☑	577	906.4692	1810.9238	1810.8923	17.4	0	9	7.3	1	R.TNLEPMFEAYITNLR.R

---

8. IPI00138892 **Mass:** 15004 **Score:** 43 **Queries matched:** 3 **emPAI:** 0.23
Tax_Id=10090 Gene_Symbol=Uba52 Ubiquitin-60S ribosomal protein L40
☐ Check to include this hit in error tolerant search or archive report

	Query	Observed	Mr(expt)	Mr(calc)	ppm	Miss	Score	Expect	Rank	Peptide
☑	564	894.4686	1786.9226	1786.9200	1.47	0	(35)	0.019	1	K.TITLEVEPSDTIENVK.A
☑	566	894.4698	1786.9250	1786.9200	2.82	0	37	0.011	1	K.TITLEVEPSDTIENVK.A
☑	567	894.4714	1786.9282	1786.9200	4.61	0	(21)	0.48	1	K.TITLEVEPSDTIENVK.A

Proteins matching the same set of peptides:
IPI00470152 **Mass:** 18282 **Score:** 43 **Queries matched:** 3
Tax_Id=10090 Gene_Symbol=Rps27a Ubiquitin-40S ribosomal protein S27a
IPI00895319 **Mass:** 22277 **Score:** 43 **Queries matched:** 3
Tax_Id=10090 Gene_Symbol=2810422J05Rik 22 kDa protein
IPI00895479 **Mass:** 27181 **Score:** 43 **Queries matched:** 3
Tax_Id=10090 Gene_Symbol=2810422J05Rik 27 kDa protein
IPI00918763 **Mass:** 11021 **Score:** 43 **Queries matched:** 3
Tax_Id=10090 Gene_Symbol=Uba52 Ubiqutin subunit 1
IPI00750889 **Mass:** 22822 **Score:** 43 **Queries matched:** 3
Tax_Id=10090 Gene_Symbol=Ubc Ubc protein
IPI00923013 **Mass:** 26603 **Score:** 43 **Queries matched:** 3
Tax_Id=10090 Gene_Symbol=Ubb Ubiquitin B
IPI00923037 **Mass:** 22578 **Score:** 43 **Queries matched:** 3
Tax_Id=10090 Gene_Symbol=Ubc Putative uncharacterized protein
IPI00139518 **Mass:** 34348 **Score:** 43 **Queries matched:** 3
Tax_Id=10090 Gene_Symbol=Ubb Polyubiquitin-B
IPI00755916 **Mass:** 82614 **Score:** 43 **Queries matched:** 3
Tax_Id=10090 Gene_Symbol=Ubc ubiquitin
IPI00969323 **Mass:** 82603 **Score:** 43 **Queries matched:** 3
Tax_Id=10090 Gene_Symbol=Ubc Polyubiquitin-C

---

9. IPI00874682 **Mass:** 36038 **Score:** 38 **Queries matched:** 3 **emPAI:** 0.09
Tax_Id=10090 Gene_Symbol=- 36 kDa protein
☐ Check to include this hit in error tolerant search or archive report

	Query	Observed	Mr(expt)	Mr(calc)	ppm	Miss	Score	Expect	Rank	Peptide
☑	306	685.3730	1368.7314	1368.7361	-3.40	0	38	0.011	1	R.GAAQNIIPASTGAAK.A
☑	307	685.3763	1368.7380	1368.7361	1.42	0	(6)	15	1	R.GAAQNIIPASTGAAK.A
	648	737.6991	2210.0755	2210.1075	-14.50	0	1	38	2	R.VIISAPSADAPMIVMGVNHEK.Y + 2 Oxidation (M)

---

10. IPI00223714 **Mass:** 21964 **Score:** 38 **Queries matched:** 2 **emPAI:** 0.15
Tax_Id=10090 Gene_Symbol=Hist1h1e Histone H1.4
☐ Check to include this hit in error tolerant search or archive report

Query	Observed	Mr(expt)	Mr(calc)	ppm	Miss	Score	Expect	Rank	Peptide
230	614.8427	1227.6708	1227.6711	-0.18	0	38	0.012	1	K.TSGPPVSELITK.A
231	614.8442	1227.6738	1227.6711	2.27	0	(4)	28	1	K.TSGPPVSELITK.A

Peptide matches not assigned to protein hits: (no details means no match)

Query	Observed	Mr(expt)	Mr(calc)	ppm	Miss	Score	Expect	Rank	Peptide
138	523.2836	1044.5526	1044.5564	-3.55	0	30	0.084	1	INDITLNSR
141	523.2860	1044.5574	1044.5564	1.05	0	30	0.089	1	INDITLNSR
140	523.2858	1044.5570	1044.5564	0.67	0	30	0.09	1	INDITLNSR
550	851.4625	1700.9104	1700.8985	7.02	0	29	0.065	1	AVFVDLEPTVIDEVR
756	1188.2642	3561.7708	3561.7951	-6.83	0	27	0.042	1	TAGAQVVLTDQELQLWPSDLDKPSASEGLPPGSR
82	423.2244	844.4342	844.4515	-20.40	1	26	0.29	1	AIRENSR
234	615.3033	1228.5920	1228.6088	-13.63	0	21	0.5	1	DAVLEPFPADR
93	435.7734	869.5322	869.5123	22.9	0	21	0.32	1	VSLWLPR
25	325.1812	648.3478	648.3595	-17.97	0	21	0.69	1	AFNAVK
137	523.2833	1044.5520	1044.5564	-4.12	0	20	1	1	INDITLNSR
542	851.4489	1700.8832	1700.8985	-8.98	0	19	0.8	1	AVFVDLEPTVIDEVR
78	421.7591	841.5036	841.4658	45.0	0	18	0.78	1	ATHTVSVK
270	651.8633	1301.7120	1301.7078	3.24	0	18	0.83	1	SLDLDSIIAEVK
159	556.3107	1110.6068	1110.5777	26.2	1	17	0.98	1	LMKTVQTMK + 2 Oxidation (M)
420	785.8442	1569.6738	1569.7466	-46.32	1	17	0.51	1	KWTMILMMNNTR + 2 Oxidation (M)
285	657.8660	1313.7174	1313.7092	6.30	0	17	1.3	1	LATVGELQAAWR
251	631.3092	1260.6038	1260.5656	30.3	0	17	1.5	1	SDLEAHVESMK + Oxidation (M)
132	517.2679	1032.5212	1032.4771	42.8	1	16	2.1	1	NAMRDPSSR
91	435.7730	869.5314	869.4971	39.6	0	15	1.4	1	AVVESLPR
235	615.3042	1228.5938	1228.6200	-21.31	0	14	2.3	1	IGGLNWAIDDR
68	412.7538	823.4930	823.5028	-11.85	1	14	1	1	VPSRLPR
145	525.2818	1048.5490	1048.5553	-5.93	0	14	3.3	1	AAAGEIIYNK
288	657.8696	1313.7246	1313.7092	11.8	0	14	2.3	1	LATVGELQAAWR
67	412.7532	823.4918	823.4664	30.9	0	14	1.1	1	ALSIHQR
50	379.7120	757.4094	757.3719	49.6	0	14	4.4	1	SSPAGSPR
552	851.4630	1700.9114	1700.8985	7.60	0	14	2	1	AVFVDLEPTVIDEVR
90	435.7719	869.5292	869.4858	49.9	0	14	1.7	1	VSGVEVPLA
348	738.3803	1474.7460	1474.8065	-40.98	1	14	3.2	1	VESLLENMGIKVK + Oxidation (M)
64	412.7518	823.4890	823.4916	-3.07	0	14	1.2	1	TVAPALPR
269	651.8616	1301.7086	1301.6649	33.6	0	14	2.6	1	ATIATMPVPETR + Oxidation (M)
549	851.4609	1700.9072	1700.8985	5.13	0	13	2.4	1	AVFVDLEPTVIDEVR
142	523.2862	1044.5578	1044.5829	-23.94	1	13	4.3	1	AVGRRPFDK
75	421.7574	841.5002	841.4810	22.9	0	13	2.6	1	AVLTWPR
168	565.3207	1128.6268	1128.6291	-2.01	0	13	3.6	1	IGSGLAHAYIK
86	428.7643	855.5140	855.4814	38.2	0	13	2.5	1	LGAETLPR
638	1073.0280	2144.0414	2144.0055	16.7	1	13	2.6	1	TPKPGNPSSEESSKEVPSCK
681	791.7297	2372.1673	2372.2059	-16.28	1	13	2.2	1	DPLARLSNPLCANASYWAVVR
759	1188.2699	3561.7879	3561.6931	26.6	1	13	1.1	1	TGWAMVSLCIVFAMTSGQMWNHIRGPPYAHK + Oxidation (M)
259	634.3560	1266.6974	1266.6720	20.1	1	13	2.6	1	RNIIGYFEQK
670	1168.5640	2335.1134	2335.0751	16.4	1	13	2.4	1	TPWRMDTSGTPQTTADSATSPK
352	738.3887	1474.7628	1474.7741	-7.66	0	12	4.1	1	LMVEFPLDPALSK + Oxidation (M)
117	496.2275	990.4404	990.4804	-40.33	1	12	2.8	1	AKMDLEER
76	421.7582	841.5018	841.4658	42.9	0	12	3.3	1	ATHTVSVK
74	421.7559	841.4972	841.4810	19.3	0	12	3.6	1	AVLTWPR
130	516.2977	1030.5808	1030.6036	-22.07	1	12	5.2	1	GLVGLNFRR
139	523.2856	1044.5566	1044.5564	0.28	0	12	6.6	1	INDITLNSR
271	651.8643	1301.7140	1301.7078	4.78	0	11	4.1	1	SLDLDSIIAEVK
37	692.3918	691.3845	691.3765	11.5	1	11	5.8	1	KFAGNR
94	435.7735	869.5324	869.5698	-42.96	1	11	3.1	1	VLKILER
553	851.4633	1700.9120	1700.8985	7.96	0	11	4	1	AVFVDLEPTVIDEVR
298	672.8471	1343.6796	1343.7157	-26.84	1	11	5.7	1	GSVGRSSAPAASLGK
331	717.8047	1433.5948	1433.6535	-40.92	1	11	1.7	1	ALQRDDWSGSDGK
51	379.7136	757.4126	757.4334	-27.39	0	11	9.4	1	GLNEVVK
462	801.9191	1601.8236	1601.7443	49.6	0	11	5.7	1	FHTMPACAATNLPR + Oxidation (M)
129	515.7794	1029.5442	1029.5931	-47.43	1	11	8	1	AQVKLSTQR
312	691.3352	1380.6558	1380.6741	-13.21	1	11	5.2	1	KNVIMELSEMR + 2 Oxidation (M)
467	802.8889	1603.7632	1603.7512	7.53	0	10	5.8	1	AEVMNPTRPVSSSET
654	737.7070	2210.0992	2210.0967	1.11	0	10	5.3	1	LGEHNINVLEGNEQFIDAAK
398	785.8391	1569.6636	1569.6960	-20.62	0	10	2.4	1	ESWEAAQEAWAHR
762	1188.2935	3561.8587	3561.9534	-26.60	1	10	2	1	ELLTGTDLWPVAIFTVIFILLVDLMHRHQR + Oxidation (M)
96	435.7746	869.5346	869.5698	-40.43	1	10	4.3	1	VLKEILR
713	958.1292	2871.3658	2871.4477	-28.53	1	10	4	1	WDVCLTLSEKGFQQISFVNSIATTK
350	738.3823	1474.7500	1474.6787	48.4	1	9	8.4	1	VDTEPKAEEASGDK
73	414.2264	826.4382	826.4773	-47.26	1	9	6.3	1	AALPRGSR
340	738.3669	1474.7192	1474.7602	-27.80	1	9	8.6	1	GDGLAFQMRTGLLP
543	851.4530	1700.8914	1700.8985	-4.15	0	9	7	1	AVFVDLEPTVIDEVR
95	435.7746	869.5346	869.4971	43.2	0	9	4.7	1	AVVESLPR
345	738.3784	1474.7422	1474.7932	-34.58	0	9	9.1	1	LHHTSLPDFPAIK
71	414.2235	826.4324	826.4297	3.32	0	9	6.4	1	DVLPNNR
371	760.8850	1519.7554	1519.6833	47.5	1	9	9.1	1	VLEMSMKSTCFR + 2 Oxidation (M)
77	421.7587	841.5028	841.4810	25.9	0	9	7.1	1	AVLTWPR
176	565.3246	1128.6346	1128.5928	37.1	0	9	9.3	1	TDVNPFLAPR
729	772.4192	3085.6477	3085.5989	15.8	0	8	2.7	1	MILGIILSMMVVSIFVFSCLLHHEHK + 2 Oxidation (M)

489	810.8901	1619.7656	1619.7865	-12.89	0	8	9.1	1	GLYDGPVCEVSVTPK
128	514.2868	1026.5590	1026.5458	12.9	0	8	10	1	GPLVQGVDSR
460	801.4333	1600.8520	1600.8429	5.71	0	8	9	1	SMIISATIHMQVVR + Oxidation (M)
614	1057.5195	2113.0244	2112.9687	26.4	1	8	9.3	1	KCFQTAHFYLEDNTSPR
646	737.3643	2209.0711	2209.1491	-35.32	1	8	8.3	1	LGEHNIKVLEGNEQFVNAAK
282	657.8612	1313.7078	1313.7415	-25.63	1	8	12	1	AALEASVLRATGR
314	691.3487	1380.6828	1380.7150	-23.26	0	8	12	1	EHLGLSWAELAR
69	412.7543	823.4940	823.4817	15.0	0	8	4.8	1	RPWLPR
92	435.7733	869.5320	869.5334	-1.61	1	8	7	1	VTPKQGIK
643	732.0196	2193.0370	2193.0452	-3.76	1	7	8.9	1	FGSDPATYNGFLEIMKEFK
422	785.8444	1569.6742	1569.6399	21.9	1	7	5.4	1	MKTECDSEQGETR
279	652.3647	1302.7148	1302.6608	41.5	1	7	13	1	KWQPPVFSSDI
233	615.3006	1228.5866	1228.5989	-9.97	0	7	13	1	GEWPWQASLR
72	414.2238	826.4330	826.4198	16.0	1	7	12	1	HHYSKR
508	816.8943	1631.7740	1631.8460	-44.10	0	7	14	1	LVFAAYGNVFSAFAR
663	763.3481	2287.0225	2287.1154	-40.63	1	7	6.9	1	EMKAEVNAGFSAIYESIGSLR + Oxidation (M)
546	851.4565	1700.8984	1700.8985	-0.04	0	7	12	1	AVFVDLEPTVIDEVR
297	670.8989	1339.7832	1339.7282	41.1	1	6	7.2	1	KMPVGLEPAVGSR
761	1188.2787	3561.8143	3561.7859	7.96	1	6	5.3	1	QQQQTGNHPITVHCSAGAGRTGTFIALSNILER
695	888.7545	2663.2417	2663.3009	-22.24	1	6	11	1	WMGVSDKSVIVCSTGSSLLLFSMK + 2 Oxidation (M)
622	709.6712	2125.9918	2125.9747	8.01	1	5	13	1	ITCAQTFNFTRGLCMYK + Oxidation (M)
113	485.7493	969.4840	969.4563	28.6	1	5	17	1	HCRDQVR
636	1073.0161	2144.0176	2143.9740	20.3	1	5	15	1	KWMEQFEMAMSNIKPDK + 2 Oxidation (M)
605	704.9991	2111.9755	2112.0773	-48.20	1	5	13	1	AFIATSTEGTDKGIMLNTVK + Oxidation (M)
649	737.6996	2210.0770	2210.0967	-8.94	0	5	16	1	LGEHNINVLEGNEQFIDAAK
754	891.4431	3561.7433	3561.6393	29.2	1	5	7	1	MVKDSSLVACAFSPDGGLFVTGSSGGDLTVWDDR + Oxidation (M)
89	435.7656	869.5166	869.5083	9.60	1	5	15	1	VGQPVRSK
640	1073.0392	2144.0638	2144.1399	-35.45	1	5	19	1	IGPISDTSSPELMGVGSLLKK + Oxidation (M)
299	672.8474	1343.6802	1343.6616	13.9	1	5	24	1	CASKQAAVEPGAR
680	1177.0856	2352.1566	2352.1518	2.04	0	5	16	1	ETEAEAQEVSLPALLMEHLDK
589	976.4807	1950.9468	1950.9543	-3.84	0	5	20	1	DEIQCVVATVAFGMGINK
36	692.3890	691.3817	691.3574	35.1	0	5	28	1	TALMEK
570	897.8961	1793.7776	1793.7699	4.32	1	4	12	1	MSYLKTTMEDEESSK + Oxidation (M)
688	819.4637	2455.3693	2455.2642	42.8	1	4	5.7	1	VREVEVVPSNMWGGQGLLGASVR + Oxidation (M)
210	574.3311	1146.6476	1146.6318	13.8	0	4	18	1	TAMIVAEVLGK + Oxidation (M)
571	897.9096	1793.8046	1793.8764	-40.00	1	4	16	1	NTMLSTPCVATKQSTR
709	929.7959	2786.3659	2786.5001	-48.16	1	4	15	1	GLDLLQTCFVVSGGSLNLPRLQSLSL
609	1057.0087	2112.0028	2112.0487	-21.71	1	4	19	1	EKWLLQGVPAGTAEEEEAR
734	801.4522	3201.7797	3201.8601	-25.11	1	4	4.6	1	PPPPGPAAALGTALLLLLASESSHTVLLRAR
255	631.8049	1261.5952	1261.5537	33.0	0	4	26	1	MDFSDYDLLK + Oxidation (M)
396	785.8381	1569.6616	1569.7059	-28.22	0	4	8.8	1	TEGASPEGEGTPWPR
114	485.7534	969.4922	969.4992	-7.16	0	4	23	1	SQQPGGQLR
711	933.4422	2797.3048	2797.2450	21.4	1	4	14	1	GARSVAGAGGGGCPAGGNDFQWCFSQVK
522	816.8959	1631.7772	1631.8195	-25.90	1	4	26	1	TEFTFLDYVKQNK
87	428.7668	855.5190	855.4814	44.0	1	4	20	1	IDRLPDK
573	897.9128	1793.8110	1793.7699	22.9	1	4	20	1	MSYLKTTMEDEESSK + Oxidation (M)
178	565.3273	1128.6400	1128.5961	38.9	0	4	28	1	AVMQSQKPPK + Oxidation (M)
70	414.2226	826.4306	826.4259	5.80	0	4	24	1	YLVMSAK + Oxidation (M)
760	1188.2722	3561.7948	3561.7951	-0.09	0	4	9.6	1	TAGAQVVLTDQELQLWPSDLDKPSASEGLPPGSR
671	1168.5660	2335.1174	2335.2019	-36.17	0	4	20	1	LISATDIQYSASLLNSLNEQR
131	517.2668	1032.5190	1032.5352	-15.68	1	3	39	1	RGLQYPDGK
148	530.7909	1059.5672	1059.6077	-38.14	1	3	48	1	VATHTLKYK
49	379.7111	757.4076	757.4195	-15.60	1	3	55	1	DALRQR
618	707.3326	2118.9760	2118.9826	-3.14	1	3	21	1	LKNTSEQDQPMGGWEMIR
697	903.1155	2706.3247	2706.2570	25.0	1	3	20	1	KDVLFGNMAEIYEFHNNIFMSR + 2 Oxidation (M)
281	657.8579	1313.7012	1313.6438	43.7	0	3	37	1	LGTLGYPSYGMR
490	816.8853	1631.7560	1631.7474	5.28	1	3	28	1	SMVNGGSWSGEPPRR + Oxidation (M)
532	816.8991	1631.7836	1631.8460	-38.21	0	3	33	1	LVFAAYGNVFSAFAR
478	810.4119	1618.8092	1618.8501	-25.23	1	3	37	1	SACREVFTQLPALK
647	737.6964	2210.0674	2210.0929	-11.54	1	3	26	1	ELGEHGLYEYTELKTVAMK
346	738.3788	1474.7430	1474.7528	-6.64	1	3	40	1	ADGLYRGSPLVGDR
126	514.2750	1026.5354	1026.5094	25.4	0	3	35	1	NVEGPEGLGR
110	476.7457	951.4768	951.4331	46.0	1	2	42	1	MTKGEENK + Oxidation (M)
764	905.7182	3618.8437	3618.6866	43.4	1	2	13	1	VAVVSVDGCDTGVALRFGAMLGNYSCAAQGTQGGSK + Oxidation (M)
740	851.2089	3400.8065	3400.7409	19.3	1	2	11	1	QLISVFSSPQELGASLAQLVAQRAASCLEGDR
456	795.8763	1589.7380	1589.7113	16.8	1	2	33	1	TQMQQMHGRMVPV + 3 Oxidation (M)
252	631.3129	1260.6112	1260.6172	-4.76	0	2	47	1	GLLSYVMSGYR + Oxidation (M)
776	1019.7321	4074.8993	4074.8525	11.5	1	2	11	1	LFATEECFAICNQALQMHGGYGYLKDYAVQQYMR
648	737.6991	2210.0755	2210.0500	11.5	0	2	34	1	VMQNFSFQPCQETQIPLK + Oxidation (M)
27	325.1888	648.3630	648.3629	0.28	1	2	39	1	GLGMKK + Oxidation (M)
574	897.9168	1793.8190	1793.9029	-46.73	1	2	35	1	NRNLSGGVLMGFMLNR + Oxidation (M)
501	816.8934	1631.7722	1631.7951	-13.98	1	2	44	1	AAGCPTGPGHKAQQPR
339	736.8575	1471.7004	1471.6990	0.97	0	2	44	1	MNLGNTWIHNTR + Oxidation (M)
590	656.9555	1967.8447	1967.8274	8.76	0	2	17	1	ATQADLMELDMAMEPDR + 2 Oxidation (M)
627	1072.5190	2143.0234	2143.0521	-13.35	1	1	36	1	IMGGVDAEEGKWPWQVSVR
150	533.2669	1064.5192	1064.4743	42.2	0	1	55	1	IQACAMESR
344	738.3780	1474.7414	1474.8065	-44.10	1	1	57	1	VESLLENMGIKVK + Oxidation (M)
746	863.4315	3449.6969	3449.7368	-11.57	0	1	18	1	IGVPQASDLAAEAVVLHYTDWLHPEDPTHLR
26	325.1886	648.3626	648.3595	4.86	0	1	45	1	AFNAVK
741	851.4378	3401.7221	3401.6532	20.3	0	1	19	1	MGIPLTWMLLVMMVTSWFTLAEASNSTEAR + Oxidation (M)
668	779.0337	2334.0793	2334.1196	-17.26	1	1	28	1	AGRVCEGDMVIPTGDELSVTTK

639	1073.0326	2144.0506	2143.9844	30.9	0	1	48	1	EMFAQDHPEVNSLEEVVR + Oxidation (M)
617	707.3309	2118.9709	2119.0599	-42.02	1	0	37	1	FREFVYQEAAGPHQTLAR
488	810.8825	1619.7504	1619.7209	18.2	0	0	52	1	LEASADANIQDNMGR + Oxidation (M)
632	715.6734	2143.9984	2144.0498	-23.98	1	0	41	1	KLQNSIQESTQNFDDHLK
183	574.3185	1146.6224	1146.6145	6.89	1	0	70	1	AGSAWVSKVSR
120	505.7470	1009.4794	1009.5127	-32.96	1	0	59	1	VRQMFASR + Oxidation (M)
470	805.9015	1609.7884	1609.8538	-40.57	1	0	61	1	EISNGKWLLYLMK + Oxidation (M)
1	355.0797	354.0724							
2	361.1398	360.1325							
3	361.1489	360.1416							
4	424.3160	423.3087							
5	429.1035	428.0962							
6	445.1225	444.1152							
7	445.1355	444.1282							
8	453.1935	452.1862							
9	472.2896	471.2823							
10	484.8884	483.8811							
11	503.1200	502.1127							
12	503.3114	502.3041							
13	517.2617	516.2544							
14	517.7728	516.7655							
15	519.1511	518.1438							
16	519.2682	518.2609							
17	582.3313	581.3240							
18	591.3353	590.3280							
19	591.3372	590.3299							
20	591.3375	590.3302							
21	615.7893	614.7820							
22	616.3849	615.3776							
23	618.2722	617.2649							
24	632.3665	631.3592							
28	327.6999	653.3852							
29	327.7017	653.3888							
30	332.5570	663.0994							
31	670.8994	669.8921							
32	688.3806	687.3733							
33	692.3674	691.3601							
34	692.3825	691.3752							
35	692.3873	691.3800							
38	714.7493	713.7420							
39	718.3547	717.3474							
40	722.3738	721.3665							
41	729.8591	728.8518							
42	729.8614	728.8541							
43	736.8525	735.8452							
44	742.7342	741.7269							
45	743.8713	742.8640							
46	744.4328	743.4255							
47	747.8560	746.8487							
48	755.4680	754.4607							
52	761.3905	760.3832							
53	763.4495	762.4422							
54	772.7581	771.7508							
55	793.4175	792.4102							
56	793.4280	792.4207							
57	793.4313	792.4240							
58	793.4393	792.4320							
59	797.3967	796.3894							
60	797.6718	796.6645							
61	802.7986	801.7913							
62	808.3721	807.3648							
63	810.4214	809.4141							
65	412.7524	823.4902							
66	412.7527	823.4908							
79	421.7620	841.5094							
80	842.5316	841.5243							
81	423.2233	844.4320							
83	423.2282	844.4418							
84	846.4227	845.4154							
85	846.4623	845.4550							
88	863.7095	862.7022							
97	872.5276	871.5203							
98	874.5309	873.5236							
99	438.7576	875.5006							
100	894.7733	893.7660							
101	906.5190	905.5117							
102	906.5303	905.5230							
103	906.5461	905.5388							
104	920.4861	919.4788							
106	932.4200	931.4127							
107	470.8339	939.6532							

☑	108	951.4979	950.4906
☑	109	951.8571	950.8498
☑	111	478.1041	954.1936
☑	112	963.6493	962.6420
☑	115	977.7725	976.7652
☑	116	982.6216	981.6143
☑	118	999.6141	998.6068
☑	119	505.2855	1008.5564
☑	121	510.2983	1018.5820
☑	122	510.2990	1018.5834
☑	123	510.3012	1018.5878
☑	124	1021.6116	1020.6043
☑	125	1021.8164	1020.8091
☑	127	514.2797	1026.5448
☑	136	520.2693	1038.5240
☑	143	524.7946	1047.5746
☑	144	525.2810	1048.5474
☑	146	525.2917	1048.5688
☑	147	530.7836	1059.5526
☑	149	533.2592	1064.5038
☑	151	538.1253	1074.2360
☑	152	542.7958	1083.5770
☑	153	545.7363	1089.4580
☑	154	545.7634	1089.5122
☑	155	545.7640	1089.5134
☑	156	545.7656	1089.5166
☑	157	546.7594	1091.5042
☑	158	554.2649	1106.5152
☑	160	556.3140	1110.6134
☑	161	556.3168	1110.6190
☑	162	562.2876	1122.5606
☑	163	563.8179	1125.6212
☑	164	565.3137	1128.6128
☑	165	565.3169	1128.6192
☑	166	565.3170	1128.6194
☑	167	565.3181	1128.6216
☑	169	565.3208	1128.6270
☑	170	565.3210	1128.6274
☑	171	565.3214	1128.6282
☑	172	565.3215	1128.6284
☑	173	565.3229	1128.6312
☑	174	565.3243	1128.6340
☑	175	565.3245	1128.6344
☑	177	565.3268	1128.6390
☑	179	565.3289	1128.6432
☑	180	565.3292	1128.6438
☑	181	572.3431	1142.6716
☑	182	574.3153	1146.6160
☑	185	574.3215	1146.6284
☑	186	574.3222	1146.6298
☑	187	574.3236	1146.6326
☑	188	574.3245	1146.6344
☑	189	574.3263	1146.6380
☑	194	574.3277	1146.6408
☑	196	574.3279	1146.6412
☑	206	574.3306	1146.6466
☑	211	574.3311	1146.6476
☑	215	1148.7328	1147.7255
☑	216	579.2955	1156.5764
☑	218	1162.4799	1161.4726
☑	219	582.3195	1162.6244
☑	220	582.3254	1162.6362
☑	221	582.3277	1162.6408
☑	222	582.3297	1162.6448
☑	223	583.2960	1164.5774
☑	224	584.8265	1167.6384
☑	225	588.3420	1174.6694
☑	226	590.2976	1178.5806
☑	227	590.3051	1178.5956
☑	228	606.8477	1211.6808
☑	229	614.8415	1227.6684
☑	232	615.2973	1228.5800
☑	236	615.3125	1228.6104
☑	237	615.3173	1228.6200
☑	238	616.8066	1231.5986
☑	239	618.2707	1234.5268
☑	240	626.3076	1250.6006
☑	241	629.9429	1257.8712
☑	245	631.3045	1260.5944
☑	253	631.3146	1260.6146
☑	254	631.8029	1261.5912

☑	256	631.8098	1261.6050
☑	257	632.3793	1262.7440
☑	260	1276.0366	1275.0293
☑	261	639.3495	1276.6844
☑	262	639.3512	1276.6878
☑	265	639.3650	1276.7154
☑	266	641.3467	1280.6788
☑	267	428.7611	1283.2615
☑	268	648.3688	1294.7230
☑	272	651.8669	1301.7192
☑	278	652.3638	1302.7130
☑	280	657.8510	1313.6874
☑	283	657.8622	1313.7098
☑	284	657.8630	1313.7114
☑	286	657.8670	1313.7194
☑	287	657.8687	1313.7228
☑	289	664.8458	1327.6770
☑	290	665.3529	1328.6912
☑	291	665.3602	1328.7058
☑	292	667.9150	1333.8154
☑	293	670.8458	1339.6770
☑	294	670.8692	1339.7238
☑	295	670.8762	1339.7378
☑	296	670.8839	1339.7532
☑	300	675.8537	1349.6928
☑	301	676.9324	1351.8502
☑	302	677.7803	1353.5460
☑	303	678.8408	1355.6670
☑	304	679.3489	1356.6832
☑	305	341.0215	1360.0569
☑	308	685.3792	1368.7438
☑	311	688.3701	1374.7256
☑	313	691.3355	1380.6564
☑	315	692.3530	1382.6914
☑	316	692.3760	1382.7374
☑	317	692.3795	1382.7444
☑	318	693.8282	1385.6418
☑	319	693.8896	1385.7646
☑	320	694.3635	1386.7124
☑	321	696.3606	1390.7066
☑	322	705.9033	1409.7920
☑	323	355.0473	1416.1601
☑	324	711.3118	1420.6090
☑	325	711.3287	1420.6428
☑	326	711.3327	1420.6508
☑	327	711.8225	1421.6304
☑	328	711.8459	1421.6772
☑	329	716.3568	1430.6990
☑	330	717.3319	1432.6492
☑	332	720.8417	1439.6688
☑	333	720.8440	1439.6734
☑	334	728.3522	1454.6898
☑	335	730.9031	1459.7916
☑	336	731.3462	1460.6778
☑	337	732.3721	1462.7296
☑	338	736.8455	1471.6764
☑	341	738.3699	1474.7252
☑	342	738.3713	1474.7280
☑	343	738.3729	1474.7312
☑	347	738.3795	1474.7444
☑	349	738.3814	1474.7482
☑	351	738.3840	1474.7534
☑	353	738.3912	1474.7678
☑	356	740.3666	1478.7186
☑	357	740.3692	1478.7238
☑	358	742.4603	1482.9060
☑	359	742.8790	1483.7434
☑	360	745.8330	1489.6514
☑	361	748.8511	1495.6876
☑	362	748.8543	1495.6940
☑	363	753.3779	1504.7412
☑	364	756.8419	1511.6692
☑	365	757.3382	1512.6618
☑	366	757.8568	1513.6990
☑	367	757.8580	1513.7014
☑	368	757.8599	1513.7052
☑	369	757.8612	1513.7078
☑	370	758.4351	1514.8556
☑	372	762.8272	1523.6398
☑	373	762.9028	1523.7910
☑	374	766.8405	1531.6664

375	766.8597	1531.7048
376	766.8657	1531.7168
377	766.8687	1531.7228
378	774.8998	1547.7850
379	775.8703	1549.7260
380	775.8704	1549.7262
381	775.8705	1549.7264
382	1550.7377	1549.7304
383	775.8734	1549.7322
384	775.8805	1549.7464
385	785.3551	1568.6956
386	785.8297	1569.6448
387	785.8307	1569.6468
388	785.8309	1569.6472
389	785.8321	1569.6496
390	785.8323	1569.6500
391	785.8363	1569.6580
392	785.8364	1569.6582
393	785.8367	1569.6588
394	785.8369	1569.6592
395	785.8376	1569.6606
397	785.8386	1569.6626
399	785.8392	1569.6638
400	785.8399	1569.6652
401	785.8403	1569.6660
402	785.8403	1569.6660
403	785.8405	1569.6664
404	785.8408	1569.6670
405	785.8409	1569.6672
406	785.8413	1569.6680
407	785.8414	1569.6682
408	785.8416	1569.6686
409	785.8417	1569.6688
410	785.8417	1569.6688
411	785.8420	1569.6694
412	785.8422	1569.6698
413	785.8422	1569.6698
414	785.8422	1569.6698
415	785.8427	1569.6708
416	785.8431	1569.6716
417	785.8435	1569.6724
418	785.8436	1569.6726
419	785.8437	1569.6728
421	785.8442	1569.6738
423	785.8444	1569.6742
424	785.8445	1569.6744
425	785.8447	1569.6748
426	785.8448	1569.6750
427	785.8455	1569.6764
428	785.8459	1569.6772
429	785.8461	1569.6776
430	785.8463	1569.6780
431	785.8463	1569.6780
432	785.8471	1569.6796
433	785.8471	1569.6796
434	785.8474	1569.6802
435	785.8475	1569.6804
436	785.8475	1569.6804
437	785.8478	1569.6810
438	785.8478	1569.6810
439	785.8479	1569.6812
440	785.8482	1569.6818
441	785.8482	1569.6818
442	785.8493	1569.6840
443	785.8496	1569.6846
444	785.8521	1569.6896
445	785.8522	1569.6898
446	785.8557	1569.6968
447	786.8522	1571.6898
448	786.8541	1571.6936
449	786.8574	1571.7002
450	788.7875	1575.5604
451	794.8399	1587.6652
452	794.8420	1587.6694
453	794.8519	1587.6892
454	794.8831	1587.7516
455	795.8742	1589.7338
457	795.8964	1589.7782
458	796.3791	1590.7436
459	801.4287	1600.8428
461	801.4580	1600.9014

☑	463	801.9247	1601.8348
☑	464	801.9269	1601.8392
☑	465	801.9270	1601.8394
☑	466	802.8834	1603.7522
☑	468	802.8927	1603.7708
☑	469	805.8164	1609.6182
☑	471	808.3991	1614.7836
☑	474	809.8861	1617.7576
☑	476	809.8953	1617.7760
☑	477	809.8990	1617.7834
☑	479	810.8393	1619.6640
☑	480	810.8716	1619.7286
☑	484	810.8779	1619.7412
☑	486	810.8810	1619.7474
☑	493	816.8915	1631.7684
☑	514	816.8947	1631.7748
☑	521	816.8958	1631.7770
☑	536	832.6773	1663.3400
☑	537	834.4014	1666.7882
☑	538	844.8012	1687.5878
☑	539	850.9382	1699.8618
☑	540	851.4467	1700.8788
☑	541	851.4474	1700.8802
☑	544	851.4534	1700.8922
☑	545	851.4555	1700.8964
☑	547	851.4571	1700.8996
☑	548	851.4595	1700.9044
☑	551	851.4625	1700.9104
☑	554	851.4734	1700.9322
☑	555	852.4055	1702.7964
☑	556	854.3867	1706.7588
☑	557	854.4058	1706.7970
☑	558	428.8021	1711.1793
☑	559	858.4742	1714.9338
☑	560	890.4050	1778.7954
☑	561	890.4119	1778.8092
☑	562	890.4155	1778.8164
☑	563	892.7701	1783.5256
☑	565	894.4686	1786.9226
☑	568	896.3610	1790.7074
☑	569	896.3789	1790.7432
☑	572	897.9101	1793.8056
☑	575	905.9919	1809.9692
☑	576	906.0045	1809.9944
☑	578	906.5100	1811.0054
☑	579	908.4478	1814.8810
☑	580	914.9546	1827.8946
☑	581	915.4625	1828.9104
☑	582	915.4660	1828.9174
☑	583	915.4718	1828.9290
☑	584	616.3306	1845.9700
☑	585	924.9957	1847.9768
☑	586	954.7288	1907.4430
☑	587	956.0098	1910.0050
☑	588	973.5104	1945.0062
☑	591	671.3910	2011.1512
☑	592	1011.4334	2020.8522
☑	593	1024.5026	2046.9906
☑	594	1030.4009	2058.7872
☑	595	1040.9728	2079.9310
☑	596	694.3204	2079.9394
☑	597	1040.9795	2079.9444
☑	600	1040.9845	2079.9544
☑	601	1040.9870	2079.9594
☑	603	699.3636	2095.0690
☑	606	1056.9989	2111.9832
☑	608	1057.0073	2112.0000
☑	610	705.0086	2112.0040
☑	611	705.3356	2112.9850
☑	612	705.3407	2113.0003
☑	613	1057.5079	2113.0012
☑	615	706.3426	2116.0060
☑	616	707.0169	2118.0289
☑	619	707.3403	2118.9991
☑	620	709.3424	2125.0054
☑	621	709.3670	2125.0792
☑	624	715.3429	2143.0069
☑	628	1072.5261	2143.0376
☑	630	1072.5365	2143.0584
☑	631	1072.5383	2143.0620
☑	633	1073.0101	2144.0056

☑	634	715.6771	2144.0095
☑	635	715.6795	2144.0167
☑	637	1073.0237	2144.0328
☑	641	720.3670	2158.0792
☑	642	722.6668	2164.9786
☑	644	732.0351	2193.0835
☑	645	735.3560	2203.0462
☑	650	737.6997	2210.0773
☑	651	737.7006	2210.0800
☑	652	737.7010	2210.0812
☑	653	737.7034	2210.0884
☑	655	737.7103	2210.1091
☑	656	738.0344	2211.0814
☑	657	1108.0321	2214.0496
☑	658	744.0340	2229.0802
☑	659	761.3887	2281.1443
☑	660	761.7104	2282.1094
☑	661	762.0528	2283.1366
☑	662	762.0620	2283.1642
☑	664	767.3689	2299.0849
☑	665	767.3712	2299.0918
☑	666	767.3818	2299.1236
☑	667	768.0377	2301.0913
☑	669	779.0370	2334.0892
☑	672	779.7130	2336.1172
☑	673	780.0542	2337.1408
☑	676	1177.0771	2352.1396
☑	679	1177.0840	2352.1534
☑	684	792.3639	2374.0699
☑	685	792.7459	2375.2159
☑	686	800.3902	2398.1488
☑	687	803.7632	2408.2678
☑	689	821.3797	2461.1173
☑	690	834.4104	2500.2094
☑	691	834.4430	2500.3072
☑	692	838.4274	2512.2604
☑	693	858.4812	2572.4218
☑	694	888.7502	2663.2288
☑	696	888.7626	2663.2660
☑	698	1372.7352	2743.4558
☑	699	916.4213	2746.2421
☑	700	917.0810	2748.2212
☑	701	917.0811	2748.2215
☑	702	919.0511	2754.1315
☑	703	919.7774	2756.3104
☑	704	929.1258	2784.3556
☑	705	929.4644	2785.3714
☑	706	929.7783	2786.3131
☑	707	929.7839	2786.3299
☑	708	929.7928	2786.3566
☑	710	933.4380	2797.2922
☑	712	958.1219	2871.3439
☑	714	958.1293	2871.3661
☑	715	958.1404	2871.3994
☑	716	958.1454	2871.4144
☑	717	958.1537	2871.4393
☑	718	964.1164	2889.3274
☑	719	972.9012	2915.6818
☑	720	973.4361	2917.2865
☑	721	731.1655	2920.6329
☑	722	979.8508	2936.5306
☑	723	738.4187	2949.6457
☑	724	738.4551	2949.7913
☑	725	1005.4928	3013.4566
☑	726	1015.8009	3044.3809
☑	727	766.3745	3061.4689
☑	728	1026.1361	3075.3865
☑	730	793.2418	3168.9381
☑	731	1065.4698	3193.3876
☑	732	801.2135	3200.8249
☑	733	801.4345	3201.7089
☑	735	802.1821	3204.6993
☑	736	802.4299	3205.6905
☑	737	812.4256	3245.6733
☑	738	1087.5117	3259.5133
☑	739	838.3743	3349.4681
☑	742	851.4606	3401.8133
☑	743	854.1410	3412.5349
☑	744	1139.8470	3416.5192
☑	745	859.4880	3433.9229
☑	747	872.0906	3484.3333

☑	748	872.4459	3485.7545
☑	749	877.3615	3505.4169
☑	750	1172.2100	3513.6082
☑	751	882.1300	3524.4909
☑	752	888.2696	3549.0493
☑	753	891.4270	3561.6789
☑	755	1188.2625	3561.7657
☑	757	891.4513	3561.7761
☑	758	1188.2662	3561.7768
☑	763	896.4558	3581.7941
☑	765	906.2194	3620.8485
☑	766	911.6751	3642.6713
☑	767	915.6857	3658.7137
☑	768	929.2371	3712.9193
☑	769	933.8598	3731.4101
☑	770	939.4158	3753.6341
☑	771	943.0184	3768.0445
☑	772	958.7573	3831.0001
☑	773	967.6097	3866.4097
☑	774	1004.0294	4012.0885
☑	775	1011.5858	4042.3141
☑	777	1081.1510	4320.5749
☑	778	1085.1560	4336.5949
☑	779	1140.0453	4556.1521
☑	780	1143.4764	4569.8765
☑	781	1151.2518	4600.9781
☑	782	1192.8083	4767.2041
☑	783	1223.8950	4891.5509
☑	784	1262.2261	5044.8753
☑	785	1280.1780	5116.6829
☑	786	1385.7402	5538.9317

Search Parameters

```
Type of search         : MS/MS Ion Search
Enzyme                 : Trypsin
Fixed modifications    : Carbamidomethyl (C)
Variable modifications : Oxidation (M)
Mass values            : Monoisotopic
Protein Mass           : Unrestricted
Peptide Mass Tolerance : ± 50 ppm
Fragment Mass Tolerance: ± 0.2 Da
Max Missed Cleavages   : 1
Instrument type        : ESI-QUAD-TOF
Number of queries      : 786
```

Mascot: http://www.matrixscience.com/

Mascot Search Results

```
User           : Dominic
Email          : dominic.kurian@bbsrc.ac.uk
Search title   : IPI Mouse_15-02-2010
MS data file   : D:\Peaklist\Dominic Kurian\QTofDK20010807 GATC P 0010885.pkl
Database       : IPI_mouse MOUSE_v3_79 (54943 sequences; 24917557 residues)
Timestamp      : 15 Feb 2011 at 16:07:35 GMT
Protein hits   : IPI00420725 Tax_Id=10090 Gene_Symbol=Camk2a Uncharacterized protein
                 IPI00755181 Tax_Id=10090 Gene_Symbol=Krt10 keratin, type I cytoskeletal 10
                 IPI00131366 Tax_Id=10090 Gene_Symbol=Krt6b Keratin, type II cytoskeletal 6B
                 IPI00348328 Tax_Id=10090 Gene_Symbol=Krt78 keratin Kb40
                 IPI00322209 Tax_Id=10090 Gene_Symbol=Krt8 Keratin, type II cytoskeletal 8
                 IPI00347110 Tax_Id=10090 Gene_Symbol=Krt73 Keratin, type II cytoskeletal 73
```

	IPI_mouse	Decoy	False discovery rate
Peptide matches above identity threshold	18	0	0.00 %
Peptide matches above homology or identity threshold	22	0	0.00 %

Probability Based Mowse Score

Ions score is -10*Log(P), where P is the probability that the observed match is a random event.
Individual ions scores > 37 indicate identity or extensive homology (p<0.01).
Protein scores are derived from ions scores as a non-probabilistic basis for ranking protein hits.

Score Distribution

Peptide Summary Report

Format As: Peptide Summary   Help

Significance threshold p< 0.01   Max. number of hits AUTO

Standard scoring ○ MudPIT scoring ● Ions score or expect cut-off 0   Show sub-sets 0

Show pop-ups ● Suppress pop-ups ○ Sort unassigned Decreasing Score   Require bold red ☐

Select All | Select None | Search Selected | ☐ **Error tolerant** | Archive Report

1. IPI00420725 **Mass:** 55882 **Score:** 276 **Queries matched:** 24 **emPAI:** 0.12
Tax_Id=10090 Gene_Symbol=Camk2a Uncharacterized protein
☐ Check to include this hit in error tolerant search or archive report

	Query	Observed	Mr(expt)	Mr(calc)	ppm	Miss	Score	Expect	Rank	Peptide
☑	196	574.3283	1146.6420	1146.6431	-0.89	0	(1)	44	1	K.GAILTTMLATR.N
☑	197	574.3286	1146.6426	1146.6431	-0.36	0	23	0.26	1	K.GAILTTMLATR.N
	198	574.3295	1146.6444	1146.6431	1.21	0	(2)	35	3	K.GAILTTMLATR.N
☑	268	631.3032	1260.5918	1260.5927	-0.71	0	(8)	10	1	R.FYFENLWSR.N
☑	270	631.3068	1260.5990	1260.5927	5.00	0	38	0.01	1	R.FYFENLWSR.N
☑	301	652.3496	1302.6846	1302.6932	-6.56	0	(12)	4.6	1	R.ITQYLDAGGIPR.T
☑	302	652.3525	1302.6904	1302.6932	-2.11	0	18	1.3	1	R.ITQYLDAGGIPR.T
☑	493	816.8842	1631.7538	1631.7719	-11.05	0	(55)	0.00019	1	R.FTEEYQLFEELGK.G
☑	496	816.8906	1631.7666	1631.7719	-3.21	0	(16)	1.5	1	R.FTEEYQLFEELGK.G
☑	497	816.8911	1631.7676	1631.7719	-2.59	0	(56)	0.00014	1	R.FTEEYQLFEELGK.G
☑	498	816.8913	1631.7680	1631.7719	-2.35	0	(51)	0.00053	1	R.FTEEYQLFEELGK.G
☑	500	816.8914	1631.7682	1631.7719	-2.23	0	(55)	0.00019	1	R.FTEEYQLFEELGK.G
☑	501	816.8923	1631.7700	1631.7719	-1.12	0	72	4e-006	1	R.FTEEYQLFEELGK.G
☑	502	816.8925	1631.7704	1631.7719	-0.88	0	(39)	0.0085	1	R.FTEEYQLFEELGK.G
☑	503	816.8937	1631.7728	1631.7719	0.59	0	(30)	0.069	1	R.FTEEYQLFEELGK.G
☑	505	816.8957	1631.7768	1631.7719	3.04	0	(57)	0.00013	1	R.FTEEYQLFEELGK.G
☑	507	816.8961	1631.7776	1631.7719	3.53	0	(26)	0.16	1	R.FTEEYQLFEELGK.G
☑	508	816.8961	1631.7776	1631.7719	3.53	0	(11)	5	1	R.FTEEYQLFEELGK.G
☑	511	816.8971	1631.7796	1631.7719	4.76	0	(57)	0.00013	1	R.FTEEYQLFEELGK.G
☑	514	816.8975	1631.7804	1631.7719	5.25	0	(26)	0.15	1	R.FTEEYQLFEELGK.G
☑	515	816.8981	1631.7816	1631.7719	5.99	0	(48)	0.0011	1	R.FTEEYQLFEELGK.G
☑	518	816.8998	1631.7850	1631.7719	8.07	0	(14)	2.9	1	R.FTEEYQLFEELGK.G
☑	519	816.9008	1631.7870	1631.7719	9.29	0	(65)	1.9e-005	1	R.FTEEYQLFEELGK.G
☑	521	816.9054	1631.7962	1631.7719	14.9	0	(10)	6.2	1	R.FTEEYQLFEELGK.G

Proteins matching the same set of peptides:

IPI00621806 **Mass:** 54651 **Score:** 276 **Queries matched:** 24
Tax_Id=10090 Gene_Symbol=Camk2a Isoform Alpha CaMKII of Calcium/calmodulin-dependent protein kinase type II subunit alpha

---

2. IPI00755181 **Mass:** 57178 **Score:** 79 **Queries matched:** 3 **emPAI:** 0.12
Tax_Id=10090 Gene_Symbol=Krt10 keratin, type I cytoskeletal 10
☐ Check to include this hit in error tolerant search or archive report

	Query	Observed	Mr(expt)	Mr(calc)	ppm	Miss	Score	Expect	Rank	Peptide
☑	173	545.7680	1089.5214	1089.5237	-2.05	0	68	1.2e-005	1	K.VTMQNLNDR.L
☑	344	691.3235	1380.6324	1380.6408	-6.08	0	(28)	0.083	1	R.ALEESNYELEGK.I
☑	345	691.3331	1380.6516	1380.6408	7.83	0	49	0.00069	1	R.ALEESNYELEGK.I

Proteins matching the same set of peptides:
IPI00798492 **Mass:** 57906 **Score:** 79 **Queries matched:** 3
Tax_Id=10090 Gene_Symbol=Krt10 Isoform 1 of Keratin, type I cytoskeletal 10
IPI00828744 **Mass:** 57197 **Score:** 79 **Queries matched:** 3
Tax_Id=10090 Gene_Symbol=Krt10 Isoform 2 of Keratin, type I cytoskeletal 10

---

3. IPI00131366 **Mass:** 60627 **Score:** 70 **Queries matched:** 3 **emPAI:** 0.05
Tax_Id=10090 Gene_Symbol=Krt6b Keratin, type II cytoskeletal 6B
☐ Check to include this hit in error tolerant search or archive report

	Query	Observed	Mr(expt)	Mr(calc)	ppm	Miss	Score	Expect	Rank	Peptide
☑	298	651.8590	1301.7034	1301.7078	-3.36	0	54	0.00027	1	R.SLDLDSIIAEVK.A
☑	299	651.8593	1301.7040	1301.7078	-2.90	0	(38)	0.0097	1	R.SLDLDSIIAEVK.A
☑	300	651.8599	1301.7052	1301.7078	-1.98	0	(39)	0.0084	1	R.SLDLDSIIAEVK.A

Proteins matching the same set of peptides:
IPI00131368 **Mass:** 59641 **Score:** 70 **Queries matched:** 3
Tax_Id=10090 Gene_Symbol=Krt6a Keratin, type II cytoskeletal 6A
IPI00139301 **Mass:** 61957 **Score:** 70 **Queries matched:** 3
Tax_Id=10090 Gene_Symbol=Krt5 Keratin, type II cytoskeletal 5
IPI00221797 **Mass:** 59932 **Score:** 70 **Queries matched:** 3
Tax_Id=10090 Gene_Symbol=Krt75 Keratin, type II cytoskeletal 75
IPI00330480 **Mass:** 35315 **Score:** 70 **Queries matched:** 3
Tax_Id=10090 Gene_Symbol=- 35 kDa protein
IPI00470126 **Mass:** 60553 **Score:** 70 **Queries matched:** 3
Tax_Id=10090 Gene_Symbol=Krt5 Uncharacterized protein
IPI00762364 **Mass:** 50641 **Score:** 70 **Queries matched:** 3
Tax_Id=10090 Gene_Symbol=Krt6a Uncharacterized protein
IPI00785403 **Mass:** 59832 **Score:** 70 **Queries matched:** 3
Tax_Id=10090 Gene_Symbol=Krt6b keratin, type II cytoskeletal 6B

---

4. IPI00348328 **Mass:** 114018 **Score:** 52 **Queries matched:** 2 **emPAI:** 0.03
Tax_Id=10090 Gene_Symbol=Krt78 keratin Kb40
☐ Check to include this hit in error tolerant search or archive report

	Query	Observed	Mr(expt)	Mr(calc)	ppm	Miss	Score	Expect	Rank	Peptide
	160	523.7686	1045.5226	1045.4975	24.1	0	14	4.1	2	R.GSGGQVTMPGR.G
☑	348	692.3519	1382.6892	1382.6830	4.51	0	52	0.00046	1	R.SLNNQFASFIDK.V

---

5. IPI00322209 **Mass:** 54531 **Score:** 52 **Queries matched:** 1 **emPAI:** 0.06
Tax_Id=10090 Gene_Symbol=Krt8 Keratin, type II cytoskeletal 8
☐ Check to include this hit in error tolerant search or archive report

Query	Observed	Mr(expt)	Mr(calc)	ppm	Miss	Score	Expect	Rank	Peptide
348	692.3519	1382.6892	1382.7194	-21.80	1	52	0.00046	1	K.SLNNKFASFIDK.V

---

6. IPI00347110 **Mass:** 59502 **Score:** 46 **Queries matched:** 4 **emPAI:** 0.06
Tax_Id=10090 Gene_Symbol=Krt73 Keratin, type II cytoskeletal 73
☐ Check to include this hit in error tolerant search or archive report

	Query	Observed	Mr(expt)	Mr(calc)	ppm	Miss	Score	Expect	Rank	Peptide
☑	281	639.3522	1276.6898	1276.7027	-10.04	0	(18)	1.1	1	K.LALDIEIATYR.K
☑	282	639.3556	1276.6966	1276.7027	-4.71	0	20	0.72	1	K.LALDIEIATYR.K
☑	284	639.3583	1276.7020	1276.7027	-0.48	0	(19)	0.73	1	K.LALDIEIATYR.K
☑	401	738.3970	1474.7794	1474.7780	0.99	0	46	0.0016	1	R.FLEQQNQVLQTK.W

Peptide matches not assigned to protein hits: (no details means no match)

	Query	Observed	Mr(expt)	Mr(calc)	ppm	Miss	Score	Expect	Rank	Peptide
☑	240	614.8435	1227.6724	1227.6711	1.13	0	36	0.019	1	TSGPPVSELITK
☑	157	523.2830	1044.5514	1044.5564	-4.69	0	32	0.058	1	INDITLNSR
☑	156	523.2830	1044.5514	1044.5564	-4.69	0	30	0.098	1	INDITLNSR
☑	155	523.2829	1044.5512	1044.5564	-4.89	0	30	0.1	1	INDITLNSR
☑	103	421.7574	841.5002	841.4810	22.9	0	27	0.11	1	LGTWLPR
☑	109	428.7642	855.5138	855.4814	37.9	0	24	0.22	1	LGAETLPR
☑	102	421.7571	841.4996	841.4810	22.1	0	20	0.5	1	LGTWLPR

170	533.2635	1064.5124	1064.5138	-1.28	0	20	0.68	1	AQYEDIAQK
534	851.4528	1700.8910	1700.8985	-4.39	0	20	0.64	1	AVFVDLEPTVIDEVR
104	423.2249	844.4352	844.4515	-19.22	1	19	1.7	1	AIRENSR
67	741.4396	740.4323	740.4657	-45.07	1	19	0.54	1	RLVTPR
245	616.3294	1230.6442	1230.6456	-1.06	0	18	1.4	1	ASQEVQEILSK
248	616.7965	1231.5784	1231.5621	13.2	0	18	0.95	1	IANEWEEWR
100	421.7558	841.4970	841.4810	19.0	0	18	0.9	1	LGTWLPR
391	738.3775	1474.7404	1474.6731	45.7	0	18	1.3	1	MIHTGEKPCVCK + Oxidation (M)
229	601.8072	1201.5998	1201.6302	-25.30	1	17	1.6	1	LGSEGVEEVKR
91	412.7550	823.4954	823.5028	-8.94	1	17	0.55	1	VPSRLPR
378	730.8994	1459.7842	1459.7592	17.2	1	17	1.3	1	DVLEKMELLDQK
86	407.2395	812.4644	812.4756	-13.69	1	17	1.1	1	AGPKALEK
243	615.3023	1228.5900	1228.6241	-27.69	0	16	1.4	1	AVPPHETFGFK
160	523.7686	1045.5226	1045.5597	-35.42	1	16	2.4	1	SGKFVFSFK
436	785.8358	1569.6570	1569.7245	-43.00	0	16	0.52	1	MSLAESYAQYVHR + Oxidation (M)
130	485.7459	969.4772	969.4563	21.6	1	16	1.5	1	HCRDQVR
376	730.8926	1459.7706	1459.7453	17.4	1	16	1.9	1	VDILENRVMDTR
566	894.4688	1786.9230	1786.9070	9.00	0	15	1.9	1	LVCSSEPITCKPQLR
373	728.8590	1455.7034	1455.7569	-36.71	0	15	2.1	1	ALALLEDEEQAVR
99	421.7558	841.4970	841.4810	19.0	0	14	2.1	1	AVLTWPR
110	428.7671	855.5196	855.5112	9.82	1	14	1.8	1	ALRMLPR
158	523.2841	1044.5536	1044.5564	-2.59	0	14	3.7	1	INDITLNSR
79	785.3552	784.3479	784.3868	-49.54	1	14	1.1	1	KDYGFR
149	517.7773	1033.5400	1033.5192	20.1	0	14	3.9	1	LLEFQNDR
175	546.7536	1091.4926	1091.5393	-42.77	1	14	2.2	1	RDMQVNTTK
601	705.0020	2111.9842	2112.0011	-8.01	0	14	1.9	1	TTEQLIEAVNNGDFEAYAK
223	594.3091	1186.6036	1186.6492	-38.39	1	14	3.7	1	MSRAPEILVR + Oxidation (M)
241	614.8456	1227.6766	1227.6711	4.55	0	14	3	1	TSGPPVSELITK
63	363.2190	724.4234	724.4344	-15.10	0	13	1.5	1	RPLSPR
88	412.7511	823.4876	823.4817	7.25	0	13	1.3	1	RPWLPR
254	618.2742	1234.5338	1234.5401	-5.04	0	13	1.8	1	NMSGNVHFEGK + Oxidation (M)
286	639.8024	1277.5902	1277.6265	-28.39	0	13	2.9	1	GTSDHWIGLHR
313	665.3237	1328.6328	1328.5992	25.3	0	13	3.1	1	GLMTIYEDEMK
316	665.3463	1328.6780	1328.6547	17.6	0	13	3.7	1	CLPAGPSPEVFR
327	670.8930	1339.7714	1339.7096	46.2	1	13	1.8	1	LSVGGDSDPPLKR
258	624.8118	1247.6090	1247.6180	-7.15	1	13	4	1	KLDSTAGMPNSK
154	523.2826	1044.5506	1044.5564	-5.46	0	13	5	1	INDITLNSR
105	423.2271	844.4396	844.4654	-30.50	1	13	6.6	1	SPASKLDK
115	435.7732	869.5318	869.5123	22.5	0	12	2.3	1	VSLWLPR
90	412.7530	823.4914	823.4664	30.4	1	12	1.6	1	RDIPPAR
148	517.7643	1033.5140	1033.5040	9.72	0	12	5.8	1	AVDLSSGETR
224	595.3015	1188.5884	1188.6251	-30.83	1	12	5.2	1	EVPKQQAAYR
320	665.3650	1328.7154	1328.6684	35.4	1	12	4.3	1	IAVDQEGKDQAR
535	851.4545	1700.8944	1700.8985	-2.39	0	12	3.7	1	AVFVDLEPTVIDEVR
352	696.3466	1390.6786	1390.7279	-35.39	0	12	4.9	1	QFCLELNGLAVK
47	632.3531	631.3458	631.3289	26.8	0	12	14	1	ETNLR
393	738.3797	1474.7448	1474.7528	-5.41	0	12	5.2	1	SGAGSHATHELPAIK
128	476.7454	951.4762	951.4960	-20.78	1	11	5.5	1	MVGQAFRK + Oxidation (M)
392	738.3784	1474.7422	1474.8065	-43.56	1	11	5.6	1	VESLLENMGIKVK + Oxidation (M)
72	379.7110	757.4074	757.3719	47.0	0	11	8.4	1	SSPAGSPR
159	523.3225	1044.6304	1044.5828	45.6	1	11	2.4	1	AARLGSPFAR
321	665.3677	1328.7208	1328.7187	1.60	0	11	4.9	1	NLDLDSIIAEVK
417	768.4241	1534.8336	1534.9096	-49.51	1	11	3.9	1	HFLGGLVPVRVSVR
267	630.8173	1259.6200	1259.6357	-12.44	0	11	6.6	1	AVEESITNLER
73	763.4373	762.4300	762.4276	3.20	0	11	6.8	1	LGAFDLK
120	437.7486	873.4826	873.4807	2.18	0	11	8.5	1	ATEDVLVK
165	530.7841	1059.5536	1059.5383	14.5	1	11	8.9	1	AQKMDPSGVK
491	815.3732	1628.7318	1628.7214	6.39	0	11	3.3	1	MGAPEAGMAEYLFDK
172	545.2977	1088.5808	1088.5714	8.71	0	10	8.6	1	VTDLQGDLTK
118	435.7745	869.5344	869.5123	25.5	0	10	3.7	1	VSLWLPR
234	605.8242	1209.6338	1209.6466	-10.52	1	10	7.1	1	EPVEQPRVTR
504	816.8955	1631.7764	1631.8242	-29.27	0	10	6.2	1	MHAINGFLFSNLPR + Oxidation (M)
309	659.3605	1316.7064	1316.7010	4.17	0	10	7.1	1	IEMEVEVALLR + Oxidation (M)
198	574.3295	1146.6444	1146.6649	-17.80	0	10	5.7	1	AVALDGTLFLK
145	517.2630	1032.5114	1032.5273	-15.40	1	10	9	1	KEQEQMLK
127	475.2524	948.4902	948.5280	-39.80	0	10	9	1	NYLELVAK
98	421.7497	841.4848	841.4844	0.58	0	9	6.4	1	AAPMLIAR
388	738.3769	1474.7392	1474.6949	30.1	1	9	9.3	1	QMNSFQVAFLKM + 2 Oxidation (M)
418	772.8556	1543.6966	1543.6395	37.0	0	9	5.1	1	CSNSADMTGPSFVR + Oxidation (M)
577	901.4461	1800.8776	1800.8328	24.9	0	9	8.5	1	LYGWTMEPVFVCQR + Oxidation (M)
150	519.2672	1036.5198	1036.5077	11.7	0	9	10	1	EFDGTEVLK
565	894.4647	1786.9148	1786.9764	-34.43	1	9	9	1	IAFPNLKVVMVVDGNR + Oxidation (M)
274	632.3272	1262.6398	1262.6441	-3.40	1	9	11	1	NVTIECPFKR
57	692.3731	691.3658	691.3905	-35.63	0	8	13	1	ATYLPK
89	412.7522	823.4898	823.5280	-46.27	1	8	4	1	TKILPPR
557	883.9029	1765.7912	1765.7789	7.02	1	8	5.5	1	KADSGEDSLGCLNGDTK
554	879.9472	1757.8798	1757.9060	-14.89	1	8	9.3	1	TEHAPSGKLSEIQHPK

☑	528	832.3854	1662.7562	1662.8366	-48.31	0	8	7.8	1	FVTLQIWDTAGQER
☑	179	554.2792	1106.5438	1106.5940	-45.33	1	8	13	1	ALCMTLVKR + Oxidation (M)
☑	580	913.9105	1825.8064	1825.8298	-12.82	0	8	5.5	1	EGSVGGSQVAALMMSSQR + 2 Oxidation (M)
☑	375	730.8780	1459.7414	1459.7042	25.5	0	8	12	1	GVSEPLLETDTGDK
☑	424	776.8488	1551.6830	1551.7199	-23.73	1	8	6.3	1	CKQLESTQTESNK
☑	632	758.4205	2272.2397	2272.1311	47.8	1	8	4.3	1	GFGFVTFMDQAGVDKVLAQSR
☑	371	727.8940	1453.7734	1453.7776	-2.89	0	8	10	1	ISDAVAALDPVDLR
☑	53	327.7028	653.3910	653.3609	46.2	0	8	5.1	1	SAIAHR
☑	399	738.3895	1474.7644	1474.6948	47.2	1	7	12	1	WMVAGKADPEMPK + Oxidation (M)
☑	146	517.2634	1032.5122	1032.4724	38.6	0	7	16	1	VLDEEGSER
☑	273	631.8104	1261.6062	1261.6150	-6.91	1	7	14	1	EASESQNLEKK
☑	453	785.8453	1569.6760	1569.7497	-46.93	1	7	5.7	1	GEMFVLEKGEYPR + Oxidation (M)
☑	676	753.4116	3009.6173	3009.6136	1.21	1	7	4.3	1	YLEYLFDVELAPPTLVLTQMEKILR + Oxidation (M)
☑	215	590.7988	1179.5830	1179.6111	-23.74	0	7	16	1	TYVCHIIFK
☑	361	714.3387	1426.6628	1426.7173	-38.19	1	7	10	1	CAPCRGNGVPVLK
☑	147	517.2635	1032.5124	1032.4724	38.8	0	7	18	1	VLDEEGSER
☑	307	659.3557	1316.6968	1316.6473	37.6	1	7	16	1	DAREAGFALDPR
☑	247	616.3654	1230.7162	1230.7006	12.7	0	7	7.4	1	QSVVVLMGSLAK
☑	484	801.9233	1601.8320	1601.8534	-13.34	1	7	14	1	MPLVHMSFLSIRR + Oxidation (M)
☑	624	737.7004	2210.0794	2210.0585	9.43	1	7	11	1	IITQNALHRAPHYNSCCR
☑	117	435.7735	869.5324	869.4971	40.7	0	7	8.7	1	AVVESLPR
☑	595	693.9965	2078.9677	2078.9700	-1.11	1	6	10	1	MQQHCDELMNLLKTFR + Oxidation (M)
☑	526	824.3863	1646.7580	1646.7747	-10.12	1	6	13	1	ARLGEASDSELADADK
☑	71	379.7092	757.4038	757.4082	-5.81	1	6	21	1	DKGVPSR
☑	45	619.2937	618.2864	618.2973	-17.57	0	6	19	1	IEDSR
☑	555	879.9559	1757.8972	1757.8916	3.19	1	6	16	1	QPCILLMDSLRGPSR + Oxidation (M)
☑	385	738.3738	1474.7330	1474.7780	-30.45	0	6	19	1	SYLRPITEAPSNK
☑	354	701.8492	1401.6838	1401.6783	3.97	1	6	17	1	TRPPKEQSSGCR
☑	116	435.7733	869.5320	869.5222	11.3	0	6	11	1	VTLEALPK
☑	397	738.3874	1474.7602	1474.7524	5.34	0	6	20	1	LPAAGVGDMVMATVK + Oxidation (M)
☑	261	625.3146	1248.6146	1248.5809	27.1	0	6	23	1	EGEWQTMIQK
☑	607	707.3361	2118.9865	2119.0629	-36.04	1	6	13	1	CMGPVMVGLRLPLTDVSQF
☑	144	516.3068	1030.5990	1030.5811	17.4	1	5	15	1	AKWLSVAEK
☑	477	795.8834	1589.7522	1589.8096	-36.08	1	5	17	1	SAAGLSMARAVSWQR
☑	279	639.3420	1276.6694	1276.6598	7.55	0	5	24	1	VVVQNNLCFGK
☑	294	647.3664	1292.7182	1292.6976	16.0	0	5	14	1	ISYEPITTTLR
☑	444	785.8418	1569.6690	1569.6399	18.6	1	5	7.8	1	MKTECDSEQGETR
☑	364	719.3223	1436.6300	1436.6783	-33.60	0	5	12	1	DNDSYINVQEIK
☑	571	897.9120	1793.8094	1793.8189	-5.28	1	5	14	1	MSGGGGDDVVCTGWLRK
☑	351	692.3663	1382.7180	1382.7194	-0.98	1	5	21	1	RDIVFAASLYTQ
☑	479	795.8920	1589.7694	1589.8096	-25.27	1	5	21	1	SAAGLSMARAVSWQR
☑	135	505.2794	1008.5442	1008.5716	-27.11	1	5	20	1	AKAHILNDK
☑	239	614.8431	1227.6716	1227.6625	7.43	1	5	24	1	RWGHTVFVAR
☑	510	816.8969	1631.7792	1631.7205	36.0	0	5	23	1	MMTTATSGNIMTTEK + Oxidation (M)
☑	277	633.3164	1264.6182	1264.6187	-0.33	1	5	26	1	DELEFEKEVK
☑	330	672.3507	1342.6868	1342.6915	-3.46	0	5	27	1	MPTGTTVNPAGIGK
☑	271	631.8051	1261.5956	1261.5826	10.3	0	4	24	1	EALEVDWSGEK
☑	226	599.8269	1197.6392	1197.6183	17.5	0	4	23	1	FLFTTGFHTK
☑	142	516.3029	1030.5912	1030.5519	38.2	1	4	25	1	SARAAEAVTR
☑	169	533.2592	1064.5038	1064.4774	24.8	0	4	26	1	DDVAQENFK
☑	360	711.3236	1420.6326	1420.6318	0.63	0	4	16	1	LTGASSDVEEEER
☑	591	1011.4652	2020.9158	2020.9459	-14.85	0	4	13	1	SMSQIYCIENAHGQLVR + Oxidation (M)
☑	342	688.3369	1374.6592	1374.6449	10.4	0	4	26	1	EPGETIVEAMQR + Oxidation (M)
☑	694	855.1420	3416.5389	3416.6612	-35.79	1	4	8.7	1	TTNEGLLYPNPAAQMLVGDSFARHYYFLGR + Oxidation (M)
☑	94	414.2219	826.4292	826.4661	-44.57	0	4	22	1	VAAPNLSR
☑	262	625.3168	1248.6190	1248.5809	30.6	0	4	32	1	DDGMQIWGAIK + Oxidation (M)
☑	572	897.9121	1793.8096	1793.7699	22.2	1	4	19	1	MSYLKTTMEDEESSK + Oxidation (M)
☑	573	897.9143	1793.8140	1793.7699	24.6	1	4	20	1	MSYLKTTMEDEESSK + Oxidation (M)
☑	466	785.8697	1569.7248	1569.7132	7.44	0	4	22	1	EVQESQTQGSQGHR
☑	400	738.3921	1474.7696	1474.7991	-19.95	1	4	29	1	AIEAASSLKSQAATK
☑	516	816.8981	1631.7816	1631.8301	-29.68	1	4	29	1	KVASGSAAAAAGPSCSLK
☑	517	816.8984	1631.7822	1631.7205	37.9	0	3	30	1	MMTTATSGNIMTTEK + Oxidation (M)
☑	394	738.3853	1474.7560	1474.7528	2.19	0	3	34	1	SGAGSHATHELPAIK
☑	626	738.0252	2211.0538	2211.0379	7.20	0	3	23	1	GITSFIQHPASTPCEEANPR
☑	167	530.7861	1059.5576	1059.5358	20.7	0	3	50	1	WPMLLNMR
☑	384	738.3727	1474.7308	1474.6737	38.7	0	3	36	1	MFWNLMPFTTR + 2 Oxidation (M)
☑	638	786.3861	2356.1365	2356.0868	21.1	0	3	21	1	EGQLYAMELQGFWMDIGQPK + Oxidation (M)
☑	570	897.9114	1793.8082	1793.7699	21.4	1	3	22	1	MSYLKTTMEDEESSK + Oxidation (M)
☑	512	816.8972	1631.7798	1631.7865	-4.07	1	3	32	1	CNKEIYVVTESYK
☑	138	513.7487	1025.4828	1025.5142	-30.53	0	3	31	1	QPDTPAELR
☑	675	752.1354	3004.5125	3004.5943	-27.24	1	3	16	1	TFQTALFLDRLLDGSPLPQEVVMSLSK
☑	134	505.2752	1008.5358	1008.5127	22.9	0	3	34	1	EYLESLQK
☑	21	517.2652	516.2579	516.2656	-14.89	0	3	45	1	AGDVR
☑	644	801.7284	2402.1634	2402.1570	2.67	1	3	27	1	SGLCIEANSRIMELDPNDGALK
☑	576	901.4456	1800.8766	1800.8469	16.5	0	3	37	1	LMPMFLMVMPGMISR + 3 Oxidation (M)
☑	119	435.7748	869.5350	869.5123	26.1	0	2	22	1	VSLWLPR
☑	199	574.3331	1146.6516	1146.6649	-11.52	0	2	27	1	VSATFLPTAIK

☑	218	590.8070	1179.5994	1179.5554	37.4	1	2	45	1	AVTKENSEMR + Oxidation (M)
☑	569	897.9113	1793.8080	1793.7672	22.8	1	2	26	1	CDLNMQQQEEEKAR + Oxidation (M)
☑	525	824.3836	1646.7526	1646.8055	-32.08	1	2	32	1	GLRTLCCCLPIER
☑	276	632.3409	1262.6672	1262.7095	-33.45	0	2	44	1	SRPPALEPGALR
☑	292	644.8557	1287.6968	1287.6459	39.6	1	2	42	1	AVTVEFKHDDK
☑	210	587.3012	1172.5878	1172.6149	-23.09	0	2	56	1	LNQEVSQLSR
☑	405	747.8829	1493.7512	1493.6966	36.6	1	2	40	1	NANNINMLKDGMK + 2 Oxidation (M)
☑	356	707.3298	1412.6450	1412.7129	-48.02	1	2	31	1	CRPPRCDLLAR
☑	408	757.8497	1513.6848	1513.7307	-30.29	1	2	30	1	THEAQIQEMRQK + Oxidation (M)
☑	471	1572.7390	1571.7317	1571.7171	9.30	1	2	38	1	KMGEMQLSSVTDSK + 2 Oxidation (M)
☑	415	766.8577	1531.7008	1531.7743	-47.95	0	2	35	1	EVQQHALVHQESK
☑	140	514.2820	1026.5494	1026.5757	-25.53	1	2	45	1	RLMTLTHR
☑	153	523.2803	1044.5460	1044.5638	-16.95	0	2	68	1	VLEAPMTIR + Oxidation (M)
☑	617	715.3487	2143.0243	2143.0831	-27.44	1	1	36	1	CPKVSVATPDVSLEASEGAVK
☑	168	533.2479	1064.4812	1064.5219	-38.18	1	1	37	1	MAAQMVRSR + Oxidation (M)
☑	93	414.2217	826.4288	826.4661	-45.05	0	1	40	1	VAAPNLSR
☑	478	795.8888	1589.7630	1589.7475	9.81	0	1	47	1	SGPLFSFDVHDDVR
☑	143	516.3063	1030.5980	1030.5546	42.1	0	1	40	1	VDILDIDTK
☑	275	632.3361	1262.6576	1262.6579	-0.17	1	1	54	1	LSSTPTATSRSR
☑	372	727.8994	1453.7842	1453.8252	-28.19	1	1	42	1	ALELENDRLLLR
☑	544	859.4189	1716.8232	1716.8519	-16.67	0	1	49	1	CVVPGQAHAQVTWHK
☑	428	781.3944	1560.7742	1560.7330	26.4	1	1	54	1	VYSMRFCPFAQR
☑	410	757.8604	1513.7062	1513.6323	48.8	1	1	42	1	MNSVGEACTDMKR + Oxidation (M)
☑	64	725.4449	724.4376	724.4020	49.1	1	1	23	1	KQFFR
☑	166	530.7843	1059.5540	1059.5673	-12.47	1	1	91	1	KQEGIVGSSR
☑	256	618.3239	1234.6332	1234.5942	31.6	1	1	68	1	QNGDRFIEEK
☑	139	514.2798	1026.5450	1026.5393	5.64	1	1	56	1	VALRHMER + Oxidation (M)
☑	152	519.2725	1036.5304	1036.5777	-45.63	1	0	69	1	QAAPREPLR
☑	666	958.1300	2871.3682	2871.3536	5.07	1	0	33	1	DVFQRNQQDGQLEESPGSWPGAGTIR
☑	568	897.9097	1793.8048	1793.8651	-33.60	0	0	43	1	LMAEALAMIESTQQAR + 2 Oxidation (M)
☑	1	341.0242	340.0169							
☑	2	341.0275	340.0202							
☑	3	342.2688	341.2615							
☑	4	360.9891	359.9818							
☑	5	416.2400	415.2327							
☑	6	424.3148	423.3075							
☑	7	429.0965	428.0892							
☑	8	429.0978	428.0905							
☑	9	430.2511	429.2438							
☑	10	445.1122	444.1049							
☑	11	445.1232	444.1159							
☑	12	445.1351	444.1278							
☑	13	451.1725	450.1652							
☑	14	459.2731	458.2658							
☑	15	469.1872	468.1799							
☑	16	469.1905	468.1832							
☑	17	470.2026	469.1953							
☑	18	492.2361	491.2288							
☑	19	503.0361	502.0288							
☑	20	503.3152	502.3079							
☑	22	521.0000	519.9927							
☑	23	522.9882	521.9809							
☑	24	533.2681	532.2608							
☑	25	545.3068	544.2995							
☑	26	549.0557	548.0484							
☑	27	553.0099	552.0026							
☑	28	558.7847	557.7774							
☑	29	562.2796	561.2723							
☑	30	565.0400	564.0327							
☑	31	565.3137	564.3064							
☑	32	572.3085	571.3012							
☑	33	579.0262	578.0189							
☑	34	579.2961	578.2888							
☑	35	585.0348	584.0275							
☑	36	585.0372	584.0299							
☑	37	587.0353	586.0280							
☑	38	590.3307	589.3234							
☑	39	595.0097	594.0024							
☑	40	595.2759	594.2686							
☑	41	616.3271	615.3198							
☑	42	616.3747	615.3674							
☑	43	617.2840	616.2767							
☑	44	617.3132	616.3059							
☑	46	620.7597	619.7524							
☑	48	632.3674	631.3601							
☑	49	634.9940	633.9867							
☑	50	638.3274	637.3201							

51	643.4092	642.4019
52	327.6973	653.3800
54	665.3381	664.3308
55	665.3527	664.3454
56	668.9276	667.9203
58	694.3491	693.3418
59	350.1557	698.2968
60	707.3477	706.3404
61	723.3727	722.3654
62	363.2188	724.4230
65	729.7198	728.7125
66	729.8578	728.8505
68	743.4255	742.4182
69	745.9082	744.9009
70	757.3588	756.3515
74	766.4128	765.4055
75	766.6998	765.6925
76	774.3881	773.3808
77	775.8660	774.8587
78	391.2271	780.4396
80	787.7332	786.7259
81	793.4234	792.4161
82	793.4310	792.4237
83	794.3906	793.3833
84	802.3954	801.3881
85	804.7424	803.7351
87	819.4597	818.4524
92	826.5800	825.5727
95	414.2317	826.4488
96	828.1093	827.1020
97	839.7756	838.7683
101	421.7563	841.4980
106	846.4387	845.4314
107	847.7014	846.6941
108	855.3943	854.3870
111	861.4664	860.4591
112	431.7305	861.4464
113	431.7315	861.4484
114	435.7690	869.5234
121	449.2104	896.4062
122	899.4448	898.4375
123	908.7192	907.7119
124	924.6996	923.6923
125	935.0483	934.0410
126	470.7675	939.5204
129	958.4771	957.4698
131	487.2795	972.5444
132	493.2934	984.5722
133	505.2740	1008.5334
136	505.7445	1009.4744
137	1021.3894	1020.3821
141	515.3065	1028.5984
151	519.2700	1036.5254
161	1053.5415	1052.5342
162	528.3117	1054.6088
163	530.7632	1059.5118
164	530.7643	1059.5140
171	537.2878	1072.5610
174	545.7690	1089.5234
176	546.7556	1091.4966
177	546.7605	1091.5064
178	553.2914	1104.5682
180	554.2919	1106.5692
181	556.7847	1111.5548
182	564.8070	1127.5994
183	565.3002	1128.5858
184	565.3081	1128.6016
185	565.3114	1128.6082
186	1135.5474	1134.5401
187	1137.5103	1136.5030
188	572.3028	1142.5910
189	572.3290	1142.6434
190	574.3148	1146.6150
191	574.3186	1146.6226
192	574.3219	1146.6292
193	574.3220	1146.6294
194	574.3242	1146.6338

☑	195	574.3255	1146.6364
☑	200	574.3392	1146.6638
☑	201	574.3395	1146.6644
☑	202	574.8029	1147.5912
☑	203	578.8290	1155.6434
☑	204	579.3042	1156.5938
☑	205	579.3061	1156.5976
☑	206	1158.7262	1157.7189
☑	207	580.3287	1158.6428
☑	208	582.3223	1162.6300
☑	209	582.3409	1162.6672
☑	211	587.3020	1172.5894
☑	212	588.3432	1174.6718
☑	213	589.8100	1177.6054
☑	214	590.2994	1178.5842
☑	216	590.7989	1179.5832
☑	217	590.8007	1179.5868
☑	219	590.8085	1179.6024
☑	220	592.2846	1182.5546
☑	221	1186.3834	1185.3761
☑	222	593.8180	1185.6214
☑	225	596.7852	1191.5558
☑	227	599.8465	1197.6784
☑	228	599.8491	1197.6836
☑	230	602.3141	1202.6136
☑	231	602.3174	1202.6202
☑	232	1204.8134	1203.8061
☑	233	604.8074	1207.6002
☑	235	606.8433	1211.6720
☑	236	606.8468	1211.6790
☑	237	608.3107	1214.6068
☑	238	612.7756	1223.5366
☑	242	615.3013	1228.5880
☑	244	615.3051	1228.5956
☑	246	616.3533	1230.6920
☑	249	616.7987	1231.5828
☑	250	616.8149	1231.6152
☑	251	616.8370	1231.6594
☑	252	617.8182	1233.6218
☑	253	618.2675	1234.5204
☑	255	618.3215	1234.6284
☑	257	624.2947	1246.5748
☑	259	625.3065	1248.5984
☑	260	625.3119	1248.6092
☑	263	625.3206	1248.6266
☑	264	626.3072	1250.5998
☑	265	627.7880	1253.5614
☑	266	629.9380	1257.8614
☑	269	631.3054	1260.5962
☑	272	631.8072	1261.5998
☑	278	636.3246	1270.6346
☑	280	639.3491	1276.6836
☑	283	639.3563	1276.6980
☑	285	639.3596	1276.7046
☑	287	639.8105	1277.6064
☑	288	428.7632	1283.2678
☑	289	643.8198	1285.6250
☑	290	644.7901	1287.5656
☑	291	644.8137	1287.6128
☑	293	646.3283	1290.6420
☑	295	648.3762	1294.7378
☑	296	651.8530	1301.6914
☑	297	651.8588	1301.7030
☑	303	653.7860	1305.5574
☑	304	657.3508	1312.6870
☑	305	657.8543	1313.6940
☑	306	659.3350	1316.6554
☑	308	659.3590	1316.7034
☑	310	662.8101	1323.6056
☑	311	662.8180	1323.6214
☑	312	664.8234	1327.6322
☑	314	665.3420	1328.6694
☑	315	665.3454	1328.6762
☑	317	665.3479	1328.6812
☑	318	665.3576	1328.7006
☑	319	665.3637	1328.7128
☑	322	668.3506	1334.6866

323	669.8050	1337.5954
324	670.3049	1338.5952
325	670.3211	1338.6276
326	670.8461	1339.6776
328	671.3350	1340.6554
329	671.3575	1340.7004
331	672.3639	1342.7132
332	672.8434	1343.6722
333	674.8414	1347.6682
334	675.3286	1348.6426
335	676.9155	1351.8164
336	677.8329	1353.6512
337	680.3687	1358.7228
338	681.3326	1360.6506
339	682.3409	1362.6672
340	685.3844	1368.7542
341	687.3656	1372.7166
343	688.3516	1374.6886
346	691.3408	1380.6670
347	692.3462	1382.6778
349	692.3532	1382.6918
350	692.3560	1382.6974
353	698.8453	1395.6760
355	702.8401	1403.6656
357	708.9245	1415.8344
358	710.8427	1419.6708
359	711.3199	1420.6252
362	714.7645	1427.5144
363	715.8468	1429.6790
365	719.3273	1436.6400
366	720.3449	1438.6752
367	720.8420	1439.6694
368	722.8765	1443.7384
369	722.8981	1443.7816
370	723.3694	1444.7242
374	730.8651	1459.7156
377	730.8934	1459.7722
379	730.8994	1459.7842
380	730.9032	1459.7918
381	733.3936	1464.7726
382	735.8239	1469.6332
383	737.8687	1473.7228
386	738.3757	1474.7368
387	738.3763	1474.7380
389	738.3772	1474.7398
390	738.3773	1474.7400
395	738.3854	1474.7562
396	738.3863	1474.7580
398	738.3889	1474.7632
402	740.3651	1478.7156
403	746.8673	1491.7200
404	747.8588	1493.7030
406	748.8496	1495.6846
407	748.8518	1495.6890
409	757.8577	1513.7008
411	758.4211	1514.8276
412	759.4084	1516.8022
413	759.4500	1516.8854
414	766.8566	1531.6986
416	766.8604	1531.7062
419	773.8304	1545.6462
420	774.8945	1547.7744
421	775.3884	1548.7622
422	775.8660	1549.7174
423	775.8663	1549.7180
425	1552.7470	1551.7397
426	778.8869	1555.7592
427	778.9056	1555.7966
429	783.8977	1565.7808
430	784.8284	1567.6422
431	785.3452	1568.6758
432	785.3531	1568.6916
433	785.8189	1569.6232
434	785.8344	1569.6542
435	785.8349	1569.6552
437	785.8361	1569.6576
438	785.8381	1569.6616

439	785.8392	1569.6638
440	785.8394	1569.6642
441	785.8402	1569.6658
442	785.8404	1569.6662
443	785.8405	1569.6664
445	785.8419	1569.6692
446	785.8422	1569.6698
447	785.8422	1569.6698
448	785.8430	1569.6714
449	785.8434	1569.6722
450	785.8450	1569.6754
451	785.8452	1569.6758
452	785.8452	1569.6758
454	785.8458	1569.6770
455	785.8459	1569.6772
456	785.8461	1569.6776
457	785.8469	1569.6792
458	785.8471	1569.6796
459	785.8486	1569.6826
460	785.8491	1569.6836
461	785.8521	1569.6896
462	785.8521	1569.6896
463	785.8529	1569.6912
464	785.8530	1569.6914
465	785.8535	1569.6924
467	786.3513	1570.6880
468	786.3592	1570.7038
469	786.8524	1571.6902
470	786.8537	1571.6928
472	793.4410	1584.8674
473	794.8373	1587.6600
474	794.8431	1587.6716
475	794.8470	1587.6794
476	795.8804	1589.7462
480	801.9072	1601.7998
481	801.9081	1601.8016
482	801.9114	1601.8082
483	801.9171	1601.8196
485	803.8721	1605.7296
486	803.8863	1605.7580
487	809.8927	1617.7708
488	809.9073	1617.8000
489	810.8683	1619.7220
490	810.8767	1619.7388
492	816.3729	1630.7312
494	816.8885	1631.7624
495	816.8889	1631.7632
499	816.8913	1631.7680
506	816.8958	1631.7770
509	816.8964	1631.7782
513	816.8972	1631.7798
520	816.9018	1631.7890
522	816.9097	1631.8048
523	818.8920	1635.7694
524	824.3835	1646.7524
527	827.7864	1653.5582
529	834.9656	1667.9166
530	839.8098	1677.6050
531	843.3881	1684.7616
532	849.9309	1697.8472
533	851.3857	1700.7568
536	851.4559	1700.8972
537	851.4569	1700.8992
538	851.4605	1700.9064
539	851.4640	1700.9134
540	854.3861	1706.7576
541	854.3962	1706.7778
542	854.4028	1706.7910
543	428.7939	1711.1465
545	859.4514	1716.8882
546	864.9410	1727.8674
547	864.9565	1727.8984
548	870.4334	1738.8522
549	870.8630	1739.7114
550	872.4373	1742.8600
551	878.4237	1754.8328
552	878.9592	1755.9038

553	879.9443	1757.8740
556	883.8915	1765.7684
558	883.9031	1765.7916
559	886.4130	1770.8114
560	888.4164	1774.8182
561	445.0783	1776.2841
562	595.3426	1783.0060
563	892.8724	1783.7302
564	892.8902	1783.7658
567	896.3662	1790.7178
574	898.4035	1794.7924
575	900.1204	1798.2262
578	913.4081	1824.8016
579	913.9026	1825.7906
581	913.9164	1825.8182
582	914.4834	1826.9522
583	917.4860	1832.9574
584	917.4905	1832.9664
585	918.5268	1835.0390
586	929.0120	1856.0094
587	937.9552	1873.8958
588	959.0217	1916.0288
589	973.5071	1944.9996
590	979.4918	1956.9690
592	678.8873	2033.6401
593	688.3813	2062.1221
594	1039.0304	2076.0462
596	1040.9813	2079.9480
597	1040.9854	2079.9562
598	697.7272	2090.1598
599	704.9998	2111.9776
600	705.0000	2111.9782
602	705.0046	2111.9920
603	1057.0109	2112.0072
604	705.3431	2113.0075
605	707.3242	2118.9508
606	707.3287	2118.9643
608	707.3390	2118.9952
609	707.3412	2119.0018
610	707.9768	2120.9086
611	708.3425	2122.0057
612	712.6883	2135.0431
613	715.3429	2143.0069
614	715.3459	2143.0159
615	1072.5183	2143.0220
616	1072.5190	2143.0234
618	1072.5215	2143.0284
619	1072.5273	2143.0400
620	1072.5293	2143.0440
621	1074.9668	2147.9190
622	723.3387	2166.9943
623	737.6983	2210.0731
625	737.7109	2210.1109
627	743.4150	2227.2232
628	745.4305	2233.2697
629	748.0370	2241.0892
630	752.6371	2254.8895
631	757.3587	2269.0543
633	762.0526	2283.1360
634	769.3136	2304.9190
635	773.5890	2317.7452
636	776.3817	2326.1233
637	776.3966	2326.1680
639	1186.5781	2371.1416
640	1187.2501	2372.4856
641	795.3820	2383.1242
642	1193.6521	2385.2896
643	801.3739	2401.0999
645	1204.4891	2406.9636
646	1206.2017	2410.3888
647	813.3870	2437.1392
648	1233.2035	2464.3924
649	828.0746	2481.2020
650	843.8010	2528.3812
651	857.3363	2568.9871
652	859.6450	2575.9132
653	875.4052	2623.1938

☑	654	670.4191	2677.6473
☑	655	896.6437	2686.9093
☑	656	899.5475	2695.6207
☑	657	900.0706	2697.1900
☑	658	915.8900	2744.6482
☑	659	918.9715	2753.8927
☑	660	929.7820	2786.3242
☑	661	930.1228	2787.3466
☑	662	706.6091	2822.4073
☑	663	958.1210	2871.3412
☑	664	958.1260	2871.3562
☑	665	958.1278	2871.3616
☑	667	961.4618	2881.3636
☑	668	973.1750	2916.5032
☑	669	732.3851	2925.5113
☑	670	737.3884	2945.5245
☑	671	742.2606	2965.0133
☑	672	748.3859	2989.5145
☑	673	748.4033	2989.5841
☑	674	749.1548	2992.5901
☑	677	758.6400	3030.5309
☑	678	1016.1148	3045.3226
☑	679	1022.4659	3064.3759
☑	680	1031.4298	3091.2676
☑	681	774.1323	3092.5001
☑	682	776.1229	3100.4625
☑	683	785.6156	3138.4333
☑	684	786.3461	3141.3553
☑	685	789.1204	3152.4525
☑	686	797.1930	3184.7429
☑	687	810.3776	3237.4813
☑	688	810.3818	3237.4981
☑	689	1085.5421	3253.6045
☑	690	816.9273	3263.6801
☑	691	823.1737	3288.6657
☑	692	829.1945	3312.7489
☑	693	1119.1757	3354.5053
☑	695	858.6205	3430.4529
☑	696	869.3155	3473.2329
☑	697	870.4107	3477.6137
☑	698	873.2922	3489.1397
☑	699	1176.4674	3526.3804
☑	700	886.1975	3540.7609
☑	701	889.4472	3553.7597
☑	702	908.3743	3629.4681
☑	703	914.8817	3655.4977
☑	704	930.3966	3717.5573
☑	705	932.3429	3725.3425
☑	706	944.9282	3775.6837
☑	707	959.3601	3833.4113
☑	708	962.2110	3844.8149
☑	709	968.3356	3869.3133
☑	710	969.9287	3875.6857
☑	711	982.5540	3926.1869
☑	712	1342.2548	4023.7426
☑	713	1011.4422	4041.7397
☑	714	1015.4938	4057.9461
☑	715	1078.9778	4311.8821
☑	716	1094.4548	4373.7901
☑	717	1133.5477	4530.1617
☑	718	1134.9469	4535.7585
☑	719	1135.4979	4537.9625
☑	720	1139.0413	4552.1361
☑	721	1169.6051	4674.3913
☑	722	1210.4652	4837.8317
☑	723	1225.5979	4898.3625
☑	724	1309.0933	5232.3441
☑	725	1359.3607	5433.4137

Search Parameters

```
Type of search         : MS/MS Ion Search
Enzyme                 : Trypsin
Fixed modifications    : Carbamidomethyl (C)
Variable modifications : Oxidation (M)
Mass values            : Monoisotopic
Protein Mass           : Unrestricted
Peptide Mass Tolerance : ± 50 ppm
```

```
Fragment Mass Tolerance: ± 0.2 Da
Max Missed Cleavages   : 1
Instrument type        : ESI-QUAD-TOF
Number of queries      : 725
```

Mascot: http://www.matrixscience.com/

Mascot Search Results

User : Dominic
Email : dominic.kurian@bbsrc.ac.uk
Search title : IPI Mouse_15-02-2010
MS data file : C:\Program Files\Matrix Science\Mascot Daemon\mgf\33 10753\mascot_daemon_merge.mgf
Database : IPI_mouse MOUSE_v3_79 (54943 sequences; 24917557 residues)
Timestamp : 15 Feb 2011 at 12:03:54 GMT
Protein hits : IPI00420725 Tax_Id=10090 Gene_Symbol=Camk2a Uncharacterized protein
IPI00474502 Tax_Id=10090 Gene_Symbol=Camk2b Calcium/calmodulin-dependent protein kinase type II subunit beta
IPI00117352 Tax_Id=10090 Gene_Symbol=Tubb5 Tubulin beta-5 chain
IPI00112584 Tax_Id=10090 Gene_Symbol=Camk2d Isoform 1 of Calcium/calmodulin-dependent protein kinase type II subunit
IPI00608020 Tax_Id=10090 Gene_Symbol=Ftl1 ferritin light chain 1
IPI00109061 Tax_Id=10090 Gene_Symbol=Tubb2b Tubulin beta-2B chain
IPI00169463 Tax_Id=10090 Gene_Symbol=Tubb2c Tubulin beta-2C chain
IPI00124695 Tax_Id=10090 Gene_Symbol=Camk2g Isoform 1 of Calcium/calmodulin-dependent protein kinase type II subunit
IPI00117348 Tax_Id=10090 Gene_Symbol=Tuba1b Tubulin alpha-1B chain
IPI00230145 Tax_Id=10090 Gene_Symbol=Fth1 Ferritin heavy chain
IPI00112251 Tax_Id=10090 Gene_Symbol=Tubb3 Tubulin beta-3 chain
IPI00115546 Tax_Id=10090 Gene_Symbol=Gnao1 Isoform Alpha-2 of Guanine nucleotide-binding protein G(o) subunit alpha
IPI00138892 Tax_Id=10090 Gene_Symbol=Uba52 Ubiquitin-60S ribosomal protein L40
IPI00122048 Tax_Id=10090 Gene_Symbol=Atp1a3 Sodium/potassium-transporting ATPase subunit alpha-3
IPI00121038 Tax_Id=10090 Gene_Symbol=Vcan Isoform V0 of Versican core protein
IPI00621468 Tax_Id=10090 Gene_Symbol=Gm7866 similar to ubiquitin A-52 residue ribosomal protein fusion product 1
IPI00110850 Tax_Id=10090 Gene_Symbol=Actb Actin, cytoplasmic 1
IPI00136929 Tax_Id=10090 Gene_Symbol=Actg1 Gamma actin-like protein
IPI00123176 Tax_Id=10090 Gene_Symbol=Gm6316 Glyceraldehyde-3-phosphate dehydrogenase
IPI00114642 Tax_Id=10090 Gene_Symbol=Hist1h2bn;Hist1h2bl;Hist1h2bj;Hist1h2bf Histone H2B type 1-F/J/L
IPI00380227 Tax_Id=10090 Gene_Symbol=Nrgn Neurogranin
IPI00135106 Tax_Id=10090 Gene_Symbol=Mobp Isoform 1 of Myelin-associated oligodendrocyte basic protein
IPI00221528 Tax_Id=10090 Gene_Symbol=Actbl2 Beta-actin-like protein 2
IPI00136703 Tax_Id=10090 Gene_Symbol=Ckb Creatine kinase B-type
IPI00404590 Tax_Id=10090 Gene_Symbol=H1f0 Putative uncharacterized protein
IPI00131366 Tax_Id=10090 Gene_Symbol=Krt6b Keratin, type II cytoskeletal 6B
IPI00348094 Tax_Id=10090 Gene_Symbol=Tubb1 Tubulin, beta 1
IPI00229525 Tax_Id=10090 Gene_Symbol=Gnal Guanine nucleotide-binding protein G(olf) subunit alpha
IPI00118569 Tax_Id=10090 Gene_Symbol=Gna13 Guanine nucleotide-binding protein subunit alpha-13
IPI00119067 Tax_Id=10090 Gene_Symbol=Tppp Tubulin polymerization-promoting protein

	IPI_mouse	Decoy	False discovery rate
Peptide matches above identity threshold	111	2	1.80 %
Peptide matches above homology or identity threshold	121	3	2.48 %

Probability Based Mowse Score

Ions score is -10*Log(P), where P is the probability that the observed match is a random event.
Individual ions scores > 37 indicate identity or extensive homology (p<0.01).
Protein scores are derived from ions scores as a non-probabilistic basis for ranking protein hits.

Score Distribution

Peptide Summary Report

Format As Peptide Summary Help

Significance threshold p< 0.01 Max. number of hits AUTO

Standard scoring MudPIT scoring Ions score or expect cut-off 0 Show sub-sets 0

Show pop-ups Suppress pop-ups Sort unassigned Decreasing Score Require bold red

Select All Select None Search Selected Error tolerant Archive Report

1. IPI00420725 **Mass:** 55882 **Score:** 921 **Queries matched:** 37 **emPAI:** 1.23
Tax_Id=10090 Gene_Symbol=Camk2a Uncharacterized protein
Check to include this hit in error tolerant search or archive report

	Query	Observed	Mr(expt)	Mr(calc)	ppm	Miss	Score	Expect	Rank	Peptide
☑	155	445.2853	444.2780	444.2696	18.9	0	5	23	1	K.GAAVK.L
☑	414	432.7276	863.4406	863.4501	-10.97	0	20	1.1	1	R.SGAPSVLPH.-
	424	446.2583	890.5020	890.5086	-7.40	1	8	12	2	K.GAFSVVRR.C
	441	452.2508	902.4870	902.4895	-2.74	0	4	41	2	K.MLTINPSK.R
☑	502	525.2626	1048.5106	1048.5553	-42.57	0	53	0.0004	1	K.VLAGQEYAAK.I
	503	525.2869	1048.5592	1048.5553	3.78	0	(3)	38	3	K.VLAGQEYAAK.I
☑	510	530.2837	1058.5528	1058.5906	-35.68	1	(40)	0.0088	1	K.MLTINPSKR.I
☑	511	530.2865	1058.5584	1058.5906	-30.39	1	(47)	0.002	1	K.MLTINPSKR.I

☑	512	530.2960	1058.5774	1058.5906	-12.44	1	(42)	0.0056	1	K.MLTINPSKR.I
☑	513	530.3043	1058.5940	1058.5906	3.24	1	47	0.0014	1	K.MLTINPSKR.I
☑	529	538.2988	1074.5830	1074.5855	-2.32	1	(43)	0.0044	1	K.MLTINPSKR.I + Oxidation (M)
☑	530	538.3007	1074.5868	1074.5855	1.22	1	(43)	0.0046	1	K.MLTINPSKR.I + Oxidation (M)
☑	531	538.3033	1074.5920	1074.5855	6.06	1	(44)	0.0033	1	K.MLTINPSKR.I + Oxidation (M)
☑	554	574.3305	1146.6464	1146.6431	2.95	0	(46)	0.0012	1	K.GAILTTMLATR.N
☑	563	582.3281	1162.6416	1162.6380	3.15	0	83	2.8e-007	1	K.GAILTTMLATR.N + Oxidation (M)
☑	564	582.3326	1162.6506	1162.6380	10.9	0	(10)	5	1	K.GAILTTMLATR.N + Oxidation (M)
☑	601	631.2774	1260.5402	1260.5927	-41.64	0	(42)	0.0017	1	R.FYFENLWSR.N
☑	602	631.2971	1260.5796	1260.5927	-10.39	0	54	0.00022	1	R.FYFENLWSR.N
☑	603	631.2980	1260.5814	1260.5927	-8.96	0	(8)	9.1	1	R.FYFENLWSR.N
☑	630	652.3443	1302.6740	1302.6932	-14.70	0	(49)	0.0011	1	R.ITQYLDAGGIPR.T
☑	631	652.3544	1302.6942	1302.6932	0.81	0	(61)	5.6e-005	1	R.ITQYLDAGGIPR.T
☑	632	652.3555	1302.6964	1302.6932	2.50	0	105	2.4e-009	1	R.ITQYLDAGGIPR.T
☑	656	670.8677	1339.7208	1339.7711	-37.49	0	(77)	1.1e-006	1	R.DLKPENLLLASK.L
☑	657	670.8842	1339.7538	1339.7711	-12.86	0	78	7.5e-007	1	R.DLKPENLLLASK.L
☑	658	447.5925	1339.7557	1339.7711	-11.49	0	(59)	6e-005	1	R.DLKPENLLLASK.L
☑	799	816.8861	1631.7576	1631.7719	-8.72	0	66	1.5e-005	1	R.FTEEYQLFEELGK.G
☑	800	816.8922	1631.7698	1631.7719	-1.25	0	(64)	2.3e-005	1	R.FTEEYQLFEELGK.G
☑	801	816.8947	1631.7748	1631.7719	1.82	0	(7)	12	1	R.FTEEYQLFEELGK.G
☑	802	816.8953	1631.7760	1631.7719	2.55	0	(63)	3.3e-005	1	R.FTEEYQLFEELGK.G
☑	883	1040.9640	2079.9134	2079.9426	-13.99	0	78	5.2e-007	1	K.AGAYDFPSPEWDTVTPEAK.D
☑	908	783.7256	2348.1550	2348.1689	-5.91	1	(26)	0.13	1	R.FTEEYQLFEELGKGAFSVVR.R
☑	909	783.7288	2348.1646	2348.1689	-1.82	1	125	1.7e-011	1	R.FTEEYQLFEELGKGAFSVVR.R
☑	910	783.7297	2348.1673	2348.1689	-0.67	1	(52)	0.00031	1	R.FTEEYQLFEELGKGAFSVVR.R
☑	928	888.7346	2663.1820	2663.2755	-35.12	1	(94)	1.1e-008	1	K.AGAYDFPSPEWDTVTPEAKDLINK.M
☑	929	1332.6436	2663.2726	2663.2755	-1.07	1	(59)	5.1e-005	1	K.AGAYDFPSPEWDTVTPEAKDLINK.M
☑	930	888.7660	2663.2762	2663.2755	0.25	1	100	4.3e-009	1	K.AGAYDFPSPEWDTVTPEAKDLINK.M
☑	931	888.7662	2663.2768	2663.2755	0.48	1	(95)	1.3e-008	1	K.AGAYDFPSPEWDTVTPEAKDLINK.M

Proteins matching the same set of peptides:
IPI00621806 **Mass:** 54651 **Score:** 921 **Queries matched:** 37
Tax_Id=10090 Gene_Symbol=Camk2a Isoform Alpha CaMKII of Calcium/calmodulin-dependent protein kinase type II subunit alpha

---

2. IPI00474502 **Mass:** 61164 **Score:** 816 **Queries matched:** 31 **emPAI:** 1.57
Tax_Id=10090 Gene_Symbol=Camk2b Calcium/calmodulin-dependent protein kinase type II subunit beta
☐ Check to include this hit in error tolerant search or archive report

	Query	Observed	Mr(expt)	Mr(calc)	ppm	Miss	Score	Expect	Rank	Peptide
	155	445.2853	444.2780	444.2696	18.9	0	5	23	1	K.GAAVK.L
	424	446.2583	890.5020	890.5086	-7.40	1	8	12	2	K.GAFSVVRR.C
☑	489	510.2411	1018.4676	1018.4688	-1.14	0	37	0.011	1	R.STVASMMHR.Q
☑	495	518.2553	1034.4960	1034.4637	31.2	0	(28)	0.13	1	R.STVASMMHR.Q + Oxidation (M)
☑	552	572.8067	1143.5988	1143.5964	2.12	0	(22)	0.54	1	R.FYFENLLAK.N
☑	553	572.8070	1143.5994	1143.5964	2.65	0	23	0.44	1	R.FYFENLLAK.N
	554	574.3305	1146.6464	1146.6431	2.95	0	(46)	0.0012	1	K.GAILTTMLATR.N
	563	582.3281	1162.6416	1162.6380	3.15	0	83	2.8e-007	1	K.GAILTTMLATR.N + Oxidation (M)
	564	582.3326	1162.6506	1162.6380	10.9	0	(10)	5	1	K.GAILTTMLATR.N + Oxidation (M)
☑	596	623.3240	1244.6334	1244.6361	-2.11	0	63	4e-005	1	K.ADGVKPQTNSTK.N
	656	670.8677	1339.7208	1339.7711	-37.49	0	(77)	1.1e-006	1	R.DLKPENLLLASK.C
	657	670.8842	1339.7538	1339.7711	-12.86	0	78	7.5e-007	1	R.DLKPENLLLASK.C
	658	447.5925	1339.7557	1339.7711	-11.49	0	(59)	6e-005	1	R.DLKPENLLLASK.C
	680	458.5831	1372.7275	1372.7310	-2.59	1	(2)	51	7	K.KADGVKPQTNSTK.N
☑	681	687.3759	1372.7372	1372.7310	4.53	1	41	0.0061	1	K.KADGVKPQTNSTK.N
☑	688	468.5813	1402.7221	1402.7317	-6.87	0	(26)	0.16	1	R.LTQYIDGQGRPR.T
☑	689	468.5816	1402.7230	1402.7317	-6.23	0	(40)	0.0067	1	R.LTQYIDGQGRPR.T
☑	690	702.3738	1402.7330	1402.7317	0.96	0	(37)	0.015	1	R.LTQYIDGQGRPR.T
☑	691	702.3776	1402.7406	1402.7317	6.38	0	51	0.00048	1	R.LTQYIDGQGRPR.T
☑	707	743.4136	1484.8126	1484.8021	7.13	0	75	1.7e-006	1	K.NLINQMLTINPAK.R + Oxidation (M)
☑	786	810.8771	1619.7396	1619.7355	2.56	0	66	1.4e-005	1	R.FTDEYQLYEDIGK.G
☑	787	810.8881	1619.7616	1619.7355	16.1	0	(29)	0.068	1	R.FTDEYQLYEDIGK.G
☑	794	542.6372	1624.8898	1624.9082	-11.37	1	89	5.7e-008	1	K.NLINQMLTINPAKR.I
☑	795	813.4636	1624.9126	1624.9082	2.71	1	(55)	9.8e-005	1	K.NLINQMLTINPAKR.I
	883	1040.9640	2079.9134	2079.9426	-13.99	0	78	5.2e-007	1	K.AGAYDFPSPEWDTVTPEAK.N
☑	890	1056.9772	2111.9398	2112.0011	-29.00	0	(1)	25	1	K.TTEQLIEAVNNGDFEAYAK.I
☑	891	1057.0093	2112.0040	2112.0011	1.40	0	95	1.6e-008	1	K.TTEQLIEAVNNGDFEAYAK.I
☑	906	779.7075	2336.1007	2336.1325	-13.61	1	(59)	5.7e-005	1	R.FTDEYQLYEDIGKGAFSVVR.R
☑	907	779.7144	2336.1214	2336.1325	-4.75	1	124	1.7e-011	1	R.FTDEYQLYEDIGKGAFSVVR.R
☑	911	1177.0757	2352.1368	2352.1301	2.86	0	128	7.5e-012	1	R.QTTAPATMSTAASGTTMGLVEQAK.S
☑	912	1185.0782	2368.1418	2368.1250	7.10	0	(118)	6.1e-011	1	R.QTTAPATMSTAASGTTMGLVEQAK.S + Oxidation (M)

Proteins matching the same set of peptides:
IPI00649778 **Mass:** 73827 **Score:** 816 **Queries matched:** 31
Tax_Id=10090 Gene_Symbol=Camk2b Calcium/calmodulin-dependent protein kinase II, beta, isoform CRA_b
IPI00875723 **Mass:** 68686 **Score:** 816 **Queries matched:** 31
Tax_Id=10090 Gene_Symbol=Camk2b 68 kDa protein

---

3. IPI00117352 **Mass:** 50095 **Score:** 504 **Queries matched:** 18 **emPAI:** 1.15
Tax_Id=10090 Gene_Symbol=Tubb5 Tubulin beta-5 chain
☐ Check to include this hit in error tolerant search or archive report

Query	Observed	Mr(expt)	Mr(calc)	ppm	Miss	Score	Expect	Rank	Peptide

	Query	Observed	Mr(expt)	Mr(calc)	ppm	Miss	Score	Expect	Rank	Peptide
☑	557	580.3218	1158.6290	1158.6219	6.13	0	48	0.0012	1	K.LAVNMVPFPR.L + Oxidation (M)
☑	643	660.3592	1318.7038	1318.6955	6.32	0	63	3.3e-005	1	R.IMNTFSVVPSPK.V
☑	653	668.3525	1334.6904	1334.6904	0.01	0	(8)	12	1	R.IMNTFSVVPSPK.V + Oxidation (M)
	654	668.3532	1334.6918	1334.6904	1.06	0	(4)	28	4	R.IMNTFSVVPSPK.V + Oxidation (M)
☑	783	808.4200	1614.8254	1614.8287	-2.02	0	(49)	0.00091	1	R.AILVDLEPGTMDSVR.S
☑	788	540.9453	1619.8141	1619.8283	-8.76	0	(53)	0.00031	1	R.LHFFMPGFAPLTSR.G
☑	789	810.9224	1619.8302	1619.8283	1.23	0	57	0.00013	1	R.LHFFMPGFAPLTSR.G
☑	790	810.9226	1619.8306	1619.8283	1.47	0	(45)	0.0019	1	R.LHFFMPGFAPLTSR.G
☑	797	816.4169	1630.8192	1630.8236	-2.68	0	59	0.0001	1	R.AILVDLEPGTMDSVR.S + Oxidation (M)
☑	808	830.4500	1658.8854	1658.8879	-1.50	0	62	3.2e-005	1	R.ALTVPELTQQVFDAK.N
☑	819	848.9158	1695.8170	1695.8257	-5.07	0	64	2.6e-005	1	K.NSSYFVEWIPNNVK.T
☑	820	848.9227	1695.8308	1695.8257	3.06	0	(47)	0.0013	1	K.NSSYFVEWIPNNVK.T
☑	877	653.6544	1957.9414	1957.9745	-16.94	0	(41)	0.0039	1	K.GHYTEGAELVDSVLDVVR.K
☑	878	980.0190	1958.0234	1957.9745	25.0	0	101	4.2e-009	1	K.GHYTEGAELVDSVLDVVR.K
☑	936	933.4480	2797.3222	2797.3361	-4.99	0	(89)	4.3e-008	1	R.SGPFGQIFRPDNFVFGQSGAGNNWAK.G
☑	937	933.4524	2797.3354	2797.3361	-0.27	0	99	5.2e-009	1	R.SGPFGQIFRPDNFVFGQSGAGNNWAK.G
☑	938	1399.6794	2797.3442	2797.3361	2.91	0	(71)	2.9e-006	1	R.SGPFGQIFRPDNFVFGQSGAGNNWAK.G
☑	939	933.4583	2797.3531	2797.3361	6.06	0	(34)	0.016	1	R.SGPFGQIFRPDNFVFGQSGAGNNWAK.G

---

4. IPI00112584 **Mass:** 56961 **Score:** 475 **Queries matched:** 21 **emPAI:** 0.66
Tax_Id=10090 Gene_Symbol=Camk2d Isoform 1 of Calcium/calmodulin-dependent protein kinase type II subunit delta
☐ Check to include this hit in error tolerant search or archive report

	Query	Observed	Mr(expt)	Mr(calc)	ppm	Miss	Score	Expect	Rank	Peptide
	155	445.2853	444.2780	444.2696	18.9	0	5	23	1	K.GAAVK.L
	424	446.2583	890.5020	890.5086	-7.40	1	8	12	2	K.GAFSVVRR.C
	489	510.2411	1018.4676	1018.4688	-1.14	0	37	0.011	1	R.STVASMMHR.Q
	495	518.2553	1034.4960	1034.4637	31.2	0	(28)	0.13	1	R.STVASMMHR.Q + Oxidation (M)
	510	530.2837	1058.5528	1058.5906	-35.68	1	6	24	3	K.MLTINPAKR.I + Oxidation (M)
	513	530.3043	1058.5940	1058.5906	3.24	1	(5)	20	8	K.MLTINPAKR.I + Oxidation (M)
☑	547	559.7811	1117.5476	1117.5444	2.92	0	20	0.77	1	R.FYFENALSK.S
☑	548	559.7846	1117.5546	1117.5444	9.18	0	(17)	1.8	1	R.FYFENALSK.S
	554	574.3305	1146.6464	1146.6431	2.95	0	(46)	0.0012	1	K.GAILTTMLATR.N
	563	582.3281	1162.6416	1162.6380	3.15	0	83	2.8e-007	1	K.GAILTTMLATR.N + Oxidation (M)
	564	582.3326	1162.6506	1162.6380	10.9	0	(10)	5	1	K.GAILTTMLATR.N + Oxidation (M)
	656	670.8677	1339.7208	1339.7711	-37.49	0	(77)	1.1e-006	1	R.DLKPENLLLASK.S
	657	670.8842	1339.7538	1339.7711	-12.86	0	78	7.5e-007	1	R.DLKPENLLLASK.S
	658	447.5925	1339.7557	1339.7711	-11.49	0	(59)	6e-005	1	R.DLKPENLLLASK.S
☑	785	809.8809	1617.7472	1617.7562	-5.56	0	52	0.00035	1	R.FTDEYQLFEELGK.G
	883	1040.9640	2079.9134	2079.9426	-13.99	0	78	5.2e-007	1	K.AGAYDFPSPEWDTVTPEAK.D
☑	905	779.0534	2334.1384	2334.1532	-6.36	1	26	0.11	1	R.FTDEYQLFEELGKGAFSVVR.R
	928	888.7346	2663.1820	2663.2755	-35.12	1	(94)	1.1e-008	1	K.AGAYDFPSPEWDTVTPEAKDLINK.M
	929	1332.6436	2663.2726	2663.2755	-1.07	1	(59)	5.1e-005	1	K.AGAYDFPSPEWDTVTPEAKDLINK.M
	930	888.7660	2663.2762	2663.2755	0.25	1	100	4.3e-009	1	K.AGAYDFPSPEWDTVTPEAKDLINK.M
	931	888.7662	2663.2768	2663.2755	0.48	1	(95)	1.3e-008	1	K.AGAYDFPSPEWDTVTPEAKDLINK.M

Proteins matching the same set of peptides:
IPI00406790 **Mass:** 60593 **Score:** 475 **Queries matched:** 21
Tax_Id=10090 Gene_Symbol=Camk2d Isoform 4 of Calcium/calmodulin-dependent protein kinase type II subunit delta
IPI00475044 **Mass:** 55899 **Score:** 475 **Queries matched:** 21
Tax_Id=10090 Gene_Symbol=Camk2d Isoform 2 of Calcium/calmodulin-dependent protein kinase type II subunit delta
IPI00828919 **Mass:** 58492 **Score:** 475 **Queries matched:** 21
Tax_Id=10090 Gene_Symbol=Camk2d Isoform 3 of Calcium/calmodulin-dependent protein kinase type II subunit delta
IPI00857865 **Mass:** 54664 **Score:** 475 **Queries matched:** 21
Tax_Id=10090 Gene_Symbol=Camk2d Putative uncharacterized protein
IPI00858128 **Mass:** 58283 **Score:** 475 **Queries matched:** 21
Tax_Id=10090 Gene_Symbol=Camk2d calcium/calmodulin-dependent protein kinase type II subunit delta isoform 1
IPI00858144 **Mass:** 55828 **Score:** 475 **Queries matched:** 21
Tax_Id=10090 Gene_Symbol=Camk2d 55 kDa protein

---

5. IPI00608020 **Mass:** 20817 **Score:** 452 **Queries matched:** 12 **emPAI:** 1.11
Tax_Id=10090 Gene_Symbol=Ftl1 ferritin light chain 1
☐ Check to include this hit in error tolerant search or archive report

	Query	Observed	Mr(expt)	Mr(calc)	ppm	Miss	Score	Expect	Rank	Peptide
☑	633	652.8418	1303.6690	1303.6633	4.42	1	29	0.097	1	R.LLEFQNDRGGR.A
☑	705	740.8561	1479.6976	1479.6954	1.55	0	(90)	6.4e-008	1	R.QNYSTEVEAAVNR.L
☑	706	740.8602	1479.7058	1479.6954	7.09	0	95	1.8e-008	1	R.QNYSTEVEAAVNR.L
☑	829	569.3081	1704.9025	1704.9271	-14.43	0	84	2e-007	1	K.NLNQALLDLHALGSAR.A
☑	830	569.3106	1704.9100	1704.9271	-10.03	0	(83)	2.1e-007	1	K.NLNQALLDLHALGSAR.A
☑	831	853.4784	1704.9422	1704.9271	8.90	0	(32)	0.018	1	K.NLNQALLDLHALGSAR.A
☑	832	853.4784	1704.9422	1704.9271	8.90	0	(31)	0.027	1	K.NLNQALLDLHALGSAR.A
☑	919	843.7637	2528.2693	2528.2772	-3.13	1	84	1.4e-007	1	R.RVAGPQPAQTGAPQGSLGEYLFER.L
☑	953	820.6315	3278.4969	3278.5587	-18.86	1	(72)	1.3e-006	1	R.ALFQDVQKPSQDEWGKTQEAMEAALAMEK.N
☑	954	820.6387	3278.5257	3278.5587	-10.07	1	(20)	0.28	1	R.ALFQDVQKPSQDEWGKTQEAMEAALAMEK.N
☑	955	820.6396	3278.5293	3278.5587	-8.97	1	(79)	3.8e-007	1	R.ALFQDVQKPSQDEWGKTQEAMEAALAMEK.N
☑	957	820.6479	3278.5625	3278.5587	1.15	1	84	1.1e-007	1	R.ALFQDVQKPSQDEWGKTQEAMEAALAMEK.N

Proteins matching the same set of peptides:
IPI00762203 **Mass:** 20847 **Score:** 452 **Queries matched:** 12
Tax_Id=10090 Gene_Symbol=Ftl1 Ferritin light chain 1

---

6. IPI00109061 **Mass:** 50377 **Score:** 449 **Queries matched:** 16 **emPAI:** 0.88

Tax_Id=10090 Gene_Symbol=Tubb2b Tubulin beta-2B chain

☐ Check to include this hit in error tolerant search or archive report

Query	Observed	Mr(expt)	Mr(calc)	ppm	Miss	Score	Expect	Rank	Peptide
557	580.3218	1158.6290	1158.6219	6.13	0	48	0.0012	1	K.LAVNMVPFPR.L + Oxidation (M)
783	808.4200	1614.8254	1614.8287	-2.02	0	(49)	0.00091	1	R.AILVDLEPGTMDSVR.S
788	540.9453	1619.8141	1619.8283	-8.76	0	(53)	0.00031	1	R.LHFFMPGFAPLTSR.G
789	810.9224	1619.8302	1619.8283	1.23	0	57	0.00013	1	R.LHFFMPGFAPLTSR.G
790	810.9226	1619.8306	1619.8283	1.47	0	(45)	0.0019	1	R.LHFFMPGFAPLTSR.G
797	816.4169	1630.8192	1630.8236	-2.68	0	59	0.0001	1	R.AILVDLEPGTMDSVR.S + Oxidation (M)
819	848.9158	1695.8170	1695.8257	-5.07	0	64	2.6e-005	1	K.NSSYFVEWIPNNVK.T
820	848.9227	1695.8308	1695.8257	3.06	0	(47)	0.0013	1	K.NSSYFVEWIPNNVK.T
833	854.4404	1706.8662	1706.8549	6.64	0	28	0.092	2	R.ALTVPELTQQMFDSK.N
877	653.6544	1957.9414	1957.9745	-16.94	0	(41)	0.0039	1	K.GHYTEGAELVDSVLDVVR.K
878	980.0190	1958.0234	1957.9745	25.0	0	101	4.2e-009	1	K.GHYTEGAELVDSVLDVVR.K
936	933.4480	2797.3222	2797.3361	-4.99	0	(89)	4.3e-008	1	R.SGPFGQIFRPDNFVFGQSGAGNNWAK.G
937	933.4524	2797.3354	2797.3361	-0.27	0	99	5.2e-009	1	R.SGPFGQIFRPDNFVFGQSGAGNNWAK.G
938	1399.6794	2797.3442	2797.3361	2.91	0	(71)	2.9e-006	1	R.SGPFGQIFRPDNFVFGQSGAGNNWAK.G
939	933.4583	2797.3531	2797.3361	6.06	0	(34)	0.016	1	R.SGPFGQIFRPDNFVFGQSGAGNNWAK.G
☑ 951	776.3458	3101.3541	3101.4003	-14.89	0	9	2.8	1	K.FWEVISDEHGIDPTGSYHGDSDLQLER.I

Proteins matching the same set of peptides:
IPI00338039 **Mass:** 50274 **Score:** 449 **Queries matched:** 16
Tax_Id=10090 Gene_Symbol=Tubb2a Tubulin beta-2A chain

---

7. IPI00169463 **Mass:** 50255 **Score:** 446 **Queries matched:** 17 **emPAI:** 1.01
Tax_Id=10090 Gene_Symbol=Tubb2c Tubulin beta-2C chain

☐ Check to include this hit in error tolerant search or archive report

Query	Observed	Mr(expt)	Mr(calc)	ppm	Miss	Score	Expect	Rank	Peptide
557	580.3218	1158.6290	1158.6219	6.13	0	48	0.0012	1	K.LAVNMVPFPR.L + Oxidation (M)
643	660.3592	1318.7038	1318.6955	6.32	0	63	3.3e-005	1	R.IMNTFSVVPSPK.V
653	668.3525	1334.6904	1334.6904	0.01	0	(8)	12	1	R.IMNTFSVVPSPK.V + Oxidation (M)
654	668.3532	1334.6918	1334.6904	1.06	0	(4)	28	4	R.IMNTFSVVPSPK.V + Oxidation (M)
788	540.9453	1619.8141	1619.8283	-8.76	0	(53)	0.00031	1	R.LHFFMPGFAPLTSR.G
789	810.9224	1619.8302	1619.8283	1.23	0	57	0.00013	1	R.LHFFMPGFAPLTSR.G
790	810.9226	1619.8306	1619.8283	1.47	0	(45)	0.0019	1	R.LHFFMPGFAPLTSR.G
☑ 816	846.4307	1690.8468	1690.8600	-7.78	0	43	0.0034	1	R.ALTVPELTQQMFDAK.N
819	848.9158	1695.8170	1695.8257	-5.07	0	64	2.6e-005	1	K.NSSYFVEWIPNNVK.T
820	848.9227	1695.8308	1695.8257	3.06	0	(47)	0.0013	1	K.NSSYFVEWIPNNVK.T
☑ 833	854.4404	1706.8662	1706.8549	6.64	0	(38)	0.0095	1	R.ALTVPELTQQMFDAK.N + Oxidation (M)
877	653.6544	1957.9414	1957.9745	-16.94	0	(41)	0.0039	1	K.GHYTEGAELVDSVLDVVR.K
878	980.0190	1958.0234	1957.9745	25.0	0	101	4.2e-009	1	K.GHYTEGAELVDSVLDVVR.K
936	933.4480	2797.3222	2797.3361	-4.99	0	(89)	4.3e-008	1	R.SGPFGQIFRPDNFVFGQSGAGNNWAK.G
937	933.4524	2797.3354	2797.3361	-0.27	0	99	5.2e-009	1	R.SGPFGQIFRPDNFVFGQSGAGNNWAK.G
938	1399.6794	2797.3442	2797.3361	2.91	0	(71)	2.9e-006	1	R.SGPFGQIFRPDNFVFGQSGAGNNWAK.G
939	933.4583	2797.3531	2797.3361	6.06	0	(34)	0.016	1	R.SGPFGQIFRPDNFVFGQSGAGNNWAK.G

---

8. IPI00124695 **Mass:** 60254 **Score:** 412 **Queries matched:** 18 **emPAI:** 0.79
Tax_Id=10090 Gene_Symbol=Camk2g Isoform 1 of Calcium/calmodulin-dependent protein kinase type II subunit gamma

☐ Check to include this hit in error tolerant search or archive report

Query	Observed	Mr(expt)	Mr(calc)	ppm	Miss	Score	Expect	Rank	Peptide
155	445.2853	444.2780	444.2696	18.9	0	5	23	1	K.GAAVK.L
424	446.2583	890.5020	890.5086	-7.40	1	8	12	2	K.GAFSVVRR.C
489	510.2411	1018.4676	1018.4688	-1.14	0	37	0.011	1	R.STVASMMHR.Q
495	518.2553	1034.4960	1034.4637	31.2	0	(28)	0.13	1	R.STVASMMHR.Q + Oxidation (M)
☑ 549	563.7897	1125.5648	1125.5666	-1.53	1	50	0.00076	1	K.KTSTQEYAAK.I
558	580.8033	1159.5920	1159.5913	0.61	0	2	56	6	K.FYFENLLSK.N
656	670.8677	1339.7208	1339.7711	-37.49	0	(77)	1.1e-006	1	R.DLKPENLLLASK.C
657	670.8842	1339.7538	1339.7711	-12.86	0	78	7.5e-007	1	R.DLKPENLLLASK.C
658	447.5925	1339.7557	1339.7711	-11.49	0	(59)	6e-005	1	R.DLKPENLLLASK.C
688	468.5813	1402.7221	1402.7317	-6.87	0	(26)	0.16	1	R.LTQYIDGQGRPR.T
689	468.5816	1402.7230	1402.7317	-6.23	0	(40)	0.0067	1	R.LTQYIDGQGRPR.T
690	702.3738	1402.7330	1402.7317	0.96	0	(37)	0.015	1	R.LTQYIDGQGRPR.T
691	702.3776	1402.7406	1402.7317	6.38	0	51	0.00048	1	R.LTQYIDGQGRPR.T
707	743.4136	1484.8126	1484.8021	7.13	0	75	1.7e-006	1	K.NLINQMLTINPAK.R + Oxidation (M)
☑ 782	802.8676	1603.7206	1603.7406	-12.44	0	90	3.8e-008	1	R.FTDDYQLFEELGK.G
794	542.6372	1624.8898	1624.9082	-11.37	1	89	5.7e-008	1	K.NLINQMLTINPAKR.I
795	813.4636	1624.9126	1624.9082	2.71	1	(55)	9.8e-005	1	K.NLINQMLTINPAKR.I
883	1040.9640	2079.9134	2079.9426	-13.99	0	78	5.2e-007	1	K.AGAYDFPSPEWDTVTPEAK.N

Proteins matching the same set of peptides:
IPI00228044 **Mass:** 59013 **Score:** 412 **Queries matched:** 18
Tax_Id=10090 Gene_Symbol=Camk2g Isoform 2 of Calcium/calmodulin-dependent protein kinase type II subunit gamma
IPI00228045 **Mass:** 56610 **Score:** 412 **Queries matched:** 18
Tax_Id=10090 Gene_Symbol=Camk2g Isoform 3 of Calcium/calmodulin-dependent protein kinase type II subunit gamma

---

9. IPI00117348 **Mass:** 50804 **Score:** 341 **Queries matched:** 12 **emPAI:** 0.46
Tax_Id=10090 Gene_Symbol=Tuba1b Tubulin alpha-1B chain

☐ Check to include this hit in error tolerant search or archive report

Query	Observed	Mr(expt)	Mr(calc)	ppm	Miss	Score	Expect	Rank	Peptide
☑ 708	744.4436	1486.8726	1486.8719	0.52	0	90	2.2e-008	1	R.LISQIVSSITASLR.F
☑ 822	851.4536	1700.8926	1700.8985	-3.45	0	64	2.3e-005	1	R.AVFVDLEPTVIDEVR.T
☑ 823	851.4573	1700.9000	1700.8985	0.90	0	(60)	5.7e-005	1	R.AVFVDLEPTVIDEVR.T

841	878.9860	1755.9574	1755.9559	0.86	0	67	8.3e-006	1	R.IHFPLATYAPVISAEK.A
842	878.9954	1755.9762	1755.9559	11.6	0	(66)	9.9e-006	1	R.IHFPLATYAPVISAEK.A
857	912.9819	1823.9492	1823.9782	-15.85	0	(26)	0.13	1	K.VGINYQPPTVVPGGDLAK.V
858	913.0020	1823.9894	1823.9782	6.19	0	29	0.054	1	K.VGINYQPPTVVPGGDLAK.V
914	1205.1045	2408.1944	2408.2012	-2.82	0	128	7.5e-012	1	R.FDGALNVDLTEFQTNLVPYPR.I
915	604.5469	2414.1585	2414.1978	-16.29	1	(39)	0.0058	1	R.QLFHPEQLITGKEDAANNYAR.G
916	604.5476	2414.1613	2414.1978	-15.13	1	54	0.0002	1	R.QLFHPEQLITGKEDAANNYAR.G
917	604.5506	2414.1733	2414.1978	-10.16	1	(24)	0.19	1	R.QLFHPEQLITGKEDAANNYAR.G
918	604.5528	2414.1821	2414.1978	-6.52	1	(18)	0.68	1	R.QLFHPEQLITGKEDAANNYAR.G

Proteins matching the same set of peptides:
IPI00129028 **Mass:** 50689 **Score:** 341 **Queries matched:** 12
Tax_Id=10090 Gene_Symbol=Gm5620 Uncharacterized protein
IPI00403810 **Mass:** 50562 **Score:** 341 **Queries matched:** 12
Tax_Id=10090 Gene_Symbol=Tuba1c Tubulin alpha-1C chain

---

10. IPI00230145 **Mass:** 21224 **Score:** 297 **Queries matched:** 10 **emPAI:** 1.09
Tax_Id=10090 Gene_Symbol=Fth1 Ferritin heavy chain
Check to include this hit in error tolerant search or archive report

Query	Observed	Mr(expt)	Mr(calc)	ppm	Miss	Score	Expect	Rank	Peptide
605	423.2317	1266.6733	1266.6932	-15.71	0	58	9.9e-005	1	K.SVNQSLLELHK.L
606	634.3522	1266.6898	1266.6932	-2.63	0	(57)	0.0001	1	K.SVNQSLLELHK.L
607	634.3550	1266.6954	1266.6932	1.79	0	(57)	9.5e-005	1	K.SVNQSLLELHK.L
718	765.3590	1528.7034	1528.7018	1.06	0	(64)	1.9e-005	1	R.QNYHQDAEAAINR.Q
719	765.3598	1528.7050	1528.7018	2.11	0	72	3e-006	1	R.QNYHQDAEAAINR.Q
920	641.5282	2562.0837	2562.1155	-12.42	1	(41)	0.0013	1	K.MGAPEAGMAEYLFDKHTLGHGDES.-
921	855.0369	2562.0889	2562.1155	-10.40	1	(26)	0.045	1	K.MGAPEAGMAEYLFDKHTLGHGDES.-
922	641.5305	2562.0929	2562.1155	-8.83	1	(13)	0.83	1	K.MGAPEAGMAEYLFDKHTLGHGDES.-
923	855.0422	2562.1048	2562.1155	-4.19	1	111	1.6e-010	1	K.MGAPEAGMAEYLFDKHTLGHGDES.-
924	855.0442	2562.1108	2562.1155	-1.85	1	(46)	0.00051	1	K.MGAPEAGMAEYLFDKHTLGHGDES.-

---

11. IPI00112251 **Mass:** 50842 **Score:** 277 **Queries matched:** 14 **emPAI:** 0.76
Tax_Id=10090 Gene_Symbol=Tubb3 Tubulin beta-3 chain
Check to include this hit in error tolerant search or archive report

Query	Observed	Mr(expt)	Mr(calc)	ppm	Miss	Score	Expect	Rank	Peptide
557	580.3218	1158.6290	1158.6219	6.13	0	48	0.0012	1	K.LAVNMVPFPR.L + Oxidation (M)
643	660.3592	1318.7038	1318.6955	6.32	0	63	3.3e-005	1	R.IMNTFSVVPSPK.V
653	668.3525	1334.6904	1334.6904	0.01	0	(8)	12	1	R.IMNTFSVVPSPK.V + Oxidation (M)
654	668.3532	1334.6918	1334.6904	1.06	0	(4)	28	4	R.IMNTFSVVPSPK.V + Oxidation (M)
783	808.4200	1614.8254	1614.8287	-2.02	0	(49)	0.00091	1	R.AILVDLEPGTMDSVR.S
789	810.9224	1619.8302	1619.8283	1.23	0	18	0.99	2	R.LHFFMPGFAPLTAR.G + Oxidation (M)
790	810.9226	1619.8306	1619.8283	1.47	0	(17)	1.3	2	R.LHFFMPGFAPLTAR.G + Oxidation (M)
797	816.4169	1630.8192	1630.8236	-2.68	0	59	0.0001	1	R.AILVDLEPGTMDSVR.S + Oxidation (M)
816	846.4307	1690.8468	1690.8600	-7.78	0	43	0.0034	1	R.ALTVPELTQQMFDAK.N
819	848.9158	1695.8170	1695.8257	-5.07	0	64	2.6e-005	1	K.NSSYFVEWIPNNVK.V
820	848.9227	1695.8308	1695.8257	3.06	0	(47)	0.0013	1	K.NSSYFVEWIPNNVK.V
833	854.4404	1706.8662	1706.8549	6.64	0	(38)	0.0095	1	R.ALTVPELTQQMFDAK.N + Oxidation (M)
877	653.6544	1957.9414	1957.9745	-16.94	0	(41)	0.0039	1	K.GHYTEGAELVDSVLDVVR.K
878	980.0190	1958.0234	1957.9745	25.0	0	101	4.2e-009	1	K.GHYTEGAELVDSVLDVVR.K

---

12. IPI00115546 **Mass:** 40524 **Score:** 212 **Queries matched:** 4 **emPAI:** 0.27
Tax_Id=10090 Gene_Symbol=Gnao1 Isoform Alpha-2 of Guanine nucleotide-binding protein G(o) subunit alpha
Check to include this hit in error tolerant search or archive report

Query	Observed	Mr(expt)	Mr(calc)	ppm	Miss	Score	Expect	Rank	Peptide
509	529.3168	1056.6190	1056.6179	1.10	0	45	0.0021	1	K.LLLLGAGESGK.S
811	838.4183	1674.8220	1674.8213	0.44	0	98	1.1e-008	1	R.IGAGDYQPTEQDILR.T
812	838.4184	1674.8222	1674.8213	0.56	0	(59)	8.4e-005	1	R.IGAGDYQPTEQDILR.T
876	979.4533	1956.8920	1956.8631	14.8	0	106	9.8e-010	1	R.MEDTEPFSAELLSAMMR.L

Proteins matching the same set of peptides:
IPI00230192 **Mass:** 40629 **Score:** 212 **Queries matched:** 4
Tax_Id=10090 Gene_Symbol=Gnao1 Isoform Alpha-1 of Guanine nucleotide-binding protein G(o) subunit alpha

---

13. IPI00138892 **Mass:** 15004 **Score:** 171 **Queries matched:** 3 **emPAI:** 0.51
Tax_Id=10090 Gene_Symbol=Uba52 Ubiquitin-60S ribosomal protein L40
Check to include this hit in error tolerant search or archive report

Query	Observed	Mr(expt)	Mr(calc)	ppm	Miss	Score	Expect	Rank	Peptide
712	762.3933	1522.7720	1522.7740	-1.25	1	(66)	1.8e-005	1	K.IQDKEGIPPDQQR.L
713	762.4007	1522.7868	1522.7740	8.47	1	83	3.2e-007	1	K.IQDKEGIPPDQQR.L
881	663.0222	1986.0448	1986.0521	-3.68	1	86	1.3e-007	1	K.TITLEVEPSDTIENVKAK.I

Proteins matching the same set of peptides:
IPI00470152 **Mass:** 18282 **Score:** 171 **Queries matched:** 3
Tax_Id=10090 Gene_Symbol=Rps27a Ubiquitin-40S ribosomal protein S27a
IPI00895319 **Mass:** 22277 **Score:** 171 **Queries matched:** 3
Tax_Id=10090 Gene_Symbol=2810422J05Rik 22 kDa protein
IPI00895479 **Mass:** 27181 **Score:** 171 **Queries matched:** 3
Tax_Id=10090 Gene_Symbol=2810422J05Rik 27 kDa protein

IPI00918763 **Mass:** 11021 **Score:** 171 **Queries matched:** 3
Tax_Id=10090 Gene_Symbol=Uba52 Ubiqutin subunit 1
IPI00750889 **Mass:** 22822 **Score:** 171 **Queries matched:** 3
Tax_Id=10090 Gene_Symbol=Ubc Ubc protein
IPI00923013 **Mass:** 26603 **Score:** 171 **Queries matched:** 3
Tax_Id=10090 Gene_Symbol=Ubb Ubiquitin B
IPI00923037 **Mass:** 22578 **Score:** 171 **Queries matched:** 3
Tax_Id=10090 Gene_Symbol=Ubc Putative uncharacterized protein
IPI00139518 **Mass:** 34348 **Score:** 171 **Queries matched:** 3
Tax_Id=10090 Gene_Symbol=Ubb Polyubiquitin-B
IPI00969323 **Mass:** 82603 **Score:** 171 **Queries matched:** 3
Tax_Id=10090 Gene_Symbol=Ubc Polyubiquitin-C
IPI00755916 **Mass:** 82614 **Score:** 171 **Queries matched:** 3
Tax_Id=10090 Gene_Symbol=Ubc ubiquitin

---

14. IPI00122048 **Mass:** 113045 **Score:** 130 **Queries matched:** 4 **emPAI:** 0.09
Tax_Id=10090 Gene_Symbol=Atp1a3 Sodium/potassium-transporting ATPase subunit alpha-3
☐ Check to include this hit in error tolerant search or archive report

	Query	Observed	Mr(expt)	Mr(calc)	ppm	Miss	Score	Expect	Rank	Peptide
☑	592	618.8615	1235.7084	1235.6986	7.96	0	40	0.0032	1	R.LNIPVSQVNPR.D
☑	859	915.4680	1828.9214	1828.9167	2.62	0	109	7.1e-010	1	K.GVGIISEGNETVEDIAAR.L
☑	944	956.1194	2865.3364	2865.3490	-4.42	1	42	0.0022	1	K.EQPLDEEMKEAFQNAYLELGGLGER.V
☑	945	956.1257	2865.3553	2865.3490	2.18	1	(31)	0.03	1	K.EQPLDEEMKEAFQNAYLELGGLGER.V

Proteins matching the same set of peptides:
IPI00752412 **Mass:** 117548 **Score:** 130 **Queries matched:** 4
Tax_Id=10090 Gene_Symbol=Atp1a3 ATPase, Na+/K+ transporting, alpha 3 polypeptide, isoform CRA_c

---

15. IPI00121038 **Mass:** 368613 **Score:** 123 **Queries matched:** 2 **emPAI:** 0.02
Tax_Id=10090 Gene_Symbol=Vcan Isoform V0 of Versican core protein
☐ Check to include this hit in error tolerant search or archive report

	Query	Observed	Mr(expt)	Mr(calc)	ppm	Miss	Score	Expect	Rank	Peptide
☑	639	657.8640	1313.7134	1313.7092	3.26	0	81	5e-007	1	R.LATVGELQAAWR.N
☑	694	709.3778	1416.7410	1416.7283	9.03	1	73	4.4e-006	1	K.METSPPVKGSLSGK.V

Proteins matching the same set of peptides:
IPI00230441 **Mass:** 264594 **Score:** 123 **Queries matched:** 2
Tax_Id=10090 Gene_Symbol=Vcan Isoform V1 of Versican core protein
IPI00469172 **Mass:** 76209 **Score:** 123 **Queries matched:** 2
Tax_Id=10090 Gene_Symbol=Vcan versican core protein isoform 4
IPI00469565 **Mass:** 180325 **Score:** 123 **Queries matched:** 2
Tax_Id=10090 Gene_Symbol=Vcan versican core protein isoform 3
IPI00473932 **Mass:** 41850 **Score:** 123 **Queries matched:** 2
Tax_Id=10090 Gene_Symbol=Vcan versican core protein isoform 5
IPI00656277 **Mass:** 41758 **Score:** 123 **Queries matched:** 2
Tax_Id=10090 Gene_Symbol=Vcan Versican
IPI00875672 **Mass:** 368720 **Score:** 123 **Queries matched:** 2
Tax_Id=10090 Gene_Symbol=Vcan versican core protein isoform 1

---

16. IPI00621468 **Mass:** 23411 **Score:** 114 **Queries matched:** 3 **emPAI:** 0.14
Tax_Id=10090 Gene_Symbol=Gm7866 similar to ubiquitin A-52 residue ribosomal protein fusion product 1
☐ Check to include this hit in error tolerant search or archive report

	Query	Observed	Mr(expt)	Mr(calc)	ppm	Miss	Score	Expect	Rank	Peptide
	712	762.3933	1522.7720	1522.7740	-1.25	1	(66)	1.8e-005	1	K.IQDKEGIPPDQQR.L
	713	762.4007	1522.7868	1522.7740	8.47	1	83	3.2e-007	1	K.IQDKEGIPPDQQR.L
☑	836	582.9590	1745.8552	1745.8402	8.59	0	7	13	1	K.QFTAALIMMTALMEK.I + 3 Oxidation (M)

---

17. IPI00110850 **Mass:** 42052 **Score:** 107 **Queries matched:** 6 **emPAI:** 0.16
Tax_Id=10090 Gene_Symbol=Actb Actin, cytoplasmic 1
☐ Check to include this hit in error tolerant search or archive report

	Query	Observed	Mr(expt)	Mr(calc)	ppm	Miss	Score	Expect	Rank	Peptide
☑	576	599.8588	1197.7030	1197.6982	4.03	0	24	0.13	1	R.AVFPSIVGRPR.H
	791	812.4296	1622.8446	1622.8338	6.70	1	3	30	3	R.LDLAGRDLTDYLMK.I
☑	849	895.9518	1789.8890	1789.8846	2.47	0	21	0.43	1	K.SYELPDGQVITIGNER.F
	874	652.0251	1953.0535	1953.0571	-1.86	0	5	12	2	R.VAPEEHPVLLTEAPLNPK.A
☑	897	1108.0496	2214.0846	2214.0627	9.92	0	71	3.8e-006	1	K.DLYANTVLSGGTTMYPGIADR.M
☑	952	796.6569	3182.5985	3182.6071	-2.69	0	67	5.2e-006	1	R.TTGIVMDSGDGVTHTVPIYEGYALPHAILR.L

Proteins matching the same set of peptides:
IPI00473320 **Mass:** 42115 **Score:** 107 **Queries matched:** 6
Tax_Id=10090 Gene_Symbol=Actb Putative uncharacterized protein
IPI00652436 **Mass:** 42053 **Score:** 107 **Queries matched:** 6
Tax_Id=10090 Gene_Symbol=Actg1 Putative uncharacterized protein
IPI00874482 **Mass:** 42108 **Score:** 107 **Queries matched:** 6
Tax_Id=10090 Gene_Symbol=Actg1 Actin, cytoplasmic 2

---

18. IPI00136929 **Mass:** 44029 **Score:** 107 **Queries matched:** 5 **emPAI:** 0.16
Tax_Id=10090 Gene_Symbol=Actg1 Gamma actin-like protein
☐ Check to include this hit in error tolerant search or archive report

Query	Observed	Mr(expt)	Mr(calc)	ppm	Miss	Score	Expect	Rank	Peptide
791	812.4296	1622.8446	1622.8338	6.70	1	3	30	3	R.LDLAGRDLTDYLMK.I
849	895.9518	1789.8890	1789.8846	2.47	0	21	0.43	1	K.SYELPDGQVITIGNER.F
874	652.0251	1953.0535	1953.0571	-1.86	0	5	12	2	R.VAPEEHPVLLTEAPLNPK.R
897	1108.0496	2214.0846	2214.0627	9.92	0	71	3.8e-006	1	K.DLYANTVLSGGTTMYPGLADR.M
952	796.6569	3182.5985	3182.6071	-2.69	0	67	5.2e-006	1	R.TTGIVMDSGDGVTHTVPIYEGYALPHAILR.L

---

19. IPI00123176 **Mass:** 36804 **Score:** 99 **Queries matched:** 3 **emPAI:** 0.19
Tax_Id=10090 Gene_Symbol=Gm6316 Glyceraldehyde-3-phosphate dehydrogenase
☐ Check to include this hit in error tolerant search or archive report

	Query	Observed	Mr(expt)	Mr(calc)	ppm	Miss	Score	Expect	Rank	Peptide
☑	678	**685.3767**	**1368.7388**	**1368.7361**	**2.01**	**0**	**51**	**0.00049**	**1**	**R.GAAQNIIPASTGAAK.A**
☑	679	**685.3803**	**1368.7460**	**1368.7361**	**7.27**	**0**	**(27)**	**0.1**	**1**	**R.GAAQNIIPASTGAAK.A**
☑	846	**890.4040**	**1778.7934**	**1778.7900**	**1.95**	**0**	**85**	**1.4e-007**	**1**	**K.LISWYDNEYGYSNR.M**

Proteins matching the same set of peptides:
IPI00135284 **Mass:** 36092 **Score:** 99 **Queries matched:** 3
Tax_Id=10090 Gene_Symbol=Gm16374 Glyceraldehyde-3-phosphate dehydrogenase
IPI00271869 **Mass:** 36293 **Score:** 99 **Queries matched:** 3
Tax_Id=10090 Gene_Symbol=- 36 kDa protein
IPI00273646 **Mass:** 36072 **Score:** 99 **Queries matched:** 3
Tax_Id=10090 Gene_Symbol=Gapdh;LOC100042025 Glyceraldehyde-3-phosphate dehydrogenase
IPI00622795 **Mass:** 36074 **Score:** 99 **Queries matched:** 3
Tax_Id=10090 Gene_Symbol=- 36 kDa protein
IPI00752289 **Mass:** 36308 **Score:** 99 **Queries matched:** 3
Tax_Id=10090 Gene_Symbol=Gm10290 Glyceraldehyde-3-phosphate dehydrogenase
IPI00848801 **Mass:** 36100 **Score:** 99 **Queries matched:** 3
Tax_Id=10090 Gene_Symbol=- 36 kDa protein
IPI00849045 **Mass:** 36072 **Score:** 99 **Queries matched:** 3
Tax_Id=10090 Gene_Symbol=- 36 kDa protein
IPI00850337 **Mass:** 44633 **Score:** 99 **Queries matched:** 3
Tax_Id=10090 Gene_Symbol=LOC100048117 similar to Glyceraldehyde-3-phosphate dehydrogenase (GAPDH) isoform 1
IPI00850377 **Mass:** 35975 **Score:** 99 **Queries matched:** 3
Tax_Id=10090 Gene_Symbol=- 36 kDa protein
IPI00850779 **Mass:** 36076 **Score:** 99 **Queries matched:** 3
Tax_Id=10090 Gene_Symbol=- 36 kDa protein
IPI00874682 **Mass:** 36038 **Score:** 99 **Queries matched:** 3
Tax_Id=10090 Gene_Symbol=- 36 kDa protein
IPI00874964 **Mass:** 36512 **Score:** 99 **Queries matched:** 3
Tax_Id=10090 Gene_Symbol=- 36 kDa protein

---

20. IPI00114642 **Mass:** 13928 **Score:** 97 **Queries matched:** 1 **emPAI:** 0.25
Tax_Id=10090 Gene_Symbol=Hist1h2bn;Hist1h2bl;Hist1h2bj;Hist1h2bf Histone H2B type 1-F/J/L
☐ Check to include this hit in error tolerant search or archive report

	Query	Observed	Mr(expt)	Mr(calc)	ppm	Miss	Score	Expect	Rank	Peptide
☑	835	**872.4168**	**1742.8190**	**1742.8120**	**4.05**	**0**	**97**	**1.2e-008**	**1**	**K.AMGIMNSFVNDIFER.I**

Proteins matching the same set of peptides:
IPI00124518 **Mass:** 13359 **Score:** 97 **Queries matched:** 1
Tax_Id=10090 Gene_Symbol=Hist2h2bb Hist2h2bb protein
IPI00134534 **Mass:** 14879 **Score:** 97 **Queries matched:** 1
Tax_Id=10090 Gene_Symbol=Gm11277;Gm13646 Histone H2B
IPI00187543 **Mass:** 13985 **Score:** 97 **Queries matched:** 1
Tax_Id=10090 Gene_Symbol=Hist2h2be Histone H2B type 2-E
IPI00227930 **Mass:** 13912 **Score:** 97 **Queries matched:** 1
Tax_Id=10090 Gene_Symbol=Hist1h2bh Histone H2B type 1-H
IPI00229539 **Mass:** 17248 **Score:** 97 **Queries matched:** 1
Tax_Id=10090 Gene_Symbol=Hist3h2bb-ps histone H2B type 3-B
IPI00265768 **Mass:** 13912 **Score:** 97 **Queries matched:** 1
Tax_Id=10090 Gene_Symbol=Hist1h2bk Histone H2B type 1-K
IPI00282266 **Mass:** 13898 **Score:** 97 **Queries matched:** 1
Tax_Id=10090 Gene_Symbol=Hist1h2bg;Hist1h2be;Gm9998;Hist1h2bc Histone H2B type 1-C/E/G
IPI00282269 **Mass:** 13928 **Score:** 97 **Queries matched:** 1
Tax_Id=10090 Gene_Symbol=Hist1h2bm Histone H2B type 1-M
IPI00348270 **Mass:** 13912 **Score:** 97 **Queries matched:** 1
Tax_Id=10090 Gene_Symbol=Hist2h2bb Histone H2B type 2-B
IPI00453744 **Mass:** 15968 **Score:** 97 **Queries matched:** 1
Tax_Id=10090 Gene_Symbol=Hist1h2bp 16 kDa protein
IPI00461514 **Mass:** 14987 **Score:** 97 **Queries matched:** 1
Tax_Id=10090 Gene_Symbol=Gm13646 Histone H2B
IPI00553774 **Mass:** 13986 **Score:** 97 **Queries matched:** 1
Tax_Id=10090 Gene_Symbol=Hist3h2ba Histone H2B type 3-A
IPI00554853 **Mass:** 13944 **Score:** 97 **Queries matched:** 1
Tax_Id=10090 Gene_Symbol=Hist1h2bb Histone H2B type 1-B
IPI00648991 **Mass:** 13984 **Score:** 97 **Queries matched:** 1
Tax_Id=10090 Gene_Symbol=Hist1h2bp Isoform 1 of Histone H2B type 1-P
IPI00761713 **Mass:** 15555 **Score:** 97 **Queries matched:** 1
Tax_Id=10090 Gene_Symbol=Hist1h2bp Isoform 2 of Histone H2B type 1-P
IPI00874492 **Mass:** 14746 **Score:** 97 **Queries matched:** 1
Tax_Id=10090 Gene_Symbol=Hist1h2bm 15 kDa protein
IPI00874654 **Mass:** 14787 **Score:** 97 **Queries matched:** 1
Tax_Id=10090 Gene_Symbol=Hist1h2bg;Hist1h2be;Gm9998;Hist1h2bc Histone H2B

IPI00874970 **Mass:** 14951 **Score:** 97 **Queries matched:** 1
Tax_Id=10090 Gene_Symbol=Hist1h2bn;Hist1h2bl;Hist1h2bj;Hist1h2bf Histone H2B
IPI00875277 **Mass:** 14242 **Score:** 97 **Queries matched:** 1
Tax_Id=10090 Gene_Symbol=Hist1h2bk 14 kDa protein
IPI00875523 **Mass:** 14900 **Score:** 97 **Queries matched:** 1
Tax_Id=10090 Gene_Symbol=Hist1h2bh Histone H2B
IPI00875901 **Mass:** 14806 **Score:** 97 **Queries matched:** 1
Tax_Id=10090 Gene_Symbol=Hist1h2bk Histone H2B
IPI00876167 **Mass:** 14126 **Score:** 97 **Queries matched:** 1
Tax_Id=10090 Gene_Symbol=Hist1h2bn;Hist1h2bl;Hist1h2bj;Hist1h2bf 14 kDa protein
IPI00876432 **Mass:** 14148 **Score:** 97 **Queries matched:** 1
Tax_Id=10090 Gene_Symbol=Hist1h2bn;Hist1h2bl;Hist1h2bj;Hist1h2bf Histone H2B
IPI00876549 **Mass:** 14902 **Score:** 97 **Queries matched:** 1
Tax_Id=10090 Gene_Symbol=Hist1h2bn;Hist1h2bl;Hist1h2bj;Hist1h2bf Histone H2B
IPI00876550 **Mass:** 14812 **Score:** 97 **Queries matched:** 1
Tax_Id=10090 Gene_Symbol=- 15 kDa protein

---

21. IPI00380227 **Mass:** 7720 **Score:** 76 **Queries matched:** 1 **emPAI:** 0.45
Tax_Id=10090 Gene_Symbol=Nrgn Neurogranin
☐ Check to include this hit in error tolerant search or archive report

	Query	Observed	Mr(expt)	Mr(calc)	ppm	Miss	Score	Expect	Rank	Peptide
☑	573	**596.8132**	**1191.6118**	**1191.6109**	**0.81**	**1**	**76**	**2.1e-006**	**1**	**R.KGPGPGGPGGAGGAR.G**

---

22. IPI00135106 **Mass:** 19584 **Score:** 71 **Queries matched:** 4 **emPAI:** 0.61
Tax_Id=10090 Gene_Symbol=Mobp Isoform 1 of Myelin-associated oligodendrocyte basic protein
☐ Check to include this hit in error tolerant search or archive report

	Query	Observed	Mr(expt)	Mr(calc)	ppm	Miss	Score	Expect	Rank	Peptide
	435	**449.7448**	**897.4750**	**897.4781**	**-3.35**	**1**	**3**	**33**	**7**	**R.GGSPTRAPR.F**
☑	477	**498.7868**	**995.5590**	**995.5586**	**0.46**	**0**	**46**	**0.0011**	**1**	**K.SPLMPAKPR.S**
☑	546	**557.8030**	**1113.5914**	**1113.5891**	**2.14**	**1**	**38**	**0.012**	**1**	**R.SSPLRGPGTSR.G**
☑	559	**580.8307**	**1159.6468**	**1159.6462**	**0.56**	**0**	**48**	**0.00095**	**1**	**K.HQPAASPVVVR.A**

---

23. IPI00221528 **Mass:** 42319 **Score:** 67 **Queries matched:** 4 **emPAI:** 0.08
Tax_Id=10090 Gene_Symbol=Actbl2 Beta-actin-like protein 2
☐ Check to include this hit in error tolerant search or archive report

	Query	Observed	Mr(expt)	Mr(calc)	ppm	Miss	Score	Expect	Rank	Peptide
	791	812.4296	1622.8446	1622.8338	6.70	1	3	30	3	R.LDLAGRDLTDYLMK.I
	849	895.9518	1789.8890	1789.8846	2.47	0	21	0.43	1	R.SYELPDGQVITIGNER.F
☑	874	652.0251	1953.0535	1953.0571	-1.86	0	6	10	1	R.VAPDEHPILLTEAPLNPK.I
	952	796.6569	3182.5985	3182.6071	-2.69	0	67	5.2e-006	1	R.TTGIVMDSGDGVTHTVPIYEGYALPHAILR.L

---

24. IPI00136703 **Mass:** 42971 **Score:** 58 **Queries matched:** 1 **emPAI:** 0.08
Tax_Id=10090 Gene_Symbol=Ckb Creatine kinase B-type
☐ Check to include this hit in error tolerant search or archive report

	Query	Observed	Mr(expt)	Mr(calc)	ppm	Miss	Score	Expect	Rank	Peptide
☑	807	**828.9211**	**1655.8276**	**1655.8188**	**5.31**	**0**	**58**	**9.4e-005**	**1**	**R.LEQGQAIDDLMPAQK.-**

---

25. IPI00404590 **Mass:** 19412 **Score:** 53 **Queries matched:** 2 **emPAI:** 0.38
Tax_Id=10090 Gene_Symbol=H1f0 Putative uncharacterized protein
☐ Check to include this hit in error tolerant search or archive report

	Query	Observed	Mr(expt)	Mr(calc)	ppm	Miss	Score	Expect	Rank	Peptide
☑	514	**530.7678**	**1059.5210**	**1059.5196**	**1.33**	**0**	**42**	**0.0063**	**1**	**K.VGENADSQIK.L**
☑	624	**651.3369**	**1300.6592**	**1300.6623**	**-2.32**	**0**	**48**	**0.0013**	**1**	**M.TENSTSAPAAKPK.R**

Proteins matching the same set of peptides:
IPI00467914 **Mass:** 20848 **Score:** 53 **Queries matched:** 2
Tax_Id=10090 Gene_Symbol=H1f0 Histone H1.0

---

26. IPI00131366 **Mass:** 60627 **Score:** 50 **Queries matched:** 1 **emPAI:** 0.05
Tax_Id=10090 Gene_Symbol=Krt6b Keratin, type II cytoskeletal 6B
☐ Check to include this hit in error tolerant search or archive report

	Query	Observed	Mr(expt)	Mr(calc)	ppm	Miss	Score	Expect	Rank	Peptide
☑	627	**651.8530**	**1301.6914**	**1301.7078**	**-12.58**	**0**	**50**	**0.00072**	**1**	**R.SLDLDSIIAEVK.A**

Proteins matching the same set of peptides:
IPI00131368 **Mass:** 59641 **Score:** 50 **Queries matched:** 1
Tax_Id=10090 Gene_Symbol=Krt6a Keratin, type II cytoskeletal 6A
IPI00139301 **Mass:** 61957 **Score:** 50 **Queries matched:** 1
Tax_Id=10090 Gene_Symbol=Krt5 Keratin, type II cytoskeletal 5
IPI00221797 **Mass:** 59932 **Score:** 50 **Queries matched:** 1
Tax_Id=10090 Gene_Symbol=Krt75 Keratin, type II cytoskeletal 75
IPI00330480 **Mass:** 35315 **Score:** 50 **Queries matched:** 1
Tax_Id=10090 Gene_Symbol=- 35 kDa protein
IPI00470126 **Mass:** 60553 **Score:** 50 **Queries matched:** 1
Tax_Id=10090 Gene_Symbol=Krt5 Uncharacterized protein
IPI00762364 **Mass:** 50641 **Score:** 50 **Queries matched:** 1

Tax_Id=10090 Gene_Symbol=Krt6a Uncharacterized protein
IPI00785403 **Mass:** 59832 **Score:** 50 **Queries matched:** 1
Tax_Id=10090 Gene_Symbol=Krt6b keratin, type II cytoskeletal 6B

---

27. IPI00348094 **Mass:** 51093 **Score:** 48 **Queries matched:** 2 **emPAI:** 0.06
Tax_Id=10090 Gene_Symbol=Tubb1 Tubulin, beta 1
☐ Check to include this hit in error tolerant search or archive report

Query	Observed	Mr(expt)	Mr(calc)	ppm	Miss	Score	Expect	Rank	Peptide
557	580.3218	1158.6290	1158.6219	6.13	0	48	0.0012	1	K.LAVNMVPFPR.L + Oxidation (M)
643	660.3592	1318.7038	1318.6955	6.33	0	11	6	2	R.ILNSFSVMPSPK.V

---

28. IPI00229525 **Score:** 45 **Queries matched:** 2
Tax_Id=10090 Gene_Symbol=Gnal Guanine nucleotide-binding protein G(olf) subunit alpha
☐ Check to include this hit in error tolerant search or archive report

Query	Observed	Mr(expt)	Mr(calc)	ppm	Miss	Score	Expect	Rank	Peptide
509	529.3168	1056.6190	1056.6179	1.10	0	45	0.0021	1	R.LLLLGAGESGK.S
571	**595.8328**	**1189.6510**	**1189.6165**	**29.0**	**1**	**0**	**76**	**6**	**R.MHLKQYELL.- + Oxidation (M)**

Proteins matching the same set of peptides:
IPI00272681 **Score:** 45 **Queries matched:** 2

---

29. IPI00118569 **Mass:** 44369 **Score:** 45 **Queries matched:** 1 **emPAI:** 0.07
Tax_Id=10090 Gene_Symbol=Gna13 Guanine nucleotide-binding protein subunit alpha-13
☐ Check to include this hit in error tolerant search or archive report

Query	Observed	Mr(expt)	Mr(calc)	ppm	Miss	Score	Expect	Rank	Peptide
509	529.3168	1056.6190	1056.6179	1.10	0	45	0.0021	1	K.ILLLGAGESGK.S

Proteins matching the same set of peptides:
IPI00230191 **Mass:** 44238 **Score:** 45 **Queries matched:** 1
Tax_Id=10090 Gene_Symbol=Gna12 Guanine nucleotide-binding protein subunit alpha-12
IPI00649388 **Mass:** 20095 **Score:** 45 **Queries matched:** 1
Tax_Id=10090 Gene_Symbol=Gna13 Putative uncharacterized protein

---

30. IPI00119067 **Mass:** 23731 **Score:** 44 **Queries matched:** 1 **emPAI:** 0.14
Tax_Id=10090 Gene_Symbol=Tppp Tubulin polymerization-promoting protein
☐ Check to include this hit in error tolerant search or archive report

	Query	Observed	Mr(expt)	Mr(calc)	ppm	Miss	Score	Expect	Rank	Peptide
☑	871	**948.9888**	**1895.9630**	**1895.9516**	**6.02**	**0**	**44**	**0.0026**	**1**	**R.TITFEQFQEALEELAK.K**

Peptide matches not assigned to protein hits: (no details means no match)

	Query	Observed	Mr(expt)	Mr(calc)	ppm	Miss	Score	Expect	Rank	Peptide
☑	684	**696.3746**	**1390.7346**	**1390.7245**	**7.29**	**0**	**28**	**0.094**	**1**	**LEGVVFPYQPSR**
☑	792	**812.4532**	**1622.8918**	**1622.8992**	**-4.50**	**0**	**28**	**0.068**	**1**	**LAGTQPLEVLEAVQR**
☑	685	**697.3685**	**1392.7224**	**1392.7038**	**13.4**	**0**	**25**	**0.22**	**1**	**ELGGEVFYVGPAR**
☑	960	**1259.2880**	**3774.8422**	**3774.8417**	**0.12**	**0**	**23**	**0.092**	**1**	**ADTPIEEFTPTPAFPALQYLESVDEGGVAWQAGLR**
☑	595	**622.7920**	**1243.5694**	**1243.5581**	**9.10**	**1**	**22**	**0.34**	**1**	**ESQAYYDGRR**
☑	663	**673.8612**	**1345.7078**	**1345.6925**	**11.4**	**1**	**22**	**0.54**	**1**	**AGGQGFKMALNPR**
☑	404	**421.7574**	**841.5002**	**841.4810**	**22.9**	**0**	**21**	**0.48**	**1**	**AVLTWPR**
☑	515	**530.7717**	**1059.5288**	**1059.5713**	**-40.04**	**0**	**17**	**2**	**1**	**AGSLAFPELR**
☑	304	**346.2065**	**690.3984**	**690.3912**	**10.5**	**1**	**17**	**1.4**	**1**	**KDVTTK**
☑	565	**587.8109**	**1173.6072**	**1173.6353**	**-23.93**	**0**	**17**	**2**	**1**	**NATITQALTNK**
☑	488	**508.7708**	**1015.5270**	**1015.5298**	**-2.76**	**0**	**16**	**2.6**	**1**	**QHTVTTTTK**
☑	551	**566.2957**	**1130.5768**	**1130.5720**	**4.27**	**0**	**16**	**2.2**	**1**	**TTHYGSLPQK**
☑	496	**346.2080**	**1035.6022**	**1035.5964**	**5.55**	**0**	**16**	**0.9**	**1**	**KPIPLEDPK**
☑	508	**529.2868**	**1056.5590**	**1056.5716**	**-11.89**	**1**	**15**	**2.4**	**1**	**KPDWDLKR**
☑	394	**411.2468**	**820.4790**	**820.4668**	**15.0**	**1**	**15**	**1.5**	**1**	**RQPAPPR**
☑	709	**746.3885**	**1490.7624**	**1490.7841**	**-14.54**	**0**	**14**	**2.9**	**1**	**VIEDGPEPRPNLR**
☑	604	**633.3046**	**1264.5946**	**1264.6425**	**-37.83**	**1**	**14**	**2.7**	**1**	**FQQEAPHPRR**
☑	424	446.2583	890.5020	890.5226	-23.04	1	14	3.3	1	GVQFVIKT
☑	475	**329.1741**	**984.5005**	**984.4923**	**8.26**	**1**	**14**	**2.8**	**1**	**CVHKQTGR**
☑	436	**449.7468**	**897.4790**	**897.4920**	**-14.39**	**0**	**14**	**3**	**1**	**KPEGKPDK**
☑	470	**487.3048**	**972.5950**	**972.5869**	**8.38**	**0**	**13**	**1.5**	**1**	**VPGHLPVVR**
☑	418	**441.2692**	**880.5238**	**880.5355**	**-13.24**	**0**	**13**	**1.1**	**1**	**RPTVRPR**
☑	539	**551.8070**	**1101.5994**	**1101.5455**	**49.0**	**1**	**13**	**4**	**1**	**KDSGYYTLR**
☑	507	**529.2727**	**1056.5308**	**1056.5676**	**-34.77**	**1**	**13**	**3.6**	**1**	**QLGDLERAR**
☑	634	**653.3388**	**1304.6630**	**1304.6143**	**37.4**	**1**	**13**	**4.3**	**1**	**LGADMEDLRNR + Oxidation (M)**
☑	641	**659.8417**	**1317.6688**	**1317.7115**	**-32.35**	**1**	**12**	**4.4**	**1**	**LLYMAIDGVAPR**
☑	723	**776.3270**	**1550.6394**	**1550.6759**	**-23.48**	**1**	**12**	**0.97**	**1**	**GYDLAFRFCSCR**
☑	358	**780.3290**	**779.3217**	**779.3054**	**20.9**	**0**	**12**	**2**	**1**	**ANECMR**
☑	615	**643.8413**	**1285.6680**	**1285.7064**	**-29.81**	**1**	**12**	**5**	**1**	**TIMKDEIAIPR**
☑	478	**499.2877**	**996.5608**	**996.5141**	**46.9**	**0**	**12**	**3**	**1**	**VPWGSAQPR**
☑	324	**743.4591**	**742.4518**	**742.4198**	**43.1**	**1**	**12**	**5.5**	**1**	**RGAAVGGR**
☑	582	**606.7893**	**1211.5640**	**1211.6007**	**-30.23**	**1**	**11**	**4.1**	**1**	**DSLAEATRGHR**
☑	455	**470.7701**	**939.5256**	**939.5548**	**-31.07**	**1**	**11**	**3.5**	**1**	**MRKPRPR**
☑	519	**531.2891**	**1060.5636**	**1060.5665**	**-2.70**	**0**	**11**	**6.6**	**1**	**YLLASNAPGR**
☑	491	**517.2959**	**1032.5772**	**1032.6001**	**-22.17**	**1**	**11**	**5.1**	**1**	**KVIAAMTVGK + Oxidation (M)**
☑	569	**595.8130**	**1189.6114**	**1189.6489**	**-31.45**	**1**	**11**	**8**	**1**	**KTNIIDSMLR**

683	458.9068	1373.6986	1373.7085	-7.24	1	10	7.5	1	VTAAAMAGNKSTPR
710	756.8234	1511.6322	1511.6939	-40.80	1	10	2.3	1	YYGTSMHERVNR
687	699.8989	1397.7832	1397.7263	40.8	1	10	4.7	1	ELKTDNLPNQAR
614	643.3510	1284.6874	1284.7150	-21.41	1	10	6.8	1	QKNSINIQAAAK
480	500.2594	998.5042	998.5509	-46.69	0	10	6.7	1	VQIQQAANK
435	449.7448	897.4750	897.4556	21.7	0	10	7.2	1	DAETPLPR
809	834.9677	1667.9208	1667.8970	14.3	1	10	4.4	1	QPFFPTHMGPIKLR
301	685.3705	684.3632	684.3667	-5.08	0	10	4.9	1	GAERPR
840	878.4757	1754.9368	1754.9567	-11.28	1	10	4.7	1	ELLDLVASHFNLKEK
594	622.7913	1243.5680	1243.5681	-0.00	1	10	5.4	1	KQGGSPDEPDSK
443	455.2521	908.4896	908.4790	11.8	0	10	6.8	1	ELVMFVR + Oxidation (M)
506	528.7111	1055.4076	1055.4310	-22.17	1	10	1	1	MGNGMCSRK + Oxidation (M)
517	530.8016	1059.5886	1059.5672	20.2	1	10	8.9	1	SERDTLLAR
400	417.2493	832.4840	832.4443	47.7	0	9	5.6	1	SLPVDFR
518	530.8044	1059.5942	1059.5672	25.5	1	9	8.5	1	SERDTLLAR
635	653.8335	1305.6524	1305.6751	-17.32	1	9	8.3	1	YEALKEPMVAR
479	499.7479	997.4812	997.4507	30.6	1	9	6.6	1	MAMAMRQK + 2 Oxidation (M)
273	320.1377	638.2608	638.2880	-42.53	0	9	2.7	1	SMVMR + Oxidation (M)
501	523.2865	1044.5584	1044.5101	46.3	1	9	12	1	KGAAGWEGGGR
584	607.3194	1212.6242	1212.6211	2.61	1	9	9.2	1	LSASPSPADRGR
668	678.3817	1354.7488	1354.7820	-24.48	0	9	6.7	1	IVQLLAGVADQTK
680	458.5831	1372.7275	1372.7184	6.58	1	9	9.5	1	QAQHGRRPGNPR
522	532.2796	1062.5446	1062.4917	49.9	0	9	13	1	AGEMAQWVR + Oxidation (M)
516	530.8014	1059.5882	1059.5672	19.8	1	8	11	1	SERDTLLAR
180	487.3051	486.2978	486.2914	13.2	0	8	19	1	AGAIR
498	520.2791	1038.5436	1038.5458	-2.05	0	8	9.6	1	AQASAPAQAPK
810	838.3979	1674.7812	1674.8246	-25.91	1	8	8.7	1	ALDGLQREAMEELGK + Oxidation (M)
201	518.2473	517.2400	517.2609	-40.29	0	8	9.8	1	AGGTGR
654	668.3532	1334.6918	1334.7050	-9.86	1	8	12	1	DKPLISRTMMK + Oxidation (M)
854	903.4784	1804.9422	1805.0121	-38.69	0	8	9.1	1	MAGLVFLGLALSSGATVAK
538	548.2862	1094.5578	1094.5253	29.8	0	8	11	1	LWVMDMIR + 2 Oxidation (M)
608	639.2949	1276.5752	1276.6380	-49.15	1	8	8	1	GIRTCLICER
426	446.2641	890.5136	890.5226	-10.02	1	8	10	1	GVQFVIKT
454	467.7639	933.5132	933.4702	46.1	0	7	13	1	LSNNMLAR + Oxidation (M)
406	423.2285	844.4424	844.4767	-40.51	0	7	22	1	IGTSLAQR
256	607.3195	606.3122	606.3047	12.4	0	7	15	1	MDVVK + Oxidation (M)
504	525.7818	1049.5490	1049.5943	-43.14	0	7	15	1	VLFAVLNMK + Oxidation (M)
682	687.8503	1373.6860	1373.6939	-5.73	0	7	15	1	EVYAHAGTTLQGK
537	545.2896	1088.5646	1088.5978	-30.49	1	7	17	1	KDFTVNLPR
442	452.7382	903.4618	903.4522	10.7	1	7	20	1	GAATADSRR
306	350.1603	698.3060	698.3096	-5.09	0	7	4.1	1	SAHGDGR
560	581.3129	1160.6112	1160.5859	21.8	0	7	19	1	QMPSTIAIER + Oxidation (M)
460	475.7495	949.4844	949.4406	46.2	0	7	18	1	GQEQWFR
567	595.3054	1188.5962	1188.6033	-5.95	1	7	18	1	QMQASRLSPR + Oxidation (M)
503	525.2869	1048.5592	1048.5084	48.5	1	7	17	1	MAGLARGDSR + Oxidation (M)
303	343.7281	685.4416	685.4235	26.5	1	7	12	1	GAKALAR
597	623.3267	1244.6388	1244.5819	45.7	0	7	19	1	QDMPKPSEAAR + Oxidation (M)
873	956.4583	1910.9020	1910.9964	-49.39	1	6	11	1	LFEDMGLFEAFKIPVR
572	596.3065	1190.5984	1190.5649	28.2	1	6	19	1	RCSMTGQPVR
664	675.3417	1348.6688	1348.6735	-3.47	0	6	20	1	GADAASPPPVAGSPR
461	476.2360	950.4574	950.4682	-11.33	0	6	18	1	QAPGAHGASR
743	785.8390	1569.6634	1569.6664	-1.90	1	6	5.9	1	WTEGTCKGSTQCR
593	622.7913	1243.5680	1243.5581	7.97	1	6	13	1	ESQAYYDGRR
293	675.3319	674.3246	674.3347	-14.99	0	6	25	1	SSAASPR
927	881.7323	2642.1751	2642.2348	-22.59	1	6	7.6	1	ENSHPHHHHQQPPPQPGPSGERR
453	463.2427	924.4708	924.4310	43.1	0	6	17	1	FLCACVR
667	677.8867	1353.7588	1353.7001	43.4	1	6	11	1	TESGGALRDLAHK
213	531.2889	530.2816	530.2635	34.2	0	6	44	1	MAGPR
640	659.3407	1316.6668	1316.6725	-4.26	1	6	24	1	LVKEAFQDDPR
666	676.3508	1350.6870	1350.6271	44.4	1	5	22	1	EAMSKALDPAMR + 2 Oxidation (M)
600	630.3074	1258.6002	1258.6153	-11.97	1	5	19	1	KEVVEEAENGR
329	379.7334	757.4522	757.4698	-23.12	1	5	32	1	KELLQK
558	580.8033	1159.5920	1159.6131	-18.19	1	5	27	1	MQLAELQRR + Oxidation (M)
619	645.8328	1289.6510	1289.7013	-38.97	1	5	24	1	AEGTVVMKLAQK + Oxidation (M)
652	667.3491	1332.6836	1332.6972	-10.20	0	5	24	1	LGGLPGPGPMANPR
302	343.2315	684.4484	684.4646	-23.64	1	5	14	1	KAVVIR
677	684.3544	1366.6942	1366.6551	28.6	0	5	22	1	EGMVTFSNTLPR + Oxidation (M)
555	577.2852	1152.5558	1152.5564	-0.45	0	5	22	1	EAFGFEQAVR
850	897.9155	1793.8164	1793.8941	-43.31	1	5	17	1	TERSNTLNTAIVNMSK + Oxidation (M)
441	452.2508	902.4870	902.4709	17.9	1	5	36	1	KSDPELSK
476	496.2224	990.4302	990.4744	-44.54	0	5	12	1	LGGNHSNHR
571	595.8328	1189.6510	1189.6819	-25.93	1	5	27	1	KLFVQDLATR
669	678.8935	1355.7724	1355.7846	-8.98	1	5	16	1	QDPKLLLMLAAK + Oxidation (M)
626	651.8315	1301.6484	1301.6463	1.66	0	4	28	1	KPTAQEDSADLK
934	916.4277	2746.2613	2746.2996	-13.94	1	4	12	1	QAPPQYTFIGELNSMGIWYRMGR + 2 Oxidation (M)
556	577.2904	1152.5662	1152.6147	-42.08	1	4	26	1	LTGRLMFMGK
636	655.3013	1308.5880	1308.5252	48.0	0	4	17	1	DINDQMDESSR
520	531.8290	1061.6434	1061.6386	4.60	1	4	7.9	1	SFLILWKR
898	1110.6010	2219.1874	2219.1950	-3.39	1	4	15	1	QLFLEPSQKLEGPPALPEAR
487	505.7451	1009.4756	1009.4829	-7.15	0	4	24	1	EATPGEPGPR
570	595.8189	1189.6232	1189.6012	18.5	1	4	40	1	KQDIEAVMEK

642	660.3415	1318.6684	1318.6526	12.0	0	4	32	1	LTGAIMHFGNMK
578	604.7988	1207.5830	1207.5615	17.8	1	4	33	1	KGETSAANMQR + Oxidation (M)
817	847.9592	1693.9038	1693.8423	36.3	0	4	23	1	LRPIYDYLDNGNNK
711	756.8477	1511.6808	1511.6939	-8.65	1	4	21	1	YYGTSMHERVNR
425	446.2607	890.5068	890.4875	21.7	1	3	33	1	FQRWVR
791	812.4296	1622.8446	1622.9144	-42.98	0	3	27	1	FSAELIQHIPLSLR
568	595.8109	1189.6072	1189.6489	-34.98	1	3	46	1	KTNIIDSMLR
305	697.3679	696.3606	696.3919	-44.86	0	3	22	1	LPSPQR
784	809.3986	1616.7826	1616.8279	-27.99	1	3	32	1	KMGNKPHMIQVYR + Oxidation (M)
382	810.4398	809.4325	809.3953	46.0	0	3	24	1	MSLTNTK + Oxidation (M)
271	631.3865	630.3792	630.3813	-3.27	1	3	61	1	KQISR
274	320.1440	638.2734	638.3024	-45.33	0	3	15	1	HLDPAS
947	961.7438	2882.2096	2882.3141	-36.26	0	3	5.7	1	DPGSPQLNQEAMADGVEGTPWSAEKPR + Oxidation (M)
879	980.4991	1958.9836	1958.9810	1.36	0	3	32	1	SNNGTDVNAGQIPQLLYR
486	504.2251	1006.4356	1006.4693	-33.42	0	3	18	1	QSHSQQHR
270	631.3174	630.3101	630.3198	-15.29	1	3	43	1	QDGRR
536	545.2885	1088.5624	1088.6012	-35.59	1	3	51	1	KALTGSMPIR + Oxidation (M)
620	647.3015	1292.5884	1292.6183	-23.12	0	3	30	1	WTVVTPMSSNR + Oxidation (M)
292	672.3444	671.3371	671.3602	-34.42	0	2	24	1	LGETPR
773	785.8511	1569.6876	1569.7068	-12.20	1	2	20	1	KFPEWMSMQSQR + Oxidation (M)
777	795.4383	1588.8620	1588.9044	-26.68	1	2	31	1	LLKAGVISALACMVK + Oxidation (M)
577	600.2880	1198.5614	1198.5441	14.5	0	2	35	1	MGFPEAASSFR
943	1414.1135	2826.2124	2826.2840	-25.32	0	2	12	1	EYTESMCLLENELEAQLGEFHLR + Oxidation (M)
381	809.4304	808.4231	808.4443	-26.18	1	1	46	1	AVGKYSGK
629	652.2864	1302.5582	1302.6099	-39.65	1	1	20	1	RCADVPGASGTGR
245	600.3077	599.3004	599.3027	-3.83	0	1	21	1	DNPVR
250	601.3277	600.3204	600.3231	-4.49	0	1	36	1	ETVPR
545	556.8411	1111.6676	1111.6462	19.3	1	1	21	1	KPQLDTRVR
942	1400.1620	2798.3094	2798.3075	0.68	1	1	27	1	LGTNMYSASRPVETHMSATSKNAASR + 2 Oxidation (M)
903	762.0558	2283.1456	2283.0334	49.1	1	1	36	1	KCSCVICSPTYVPEDLEAR
421	445.2847	888.5548	888.5280	30.2	1	1	16	1	KDSSLIVK
887	1048.9823	2095.9500	2095.9469	1.52	0	1	33	1	SNPFHYPSNSVFFPFYF
715	510.2402	1527.6988	1527.6922	4.29	1	1	42	1	CNDPVMNEKLHR + Oxidation (M)
896	738.0253	2211.0541	2211.0477	2.86	0	0	45	1	GPELMAIEGLENGEGPTTAGPR + Oxidation (M)
941	933.7730	2798.2972	2798.3075	-3.71	1	0	32	1	LGTNMYSASRPVETHMSATSKNAASR + 2 Oxidation (M)
1	300.0226	299.0153							
2	301.1499	300.1426							
3	301.1562	300.1489							
4	301.1611	300.1538							
5	301.1714	300.1641							
6	305.9528	304.9455							
7	307.9514	306.9441							
8	308.0271	307.0198							
9	309.0632	308.0559							
10	309.0973	308.0900							
11	311.1737	310.1664							
12	312.9832	311.9759							
13	313.0961	312.0888							
14	315.0642	314.0569							
15	315.0667	314.0594							
16	315.0670	314.0597							
17	315.0680	314.0607							
18	315.0689	314.0616							
19	315.2316	314.2243							
20	315.2350	314.2277							
21	317.0646	316.0573							
22	322.0896	321.0823							
23	322.9079	321.9006							
24	322.9219	321.9146							
25	324.0315	323.0242							
26	325.0964	324.0891							
27	332.9781	331.9708							
28	333.0402	332.0329							
29	333.6491	332.6418							
30	338.8961	337.8888							
31	340.0163	339.0090							
32	340.8851	339.8778							
33	341.0215	340.0142							
34	341.0225	340.0152							
35	341.0247	340.0174							
36	341.0261	340.0188							
37	341.0267	340.0194							
38	341.0269	340.0196							
39	341.0484	340.0411							
40	341.0866	340.0793							
41	342.0295	341.0222							
42	342.0844	341.0771							
43	342.0868	341.0795							
44	342.0894	341.0821							
45	342.0905	341.0832							
46	342.0916	341.0843							

47	342.9606	341.9533
48	343.0220	342.0147
49	343.7295	342.7222
50	344.2295	343.2222
51	344.9496	343.9423
52	345.0750	344.0677
53	345.2236	344.2163
54	346.9440	345.9367
55	354.9622	353.9549
56	354.9651	353.9578
57	355.0676	354.0603
58	355.0696	354.0623
59	355.0723	354.0650
60	355.0738	354.0665
61	355.0746	354.0673
62	355.0752	354.0679
63	355.0760	354.0687
64	355.0764	354.0691
65	355.0774	354.0701
66	355.0786	354.0713
67	355.0806	354.0733
68	355.0832	354.0759
69	356.0023	354.9950
70	356.0729	355.0656
71	356.0748	355.0675
72	356.0748	355.0675
73	356.0791	355.0718
74	356.9736	355.9663
75	357.0002	355.9929
76	357.0729	356.0656
77	359.0438	358.0365
78	365.9749	364.9676
79	366.1819	365.1746
80	371.2194	370.2121
81	374.2417	373.2344
82	375.0931	374.0858
83	375.0943	374.0870
84	375.0979	374.0906
85	375.0984	374.0911
86	375.0995	374.0922
87	376.9003	375.8930
88	377.0886	376.0813
89	377.0909	376.0836
90	377.1556	376.1483
91	378.2327	377.2254
92	378.9160	377.9087
93	385.8234	384.8161
94	402.1234	401.1161
95	404.2323	403.2250
96	414.8633	413.8560
97	414.8758	413.8685
98	414.9092	413.9019
99	415.0326	414.0253
100	415.0343	414.0270
101	415.0360	414.0287
102	415.0377	414.0304
103	415.0380	414.0307
104	415.0395	414.0322
105	415.0411	414.0338
106	415.0453	414.0380
107	415.0509	414.0436
108	415.0523	414.0450
109	415.0569	414.0496
110	415.0619	414.0546
111	415.8512	414.8439
112	415.8564	414.8491
113	415.8646	414.8573
114	415.8731	414.8658
115	416.0414	415.0341
116	416.0461	415.0388
117	417.0393	416.0320
118	417.2494	416.2421
119	417.8458	416.8385
120	417.8559	416.8486
121	417.8654	416.8581
122	418.1017	417.0944
123	418.9891	417.9818
124	425.9435	424.9362
125	426.0362	425.0289
126	429.0944	428.0871
127	429.0957	428.0884

☑	128	429.0975	428.0902
☑	129	430.0917	429.0844
☑	130	430.0920	429.0847
☑	131	430.0957	429.0884
☑	132	430.0992	429.0919
☑	133	431.0923	430.0850
☑	134	431.0932	430.0859
☑	135	431.0937	430.0864
☑	136	431.0951	430.0878
☑	137	431.0982	430.0909
☑	138	432.0933	431.0860
☑	139	432.0939	431.0866
☑	140	432.0958	431.0885
☑	141	432.0974	431.0901
☑	142	433.0854	432.0781
☑	143	433.0930	432.0857
☑	144	445.1251	444.1178
☑	145	445.1266	444.1193
☑	146	445.1275	444.1202
☑	147	445.1280	444.1207
☑	148	445.1287	444.1214
☑	149	445.1289	444.1216
☑	150	445.1302	444.1229
☑	151	445.1305	444.1232
☑	152	445.1331	444.1258
☑	153	445.1368	444.1295
☑	154	445.2821	444.2748
☑	156	445.8061	444.7988
☑	157	446.1269	445.1196
☑	158	446.1286	445.1213
☑	159	446.1309	445.1236
☑	160	446.1360	445.1287
☑	161	446.1412	445.1339
☑	162	446.2843	445.2770
☑	163	446.2873	445.2800
☑	164	446.2876	445.2803
☑	165	446.2899	445.2826
☑	166	446.2906	445.2833
☑	167	447.0993	446.0920
☑	168	447.1175	446.1102
☑	169	447.1263	446.1190
☑	170	447.1268	446.1195
☑	171	447.1297	446.1224
☑	172	447.1343	446.1270
☑	173	447.1383	446.1310
☑	174	448.1143	447.1070
☑	175	449.7466	448.7393
☑	176	450.2478	449.2405
☑	177	461.2213	460.2140
☑	178	461.7260	460.7187
☑	179	472.2944	471.2871
☑	181	500.5695	499.5622
☑	182	500.5753	499.5680
☑	183	500.5760	499.5687
☑	184	500.9095	499.9022
☑	185	500.9131	499.9058
☑	186	500.9177	499.9104
☑	187	501.2471	500.2398
☑	188	501.2475	500.2402
☑	189	503.1157	502.1084
☑	190	503.1174	502.1101
☑	191	503.1176	502.1103
☑	192	503.1184	502.1111
☑	193	503.1201	502.1128
☑	194	503.1308	502.1235
☑	195	504.1160	503.1087
☑	196	504.1167	503.1094
☑	197	505.1104	504.1031
☑	198	505.1158	504.1085
☑	199	505.1231	504.1158
☑	200	506.1123	505.1050
☑	202	519.1440	518.1367
☑	203	519.1457	518.1384
☑	204	519.1489	518.1416
☑	205	519.1493	518.1420
☑	206	519.3159	518.3086
☑	207	519.3237	518.3164
☑	208	521.1315	520.1242
☑	209	521.1358	520.1285
☑	210	525.7811	524.7738
☑	211	528.3071	527.2998

☑	212	529.7824	528.7751
☑	214	536.1706	535.1633
☑	215	536.1713	535.1640
☑	216	536.1730	535.1657
☑	217	536.1737	535.1664
☑	218	536.1744	535.1671
☑	219	536.1745	535.1672
☑	220	536.1812	535.1739
☑	221	536.8585	535.8512
☑	222	536.8828	535.8755
☑	223	537.1801	536.1728
☑	224	538.1652	537.1579
☑	225	538.1685	537.1612
☑	226	538.7639	537.7566
☑	227	539.1697	538.1624
☑	228	540.8604	539.8531
☑	229	540.8785	539.8712
☑	230	553.0611	552.0538
☑	231	556.0690	555.0617
☑	232	580.8301	579.8228
☑	233	581.3103	580.3030
☑	234	581.7997	580.7924
☑	235	582.2520	581.2447
☑	236	582.9348	581.9275
☑	237	588.3419	587.3346
☑	238	597.8699	596.8626
☑	239	598.2394	597.2321
☑	240	598.2418	597.2345
☑	241	598.2443	597.2370
☑	242	300.0219	598.0292
☑	243	599.2518	598.2445
☑	244	600.3033	599.2960
☑	246	600.7967	599.7894
☑	247	600.8087	599.8014
☑	248	301.0960	600.1774
☑	249	301.1556	600.2966
☑	251	301.1781	600.3416
☑	252	301.1829	600.3512
☑	253	605.0685	604.0612
☑	254	304.0132	606.0118
☑	255	607.0647	606.0574
☑	257	607.8162	606.8089
☑	258	610.1964	609.1891
☑	259	610.2040	609.1967
☑	260	305.9519	609.8892
☑	261	307.9505	613.8864
☑	262	308.0223	614.0300
☑	263	311.6292	621.2438
☑	264	312.9918	623.9690
☑	265	313.1710	624.3274
☑	266	315.0595	628.1044
☑	267	315.0653	628.1160
☑	268	315.0656	628.1166
☑	269	315.0695	628.1244
☑	272	631.8058	630.7985
☑	275	321.0964	640.1782
☑	276	322.0926	642.1706
☑	277	645.7817	644.7744
☑	278	647.1479	646.1406
☑	279	647.2982	646.2909
☑	280	648.3733	647.3660
☑	281	325.1739	648.3332
☑	282	651.8560	650.8487
☑	283	653.6557	652.6484
☑	284	653.9991	652.9918
☑	285	654.3298	653.3225
☑	286	655.8140	654.8067
☑	287	329.1692	656.3238
☑	288	333.0366	664.0586
☑	289	333.0381	664.0616
☑	290	671.8058	670.7985
☑	291	672.2556	671.2483
☑	294	676.8664	675.8591
☑	295	341.0230	680.0314
☑	296	341.0260	680.0374
☑	297	341.6445	681.2744
☑	298	342.0403	682.0660
☑	299	342.0908	682.1670
☑	300	343.0520	684.0894
☑	307	355.0086	708.0026
☑	308	355.0699	708.1252

☑	309	355.0714	708.1282
☑	310	355.0716	708.1286
☑	311	355.0768	708.1390
☑	312	357.0765	712.1384
☑	313	357.0783	712.1420
☑	314	357.0813	712.1480
☑	315	715.0626	714.0553
☑	316	717.2094	716.2021
☑	317	719.2115	718.2042
☑	318	363.9105	725.8064
☑	319	365.9737	729.9328
☑	320	367.9813	733.9480
☑	321	735.2515	734.2442
☑	322	735.6586	734.6513
☑	323	371.2217	740.4288
☑	325	743.7808	742.7735
☑	326	377.0889	752.1632
☑	327	377.1041	752.1936
☑	328	378.7470	755.4794
☑	330	380.1723	758.3300
☑	331	764.6671	763.6598
☑	332	766.6816	765.6743
☑	333	768.4365	767.4292
☑	334	768.6876	767.6803
☑	335	768.9266	767.9193
☑	336	769.0150	768.0077
☑	337	776.5955	775.5882
☑	338	776.6066	775.5993
☑	339	776.7003	775.6930
☑	340	776.8279	775.8206
☑	341	776.8330	775.8257
☑	342	776.8347	775.8274
☑	343	776.8354	775.8281
☑	344	776.8408	775.8335
☑	345	776.8497	775.8424
☑	346	776.8613	775.8540
☑	347	777.0920	776.0847
☑	348	777.0993	776.0920
☑	349	777.3115	776.3042
☑	350	777.3357	776.3284
☑	351	777.3363	776.3290
☑	352	777.3436	776.3363
☑	353	777.3442	776.3369
☑	354	778.4701	777.4628
☑	355	778.7656	777.7583
☑	356	779.8745	778.8672
☑	357	779.8841	778.8768
☑	359	391.8338	781.6530
☑	360	783.7374	782.7301
☑	361	784.0562	783.0489
☑	362	784.3928	783.3855
☑	363	785.8331	784.8258
☑	364	785.8378	784.8305
☑	365	785.8421	784.8348
☑	366	785.8471	784.8398
☑	367	786.3374	785.3301
☑	368	786.3397	785.3324
☑	369	786.3427	785.3354
☑	370	786.3428	785.3355
☑	371	786.3466	785.3393
☑	372	786.8060	785.7987
☑	373	786.8641	785.8568
☑	374	787.3468	786.3395
☑	375	787.3529	786.3456
☑	376	788.7139	787.7066
☑	377	798.4542	797.4469
☑	378	798.4572	797.4499
☑	379	798.8981	797.8908
☑	380	801.2337	800.2264
☑	383	812.9364	811.9291
☑	384	813.4753	812.4680
☑	385	815.6945	814.6872
☑	386	816.2376	815.2303
☑	387	816.8932	815.8859
☑	388	817.4152	816.4079
☑	389	817.4265	816.4192
☑	390	818.4018	817.3945
☑	391	819.3159	818.3086
☑	392	820.6399	819.6326
☑	393	821.1201	820.1128
☑	395	823.3724	822.3651

396	823.7456	822.7383
397	414.8913	827.7680
398	415.0452	828.0758
399	830.2775	829.2702
401	837.4243	836.4170
402	838.5903	837.5830
403	841.7367	840.7294
405	845.1757	844.1684
407	849.6374	848.6301
408	849.7866	848.7793
409	851.4526	850.4453
410	857.9733	856.9660
411	860.7335	859.7262
412	431.0923	860.1700
413	862.0374	861.0301
415	870.7036	869.6963
416	880.8088	879.8015
417	881.1193	880.1120
419	888.5085	887.5012
420	445.1362	888.2578
422	446.1226	890.2306
423	446.1356	890.2566
427	446.2776	890.5406
428	446.2813	890.5480
429	446.2857	890.5568
430	446.2881	890.5616
431	447.1323	892.2500
432	896.6285	895.6212
433	897.0573	896.0500
434	898.1887	897.1814
437	899.5178	898.5105
438	899.6995	898.6922
439	900.0301	899.0228
440	902.5184	901.5111
444	913.8610	912.8537
445	915.2546	914.2473
446	915.5565	914.5492
447	915.7029	914.6956
448	915.7895	914.7822
449	919.6974	918.6901
450	920.1298	919.1225
451	922.8311	921.8238
452	924.2578	923.2505
456	941.0922	940.0849
457	315.0633	942.1681
458	317.0587	948.1543
459	475.2968	948.5790
462	957.1169	956.1096
463	962.7478	961.7405
464	963.0892	962.0819
465	967.9174	966.9101
466	968.1363	967.1290
467	968.4897	967.4824
468	969.1295	968.1222
469	970.4609	969.4536
471	980.0015	978.9942
472	980.0056	978.9983
473	980.4962	979.4889
474	980.5060	979.4987
481	500.5687	999.1228
482	500.5759	999.1372
483	501.2654	1000.5162
484	1004.0667	1003.0594
485	504.1158	1006.2170
490	343.6946	1028.0620
492	1034.7859	1033.7786
493	1035.1492	1034.1419
494	1035.4902	1034.4829
497	519.1487	1036.2828
499	1039.7976	1038.7903
500	1040.5476	1039.5403
505	528.3087	1054.6028
521	355.0849	1062.2329
523	536.1694	1070.3242
524	536.1718	1070.3290
525	537.1768	1072.3390
526	537.1837	1072.3528
527	538.1855	1074.3564
528	538.1870	1074.3594
532	1084.8878	1083.8805
533	1085.2523	1084.2450

☑	534	1086.2480	1085.2407
☑	535	1086.8662	1085.8589
☑	540	553.5438	1105.0730
☑	541	554.0579	1106.1012
☑	542	554.5394	1107.0642
☑	543	555.0557	1108.0968
☑	544	556.5324	1111.0502
☑	550	1130.9424	1129.9351
☑	561	582.2452	1162.4758
☑	562	582.2461	1162.4776
☑	566	588.3422	1174.6698
☑	574	598.2422	1194.4698
☑	575	598.2469	1194.4792
☑	579	605.0485	1208.0824
☑	580	605.5365	1209.0584
☑	581	606.0610	1210.1074
☑	583	607.0651	1212.1156
☑	585	608.0646	1214.1146
☑	586	608.5372	1215.0598
☑	587	609.5399	1217.0652
☑	588	1218.7135	1217.7062
☑	589	610.2018	1218.3890
☑	590	610.5284	1219.0422
☑	591	611.5313	1221.0480
☑	598	624.0483	1246.0820
☑	599	627.3233	1252.6320
☑	609	641.3354	1280.6562
☑	610	641.5300	1281.0454
☑	611	641.5305	1281.0464
☑	612	641.7771	1281.5396
☑	613	641.7777	1281.5408
☑	616	644.2595	1286.5044
☑	617	645.5241	1289.0336
☑	618	645.7799	1289.5452
☑	621	648.3745	1294.7344
☑	622	649.5335	1297.0524
☑	623	649.7742	1297.5338
☑	625	1302.0707	1301.0634
☑	628	1303.1967	1302.1894
☑	637	655.8581	1309.7016
☑	638	1313.2651	1312.2578
☑	644	1319.8895	1318.8822
☑	645	1320.5833	1319.5760
☑	646	1321.4066	1320.3993
☑	647	1322.7480	1321.7407
☑	648	1322.9954	1321.9881
☑	649	1324.2865	1323.2792
☑	650	664.3196	1326.6246
☑	651	665.3572	1328.6998
☑	655	1340.6353	1339.6280
☑	659	1343.6578	1342.6505
☑	660	672.3425	1342.6704
☑	661	1343.8400	1342.8327
☑	662	672.8325	1343.6504
☑	665	675.8490	1349.6834
☑	670	1357.9689	1356.9616
☑	671	1358.2532	1357.2459
☑	672	1358.6161	1357.6088
☑	673	1363.3037	1362.2964
☑	674	1363.5789	1362.5716
☑	675	1363.9362	1362.9289
☑	676	1364.2750	1363.2677
☑	686	699.8782	1397.7418
☑	692	708.4047	1414.7948
☑	693	708.4058	1414.7970
☑	695	715.0639	1428.1132
☑	696	715.5601	1429.1056
☑	697	716.0361	1430.0576
☑	698	716.5448	1431.0750
☑	699	717.0455	1432.0764
☑	700	717.5436	1433.0726
☑	701	718.0258	1434.0370
☑	702	718.3358	1434.6570
☑	703	718.5233	1435.0320
☑	704	736.7929	1471.5712
☑	714	1527.5295	1526.5222
☑	716	1528.9178	1527.9105
☑	717	1529.1819	1528.1746
☑	720	768.4321	1534.8496
☑	721	768.7252	1535.4358
☑	722	770.2192	1538.4238

☑	724	776.5931	1551.1716
☑	725	776.8279	1551.6412
☑	726	776.8310	1551.6474
☑	727	776.8319	1551.6492
☑	728	776.8325	1551.6504
☑	729	776.8341	1551.6536
☑	730	776.8350	1551.6554
☑	731	776.8358	1551.6570
☑	732	776.8389	1551.6632
☑	733	776.8391	1551.6636
☑	734	776.8400	1551.6654
☑	735	776.8446	1551.6746
☑	736	780.0881	1558.1616
☑	737	780.0950	1558.1754
☑	738	780.0955	1558.1764
☑	739	780.0988	1558.1830
☑	740	780.3696	1558.7246
☑	741	785.6671	1569.3196
☑	742	785.8365	1569.6584
☑	744	785.8393	1569.6640
☑	745	785.8394	1569.6642
☑	746	785.8405	1569.6664
☑	747	785.8409	1569.6672
☑	748	785.8411	1569.6676
☑	749	785.8415	1569.6684
☑	750	785.8415	1569.6684
☑	751	785.8418	1569.6690
☑	752	785.8422	1569.6698
☑	753	785.8427	1569.6708
☑	754	785.8434	1569.6722
☑	755	785.8435	1569.6724
☑	756	785.8438	1569.6730
☑	757	785.8438	1569.6730
☑	758	785.8439	1569.6732
☑	759	785.8442	1569.6738
☑	760	785.8443	1569.6740
☑	761	785.8443	1569.6740
☑	762	785.8445	1569.6744
☑	763	785.8446	1569.6746
☑	764	785.8452	1569.6758
☑	765	785.8453	1569.6760
☑	766	785.8463	1569.6780
☑	767	785.8466	1569.6786
☑	768	785.8469	1569.6792
☑	769	785.8472	1569.6798
☑	770	785.8482	1569.6818
☑	771	785.8499	1569.6852
☑	772	785.8504	1569.6862
☑	774	785.8514	1569.6882
☑	775	787.3445	1572.6744
☑	776	791.1782	1580.3418
☑	778	798.4543	1594.8940
☑	779	798.4565	1594.8984
☑	780	800.2484	1598.4822
☑	781	802.2179	1602.4212
☑	793	812.9390	1623.8634
☑	796	814.1793	1626.3440
☑	798	816.6277	1631.2408
☑	803	816.9065	1631.7984
☑	804	820.6395	1639.2644
☑	805	820.6462	1639.2778
☑	806	820.8856	1639.7566
☑	813	840.0740	1678.1334
☑	814	842.7434	1683.4722
☑	815	844.2412	1686.4678
☑	818	848.4417	1694.8688
☑	821	850.9721	1699.9296
☑	824	851.4576	1700.9006
☑	825	851.4578	1700.9010
☑	826	851.4581	1700.9016
☑	827	851.4586	1700.9026
☑	828	852.9587	1703.9028
☑	834	861.8549	1721.6952
☑	837	874.0439	1746.0732
☑	838	874.5388	1747.0630
☑	839	878.0348	1754.0550
☑	843	885.9266	1769.8386
☑	844	889.1068	1776.1990
☑	845	889.7362	1777.4578
☑	847	595.3176	1782.9310
☑	848	895.6211	1789.2276

☑	851	897.9429	1793.8712
☑	852	899.5272	1797.0398
☑	853	902.4464	1802.8782
☑	855	1809.2770	1808.2697
☑	856	1810.1915	1809.1842
☑	860	921.1354	1840.2562
☑	861	1844.5225	1843.5152
☑	862	924.6959	1847.3772
☑	863	1850.4302	1849.4229
☑	864	928.0735	1854.1324
☑	865	934.9470	1867.8794
☑	866	1875.9568	1874.9495
☑	867	1881.9951	1880.9878
☑	868	1883.1820	1882.1747
☑	869	942.1296	1882.2446
☑	870	1886.2216	1885.2143
☑	872	956.1188	1910.2230
☑	875	978.5511	1955.0876
☑	880	1962.2844	1961.2771
☑	882	1039.9951	2077.9756
☑	884	1048.4742	2094.9338
☑	885	1048.9790	2095.9434
☑	886	1048.9817	2095.9488
☑	888	699.7144	2096.1214
☑	889	1056.4691	2110.9236
☑	892	1064.9711	2127.9276
☑	893	1072.9751	2143.9356
☑	894	1072.9877	2143.9608
☑	895	737.0175	2208.0307
☑	899	1111.1013	2220.1880
☑	900	1131.2676	2260.5206
☑	901	761.3976	2281.1710
☑	902	761.7251	2282.1535
☑	904	768.6889	2303.0449
☑	913	800.0605	2397.1597
☑	925	860.3876	2578.1410
☑	926	880.1218	2637.3436
☑	932	893.7734	2678.2984
☑	933	894.0991	2679.2755
☑	935	927.7458	2780.2156
☑	940	933.7698	2798.2876
☑	946	961.0908	2880.2506
☑	948	961.7506	2882.2300
☑	949	972.4224	2914.2454
☑	950	978.5491	2932.6255
☑	956	820.6473	3278.5601
☑	958	1130.5444	3388.6114
☑	959	1130.5486	3388.6240
☑	961	1493.0217	4476.0433

Search Parameters

```
Type of search         : MS/MS Ion Search
Enzyme                 : Trypsin
Fixed modifications    : Carbamidomethyl (C)
Variable modifications : Oxidation (M)
Mass values            : Monoisotopic
Protein Mass           : Unrestricted
Peptide Mass Tolerance : ± 50 ppm
Fragment Mass Tolerance: ± 0.2 Da
Max Missed Cleavages   : 1
Instrument type        : ESI-QUAD-TOF
Number of queries      : 961
```

Mascot: http://www.matrixscience.com/

Mascot Search Results

```
User                    : Dominic
Email                   : dominic.kurian@bbsrc.ac.uk
Search title            : IPI Mouse_15-02-2010
MS data file            : C:\Program Files\Matrix Science\Mascot Daemon\mgf\31 MSadmin\mascot_daemon_merge.mgf
Database                : IPI_mouse MOUSE_v3_79 (54943 sequences; 24917557 residues)
Timestamp               : 15 Feb 2011 at 11:16:52 GMT
Enzyme                  : Trypsin
Fixed modifications     : Carbamidomethyl (C)
Variable modifications  : Oxidation (M)
Mass values             : Monoisotopic
Protein Mass            : Unrestricted
Peptide Mass Tolerance  : ± 50 ppm
Fragment Mass Tolerance : ± 0.2 Da
Max Missed Cleavages    : 1
Instrument type         : ESI-QUAD-TOF
Number of queries       : 1043
Protein hits            : IPI00420725 Tax_Id=10090 Gene_Symbol=Camk2a Uncharacterized protein
                          IPI00474502 Tax_Id=10090 Gene_Symbol=Camk2b Calcium/calmodulin-dependent protein kinase type II subunit beta
                          IPI00473320 Tax_Id=10090 Gene_Symbol=Actb Putative uncharacterized protein
                          IPI00136929 Tax_Id=10090 Gene_Symbol=Actg1 Gamma actin-like protein
                          IPI00112584 Tax_Id=10090 Gene_Symbol=Camk2d Isoform 1 of Calcium/calmodulin-dependent protein kinase type II
                          IPI00124695 Tax_Id=10090 Gene_Symbol=Camk2g Isoform 1 of Calcium/calmodulin-dependent protein kinase type II
                          IPI00221528 Tax_Id=10090 Gene_Symbol=Actbl2 Beta-actin-like protein 2
                          IPI00169463 Tax_Id=10090 Gene_Symbol=Tubb2c Tubulin beta-2C chain
                          IPI00109073 Tax_Id=10090 Gene_Symbol=Tubb4 Tubulin beta-4 chain
                          IPI00109061 Tax_Id=10090 Gene_Symbol=Tubb2b Tubulin beta-2B chain
                          IPI00136703 Tax_Id=10090 Gene_Symbol=Ckb Creatine kinase B-type
                          IPI00420569 Tax_Id=10090 Gene_Symbol=Atp1a2 Sodium/potassium-transporting ATPase subunit alpha-2
                          IPI00122048 Tax_Id=10090 Gene_Symbol=Atp1a3 Sodium/potassium-transporting ATPase subunit alpha-3
                          IPI00117352 Tax_Id=10090 Gene_Symbol=Tubb5 Tubulin beta-5 chain
                          IPI00115546 Tax_Id=10090 Gene_Symbol=Gnao1 Isoform Alpha-2 of Guanine nucleotide-binding protein G(o) subunit
                          IPI00110753 Tax_Id=10090 Gene_Symbol=Tuba1a Tubulin alpha-1A chain
                          IPI00120793 Tax_Id=10090 Gene_Symbol=Prnp Major prion protein
                          IPI00112251 Tax_Id=10090 Gene_Symbol=Tubb3 Tubulin beta-3 chain
                          IPI00122928 Tax_Id=10090 Gene_Symbol=Tubb6 Tubulin beta-6 chain
                          IPI00135284 Tax_Id=10090 Gene_Symbol=Gm16374 Glyceraldehyde-3-phosphate dehydrogenase
                          IPI00135106 Tax_Id=10090 Gene_Symbol=Mobp Isoform 1 of Myelin-associated oligodendrocyte basic protein
                          IPI00114375 Tax_Id=10090 Gene_Symbol=Dpysl2 Dihydropyrimidinase-related protein 2
                          IPI00132042 Tax_Id=10090 Gene_Symbol=Pdhb Pyruvate dehydrogenase E1 component subunit beta, mitochondrial
                          IPI00138892 Tax_Id=10090 Gene_Symbol=Uba52 Ubiquitin-60S ribosomal protein L40
                          IPI00121038 Tax_Id=10090 Gene_Symbol=Vcan Isoform V0 of Versican core protein
                          IPI00323571 Tax_Id=10090 Gene_Symbol=Apoe Apolipoprotein E
                          IPI00229525 Tax_Id=10090 Gene_Symbol=Gnal Guanine nucleotide-binding protein G(olf) subunit alpha
                          IPI00118569 Tax_Id=10090 Gene_Symbol=Gna13 Guanine nucleotide-binding protein subunit alpha-13
                          IPI00129240 Tax_Id=10090 Gene_Symbol=Vtn Vitronectin
                          IPI00323357 Tax_Id=10090 Gene_Symbol=Hspa8 Heat shock cognate 71 kDa protein
                          IPI00131366 Tax_Id=10090 Gene_Symbol=Krt6b Keratin, type II cytoskeletal 6B
                          IPI00134344 Tax_Id=10090 Gene_Symbol=Spnb3 Putative uncharacterized protein
                          IPI00123313 Tax_Id=10090 Gene_Symbol=Uba1 Ubiquitin-like modifier-activating enzyme 1
                          IPI00113112 Tax_Id=10090 Gene_Symbol=Rab3b Ras-related protein Rab-3B
                          IPI00116697 Tax_Id=10090 Gene_Symbol=Rab6 Isoform 1 of Ras-related protein Rab-6A
                          IPI00136271 Tax_Id=10090 Gene_Symbol=Rab33b Putative uncharacterized protein
                          IPI00170032 Tax_Id=10090 Gene_Symbol=Rab15 Ras-related protein Rab-15
                          IPI00656325 Tax_Id=10090 Gene_Symbol=Nsf Vesicle-fusing ATPase
```

	IPI_mouse	Decoy	False discovery rate
Peptide matches above identity threshold	123	0	0.00 %
Peptide matches above homology or identity threshold	143	2	1.40 %

Select Summary Report

Format As   Select Summary (protein hits)   Help

Significance threshold p< 0.01   Max. number of hits AUTO

Standard scoring   MudPIT scoring   Ions score or expect cut-off 0   Show sub-sets 0

Show pop-ups   Suppress pop-ups   Sort unassigned Decreasing Score   Require bold red

1. IPI00420725 **Mass:** 55882 **Score:** 883 **Queries matched:** 42 **emPAI:** 1.23
Tax_Id=10090 Gene_Symbol=Camk2a Uncharacterized protein

Query	Observed	Mr(expt)	Mr(calc)	ppm	Miss	Score	Expect	Rank	Peptide
245	432.7284	863.4422	863.4501	-9.12	0	17	2.1	1	R.SGAPSVLPH.-
261	446.2625	890.5104	890.5086	2.03	1	4	22	2	K.GAFSVVRR.C
283	463.2463	924.4780	924.4777	0.37	1	25	0.2	1	R.DHQKLER.E 282 284
373	525.2643	1048.5140	1048.5553	-39.33	0	53	0.00048	1	K.VLAGQEYAAK.I 374
384	530.3027	1058.5908	1058.5906	0.21	1	(43)	0.0034	1	K.MLTINPSKR.I 383 385
398	538.2980	1074.5814	1074.5855	-3.81	1	52	0.00054	1	K.MLTINPSKR.I 399 400 401
424	545.3502	1088.6858	1088.6818	3.70	1	37	0.003	1	R.LLKHPNIVR.L
480	574.3285	1146.6424	1146.6431	-0.54	0	64	2.5e-005	1	K.GAILTTMLATR.N
497	582.3258	1162.6370	1162.6380	-0.81	0	(59)	8.6e-005	1	K.GAILTTMLATR.N 498
586	631.3029	1260.5912	1260.5927	-1.19	0	58	0.0001	1	R.FYFENLWSR.N 585 587
628	652.3355	1302.6564	1302.6932	-28.21	0	73	4.2e-006	1	R.ITQYLDAGGIPR.T 629 630 632
669	670.8956	1339.7766	1339.7711	4.16	0	74	1.2e-006	1	R.DLKPENLLLASK.L 664 665 666 667 668
836	816.8978	1631.7810	1631.7719	5.62	0	68	1.1e-005	1	R.FTEEYQLFEELGK.G 832 835
937	1040.9652	2079.9158	2079.9426	-12.84	0	77	6.5e-007	1	K.AGAYDFPSPEWDTVTPEAK.D 936
983	783.7271	2348.1595	2348.1689	-4.00	1	78	7.4e-007	1	R.FTEEYQLFEELGKGAFSVVR.R 982 984

1005	888.7645	2663.2717	2663.2755	-1.44	1	101	3.3e-009	1	K.AGAYDFPSPEWDTVTPEAKDLINK.M 1004 1006

Proteins matching the same set of peptides:
IPI00621806 **Mass:** 54651 **Score:** 883 **Queries matched:** 42
Tax_Id=10090 Gene_Symbol=Camk2a Isoform Alpha CaMKII of Calcium/calmodulin-dependent protein kinase type II subunit alpha

---

2. IPI00474502 **Mass:** 61164 **Score:** 771 **Queries matched:** 36 **emPAI:** 1.57
Tax_Id=10090 Gene_Symbol=Camk2b Calcium/calmodulin-dependent protein kinase type II subunit beta

Query	Observed	Mr(expt)	Mr(calc)	ppm	Miss	Score	Expect	Rank	Peptide
261	446.2625	890.5104	890.5086	2.03	1	4	22	2	K.GAFSVVRR.C
283	463.2463	924.4780	924.4777	0.37	1	25	0.2	1	R.DHQKLER.E 282 284
376	526.2389	1050.4632	1050.4586	4.38	0	21	0.3	1	R.STVASMMHR.Q
477	572.8038	1143.5930	1143.5964	-2.95	0	15	2.9	1	R.FYFENLLAK.N 478
480	574.3285	1146.6424	1146.6431	-0.54	0	64	2.5e-005	1	K.GAILTTMLATR.N
497	582.3258	1162.6370	1162.6380	-0.81	0	(59)	8.6e-005	1	K.GAILTTMLATR.N 498
573	623.3220	1244.6294	1244.6361	-5.33	0	43	0.0047	1	K.ADGVKPQTNSTK.N
669	670.8956	1339.7766	1339.7711	4.16	0	74	1.2e-006	1	R.DLKPENLLLASK.C 664 665 666 667 668
692	458.5797	1372.7173	1372.7310	-10.02	1	24	0.27	1	K.KADGVKPQTNSTK.N
712	702.3754	1402.7362	1402.7317	3.24	0	60	6.5e-005	1	R.LTQYIDGQGRPR.T 711 713
821	810.8781	1619.7416	1619.7355	3.79	0	80	5.2e-007	1	R.FTDEYQLYEDIGK.G
827	542.6376	1624.8910	1624.9082	-10.63	1	72	2.8e-006	1	K.NLINQMLTINPAKR.I 826
937	1040.9652	2079.9158	2079.9426	-12.84	0	77	6.5e-007	1	K.AGAYDFPSPEWDTVTPEAK.N 936
945	1057.0130	2112.0114	2112.0011	4.90	0	90	5e-008	1	K.TTEQLIEAVNNGDFEAYAK.I 942 944
981	779.7127	2336.1163	2336.1325	-6.94	1	98	6.8e-009	1	R.FTDEYQLYEDIGKGAFSVVR.R 979 980
988	1177.0770	2352.1394	2352.1301	3.97	0	92	2.6e-008	1	R.QTTAPATMSTAASGTTMGLVEQAK.S 986
989	785.4238	2353.2496	2353.2642	-6.20	0	61	2.7e-005	1	K.GSLPPAALEPQTTVIHNPVDGIK.E
991	1185.0753	2368.1360	2368.1250	4.65	0	(66)	1.1e-005	1	R.QTTAPATMSTAASGTTMGLVEQAK.S

Proteins matching the same set of peptides:
IPI00649778 **Mass:** 73827 **Score:** 771 **Queries matched:** 36
Tax_Id=10090 Gene_Symbol=Camk2b Calcium/calmodulin-dependent protein kinase II, beta, isoform CRA_b
IPI00875723 **Mass:** 68686 **Score:** 771 **Queries matched:** 36
Tax_Id=10090 Gene_Symbol=Camk2b 68 kDa protein

---

3. IPI00473320 **Mass:** 42115 **Score:** 665 **Queries matched:** 20 **emPAI:** 1.13
Tax_Id=10090 Gene_Symbol=Actb Putative uncharacterized protein

Query	Observed	Mr(expt)	Mr(calc)	ppm	Miss	Score	Expect	Rank	Peptide
94	630.2972	629.2899	629.2769	20.7	0	21	0.16	1	R.GDDAPR.A
494	581.3107	1160.6068	1160.6111	-3.65	0	23	0.45	1	K.EITALAPSTMK.I
530	599.8568	1197.6990	1197.6982	0.69	0	51	0.00032	1	R.AVFPSIVGRPR.H 531
676	677.8154	1353.6162	1353.6161	0.14	1	80	5.7e-007	1	K.DSYVGDEAQSKR.G
757	774.9159	1547.8172	1547.8051	7.85	1	93	3.4e-008	1	R.MQKEITALAPSTMK.I 755 756
824	812.4301	1622.8456	1622.8338	7.32	1	16	1.3	1	R.LDLAGRDLTDYLMK.I
885	895.9537	1789.8928	1789.8846	4.59	0	103	3.1e-009	1	K.SYELPDGQVITIGNER.F 886
918	652.0211	1953.0415	1953.0571	-8.00	0	73	2e-006	1	R.VAPEEHPVLLTEAPLNPK.A 917 919
966	1108.0448	2214.0750	2214.0627	5.58	0	71	3.7e-006	1	K.DLYANTVLSGGTTMYPGIADR.M
1038	1061.8691	3182.5855	3182.6071	-6.78	0	109	3.5e-010	1	R.TTGIVMDSGDGVTHTVPIYEGYALPHAILR.L 1035 1036 1037
1039	800.6558	3198.5941	3198.6020	-2.46	0	(84)	1.2e-007	1	R.TTGIVMDSGDGVTHTVPIYEGYALPHAILR.L

---

4. IPI00136929 **Mass:** 44029 **Score:** 627 **Queries matched:** 17 **emPAI:** 0.92
Tax_Id=10090 Gene_Symbol=Actg1 Gamma actin-like protein

Query	Observed	Mr(expt)	Mr(calc)	ppm	Miss	Score	Expect	Rank	Peptide
494	581.3107	1160.6068	1160.6111	-3.65	0	23	0.45	1	K.EITALAPSTMK.I
676	677.8154	1353.6162	1353.6161	0.14	1	80	5.7e-007	1	K.DSYVGDEAQSKR.G
757	774.9159	1547.8172	1547.8051	7.85	1	93	3.4e-008	1	R.MQKEITALAPSTMK.I 755 756
824	812.4301	1622.8456	1622.8338	7.32	1	16	1.3	1	R.LDLAGRDLTDYLMK.I
885	895.9537	1789.8928	1789.8846	4.59	0	103	3.1e-009	1	K.SYELPDGQVITIGNER.F 886
918	652.0211	1953.0415	1953.0571	-8.00	0	73	2e-006	1	R.VAPEEHPVLLTEAPLNPK.R 917 919
966	1108.0448	2214.0750	2214.0627	5.58	0	71	3.7e-006	1	K.DLYANTVLSGGTTMYPGLADR.M
1038	1061.8691	3182.5855	3182.6071	-6.78	0	109	3.5e-010	1	R.TTGIVMDSGDGVTHTVPIYEGYALPHAILR.L 1035 1036 1037
1039	800.6558	3198.5941	3198.6020	-2.46	0	(84)	1.2e-007	1	R.TTGIVMDSGDGVTHTVPIYEGYALPHAILR.L

---

5. IPI00112584 **Mass:** 56961 **Score:** 456 **Queries matched:** 24 **emPAI:** 0.66
Tax_Id=10090 Gene_Symbol=Camk2d Isoform 1 of Calcium/calmodulin-dependent protein kinase type II subunit delta

Query	Observed	Mr(expt)	Mr(calc)	ppm	Miss	Score	Expect	Rank	Peptide
261	446.2625	890.5104	890.5086	2.03	1	4	22	2	K.GAFSVVRR.C
283	463.2463	924.4780	924.4777	0.37	1	25	0.2	1	R.DHQKLER.E 282 284
376	526.2389	1050.4632	1050.4586	4.38	0	21	0.3	1	R.STVASMMHR.Q
383	530.2958	1058.5770	1058.5906	-12.82	1	20	0.97	2	K.MLTINPAKR.I
424	545.3502	1088.6858	1088.6818	3.70	1	37	0.003	1	R.LLKHPNIVR.L
460	559.7792	1117.5438	1117.5444	-0.48	0	13	4.2	1	R.FYFENALSK.S
480	574.3285	1146.6424	1146.6431	-0.54	0	64	2.5e-005	1	K.GAILTTMLATR.N
497	582.3258	1162.6370	1162.6380	-0.81	0	(59)	8.6e-005	1	K.GAILTTMLATR.N 498
669	670.8956	1339.7766	1339.7711	4.16	0	74	1.2e-006	1	R.DLKPENLLLASK.S 664 665 666 667 668
679	680.3028	1358.5910	1358.5846	4.72	0	20	0.3	1	R.LTQYMDSGGMPK.T
819	809.8877	1617.7608	1617.7562	2.85	0	59	6.8e-005	1	R.FTDEYQLFEELGK.G
937	1040.9652	2079.9158	2079.9426	-12.84	0	77	6.5e-007	1	K.AGAYDFPSPEWDTVTPEAK.D 936
1005	888.7645	2663.2717	2663.2755	-1.44	1	101	3.3e-009	1	K.AGAYDFPSPEWDTVTPEAKDLINK.M 1004 1006

Proteins matching the same set of peptides:
IPI00406790 **Mass:** 60593 **Score:** 456 **Queries matched:** 24
Tax_Id=10090 Gene_Symbol=Camk2d Isoform 4 of Calcium/calmodulin-dependent protein kinase type II subunit delta
IPI00475044 **Mass:** 55899 **Score:** 456 **Queries matched:** 24
Tax_Id=10090 Gene_Symbol=Camk2d Isoform 2 of Calcium/calmodulin-dependent protein kinase type II subunit delta
IPI00828919 **Mass:** 58492 **Score:** 456 **Queries matched:** 24
Tax_Id=10090 Gene_Symbol=Camk2d Isoform 3 of Calcium/calmodulin-dependent protein kinase type II subunit delta

IPI00857865 **Mass:** 54664 **Score:** 456 **Queries matched:** 24
Tax_Id=10090 Gene_Symbol=Camk2d Putative uncharacterized protein
IPI00858128 **Mass:** 58283 **Score:** 456 **Queries matched:** 24
Tax_Id=10090 Gene_Symbol=Camk2d calcium/calmodulin-dependent protein kinase type II subunit delta isoform 1
IPI00858144 **Mass:** 55828 **Score:** 456 **Queries matched:** 24
Tax_Id=10090 Gene_Symbol=Camk2d 55 kDa protein

---

6. IPI00124695 **Mass:** 60254 **Score:** 420 **Queries matched:** 21 **emPAI:** 0.79
Tax_Id=10090 Gene_Symbol=Camk2g Isoform 1 of Calcium/calmodulin-dependent protein kinase type II subunit gamma

Query	Observed	Mr(expt)	Mr(calc)	ppm	Miss	Score	Expect	Rank	Peptide
261	446.2625	890.5104	890.5086	2.03	1	4	22	2	K.GAFSVVRR.C
283	463.2463	924.4780	924.4777	0.37	1	25	0.2	1	R.DHQKLER.E 282 284
376	526.2389	1050.4632	1050.4586	4.38	0	21	0.3	1	R.STVASMMHR.Q
424	545.3502	1088.6858	1088.6818	3.70	1	37	0.003	1	R.LLKHPNIVR.L
669	670.8956	1339.7766	1339.7711	4.16	0	74	1.2e-006	1	R.DLKPENLLLASK.C 664 665 666 667 668
712	702.3754	1402.7362	1402.7317	3.24	0	60	6.5e-005	1	R.LTQYIDGQGRPR.T 711 713
811	802.8796	1603.7446	1603.7406	2.52	0	77	1e-006	1	R.FTDDYQLFEELGK.G
827	542.6376	1624.8910	1624.9082	-10.63	1	72	2.8e-006	1	K.NLINQMLTINPAKR.I 826
937	1040.9652	2079.9158	2079.9426	-12.84	0	77	6.5e-007	1	K.AGAYDFPSPEWDTVTPEAK.N 936
978	774.3764	2320.1074	2320.1376	-13.02	1	53	0.00023	1	R.FTDDYQLFEELGKGAFSVVR.R

Proteins matching the same set of peptides:
IPI00228044 **Mass:** 59013 **Score:** 420 **Queries matched:** 21
Tax_Id=10090 Gene_Symbol=Camk2g Isoform 2 of Calcium/calmodulin-dependent protein kinase type II subunit gamma
IPI00228045 **Mass:** 56610 **Score:** 420 **Queries matched:** 21
Tax_Id=10090 Gene_Symbol=Camk2g Isoform 3 of Calcium/calmodulin-dependent protein kinase type II subunit gamma

---

7. IPI00221528 **Mass:** 42319 **Score:** 401 **Queries matched:** 11 **emPAI:** 0.46
Tax_Id=10090 Gene_Symbol=Actbl2 Beta-actin-like protein 2

Query	Observed	Mr(expt)	Mr(calc)	ppm	Miss	Score	Expect	Rank	Peptide
824	812.4301	1622.8456	1622.8338	7.32	1	16	1.3	1	R.LDLAGRDLTDYLMK.I
885	895.9537	1789.8928	1789.8846	4.59	0	103	3.1e-009	1	R.SYELPDGQVITIGNER.F 886
918	652.0211	1953.0415	1953.0571	-8.00	0	47	0.00096	2	R.VAPDEHPILLTEAPLNPK.I 917 919
1038	1061.8691	3182.5855	3182.6071	-6.78	0	109	3.5e-010	1	R.TTGIVMDSGDGVTHTVPIYEGYALPHAILR.L 1035 1036 1037
1039	800.6558	3198.5941	3198.6020	-2.46	0	(84)	1.2e-007	1	R.TTGIVMDSGDGVTHTVPIYEGYALPHAILR.L

---

8. IPI00169463 **Mass:** 50255 **Score:** 392 **Queries matched:** 19 **emPAI:** 1.01
Tax_Id=10090 Gene_Symbol=Tubb2c Tubulin beta-2C chain

Query	Observed	Mr(expt)	Mr(calc)	ppm	Miss	Score	Expect	Rank	Peptide
404	539.2755	1076.5364	1076.5250	10.6	1	21	0.68	1	K.IREEYPDR.I 405
554	615.3074	1228.6002	1228.5910	7.50	0	66	1.6e-005	1	R.ISEQFTAMFR.R
651	660.3555	1318.6964	1318.6955	0.71	0	70	7e-006	1	R.IMNTFSVVPSPK.V
806	801.4128	1600.8110	1600.8131	-1.26	0	62	4.4e-005	1	R.AVLVDLEPGTMDSVR.S
817	809.4195	1616.8244	1616.8080	10.2	0	(37)	0.014	1	R.AVLVDLEPGTMDSVR.S 818
823	540.9528	1619.8366	1619.8283	5.13	0	53	0.00035	1	R.LHFFMPGFAPLTSR.G 822
838	818.9071	1635.7996	1635.8232	-14.39	0	(15)	2.2	1	R.LHFFMPGFAPLTSR.G
849	846.4417	1690.8688	1690.8600	5.23	0	71	5e-006	1	R.ALTVPELTQQMFDAK.N
850	848.9232	1695.8318	1695.8257	3.65	0	53	0.0003	1	K.NSSYFVEWIPNNVK.T 851
905	929.4750	1856.9354	1856.9342	0.66	0	35	0.017	2	K.MSATFIGNSTAIQELFK.R
924	979.9998	1957.9850	1957.9745	5.37	0	44	0.0021	1	K.GHYTEGAELVDSVLDVVR.K
931	672.0128	2013.0166	2013.0353	-9.32	1	98	9.4e-009	1	K.MSATFIGNSTAIQELFKR.I
1012	933.4506	2797.3300	2797.3361	-2.20	0	88	5.4e-008	1	R.SGPFGQIFRPDNFVFGQSGAGNNWAK.G 1011 1013

---

9. IPI00109073 **Mass:** 50010 **Score:** 375 **Queries matched:** 17 **emPAI:** 0.89
Tax_Id=10090 Gene_Symbol=Tubb4 Tubulin beta-4 chain

Query	Observed	Mr(expt)	Mr(calc)	ppm	Miss	Score	Expect	Rank	Peptide
389	531.2863	1060.5580	1060.5301	26.3	1	10	8.7	5	K.IREEFPDR.I
554	615.3074	1228.6002	1228.5910	7.50	0	66	1.6e-005	1	R.ISEQFTAMFR.R
651	660.3555	1318.6964	1318.6955	0.71	0	70	7e-006	1	R.IMNTFSVVPSPK.V
806	801.4128	1600.8110	1600.8131	-1.26	0	62	4.4e-005	1	R.AVLVDLEPGTMDSVR.S
817	809.4195	1616.8244	1616.8080	10.2	0	(37)	0.014	1	R.AVLVDLEPGTMDSVR.S 818
823	540.9528	1619.8366	1619.8283	5.13	0	53	0.00035	1	R.LHFFMPGFAPLTSR.G 822
838	818.9071	1635.7996	1635.8232	-14.39	0	(15)	2.2	1	R.LHFFMPGFAPLTSR.G
849	846.4417	1690.8688	1690.8600	5.23	0	71	5e-006	1	R.ALTVPELTQQMFDAK.N
850	848.9232	1695.8318	1695.8257	3.65	0	53	0.0003	1	K.NSSYFVEWIPNNVK.T 851
905	929.4750	1856.9354	1856.9342	0.66	0	37	0.012	1	K.MAATFIGNSTAIQELFK.R
931	672.0128	2013.0166	2013.0353	-9.32	1	98	9.8e-009	2	K.MAATFIGNSTAIQELFKR.I
1012	933.4506	2797.3300	2797.3361	-2.20	0	88	5.4e-008	1	R.SGPFGQIFRPDNFVFGQSGAGNNWAK.G 1011 1013

---

10. IPI00109061 **Mass:** 50377 **Score:** 358 **Queries matched:** 20 **emPAI:** 0.88
Tax_Id=10090 Gene_Symbol=Tubb2b Tubulin beta-2B chain

Query	Observed	Mr(expt)	Mr(calc)	ppm	Miss	Score	Expect	Rank	Peptide
404	539.2755	1076.5364	1076.5250	10.6	1	21	0.68	1	K.IREEYPDR.I 405
554	615.3074	1228.6002	1228.5910	7.50	0	66	1.6e-005	1	R.ISEQFTAMFR.R
675	676.3422	1350.6698	1350.6676	1.69	0	73	3.5e-006	1	R.IMNTFSVMPSPK.V
814	808.4207	1614.8268	1614.8287	-1.15	0	47	0.0015	1	R.AILVDLEPGTMDSVR.S
823	540.9528	1619.8366	1619.8283	5.13	0	53	0.00035	1	R.LHFFMPGFAPLTSR.G 822
829	816.4133	1630.8120	1630.8236	-7.10	0	(41)	0.0058	1	R.AILVDLEPGTMDSVR.S 830 831
838	818.9071	1635.7996	1635.8232	-14.39	0	(15)	2.2	1	R.LHFFMPGFAPLTSR.G
850	848.9232	1695.8318	1695.8257	3.65	0	53	0.0003	1	K.NSSYFVEWIPNNVK.T 851
905	929.4750	1856.9354	1856.9342	0.66	0	35	0.017	2	K.MSATFIGNSTAIQELFK.R
924	979.9998	1957.9850	1957.9745	5.37	0	44	0.0021	1	K.GHYTEGAELVDSVLDVVR.K
931	672.0128	2013.0166	2013.0353	-9.32	1	98	9.4e-009	1	K.MSATFIGNSTAIQELFKR.I
1012	933.4506	2797.3300	2797.3361	-2.20	0	88	5.4e-008	1	R.SGPFGQIFRPDNFVFGQSGAGNNWAK.G 1011 1013
1034	776.3489	3101.3665	3101.4003	-10.89	0	7	4.2	1	K.FWEVISDEHGIDPTGSYHGDSDLQLER.I

Proteins matching the same set of peptides:
IPI00338039 **Mass:** 50274 **Score:** 358 **Queries matched:** 20
Tax_Id=10090 Gene_Symbol=Tubb2a Tubulin beta-2A chain

---

11. IPI00136703 **Mass:** 42971 **Score:** 341 **Queries matched:** 8 **emPAI:** 0.45
Tax_Id=10090 Gene_Symbol=Ckb Creatine kinase B-type

Query	Observed	Mr(expt)	Mr(calc)	ppm	Miss	Score	Expect	Rank	Peptide
632	652.3672	1302.7198	1302.7183	1.17	0	84	2.2e-007	1	K.VLTPELYAELR.A 631
809	**801.9227**	**1601.8308**	**1601.8260**	**3.00**	**0**	**96**	**1.7e-008**	**1**	**K.LAVEALSSLDGDLSGR.Y**
841	**828.9185**	**1655.8224**	**1655.8188**	**2.17**	**0**	**72**	**4.3e-006**	**1**	**R.LEQGQAIDDLMPAQK.-**
903	**924.9891**	**1847.9636**	**1847.9703**	**-3.60**	**0**	**31**	**0.044**	**1**	**R.LGFSEVELVQMVVDGVK.L**
1040	**897.6892**	**3586.7277**	**3586.7800**	**-14.57**	**0**	**87**	**4.9e-008**	**1**	**K.SMTEAEQQQLIDDHFLFDKPVSPLLLASGMAR.D** 1041 1042

---

12. IPI00420569 **Mass:** 113457 **Score:** 312 **Queries matched:** 7 **emPAI:** 0.12
Tax_Id=10090 Gene_Symbol=Atp1a2 Sodium/potassium-transporting ATPase subunit alpha-2

Query	Observed	Mr(expt)	Mr(calc)	ppm	Miss	Score	Expect	Rank	Peptide
558	**618.8573**	**1235.7000**	**1235.6986**	**1.17**	**0**	**52**	**0.00022**	**1**	**R.LNIPVSQVNPR.E** 559
590	**634.8586**	**1267.7026**	**1267.7071**	**-3.48**	**0**	**22**	**0.3**	**1**	**K.NMVPQQALVIR.E**
898	**906.4799**	**1810.9452**	**1810.9425**	**1.51**	**0**	**103**	**2.8e-009**	**1**	**R.QGAIVAVTGDGVNDSPALK.K** 897
902	**915.4670**	**1828.9194**	**1828.9167**	**1.53**	**0**	**124**	**2.5e-011**	**1**	**K.GVGIISEGNETVEDIAAR.L**
1020	**952.1171**	**2853.3295**	**2853.3313**	**-0.64**	**1**	**53**	**0.00017**	**1**	**K.EIPLDKEMQDAFQNAYMELGGLGER.V**

Proteins matching the same set of peptides:
IPI00762871 **Mass:** 104717 **Score:** 312 **Queries matched:** 7
Tax_Id=10090 Gene_Symbol=Atp1a2 Uncharacterized protein

---

13. IPI00122048 **Mass:** 113045 **Score:** 309 **Queries matched:** 7 **emPAI:** 0.12
Tax_Id=10090 Gene_Symbol=Atp1a3 Sodium/potassium-transporting ATPase subunit alpha-3

Query	Observed	Mr(expt)	Mr(calc)	ppm	Miss	Score	Expect	Rank	Peptide
558	618.8573	1235.7000	1235.6986	1.17	0	52	0.00022	1	R.LNIPVSQVNPR.D 559
590	634.8586	1267.7026	1267.7071	-3.48	0	22	0.3	1	K.NMVPQQALVIR.E
898	906.4799	1810.9452	1810.9425	1.51	0	103	2.8e-009	1	R.QGAIVAVTGDGVNDSPALK.K 897
902	915.4670	1828.9194	1828.9167	1.53	0	124	2.5e-011	1	K.GVGIISEGNETVEDIAAR.L
1022	**956.1228**	**2865.3466**	**2865.3490**	**-0.86**	**1**	**48**	**0.00055**	**1**	**K.EQPLDEEMKEAFQNAYLELGGLGER.V**

Proteins matching the same set of peptides:
IPI00752412 **Mass:** 117548 **Score:** 309 **Queries matched:** 7
Tax_Id=10090 Gene_Symbol=Atp1a3 ATPase, Na+/K+ transporting, alpha 3 polypeptide, isoform CRA_c

---

14. IPI00117352 **Mass:** 50095 **Score:** 290 **Queries matched:** 17 **emPAI:** 0.78
Tax_Id=10090 Gene_Symbol=Tubb5 Tubulin beta-5 chain

Query	Observed	Mr(expt)	Mr(calc)	ppm	Miss	Score	Expect	Rank	Peptide
404	539.2755	1076.5364	1076.5250	10.6	1	21	0.68	1	K.IREEYPDR.I 405
554	615.3074	1228.6002	1228.5910	7.50	0	66	1.6e-005	1	R.ISEQFTAMFR.R
651	660.3555	1318.6964	1318.6955	0.71	0	70	7e-006	1	R.IMNTFSVVPSPK.V
814	808.4207	1614.8268	1614.8287	-1.15	0	47	0.0015	1	R.AILVDLEPGTMDSVR.S
823	540.9528	1619.8366	1619.8283	5.13	0	53	0.00035	1	R.LHFFMPGFAPLTSR.G 822
829	816.4133	1630.8120	1630.8236	-7.10	0	(41)	0.0058	1	R.AILVDLEPGTMDSVR.S 830 831
838	818.9071	1635.7996	1635.8232	-14.39	0	(15)	2.2	1	R.LHFFMPGFAPLTSR.G
850	848.9232	1695.8318	1695.8257	3.65	0	53	0.0003	1	K.NSSYFVEWIPNNVK.T 851
924	979.9998	1957.9850	1957.9745	5.37	0	44	0.0021	1	K.GHYTEGAELVDSVLDVVR.K
1012	933.4506	2797.3300	2797.3361	-2.20	0	88	5.4e-008	1	R.SGPFGQIFRPDNFVFGQSGAGNNWAK.G 1011 1013

---

15. IPI00115546 **Mass:** 40524 **Score:** 278 **Queries matched:** 6 **emPAI:** 0.37
Tax_Id=10090 Gene_Symbol=Gnao1 Isoform Alpha-2 of Guanine nucleotide-binding protein G(o) subunit alpha

Query	Observed	Mr(expt)	Mr(calc)	ppm	Miss	Score	Expect	Rank	Peptide
380	**529.3142**	**1056.6138**	**1056.6179**	**-3.82**	**0**	**77**	**1.3e-006**	**1**	**K.LLLLGAGESGK.S** 381
799	**792.3738**	**1582.7330**	**1582.7297**	**2.11**	**1**	**53**	**0.00024**	**1**	**R.AMDTLGVEYGDKER.K**
845	**838.4210**	**1674.8274**	**1674.8213**	**3.67**	**0**	**101**	**5.3e-009**	**1**	**R.IGAGDYQPTEQDILR.T** 844
922	**979.4433**	**1956.8720**	**1956.8631**	**4.58**	**0**	**69**	**4.7e-006**	**1**	**R.MEDTEPFSAELLSAMMR.L**

Proteins matching the same set of peptides:
IPI00230192 **Mass:** 40629 **Score:** 278 **Queries matched:** 6
Tax_Id=10090 Gene_Symbol=Gnao1 Isoform Alpha-1 of Guanine nucleotide-binding protein G(o) subunit alpha

---

16. IPI00110753 **Mass:** 50788 **Score:** 246 **Queries matched:** 7 **emPAI:** 0.29
Tax_Id=10090 Gene_Symbol=Tuba1a Tubulin alpha-1A chain

Query	Observed	Mr(expt)	Mr(calc)	ppm	Miss	Score	Expect	Rank	Peptide
351	**508.2901**	**1014.5656**	**1014.5709**	**-5.21**	**0**	**8**	**15**	**5**	**K.DVNAAIATIK.T**
739	**729.4378**	**1456.8610**	**1456.8613**	**-0.18**	**0**	**20**	**0.2**	**1**	**R.LIGQIVSSITASLR.F**
855	**851.4660**	**1700.9174**	**1700.8985**	**11.1**	**0**	**91**	**3.7e-008**	**1**	**R.AVFVDLEPTVIDEVR.T** 854
868	**586.3238**	**1755.9496**	**1755.9559**	**-3.62**	**0**	**49**	**0.00055**	**1**	**R.IHFPLATYAPVISAEK.A**
900	**913.0059**	**1823.9972**	**1823.9782**	**10.5**	**0**	**32**	**0.021**	**1**	**K.VGINYQPPTVVPGGDLAK.V**
994	**1205.1074**	**2408.2002**	**2408.2012**	**-0.41**	**0**	**112**	**2.7e-010**	**1**	**R.FDGALNVDLTEFQTNLVPYPR.I**

---

17. IPI00120793 **Mass:** 28131 **Score:** 236 **Queries matched:** 13 **emPAI:** 0.75
Tax_Id=10090 Gene_Symbol=Prnp Major prion protein

Query	Observed	Mr(expt)	Mr(calc)	ppm	Miss	Score	Expect	Rank	Peptide
354	**508.7722**	**1015.5298**	**1015.5298**	**0.00**	**0**	**32**	**0.074**	**1**	**K.QHTVTTTTK.G**
421	**544.7347**	**1087.4548**	**1087.4570**	**-2.01**	**0**	**41**	**0.0017**	**1**	**K.ESQAYYDGR.R**
474	**570.2632**	**1138.5118**	**1138.5142**	**-2.10**	**0**	**36**	**0.012**	**1**	**K.GENFTETDVK.M** 475
566	**622.7841**	**1243.5536**	**1243.5581**	**-3.61**	**1**	**37**	**0.0081**	**1**	**K.ESQAYYDGRR.S** 567 568 569 570 571
951	**713.0164**	**2136.0274**	**2136.0335**	**-2.88**	**1**	**110**	**4.9e-010**	**1**	**K.QHTVTTTTKGENFTETDVK.M** 950 952

---

18. IPI00112251 **Mass:** 50842 **Score:** 228 **Queries matched:** 11 **emPAI:** 0.55

Tax_Id=10090 Gene_Symbol=Tubb3 Tubulin beta-3 chain

Query	Observed	Mr(expt)	Mr(calc)	ppm	Miss	Score	Expect	Rank	Peptide
554	615.3074	1228.6002	1228.5910	7.50	0	66	1.6e-005	1	R.ISEQFTAMFR.R
651	660.3555	1318.6964	1318.6955	0.71	0	70	7e-006	1	R.IMNTFSVVPSPK.V
814	808.4207	1614.8268	1614.8287	-1.15	0	47	0.0015	1	R.AILVDLEPGTMDSVR.S
822	810.9188	1619.8230	1619.8283	-3.22	0	13	3.2	2	R.LHFFMPGFAPLTAR.G
829	816.4133	1630.8120	1630.8236	-7.10	0	(41)	0.0058	1	R.AILVDLEPGTMDSVR.S 830 831
849	846.4417	1690.8688	1690.8600	5.23	0	71	5e-006	1	R.ALTVPELTQQMFDAK.N
850	848.9232	1695.8318	1695.8257	3.65	0	53	0.0003	1	K.NSSYFVEWIPNNVK.V 851
924	979.9998	1957.9850	1957.9745	5.37	0	44	0.0021	1	K.GHYTEGAELVDSVLDVVR.K

19. IPI00122928 **Mass:** 50514 **Score:** 208 **Queries matched:** 10 **emPAI:** 0.37
Tax_Id=10090 Gene_Symbol=Tubb6 Tubulin beta-6 chain

Query	Observed	Mr(expt)	Mr(calc)	ppm	Miss	Score	Expect	Rank	Peptide
404	539.2755	1076.5364	1076.5250	10.6	1	21	0.68	1	K.IREEYPDR.I 405
675	676.3422	1350.6698	1350.6676	1.69	0	73	3.5e-006	1	R.IMNTFSVMPSPK.V
822	810.9188	1619.8230	1619.8283	-3.22	0	13	3.2	2	R.LHFFMPGFAPLTAR.G
849	846.4417	1690.8688	1690.8600	5.23	0	71	5e-006	1	R.ALTVPELTQQMFDAK.N
850	848.9232	1695.8318	1695.8257	3.65	0	53	0.0003	1	K.NSSYFVEWIPNNVK.V 851
905	929.4750	1856.9354	1856.9342	0.66	0	31	0.046	3	K.MASTFIGNSTAIQELFK.R
924	979.9998	1957.9850	1957.9745	5.37	0	44	0.0021	1	K.GHYTEGAELVDSVLDVVR.K
931	672.0128	2013.0166	2013.0353	-9.32	1	82	3.9e-007	3	K.MASTFIGNSTAIQELFKR.I

20. IPI00135284 **Mass:** 36092 **Score:** 186 **Queries matched:** 4 **emPAI:** 0.30
Tax_Id=10090 Gene_Symbol=Gm16374 Glyceraldehyde-3-phosphate dehydrogenase

Query	Observed	Mr(expt)	Mr(calc)	ppm	Miss	Score	Expect	Rank	Peptide
688	**685.3770**	**1368.7394**	**1368.7361**	**2.45**	**0**	**61**	**4.6e-005**	**1**	**R.GAAQNIIPASTGAAK.A**
691	**686.8327**	**1371.6508**	**1371.6414**	**6.89**	**1**	**52**	**0.00038**	**1**	**R.VVDLMAYMASKE.-**
875	**890.4037**	**1778.7928**	**1778.7900**	**1.61**	**0**	**85**	**1.5e-007**	**1**	**K.LISWYDNEYGYSNR.V** 876

Proteins matching the same set of peptides:
IPI00271869 **Mass:** 36293 **Score:** 186 **Queries matched:** 4
Tax_Id=10090 Gene_Symbol=- 36 kDa protein
IPI00273646 **Mass:** 36072 **Score:** 186 **Queries matched:** 4
Tax_Id=10090 Gene_Symbol=Gapdh;LOC100042025 Glyceraldehyde-3-phosphate dehydrogenase
IPI00622795 **Mass:** 36074 **Score:** 186 **Queries matched:** 4
Tax_Id=10090 Gene_Symbol=- 36 kDa protein
IPI00752289 **Mass:** 36308 **Score:** 186 **Queries matched:** 4
Tax_Id=10090 Gene_Symbol=Gm10290 Glyceraldehyde-3-phosphate dehydrogenase
IPI00848801 **Mass:** 36100 **Score:** 186 **Queries matched:** 4
Tax_Id=10090 Gene_Symbol=- 36 kDa protein
IPI00849045 **Mass:** 36072 **Score:** 186 **Queries matched:** 4
Tax_Id=10090 Gene_Symbol=- 36 kDa protein
IPI00850337 **Mass:** 44633 **Score:** 186 **Queries matched:** 4
Tax_Id=10090 Gene_Symbol=LOC100048117 similar to Glyceraldehyde-3-phosphate dehydrogenase (GAPDH) isoform 1
IPI00850377 **Mass:** 35975 **Score:** 186 **Queries matched:** 4
Tax_Id=10090 Gene_Symbol=- 36 kDa protein
IPI00850779 **Mass:** 36076 **Score:** 186 **Queries matched:** 4
Tax_Id=10090 Gene_Symbol=- 36 kDa protein
IPI00874682 **Mass:** 36038 **Score:** 186 **Queries matched:** 4
Tax_Id=10090 Gene_Symbol=- 36 kDa protein
IPI00874964 **Mass:** 36512 **Score:** 186 **Queries matched:** 4
Tax_Id=10090 Gene_Symbol=- 36 kDa protein

21. IPI00135106 **Mass:** 19584 **Score:** 113 **Queries matched:** 12 **emPAI:** 0.89
Tax_Id=10090 Gene_Symbol=Mobp Isoform 1 of Myelin-associated oligodendrocyte basic protein

Query	Observed	Mr(expt)	Mr(calc)	ppm	Miss	Score	Expect	Rank	Peptide
271	**449.7446**	**897.4746**	**897.4781**	**-3.80**	**1**	**10**	**7**	**1**	**R.GGSPTRAPR.F** 270
332	**498.7873**	**995.5600**	**995.5586**	**1.46**	**0**	**59**	**5.6e-005**	**1**	**K.SPLMPAKPR.S** 333
343	**503.3052**	**1004.5958**	**1004.5879**	**7.89**	**0**	**22**	**0.2**	**1**	**R.SPPRPAKPR.S** 342
349	**506.7819**	**1011.5492**	**1011.5535**	**-4.22**	**0**	**(49)**	**0.00066**	**1**	**K.SPLMPAKPR.S**
453	**557.8041**	**1113.5936**	**1113.5891**	**4.11**	**1**	**36**	**0.019**	**1**	**R.SSPLRGPGTSR.G** 452 454
491	**580.8288**	**1159.6430**	**1159.6462**	**-2.71**	**0**	**53**	**0.00038**	**1**	**K.HQPAASPVVVR.A** 492

22. IPI00114375 **Mass:** 62638 **Score:** 110 **Queries matched:** 1 **emPAI:** 0.05
Tax_Id=10090 Gene_Symbol=Dpysl2 Dihydropyrimidinase-related protein 2

Query	Observed	Mr(expt)	Mr(calc)	ppm	Miss	Score	Expect	Rank	Peptide
911	**956.0205**	**1910.0264**	**1910.0149**	**6.03**	**0**	**110**	**4.8e-010**	**1**	**R.FQLTDSQIYEVLSVIR.D**

23. IPI00132042 **Mass:** 39254 **Score:** 105 **Queries matched:** 1 **emPAI:** 0.08
Tax_Id=10090 Gene_Symbol=Pdhb Pyruvate dehydrogenase E1 component subunit beta, mitochondrial

Query	Observed	Mr(expt)	Mr(calc)	ppm	Miss	Score	Expect	Rank	Peptide
867	**874.4476**	**1746.8806**	**1746.8763**	**2.48**	**0**	**105**	**1.9e-009**	**1**	**R.IMEGPAFNFLDAPAVR.V**

24. IPI00138892 **Mass:** 15004 **Score:** 101 **Queries matched:** 2 **emPAI:** 0.23
Tax_Id=10090 Gene_Symbol=Uba52 Ubiquitin-60S ribosomal protein L40

Query	Observed	Mr(expt)	Mr(calc)	ppm	Miss	Score	Expect	Rank	Peptide
883	**894.4674**	**1786.9202**	**1786.9200**	**0.13**	**0**	**101**	**5.2e-009**	**1**	**K.TITLEVEPSDTIENVK.A** 884

Proteins matching the same set of peptides:
IPI00470152 **Mass:** 18282 **Score:** 101 **Queries matched:** 2
Tax_Id=10090 Gene_Symbol=Rps27a Ubiquitin-40S ribosomal protein S27a
IPI00895319 **Mass:** 22277 **Score:** 101 **Queries matched:** 2
Tax_Id=10090 Gene_Symbol=2810422J05Rik 22 kDa protein

IPI00895479 **Mass:** 27181 **Score:** 101 **Queries matched:** 2
Tax_Id=10090 Gene_Symbol=2810422J05Rik 27 kDa protein
IPI00918763 **Mass:** 11021 **Score:** 101 **Queries matched:** 2
Tax_Id=10090 Gene_Symbol=Uba52 Ubiqutin subunit 1
IPI00750889 **Mass:** 22822 **Score:** 101 **Queries matched:** 2
Tax_Id=10090 Gene_Symbol=Ubc Ubc protein
IPI00923013 **Mass:** 26603 **Score:** 101 **Queries matched:** 2
Tax_Id=10090 Gene_Symbol=Ubb Ubiquitin B
IPI00923037 **Mass:** 22578 **Score:** 101 **Queries matched:** 2
Tax_Id=10090 Gene_Symbol=Ubc Putative uncharacterized protein
IPI00139518 **Mass:** 34348 **Score:** 101 **Queries matched:** 2
Tax_Id=10090 Gene_Symbol=Ubb Polyubiquitin-B
IPI00755916 **Mass:** 82614 **Score:** 101 **Queries matched:** 2
Tax_Id=10090 Gene_Symbol=Ubc ubiquitin
IPI00969323 **Mass:** 82603 **Score:** 101 **Queries matched:** 2
Tax_Id=10090 Gene_Symbol=Ubc Polyubiquitin-C

---

25. IPI00121038 **Mass:** 368613 **Score:** 100 **Queries matched:** 3 **emPAI:** 0.02
Tax_Id=10090 Gene_Symbol=Vcan Isoform V0 of Versican core protein

Query	Observed	Mr(expt)	Mr(calc)	ppm	Miss	Score	Expect	Rank	Peptide
641	657.8627	1313.7108	1313.7092	1.28	0	15	2.3	1	R.LATVGELQAAWR.N
716	709.3745	1416.7344	1416.7283	4.37	1	45	0.0023	1	K.METSPPVKGSLSGK.V
926	656.6585	1966.9537	1966.9670	-6.79	0	87	1.2e-007	1	R.VSVPTHPDDVGDASLTMVK.L

Proteins matching the same set of peptides:
IPI00230441 **Mass:** 264594 **Score:** 100 **Queries matched:** 3
Tax_Id=10090 Gene_Symbol=Vcan Isoform V1 of Versican core protein
IPI00469172 **Mass:** 76209 **Score:** 100 **Queries matched:** 3
Tax_Id=10090 Gene_Symbol=Vcan versican core protein isoform 4
IPI00469565 **Mass:** 180325 **Score:** 100 **Queries matched:** 3
Tax_Id=10090 Gene_Symbol=Vcan versican core protein isoform 3
IPI00473932 **Mass:** 41850 **Score:** 100 **Queries matched:** 3
Tax_Id=10090 Gene_Symbol=Vcan versican core protein isoform 5
IPI00656277 **Mass:** 41758 **Score:** 100 **Queries matched:** 3
Tax_Id=10090 Gene_Symbol=Vcan Versican
IPI00875672 **Mass:** 368720 **Score:** 100 **Queries matched:** 3
Tax_Id=10090 Gene_Symbol=Vcan versican core protein isoform 1

---

26. IPI00323571 **Mass:** 35901 **Score:** 92 **Queries matched:** 7 **emPAI:** 0.30
Tax_Id=10090 Gene_Symbol=Apoe Apolipoprotein E

Query	Observed	Mr(expt)	Mr(calc)	ppm	Miss	Score	Expect	Rank	Peptide
315	484.7793	967.5440	967.5451	-1.06	0	23	0.19	1	R.LGPLVEQGR.Q
506	585.8336	1169.6526	1169.6516	0.86	1	68	9.6e-006	1	R.LGKEVQAAQAR.L 507
550	614.8275	1227.6404	1227.6320	6.90	1	40	0.0081	1	R.GRLEEVGNQAR.D
749	755.9121	1509.8096	1509.8012	5.62	1	10	4.9	1	R.TANLGAGAAQPLRDR.A
805	800.4014	1598.7882	1598.7787	5.94	0	47	0.0014	1	K.ELEEQLGPVAEETR.A
927	997.5323	1993.0500	1993.0480	1.03	0	18	0.84	1	K.IQASVATNPIITPVAQENQ.-

---

27. IPI00229525 **Score:** 88 **Queries matched:** 3
Tax_Id=10090 Gene_Symbol=Gnal Guanine nucleotide-binding protein G(olf) subunit alpha

Query	Observed	Mr(expt)	Mr(calc)	ppm	Miss	Score	Expect	Rank	Peptide
380	529.3142	1056.6138	1056.6179	-3.82	0	77	1.3e-006	1	R.LLLLGAGESGK.S 381
527	595.8279	1189.6412	1189.6165	20.8	1	0	84	9	R.MHLKQYELL.-

Proteins matching the same set of peptides:
IPI00272681 **Score:** 88 **Queries matched:** 3

---

28. IPI00118569 **Mass:** 44369 **Score:** 88 **Queries matched:** 2 **emPAI:** 0.07
Tax_Id=10090 Gene_Symbol=Gna13 Guanine nucleotide-binding protein subunit alpha-13

Query	Observed	Mr(expt)	Mr(calc)	ppm	Miss	Score	Expect	Rank	Peptide
380	529.3142	1056.6138	1056.6179	-3.82	0	77	1.3e-006	1	K.ILLLGAGESGK.S 381

Proteins matching the same set of peptides:
IPI00230191 **Mass:** 44238 **Score:** 88 **Queries matched:** 2
Tax_Id=10090 Gene_Symbol=Gna12 Guanine nucleotide-binding protein subunit alpha-12
IPI00649388 **Mass:** 20095 **Score:** 88 **Queries matched:** 2
Tax_Id=10090 Gene_Symbol=Gna13 Putative uncharacterized protein

---

29. IPI00129240 **Mass:** 55613 **Score:** 54 **Queries matched:** 1 **emPAI:** 0.06
Tax_Id=10090 Gene_Symbol=Vtn Vitronectin

Query	Observed	Mr(expt)	Mr(calc)	ppm	Miss	Score	Expect	Rank	Peptide
929	1001.0344	2000.0542	2000.0367	8.76	0	54	0.00022	1	K.LIQDVWGIEGPIDAAFTR.I

---

30. IPI00323357 **Mass:** 71055 **Score:** 52 **Queries matched:** 2 **emPAI:** 0.05
Tax_Id=10090 Gene_Symbol=Hspa8 Heat shock cognate 71 kDa protein

Query	Observed	Mr(expt)	Mr(calc)	ppm	Miss	Score	Expect	Rank	Peptide
551	614.8422	1227.6698	1227.6207	40.0	0	1	52	4	K.VEIIANDQGNR.T
816	808.9132	1615.8118	1615.7804	19.5	0	52	0.00038	1	K.SFYPEEVSSMVLTK.M

Proteins matching the same set of peptides:
IPI00886297 **Mass:** 68964 **Score:** 52 **Queries matched:** 2
Tax_Id=10090 Gene_Symbol=Hspa8 Hspa8 protein

---

31. IPI00131366 **Mass:** 60627 **Score:** 50 **Queries matched:** 1 **emPAI:** 0.05
Tax_Id=10090 Gene_Symbol=Krt6b Keratin, type II cytoskeletal 6B

Query	Observed	Mr(expt)	Mr(calc)	ppm	Miss	Score	Expect	Rank	Peptide
627	**651.8628**	**1301.7110**	**1301.7078**	**2.48**	**0**	**50**	**0.00059**	**1**	**R.SLDLDSIIAEVK.A**

Proteins matching the same set of peptides:

IPI00131368 **Mass:** 59641 **Score:** 50 **Queries matched:** 1
Tax_Id=10090 Gene_Symbol=Krt6a Keratin, type II cytoskeletal 6A
IPI00139301 **Mass:** 61957 **Score:** 50 **Queries matched:** 1
Tax_Id=10090 Gene_Symbol=Krt5 Keratin, type II cytoskeletal 5
IPI00221797 **Mass:** 59932 **Score:** 50 **Queries matched:** 1
Tax_Id=10090 Gene_Symbol=Krt75 Keratin, type II cytoskeletal 75
IPI00330480 **Mass:** 35315 **Score:** 50 **Queries matched:** 1
Tax_Id=10090 Gene_Symbol=- 35 kDa protein
IPI00470126 **Mass:** 60553 **Score:** 50 **Queries matched:** 1
Tax_Id=10090 Gene_Symbol=Krt5 Uncharacterized protein
IPI00762364 **Mass:** 50641 **Score:** 50 **Queries matched:** 1
Tax_Id=10090 Gene_Symbol=Krt6a Uncharacterized protein
IPI00785403 **Mass:** 59832 **Score:** 50 **Queries matched:** 1
Tax_Id=10090 Gene_Symbol=Krt6b keratin, type II cytoskeletal 6B

---

32. IPI00134344 **Mass:** 272022 **Score:** 48 **Queries matched:** 1 **emPAI:** 0.01
Tax_Id=10090 Gene_Symbol=Spnb3 Putative uncharacterized protein

Query	Observed	Mr(expt)	Mr(calc)	ppm	Miss	Score	Expect	Rank	Peptide
930	**1001.5367**	**2001.0588**	**2001.0531**	**2.88**	**0**	**48**	**0.00063**	**1**	**R.LVSQDNFGLELAAVEAAVR.K**

---

33. IPI00123313 **Mass:** 118931 **Score:** 47 **Queries matched:** 1 **emPAI:** 0.03
Tax_Id=10090 Gene_Symbol=Uba1 Ubiquitin-like modifier-activating enzyme 1

Query	Observed	Mr(expt)	Mr(calc)	ppm	Miss	Score	Expect	Rank	Peptide
825	**812.4624**	**1622.9102**	**1622.8992**	**6.84**	**0**	**47**	**0.00062**	**1**	**R.LAGTQPLEVLEAVQR.S**

---

34. IPI00113112 **Score:** 38 **Queries matched:** 1
Tax_Id=10090 Gene_Symbol=Rab3b Ras-related protein Rab-3B

Query	Observed	Mr(expt)	Mr(calc)	ppm	Miss	Score	Expect	Rank	Peptide
642	**658.8356**	**1315.6566**	**1315.6521**	**3.49**	**0**	**38**	**0.011**	**1**	**K.LQIWDTAGQER.Y**

Proteins matching the same set of peptides:

IPI00113127 **Score:** 38 **Queries matched:** 1
IPI00114560 **Score:** 38 **Queries matched:** 1
IPI00116688 **Score:** 38 **Queries matched:** 1
IPI00122965 **Score:** 38 **Queries matched:** 1
IPI00123180 **Score:** 38 **Queries matched:** 1
IPI00126042 **Score:** 38 **Queries matched:** 1
IPI00130118 **Score:** 38 **Queries matched:** 1
IPI00130489 **Score:** 38 **Queries matched:** 1
IPI00133706 **Score:** 38 **Queries matched:** 1
IPI00169699 **Score:** 38 **Queries matched:** 1
IPI00223332 **Score:** 38 **Queries matched:** 1
IPI00224319 **Score:** 38 **Queries matched:** 1
IPI00271059 **Score:** 38 **Queries matched:** 1
IPI00272230 **Score:** 38 **Queries matched:** 1
IPI00316495 **Score:** 38 **Queries matched:** 1
IPI00331128 **Score:** 38 **Queries matched:** 1
IPI00331205 **Score:** 38 **Queries matched:** 1
IPI00404357 **Score:** 38 **Queries matched:** 1
IPI00411115 **Score:** 38 **Queries matched:** 1
IPI00468209 **Score:** 38 **Queries matched:** 1
IPI00648888 **Score:** 38 **Queries matched:** 1
IPI00651876 **Score:** 38 **Queries matched:** 1
IPI00654255 **Score:** 38 **Queries matched:** 1
IPI00671283 **Score:** 38 **Queries matched:** 1
IPI00751303 **Score:** 38 **Queries matched:** 1
IPI00757464 **Score:** 38 **Queries matched:** 1
IPI00830932 **Score:** 38 **Queries matched:** 1
IPI00894915 **Score:** 38 **Queries matched:** 1
IPI00895100 **Score:** 38 **Queries matched:** 1
IPI00944081 **Score:** 38 **Queries matched:** 1

---

35. IPI00116697 **Score:** 38 **Queries matched:** 1
Tax_Id=10090 Gene_Symbol=Rab6 Isoform 1 of Ras-related protein Rab-6A

Query	Observed	Mr(expt)	Mr(calc)	ppm	Miss	Score	Expect	Rank	Peptide
642	658.8356	1315.6566	1315.6521	3.49	0	38	0.011	1	R.LQLWDTAGQER.F

Proteins matching the same set of peptides:

IPI00221836 **Score:** 38 **Queries matched:** 1
IPI00230011 **Score:** 38 **Queries matched:** 1
IPI00378145 **Score:** 38 **Queries matched:** 1
IPI00757748 **Score:** 38 **Queries matched:** 1

---

36. IPI00136271 **Score:** 38 **Queries matched:** 1
Tax_Id=10090 Gene_Symbol=Rab33b Putative uncharacterized protein

Query	Observed	Mr(expt)	Mr(calc)	ppm	Miss	Score	Expect	Rank	Peptide
642	658.8356	1315.6566	1315.6521	3.49	0	38	0.011	1	K.IQLWDTAGQER.F

Proteins matching the same set of peptides:

IPI00890284 **Score:** 38 **Queries matched:** 1

---

37. IPI00170032 **Mass:** 24588 **Score:** 38 **Queries matched:** 1 **emPAI:** 0.14
Tax_Id=10090 Gene_Symbol=Rab15 Ras-related protein Rab-15

Query	Observed	Mr(expt)	Mr(calc)	ppm	Miss	Score	Expect	Rank	Peptide
642	658.8356	1315.6566	1315.6521	3.49	0	38	0.011	1	R.IQIWDTAGQER.Y

Proteins matching the same set of peptides:
IPI00622236 **Mass:** 19558 **Score:** 38 **Queries matched:** 1
Tax_Id=10090 Gene_Symbol=Rab15 Rab15 protein
IPI00880661 **Mass:** 21411 **Score:** 38 **Queries matched:** 1
Tax_Id=10090 Gene_Symbol=Rab15 Uncharacterized protein
IPI00880893 **Mass:** 19333 **Score:** 38 **Queries matched:** 1
Tax_Id=10090 Gene_Symbol=Rab15 Uncharacterized protein
IPI00881140 **Mass:** 13439 **Score:** 38 **Queries matched:** 1
Tax_Id=10090 Gene_Symbol=Rab15 Uncharacterized protein
IPI00881258 **Mass:** 16230 **Score:** 38 **Queries matched:** 1
Tax_Id=10090 Gene_Symbol=Rab15 Uncharacterized protein
IPI00884469 **Mass:** 24602 **Score:** 38 **Queries matched:** 1
Tax_Id=10090 Gene_Symbol=Rab15 ras-related protein Rab-15

---

38. IPI00656325 **Mass:** 83131 **Score:** 37 **Queries matched:** 2 **emPAI:** 0.04
Tax_Id=10090 Gene_Symbol=Nsf Vesicle-fusing ATPase

Query	Observed	Mr(expt)	Mr(calc)	ppm	Miss	Score	Expect	Rank	Peptide
615	**646.3392**	**1290.6638**	**1290.6568**	**5.46**	**0**	**37**	**0.015**	**1**	**K.NFSGAELEGLVR.A**
738	**729.3986**	**1456.7826**	**1456.7773**	**3.65**	**0**	**17**	**1.1**	**1**	**R.VLDDGELLVQQTK.N**

Proteins matching the same set of peptides:
IPI00876255 **Mass:** 83174 **Score:** 37 **Queries matched:** 2
Tax_Id=10090 Gene_Symbol=Nsf 83 kDa protein

Mascot: http://www.matrixscience.com/

Mascot Search Results

User	: Dominic
Email	: dominic.kurian@bbsrc.ac.uk
Search title	: IPI Mouse_15-02-2010
MS data file	: C:\Program Files\Matrix Science\Mascot Daemon\mgf\32 10755\mascot_daemon_merge.mgf
Database	: IPI_mouse MOUSE_v3_79 (54943 sequences; 24917557 residues)
Timestamp	: 15 Feb 2011 at 11:28:31 GMT
Enzyme	: Trypsin
Fixed modifications	: Carbamidomethyl (C)
Variable modifications	: Oxidation (M)
Mass values	: Monoisotopic
Protein Mass	: Unrestricted
Peptide Mass Tolerance	: ± 50 ppm
Fragment Mass Tolerance	: ± 0.2 Da
Max Missed Cleavages	: 1
Instrument type	: ESI-QUAD-TOF
Number of queries	: 1078
Protein hits	: IPI00110850 Tax_Id=10090 Gene_Symbol=Actb Actin, cytoplasmic 1
	IPI00136929 Tax_Id=10090 Gene_Symbol=Actg1 Gamma actin-like protein
	IPI00169463 Tax_Id=10090 Gene_Symbol=Tubb2c Tubulin beta-2C chain
	IPI00109073 Tax_Id=10090 Gene_Symbol=Tubb4 Tubulin beta-4 chain
	IPI00338039 Tax_Id=10090 Gene_Symbol=Tubb2a Tubulin beta-2A chain
	IPI00117352 Tax_Id=10090 Gene_Symbol=Tubb5 Tubulin beta-5 chain
	IPI00221528 Tax_Id=10090 Gene_Symbol=Actbl2 Beta-actin-like protein 2
	IPI00117348 Tax_Id=10090 Gene_Symbol=Tuba1b Tubulin alpha-1B chain
	IPI00110753 Tax_Id=10090 Gene_Symbol=Tuba1a Tubulin alpha-1A chain
	IPI00112251 Tax_Id=10090 Gene_Symbol=Tubb3 Tubulin beta-3 chain
	IPI00122048 Tax_Id=10090 Gene_Symbol=Atp1a3 Sodium/potassium-transporting ATPase subunit alpha-3
	IPI00420725 Tax_Id=10090 Gene_Symbol=Camk2a Uncharacterized protein
	IPI00122928 Tax_Id=10090 Gene_Symbol=Tubb6 Tubulin beta-6 chain
	IPI00115546 Tax_Id=10090 Gene_Symbol=Gnao1 Isoform Alpha-2 of Guanine nucleotide-binding protein G(o) subunit
	IPI00117350 Tax_Id=10090 Gene_Symbol=Tuba4a Tubulin alpha-4A chain
	IPI00120793 Tax_Id=10090 Gene_Symbol=Prnp Major prion protein
	IPI00135284 Tax_Id=10090 Gene_Symbol=Gm16374 Glyceraldehyde-3-phosphate dehydrogenase
	IPI00123176 Tax_Id=10090 Gene_Symbol=Gm6316 Glyceraldehyde-3-phosphate dehydrogenase
	IPI00474502 Tax_Id=10090 Gene_Symbol=Camk2b Calcium/calmodulin-dependent protein kinase type II subunit beta
	IPI00119063 Tax_Id=10090 Gene_Symbol=Lrp1 Prolow-density lipoprotein receptor-related protein 1
	IPI00136703 Tax_Id=10090 Gene_Symbol=Ckb Creatine kinase B-type
	IPI00319602 Tax_Id=10090 Gene_Symbol=Cnp Isoform CNPII of 2',3'-cyclic-nucleotide 3'-phosphodiesterase
	IPI00138892 Tax_Id=10090 Gene_Symbol=Uba52 Ubiquitin-60S ribosomal protein L40
	IPI00323357 Tax_Id=10090 Gene_Symbol=Hspa8 Heat shock cognate 71 kDa protein
	IPI00348094 Tax_Id=10090 Gene_Symbol=Tubb1 Tubulin, beta 1
	IPI00608020 Tax_Id=10090 Gene_Symbol=Ftl1 ferritin light chain 1
	IPI00136372 Tax_Id=10090 Gene_Symbol=Syn1 Isoform Ib of Synapsin-1
	IPI00555023 Tax_Id=10090 Gene_Symbol=Gstp1 Glutathione S-transferase P 1
	IPI00114375 Tax_Id=10090 Gene_Symbol=Dpysl2 Dihydropyrimidinase-related protein 2
	IPI00652902 Tax_Id=10090 Gene_Symbol=Gnai2 Putative uncharacterized protein
	IPI00114642 Tax_Id=10090 Gene_Symbol=Hist1h2bn;Hist1h2bl;Hist1h2bj;Hist1h2bf Histone H2B type 1-F/J/L
	IPI00118569 Tax_Id=10090 Gene_Symbol=Gna13 Guanine nucleotide-binding protein subunit alpha-13
	IPI00310091 Tax_Id=10090 Gene_Symbol=Ppp2r1a Serine/threonine-protein phosphatase 2A 65 kDa regulatory subuni
	IPI00130280 Tax_Id=10090 Gene_Symbol=Atp5a1 ATP synthase subunit alpha, mitochondrial
	IPI00625729 Tax_Id=10090 Gene_Symbol=Krt1 Keratin, type II cytoskeletal 1
	IPI00462140 Tax_Id=10090 Gene_Symbol=Krt77 Keratin, type II cytoskeletal 1b
	IPI00408378 Tax_Id=10090 Gene_Symbol=Ywhaq Isoform 1 of 14-3-3 protein theta
	IPI00119067 Tax_Id=10090 Gene_Symbol=Tppp Tubulin polymerization-promoting protein
	IPI00323571 Tax_Id=10090 Gene_Symbol=Apoe Apolipoprotein E
	IPI00272878 Tax_Id=10090 Gene_Symbol=Dnm1 Isoform 1 of Dynamin-1
	IPI00230145 Tax_Id=10090 Gene_Symbol=Fth1 Ferritin heavy chain
	IPI00123313 Tax_Id=10090 Gene_Symbol=Uba1 Ubiquitin-like modifier-activating enzyme 1
	IPI00131366 Tax_Id=10090 Gene_Symbol=Krt6b Keratin, type II cytoskeletal 6B
	IPI00462072 Tax_Id=10090 Gene_Symbol=Eno1;Gm5506 Alpha-enolase
	IPI00266188 Tax_Id=10090 Gene_Symbol=Cfl2 Cofilin-2
	IPI00230680 Tax_Id=10090 Gene_Symbol=Atp4a Potassium-transporting ATPase alpha chain 1
	IPI00268145 Tax_Id=10090 Gene_Symbol=Atp12a Potassium-transporting ATPase alpha chain 2
	IPI00222632 Tax_Id=10090 Gene_Symbol=Rab31 ras-related protein Rab-31
	IPI00755181 Tax_Id=10090 Gene_Symbol=Krt10 keratin, type I cytoskeletal 10
	IPI00134492 Tax_Id=10090 Gene_Symbol=Syn2 Isoform IIb of Synapsin-2
	IPI00415402 Tax_Id=10090 Gene_Symbol=Stxbp1 Isoform 1 of Syntaxin-binding protein 1
	IPI00656325 Tax_Id=10090 Gene_Symbol=Nsf Vesicle-fusing ATPase
	IPI00135106 Tax_Id=10090 Gene_Symbol=Mobp Isoform 1 of Myelin-associated oligodendrocyte basic protein

	IPI_mouse	Decoy	False discovery rate
Peptide matches above identity threshold	145	0	0.00 %
Peptide matches above homology or identity threshold	161	0	0.00 %

Select Summary Report

Format As | Select Summary (protein hits) | Help

Significance threshold p< 0.01 | Max. number of hits AUTO

Standard scoring | MudPIT scoring | Ions score or expect cut-off 0 | Show sub-sets 0

Show pop-ups | Suppress pop-ups | Sort unassigned Decreasing Score | Require bold red

1. IPI00110850 **Mass:** 42052 **Score:** 818 **Queries matched:** 24 **emPAI:** 1.88
Tax_Id=10090 Gene_Symbol=Actb Actin, cytoplasmic 1

Query	Observed	Mr(expt)	Mr(calc)	ppm	Miss	Score	Expect	Rank	Peptide

Query	Observed	Mr(expt)	Mr(calc)	ppm	Miss	Score	Expect	Rank	Peptide
279	488.7281	975.4416	975.4410	0.65	0	22	0.31	1	K.AGFAGDDAPR.A
403	566.7664	1131.5182	1131.5197	-1.24	0	39	0.0064	1	R.GYSFTTTAER.E
437	581.3213	1160.6280	1160.6111	14.6	0	44	0.0035	1	K.EITALAPSTMK.I 436
450	586.2927	1170.5708	1170.5638	6.02	0	41	0.0053	1	R.HQGVMVGMGQK.D 451
456	589.3190	1176.6234	1176.6060	14.8	0	(5)	21	5	K.EITALAPSTMK.I
482	599.8567	1197.6988	1197.6982	0.52	0	60	3.5e-005	1	R.AVFPSIVGRPR.H 483
488	602.2874	1202.5602	1202.5536	5.50	0	(34)	0.027	1	R.HQGVMVGMGQK.D
665	677.8166	1353.6186	1353.6161	1.91	1	74	2.2e-006	1	K.DSYVGDEAQSKR.G 664
763	774.9140	1547.8134	1547.8051	5.39	1	91	5.1e-008	1	R.MQKEITALAPSTMK.I
854	812.4282	1622.8418	1622.8338	4.98	1	52	0.00033	1	R.LDLAGRDLTDYLMK.I
923	895.9495	1789.8844	1789.8846	-0.10	0	95	1.9e-008	1	K.SYELPDGQVITIGNER.F 922 924 925
972	652.0197	1953.0373	1953.0571	-10.15	0	68	7.4e-006	1	R.VAPEEHPVLLTEAPLNPK.A 973
1011	1108.0479	2214.0812	2214.0627	8.39	0	66	1.3e-005	1	K.DLYANTVLSGGTTMYPGIADR.M
1068	1061.8734	3182.5984	3182.6071	-2.73	0	159	3.8e-015	1	R.TTGIVMDSGDGVTHTVPIYEGYALPHAILR.L 1066 1067

Proteins matching the same set of peptides:
IPI00874482 **Mass:** 42108 **Score:** 818 **Queries matched:** 24
Tax_Id=10090 Gene_Symbol=Actg1 Actin, cytoplasmic 2

2. IPI00136929 **Mass:** 44029 **Score:** 726 **Queries matched:** 18 **emPAI:** 1.21
Tax_Id=10090 Gene_Symbol=Actg1 Gamma actin-like protein

Query	Observed	Mr(expt)	Mr(calc)	ppm	Miss	Score	Expect	Rank	Peptide
403	566.7664	1131.5182	1131.5197	-1.24	0	39	0.0064	1	R.GYSFTTTAER.E
437	581.3213	1160.6280	1160.6111	14.6	0	44	0.0035	1	K.EITALAPSTMK.I 436
456	589.3190	1176.6234	1176.6060	14.8	0	(5)	21	5	K.EITALAPSTMK.I
665	677.8166	1353.6186	1353.6161	1.91	1	74	2.2e-006	1	K.DSYVGDEAQSKR.G 664
763	774.9140	1547.8134	1547.8051	5.39	1	91	5.1e-008	1	R.MQKEITALAPSTMK.I
854	812.4282	1622.8418	1622.8338	4.98	1	52	0.00033	1	R.LDLAGRDLTDYLMK.I
923	895.9495	1789.8844	1789.8846	-0.10	0	95	1.9e-008	1	K.SYELPDGQVITIGNER.F 922 924 925
972	652.0197	1953.0373	1953.0571	-10.15	0	68	7.4e-006	1	R.VAPEEHPVLLTEAPLNPK.R 973
1011	1108.0479	2214.0812	2214.0627	8.39	0	66	1.3e-005	1	K.DLYANTVLSGGTTMYPGLADR.M
1068	1061.8734	3182.5984	3182.6071	-2.73	0	159	3.8e-015	1	R.TTGIVMDSGDGVTHTVPIYEGYALPHAILR.L 1066 1067

3. IPI00169463 **Mass:** 50255 **Score:** 656 **Queries matched:** 29 **emPAI:** 1.94
Tax_Id=10090 Gene_Symbol=Tubb2c Tubulin beta-2C chain

Query	Observed	Mr(expt)	Mr(calc)	ppm	Miss	Score	Expect	Rank	Peptide
339	539.2715	1076.5284	1076.5250	3.17	1	35	0.026	1	K.IREEYPDR.I 338 340
409	572.3189	1142.6232	1142.6270	-3.31	0	(64)	3.3e-005	1	K.LAVNMVPFPR.L 410
431	580.3211	1158.6276	1158.6219	4.92	0	83	4.5e-007	1	K.LAVNMVPFPR.L
521	615.3052	1228.5958	1228.5910	3.92	0	68	1.2e-005	1	R.ISEQFTAMFR.R
541	623.2966	1244.5786	1244.5860	-5.87	0	(6)	14	1	R.ISEQFTAMFR.R
632	660.3535	1318.6924	1318.6955	-2.32	0	72	5e-006	1	R.IMNTFSVVPSPK.V 633
643	664.8295	1327.6444	1327.6408	2.74	0	70	6.3e-006	1	R.INVYYNEATGGK.Y
648	668.3517	1334.6888	1334.6904	-1.19	0	(50)	0.00078	1	R.IMNTFSVVPSPK.V
708	723.8540	1445.6934	1445.6820	7.90	0	59	8.6e-005	1	K.EVDEQMLNVQNK.N
837	801.4127	1600.8108	1600.8131	-1.38	0	(28)	0.098	1	R.AVLVDLEPGTMDSVR.S
844	809.4111	1616.8076	1616.8080	-0.21	0	39	0.0077	1	R.AVLVDLEPGTMDSVR.S
849	540.9459	1619.8159	1619.8283	-7.65	0	47	0.0014	1	R.LHFFMPGFAPLTSR.G 850
875	846.4407	1690.8668	1690.8600	4.05	0	72	4.3e-006	1	R.ALTVPELTQQMFDAK.N 874
878	848.9237	1695.8328	1695.8257	4.24	0	50	0.00065	1	K.NSSYFVEWIPNNVK.T 877
887	854.4349	1706.8552	1706.8549	0.19	0	(53)	0.00036	2	R.ALTVPELTQQMFDAK.N 889 890
949	929.4739	1856.9332	1856.9342	-0.52	0	46	0.0016	1	K.MSATFIGNSTAIQELFK.R
992	672.0167	2013.0283	2013.0353	-3.50	1	134	1.9e-012	1	K.MSATFIGNSTAIQELFKR.I
1048	933.4539	2797.3399	2797.3361	1.34	0	104	1.4e-009	1	R.SGPFGQIFRPDNFVFGQSGAGNNWAK.G 1047 1049

4. IPI00109073 **Mass:** 50010 **Score:** 638 **Queries matched:** 25 **emPAI:** 1.60
Tax_Id=10090 Gene_Symbol=Tubb4 Tubulin beta-4 chain

Query	Observed	Mr(expt)	Mr(calc)	ppm	Miss	Score	Expect	Rank	Peptide
409	572.3189	1142.6232	1142.6270	-3.31	0	(64)	3.3e-005	1	K.LAVNMVPFPR.L 410
431	580.3211	1158.6276	1158.6219	4.92	0	83	4.5e-007	1	K.LAVNMVPFPR.L
521	615.3052	1228.5958	1228.5910	3.92	0	68	1.2e-005	1	R.ISEQFTAMFR.R
541	623.2966	1244.5786	1244.5860	-5.87	0	(6)	14	1	R.ISEQFTAMFR.R
632	660.3535	1318.6924	1318.6955	-2.32	0	72	5e-006	1	R.IMNTFSVVPSPK.V 633
648	668.3517	1334.6888	1334.6904	-1.19	0	(50)	0.00078	1	R.IMNTFSVVPSPK.V
837	801.4127	1600.8108	1600.8131	-1.38	0	(28)	0.098	1	R.AVLVDLEPGTMDSVR.S
844	809.4111	1616.8076	1616.8080	-0.21	0	39	0.0077	1	R.AVLVDLEPGTMDSVR.S
849	540.9459	1619.8159	1619.8283	-7.65	0	47	0.0014	1	R.LHFFMPGFAPLTSR.G 850
875	846.4407	1690.8668	1690.8600	4.05	0	72	4.3e-006	1	R.ALTVPELTQQMFDAK.N 874
878	848.9237	1695.8328	1695.8257	4.24	0	50	0.00065	1	K.NSSYFVEWIPNNVK.T 877
887	854.4349	1706.8552	1706.8549	0.19	0	(53)	0.00036	2	R.ALTVPELTQQMFDAK.N 889 890
937	915.4451	1828.8756	1828.8744	0.69	0	114	2.2e-010	1	R.INVYYNEATGGNYVPR.A
949	929.4739	1856.9332	1856.9342	-0.52	0	43	0.0029	2	K.MAATFIGNSTAIQELFK.R
992	672.0167	2013.0283	2013.0353	-3.50	1	116	1.3e-010	3	K.MAATFIGNSTAIQELFKR.I
1048	933.4539	2797.3399	2797.3361	1.34	0	104	1.4e-009	1	R.SGPFGQIFRPDNFVFGQSGAGNNWAK.G 1047 1049

5. IPI00338039 **Mass:** 50274 **Score:** 600 **Queries matched:** 30 **emPAI:** 1.76
Tax_Id=10090 Gene_Symbol=Tubb2a Tubulin beta-2A chain

Query	Observed	Mr(expt)	Mr(calc)	ppm	Miss	Score	Expect	Rank	Peptide
339	539.2715	1076.5284	1076.5250	3.17	1	35	0.026	1	K.IREEYPDR.I 338 340
409	572.3189	1142.6232	1142.6270	-3.31	0	(64)	3.3e-005	1	K.LAVNMVPFPR.L 410
431	580.3211	1158.6276	1158.6219	4.92	0	83	4.5e-007	1	K.LAVNMVPFPR.L
521	615.3052	1228.5958	1228.5910	3.92	0	68	1.2e-005	1	R.ISEQFTAMFR.R
541	623.2966	1244.5786	1244.5860	-5.87	0	(6)	14	1	R.ISEQFTAMFR.R
660	676.3409	1350.6672	1350.6676	-0.24	0	63	3.8e-005	1	R.IMNTFSVMPSPK.V 659
708	723.8540	1445.6934	1445.6820	7.90	0	59	8.6e-005	1	K.EVDEQMLNVQNK.N
841	808.4230	1614.8314	1614.8287	1.70	0	77	1.5e-006	1	R.AILVDLEPGTMDSVR.S 842
849	540.9459	1619.8159	1619.8283	-7.65	0	47	0.0014	1	R.LHFFMPGFAPLTSR.G 850

858	816.4194	1630.8242	1630.8236	0.38	0	(58)	0.00011	1	R.AILVDLEPGTMDSVR.S 859
878	848.9237	1695.8328	1695.8257	4.24	0	50	0.00065	1	K.NSSYFVEWIPNNVK.T 877
887	854.4349	1706.8552	1706.8549	0.19	0	66	1.6e-005	1	R.ALTVPELTQQMFDSK.N 889 890
896	862.4366	1722.8586	1722.8498	5.11	0	(11)	4.4	1	R.ALTVPELTQQMFDSK.N
949	929.4739	1856.9332	1856.9342	-0.52	0	46	0.0016	1	K.MSATFIGNSTAIQELFK.R
958	624.3088	1869.9046	1869.9373	-17.50	1	33	0.028	1	R.INVYYNEAAGNKYVPR.A
992	672.0167	2013.0283	2013.0353	-3.50	1	134	1.9e-012	1	K.MSATFIGNSTAIQELFKR.I
1048	933.4539	2797.3399	2797.3361	1.34	0	104	1.4e-009	1	R.SGPFGQIFRPDNFVFGQSGAGNNWAK.G 1047 1049
1065	776.3421	3101.3393	3101.4003	-19.66	0	29	0.022	1	K.FWEVISDEHGIDPTGSYHGDSDLQLER.I

---

6. IPI00117352 **Mass:** 50095 **Score:** 553 **Queries matched:** 25 **emPAI:** 1.60

Tax_Id=10090 Gene_Symbol=Tubb5 Tubulin beta-5 chain

Query	Observed	Mr(expt)	Mr(calc)	ppm	Miss	Score	Expect	Rank	Peptide
339	539.2715	1076.5284	1076.5250	3.17	1	35	0.026	1	K.IREEYPDR.I 338 340
409	572.3189	1142.6232	1142.6270	-3.31	0	(64)	3.3e-005	1	K.LAVNMVPFPR.L 410
431	580.3211	1158.6276	1158.6219	4.92	0	83	4.5e-007	1	K.LAVNMVPFPR.L
521	615.3052	1228.5958	1228.5910	3.92	0	68	1.2e-005	1	R.ISEQFTAMFR.R
541	623.2966	1244.5786	1244.5860	-5.87	0	(6)	14	1	R.ISEQFTAMFR.R
608	651.3157	1300.6168	1300.6299	-10.05	0	52	0.00041	1	R.ISVYYNEATGGK.Y
632	660.3535	1318.6924	1318.6955	-2.32	0	72	5e-006	1	R.IMNTFSVVPSPK.V 633
648	668.3517	1334.6888	1334.6904	-1.19	0	(50)	0.00078	1	R.IMNTFSVVPSPK.V
708	723.8540	1445.6934	1445.6820	7.90	0	59	8.6e-005	1	K.EVDEQMLNVQNK.N
841	808.4230	1614.8314	1614.8287	1.70	0	77	1.5e-006	1	R.AILVDLEPGTMDSVR.S 842
849	540.9459	1619.8159	1619.8283	-7.65	0	47	0.0014	1	R.LHFFMPGFAPLTSR.G 850
858	816.4194	1630.8242	1630.8236	0.38	0	(58)	0.00011	1	R.AILVDLEPGTMDSVR.S 859
864	830.4438	1658.8730	1658.8779	-8.98	0	72	3.8e-006	1	R.ALTVPELTQQVFDAK.N
878	848.9237	1695.8328	1695.8257	4.24	0	50	0.00065	1	K.NSSYFVEWIPNNVK.T 877
1048	933.4539	2797.3399	2797.3361	1.34	0	104	1.4e-009	1	R.SGPFGQIFRPDNFVFGQSGAGNNWAK.G 1047 1049

---

7. IPI00221528 **Mass:** 42319 **Score:** 498 **Queries matched:** 13 **emPAI:** 0.83

Tax_Id=10090 Gene_Symbol=Actbl2 Beta-actin-like protein 2

Query	Observed	Mr(expt)	Mr(calc)	ppm	Miss	Score	Expect	Rank	Peptide
450	586.2927	1170.5708	1170.5638	6.02	0	41	0.0053	1	R.HQGVMVGMGQK.D 451
488	602.2874	1202.5602	1202.5536	5.50	0	(34)	0.027	1	R.HQGVMVGMGQK.D
854	812.4282	1622.8418	1622.8338	4.98	1	52	0.00033	1	R.LDLAGRDLTDYLMK.I
923	895.9495	1789.8844	1789.8846	-0.10	0	95	1.9e-008	1	R.SYELPDGQVITIGNER.F 922 924 925
972	652.0197	1953.0373	1953.0571	-10.15	0	50	0.00051	2	R.VAPDEHPILLTEAPLNPK.I 973
1068	1061.8734	3182.5984	3182.6071	-2.73	0	159	3.8e-015	1	R.TTGIVMDSGDGVTHTVPIYEGYALPHAILR.L 1066 1067

---

8. IPI00117348 **Mass:** 50804 **Score:** 427 **Queries matched:** 16 **emPAI:** 0.55

Tax_Id=10090 Gene_Symbol=Tuba1b Tubulin alpha-1B chain

Query	Observed	Mr(expt)	Mr(calc)	ppm	Miss	Score	Expect	Rank	Peptide
243	455.2580	908.5014	908.4967	5.21	1	2	33	2	R.LSVDYGKK.S
294	508.2930	1014.5714	1014.5709	0.50	0	26	0.19	1	K.DVNAAIATIK.T
736	744.4408	1486.8670	1486.8719	-3.24	0	18	0.45	1	R.LISQIVSSITASLR.F
883	851.4699	1700.9252	1700.8985	15.7	0	80	4.4e-007	1	R.AVFVDLEPTVIDEVR.T 882
893	859.9458	1717.8770	1717.8747	1.36	0	67	1.3e-005	1	R.NLDIERPTYTNLNR.L 894
934	912.9977	1823.9808	1823.9782	1.47	0	29	0.052	1	K.VGINYQPPTVVPGGDLAK.V 935
990	1004.4575	2006.9004	2006.8858	7.30	0	89	5.8e-008	1	K.TIGGGDDSFNTFFSETGAGK.H
1028	1205.1035	2408.1924	2408.2012	-3.65	0	125	1.4e-011	1	R.FDGALNVDLTEFQTNLVPYPR.I
1033	805.7347	2414.1823	2414.1978	-6.44	1	78	8e-007	1	R.QLFHPEQLITGKEDAANNYAR.G 1030 1031 1032 1034

Proteins matching the same set of peptides:

IPI00403810 **Mass:** 50562 **Score:** 427 **Queries matched:** 16

Tax_Id=10090 Gene_Symbol=Tuba1c Tubulin alpha-1C chain

---

9. IPI00110753 **Mass:** 50788 **Score:** 427 **Queries matched:** 16 **emPAI:** 0.55

Tax_Id=10090 Gene_Symbol=Tuba1a Tubulin alpha-1A chain

Query	Observed	Mr(expt)	Mr(calc)	ppm	Miss	Score	Expect	Rank	Peptide
243	455.2580	908.5014	908.4967	5.21	1	2	33	2	R.LSVDYGKK.S
294	508.2930	1014.5714	1014.5709	0.50	0	26	0.19	1	K.DVNAAIATIK.T
713	729.4382	1456.8618	1456.8613	0.37	0	8	2.8	1	R.LIGQIVSSITASLR.F
883	851.4699	1700.9252	1700.8985	15.7	0	80	4.4e-007	1	R.AVFVDLEPTVIDEVR.T 882
893	859.9458	1717.8770	1717.8747	1.36	0	67	1.3e-005	1	R.NLDIERPTYTNLNR.L 894
934	912.9977	1823.9808	1823.9782	1.47	0	29	0.052	1	K.VGINYQPPTVVPGGDLAK.V 935
990	1004.4575	2006.9004	2006.8858	7.30	0	89	5.8e-008	1	K.TIGGGDDSFNTFFSETGAGK.H
1028	1205.1035	2408.1924	2408.2012	-3.65	0	125	1.4e-011	1	R.FDGALNVDLTEFQTNLVPYPR.I
1033	805.7347	2414.1823	2414.1978	-6.44	1	78	8e-007	1	R.QLFHPEQLITGKEDAANNYAR.G 1030 1031 1032 1034

---

10. IPI00112251 **Mass:** 50842 **Score:** 426 **Queries matched:** 21 **emPAI:** 0.87

Tax_Id=10090 Gene_Symbol=Tubb3 Tubulin beta-3 chain

Query	Observed	Mr(expt)	Mr(calc)	ppm	Miss	Score	Expect	Rank	Peptide
332	532.2687	1062.5228	1062.5094	12.7	1	25	0.26	1	K.VREEYPDR.I
409	572.3189	1142.6232	1142.6270	-3.31	0	(64)	3.3e-005	1	K.LAVNMVPFPR.L 410
431	580.3211	1158.6276	1158.6219	4.92	0	83	4.5e-007	1	K.LAVNMVPFPR.L
521	615.3052	1228.5958	1228.5910	3.92	0	68	1.2e-005	1	R.ISEQFTAMFR.R
541	623.2966	1244.5786	1244.5860	-5.87	0	(6)	14	1	R.ISEQFTAMFR.R
632	660.3535	1318.6924	1318.6955	-2.32	0	72	5e-006	1	R.IMNTFSVVPSPK.V 633
648	668.3517	1334.6888	1334.6904	-1.19	0	(50)	0.00078	1	R.IMNTFSVVPSPK.V
841	808.4230	1614.8314	1614.8287	1.70	0	77	1.5e-006	1	R.AILVDLEPGTMDSVR.S 842
850	810.9166	1619.8186	1619.8283	-5.94	0	19	0.83	2	R.LHFFMPGFAPLTAR.G
858	816.4194	1630.8242	1630.8236	0.38	0	(58)	0.00011	1	R.AILVDLEPGTMDSVR.S 859
875	846.4407	1690.8668	1690.8600	4.05	0	72	4.3e-006	1	R.ALTVPELTQQMFDAK.N 874
878	848.9237	1695.8328	1695.8257	4.24	0	50	0.00065	1	K.NSSYFVEWIPNNVK.V 877
887	854.4349	1706.8552	1706.8549	0.19	0	(53)	0.00036	2	R.ALTVPELTQQMFDAK.N 889 890

11. IPI00122048 **Mass:** 113045 **Score:** 397 **Queries matched:** 12 **emPAI:** 0.22
Tax_Id=10090 Gene_Symbol=Atp1a3 Sodium/potassium-transporting ATPase subunit alpha-3

Query	Observed	Mr(expt)	Mr(calc)	ppm	Miss	Score	Expect	Rank	Peptide
327	528.2854	1054.5562	1054.5520	4.07	1	18	1.1	1	K.GGDRVPADLR.I
529	618.8585	1235.7024	1235.6986	3.11	0	54	0.00015	1	R.LNIPVSQVNPR.D 530
573	634.8594	1267.7042	1267.7071	-2.22	0	40	0.0054	1	K.NMVPQQALVIR.E 574
685	691.3808	1380.7470	1380.7435	2.55	0	57	0.00012	1	K.VIMVTGDHPITAK.A
931	906.4794	1810.9442	1810.9425	0.96	0	91	4.8e-008	1	R.QGAIVAVTGDGVNDSPALK.K
938	915.4692	1828.9238	1828.9167	3.93	0	116	1.6e-010	1	K.GVGIISEGNETVEDIAAR.L
970	647.3470	1939.0192	1939.0375	-9.43	1	33	0.025	1	R.QGAIVAVTGDGVNDSPALKK.A
1039	1232.6124	2463.2102	2463.1951	6.13	0	133	2.6e-012	1	K.QAADMILLDDNFASIVTGVEEGR.L
1057	956.1147	2865.3223	2865.3490	-9.34	1	64	1.1e-005	1	K.EQPLDEEMKEAFQNAYLELGGLGER.V 1058

Proteins matching the same set of peptides:
IPI00752412 **Mass:** 117548 **Score:** 397 **Queries matched:** 12
Tax_Id=10090 Gene_Symbol=Atp1a3 ATPase, Na+/K+ transporting, alpha 3 polypeptide, isoform CRA_c

12. IPI00420725 **Mass:** 55882 **Score:** 371 **Queries matched:** 16 **emPAI:** 0.67
Tax_Id=10090 Gene_Symbol=Camk2a Uncharacterized protein

Query	Observed	Mr(expt)	Mr(calc)	ppm	Miss	Score	Expect	Rank	Peptide
323	525.2839	1048.5532	1048.5553	-1.94	0	22	0.58	1	K.VLAGQEYAAK.I
330	530.3022	1058.5898	1058.5906	-0.73	1	51	0.00061	1	K.MLTINPSKR.I 331
417	574.3328	1146.6510	1146.6431	6.96	0	62	3.2e-005	1	K.GAILTTMLATR.N
568	631.3073	1260.6000	1260.5927	5.79	0	62	4.9e-005	1	R.FYFENLWSR.N 566
614	652.3557	1302.6968	1302.6932	2.81	0	71	5e-006	1	R.ITQYLDAGGIPR.T 613
653	670.8925	1339.7704	1339.7711	-0.47	0	63	2e-005	1	R.DLKPENLLLASK.L 654
860	816.8933	1631.7720	1631.7719	0.10	0	69	7.9e-006	1	R.FTEEYQLFEELGK.G
996	1040.9818	2079.9490	2079.9426	3.12	0	76	1e-006	1	K.AGAYDFPSPEWDTVTPEAK.D 997
1024	783.7260	2348.1562	2348.1689	-5.40	1	52	0.00029	1	R.FTEEYQLFEELGKGAFSVVR.R
1046	888.7650	2663.2732	2663.2755	-0.87	1	47	0.00083	1	K.AGAYDFPSPEWDTVTPEAKDLINK.M 1045

Proteins matching the same set of peptides:
IPI00621806 **Mass:** 54651 **Score:** 371 **Queries matched:** 16
Tax_Id=10090 Gene_Symbol=Camk2a Isoform Alpha CaMKII of Calcium/calmodulin-dependent protein kinase type II subunit alpha

13. IPI00122928 **Mass:** 50514 **Score:** 368 **Queries matched:** 18 **emPAI:** 0.66
Tax_Id=10090 Gene_Symbol=Tubb6 Tubulin beta-6 chain

Query	Observed	Mr(expt)	Mr(calc)	ppm	Miss	Score	Expect	Rank	Peptide
339	539.2715	1076.5284	1076.5250	3.17	1	35	0.026	1	K.IREEYPDR.I 338 340
409	572.3189	1142.6232	1142.6270	-3.31	0	(64)	3.3e-005	1	K.LAVNMVPFPR.L 410
431	580.3211	1158.6276	1158.6219	4.92	0	83	4.5e-007	1	K.LAVNMVPFPR.L
660	676.3409	1350.6672	1350.6676	-0.24	0	63	3.8e-005	1	R.IMNTFSVMPSPK.V 659
850	810.9166	1619.8186	1619.8283	-5.94	0	19	0.83	2	R.LHFFMPGFAPLTAR.G
875	846.4407	1690.8668	1690.8600	4.05	0	72	4.3e-006	1	R.ALTVPELTQQMFDAK.N 874
878	848.9237	1695.8328	1695.8257	4.24	0	50	0.00065	1	K.NSSYFVEWIPNNVK.V 877
887	854.4349	1706.8552	1706.8549	0.19	0	(53)	0.00036	2	R.ALTVPELTQQMFDAK.N 889 890
949	929.4739	1856.9332	1856.9342	-0.52	0	36	0.016	3	K.MASTFIGNSTAIQELFK.R
992	672.0167	2013.0283	2013.0353	-3.50	1	116	1.3e-010	2	K.MASTFIGNSTAIQELFKR.I

14. IPI00115546 **Mass:** 40524 **Score:** 367 **Queries matched:** 10 **emPAI:** 0.48
Tax_Id=10090 Gene_Symbol=Gnao1 Isoform Alpha-2 of Guanine nucleotide-binding protein G(o) subunit alpha

Query	Observed	Mr(expt)	Mr(calc)	ppm	Miss	Score	Expect	Rank	Peptide
233	446.2509	890.4872	890.4610	29.4	0	14	3.5	1	R.LFDVGGQR.S
329	529.3133	1056.6120	1056.6179	-5.53	0	72	4.1e-006	1	K.LLLLGAGESGK.S
416	573.3207	1144.6268	1144.6088	15.8	1	4	34	8	K.NLKEDGISAAK.D
707	723.3447	1444.6748	1444.6834	-5.94	0	8	9.9	2	K.IIHEDGFSGEDVK.Q
834	792.3745	1582.7344	1582.7297	3.00	1	62	3.2e-005	1	R.AMDTLGVEYGDKER.K
871	838.4184	1674.8222	1674.8213	0.56	0	115	2.1e-010	1	R.IGAGDYQPTEQDILR.T 872
976	979.4461	1956.8776	1956.8631	7.44	0	101	2.8e-009	1	R.MEDTEPFSAELLSAMMR.L 975
986	987.4356	1972.8566	1972.8580	-0.69	0	(47)	0.00058	1	R.MEDTEPFSAELLSAMMR.L

Proteins matching the same set of peptides:
IPI00230192 **Mass:** 40629 **Score:** 367 **Queries matched:** 10
Tax_Id=10090 Gene_Symbol=Gnao1 Isoform Alpha-1 of Guanine nucleotide-binding protein G(o) subunit alpha

15. IPI00117350 **Mass:** 50634 **Score:** 316 **Queries matched:** 13 **emPAI:** 0.46
Tax_Id=10090 Gene_Symbol=Tuba4a Tubulin alpha-4A chain

Query	Observed	Mr(expt)	Mr(calc)	ppm	Miss	Score	Expect	Rank	Peptide
243	455.2580	908.5014	908.4967	5.21	1	2	33	2	R.LSVDYGKK.S
736	744.4408	1486.8670	1486.8719	-3.24	0	18	0.45	1	R.LISQIVSSITASLR.F
893	859.9458	1717.8770	1717.8747	1.36	0	67	1.3e-005	1	R.NLDIERPTYTNLNR.L 894
934	912.9977	1823.9808	1823.9782	1.47	0	29	0.052	1	K.VGINYQPPTVVPGGDLAK.V 935
1021	768.4006	2302.1800	2302.1957	-6.85	1	45	0.0013	1	R.AVFVDLEPTVIDEIRNGPYR.Q
1028	1205.1035	2408.1924	2408.2012	-3.65	0	125	1.4e-011	1	R.FDGALNVDLTEFQTNLVPYPR.I
1033	805.7347	2414.1823	2414.1978	-6.44	1	78	8e-007	1	R.QLFHPEQLITGKEDAANNYAR.G 1030 1031 1032 1034

16. IPI00120793 **Mass:** 28131 **Score:** 282 **Queries matched:** 11 **emPAI:** 0.40
Tax_Id=10090 Gene_Symbol=Prnp Major prion protein

Query	Observed	Mr(expt)	Mr(calc)	ppm	Miss	Score	Expect	Rank	Peptide
295	508.7724	1015.5302	1015.5298	0.40	0	23	0.55	1	K.QHTVTTTTK.G
349	544.7376	1087.4606	1087.4570	3.33	0	41	0.0019	1	K.ESQAYYDGR.R
406	570.2620	1138.5094	1138.5142	-4.21	0	23	0.2	1	K.GENFTETDVK.M
539	622.7877	1243.5608	1243.5581	2.18	1	39	0.005	1	K.ESQAYYDGRR.S 536 537 538
1002	713.0141	2136.0205	2136.0335	-6.11	1	106	1.2e-009	1	K.QHTVTTTTKGENFTETDVK.M 1003 1004 1005

17. IPI00135284 **Mass:** 36092 **Score:** 250 **Queries matched:** 12 **emPAI:** 0.42
Tax_Id=10090 Gene_Symbol=Gm16374 Glyceraldehyde-3-phosphate dehydrogenase

Query	Observed	Mr(expt)	Mr(calc)	ppm	Miss	Score	Expect	Rank	Peptide
61	596.3636	595.3563	595.3442	20.4	0	8	4.6	2	K.AGAHLK.G
243	455.2580	908.5014	908.5192	-19.52	1	0	52	8	K.AGAHLKGGAK.R
667	678.8332	1355.6518	1355.6465	3.96	1	51	0.00049	1	R.VVDLMAYMASKE.- 666
675	685.3760	1368.7374	1368.7361	0.99	0	59	6.8e-005	1	R.GAAQNIIPASTGAAK.A 674
676	686.8303	1371.6460	1371.6414	3.39	1	(13)	3.2	1	R.VVDLMAYMASKE.-
690	694.8303	1387.6460	1387.6363	7.01	1	(21)	0.49	1	R.VVDLMAYMASKE.-
879	849.4503	1696.8860	1696.8856	0.25	1	76	1.5e-006	1	R.DGRGAAQNIIPASTGAAK.A 880
914	890.4036	1778.7926	1778.7900	1.50	0	85	1.5e-007	1	K.LISWYDNEYGYSNR.V 913

Proteins matching the same set of peptides:
IPI00273646 **Mass:** 36072 **Score:** 250 **Queries matched:** 12
Tax_Id=10090 Gene_Symbol=Gapdh;LOC100042025 Glyceraldehyde-3-phosphate dehydrogenase
IPI00622795 **Mass:** 36074 **Score:** 250 **Queries matched:** 12
Tax_Id=10090 Gene_Symbol=- 36 kDa protein
IPI00848801 **Mass:** 36100 **Score:** 250 **Queries matched:** 12
Tax_Id=10090 Gene_Symbol=- 36 kDa protein
IPI00849045 **Mass:** 36072 **Score:** 250 **Queries matched:** 12
Tax_Id=10090 Gene_Symbol=- 36 kDa protein
IPI00850337 **Mass:** 44633 **Score:** 250 **Queries matched:** 12
Tax_Id=10090 Gene_Symbol=LOC100048117 similar to Glyceraldehyde-3-phosphate dehydrogenase (GAPDH) isoform 1
IPI00850377 **Mass:** 35975 **Score:** 250 **Queries matched:** 12
Tax_Id=10090 Gene_Symbol=- 36 kDa protein
IPI00874682 **Mass:** 36038 **Score:** 250 **Queries matched:** 12
Tax_Id=10090 Gene_Symbol=- 36 kDa protein
IPI00874964 **Mass:** 36512 **Score:** 250 **Queries matched:** 12
Tax_Id=10090 Gene_Symbol=- 36 kDa protein

18. IPI00123176 **Mass:** 36804 **Score:** 236 **Queries matched:** 9 **emPAI:** 0.29
Tax_Id=10090 Gene_Symbol=Gm6316 Glyceraldehyde-3-phosphate dehydrogenase

Query	Observed	Mr(expt)	Mr(calc)	ppm	Miss	Score	Expect	Rank	Peptide
61	596.3636	595.3563	595.3442	20.4	0	8	4.6	2	K.AGAHLK.G
243	455.2580	908.5014	908.5192	-19.52	1	0	52	8	K.AGAHLKGGAK.R
675	685.3760	1368.7374	1368.7361	0.99	0	59	6.8e-005	1	R.GAAQNIIPASTGAAK.A 674
841	808.4230	1614.8314	1614.8512	-12.23	1	0	70	6	R.VPTRNVSVVDLTCR.L
879	849.4503	1696.8860	1696.8856	0.25	1	76	1.5e-006	1	R.DGRGAAQNIIPASTGAAK.A 880
914	890.4036	1778.7926	1778.7900	1.50	0	85	1.5e-007	1	K.LISWYDNEYGYSNR.M 913

19. IPI00474502 **Mass:** 61164 **Score:** 171 **Queries matched:** 10 **emPAI:** 0.23
Tax_Id=10090 Gene_Symbol=Camk2b Calcium/calmodulin-dependent protein kinase type II subunit beta

Query	Observed	Mr(expt)	Mr(calc)	ppm	Miss	Score	Expect	Rank	Peptide
414	572.8060	1143.5974	1143.5964	0.90	0	18	1.6	1	R.FYFENLLAK.N
417	574.3328	1146.6510	1146.6431	6.96	0	62	3.2e-005	1	K.GAILTTMLATR.N
653	670.8925	1339.7704	1339.7711	-0.47	0	63	2e-005	1	R.DLKPENLLLASK.C 654
678	687.3762	1372.7378	1372.7310	4.96	1	23	0.38	1	K.KADGVKPQTNSTK.N 677
696	702.3779	1402.7412	1402.7317	6.80	0	46	0.0019	1	R.LTQYIDGQGRPR.T
848	810.8851	1619.7556	1619.7355	12.4	0	9	7.7	1	R.FTDEYQLYEDIGK.G
996	1040.9818	2079.9490	2079.9426	3.12	0	76	1e-006	1	K.AGAYDFPSPEWDTVTPEAK.N 997

Proteins matching the same set of peptides:
IPI00475302 **Mass:** 60053 **Score:** 171 **Queries matched:** 10
Tax_Id=10090 Gene_Symbol=Camk2b calcium/calmodulin-dependent protein kinase type II subunit beta isoform 3
IPI00515669 **Mass:** 57159 **Score:** 171 **Queries matched:** 10
Tax_Id=10090 Gene_Symbol=Camk2b Calcium/calmodulin-dependent protein kinase II, beta
IPI00648630 **Mass:** 58788 **Score:** 171 **Queries matched:** 10
Tax_Id=10090 Gene_Symbol=Camk2b Calcium/calmodulin-dependent protein kinase II, beta
IPI00649778 **Mass:** 73827 **Score:** 171 **Queries matched:** 10
Tax_Id=10090 Gene_Symbol=Camk2b Calcium/calmodulin-dependent protein kinase II, beta, isoform CRA_b
IPI00875723 **Mass:** 68686 **Score:** 171 **Queries matched:** 10
Tax_Id=10090 Gene_Symbol=Camk2b 68 kDa protein
IPI00470962 **Mass:** 61436 **Score:** 171 **Queries matched:** 10
Tax_Id=10090 Gene_Symbol=Camk2b calcium/calmodulin-dependent protein kinase type II subunit beta isoform 1
IPI00649296 **Mass:** 65906 **Score:** 171 **Queries matched:** 10
Tax_Id=10090 Gene_Symbol=Camk2b Calcium/calmodulin-dependent protein kinase II, beta

20. IPI00119063 **Mass:** 523342 **Score:** 170 **Queries matched:** 4 **emPAI:** 0.02
Tax_Id=10090 Gene_Symbol=Lrp1 Prolow-density lipoprotein receptor-related protein 1

Query	Observed	Mr(expt)	Mr(calc)	ppm	Miss	Score	Expect	Rank	Peptide
679	687.8944	1373.7742	1373.7667	5.50	0	73	2.2e-006	1	R.YVVISQGLDKPR.A 680
851	811.4091	1620.8036	1620.8148	-6.85	0	75	2.2e-006	1	K.AVTDEEPFLIFANR.Y
891	854.9852	1707.9558	1707.9308	14.7	0	51	0.00023	1	K.TVLWPNGLSLDIPAGR.L

21. IPI00136703 **Mass:** 42971 **Score:** 170 **Queries matched:** 5 **emPAI:** 0.35
Tax_Id=10090 Gene_Symbol=Ckb Creatine kinase B-type

Query	Observed	Mr(expt)	Mr(calc)	ppm	Miss	Score	Expect	Rank	Peptide
305	516.2994	1030.5842	1030.5481	35.1	0	15	2.4	1	K.LLIEMEQR.L
838	801.9229	1601.8312	1601.8260	3.25	0	96	1.6e-008	1	K.LAVEALSSLDGDLSGR.Y
863	828.9160	1655.8174	1655.8188	-0.85	0	59	8.3e-005	1	R.LEQGQAIDDLMPAQK.-
944	924.9993	1847.9840	1847.9703	7.44	0	56	0.00012	1	R.LGFSEVELVQMVVDGVK.L
1075	897.6848	3586.7101	3586.7800	-19.48	0	54	9.8e-005	1	K.SMTEAEQQQLIDDHFLFDKPVSPLLLASGMAR.D

22. IPI00319602 **Mass:** 47493 **Score:** 159 **Queries matched:** 3 **emPAI:** 0.14
Tax_Id=10090 Gene_Symbol=Cnp Isoform CNPII of 2',3'-cyclic-nucleotide 3'-phosphodiesterase

Query	Observed	Mr(expt)	Mr(calc)	ppm	Miss	Score	Expect	Rank	Peptide
291	500.7604	999.5062	999.4808	25.5	1	1	57	3	-.MNTSFTRK.S
560	628.8163	1255.6180	1255.6157	1.89	0	70	6.3e-006	1	K.GGSQGEAVGELPR.G
861	824.9201	1647.8256	1647.8216	2.45	1	124	2.8e-011	1	K.AAGAEEYAQQEVVKR.S

23. IPI00138892 **Mass:** 15004 **Score:** 158 **Queries matched:** 4 **emPAI:** 0.85
Tax_Id=10090 Gene_Symbol=Uba52 Ubiquitin-60S ribosomal protein L40

Query	Observed	Mr(expt)	Mr(calc)	ppm	Miss	Score	Expect	Rank	Peptide
82	648.4186	647.4113	647.4006	16.5	0	35	0.0084	1	R.LIFAGK.Q
752	762.3943	1522.7740	1522.7740	0.06	1	82	4e-007	1	K.IQDKEGIPPDQQR.L 753
921	894.4671	1786.9196	1786.9200	-0.20	0	82	3.7e-007	1	K.TITLEVEPSDTIENVK.A

Proteins matching the same set of peptides:
IPI00470152 **Mass:** 18282 **Score:** 158 **Queries matched:** 4
Tax_Id=10090 Gene_Symbol=Rps27a Ubiquitin-40S ribosomal protein S27a
IPI00895319 **Mass:** 22277 **Score:** 158 **Queries matched:** 4
Tax_Id=10090 Gene_Symbol=2810422J05Rik 22 kDa protein
IPI00895479 **Mass:** 27181 **Score:** 158 **Queries matched:** 4
Tax_Id=10090 Gene_Symbol=2810422J05Rik 27 kDa protein
IPI00918763 **Mass:** 11021 **Score:** 158 **Queries matched:** 4
Tax_Id=10090 Gene_Symbol=Uba52 Ubiqutin subunit 1
IPI00750889 **Mass:** 22822 **Score:** 158 **Queries matched:** 4
Tax_Id=10090 Gene_Symbol=Ubc Ubc protein
IPI00923013 **Mass:** 26603 **Score:** 158 **Queries matched:** 4
Tax_Id=10090 Gene_Symbol=Ubb Ubiquitin B
IPI00923037 **Mass:** 22578 **Score:** 158 **Queries matched:** 4
Tax_Id=10090 Gene_Symbol=Ubc Putative uncharacterized protein
IPI00139518 **Mass:** 34348 **Score:** 158 **Queries matched:** 4
Tax_Id=10090 Gene_Symbol=Ubb Polyubiquitin-B
IPI00969323 **Mass:** 82603 **Score:** 158 **Queries matched:** 4
Tax_Id=10090 Gene_Symbol=Ubc Polyubiquitin-C
IPI00755916 **Mass:** 82614 **Score:** 158 **Queries matched:** 4
Tax_Id=10090 Gene_Symbol=Ubc ubiquitin

24. IPI00323357 **Mass:** 71055 **Score:** 136 **Queries matched:** 7 **emPAI:** 0.09
Tax_Id=10090 Gene_Symbol=Hspa8 Heat shock cognate 71 kDa protein

Query	Observed	Mr(expt)	Mr(calc)	ppm	Miss	Score	Expect	Rank	Peptide
464	590.8160	1179.6174	1179.6135	3.31	1	25	0.25	1	K.VQVEYKGETK.S
520	614.8207	1227.6268	1227.6207	4.97	0	14	3	2	K.VEIIANDQGNR.T
612	652.3111	1302.6076	1302.5914	12.5	0	5	16	2	K.NSLESYAFNMK.A
733	744.3572	1486.6998	1486.6940	3.93	0	69	7.6e-006	1	R.TTPSYVAFTDTER.L 734
843	808.9025	1615.7904	1615.7804	6.25	0	3	35	2	K.SFYPEEVSSMVLTK.M
987	991.5050	1980.9954	1980.9905	2.49	0	59	6.2e-005	1	K.TVTNAVVTVPAYFNDSQR.Q

25. IPI00348094 **Mass:** 51093 **Score:** 133 **Queries matched:** 8 **emPAI:** 0.21
Tax_Id=10090 Gene_Symbol=Tubb1 Tubulin, beta 1

Query	Observed	Mr(expt)	Mr(calc)	ppm	Miss	Score	Expect	Rank	Peptide
339	539.2715	1076.5284	1076.5250	3.17	1	35	0.026	1	K.IREEYPDR.I 338 340
409	572.3189	1142.6232	1142.6270	-3.31	0	(64)	3.3e-005	1	K.LAVNMVPFPR.L 410
431	580.3211	1158.6276	1158.6219	4.92	0	83	4.5e-007	1	K.LAVNMVPFPR.L
632	660.3535	1318.6924	1318.6955	-2.31	0	11	6.1	2	R.ILNSFSVMPSPK.V 633

26. IPI00608020 **Mass:** 20817 **Score:** 116 **Queries matched:** 2 **emPAI:** 0.35
Tax_Id=10090 Gene_Symbol=Ftl1 ferritin light chain 1

Query	Observed	Mr(expt)	Mr(calc)	ppm	Miss	Score	Expect	Rank	Peptide
730	740.8624	1479.7102	1479.6954	10.1	0	87	1.2e-007	1	R.QNYSTEVEAAVNR.L
1071	820.6419	3278.5385	3278.5587	-6.17	1	62	1.9e-005	1	R.ALFQDVQKPSQDEWGKTQEAMEAALAMEK.N

Proteins matching the same set of peptides:
IPI00762203 **Mass:** 20847 **Score:** 116 **Queries matched:** 2
Tax_Id=10090 Gene_Symbol=Ftl1 Ferritin light chain 1

27. IPI00136372 **Mass:** 70163 **Score:** 94 **Queries matched:** 5 **emPAI:** 0.10
Tax_Id=10090 Gene_Symbol=Syn1 Isoform Ib of Synapsin-1

Query	Observed	Mr(expt)	Mr(calc)	ppm	Miss	Score	Expect	Rank	Peptide
238	450.2198	898.4250	898.4621	-41.20	0	6	11	2	R.QAGAPQATR.Q
470	594.3597	1186.7048	1186.7074	-2.14	0	24	0.15	1	R.SLKPDFVLIR.Q
639	663.8334	1325.6522	1325.6503	1.46	0	57	0.00014	1	K.TYATAEPFIDAK.Y 640
897	862.9027	1723.7908	1723.7869	2.29	0	47	0.00094	1	K.TNTGSAMLEQIAMSDR.Y

Proteins matching the same set of peptides:
IPI00649467 **Mass:** 71610 **Score:** 94 **Queries matched:** 5
Tax_Id=10090 Gene_Symbol=Syn1 Isoform 3 of Synapsin-1
IPI00649886 **Mass:** 74223 **Score:** 94 **Queries matched:** 5
Tax_Id=10090 Gene_Symbol=Syn1 Isoform Ia of Synapsin-1

28. IPI00555023 **Mass:** 23765 **Score:** 91 **Queries matched:** 2 **emPAI:** 0.14
Tax_Id=10090 Gene_Symbol=Gstp1 Glutathione S-transferase P 1

Query	Observed	Mr(expt)	Mr(calc)	ppm	Miss	Score	Expect	Rank	Peptide
662	676.3783	1350.7420	1350.7336	6.25	0	70	5.6e-006	1	M.PPYTIVYFPVR.G 661

29. IPI00114375 **Mass:** 62638 **Score:** 88 **Queries matched:** 1 **emPAI:** 0.05
Tax_Id=10090 Gene_Symbol=Dpysl2 Dihydropyrimidinase-related protein 2

Query	Observed	Mr(expt)	Mr(calc)	ppm	Miss	Score	Expect	Rank	Peptide
967	**956.0165**	**1910.0184**	**1910.0149**	**1.85**	**0**	**88**	**7.4e-008**	**1**	**R.FQLTDSQIYEVLSVIR.D**

---

30. IPI00652902 **Mass:** 41033 **Score:** 88 **Queries matched:** 2 **emPAI:** 0.17
Tax_Id=10090 Gene_Symbol=Gnai2 Putative uncharacterized protein

Query	Observed	Mr(expt)	Mr(calc)	ppm	Miss	Score	Expect	Rank	Peptide
329	529.3133	1056.6120	1056.6179	-5.53	0	72	4.1e-006	1	K.LLLLGAGESGK.S
902	**868.9853**	**1735.9560**	**1735.9542**	**1.05**	**0**	**50**	**0.00034**	**1**	**R.AVVYSNTIQSIMAIVK.A**

---

31. IPI00114642 **Mass:** 13928 **Score:** 77 **Queries matched:** 2 **emPAI:** 0.25
Tax_Id=10090 Gene_Symbol=Hist1h2bn;Hist1h2bl;Hist1h2bj;Hist1h2bf Histone H2B type 1-F/J/L

Query	Observed	Mr(expt)	Mr(calc)	ppm	Miss	Score	Expect	Rank	Peptide
903	**872.4119**	**1742.8092**	**1742.8120**	**-1.58**	**0**	**75**	**1.9e-006**	**1**	**K.AMGIMNSFVNDIFER.I** 904

Proteins matching the same set of peptides:
IPI00124518 **Mass:** 13359 **Score:** 77 **Queries matched:** 2
Tax_Id=10090 Gene_Symbol=Hist2h2bb Hist2h2bb protein
IPI00134534 **Mass:** 14879 **Score:** 77 **Queries matched:** 2
Tax_Id=10090 Gene_Symbol=Gm11277;Gm13646 Histone H2B
IPI00187543 **Mass:** 13985 **Score:** 77 **Queries matched:** 2
Tax_Id=10090 Gene_Symbol=Hist2h2be Histone H2B type 2-E
IPI00227930 **Mass:** 13912 **Score:** 77 **Queries matched:** 2
Tax_Id=10090 Gene_Symbol=Hist1h2bh Histone H2B type 1-H
IPI00229539 **Mass:** 17248 **Score:** 77 **Queries matched:** 2
Tax_Id=10090 Gene_Symbol=Hist3h2bb-ps histone H2B type 3-B
IPI00265768 **Mass:** 13912 **Score:** 77 **Queries matched:** 2
Tax_Id=10090 Gene_Symbol=Hist1h2bk Histone H2B type 1-K
IPI00282266 **Mass:** 13898 **Score:** 77 **Queries matched:** 2
Tax_Id=10090 Gene_Symbol=Hist1h2bg;Hist1h2be;Gm9998;Hist1h2bc Histone H2B type 1-C/E/G
IPI00282269 **Mass:** 13928 **Score:** 77 **Queries matched:** 2
Tax_Id=10090 Gene_Symbol=Hist1h2bm Histone H2B type 1-M
IPI00348270 **Mass:** 13912 **Score:** 77 **Queries matched:** 2
Tax_Id=10090 Gene_Symbol=Hist2h2bb Histone H2B type 2-B
IPI00453744 **Mass:** 15968 **Score:** 77 **Queries matched:** 2
Tax_Id=10090 Gene_Symbol=Hist1h2bp 16 kDa protein
IPI00461514 **Mass:** 14987 **Score:** 77 **Queries matched:** 2
Tax_Id=10090 Gene_Symbol=Gm13646 Histone H2B
IPI00553774 **Mass:** 13986 **Score:** 77 **Queries matched:** 2
Tax_Id=10090 Gene_Symbol=Hist3h2ba Histone H2B type 3-A
IPI00554853 **Mass:** 13944 **Score:** 77 **Queries matched:** 2
Tax_Id=10090 Gene_Symbol=Hist1h2bb Histone H2B type 1-B
IPI00648991 **Mass:** 13984 **Score:** 77 **Queries matched:** 2
Tax_Id=10090 Gene_Symbol=Hist1h2bp Isoform 1 of Histone H2B type 1-P
IPI00761713 **Mass:** 15555 **Score:** 77 **Queries matched:** 2
Tax_Id=10090 Gene_Symbol=Hist1h2bp Isoform 2 of Histone H2B type 1-P
IPI00874492 **Mass:** 14746 **Score:** 77 **Queries matched:** 2
Tax_Id=10090 Gene_Symbol=Hist1h2bm 15 kDa protein
IPI00874654 **Mass:** 14787 **Score:** 77 **Queries matched:** 2
Tax_Id=10090 Gene_Symbol=Hist1h2bg;Hist1h2be;Gm9998;Hist1h2bc Histone H2B
IPI00874970 **Mass:** 14951 **Score:** 77 **Queries matched:** 2
Tax_Id=10090 Gene_Symbol=Hist1h2bn;Hist1h2bl;Hist1h2bj;Hist1h2bf Histone H2B
IPI00875277 **Mass:** 14242 **Score:** 77 **Queries matched:** 2
Tax_Id=10090 Gene_Symbol=Hist1h2bk 14 kDa protein
IPI00875523 **Mass:** 14900 **Score:** 77 **Queries matched:** 2
Tax_Id=10090 Gene_Symbol=Hist1h2bh Histone H2B
IPI00875901 **Mass:** 14806 **Score:** 77 **Queries matched:** 2
Tax_Id=10090 Gene_Symbol=Hist1h2bk Histone H2B
IPI00876167 **Mass:** 14126 **Score:** 77 **Queries matched:** 2
Tax_Id=10090 Gene_Symbol=Hist1h2bn;Hist1h2bl;Hist1h2bj;Hist1h2bf 14 kDa protein
IPI00876432 **Mass:** 14148 **Score:** 77 **Queries matched:** 2
Tax_Id=10090 Gene_Symbol=Hist1h2bn;Hist1h2bl;Hist1h2bj;Hist1h2bf Histone H2B
IPI00876549 **Mass:** 14902 **Score:** 77 **Queries matched:** 2
Tax_Id=10090 Gene_Symbol=Hist1h2bn;Hist1h2bl;Hist1h2bj;Hist1h2bf Histone H2B
IPI00876550 **Mass:** 14812 **Score:** 77 **Queries matched:** 2
Tax_Id=10090 Gene_Symbol=- 15 kDa protein

---

32. IPI00118569 **Mass:** 44369 **Score:** 72 **Queries matched:** 1 **emPAI:** 0.07
Tax_Id=10090 Gene_Symbol=Gna13 Guanine nucleotide-binding protein subunit alpha-13

Query	Observed	Mr(expt)	Mr(calc)	ppm	Miss	Score	Expect	Rank	Peptide
329	529.3133	1056.6120	1056.6179	-5.53	0	72	4.1e-006	1	K.ILLLGAGESGK.S

Proteins matching the same set of peptides:
IPI00230191 **Mass:** 44238 **Score:** 72 **Queries matched:** 1
Tax_Id=10090 Gene_Symbol=Gna12 Guanine nucleotide-binding protein subunit alpha-12
IPI00649388 **Mass:** 20095 **Score:** 72 **Queries matched:** 1
Tax_Id=10090 Gene_Symbol=Gna13 Putative uncharacterized protein

---

33. IPI00310091 **Mass:** 66079 **Score:** 72 **Queries matched:** 1 **emPAI:** 0.05
Tax_Id=10090 Gene_Symbol=Ppp2r1a Serine/threonine-protein phosphatase 2A 65 kDa regulatory subunit A alpha isoform

Query	Observed	Mr(expt)	Mr(calc)	ppm	Miss	Score	Expect	Rank	Peptide
1008	**732.0782**	**2193.2128**	**2193.2256**	**-5.86**	**0**	**72**	**1.5e-006**	**1**	**K.IGPILDNSTLQSEVKPILEK.L**

---

34. IPI00130280 **Mass:** 59830 **Score:** 72 **Queries matched:** 1 **emPAI:** 0.05
Tax_Id=10090 Gene_Symbol=Atp5a1 ATP synthase subunit alpha, mitochondrial

Query	Observed	Mr(expt)	Mr(calc)	ppm	Miss	Score	Expect	Rank	Peptide

Query	Observed	Mr(expt)	Mr(calc)	ppm	Miss	Score	Expect	Rank	Peptide
1023	1169.5824	2337.1502	2337.1601	-4.21	0	72	2.9e-006	1	R.EVAAFAQFGSDLDAATQQLLSR.G

Proteins matching the same set of peptides:
IPI00857439 **Mass:** 54675 **Score:** 72 **Queries matched:** 1
Tax_Id=10090 Gene_Symbol=Atp5a1 ATP synthase subunit alpha

---

35. IPI00625729 **Mass:** 66079 **Score:** 70 **Queries matched:** 3 **emPAI:** 0.05
Tax_Id=10090 Gene_Symbol=Krt1 Keratin, type II cytoskeletal 1

Query	Observed	Mr(expt)	Mr(calc)	ppm	Miss	Score	Expect	Rank	Peptide
694	697.3569	1392.6992	1392.7249	-18.40	1	0	75	8	R.TNAENEFVTIKK.D
728	738.4001	1474.7856	1474.7780	5.20	0	70	6.4e-006	1	R.FLEQQNQVLQTK.W
880	849.4527	1696.8908	1696.8342	33.4	1	3	28	4	K.DVDSAYMTKVELQAK.A

---

36. IPI00462140 **Mass:** 61379 **Score:** 70 **Queries matched:** 2 **emPAI:** 0.05
Tax_Id=10090 Gene_Symbol=Krt77 Keratin, type II cytoskeletal 1b

Query	Observed	Mr(expt)	Mr(calc)	ppm	Miss	Score	Expect	Rank	Peptide
681	689.3424	1376.6702	1376.7300	-43.38	1	1	65	9	R.TNAENDFVVLKK.D
728	738.4001	1474.7856	1474.7780	5.20	0	70	6.4e-006	1	R.FLEQQNQVLQTK.W

---

37. IPI00408378 **Mass:** 28046 **Score:** 69 **Queries matched:** 1 **emPAI:** 0.12
Tax_Id=10090 Gene_Symbol=Ywhaq Isoform 1 of 14-3-3 protein theta

Query	Observed	Mr(expt)	Mr(calc)	ppm	Miss	Score	Expect	Rank	Peptide
755	766.8602	1531.7058	1531.7114	-3.62	0	69	7.3e-006	1	K.AVTEQGAELSNEER.N

Proteins matching the same set of peptides:
IPI00656269 **Mass:** 28025 **Score:** 69 **Queries matched:** 1
Tax_Id=10090 Gene_Symbol=Ywhaq Isoform 2 of 14-3-3 protein theta
IPI00754545 **Mass:** 35930 **Score:** 69 **Queries matched:** 1
Tax_Id=10090 Gene_Symbol=Gm2423 similar to Ywhaq protein
IPI00853924 **Mass:** 34613 **Score:** 69 **Queries matched:** 1
Tax_Id=10090 Gene_Symbol=Ywhaq 34 kDa protein

---

38. IPI00119067 **Mass:** 23731 **Score:** 68 **Queries matched:** 1 **emPAI:** 0.14
Tax_Id=10090 Gene_Symbol=Tppp Tubulin polymerization-promoting protein

Query	Observed	Mr(expt)	Mr(calc)	ppm	Miss	Score	Expect	Rank	Peptide
964	948.9969	1895.9792	1895.9516	14.6	0	68	1.1e-005	1	R.TITFEQFQEALEELAK.K

---

39. IPI00323571 **Mass:** 35901 **Score:** 67 **Queries matched:** 5 **emPAI:** 0.30
Tax_Id=10090 Gene_Symbol=Apoe Apolipoprotein E

Query	Observed	Mr(expt)	Mr(calc)	ppm	Miss	Score	Expect	Rank	Peptide
448	585.8325	1169.6504	1169.6516	-1.02	1	43	0.0027	1	R.LGKEVQAAQAR.L 447
520	614.8207	1227.6268	1227.6320	-4.17	1	45	0.0025	1	R.GRLEEVGNQAR.D
681	689.3424	1376.6702	1376.6718	-1.12	1	36	0.021	1	R.SKMEEQTQQIR.L
749	755.9105	1509.8064	1509.8012	3.50	1	6	14	1	R.TANLGAGAAQPLRDR.A

---

40. IPI00272878 **Mass:** 98140 **Score:** 66 **Queries matched:** 2 **emPAI:** 0.03
Tax_Id=10090 Gene_Symbol=Dnm1 Isoform 1 of Dynamin-1

Query	Observed	Mr(expt)	Mr(calc)	ppm	Miss	Score	Expect	Rank	Peptide
328	529.2714	1056.5282	1056.5200	7.82	0	6	15	1	R.SPTSSPTPQR.R
657	672.8652	1343.7158	1343.7045	8.46	0	66	1.4e-005	1	K.TSGNQDEILVIR.K

Proteins matching the same set of peptides:
IPI00331293 **Mass:** 97762 **Score:** 66 **Queries matched:** 2
Tax_Id=10090 Gene_Symbol=Dnm1 Isoform 4 of Dynamin-1
IPI00465648 **Mass:** 96209 **Score:** 66 **Queries matched:** 2
Tax_Id=10090 Gene_Symbol=Dnm1 Isoform 3 of Dynamin-1
IPI00753037 **Mass:** 96395 **Score:** 66 **Queries matched:** 2
Tax_Id=10090 Gene_Symbol=Dnm1 Isoform 5 of Dynamin-1
IPI00890133 **Mass:** 97954 **Score:** 66 **Queries matched:** 2
Tax_Id=10090 Gene_Symbol=Dnm1 Uncharacterized protein

---

41. IPI00230145 **Mass:** 21224 **Score:** 62 **Queries matched:** 3 **emPAI:** 0.16
Tax_Id=10090 Gene_Symbol=Fth1 Ferritin heavy chain

Query	Observed	Mr(expt)	Mr(calc)	ppm	Miss	Score	Expect	Rank	Peptide
422	577.3004	1152.5862	1152.5887	-2.15	0	11	6	1	K.ELGDHVTNLR.K
571	634.3499	1266.6852	1266.6932	-6.26	0	59	7.2e-005	1	K.SVNQSLLELHK.L 572

---

42. IPI00123313 **Mass:** 118931 **Score:** 62 **Queries matched:** 1 **emPAI:** 0.03
Tax_Id=10090 Gene_Symbol=Uba1 Ubiquitin-like modifier-activating enzyme 1

Query	Observed	Mr(expt)	Mr(calc)	ppm	Miss	Score	Expect	Rank	Peptide
962	943.0261	1884.0376	1884.0357	1.06	0	62	2.1e-005	1	R.AAVASLLQSVQVPEFTPK.S

---

43. IPI00131366 **Mass:** 60627 **Score:** 61 **Queries matched:** 4 **emPAI:** 0.05
Tax_Id=10090 Gene_Symbol=Krt6b Keratin, type II cytoskeletal 6B

Query	Observed	Mr(expt)	Mr(calc)	ppm	Miss	Score	Expect	Rank	Peptide
610	651.8621	1301.7096	1301.7078	1.40	0	61	4.7e-005	1	R.SLDLDSIIAEVK.A
705	717.8544	1433.6942	1433.7375	-30.17	1	0	57	3	R.GYSASSARVPGLNR.S
880	849.4527	1696.8908	1696.8342	33.4	1	3	28	4	K.DVDAAYMTKVELQAK.A 879

Proteins matching the same set of peptides:
IPI00785403 **Mass:** 59832 **Score:** 61 **Queries matched:** 4
Tax_Id=10090 Gene_Symbol=Krt6b keratin, type II cytoskeletal 6B

44. IPI00462072 **Mass:** 47453 **Score:** 60 **Queries matched:** 2 **emPAI:** 0.07
Tax_Id=10090 Gene_Symbol=Eno1;Gm5506 Alpha-enolase

Query	Observed	Mr(expt)	Mr(calc)	ppm	Miss	Score	Expect	Rank	Peptide
706	720.3782	1438.7418	1438.7344	5.19	0	60	7.3e-005	1	R.YITPDQLADLYK.S
1062	990.7887	2969.3443	2969.3508	-2.21	0	14	0.96	1	K.SFVQNYPVVSIEDPFDQDDWGAWQK.F

Proteins matching the same set of peptides:
IPI00648821 **Mass:** 40099 **Score:** 60 **Queries matched:** 2
Tax_Id=10090 Gene_Symbol=Eno1 Enolase

45. IPI00266188 **Mass:** 18812 **Score:** 58 **Queries matched:** 2 **emPAI:** 0.18
Tax_Id=10090 Gene_Symbol=Cfl2 Cofilin-2

Query	Observed	Mr(expt)	Mr(calc)	ppm	Miss	Score	Expect	Rank	Peptide
651	669.3212	1336.6278	1336.6187	6.87	0	54	0.00022	1	R.YALYDATYETK.E 650

Proteins matching the same set of peptides:
IPI00407543 **Mass:** 25293 **Score:** 58 **Queries matched:** 2
Tax_Id=10090 Gene_Symbol=Cfl1 Putative uncharacterized protein
IPI00848816 **Mass:** 18731 **Score:** 58 **Queries matched:** 2
Tax_Id=10090 Gene_Symbol=LOC100048522 similar to Cofilin-1
IPI00890117 **Mass:** 18776 **Score:** 58 **Queries matched:** 2
Tax_Id=10090 Gene_Symbol=Cfl1 Cofilin-1

46. IPI00230680 **Mass:** 115702 **Score:** 57 **Queries matched:** 2 **emPAI:** 0.03
Tax_Id=10090 Gene_Symbol=Atp4a Potassium-transporting ATPase alpha chain 1

Query	Observed	Mr(expt)	Mr(calc)	ppm	Miss	Score	Expect	Rank	Peptide
327	528.2854	1054.5562	1054.5520	4.07	1	18	1.1	1	K.GGDRVPADIR.I
685	691.3808	1380.7470	1380.7435	2.55	0	57	0.00012	1	R.VIMVTGDHPITAK.A

47. IPI00268145 **Mass:** 115394 **Score:** 57 **Queries matched:** 3 **emPAI:** 0.03
Tax_Id=10090 Gene_Symbol=Atp12a Potassium-transporting ATPase alpha chain 2

Query	Observed	Mr(expt)	Mr(calc)	ppm	Miss	Score	Expect	Rank	Peptide
447	585.8287	1169.6428	1169.6516	-7.52	1	11	4.9	2	R.NIAVEQVNKR.E 448
685	691.3808	1380.7470	1380.7435	2.55	0	57	0.00012	1	K.VIMVTGDHPITAK.A

Proteins matching the same set of peptides:
IPI00877215 **Mass:** 115395 **Score:** 57 **Queries matched:** 3
Tax_Id=10090 Gene_Symbol=Atp12a potassium-transporting ATPase alpha chain 2

48. IPI00222632 **Mass:** 21791 **Score:** 52 **Queries matched:** 1 **emPAI:** 0.15
Tax_Id=10090 Gene_Symbol=Rab31 ras-related protein Rab-31

Query	Observed	Mr(expt)	Mr(calc)	ppm	Miss	Score	Expect	Rank	Peptide
839	802.4271	1602.8396	1602.8365	1.94	0	52	0.00043	1	K.NAINIEELFQGISR.Q

49. IPI00755181 **Mass:** 57178 **Score:** 49 **Queries matched:** 1 **emPAI:** 0.06
Tax_Id=10090 Gene_Symbol=Krt10 keratin, type I cytoskeletal 10

Query	Observed	Mr(expt)	Mr(calc)	ppm	Miss	Score	Expect	Rank	Peptide
683	691.3288	1380.6430	1380.6408	1.60	0	49	0.00076	1	R.ALEESNYELEGK.I

Proteins matching the same set of peptides:
IPI00798492 **Mass:** 57906 **Score:** 49 **Queries matched:** 1
Tax_Id=10090 Gene_Symbol=Krt10 Isoform 1 of Keratin, type I cytoskeletal 10
IPI00828744 **Mass:** 57197 **Score:** 49 **Queries matched:** 1
Tax_Id=10090 Gene_Symbol=Krt10 Isoform 2 of Keratin, type I cytoskeletal 10

50. IPI00134492 **Mass:** 52818 **Score:** 47 **Queries matched:** 2 **emPAI:** 0.13
Tax_Id=10090 Gene_Symbol=Syn2 Isoform IIb of Synapsin-2

Query	Observed	Mr(expt)	Mr(calc)	ppm	Miss	Score	Expect	Rank	Peptide
726	737.4225	1472.8304	1472.8313	-0.57	0	29	0.051	1	R.EMLTLPTFPVVVK.I
897	862.9027	1723.7908	1723.7869	2.29	0	47	0.00094	1	K.TNTGSAMLEQIAMSDR.Y

Proteins matching the same set of peptides:
IPI00469548 **Mass:** 63618 **Score:** 47 **Queries matched:** 2
Tax_Id=10090 Gene_Symbol=Syn2 Isoform IIa of Synapsin-2

51. IPI00415402 **Mass:** 67925 **Score:** 47 **Queries matched:** 1 **emPAI:** 0.05
Tax_Id=10090 Gene_Symbol=Stxbp1 Isoform 1 of Syntaxin-binding protein 1

Query	Observed	Mr(expt)	Mr(calc)	ppm	Miss	Score	Expect	Rank	Peptide
516	612.8163	1223.6180	1223.6146	2.82	0	47	0.0014	1	R.ISEQTYQLSR.W

Proteins matching the same set of peptides:
IPI00415403 **Mass:** 69091 **Score:** 47 **Queries matched:** 1
Tax_Id=10090 Gene_Symbol=Stxbp1 Isoform 2 of Syntaxin-binding protein 1
IPI00756061 **Mass:** 49457 **Score:** 47 **Queries matched:** 1
Tax_Id=10090 Gene_Symbol=Stxbp1 49 kDa protein

52. IPI00656325 **Mass:** 83131 **Score:** 45 **Queries matched:** 2 **emPAI:** 0.04
Tax_Id=10090 Gene_Symbol=Nsf Vesicle-fusing ATPase

Query	Observed	Mr(expt)	Mr(calc)	ppm	Miss	Score	Expect	Rank	Peptide
712	729.3977	1456.7808	1456.7773	2.42	0	45	0.0018	1	R.VLDDGELLVQQTK.N 711

Proteins matching the same set of peptides:

IPI00876255 **Mass:** 83174 **Score:** 45 **Queries matched:** 2
Tax_Id=10090 Gene_Symbol=Nsf 83 kDa protein

---

53. IPI00135106 **Mass:** 19584 **Score:** 41 **Queries matched:** 4 **emPAI:** 0.37
Tax_Id=10090 Gene_Symbol=Mobp Isoform 1 of Myelin-associated oligodendrocyte basic protein

Query	Observed	Mr(expt)	Mr(calc)	ppm	Miss	Score	Expect	Rank	Peptide
377	557.8031	1113.5916	1113.5891	2.32	1	33	0.041	1	R.SSPLRGPGTSR.G 378
433	580.8276	1159.6406	1159.6462	-4.78	0	38	0.011	1	K.HQPAASPVVVR.A 434

Mascot: http://www.matrixscience.com/

Mascot Search Results

```
User          : Dominic
Email         : dominic.kurian@bbsrc.ac.uk
Search title  : IPI Mouse_15-02-2010
MS data file  : D:\Peaklist\Dominic Kurian\QTofDK20010803 GATC P 0010881.pkl
Database      : IPI_mouse MOUSE_v3_79 (54943 sequences; 24917557 residues)
Timestamp     : 15 Feb 2011 at 15:10:35 GMT
Protein hits  : IPI00420725 Tax_Id=10090 Gene_Symbol=Camk2a Uncharacterized protein
                IPI00474502 Tax_Id=10090 Gene_Symbol=Camk2b Calcium/calmodulin-dependent protein kinase type II subunit beta
                IPI00112584 Tax_Id=10090 Gene_Symbol=Camk2d Isoform 1 of Calcium/calmodulin-dependent protein kinase type II subunit
                IPI00124695 Tax_Id=10090 Gene_Symbol=Camk2g Isoform 1 of Calcium/calmodulin-dependent protein kinase type II subunit
                IPI00109073 Tax_Id=10090 Gene_Symbol=Tubb4 Tubulin beta-4 chain
                IPI00115546 Tax_Id=10090 Gene_Symbol=Gnao1 Isoform Alpha-2 of Guanine nucleotide-binding protein G(o) subunit alpha
                IPI00323571 Tax_Id=10090 Gene_Symbol=Apoe Apolipoprotein E
                IPI00112251 Tax_Id=10090 Gene_Symbol=Tubb3 Tubulin beta-3 chain
                IPI00110850 Tax_Id=10090 Gene_Symbol=Actb Actin, cytoplasmic 1
                IPI00109061 Tax_Id=10090 Gene_Symbol=Tubb2b Tubulin beta-2B chain
                IPI00122048 Tax_Id=10090 Gene_Symbol=Atp1a3 Sodium/potassium-transporting ATPase subunit alpha-3
                IPI00138892 Tax_Id=10090 Gene_Symbol=Uba52 Ubiquitin-60S ribosomal protein L40
                IPI00120793 Tax_Id=10090 Gene_Symbol=Prnp Major prion protein
                IPI00122928 Tax_Id=10090 Gene_Symbol=Tubb6 Tubulin beta-6 chain
                IPI00225062 Tax_Id=10090 Gene_Symbol=Srrm2 Isoform 3 of Serine/arginine repetitive matrix protein 2
                IPI00221528 Tax_Id=10090 Gene_Symbol=Actbl2 Beta-actin-like protein 2
                IPI00404590 Tax_Id=10090 Gene_Symbol=H1f0 Putative uncharacterized protein
                IPI00323357 Tax_Id=10090 Gene_Symbol=Hspa8 Heat shock cognate 71 kDa protein
                IPI00469012 Tax_Id=10090 Gene_Symbol=Tbc1d10b TBC1 domain family member 10B
                IPI00223714 Tax_Id=10090 Gene_Symbol=Hist1h1e Histone H1.4
                IPI00656325 Tax_Id=10090 Gene_Symbol=Nsf Vesicle-fusing ATPase
                IPI00377681 Tax_Id=10090 Gene_Symbol=N28178 Isoform 2 of Protein KIAA1045
                IPI00122069 Tax_Id=10090 Gene_Symbol=Prkcc Protein kinase C gamma type
                IPI00136703 Tax_Id=10090 Gene_Symbol=Ckb Creatine kinase B-type
                IPI00347110 Tax_Id=10090 Gene_Symbol=Krt73 Keratin, type II cytoskeletal 73
                IPI00403219 Tax_Id=10090 Gene_Symbol=Gm14496 Novel vomeronasal 2, receptor family protein
                IPI00119853 Tax_Id=10090 Gene_Symbol=Gnas Isoform Gnas-1 of Guanine nucleotide-binding protein G(s) subunit alpha is
                IPI00347770 Tax_Id=10090 Gene_Symbol=Gnat3 Guanine nucleotide-binding protein G(t) subunit alpha-3
                IPI00118569 Tax_Id=10090 Gene_Symbol=Gna13 Guanine nucleotide-binding protein subunit alpha-13
                IPI00123176 Tax_Id=10090 Gene_Symbol=Gm6316 Glyceraldehyde-3-phosphate dehydrogenase
                IPI00348094 Tax_Id=10090 Gene_Symbol=Tubb1 Tubulin, beta 1
                IPI00116074 Tax_Id=10090 Gene_Symbol=Aco2 Aconitate hydratase, mitochondrial
                IPI00119063 Tax_Id=10090 Gene_Symbol=Lrp1 Prolow-density lipoprotein receptor-related protein 1
```

	IPI_mouse	Decoy	False discovery rate
Peptide matches above identity threshold	161	0	0.00 %
Peptide matches above homology or identity threshold	181	0	0.00 %

Probability Based Mowse Score

Ions score is -10*Log(P), where P is the probability that the observed match is a random event.
Individual ions scores > 37 indicate identity or extensive homology (p<0.01).
Protein scores are derived from ions scores as a non-probabilistic basis for ranking protein hits.

Score Distribution

Peptide Summary Report

Format As | Peptide Summary | Help

Significance threshold p< 0.01 | Max. number of hits AUTO

Standard scoring ○ MudPIT scoring ● | Ions score or expect cut-off 0 | Show sub-sets 0

Show pop-ups ● Suppress pop-ups ○ | Sort unassigned Decreasing Score | Require bold red ☐

Select All | Select None | Search Selected | ☐ **Error tolerant** | Archive Report

1. IPI00420725 **Mass:** 55882 **Score:** 1434 **Queries matched:** 119 **emPAI:** 0.99
Tax_Id=10090 Gene_Symbol=Camk2a Uncharacterized protein
☐ Check to include this hit in error tolerant search or archive report

	Query	Observed	Mr(expt)	Mr(calc)	ppm	Miss	Score	Expect	Rank	Peptide
☑	33	602.3627	601.3554	601.3435	19.8	0	20	1.6	1	K.DLINK.M
☑	81	432.7283	863.4420	863.4501	-9.35	0	(7)	20	1	R.SGAPSVLPH.-
☑	82	432.7314	863.4482	863.4501	-2.17	0	(12)	6.1	1	R.SGAPSVLPH.-
☑	83	432.7319	863.4492	863.4501	-1.01	0	20	0.94	1	R.SGAPSVLPH.-
☑	118	452.2494	902.4842	902.4895	-5.84	0	(13)	5.2	1	K.MLTINPSK.R

☑	119	452.2506	902.4866	902.4895	-3.18	0	15	3.5	1	K.MLTINPSK.R
☑	125	461.2198	920.4250	920.4199	5.57	0	43	0.0033	1	R.TAQSEETR.V
☑	126	463.2444	924.4742	924.4777	-3.74	1	11	5.1	1	R.DHQKLER.E
☑	197	525.2842	1048.5538	1048.5553	-1.37	0	(33)	0.046	1	K.VLAGQEYAAK.I
☑	198	525.2860	1048.5574	1048.5553	2.06	0	(53)	0.0004	1	K.VLAGQEYAAK.I
☑	199	525.2860	1048.5574	1048.5553	2.06	0	55	0.00028	1	K.VLAGQEYAAK.I
☑	200	525.2863	1048.5580	1048.5553	2.64	0	(41)	0.0067	1	K.VLAGQEYAAK.I
☑	201	525.2864	1048.5582	1048.5553	2.83	0	(48)	0.0013	1	K.VLAGQEYAAK.I
☑	202	525.2868	1048.5590	1048.5553	3.59	0	(51)	0.00065	1	K.VLAGQEYAAK.I
	205	530.2988	1058.5830	1058.5906	-7.15	1	(10)	9.3	2	K.MLTINPSKR.I
☑	206	530.2996	1058.5846	1058.5906	-5.64	1	(11)	6.5	1	K.MLTINPSKR.I
☑	207	530.3001	1058.5856	1058.5906	-4.70	1	41	0.0064	1	K.MLTINPSKR.I
☑	208	530.3005	1058.5864	1058.5906	-3.94	1	(40)	0.0086	1	K.MLTINPSKR.I
☑	209	530.3017	1058.5888	1058.5906	-1.68	1	(31)	0.054	1	K.MLTINPSKR.I
☑	210	530.3018	1058.5890	1058.5906	-1.49	1	(14)	3.1	1	K.MLTINPSKR.I
☑	211	530.3021	1058.5896	1058.5906	-0.92	1	(34)	0.028	1	K.MLTINPSKR.I
☑	212	530.3030	1058.5914	1058.5906	0.78	1	(36)	0.017	1	K.MLTINPSKR.I
☑	213	530.3034	1058.5922	1058.5906	1.54	1	(29)	0.09	1	K.MLTINPSKR.I
☑	214	530.3041	1058.5936	1058.5906	2.86	1	(34)	0.031	1	K.MLTINPSKR.I
☑	215	530.3043	1058.5940	1058.5906	3.24	1	(36)	0.017	1	K.MLTINPSKR.I
☑	216	530.3059	1058.5972	1058.5906	6.26	1	(6)	19	1	K.MLTINPSKR.I
	217	530.3068	1058.5990	1058.5906	7.96	1	(5)	23	2	K.MLTINPSKR.I
	220	530.3162	1058.6178	1058.5906	25.7	1	(7)	9.3	2	K.MLTINPSKR.I
☑	280	574.3269	1146.6392	1146.6431	-3.33	0	(48)	0.00085	1	K.GAILTTMLATR.N
☑	281	574.3270	1146.6394	1146.6431	-3.16	0	54	0.00024	1	K.GAILTTMLATR.N
☑	282	574.3282	1146.6418	1146.6431	-1.06	0	(46)	0.0015	1	K.GAILTTMLATR.N
☑	334	631.3043	1260.5940	1260.5927	1.04	0	(33)	0.032	1	R.FYFENLWSR.N
☑	335	631.3046	1260.5946	1260.5927	1.51	0	(54)	0.00029	1	R.FYFENLWSR.N
☑	336	631.3046	1260.5946	1260.5927	1.51	0	(54)	0.00029	1	R.FYFENLWSR.N
☑	337	631.3046	1260.5946	1260.5927	1.51	0	(38)	0.0095	1	R.FYFENLWSR.N
☑	338	631.3051	1260.5956	1260.5927	2.30	0	58	0.00011	1	R.FYFENLWSR.N
☑	339	631.3056	1260.5966	1260.5927	3.10	0	(54)	0.00026	1	R.FYFENLWSR.N
☑	340	631.3057	1260.5968	1260.5927	3.26	0	(54)	0.00029	1	R.FYFENLWSR.N
☑	341	631.3057	1260.5968	1260.5927	3.26	0	(38)	0.012	1	R.FYFENLWSR.N
☑	342	631.3059	1260.5972	1260.5927	3.57	0	(34)	0.025	1	R.FYFENLWSR.N
☑	343	631.3104	1260.6062	1260.5927	10.7	0	(53)	0.00038	1	R.FYFENLWSR.N
☑	344	631.3109	1260.6072	1260.5927	11.5	0	(54)	0.00033	1	R.FYFENLWSR.N
☑	357	652.3538	1302.6930	1302.6932	-0.11	0	(72)	4.3e-006	1	R.ITQYLDAGGIPR.T
☑	358	652.3549	1302.6952	1302.6932	1.58	0	81	5.8e-007	1	R.ITQYLDAGGIPR.T
☑	359	652.3557	1302.6968	1302.6932	2.81	0	(79)	9.7e-007	1	R.ITQYLDAGGIPR.T
☑	360	652.3559	1302.6972	1302.6932	3.11	0	(59)	9.2e-005	1	R.ITQYLDAGGIPR.T
☑	361	652.3559	1302.6972	1302.6932	3.11	0	(55)	0.00023	1	R.ITQYLDAGGIPR.T
☑	362	652.3560	1302.6974	1302.6932	3.27	0	(66)	1.6e-005	1	R.ITQYLDAGGIPR.T
☑	370	670.8879	1339.7612	1339.7711	-7.33	0	(37)	0.0084	1	R.DLKPENLLLASK.L
☑	371	670.8884	1339.7622	1339.7711	-6.59	0	(26)	0.11	1	R.DLKPENLLLASK.L
☑	372	670.8900	1339.7654	1339.7711	-4.20	0	(26)	0.1	1	R.DLKPENLLLASK.L
☑	373	670.8904	1339.7662	1339.7711	-3.60	0	(41)	0.0029	1	R.DLKPENLLLASK.L
☑	374	670.8911	1339.7676	1339.7711	-2.56	0	67	7.6e-006	1	R.DLKPENLLLASK.L
☑	375	670.8915	1339.7684	1339.7711	-1.96	0	(39)	0.0044	1	R.DLKPENLLLASK.L
☑	376	670.8923	1339.7700	1339.7711	-0.76	0	(27)	0.069	1	R.DLKPENLLLASK.L
☑	377	670.8923	1339.7700	1339.7711	-0.76	0	(34)	0.015	1	R.DLKPENLLLASK.L
☑	378	670.8926	1339.7706	1339.7711	-0.32	0	(45)	0.0012	1	R.DLKPENLLLASK.L
☑	379	670.8926	1339.7706	1339.7711	-0.32	0	(39)	0.0046	1	R.DLKPENLLLASK.L
☑	380	670.8930	1339.7714	1339.7711	0.28	0	(36)	0.01	1	R.DLKPENLLLASK.L
☑	381	670.8934	1339.7722	1339.7711	0.88	0	(35)	0.011	1	R.DLKPENLLLASK.L
☑	382	670.8936	1339.7726	1339.7711	1.18	0	(32)	0.026	1	R.DLKPENLLLASK.L
☑	383	670.8938	1339.7730	1339.7711	1.47	0	(28)	0.054	1	R.DLKPENLLLASK.L
☑	384	670.8939	1339.7732	1339.7711	1.62	0	(47)	0.00072	1	R.DLKPENLLLASK.L
	385	670.8940	1339.7734	1339.7711	1.77	0	(1)	30	9	R.DLKPENLLLASK.L
☑	386	670.8944	1339.7742	1339.7711	2.37	0	(32)	0.02	1	R.DLKPENLLLASK.L
☑	387	670.8953	1339.7760	1339.7711	3.71	0	(47)	0.00064	1	R.DLKPENLLLASK.L
☑	388	670.8953	1339.7760	1339.7711	3.71	0	(47)	0.00077	1	R.DLKPENLLLASK.L
☑	389	670.8954	1339.7762	1339.7711	3.86	0	(21)	0.31	1	R.DLKPENLLLASK.L
☑	390	670.8956	1339.7766	1339.7711	4.16	0	(38)	0.006	1	R.DLKPENLLLASK.L
☑	500	816.8917	1631.7688	1631.7719	-1.86	0	(17)	1.2	1	R.FTEEYQLFEELGK.G
☑	501	816.8925	1631.7704	1631.7719	-0.88	0	(49)	0.00075	1	R.FTEEYQLFEELGK.G
☑	503	816.8930	1631.7714	1631.7719	-0.27	0	(52)	0.00039	1	R.FTEEYQLFEELGK.G
☑	504	816.8939	1631.7732	1631.7719	0.84	0	(63)	2.9e-005	1	R.FTEEYQLFEELGK.G
☑	505	816.8942	1631.7738	1631.7719	1.21	0	(36)	0.015	1	R.FTEEYQLFEELGK.G
☑	506	816.8947	1631.7748	1631.7719	1.82	0	(18)	1	1	R.FTEEYQLFEELGK.G
☑	507	816.8947	1631.7748	1631.7719	1.82	0	(60)	6.1e-005	1	R.FTEEYQLFEELGK.G
☑	508	816.8950	1631.7754	1631.7719	2.19	0	(42)	0.0042	1	R.FTEEYQLFEELGK.G
☑	509	816.8950	1631.7754	1631.7719	2.19	0	(59)	8.7e-005	1	R.FTEEYQLFEELGK.G
☑	510	816.8951	1631.7756	1631.7719	2.31	0	(61)	4.6e-005	1	R.FTEEYQLFEELGK.G
☑	511	816.8951	1631.7756	1631.7719	2.31	0	(30)	0.065	1	R.FTEEYQLFEELGK.G
☑	512	816.8953	1631.7760	1631.7719	2.55	0	67	1.2e-005	1	R.FTEEYQLFEELGK.G
☑	513	816.8953	1631.7760	1631.7719	2.55	0	(53)	0.0003	1	R.FTEEYQLFEELGK.G
☑	515	816.8961	1631.7776	1631.7719	3.53	0	(44)	0.0026	1	R.FTEEYQLFEELGK.G
☑	516	816.8964	1631.7782	1631.7719	3.90	0	(30)	0.062	1	R.FTEEYQLFEELGK.G
☑	517	816.8969	1631.7792	1631.7719	4.51	0	(42)	0.0043	1	R.FTEEYQLFEELGK.G
☑	518	816.8969	1631.7792	1631.7719	4.51	0	(48)	0.0011	1	R.FTEEYQLFEELGK.G

520	816.8972	1631.7798	1631.7719	4.88	0	(56)	0.00017	1	R.FTEEYQLFEELGK.G
521	816.8972	1631.7798	1631.7719	4.88	0	(0)	58	9	R.FTEEYQLFEELGK.G
523	816.8978	1631.7810	1631.7719	5.62	0	(52)	0.00042	1	R.FTEEYQLFEELGK.G
524	816.8982	1631.7818	1631.7719	6.11	0	(52)	0.00039	1	R.FTEEYQLFEELGK.G
526	816.9008	1631.7870	1631.7719	9.29	0	(29)	0.082	1	R.FTEEYQLFEELGK.G
528	816.9053	1631.7960	1631.7719	14.8	0	(35)	0.022	1	R.FTEEYQLFEELGK.G
578	694.3150	2079.9232	2079.9426	-9.32	0	75	1e-006	1	K.AGAYDFPSPEWDTVTPEAK.D
579	694.3163	2079.9271	2079.9426	-7.44	0	(64)	1.4e-005	1	K.AGAYDFPSPEWDTVTPEAK.D
580	1040.9812	2079.9478	2079.9426	2.55	0	(28)	0.064	1	K.AGAYDFPSPEWDTVTPEAK.D
581	1040.9817	2079.9488	2079.9426	3.03	0	(51)	0.00036	1	K.AGAYDFPSPEWDTVTPEAK.D
582	1040.9819	2079.9492	2079.9426	3.22	0	(60)	4.1e-005	1	K.AGAYDFPSPEWDTVTPEAK.D
583	1040.9828	2079.9510	2079.9426	4.09	0	(58)	6.4e-005	1	K.AGAYDFPSPEWDTVTPEAK.D
584	1040.9840	2079.9534	2079.9426	5.24	0	(36)	0.01	1	K.AGAYDFPSPEWDTVTPEAK.D
590	715.3455	2143.0147	2143.0321	-8.12	0	60	5.1e-005	1	K.VTEQLIEAISNGDFESYTK.M
657	888.7528	2663.2366	2663.2755	-14.62	1	(19)	0.51	1	K.AGAYDFPSPEWDTVTPEAKDLINK.M
658	888.7558	2663.2456	2663.2755	-11.24	1	(7)	7.8	2	K.AGAYDFPSPEWDTVTPEAKDLINK.M
659	888.7559	2663.2459	2663.2755	-11.12	1	(26)	0.11	1	K.AGAYDFPSPEWDTVTPEAKDLINK.M
660	888.7564	2663.2474	2663.2755	-10.56	1	(47)	0.00075	1	K.AGAYDFPSPEWDTVTPEAKDLINK.M
661	888.7583	2663.2531	2663.2755	-8.42	1	(17)	0.87	1	K.AGAYDFPSPEWDTVTPEAKDLINK.M
662	888.7584	2663.2534	2663.2755	-8.31	1	(63)	1.9e-005	1	K.AGAYDFPSPEWDTVTPEAKDLINK.M
663	888.7586	2663.2540	2663.2755	-8.08	1	(54)	0.00018	1	K.AGAYDFPSPEWDTVTPEAKDLINK.M
664	888.7588	2663.2546	2663.2755	-7.86	1	(63)	2e-005	1	K.AGAYDFPSPEWDTVTPEAKDLINK.M
665	888.7589	2663.2549	2663.2755	-7.74	1	(64)	1.8e-005	1	K.AGAYDFPSPEWDTVTPEAKDLINK.M
666	888.7592	2663.2558	2663.2755	-7.41	1	(26)	0.12	1	K.AGAYDFPSPEWDTVTPEAKDLINK.M
667	888.7593	2663.2561	2663.2755	-7.29	1	(17)	0.85	1	K.AGAYDFPSPEWDTVTPEAKDLINK.M
668	888.7594	2663.2564	2663.2755	-7.18	1	(45)	0.0012	1	K.AGAYDFPSPEWDTVTPEAKDLINK.M
669	888.7597	2663.2573	2663.2755	-6.84	1	80	4e-007	1	K.AGAYDFPSPEWDTVTPEAKDLINK.M
670	888.7598	2663.2576	2663.2755	-6.73	1	(75)	1.2e-006	1	K.AGAYDFPSPEWDTVTPEAKDLINK.M
671	888.7599	2663.2579	2663.2755	-6.62	1	(21)	0.33	1	K.AGAYDFPSPEWDTVTPEAKDLINK.M
672	888.7599	2663.2579	2663.2755	-6.62	1	(4)	15	1	K.AGAYDFPSPEWDTVTPEAKDLINK.M
674	888.7605	2663.2597	2663.2755	-5.94	1	(28)	0.072	1	K.AGAYDFPSPEWDTVTPEAKDLINK.M
675	888.7608	2663.2606	2663.2755	-5.60	1	(67)	8e-006	1	K.AGAYDFPSPEWDTVTPEAKDLINK.M
676	888.7608	2663.2606	2663.2755	-5.60	1	(27)	0.086	1	K.AGAYDFPSPEWDTVTPEAKDLINK.M

Proteins matching the same set of peptides:
IPI00621806 **Mass:** 54651 **Score:** 1434 **Queries matched:** 119
Tax_Id=10090 Gene_Symbol=Camk2a Isoform Alpha CaMKII of Calcium/calmodulin-dependent protein kinase type II subunit alpha

---

2. IPI00474502 **Mass:** 61164 **Score:** 978 **Queries matched:** 66 **emPAI:** 0.78
Tax_Id=10090 Gene_Symbol=Camk2b Calcium/calmodulin-dependent protein kinase type II subunit beta
Check to include this hit in error tolerant search or archive report

Query	Observed	Mr(expt)	Mr(calc)	ppm	Miss	Score	Expect	Rank	Peptide
120	452.7383	903.4620	903.4661	-4.53	0	(11)	7.7	1	K.NSSAITSPK.G
121	452.7405	903.4664	903.4661	0.34	0	(4)	43	3	K.NSSAITSPK.G
122	452.7416	903.4686	903.4661	2.77	0	31	0.089	1	K.NSSAITSPK.G
123	452.7432	903.4718	903.4661	6.32	0	(28)	0.16	1	K.NSSAITSPK.G
126	463.2444	924.4742	924.4777	-3.74	1	11	5.1	1	R.DHQKLER.E
132	469.2222	936.4298	936.4148	16.0	0	26	0.16	1	R.TSQSEETR.V
274	572.8061	1143.5976	1143.5964	1.07	0	(22)	0.51	1	R.FYFENLLAK.N
275	572.8066	1143.5986	1143.5964	1.95	0	(12)	5.4	1	R.FYFENLLAK.N
276	572.8070	1143.5994	1143.5964	2.65	0	23	0.44	1	R.FYFENLLAK.N
277	572.8071	1143.5996	1143.5964	2.82	0	(11)	7.5	1	R.FYFENLLAK.N
278	572.8080	1143.6014	1143.5964	4.40	0	(16)	2.1	1	R.FYFENLLAK.N
280	574.3269	1146.6392	1146.6431	-3.33	0	(48)	0.00085	1	K.GAILTTMLATR.N
281	574.3270	1146.6394	1146.6431	-3.16	0	54	0.00024	1	K.GAILTTMLATR.N
282	574.3282	1146.6418	1146.6431	-1.06	0	(46)	0.0015	1	K.GAILTTMLATR.N
370	670.8879	1339.7612	1339.7711	-7.33	0	(37)	0.0084	1	R.DLKPENLLLASK.C
371	670.8884	1339.7622	1339.7711	-6.59	0	(26)	0.11	1	R.DLKPENLLLASK.C
372	670.8900	1339.7654	1339.7711	-4.20	0	(26)	0.1	1	R.DLKPENLLLASK.C
373	670.8904	1339.7662	1339.7711	-3.60	0	(41)	0.0029	1	R.DLKPENLLLASK.C
374	670.8911	1339.7676	1339.7711	-2.56	0	67	7.6e-006	1	R.DLKPENLLLASK.C
375	670.8915	1339.7684	1339.7711	-1.96	0	(39)	0.0044	1	R.DLKPENLLLASK.C
376	670.8923	1339.7700	1339.7711	-0.76	0	(27)	0.069	1	R.DLKPENLLLASK.C
377	670.8923	1339.7700	1339.7711	-0.76	0	(34)	0.015	1	R.DLKPENLLLASK.C
378	670.8926	1339.7706	1339.7711	-0.32	0	(45)	0.0012	1	R.DLKPENLLLASK.C
379	670.8926	1339.7706	1339.7711	-0.32	0	(39)	0.0046	1	R.DLKPENLLLASK.C
380	670.8930	1339.7714	1339.7711	0.28	0	(36)	0.01	1	R.DLKPENLLLASK.C
381	670.8934	1339.7722	1339.7711	0.88	0	(35)	0.011	1	R.DLKPENLLLASK.C
382	670.8936	1339.7726	1339.7711	1.18	0	(32)	0.026	1	R.DLKPENLLLASK.C
383	670.8938	1339.7730	1339.7711	1.47	0	(28)	0.054	1	R.DLKPENLLLASK.C
384	670.8939	1339.7732	1339.7711	1.62	0	(47)	0.00072	1	R.DLKPENLLLASK.C
385	670.8940	1339.7734	1339.7711	1.77	0	(1)	30	9	R.DLKPENLLLASK.C
386	670.8944	1339.7742	1339.7711	2.37	0	(32)	0.02	1	R.DLKPENLLLASK.C
387	670.8953	1339.7760	1339.7711	3.71	0	(47)	0.00064	1	R.DLKPENLLLASK.C
388	670.8953	1339.7760	1339.7711	3.71	0	(47)	0.00077	1	R.DLKPENLLLASK.C
389	670.8954	1339.7762	1339.7711	3.86	0	(21)	0.31	1	R.DLKPENLLLASK.C
390	670.8956	1339.7766	1339.7711	4.16	0	(38)	0.006	1	R.DLKPENLLLASK.C
414	702.3716	1402.7286	1402.7317	-2.18	0	(2)	41	1	R.LTQYIDGQGRPR.T
415	702.3759	1402.7372	1402.7317	3.95	0	5	23	2	R.LTQYIDGQGRPR.T
429	735.4117	1468.8088	1468.8071	1.16	0	(58)	6.9e-005	1	K.NLINQMLTINPAK.R
430	735.4119	1468.8092	1468.8071	1.43	0	(63)	2.2e-005	1	K.NLINQMLTINPAK.R
431	735.4133	1468.8120	1468.8071	3.34	0	(74)	2e-006	1	K.NLINQMLTINPAK.R
432	735.4143	1468.8140	1468.8071	4.70	0	76	1.2e-006	1	K.NLINQMLTINPAK.R

	Query	Observed	Mr(expt)	Mr(calc)	ppm	Miss	Score	Expect	Rank	Peptide
☑	539	871.3708	1740.7270	1740.7173	5.57	0	(7)	3.6	1	K.ESSDSTNTTIEDEDAK.A
☑	540	871.3715	1740.7284	1740.7173	6.38	0	17	0.37	1	K.ESSDSTNTTIEDEDAK.A
☑	572	656.9528	1967.8366	1967.8556	-9.65	1	94	8.3e-009	1	K.ESSDSTNTTIEDEDAKAR.K
	578	694.3150	2079.9232	2079.9426	-9.32	0	75	1e-006	1	K.AGAYDFPSPEWDTVTPEAK.N
	579	694.3163	2079.9271	2079.9426	-7.44	0	(64)	1.4e-005	1	K.AGAYDFPSPEWDTVTPEAK.N
	580	1040.9812	2079.9478	2079.9426	2.55	0	(28)	0.064	1	K.AGAYDFPSPEWDTVTPEAK.N
	581	1040.9817	2079.9488	2079.9426	3.03	0	(51)	0.00036	1	K.AGAYDFPSPEWDTVTPEAK.N
	582	1040.9819	2079.9492	2079.9426	3.22	0	(60)	4.1e-005	1	K.AGAYDFPSPEWDTVTPEAK.N
	583	1040.9828	2079.9510	2079.9426	4.09	0	(58)	6.4e-005	1	K.AGAYDFPSPEWDTVTPEAK.N
	584	1040.9840	2079.9534	2079.9426	5.24	0	(36)	0.01	1	K.AGAYDFPSPEWDTVTPEAK.N
☑	588	705.0029	2111.9869	2112.0011	-6.74	0	71	3.9e-006	1	K.TTEQLIEAVNNGDFEAYAK.I
☑	634	779.7132	2336.1178	2336.1325	-6.29	1	17	0.81	1	R.FTDEYQLYEDIGKGAFSVVR.R
☑	635	779.7135	2336.1187	2336.1325	-5.91	1	(9)	6	1	R.FTDEYQLYEDIGKGAFSVVR.R
☑	637	779.7166	2336.1280	2336.1325	-1.93	1	(15)	1.4	1	R.FTDEYQLYEDIGKGAFSVVR.R
☑	643	785.0448	2352.1126	2352.1301	-7.46	0	(89)	4.8e-008	1	R.QTTAPATMSTAASGTTMGLVEQAK.S
☑	644	785.0455	2352.1147	2352.1301	-6.56	0	(88)	6.7e-008	1	R.QTTAPATMSTAASGTTMGLVEQAK.S
☑	645	1177.0762	2352.1378	2352.1301	3.29	0	(96)	1e-008	1	R.QTTAPATMSTAASGTTMGLVEQAK.S
☑	646	1177.0767	2352.1388	2352.1301	3.71	0	98	7.3e-009	1	R.QTTAPATMSTAASGTTMGLVEQAK.S
☑	647	785.4142	2353.2208	2353.2642	-18.44	0	(35)	0.013	1	K.GSLPPAALEPQTTVIHNPVDGIK.E
☑	648	785.4187	2353.2343	2353.2642	-12.70	0	64	1.6e-005	1	K.GSLPPAALEPQTTVIHNPVDGIK.E
☑	649	785.4208	2353.2406	2353.2642	-10.02	0	(62)	2.2e-005	1	K.GSLPPAALEPQTTVIHNPVDGIK.E
☑	650	785.4230	2353.2472	2353.2642	-7.22	0	(44)	0.0014	1	K.GSLPPAALEPQTTVIHNPVDGIK.E
☑	651	785.4244	2353.2514	2353.2642	-5.43	0	(40)	0.0036	1	K.GSLPPAALEPQTTVIHNPVDGIK.E
☑	652	790.3753	2368.1041	2368.1250	-8.85	0	(85)	1.2e-007	1	R.QTTAPATMSTAASGTTMGLVEQAK.S + Oxidation (M)
☑	653	790.3780	2368.1122	2368.1250	-5.43	0	(79)	4.5e-007	1	R.QTTAPATMSTAASGTTMGLVEQAK.S + Oxidation (M)

Proteins matching the same set of peptides:
IPI00875723 **Mass:** 68686 **Score:** 978 **Queries matched:** 66
Tax_Id=10090 Gene_Symbol=Camk2b 68 kDa protein

---

3. IPI00112584 **Mass:** 56961 **Score:** 636 **Queries matched:** 57 **emPAI:** 0.48
Tax_Id=10090 Gene_Symbol=Camk2d Isoform 1 of Calcium/calmodulin-dependent protein kinase type II subunit delta
☐ Check to include this hit in error tolerant search or archive report

	Query	Observed	Mr(expt)	Mr(calc)	ppm	Miss	Score	Expect	Rank	Peptide
	33	602.3627	601.3554	601.3435	19.8	0	20	1.6	1	K.DLINK.M
	126	463.2444	924.4742	924.4777	-3.74	1	11	5.1	1	R.DHQKLER.E
☑	241	539.2839	1076.5532	1076.5502	2.83	0	10	8.9	1	K.IPTGQEYAAK.I
	280	574.3269	1146.6392	1146.6431	-3.33	0	(48)	0.00085	1	K.GAILTTMLATR.N
	281	574.3270	1146.6394	1146.6431	-3.16	0	54	0.00024	1	K.GAILTTMLATR.N
	282	574.3282	1146.6418	1146.6431	-1.06	0	(46)	0.0015	1	K.GAILTTMLATR.N
☑	366	664.3086	1326.6026	1326.5948	5.91	0	59	6.2e-005	1	R.LTQYMDGSGMPK.T
☑	367	664.3100	1326.6054	1326.5948	8.02	0	(46)	0.0015	1	R.LTQYMDGSGMPK.T
	370	670.8879	1339.7612	1339.7711	-7.33	0	(37)	0.0084	1	R.DLKPENLLLASK.S
	371	670.8884	1339.7622	1339.7711	-6.59	0	(26)	0.11	1	R.DLKPENLLLASK.S
	372	670.8900	1339.7654	1339.7711	-4.20	0	(26)	0.1	1	R.DLKPENLLLASK.S
	373	670.8904	1339.7662	1339.7711	-3.60	0	(41)	0.0029	1	R.DLKPENLLLASK.S
	374	670.8911	1339.7676	1339.7711	-2.56	0	67	7.6e-006	1	R.DLKPENLLLASK.S
	375	670.8915	1339.7684	1339.7711	-1.96	0	(39)	0.0044	1	R.DLKPENLLLASK.S
	376	670.8923	1339.7700	1339.7711	-0.76	0	(27)	0.069	1	R.DLKPENLLLASK.S
	377	670.8923	1339.7700	1339.7711	-0.76	0	(34)	0.015	1	R.DLKPENLLLASK.S
	378	670.8926	1339.7706	1339.7711	-0.32	0	(45)	0.0012	1	R.DLKPENLLLASK.S
	379	670.8926	1339.7706	1339.7711	-0.32	0	(39)	0.0046	1	R.DLKPENLLLASK.S
	380	670.8930	1339.7714	1339.7711	0.28	0	(36)	0.01	1	R.DLKPENLLLASK.S
	381	670.8934	1339.7722	1339.7711	0.88	0	(35)	0.011	1	R.DLKPENLLLASK.S
	382	670.8936	1339.7726	1339.7711	1.18	0	(32)	0.026	1	R.DLKPENLLLASK.S
	383	670.8938	1339.7730	1339.7711	1.47	0	(28)	0.054	1	R.DLKPENLLLASK.S
	384	670.8939	1339.7732	1339.7711	1.62	0	(47)	0.00072	1	R.DLKPENLLLASK.S
	385	670.8940	1339.7734	1339.7711	1.77	0	(1)	30	9	R.DLKPENLLLASK.S
	386	670.8944	1339.7742	1339.7711	2.37	0	(32)	0.02	1	R.DLKPENLLLASK.S
	387	670.8953	1339.7760	1339.7711	3.71	0	(47)	0.00064	1	R.DLKPENLLLASK.S
	388	670.8953	1339.7760	1339.7711	3.71	0	(47)	0.00077	1	R.DLKPENLLLASK.S
	389	670.8954	1339.7762	1339.7711	3.86	0	(21)	0.31	1	R.DLKPENLLLASK.S
	390	670.8956	1339.7766	1339.7711	4.16	0	(38)	0.006	1	R.DLKPENLLLASK.S
☑	492	809.8878	1617.7610	1617.7562	2.97	0	64	2e-005	1	R.FTDEYQLFEELGK.G
☑	493	809.8892	1617.7638	1617.7562	4.70	0	(49)	0.00078	1	R.FTDEYQLFEELGK.G
	578	694.3150	2079.9232	2079.9426	-9.32	0	75	1e-006	1	K.AGAYDFPSPEWDTVTPEAK.D
	579	694.3163	2079.9271	2079.9426	-7.44	0	(64)	1.4e-005	1	K.AGAYDFPSPEWDTVTPEAK.D
	580	1040.9812	2079.9478	2079.9426	2.55	0	(28)	0.064	1	K.AGAYDFPSPEWDTVTPEAK.D
	581	1040.9817	2079.9488	2079.9426	3.03	0	(51)	0.00036	1	K.AGAYDFPSPEWDTVTPEAK.D
	582	1040.9819	2079.9492	2079.9426	3.22	0	(60)	4.1e-005	1	K.AGAYDFPSPEWDTVTPEAK.D
	583	1040.9828	2079.9510	2079.9426	4.09	0	(58)	6.4e-005	1	K.AGAYDFPSPEWDTVTPEAK.D
	584	1040.9840	2079.9534	2079.9426	5.24	0	(36)	0.01	1	K.AGAYDFPSPEWDTVTPEAK.D
	657	888.7528	2663.2366	2663.2755	-14.62	1	(19)	0.51	1	K.AGAYDFPSPEWDTVTPEAKDLINK.M
	658	888.7558	2663.2456	2663.2755	-11.24	1	(7)	7.8	2	K.AGAYDFPSPEWDTVTPEAKDLINK.M
	659	888.7559	2663.2459	2663.2755	-11.12	1	(26)	0.11	1	K.AGAYDFPSPEWDTVTPEAKDLINK.M
	660	888.7564	2663.2474	2663.2755	-10.56	1	(47)	0.00075	1	K.AGAYDFPSPEWDTVTPEAKDLINK.M
	661	888.7583	2663.2531	2663.2755	-8.42	1	(17)	0.87	1	K.AGAYDFPSPEWDTVTPEAKDLINK.M
	662	888.7584	2663.2534	2663.2755	-8.31	1	(63)	1.9e-005	1	K.AGAYDFPSPEWDTVTPEAKDLINK.M
	663	888.7586	2663.2540	2663.2755	-8.08	1	(54)	0.00018	1	K.AGAYDFPSPEWDTVTPEAKDLINK.M
	664	888.7588	2663.2546	2663.2755	-7.86	1	(63)	2e-005	1	K.AGAYDFPSPEWDTVTPEAKDLINK.M
	665	888.7589	2663.2549	2663.2755	-7.74	1	(64)	1.8e-005	1	K.AGAYDFPSPEWDTVTPEAKDLINK.M
	666	888.7592	2663.2558	2663.2755	-7.41	1	(26)	0.12	1	K.AGAYDFPSPEWDTVTPEAKDLINK.M
	667	888.7593	2663.2561	2663.2755	-7.29	1	(17)	0.85	1	K.AGAYDFPSPEWDTVTPEAKDLINK.M
	668	888.7594	2663.2564	2663.2755	-7.18	1	(45)	0.0012	1	K.AGAYDFPSPEWDTVTPEAKDLINK.M
	669	888.7597	2663.2573	2663.2755	-6.84	1	80	4e-007	1	K.AGAYDFPSPEWDTVTPEAKDLINK.M
	670	888.7598	2663.2576	2663.2755	-6.73	1	(75)	1.2e-006	1	K.AGAYDFPSPEWDTVTPEAKDLINK.M

	Query	Observed	Mr(expt)	Mr(calc)	ppm	Miss	Score	Expect	Rank	Peptide
	671	888.7599	2663.2579	2663.2755	-6.62	1	(21)	0.33	1	K.AGAYDFPSPEWDTVTPEAKDLINK.M
	672	888.7599	2663.2579	2663.2755	-6.62	1	(4)	15	1	K.AGAYDFPSPEWDTVTPEAKDLINK.M
	674	888.7605	2663.2597	2663.2755	-5.94	1	(28)	0.072	1	K.AGAYDFPSPEWDTVTPEAKDLINK.M
	675	888.7608	2663.2606	2663.2755	-5.60	1	(67)	8e-006	1	K.AGAYDFPSPEWDTVTPEAKDLINK.M
	676	888.7608	2663.2606	2663.2755	-5.60	1	(27)	0.086	1	K.AGAYDFPSPEWDTVTPEAKDLINK.M

Proteins matching the same set of peptides:
IPI00406790 **Mass:** 60593 **Score:** 636 **Queries matched:** 57
Tax_Id=10090 Gene_Symbol=Camk2d Isoform 4 of Calcium/calmodulin-dependent protein kinase type II subunit delta
IPI00475044 **Mass:** 55899 **Score:** 636 **Queries matched:** 57
Tax_Id=10090 Gene_Symbol=Camk2d Isoform 2 of Calcium/calmodulin-dependent protein kinase type II subunit delta
IPI00828919 **Mass:** 58492 **Score:** 636 **Queries matched:** 57
Tax_Id=10090 Gene_Symbol=Camk2d Isoform 3 of Calcium/calmodulin-dependent protein kinase type II subunit delta
IPI00857865 **Mass:** 54664 **Score:** 636 **Queries matched:** 57
Tax_Id=10090 Gene_Symbol=Camk2d Putative uncharacterized protein
IPI00858128 **Mass:** 58283 **Score:** 636 **Queries matched:** 57
Tax_Id=10090 Gene_Symbol=Camk2d calcium/calmodulin-dependent protein kinase type II subunit delta isoform 1
IPI00858144 **Mass:** 55828 **Score:** 636 **Queries matched:** 57
Tax_Id=10090 Gene_Symbol=Camk2d 55 kDa protein

---

4. IPI00124695 **Mass:** 60254 **Score:** 516 **Queries matched:** 39 **emPAI:** 0.30
Tax_Id=10090 Gene_Symbol=Camk2g Isoform 1 of Calcium/calmodulin-dependent protein kinase type II subunit gamma

☐ Check to include this hit in error tolerant search or archive report

	Query	Observed	Mr(expt)	Mr(calc)	ppm	Miss	Score	Expect	Rank	Peptide
	126	463.2444	924.4742	924.4777	-3.74	1	11	5.1	1	R.DHQKLER.E
	132	469.2222	936.4298	936.4148	16.0	0	26	0.16	1	R.TSQSEETR.V
☑	165	**499.7433**	**997.4720**	**997.4716**	**0.43**	**0**	**23**	**0.27**	**1**	**K.TSTQEYAAK.I**
	370	670.8879	1339.7612	1339.7711	-7.33	0	(37)	0.0084	1	R.DLKPENLLLASK.C
	371	670.8884	1339.7622	1339.7711	-6.59	0	(26)	0.11	1	R.DLKPENLLLASK.C
	372	670.8900	1339.7654	1339.7711	-4.20	0	(26)	0.1	1	R.DLKPENLLLASK.C
	373	670.8904	1339.7662	1339.7711	-3.60	0	(41)	0.0029	1	R.DLKPENLLLASK.C
	374	670.8911	1339.7676	1339.7711	-2.56	0	67	7.6e-006	1	R.DLKPENLLLASK.C
	375	670.8915	1339.7684	1339.7711	-1.96	0	(39)	0.0044	1	R.DLKPENLLLASK.C
	376	670.8923	1339.7700	1339.7711	-0.76	0	(27)	0.069	1	R.DLKPENLLLASK.C
	377	670.8923	1339.7700	1339.7711	-0.76	0	(34)	0.015	1	R.DLKPENLLLASK.C
	378	670.8926	1339.7706	1339.7711	-0.32	0	(45)	0.0012	1	R.DLKPENLLLASK.C
	379	670.8926	1339.7706	1339.7711	-0.32	0	(39)	0.0046	1	R.DLKPENLLLASK.C
	380	670.8930	1339.7714	1339.7711	0.28	0	(36)	0.01	1	R.DLKPENLLLASK.C
	381	670.8934	1339.7722	1339.7711	0.88	0	(35)	0.011	1	R.DLKPENLLLASK.C
	382	670.8936	1339.7726	1339.7711	1.18	0	(32)	0.026	1	R.DLKPENLLLASK.C
	383	670.8938	1339.7730	1339.7711	1.47	0	(28)	0.054	1	R.DLKPENLLLASK.C
	384	670.8939	1339.7732	1339.7711	1.62	0	(47)	0.00072	1	R.DLKPENLLLASK.C
	385	670.8940	1339.7734	1339.7711	1.77	0	(1)	30	9	R.DLKPENLLLASK.C
	386	670.8944	1339.7742	1339.7711	2.37	0	(32)	0.02	1	R.DLKPENLLLASK.C
	387	670.8953	1339.7760	1339.7711	3.71	0	(47)	0.00064	1	R.DLKPENLLLASK.C
	388	670.8953	1339.7760	1339.7711	3.71	0	(47)	0.00077	1	R.DLKPENLLLASK.C
	389	670.8954	1339.7762	1339.7711	3.86	0	(21)	0.31	1	R.DLKPENLLLASK.C
	390	670.8956	1339.7766	1339.7711	4.16	0	(38)	0.006	1	R.DLKPENLLLASK.C
	414	702.3716	1402.7286	1402.7317	-2.18	0	(2)	41	1	R.LTQYIDGQGRPR.T
	415	702.3759	1402.7372	1402.7317	3.95	0	5	23	2	R.LTQYIDGQGRPR.T
	429	735.4117	1468.8088	1468.8071	1.16	0	(58)	6.9e-005	1	K.NLINQMLTINPAK.R
	430	735.4119	1468.8092	1468.8071	1.43	0	(63)	2.2e-005	1	K.NLINQMLTINPAK.R
	431	735.4133	1468.8120	1468.8071	3.34	0	(74)	2e-006	1	K.NLINQMLTINPAK.R
	432	735.4143	1468.8140	1468.8071	4.70	0	76	1.2e-006	1	K.NLINQMLTINPAK.R
☑	484	**802.8825**	**1603.7504**	**1603.7406**	**6.14**	**0**	**(68)**	**9.3e-006**	**1**	**R.FTDDYQLFEELGK.G**
☑	485	**802.8837**	**1603.7528**	**1603.7406**	**7.64**	**0**	**69**	**7.7e-006**	**1**	**R.FTDDYQLFEELGK.G**
	578	694.3150	2079.9232	2079.9426	-9.32	0	75	1e-006	1	K.AGAYDFPSPEWDTVTPEAK.N
	579	694.3163	2079.9271	2079.9426	-7.44	0	(64)	1.4e-005	1	K.AGAYDFPSPEWDTVTPEAK.N
	580	1040.9812	2079.9478	2079.9426	2.55	0	(28)	0.064	1	K.AGAYDFPSPEWDTVTPEAK.N
	581	1040.9817	2079.9488	2079.9426	3.03	0	(51)	0.00036	1	K.AGAYDFPSPEWDTVTPEAK.N
	582	1040.9819	2079.9492	2079.9426	3.22	0	(60)	4.1e-005	1	K.AGAYDFPSPEWDTVTPEAK.N
	583	1040.9828	2079.9510	2079.9426	4.09	0	(58)	6.4e-005	1	K.AGAYDFPSPEWDTVTPEAK.N
	584	1040.9840	2079.9534	2079.9426	5.24	0	(36)	0.01	1	K.AGAYDFPSPEWDTVTPEAK.N

Proteins matching the same set of peptides:
IPI00228044 **Mass:** 59013 **Score:** 516 **Queries matched:** 39
Tax_Id=10090 Gene_Symbol=Camk2g Isoform 2 of Calcium/calmodulin-dependent protein kinase type II subunit gamma
IPI00228045 **Mass:** 56610 **Score:** 516 **Queries matched:** 39
Tax_Id=10090 Gene_Symbol=Camk2g Isoform 3 of Calcium/calmodulin-dependent protein kinase type II subunit gamma

---

5. IPI00109073 **Mass:** 50010 **Score:** 259 **Queries matched:** 11 **emPAI:** 0.29
Tax_Id=10090 Gene_Symbol=Tubb4 Tubulin beta-4 chain

☐ Check to include this hit in error tolerant search or archive report

	Query	Observed	Mr(expt)	Mr(calc)	ppm	Miss	Score	Expect	Rank	Peptide
☑	273	**572.3212**	**1142.6278**	**1142.6270**	**0.72**	**0**	**42**	**0.0049**	**1**	**K.LAVNMVPFPR.L**
	317	**615.2972**	**1228.5798**	**1228.5910**	**-9.10**	**0**	**1**	**43**	**2**	**R.ISEQFTAMFR.R**
☑	364	**660.3562**	**1318.6978**	**1318.6955**	**1.77**	**0**	**85**	**2.5e-007**	**1**	**R.IMNTFSVVPSPK.V**
☑	365	**660.3578**	**1318.7010**	**1318.6955**	**4.20**	**0**	**(84)**	**2.7e-007**	**1**	**R.IMNTFSVVPSPK.V**
☑	481	**801.4144**	**1600.8142**	**1600.8131**	**0.74**	**0**	**82**	**3.6e-007**	**1**	**R.AVLVDLEPGTMDSVR.S**
☑	482	**801.4154**	**1600.8162**	**1600.8131**	**1.99**	**0**	**(67)**	**1.2e-005**	**1**	**R.AVLVDLEPGTMDSVR.S**
☑	483	**801.4161**	**1600.8176**	**1600.8131**	**2.86**	**0**	**(60)**	**5.9e-005**	**1**	**R.AVLVDLEPGTMDSVR.S**
☑	532	**846.4391**	**1690.8636**	**1690.8600**	**2.16**	**0**	**(20)**	**0.66**	**1**	**R.ALTVPELTQQMFDAK.N**
☑	533	**846.4404**	**1690.8662**	**1690.8600**	**3.69**	**0**	**40**	**0.0061**	**1**	**R.ALTVPELTQQMFDAK.N**
☑	534	**848.9236**	**1695.8326**	**1695.8257**	**4.12**	**0**	**15**	**2.1**	**1**	**K.NSSYFVEWIPNNVK.T**
	537	**854.4365**	**1706.8584**	**1706.8549**	**2.07**	**0**	**(30)**	**0.064**	**2**	**R.ALTVPELTQQMFDAK.N + Oxidation (M)**

Proteins matching the same set of peptides:
IPI00169463 **Mass:** 50255 **Score:** 259 **Queries matched:** 11
Tax_Id=10090 Gene_Symbol=Tubb2c Tubulin beta-2C chain

---

6. IPI00115546 **Mass:** 40524 **Score:** 227 **Queries matched:** 5 **emPAI:** 0.27
Tax_Id=10090 Gene_Symbol=Gnao1 Isoform Alpha-2 of Guanine nucleotide-binding protein G(o) subunit alpha
☐ Check to include this hit in error tolerant search or archive report

	Query	Observed	Mr(expt)	Mr(calc)	ppm	Miss	Score	Expect	Rank	Peptide
☑	203	529.3122	1056.6098	1056.6179	-7.61	0	(18)	0.98	1	K.LLLLGAGESGK.S
☑	204	529.3124	1056.6102	1056.6179	-7.23	0	44	0.0026	1	K.LLLLGAGESGK.S
☑	531	838.4189	1674.8232	1674.8213	1.16	0	113	3.4e-010	1	R.IGAGDYQPTEQDILR.T
☑	570	979.4428	1956.8710	1956.8631	4.07	0	87	7.1e-008	1	R.MEDTEPFSAELLSAMMR.L
☑	571	979.4498	1956.8850	1956.8631	11.2	0	(83)	1.8e-007	1	R.MEDTEPFSAELLSAMMR.L

Proteins matching the same set of peptides:
IPI00230192 **Mass:** 40629 **Score:** 227 **Queries matched:** 5
Tax_Id=10090 Gene_Symbol=Gnao1 Isoform Alpha-1 of Guanine nucleotide-binding protein G(o) subunit alpha

---

7. IPI00323571 **Mass:** 35901 **Score:** 226 **Queries matched:** 18 **emPAI:** 0.86
Tax_Id=10090 Gene_Symbol=Apoe Apolipoprotein E
☐ Check to include this hit in error tolerant search or archive report

	Query	Observed	Mr(expt)	Mr(calc)	ppm	Miss	Score	Expect	Rank	Peptide
☑	152	484.7774	967.5402	967.5451	-4.99	0	(50)	0.00041	1	R.LGPLVEQGR.Q
☑	153	484.7776	967.5406	967.5451	-4.57	0	52	0.00027	1	R.LGPLVEQGR.Q
☑	155	487.7528	973.4910	973.4828	8.42	1	46	0.0023	1	R.DAEDLQKR.L
☑	184	510.2458	1018.4770	1018.4753	1.69	0	56	0.00017	1	R.LGADMEDLR.N
☑	185	510.2510	1018.4874	1018.4753	11.9	0	(43)	0.0033	1	R.LGADMEDLR.N
☑	187	510.2557	1018.4968	1018.4906	6.16	0	15	2.6	1	R.QWANLMEK.I
☑	238	538.2985	1074.5824	1074.5822	0.26	0	(40)	0.0082	1	R.LQAEIFQAR.L
☑	239	538.2990	1074.5834	1074.5822	1.19	0	52	0.00055	1	R.LQAEIFQAR.L
☑	240	538.3007	1074.5868	1074.5822	4.35	0	(36)	0.021	1	R.LQAEIFQAR.L
☑	287	581.7828	1161.5510	1161.5448	5.38	0	(42)	0.0051	1	K.MEEQTQQIR.L
☑	288	581.7836	1161.5526	1161.5448	6.76	0	(42)	0.0053	1	K.MEEQTQQIR.L
☑	289	581.7837	1161.5528	1161.5448	6.93	0	(24)	0.3	1	K.MEEQTQQIR.L
☑	290	581.7856	1161.5566	1161.5448	10.2	0	49	0.001	1	K.MEEQTQQIR.L
☑	479	800.4014	1598.7882	1598.7787	5.94	0	63	2.9e-005	1	K.ELEEQLGPVAEETR.A
☑	480	800.4103	1598.8060	1598.7787	17.1	0	(54)	0.00027	1	K.ELEEQLGPVAEETR.A
☑	573	997.5337	1993.0528	1993.0480	2.43	0	25	0.14	1	K.IQASVATNPIITPVAQENQ.-
☑	702	1308.9537	3923.8393	3923.8656	-6.72	0	32	0.01	1	R.WVQTLSDQVQEELQSSQVTQELTALMEDTMTEVK.A
☑	705	1308.9666	3923.8780	3923.8656	3.15	0	(10)	1.8	1	R.WVQTLSDQVQEELQSSQVTQELTALMEDTMTEVK.A

---

8. IPI00112251 **Mass:** 50842 **Score:** 198 **Queries matched:** 12 **emPAI:** 0.29
Tax_Id=10090 Gene_Symbol=Tubb3 Tubulin beta-3 chain
☐ Check to include this hit in error tolerant search or archive report

	Query	Observed	Mr(expt)	Mr(calc)	ppm	Miss	Score	Expect	Rank	Peptide
	273	572.3212	1142.6278	1142.6270	0.72	0	42	0.0049	1	K.LAVNMVPFPR.L
	317	615.2972	1228.5798	1228.5910	-9.10	0	1	43	2	R.ISEQFTAMFR.R
	364	660.3562	1318.6978	1318.6955	1.77	0	85	2.5e-007	1	R.IMNTFSVVPSPK.V
	365	660.3578	1318.7010	1318.6955	4.20	0	(84)	2.7e-007	1	R.IMNTFSVVPSPK.V
☑	486	808.4135	1614.8124	1614.8287	-10.07	0	(41)	0.0062	1	R.AILVDLEPGTMDSVR.S
☑	487	808.4182	1614.8218	1614.8287	-4.25	0	(35)	0.026	1	R.AILVDLEPGTMDSVR.S
☑	488	808.4218	1614.8290	1614.8287	0.21	0	71	6.3e-006	1	R.AILVDLEPGTMDSVR.S
☑	497	816.4215	1630.8284	1630.8236	2.96	0	(31)	0.059	1	R.AILVDLEPGTMDSVR.S + Oxidation (M)
	532	846.4391	1690.8636	1690.8600	2.16	0	(20)	0.66	1	R.ALTVPELTQQMFDAK.N
	533	846.4404	1690.8662	1690.8600	3.69	0	40	0.0061	1	R.ALTVPELTQQMFDAK.N
	534	848.9236	1695.8326	1695.8257	4.12	0	15	2.1	1	K.NSSYFVEWIPNNVK.V
	537	854.4365	1706.8584	1706.8549	2.07	0	(30)	0.064	2	R.ALTVPELTQQMFDAK.N + Oxidation (M)

---

9. IPI00110850 **Mass:** 42052 **Score:** 175 **Queries matched:** 9 **emPAI:** 0.35
Tax_Id=10090 Gene_Symbol=Actb Actin, cytoplasmic 1
☐ Check to include this hit in error tolerant search or archive report

	Query	Observed	Mr(expt)	Mr(calc)	ppm	Miss	Score	Expect	Rank	Peptide
☑	285	581.3162	1160.6178	1160.6111	5.83	0	(27)	0.17	1	K.EITALAPSTMK.I
☑	286	581.3204	1160.6262	1160.6111	13.1	0	36	0.018	1	K.EITALAPSTMK.I
☑	307	599.7695	1197.5244	1197.5150	7.93	0	41	0.0029	1	K.DSYVGDEAQSK.R
☑	550	895.9509	1789.8872	1789.8846	1.46	0	85	2e-007	1	K.SYELPDGQVITIGNER.F
☑	551	895.9536	1789.8926	1789.8846	4.48	0	(73)	3.1e-006	1	K.SYELPDGQVITIGNER.F
☑	566	652.0146	1953.0220	1953.0571	-17.99	0	(34)	0.019	1	R.VAPEEHPVLLTEAPLNPK.A
☑	567	652.0152	1953.0238	1953.0571	-17.06	0	52	0.00028	1	R.VAPEEHPVLLTEAPLNPK.A
☑	568	652.0201	1953.0385	1953.0571	-9.54	0	(32)	0.028	1	R.VAPEEHPVLLTEAPLNPK.A
☑	569	652.0240	1953.0502	1953.0571	-3.55	0	(38)	0.0068	1	R.VAPEEHPVLLTEAPLNPK.A

Proteins matching the same set of peptides:
IPI00136929 **Mass:** 44029 **Score:** 175 **Queries matched:** 9
Tax_Id=10090 Gene_Symbol=Actg1 Gamma actin-like protein
IPI00473320 **Mass:** 42115 **Score:** 175 **Queries matched:** 9

Tax_Id=10090 Gene_Symbol=Actb Putative uncharacterized protein
IPI00652436 **Mass:** 42053 **Score:** 175 **Queries matched:** 9
Tax_Id=10090 Gene_Symbol=Actg1 Putative uncharacterized protein
IPI00874482 **Mass:** 42108 **Score:** 175 **Queries matched:** 9
Tax_Id=10090 Gene_Symbol=Actg1 Actin, cytoplasmic 2

---

10. IPI00109061 **Mass:** 50377 **Score:** 175 **Queries matched:** 10 **emPAI:** 0.29
Tax_Id=10090 Gene_Symbol=Tubb2b Tubulin beta-2B chain
☐ Check to include this hit in error tolerant search or archive report

	Query	Observed	Mr(expt)	Mr(calc)	ppm	Miss	Score	Expect	Rank	Peptide
	273	572.3212	1142.6278	1142.6270	0.72	0	42	0.0049	1	K.LAVNMVPFPR.L
	317	615.2972	1228.5798	1228.5910	-9.10	0	1	43	2	R.ISEQFTAMFR.R
☑	397	676.3429	1350.6712	1350.6676	2.72	0	(67)	1.4e-005	1	R.IMNTFSVMPSPK.V
☑	398	676.3460	1350.6774	1350.6676	7.31	0	79	1e-006	1	R.IMNTFSVMPSPK.V
	486	808.4135	1614.8124	1614.8287	-10.07	0	(41)	0.0062	1	R.AILVDLEPGTMDSVR.S
	487	808.4182	1614.8218	1614.8287	-4.25	0	(35)	0.026	1	R.AILVDLEPGTMDSVR.S
	488	808.4218	1614.8290	1614.8287	0.21	0	71	6.3e-006	1	R.AILVDLEPGTMDSVR.S
	497	816.4215	1630.8284	1630.8236	2.96	0	(31)	0.059	1	R.AILVDLEPGTMDSVR.S + Oxidation (M)
	534	848.9236	1695.8326	1695.8257	4.12	0	15	2.1	1	K.NSSYFVEWIPNNVK.T
☑	537	854.4365	1706.8584	1706.8549	2.07	0	43	0.0034	1	R.ALTVPELTQQMFDSK.N

Proteins matching the same set of peptides:
IPI00338039 **Mass:** 50274 **Score:** 175 **Queries matched:** 10
Tax_Id=10090 Gene_Symbol=Tubb2a Tubulin beta-2A chain

---

11. IPI00122048 **Mass:** 113045 **Score:** 171 **Queries matched:** 5 **emPAI:** 0.09
Tax_Id=10090 Gene_Symbol=Atp1a3 Sodium/potassium-transporting ATPase subunit alpha-3
☐ Check to include this hit in error tolerant search or archive report

	Query	Observed	Mr(expt)	Mr(calc)	ppm	Miss	Score	Expect	Rank	Peptide
☑	318	618.8549	1235.6952	1235.6986	-2.72	0	59	4.9e-005	1	R.LNIPVSQVNPR.D
☑	319	618.8561	1235.6976	1235.6986	-0.78	0	(36)	0.011	1	R.LNIPVSQVNPR.D
☑	556	906.4708	1810.9270	1810.9425	-8.54	0	(1)	51	1	R.QGAIVAVTGDGVNDSPALK.K
☑	557	906.4775	1810.9404	1810.9425	-1.14	0	70	6.2e-006	1	R.QGAIVAVTGDGVNDSPALK.K
☑	560	915.4684	1828.9222	1828.9167	3.06	0	96	1.3e-008	1	K.GVGIISEGNETVEDIAAR.L

Proteins matching the same set of peptides:
IPI00420569 **Mass:** 113457 **Score:** 171 **Queries matched:** 5
Tax_Id=10090 Gene_Symbol=Atp1a2 Sodium/potassium-transporting ATPase subunit alpha-2
IPI00752412 **Mass:** 117548 **Score:** 171 **Queries matched:** 5
Tax_Id=10090 Gene_Symbol=Atp1a3 ATPase, Na+/K+ transporting, alpha 3 polypeptide, isoform CRA_c
IPI00762871 **Mass:** 104717 **Score:** 171 **Queries matched:** 5
Tax_Id=10090 Gene_Symbol=Atp1a2 Uncharacterized protein

---

12. IPI00138892 **Mass:** 15004 **Score:** 170 **Queries matched:** 3 **emPAI:** 0.23
Tax_Id=10090 Gene_Symbol=Uba52 Ubiquitin-60S ribosomal protein L40
☐ Check to include this hit in error tolerant search or archive report

	Query	Observed	Mr(expt)	Mr(calc)	ppm	Miss	Score	Expect	Rank	Peptide
☑	547	894.4697	1786.9248	1786.9200	2.71	0	81	4.8e-007	1	K.TITLEVEPSDTIENVK.A
☑	548	894.4703	1786.9260	1786.9200	3.38	0	(78)	9.9e-007	1	K.TITLEVEPSDTIENVK.A
☑	549	894.4712	1786.9278	1786.9200	4.38	0	(73)	2.8e-006	1	K.TITLEVEPSDTIENVK.A

Proteins matching the same set of peptides:
IPI00470152 **Mass:** 18282 **Score:** 170 **Queries matched:** 3
Tax_Id=10090 Gene_Symbol=Rps27a Ubiquitin-40S ribosomal protein S27a
IPI00895319 **Mass:** 22277 **Score:** 170 **Queries matched:** 3
Tax_Id=10090 Gene_Symbol=2810422J05Rik 22 kDa protein
IPI00895479 **Mass:** 27181 **Score:** 170 **Queries matched:** 3
Tax_Id=10090 Gene_Symbol=2810422J05Rik 27 kDa protein
IPI00918763 **Mass:** 11021 **Score:** 170 **Queries matched:** 3
Tax_Id=10090 Gene_Symbol=Uba52 Ubiquitin subunit 1
IPI00750889 **Mass:** 22822 **Score:** 170 **Queries matched:** 3
Tax_Id=10090 Gene_Symbol=Ubc Ubc protein
IPI00923013 **Mass:** 26603 **Score:** 170 **Queries matched:** 3
Tax_Id=10090 Gene_Symbol=Ubb Ubiquitin B
IPI00923037 **Mass:** 22578 **Score:** 170 **Queries matched:** 3
Tax_Id=10090 Gene_Symbol=Ubc Putative uncharacterized protein
IPI00139518 **Mass:** 34348 **Score:** 170 **Queries matched:** 3
Tax_Id=10090 Gene_Symbol=Ubb Polyubiquitin-B
IPI00755916 **Mass:** 82614 **Score:** 170 **Queries matched:** 3
Tax_Id=10090 Gene_Symbol=Ubc ubiquitin
IPI00969323 **Mass:** 82603 **Score:** 170 **Queries matched:** 3
Tax_Id=10090 Gene_Symbol=Ubc Polyubiquitin-C

---

13. IPI00120793 **Mass:** 28131 **Score:** 168 **Queries matched:** 14 **emPAI:** 0.40
Tax_Id=10090 Gene_Symbol=Prnp Major prion protein
☐ Check to include this hit in error tolerant search or archive report

	Query	Observed	Mr(expt)	Mr(calc)	ppm	Miss	Score	Expect	Rank	Peptide
☑	242	544.7363	1087.4580	1087.4570	0.94	0	43	0.0012	1	K.ESQAYYDGR.R
☑	243	544.7366	1087.4586	1087.4570	1.49	0	(37)	0.0045	1	K.ESQAYYDGR.R
☑	244	544.7393	1087.4640	1087.4570	6.45	0	(41)	0.0019	1	K.ESQAYYDGR.R

Query	Observed	Mr(expt)	Mr(calc)	ppm	Miss	Score	Expect	Rank	Peptide
245	544.7401	1087.4656	1087.4570	7.92	0	(38)	0.0044	1	K.ESQAYYDGR.R
246	545.2651	1088.5156	1088.4999	14.4	0	4	22	4	R.YPGQGSPGGNR.Y
268	570.2647	1138.5148	1138.5142	0.54	0	(38)	0.008	1	K.GENFTETDVK.M
269	570.2656	1138.5166	1138.5142	2.12	0	50	0.00044	1	K.GENFTETDVK.M
270	570.2673	1138.5200	1138.5142	5.10	0	(46)	0.0011	1	K.GENFTETDVK.M
271	570.2728	1138.5310	1138.5142	14.8	0	(44)	0.0022	1	K.GENFTETDVK.M
322	622.7874	1243.5602	1243.5581	1.70	1	16	1.1	1	K.ESQAYYDGRR.S
325	622.7895	1243.5644	1243.5581	5.08	1	(11)	3.5	1	K.ESQAYYDGRR.S
327	622.7902	1243.5658	1243.5581	6.20	1	(4)	19	1	K.ESQAYYDGRR.S
328	622.7919	1243.5692	1243.5581	8.94	1	(2)	34	2	K.ESQAYYDGRR.S
437	756.8663	1511.7180	1511.7112	4.50	0	65	2.1e-005	1	R.VVEQMCVTQYQK.E

14. IPI00122928 **Mass:** 50514 **Score:** 143 **Queries matched:** 7 **emPAI:** 0.21
Tax_Id=10090 Gene_Symbol=Tubb6 Tubulin beta-6 chain
Check to include this hit in error tolerant search or archive report

Query	Observed	Mr(expt)	Mr(calc)	ppm	Miss	Score	Expect	Rank	Peptide
273	572.3212	1142.6278	1142.6270	0.72	0	42	0.0049	1	K.LAVNMVPFPR.L
397	676.3429	1350.6712	1350.6676	2.72	0	(67)	1.4e-005	1	R.IMNTFSVMPSPK.V
398	676.3460	1350.6774	1350.6676	7.31	0	79	1e-006	1	R.IMNTFSVMPSPK.V
532	846.4391	1690.8636	1690.8600	2.16	0	(20)	0.66	1	R.ALTVPELTQQMFDAK.N
533	846.4404	1690.8662	1690.8600	3.69	0	40	0.0061	1	R.ALTVPELTQQMFDAK.N
534	848.9236	1695.8326	1695.8257	4.12	0	15	2.1	1	K.NSSYFVEWIPNNVK.V
537	854.4365	1706.8584	1706.8549	2.07	0	(30)	0.064	2	R.ALTVPELTQQMFDAK.N + Oxidation (M)

15. IPI00225062 **Mass:** 285676 **Score:** 134 **Queries matched:** 4 **emPAI:** 0.03
Tax_Id=10090 Gene_Symbol=Srrm2 Isoform 3 of Serine/arginine repetitive matrix protein 2
Check to include this hit in error tolerant search or archive report

Query	Observed	Mr(expt)	Mr(calc)	ppm	Miss	Score	Expect	Rank	Peptide
404	691.3704	1380.7262	1380.7361	-7.15	0	30	0.069	1	R.TAVAPSAVNLADPR.T
405	693.3643	1384.7140	1384.7133	0.57	0	64	3.2e-005	1	R.MAPALSGANLTSPR.V
411	701.3675	1400.7204	1400.7259	-3.92	0	62	5.6e-005	1	R.TSAIPASVNLADSR.T
443	759.4198	1516.8250	1516.8362	-7.34	0	67	1.1e-005	1	R.TPQAPTPANLVVGPR.S

Proteins matching the same set of peptides:
IPI00785240 **Mass:** 295356 **Score:** 134 **Queries matched:** 4
Tax_Id=10090 Gene_Symbol=Srrm2 Isoform 1 of Serine/arginine repetitive matrix protein 2
IPI00785384 **Mass:** 284043 **Score:** 134 **Queries matched:** 4
Tax_Id=10090 Gene_Symbol=Srrm2 Isoform 2 of Serine/arginine repetitive matrix protein 2
IPI00874854 **Mass:** 295521 **Score:** 134 **Queries matched:** 4
Tax_Id=10090 Gene_Symbol=Srrm2 Srrm2 protein

16. IPI00221528 **Mass:** 42319 **Score:** 133 **Queries matched:** 6 **emPAI:** 0.16
Tax_Id=10090 Gene_Symbol=Actbl2 Beta-actin-like protein 2
Check to include this hit in error tolerant search or archive report

Query	Observed	Mr(expt)	Mr(calc)	ppm	Miss	Score	Expect	Rank	Peptide
550	895.9509	1789.8872	1789.8846	1.46	0	85	2e-007	1	R.SYELPDGQVITIGNER.F
551	895.9536	1789.8926	1789.8846	4.48	0	(73)	3.1e-006	1	R.SYELPDGQVITIGNER.F
566	652.0146	1953.0220	1953.0571	-17.99	0	(33)	0.023	2	R.VAPDEHPILLTEAPLNPK.I
567	652.0152	1953.0238	1953.0571	-17.06	0	39	0.0057	2	R.VAPDEHPILLTEAPLNPK.I
568	652.0201	1953.0385	1953.0571	-9.54	0	(27)	0.098	2	R.VAPDEHPILLTEAPLNPK.I
569	652.0240	1953.0502	1953.0571	-3.55	0	(36)	0.011	2	R.VAPDEHPILLTEAPLNPK.I

17. IPI00404590 **Mass:** 19412 **Score:** 102 **Queries matched:** 10 **emPAI:** 0.38
Tax_Id=10090 Gene_Symbol=H1f0 Putative uncharacterized protein
Check to include this hit in error tolerant search or archive report

Query	Observed	Mr(expt)	Mr(calc)	ppm	Miss	Score	Expect	Rank	Peptide
223	530.7672	1059.5198	1059.5196	0.20	0	(7)	20	1	K.VGENADSQIK.L
225	530.7692	1059.5238	1059.5196	3.97	0	(48)	0.0015	1	K.VGENADSQIK.L
226	530.7695	1059.5244	1059.5196	4.54	0	(33)	0.049	1	K.VGENADSQIK.L
227	530.7698	1059.5250	1059.5196	5.11	0	(26)	0.27	1	K.VGENADSQIK.L
228	530.7698	1059.5250	1059.5196	5.11	0	(15)	3.6	1	K.VGENADSQIK.L
229	530.7701	1059.5256	1059.5196	5.67	0	62	6.9e-005	1	K.VGENADSQIK.L
231	530.7714	1059.5282	1059.5196	8.13	0	(22)	0.72	1	K.VGENADSQIK.L
232	530.7786	1059.5426	1059.5196	21.7	0	(19)	1.4	1	K.VGENADSQIK.L
354	651.3392	1300.6638	1300.6623	1.22	0	46	0.002	1	M.TENSTSAPAAKPK.R
418	719.8656	1437.7166	1437.7173	-0.48	0	30	0.079	1	K.YSDMIVAAIQAEK.N

Proteins matching the same set of peptides:
IPI00467914 **Mass:** 20848 **Score:** 102 **Queries matched:** 10
Tax_Id=10090 Gene_Symbol=H1f0 Histone H1.0

18. IPI00323357 **Mass:** 71055 **Score:** 79 **Queries matched:** 3 **emPAI:** 0.09
Tax_Id=10090 Gene_Symbol=Hspa8 Heat shock cognate 71 kDa protein
Check to include this hit in error tolerant search or archive report

Query	Observed	Mr(expt)	Mr(calc)	ppm	Miss	Score	Expect	Rank	Peptide
356	652.3232	1302.6318	1302.5914	31.1	0	48	0.0013	1	K.NSLESYAFNMK.A
490	808.9067	1615.7988	1615.7804	11.4	0	14	2.7	1	K.SFYPEEVSSMVLTK.M

☑	529	825.4030	1648.7914	1648.7879	2.15	0	64	2.6e-005	1	K.NQVAMNPTNTVFDAK.R

Proteins matching the same set of peptides:
IPI00886297 **Mass:** 68964 **Score:** 79 **Queries matched:** 3
Tax_Id=10090 Gene_Symbol=Hspa8 Hspa8 protein

---

19. IPI00469012 **Mass:** 87791 **Score:** 76 **Queries matched:** 3 **emPAI:** 0.08
Tax_Id=10090 Gene_Symbol=Tbc1d10b TBC1 domain family member 10B
☐ Check to include this hit in error tolerant search or archive report

	Query	Observed	Mr(expt)	Mr(calc)	ppm	Miss	Score	Expect	Rank	Peptide
☑	267	568.8079	1135.6012	1135.5986	2.34	0	50	0.00078	1	R.ASPVPGPGTPTR.T
☑	561	925.0280	1848.0414	1848.0356	3.14	0	(38)	0.0037	1	R.QQPPLGPSSSLLSLPSLK.S
☑	562	925.0283	1848.0420	1848.0356	3.46	0	47	0.00049	1	R.QQPPLGPSSSLLSLPSLK.S

---

20. IPI00223714 **Mass:** 21964 **Score:** 68 **Queries matched:** 2 **emPAI:** 0.15
Tax_Id=10090 Gene_Symbol=Hist1h1e Histone H1.4
☐ Check to include this hit in error tolerant search or archive report

	Query	Observed	Mr(expt)	Mr(calc)	ppm	Miss	Score	Expect	Rank	Peptide
☑	314	614.8428	1227.6710	1227.6711	-0.01	0	(46)	0.0018	1	K.TSGPPVSELITK.A
☑	315	614.8445	1227.6744	1227.6711	2.75	0	60	6.8e-005	1	K.TSGPPVSELITK.A

---

21. IPI00656325 **Mass:** 83131 **Score:** 67 **Queries matched:** 2 **emPAI:** 0.08
Tax_Id=10090 Gene_Symbol=Nsf Vesicle-fusing ATPase
☐ Check to include this hit in error tolerant search or archive report

	Query	Observed	Mr(expt)	Mr(calc)	ppm	Miss	Score	Expect	Rank	Peptide
☑	353	646.3333	1290.6520	1290.6568	-3.68	0	40	0.0086	1	K.NFSGAELEGLVR.A
☑	425	729.3958	1456.7770	1456.7773	-0.19	0	57	0.00012	1	R.VLDDGELLVQQTK.N

Proteins matching the same set of peptides:
IPI00876255 **Mass:** 83174 **Score:** 67 **Queries matched:** 2
Tax_Id=10090 Gene_Symbol=Nsf 83 kDa protein

---

22. IPI00377681 **Mass:** 42045 **Score:** 62 **Queries matched:** 1 **emPAI:** 0.08
Tax_Id=10090 Gene_Symbol=N28178 Isoform 2 of Protein KIAA1045
☐ Check to include this hit in error tolerant search or archive report

	Query	Observed	Mr(expt)	Mr(calc)	ppm	Miss	Score	Expect	Rank	Peptide
☑	399	680.8234	1359.6322	1359.6266	4.15	0	62	3.5e-005	1	R.ALTDEQEEQAAR.Q

Proteins matching the same set of peptides:
IPI00647986 **Mass:** 45936 **Score:** 62 **Queries matched:** 1
Tax_Id=10090 Gene_Symbol=N28178 Isoform 1 of Protein KIAA1045
IPI00885599 **Mass:** 29710 **Score:** 62 **Queries matched:** 1
Tax_Id=10090 Gene_Symbol=N28178 Novel protein

---

23. IPI00122069 **Mass:** 79562 **Score:** 61 **Queries matched:** 1 **emPAI:** 0.04
Tax_Id=10090 Gene_Symbol=Prkcc Protein kinase C gamma type
☐ Check to include this hit in error tolerant search or archive report

	Query	Observed	Mr(expt)	Mr(calc)	ppm	Miss	Score	Expect	Rank	Peptide
☑	308	599.8120	1197.6094	1197.5990	8.75	0	61	5.4e-005	1	R.LGSGPDGEPTIR.A

---

24. IPI00136703 **Mass:** 42971 **Score:** 56 **Queries matched:** 3 **emPAI:** 0.08
Tax_Id=10090 Gene_Symbol=Ckb Creatine kinase B-type
☐ Check to include this hit in error tolerant search or archive report

	Query	Observed	Mr(expt)	Mr(calc)	ppm	Miss	Score	Expect	Rank	Peptide
	191	516.2849	1030.5552	1030.5481	6.96	0	8	17	2	K.LLIEMEQR.L
☑	363	652.3676	1302.7206	1302.7183	1.78	0	56	0.00016	1	K.VLTPELYAELR.A
☑	530	828.9172	1655.8198	1655.8188	0.60	0	30	0.059	1	R.LEQGQAIDDLMPAQK.-

---

25. IPI00347110 **Mass:** 59502 **Score:** 50 **Queries matched:** 1 **emPAI:** 0.06
Tax_Id=10090 Gene_Symbol=Krt73 Keratin, type II cytoskeletal 73
☐ Check to include this hit in error tolerant search or archive report

	Query	Observed	Mr(expt)	Mr(calc)	ppm	Miss	Score	Expect	Rank	Peptide
☑	434	738.3943	1474.7740	1474.7780	-2.67	0	50	0.00071	1	R.FLEQQNQVLQTK.W

Proteins matching the same set of peptides:
IPI00420970 **Mass:** 55340 **Score:** 50 **Queries matched:** 1
Tax_Id=10090 Gene_Symbol=Krt74 Keratin, type II cytoskeletal 74
IPI00462140 **Mass:** 61379 **Score:** 50 **Queries matched:** 1
Tax_Id=10090 Gene_Symbol=Krt77 Keratin, type II cytoskeletal 1b
IPI00468956 **Mass:** 57860 **Score:** 50 **Queries matched:** 1
Tax_Id=10090 Gene_Symbol=Krt71 Keratin, type II cytoskeletal 71
IPI00625729 **Mass:** 66079 **Score:** 50 **Queries matched:** 1
Tax_Id=10090 Gene_Symbol=Krt1 Keratin, type II cytoskeletal 1

26. IPI00403219 **Score:** 46 **Queries matched:** 1
Tax_Id=10090 Gene_Symbol=Gm14496 Novel vomeronasal 2, receptor family protein
Check to include this hit in error tolerant search or archive report

Query	Observed	Mr(expt)	Mr(calc)	ppm	Miss	Score	Expect	Rank	Peptide
155	487.7528	973.4910	973.4617	30.1	0	46	0.0023	1	R.WEDLGAQR.I

27. IPI00119853 **Score:** 44 **Queries matched:** 5
Tax_Id=10090 Gene_Symbol=Gnas Isoform Gnas-1 of Guanine nucleotide-binding protein G(s) subunit alpha isoforms short
Check to include this hit in error tolerant search or archive report

Query	Observed	Mr(expt)	Mr(calc)	ppm	Miss	Score	Expect	Rank	Peptide
203	529.3122	1056.6098	1056.6179	-7.61	0	(18)	0.98	1	R.LLLLGAGESGK.S
204	529.3124	1056.6102	1056.6179	-7.23	0	44	0.0026	1	R.LLLLGAGESGK.S
238	538.2985	1074.5824	1074.6073	-23.14	0	(9)	11	8	R.LQEALNLFK.S
239	538.2990	1074.5834	1074.6073	-22.21	0	21	0.64	2	R.LQEALNLFK.S
240	538.3007	1074.5868	1074.6073	-19.05	0	(11)	6.8	2	R.LQEALNLFK.S

Proteins matching the same set of peptides:
IPI00463891 **Score:** 44 **Queries matched:** 5
IPI00750570 **Score:** 44 **Queries matched:** 5
IPI00416906 **Score:** 44 **Queries matched:** 5
IPI00469793 **Score:** 44 **Queries matched:** 5

28. IPI00347770 **Score:** 44 **Queries matched:** 3
Tax_Id=10090 Gene_Symbol=Gnat3 Guanine nucleotide-binding protein G(t) subunit alpha-3
Check to include this hit in error tolerant search or archive report

Query	Observed	Mr(expt)	Mr(calc)	ppm	Miss	Score	Expect	Rank	Peptide
203	529.3122	1056.6098	1056.6179	-7.61	0	(18)	0.98	1	K.LLLLGAGESGK.S
204	529.3124	1056.6102	1056.6179	-7.23	0	44	0.0026	1	K.LLLLGAGESGK.S
**221**	**530.3175**	**1058.6204**	**1058.5873**	**31.4**	**1**	**10**	**4.6**	**4**	**K.IIHKNGYSK.Q**

29. IPI00118569 **Mass:** 44369 **Score:** 44 **Queries matched:** 4 **emPAI:** 0.07
Tax_Id=10090 Gene_Symbol=Gna13 Guanine nucleotide-binding protein subunit alpha-13
Check to include this hit in error tolerant search or archive report

Query	Observed	Mr(expt)	Mr(calc)	ppm	Miss	Score	Expect	Rank	Peptide
203	529.3122	1056.6098	1056.6179	-7.61	0	(18)	0.98	1	K.ILLLGAGESGK.S
204	529.3124	1056.6102	1056.6179	-7.23	0	44	0.0026	1	K.ILLLGAGESGK.S
335	631.3046	1260.5946	1260.5955	-0.64	0	3	34	7	R.APMAAQGMVETR.V
340	631.3057	1260.5968	1260.5955	1.10	0	(3)	35	6	R.APMAAQGMVETR.V

Proteins matching the same set of peptides:
IPI00649388 **Mass:** 20095 **Score:** 44 **Queries matched:** 4
Tax_Id=10090 Gene_Symbol=Gna13 Putative uncharacterized protein

30. IPI00123176 **Mass:** 36804 **Score:** 43 **Queries matched:** 2 **emPAI:** 0.09
Tax_Id=10090 Gene_Symbol=Gm6316 Glyceraldehyde-3-phosphate dehydrogenase
Check to include this hit in error tolerant search or archive report

Query	Observed	Mr(expt)	Mr(calc)	ppm	Miss	Score	Expect	Rank	Peptide
401	**685.3772**	**1368.7398**	**1368.7361**	**2.74**	**0**	**(26)**	**0.13**	**1**	**R.GAAQNIIPASTGAAK.A**
402	**685.3792**	**1368.7438**	**1368.7361**	**5.66**	**0**	**43**	**0.0027**	**1**	**R.GAAQNIIPASTGAAK.A**

Proteins matching the same set of peptides:
IPI00135284 **Mass:** 36092 **Score:** 43 **Queries matched:** 2
Tax_Id=10090 Gene_Symbol=Gm16374 Glyceraldehyde-3-phosphate dehydrogenase
IPI00229201 **Mass:** 16676 **Score:** 43 **Queries matched:** 2
Tax_Id=10090 Gene_Symbol=3000002C10Rik Putative uncharacterized protein
IPI00271869 **Mass:** 36293 **Score:** 43 **Queries matched:** 2
Tax_Id=10090 Gene_Symbol=- 36 kDa protein
IPI00273646 **Mass:** 36072 **Score:** 43 **Queries matched:** 2
Tax_Id=10090 Gene_Symbol=Gapdh;LOC100042025 Glyceraldehyde-3-phosphate dehydrogenase
IPI00622795 **Mass:** 36074 **Score:** 43 **Queries matched:** 2
Tax_Id=10090 Gene_Symbol=- 36 kDa protein
IPI00625893 **Mass:** 36205 **Score:** 43 **Queries matched:** 2
Tax_Id=10090 Gene_Symbol=Gm6981 Glyceraldehyde-3-phosphate dehydrogenase
IPI00752289 **Mass:** 36308 **Score:** 43 **Queries matched:** 2
Tax_Id=10090 Gene_Symbol=Gm10290 Glyceraldehyde-3-phosphate dehydrogenase
IPI00848801 **Mass:** 36100 **Score:** 43 **Queries matched:** 2
Tax_Id=10090 Gene_Symbol=- 36 kDa protein
IPI00849045 **Mass:** 36072 **Score:** 43 **Queries matched:** 2
Tax_Id=10090 Gene_Symbol=- 36 kDa protein
IPI00850337 **Mass:** 44633 **Score:** 43 **Queries matched:** 2
Tax_Id=10090 Gene_Symbol=LOC100048117 similar to Glyceraldehyde-3-phosphate dehydrogenase (GAPDH) isoform 1
IPI00850377 **Mass:** 35975 **Score:** 43 **Queries matched:** 2
Tax_Id=10090 Gene_Symbol=- 36 kDa protein
IPI00850779 **Mass:** 36076 **Score:** 43 **Queries matched:** 2
Tax_Id=10090 Gene_Symbol=- 36 kDa protein
IPI00874682 **Mass:** 36038 **Score:** 43 **Queries matched:** 2
Tax_Id=10090 Gene_Symbol=- 36 kDa protein
IPI00874964 **Mass:** 36512 **Score:** 43 **Queries matched:** 2

Tax_Id=10090 Gene_Symbol=- 36 kDa protein

31. IPI00348094 **Mass:** 51093 **Score:** 42 **Queries matched:** 3 **emPAI:** 0.06
Tax_Id=10090 Gene_Symbol=Tubb1 Tubulin, beta 1
Check to include this hit in error tolerant search or archive report

Query	Observed	Mr(expt)	Mr(calc)	ppm	Miss	Score	Expect	Rank	Peptide
273	572.3212	1142.6278	1142.6270	0.72	0	42	0.0049	1	K.LAVNMVPFPR.L
364	660.3562	1318.6978	1318.6955	1.78	0	10	7.7	2	R.ILNSFSVMPSPK.V
365	660.3578	1318.7010	1318.6955	4.21	0	(10)	7.9	2	R.ILNSFSVMPSPK.V

32. IPI00116074 **Mass:** 86151 **Score:** 40 **Queries matched:** 1 **emPAI:** 0.04
Tax_Id=10090 Gene_Symbol=Aco2 Aconitate hydratase, mitochondrial
Check to include this hit in error tolerant search or archive report

Query	Observed	Mr(expt)	Mr(calc)	ppm	Miss	Score	Expect	Rank	Peptide
428	**732.3771**	**1462.7396**	**1462.7416**	**-1.34**	**0**	**40**	**0.009**	**1**	**K.SQFTITPGSEQIR.A**

33. IPI00119063 **Mass:** 523342 **Score:** 37 **Queries matched:** 3 **emPAI:** 0.01
Tax_Id=10090 Gene_Symbol=Lrp1 Prolow-density lipoprotein receptor-related protein 1
Check to include this hit in error tolerant search or archive report

Query	Observed	Mr(expt)	Mr(calc)	ppm	Miss	Score	Expect	Rank	Peptide
152	484.7774	967.5402	967.4975	44.2	0	(5)	15	5	R.GIPLDPNDK.S
153	484.7776	967.5406	967.4975	44.6	0	9	5.4	3	R.GIPLDPNDK.S
333	**626.3710**	**1250.7274**	**1250.7347**	**-5.78**	**0**	**37**	**0.0059**	**1**	**R.GVAGAPPTVTLLR.S**

Peptide matches not assigned to protein hits: (no details means no match)

Query	Observed	Mr(expt)	Mr(calc)	ppm	Miss	Score	Expect	Rank	Peptide
407	696.3701	1390.7256	1390.7245	0.82	0	30	0.064	1	LEGVVFPYQPSR
196	523.2819	1044.5492	1044.5564	-6.80	0	29	0.12	1	INDITLNSR
154	487.3037	972.5928	972.5505	43.6	1	29	0.045	1	VRDFLAPR
195	520.2805	1038.5464	1038.5458	0.65	0	28	0.1	1	AQASAPAQAPK
309	599.8347	1197.6548	1197.6605	-4.71	0	27	0.12	1	ASGPPVSELITK
368	669.3201	1336.6256	1336.6187	5.22	0	27	0.13	1	YALYDATYETK
558	912.9982	1823.9818	1823.9782	2.02	0	22	0.24	1	VGINYQPPTVVPGGDLAK
25	531.3553	530.3480	530.3428	9.90	0	20	0.74	1	ILASK
419	720.3734	1438.7322	1438.7344	-1.48	0	19	0.76	1	YITPDQLADLYK
63	408.2385	814.4624	814.4661	-4.48	1	19	1.2	1	GTVAPSKR
606	737.7026	2210.0860	2210.0307	25.0	1	19	0.66	1	GLMVLQSIASRGCDTQDSEK + Oxidation (M)
78	421.7573	841.5000	841.4810	22.6	0	19	0.75	1	LGTWLPR
32	590.3131	589.3058	589.3006	8.87	0	18	2.1	1	ANMVR
130	464.7809	927.5472	927.5502	-3.15	1	18	0.88	1	GITTVPGKR
143	470.7710	939.5274	939.5614	-36.11	1	18	0.7	1	NRLPIATR
313	614.3115	1226.6084	1226.6448	-29.61	1	18	0.91	1	IYYYHVITR
194	520.2767	1038.5388	1038.5458	-6.67	0	18	1.1	1	AQASAPAQAPK
496	812.9454	1623.8762	1623.8832	-4.28	0	17	0.91	1	TGAIVDVPVGEELLGR
190	514.7952	1027.5758	1027.5774	-1.55	1	17	1.4	1	VAGSGKSPAVR
151	481.7329	961.4512	961.4586	-7.62	1	16	1.9	1	RQAMPGMR + Oxidation (M)
52	385.7109	769.4072	769.4446	-48.56	1	15	1.5	1	LEPKQR
27	531.3663	530.3590	530.3428	30.6	0	15	0.67	1	ILASK
26	531.3587	530.3514	530.3428	16.3	0	15	2.6	1	ILASK
80	428.7653	855.5160	855.5112	5.61	1	15	1.7	1	ALRMLPR
28	531.3701	530.3628	530.3428	37.8	0	14	0.78	1	ILASK
84	435.7735	869.5324	869.4971	40.7	0	14	1.5	1	AVVESLPR
221	530.3175	1058.6204	1058.6488	-26.77	1	14	1.9	1	KFLILNPSK
72	415.1994	828.3842	828.4202	-43.39	1	14	1.7	1	ARGESPGR
246	545.2651	1088.5156	1088.5098	5.38	0	13	3.4	1	EESADVNLGR
205	530.2988	1058.5830	1058.5906	-7.15	1	13	4.6	1	MPTLKANIR + Oxidation (M)
104	450.7215	899.4284	899.4097	20.8	0	13	2.7	1	STEPSHSR
253	556.8272	1111.6398	1111.5985	37.2	1	13	2.2	1	QQPKEALGNK
76	415.2051	828.3956	828.3838	14.3	1	12	2.6	1	NRDPDGR
612	756.0484	2265.1234	2265.0770	20.5	0	12	3.1	1	MTPAGTSLPTSWLICTSPSSR + Oxidation (M)
640	779.7223	2336.1451	2336.1617	-7.10	1	12	3	1	HMAAPLIGQLTRHEIEMTEK + 2 Oxidation (M)
255	556.8319	1111.6492	1111.5986	45.6	0	12	2.3	1	IQENVPVGTR
73	415.2014	828.3882	828.4276	-47.50	0	12	2.6	1	IHGSMLR + Oxidation (M)
348	643.3405	1284.6664	1284.6561	8.02	0	12	4.8	1	LLVVDPETDER
115	450.7243	899.4340	899.4726	-42.84	1	12	3.5	1	DGFHRLR
150	481.7226	961.4306	961.4730	-44.01	1	11	3.5	1	QDPGARYR
189	514.7838	1027.5530	1027.5927	-38.57	1	11	5.6	1	RLWPTLSR
92	450.7179	899.4212	899.4461	-27.62	0	11	3.3	1	GLTADPGNR
86	435.7745	869.5344	869.5123	25.5	0	11	3.1	1	VSLWLPR
173	506.2323	1010.4500	1010.4703	-20.02	0	11	3.2	1	GDTTTTMLR + Oxidation (M)
90	450.7172	899.4198	899.4647	-49.87	0	11	3.5	1	SLQHMLR + Oxidation (M)
177	506.2389	1010.4632	1010.4855	-22.01	0	11	4	1	NLMADYLR + Oxidation (M)
141	470.7696	939.5246	939.5614	-39.09	1	11	3.9	1	NRLPIATR
66	412.7505	823.4864	823.4817	5.80	0	11	2.2	1	RPWLPR
594	737.3668	2209.0786	2209.1491	-31.92	1	11	4.3	1	LGEHNIKVLEGNEQFVNAAK
230	530.7705	1059.5264	1059.5495	-21.74	0	11	8.8	1	QALSNMLQR
61	407.2281	812.4416	812.4756	-41.75	1	10	5.1	1	AGPKALEK

79	428.7645	855.5144	855.4814	38.6	0	10	4.5	1	LGAETLPR
140	470.7692	939.5238	939.5137	10.8	0	10	4.6	1	NPEALAAVR
101	450.7208	899.4270	899.4322	-5.68	1	10	4.3	1	NRSSGSHR
100	450.7206	899.4266	899.4647	-42.31	0	10	4.5	1	SLQHMLR + Oxidation (M)
385	670.8940	1339.7734	1339.7506	17.0	1	10	3.5	1	AERPILGAMARR
142	470.7698	939.5250	939.5515	-28.14	1	10	4.5	1	LRPGWRR
295	595.3065	1188.5984	1188.6033	-4.10	1	10	7.9	1	QMQASRLSPR + Oxidation (M)
70	414.2204	826.4262	826.4661	-48.20	0	10	5.4	1	VAAPNLSR
107	450.7224	899.4302	899.4647	-38.31	0	10	4.9	1	SLQHMLR + Oxidation (M)
75	415.2028	828.3910	828.4276	-44.12	0	10	4	1	IHGSMLR + Oxidation (M)
191	516.2849	1030.5552	1030.5923	-36.00	0	10	11	1	LLIFQNQR
91	450.7173	899.4200	899.4322	-13.47	1	10	4.3	1	NRSSGSHR
176	506.2365	1010.4584	1010.4314	26.8	0	10	4.7	1	ACMDYPVR
171	506.2305	1010.4464	1010.4781	-31.33	0	10	4.2	1	DLLHNEDR
56	810.4305	809.4232	809.4139	11.5	1	10	6.3	1	KMQMLK + 2 Oxidation (M)
19	503.2858	502.2785	502.2863	-15.54	0	9	24	1	AGSIR
85	435.7742	869.5338	869.5123	24.8	0	9	4.6	1	VSLWLPR
102	450.7211	899.4276	899.4322	-5.02	1	9	5.8	1	NRSSGSHR
89	450.7172	899.4198	899.4209	-1.22	0	9	5	1	TQQGSSHR
693	961.4171	2881.2295	2881.2557	-9.10	1	9	2.2	1	AVSAFQSMSGGSMFQCSWQHRFMAK + Oxidation (M)
98	450.7204	899.4262	899.4324	-6.79	0	9	5.4	1	FMFNGLR + Oxidation (M)
103	450.7213	899.4280	899.4322	-4.57	1	9	6	1	NRSSGSHR
283	578.8117	1155.6088	1155.6108	-1.72	1	9	9.3	1	QQQQEALRR
164	499.2584	996.5022	996.5062	-3.99	0	9	9.1	1	KPMSSYLR + Oxidation (M)
35	616.3713	615.3640	615.3592	7.90	0	9	25	1	ALSTPK
67	412.7511	823.4876	823.4817	7.25	0	9	3.7	1	RPWLPR
603	737.7007	2210.0803	2210.1843	-47.07	0	9	6.9	1	CGATSLMASTVLLLLFSWK
477	795.3494	1588.6842	1588.6787	3.47	0	9	3.9	1	NNQDNTTNTDMPPK
36	628.3656	627.3583	627.3704	-19.24	1	9	7.5	1	EKVPR
621	762.0510	2283.1312	2283.1706	-17.28	1	8	6.8	1	ATSPSNNVDEVQIPEISLSKR
349	643.3462	1284.6778	1284.6357	32.8	1	8	10	1	GRAGVCDPALNR
641	783.7235	2348.1487	2348.1471	0.68	1	8	7.1	1	SFLIWVNEEDHTRVISMEK + Oxidation (M)
112	450.7238	899.4330	899.4647	-35.20	0	8	7.5	1	SLQHMLR + Oxidation (M)
346	636.8115	1271.6084	1271.6357	-21.44	0	8	8.3	1	LLDAESEDRPK
108	450.7229	899.4312	899.4647	-37.20	0	8	7.8	1	SLQHMLR + Oxidation (M)
111	450.7234	899.4322	899.4647	-36.09	0	8	7.8	1	SLQHMLR + Oxidation (M)
403	458.9094	1373.7064	1373.7159	-6.94	0	8	13	1	KPALTMEVVCAR
97	450.7202	899.4258	899.4097	17.9	0	8	6.9	1	HVGDDTTR
220	530.3162	1058.6178	1058.5972	19.5	0	8	8.1	1	VTASLLGSPSK
88	443.2438	884.4730	884.5080	-39.48	1	8	9.3	1	SPPKTTVR
148	477.2345	952.4544	952.4284	27.4	1	8	11	1	KSSEEAMR + Oxidation (M)
658	888.7558	2663.2456	2663.2034	15.8	1	8	6.9	1	SGYFELPTSQDCARSNHQDPLGGK
237	538.2737	1074.5328	1074.5054	25.6	1	8	16	1	NSKEPGSNSR
74	415.2025	828.3904	828.4051	-17.71	0	8	6.9	1	YATIMSK + Oxidation (M)
625	762.0553	2283.1441	2283.1894	-19.87	0	7	8.3	1	EEVIDFSKPFMSLGISIMIK
691	933.7790	2798.3152	2798.3262	-3.94	1	7	6.5	1	NLGNPPNAVLCMSQGRDPETMVQVR + Oxidation (M)
99	450.7206	899.4266	899.4647	-42.31	0	7	9	1	HITNLMR + Oxidation (M)
121	452.7405	903.4664	903.4774	-12.11	0	7	19	1	LGSSQVASR
259	557.7951	1113.5756	1113.5891	-12.05	1	7	16	1	SSPLRGPGTSR
224	530.7690	1059.5234	1059.5210	2.33	0	7	20	1	QEGHLPHSR
247	545.2892	1088.5638	1088.5760	-11.18	1	7	18	1	RANNINMLK + Oxidation (M)
93	450.7189	899.4232	899.4573	-37.87	1	7	8.4	1	QSQEPRR
294	595.3062	1188.5978	1188.6172	-16.31	0	7	16	1	MLISAASPDIR + Oxidation (M)
60	406.2201	810.4256	810.4235	2.60	1	7	12	1	AQYSSKK
392	672.3433	1342.6720	1342.6364	26.5	1	7	15	1	ADAEEEAATRIPA
655	888.7497	2663.2273	2663.1670	22.6	0	7	7.6	1	NLTTIGYNWEDHNIEEQCQSGR
135	470.7658	939.5170	939.5614	-47.17	1	7	11	1	LPRASGALR
257	557.3074	1112.6002	1112.6342	-30.54	0	7	14	1	GLFLSNVVHK
265	565.3054	1128.5962	1128.6139	-15.61	0	7	17	1	TLIEQIEQR
412	701.8926	1401.7706	1401.7715	-0.61	0	7	12	1	ATQPSLTELTTLK
692	961.4110	2881.2112	2881.1807	10.6	1	7	3	1	NFSPDQSPEFAREADEECYSTQMK + Oxidation (M)
522	816.8976	1631.7806	1631.7912	-6.46	1	7	13	1	MVNRENKPPCYPK
266	565.3197	1128.6248	1128.6503	-22.51	0	7	16	1	TKPVLSSLER
620	762.0505	2283.1297	2283.1190	4.67	1	7	10	1	KGTTTNATATSTSTASTAVADAQK
622	762.0521	2283.1345	2283.1756	-17.99	1	7	10	1	GLVKVLFATETFAMGVNMPAR + 2 Oxidation (M)
166	500.2500	998.4854	998.5145	-29.08	1	6	14	1	KAEGAPNQGK
68	412.7528	823.4910	823.4817	11.4	0	6	6.5	1	RPWLPR
331	622.7954	1243.5762	1243.5721	3.37	0	6	13	1	GELYDYISER
685	933.4488	2797.3246	2797.3051	6.96	0	6	8.7	1	MATQNVPPPPYQDSPQMTATAQPPSK + Oxidation (M)
415	702.3759	1402.7372	1402.7061	22.2	0	6	16	1	MAGVGVGPLQGMVR + 2 Oxidation (M)
179	506.2391	1010.4636	1010.4314	31.9	0	6	11	1	ACMDYPVR
110	450.7234	899.4322	899.4097	25.1	0	6	12	1	STEPSHSR
114	450.7242	899.4338	899.4461	-13.61	0	6	12	1	GQSSAPPTR
542	885.9581	1769.9016	1769.8505	28.9	1	6	15	1	EEPKEEESLSMPLPR
217	530.3068	1058.5990	1058.5608	36.2	1	6	17	1	KSTESVPPSK
607	737.7040	2210.0902	2210.0967	-2.97	0	6	13	1	LGEHNINVLEGNEQFIDAAK
157	491.2421	980.4696	980.4233	47.3	0	6	19	1	AVSMDNSNK + Oxidation (M)
71	415.1973	828.3800	828.4202	-48.46	1	6	9.4	1	REQSPGR
144	470.7715	939.5284	939.5025	27.6	0	6	12	1	NPIVPTDGK
105	450.7220	899.4294	899.4726	-47.95	0	6	13	1	HRPSFTR
626	762.0572	2283.1498	2283.2370	-38.22	1	6	12	1	LMVMEIRNAYAVLYDIILK + Oxidation (M)

☑	133	470.2356	938.4566	938.4471	10.2	0	6	17	1	QAYHHQR
☑	369	670.3066	1338.5986	1338.6099	-8.39	0	6	12	1	NEGQPQGLMHGR + Oxidation (M)
☑	222	530.7654	1059.5162	1059.5574	-38.81	1	6	26	1	AGSGLRWTGR
☑	302	595.3110	1188.6074	1188.5921	12.9	1	6	23	1	MADAAASPVGKR + Oxidation (M)
☑	180	506.2392	1010.4638	1010.5033	-39.01	0	6	13	1	SSPVPNPNLS
☑	260	557.8024	1113.5902	1113.5414	43.8	0	6	22	1	DTNLNDLGPR
☑	136	470.7668	939.5190	939.5614	-45.05	1	6	15	1	NRLPIATR
☑	169	501.2503	1000.4860	1000.5302	-44.08	1	6	20	1	RLLVDDDR
☑	145	470.7721	939.5296	939.5515	-23.24	1	5	13	1	LRPGWRR
☑	138	470.7690	939.5234	939.4774	49.0	1	5	15	1	QAPKEPDR
☑	182	506.2429	1010.4712	1010.5033	-31.68	0	5	15	1	SSPVPNPNLS
☑	248	545.2919	1088.5692	1088.5536	14.4	0	5	27	1	MGLEEELLR
☑	424	728.8551	1455.6956	1455.6664	20.1	0	5	17	1	AGAQGDPDVPECLK
☑	279	573.7990	1145.5834	1145.5975	-12.28	1	5	26	1	AGDRVMVLNR + Oxidation (M)
☑	321	622.7869	1243.5592	1243.5946	-28.39	0	5	13	1	SPTGASDHFLGR
☑	219	530.3109	1058.6072	1058.5720	33.3	0	5	17	1	GSTLQDAVLR
☑	442	506.2327	1515.6763	1515.7053	-19.14	0	5	12	1	LDTPATSDPLSEDR
☑	310	600.2780	1198.5414	1198.5942	-44.03	0	5	12	1	GQPDTVQDALR
☑	688	933.7679	2798.2819	2798.2851	-1.15	1	5	9.9	1	APNTMWSILSSLSSEVPSSDDRTMR + 2 Oxidation (M)
☑	316	615.2961	1228.5776	1228.6386	-49.63	1	5	17	1	AIYLSGYRMR
☑	160	496.2260	990.4374	990.4487	-11.39	1	5	14	1	ASMSRHMR + Oxidation (M)
☑	162	496.2284	990.4422	990.4487	-6.54	1	5	16	1	ASMSRHMR + Oxidation (M)
☑	147	470.7730	939.5314	939.5250	6.87	1	5	14	1	IQTKASHR
☑	576	688.3119	2061.9139	2061.9426	-13.92	1	5	11	1	SKGSQGTSPFPMSPPSPDSR + Oxidation (M)
☑	417	711.3622	1420.7098	1420.6947	10.7	0	5	22	1	AGDPGLQGPAGAPGEK
☑	168	500.2616	998.5086	998.4716	37.1	0	5	23	1	HMEARPSR + Oxidation (M)
☑	600	737.6983	2210.0731	2210.0637	4.23	1	5	16	1	LAVTSYNDIQDLMDSGNKAR
☑	131	465.2120	928.4094	928.4147	-5.61	0	5	9	1	FMTEMVR + Oxidation (M)
☑	134	470.7655	939.5164	939.5515	-37.29	1	5	19	1	LRPGWRR
☑	139	470.7692	939.5238	939.5515	-29.41	1	5	17	1	LRPGWRR
☑	345	634.3837	1266.7528	1266.7296	18.4	0	5	6.7	1	KPKPTNQVEVK
☑	161	496.2276	990.4406	990.4479	-7.28	1	5	16	1	DQESRSNR
☑	605	737.7022	2210.0848	2210.1167	-14.45	1	4	18	1	ALFASQEMWLSHRQTHLR
☑	420	728.3528	1454.6910	1454.6572	23.3	1	4	22	1	QEMPQDPRAPDR + Oxidation (M)
☑	234	532.2764	1062.5382	1062.5393	-0.95	1	4	33	1	MRSLAWAGR + Oxidation (M)
☑	53	399.7266	797.4386	797.4548	-20.24	0	4	19	1	LPWLNR
☑	326	622.7897	1243.5648	1243.5615	2.66	0	4	18	1	ETMPVHSQSGR + Oxidation (M)
☑	423	728.8538	1455.6930	1455.6664	18.3	0	4	22	1	AGAQGDPDVPECLK
☑	305	595.3142	1188.6138	1188.6033	8.85	1	4	33	1	SSGVGKNCKPR
☑	94	450.7191	899.4236	899.4647	-45.65	0	4	17	1	SLQHMLR + Oxidation (M)
☑	632	779.7067	2336.0983	2336.0559	18.1	0	4	17	1	DQSQAADMLCVVVNNMEQLR + Oxidation (M)
☑	159	492.2374	982.4602	982.4508	9.58	0	4	22	1	NTEGPYFR
☑	656	888.7521	2663.2345	2663.1412	35.0	1	4	17	1	EFAPSDEELDSYRHGEEWDPQK
☑	521	816.8972	1631.7798	1631.8017	-13.42	1	4	28	1	MLFLSESSNWFKK + Oxidation (M)
☑	163	496.2305	990.4464	990.4671	-20.90	0	4	24	1	YNPASFHR
☑	677	888.7624	2663.2654	2663.2796	-5.33	1	4	19	1	VSSEVQQMWALTEMIRASHTSTR + Oxidation (M)
☑	312	612.3268	1222.6390	1222.5942	36.7	1	4	33	1	SQLQENTFTR
☑	328	622.7919	1243.5692	1243.6091	-32.08	1	4	23	1	ELPRCAATGGGR
☑	535	852.4255	1702.8364	1702.7688	39.7	1	3	29	1	SMSMSVGERVTLSCK + 2 Oxidation (M)
☑	624	762.0549	2283.1429	2283.1190	10.4	1	3	22	1	KGTTTNATATSTSTASTAVADAQK
☑	109	450.7229	899.4312	899.4712	-44.44	0	3	25	1	DLSPIAER
☑	416	711.3582	1420.7018	1420.6947	5.06	0	3	34	1	AGDPGLQGPAGAPGEK
☑	514	816.8953	1631.7760	1631.7638	7.50	1	3	32	1	TENLDDEEKLNNAK
☑	181	506.2395	1010.4644	1010.4703	-5.77	0	3	26	1	GDTTTTMLR + Oxidation (M)
☑	395	672.8402	1343.6658	1343.6681	-1.66	1	3	34	1	AQGDEQEAQLKK
☑	116	450.7244	899.4342	899.4461	-13.18	0	3	26	1	GTPGDQGLR
☑	393	672.3479	1342.6812	1342.6551	19.5	0	3	40	1	MDVQATTPLDPR
☑	47	732.3589	731.3516	731.3562	-6.26	0	3	50	1	DVSAANR
☑	433	736.3515	1470.6884	1470.7566	-46.32	0	3	27	1	LGSTPISGASIDPEK
☑	617	761.7278	2282.1616	2282.0559	46.3	0	3	25	1	VALLEQQMQACTLDFENEK + Oxidation (M)
☑	262	561.2590	1120.5034	1120.4958	6.84	0	3	32	1	MEDEIQDII + Oxidation (M)
☑	391	672.3428	1342.6710	1342.6551	11.9	0	3	41	1	MDVQATTPLDPR
☑	575	514.0181	2052.0433	2052.0211	10.8	0	3	27	1	NEMVAHIHELLVFQGGSR + Oxidation (M)
☑	552	897.9138	1793.8130	1793.8665	-29.80	0	3	27	1	VLCEALQHPGCNIQR
☑	527	816.9020	1631.7894	1631.7905	-0.65	0	2	38	1	YECIFTLYPEGIK
☑	306	595.3143	1188.6140	1188.6285	-12.14	1	2	47	1	KPATDGMAVRK + Oxidation (M)
☑	137	470.7682	939.5218	939.4886	35.4	1	2	30	1	TKHASGSPR
☑	263	561.2593	1120.5040	1120.5295	-22.71	0	2	34	1	MSLGQQQSAR + Oxidation (M)
☑	604	737.7008	2210.0806	2210.1405	-27.12	1	2	30	1	ALSMLRNVTTEQPYFIEAK
☑	422	728.3561	1454.6976	1454.7188	-14.51	1	2	37	1	KRPDEVPDAGPMK + Oxidation (M)
☑	106	450.7221	899.4296	899.4322	-2.79	1	2	29	1	NRSSGSHR
☑	577	688.3141	2061.9205	2061.9426	-10.72	1	2	22	1	SKGSQGTSPFPMSPPSPDSR + Oxidation (M)
☑	235	536.7872	1071.5598	1071.5747	-13.84	1	2	72	1	KLVGATTVHM + Oxidation (M)
☑	615	761.7188	2282.1346	2282.1623	-12.17	0	2	30	1	FPLDYYSIPFPTPTTPLTGR
☑	394	672.8397	1343.6648	1343.6470	13.3	1	2	42	1	KTASYYSPSGQR
☑	167	500.2557	998.4968	998.4743	22.6	0	2	41	1	DSMFGISVK + Oxidation (M)
☑	546	894.4644	1786.9142	1786.8849	16.4	0	2	42	1	TVSWLNEQLELGNER
☑	559	912.9985	1823.9824	1823.9782	2.35	0	2	27	1	VGINYQPPTVVPGGDLAK
☑	519	816.8969	1631.7792	1631.7937	-8.86	1	2	42	1	TEDGEGRAMITPALR + Oxidation (M)
☑	627	762.0612	2283.1618	2283.1958	-14.88	0	2	29	1	AVEILADIIQNSTLGEAEIER
☑	317	615.2972	1228.5798	1228.6387	-47.88	1	2	40	1	AVRFSFTVMR + Oxidation (M)

545	596.3310	1785.9712	1785.9335	21.1	1	1	35	1	KFMTLDPVSQDALPPK
554	904.9239	1807.8332	1807.7836	27.5	0	1	44	1	FGSWMTDPLAPEGDNR + Oxidation (M)
324	622.7889	1243.5632	1243.6157	-42.15	1	1	34	1	KGPGAASALDDSR
218	530.3070	1058.5994	1058.5794	18.9	0	1	51	1	IMSIPDVIR + Oxidation (M)
193	520.2688	1038.5230	1038.5458	-21.88	0	1	50	1	AQASAPAQAPK
347	638.7794	1275.5442	1275.5666	-17.53	1	1	19	1	KMYSHPQQQN + Oxidation (M)
698	972.0858	2913.2356	2913.3199	-28.95	1	1	11	1	NAFERGEDPLAGDQNDHDMDSIAGVLK
614	761.7159	2282.1259	2282.0559	30.7	0	1	38	1	VALLEQQMQACTLDFENEK + Oxidation (M)
252	556.8272	1111.6398	1111.6349	4.41	1	1	31	1	KSPPQTVLSR
610	737.7059	2210.0959	2210.0967	-0.39	0	1	40	1	LGEHNINVLEGNEQFIDAAK
304	595.3141	1188.6136	1188.6714	-48.56	1	1	66	1	NETGLLSSIKK
491	809.4144	1616.8142	1616.7947	12.1	1	1	54	1	WESLHQEFSAKQK
301	595.3109	1188.6072	1188.5887	15.6	0	1	67	1	QLVGTNEWSR
41	325.1856	648.3566	648.3442	19.2	1	1	61	1	SAESKK
146	470.7728	939.5310	939.4886	45.2	1	1	37	1	TKHASGSPR
597	737.6961	2210.0665	2210.0160	22.9	1	1	42	1	ARGTGEPASAGTSTGTSTGAGSSSR
599	737.6978	2210.0716	2210.1167	-20.42	1	1	43	1	ALFASQEMWLSHRQTHLR
39	325.1848	648.3550	648.3629	-12.06	1	0	70	1	GLGMKK + Oxidation (M)
444	760.8801	1519.7456	1519.7493	-2.42	0	0	64	1	SETFLLMQPWPR + Oxidation (M)
441	758.4306	1514.8466	1514.8000	30.8	1	0	42	1	APANCRLPARPHR
38	325.1841	648.3536	648.3595	-9.02	0	0	76	1	AFNAVK
618	762.0477	2283.1213	2283.2158	-41.38	1	0	46	1	TSGRGHPDVATMLNILALVYR
311	601.3107	1200.6068	1200.5748	26.7	1	0	75	1	GAAGWEGGGRGAR
40	325.1851	648.3556	648.3629	-11.14	1	0	75	1	GLGMKK + Oxidation (M)
1	*366.1466*	*365.1393*							
2	*396.9587*	*395.9514*							
3	*429.0963*	*428.0890*							
4	*429.1010*	*428.0937*							
5	*433.1578*	*432.1505*							
6	*445.1280*	*444.1207*							
7	*445.1297*	*444.1224*							
8	*445.1974*	*444.1901*							
9	*445.1976*	*444.1903*							
10	*446.2410*	*445.2337*							
11	*451.1967*	*450.1894*							
12	*451.2156*	*450.2083*							
13	*460.2441*	*459.2368*							
14	*469.2228*	*468.2155*							
15	*471.2780*	*470.2707*							
16	*480.2485*	*479.2412*							
17	*487.3112*	*486.3039*							
18	*493.2528*	*492.2455*							
20	*506.2326*	*505.2253*							
21	*506.2362*	*505.2289*							
22	*506.2410*	*505.2337*							
23	*515.2794*	*514.2721*							
24	*531.3441*	*530.3368*							
29	*574.3152*	*573.3079*							
30	*575.2928*	*574.2855*							
31	*585.2461*	*584.2388*							
34	*609.0488*	*608.0415*							
37	*631.3204*	*630.3131*							
42	*653.3390*	*652.3317*							
43	*698.3687*	*697.3614*							
44	*698.3768*	*697.3695*							
45	*360.7057*	*719.3968*							
46	*360.7073*	*719.4000*							
48	*732.3607*	*731.3534*							
49	*367.4966*	*732.9786*							
50	*373.6020*	*745.1894*							
51	*756.3926*	*755.3853*							
54	*399.7273*	*797.4400*							
55	*800.3978*	*799.3905*							
57	*405.7203*	*809.4260*							
58	*810.4362*	*809.4289*							
59	*810.4495*	*809.4422*							
62	*408.2381*	*814.4616*							
64	*408.2433*	*814.4720*							
65	*816.8939*	*815.8866*							
69	*825.3708*	*824.3635*							
77	*834.3805*	*833.3732*							
87	*441.2735*	*880.5324*							
95	*450.7193*	*899.4240*							
96	*450.7201*	*899.4256*							
113	*450.7239*	*899.4332*							
117	*452.2079*	*902.4012*							
124	*456.2869*	*910.5592*							
127	*463.2484*	*924.4822*							
128	*464.7739*	*927.5332*							
129	*464.7761*	*927.5376*							
149	*961.4308*	*960.4235*							

☑	156	489.2438	976.4730
☑	158	491.2500	980.4854
☑	170	501.7578	1001.5010
☑	172	506.2320	1010.4494
☑	174	506.2326	1010.4506
☑	175	506.2336	1010.4526
☑	178	506.2391	1010.4636
☑	183	510.1425	1018.2704
☑	186	510.2555	1018.4964
☑	188	514.7734	1027.5322
☑	192	519.1511	1036.2876
☑	233	531.3477	1060.6808
☑	236	538.1265	1074.2384
☑	249	547.1017	1092.1888
☑	250	556.8207	1111.6268
☑	251	556.8255	1111.6364
☑	254	556.8296	1111.6446
☑	256	556.8333	1111.6520
☑	258	557.3193	1112.6240
☑	261	558.2656	1114.5166
☑	264	561.2626	1120.5106
☑	272	572.3195	1142.6244
☑	284	579.8302	1157.6458
☑	291	588.3398	1174.6650
☑	292	592.3085	1182.6024
☑	293	595.3043	1188.5940
☑	296	595.3077	1188.6008
☑	297	595.3081	1188.6016
☑	298	595.3089	1188.6032
☑	299	595.3093	1188.6040
☑	300	595.3095	1188.6044
☑	303	595.3124	1188.6102
☑	320	622.7780	1243.5414
☑	323	622.7885	1243.5624
☑	329	622.7941	1243.5736
☑	330	622.7943	1243.5740
☑	332	624.3058	1246.5970
☑	350	645.3189	1288.6232
☑	351	645.8275	1289.6404
☑	352	645.8320	1289.6494
☑	355	651.8354	1301.6562
☑	396	672.8417	1343.6688
☑	400	683.9808	1365.9470
☑	406	696.3622	1390.7098
☑	408	699.8883	1397.7620
☑	409	699.8900	1397.7654
☑	410	699.8941	1397.7736
☑	413	702.3684	1402.7222
☑	421	728.3550	1454.6954
☑	426	729.4035	1456.7924
☑	427	732.3361	1462.6576
☑	435	740.3751	1478.7356
☑	436	748.8556	1495.6966
☑	438	757.8587	1513.7028
☑	439	757.8616	1513.7086
☑	440	757.8618	1513.7090
☑	445	766.8646	1531.7146
☑	446	766.8663	1531.7180
☑	447	775.8678	1549.7210
☑	448	775.8699	1549.7252
☑	449	785.8380	1569.6614
☑	450	785.8396	1569.6646
☑	451	785.8400	1569.6654
☑	452	785.8427	1569.6708
☑	453	785.8429	1569.6712
☑	454	785.8431	1569.6716
☑	455	785.8436	1569.6726
☑	456	785.8438	1569.6730
☑	457	785.8450	1569.6754
☑	458	785.8452	1569.6758
☑	459	785.8453	1569.6760
☑	460	785.8453	1569.6760
☑	461	785.8457	1569.6768
☑	462	785.8459	1569.6772
☑	463	785.8464	1569.6782
☑	464	785.8467	1569.6788
☑	465	785.8488	1569.6830
☑	466	785.8490	1569.6834
☑	467	785.8494	1569.6842
☑	468	785.8496	1569.6846
☑	469	785.8498	1569.6850

☑	470	785.8501	1569.6856
☑	471	785.8507	1569.6868
☑	472	785.8522	1569.6898
☑	473	785.8538	1569.6930
☑	474	785.8539	1569.6932
☑	475	794.8428	1587.6710
☑	476	794.8445	1587.6744
☑	478	795.3523	1588.6900
☑	489	808.9011	1615.7876
☑	494	811.3594	1620.7042
☑	495	811.3651	1620.7156
☑	498	816.8893	1631.7640
☑	499	816.8903	1631.7660
☑	502	816.8928	1631.7710
☑	525	816.8995	1631.7844
☑	536	852.4309	1702.8472
☑	538	858.4859	1714.9572
☑	541	885.9580	1769.9014
☑	543	890.3994	1778.7842
☑	544	893.3851	1784.7556
☑	553	897.9139	1793.8132
☑	555	906.4707	1810.9268
☑	563	644.9491	1931.8255
☑	564	973.5184	1945.0222
☑	565	650.9495	1949.8267
☑	574	1024.5062	2046.9978
☑	585	1048.9791	2095.9436
☑	586	701.6427	2101.9063
☑	587	701.6429	2101.9069
☑	589	707.3322	2118.9748
☑	591	1073.0271	2144.0396
☑	592	732.0169	2193.0289
☑	593	732.0259	2193.0559
☑	595	737.6949	2210.0629
☑	596	737.6956	2210.0650
☑	598	737.6977	2210.0713
☑	601	737.6991	2210.0755
☑	602	737.7005	2210.0797
☑	608	737.7048	2210.0926
☑	609	737.7054	2210.0944
☑	611	737.7095	2210.1067
☑	613	756.0691	2265.1855
☑	616	761.7196	2282.1370
☑	619	762.0505	2283.1297
☑	623	762.0542	2283.1408
☑	628	762.0628	2283.1666
☑	629	768.7164	2303.1274
☑	630	779.0418	2334.1036
☑	631	1168.5664	2335.1182
☑	633	779.7078	2336.1016
☑	636	779.7141	2336.1205
☑	638	779.7175	2336.1307
☑	639	779.7218	2336.1436
☑	642	784.0617	2349.1633
☑	654	888.7457	2663.2153
☑	673	888.7603	2663.2591
☑	678	916.4274	2746.2604
☑	679	916.7508	2747.2306
☑	680	917.0808	2748.2206
☑	681	917.0816	2748.2230
☑	682	933.4417	2797.3033
☑	683	933.4421	2797.3045
☑	684	933.4461	2797.3165
☑	686	933.4491	2797.3255
☑	687	933.4591	2797.3555
☑	689	933.7738	2798.2996
☑	690	933.7778	2798.3116
☑	694	961.4198	2881.2376
☑	695	961.4268	2881.2586
☑	696	961.4318	2881.2736
☑	697	961.7681	2882.2825
☑	699	972.0930	2913.2572
☑	700	972.0975	2913.2707
☑	701	972.4227	2914.2463
☑	703	1308.9585	3923.8537
☑	704	981.9737	3923.8657
☑	706	1308.9722	3923.8948
☑	707	982.2178	3924.8421
☑	708	1314.2834	3939.8284
☑	709	1130.9587	4519.8057
☑	710	1308.8093	5231.2081

Search Parameters

```
Type of search         : MS/MS Ion Search
Enzyme                 : Trypsin
Fixed modifications    : Carbamidomethyl (C)
Variable modifications : Oxidation (M)
Mass values            : Monoisotopic
Protein Mass           : Unrestricted
Peptide Mass Tolerance : ± 50 ppm
Fragment Mass Tolerance: ± 0.2 Da
Max Missed Cleavages   : 1
Instrument type        : ESI-QUAD-TOF
Number of queries      : 710
```

**Mascot:** http://www.matrixscience.com/

Mascot Search Results

```
User          : Dominic
Email         : dominic.kurian@bbsrc.ac.uk
Search title  : IPI Mouse_15-02-2010
MS data file  : D:\Peaklist\Dominic Kurian\QTofDK20010804 GATC P 0010882.pkl
Database      : IPI_mouse MOUSE_v3_79 (54943 sequences; 24917557 residues)
Timestamp     : 15 Feb 2011 at 15:23:00 GMT
Protein hits  : IPI00420725 Tax_Id=10090 Gene_Symbol=Camk2a Uncharacterized protein
                IPI00474502 Tax_Id=10090 Gene_Symbol=Camk2b Calcium/calmodulin-dependent protein kinase type II subunit beta
                IPI00109073 Tax_Id=10090 Gene_Symbol=Tubb4 Tubulin beta-4 chain
                IPI00169463 Tax_Id=10090 Gene_Symbol=Tubb2c Tubulin beta-2C chain
                IPI00124695 Tax_Id=10090 Gene_Symbol=Camk2g Isoform 1 of Calcium/calmodulin-dependent protein kinase type II subunit
                IPI00117352 Tax_Id=10090 Gene_Symbol=Tubb5 Tubulin beta-5 chain
                IPI00109061 Tax_Id=10090 Gene_Symbol=Tubb2b Tubulin beta-2B chain
                IPI00112251 Tax_Id=10090 Gene_Symbol=Tubb3 Tubulin beta-3 chain
                IPI00755181 Tax_Id=10090 Gene_Symbol=Krt10 keratin, type I cytoskeletal 10
                IPI00110753 Tax_Id=10090 Gene_Symbol=Tuba1a Tubulin alpha-1A chain
                IPI00112584 Tax_Id=10090 Gene_Symbol=Camk2d Isoform 1 of Calcium/calmodulin-dependent protein kinase type II subunit
                IPI00608020 Tax_Id=10090 Gene_Symbol=Ftl1 ferritin light chain 1
                IPI00122928 Tax_Id=10090 Gene_Symbol=Tubb6 Tubulin beta-6 chain
                IPI00347110 Tax_Id=10090 Gene_Symbol=Krt73 Keratin, type II cytoskeletal 73
                IPI00122048 Tax_Id=10090 Gene_Symbol=Atp1a3 Sodium/potassium-transporting ATPase subunit alpha-3
                IPI00138892 Tax_Id=10090 Gene_Symbol=Uba52 Ubiquitin-60S ribosomal protein L40
                IPI00625729 Tax_Id=10090 Gene_Symbol=Krt1 Keratin, type II cytoskeletal 1
                IPI00136703 Tax_Id=10090 Gene_Symbol=Ckb Creatine kinase B-type
                IPI00110850 Tax_Id=10090 Gene_Symbol=Actb Actin, cytoplasmic 1
                IPI00221797 Tax_Id=10090 Gene_Symbol=Krt75 Keratin, type II cytoskeletal 75
                IPI00348328 Tax_Id=10090 Gene_Symbol=Krt78 keratin Kb40
                IPI00322209 Tax_Id=10090 Gene_Symbol=Krt8 Keratin, type II cytoskeletal 8
                IPI00221528 Tax_Id=10090 Gene_Symbol=Actbl2 Beta-actin-like protein 2
                IPI00124499 Tax_Id=10090 Gene_Symbol=Krt79 Keratin, type II cytoskeletal 79
                IPI00230145 Tax_Id=10090 Gene_Symbol=Fth1 Ferritin heavy chain
                IPI00406377 Tax_Id=10090 Gene_Symbol=Krt7 Keratin, type II cytoskeletal 7
                IPI00222228 Tax_Id=10090 Gene_Symbol=4732456N10Rik hypothetical protein LOC239673
                IPI00135284 Tax_Id=10090 Gene_Symbol=Gm16374 Glyceraldehyde-3-phosphate dehydrogenase
                IPI00134997 Tax_Id=10090 Gene_Symbol=Dlgap3 Disks large-associated protein 3
                IPI00348094 Tax_Id=10090 Gene_Symbol=Tubb1 Tubulin, beta 1
                IPI00115546 Tax_Id=10090 Gene_Symbol=Gnao1 Isoform Alpha-2 of Guanine nucleotide-binding protein G(o) subunit alpha
                IPI00122094 Tax_Id=10090 Gene_Symbol=Dlg4 Isoform 2 of Disks large homolog 4
                IPI00119667 Tax_Id=10090 Gene_Symbol=Eef1a2 Elongation factor 1-alpha 2
                IPI00135197 Tax_Id=10090 Gene_Symbol=Krt20 Keratin, type I cytoskeletal 20
                IPI00467833 Tax_Id=10090 Gene_Symbol=Tpi1 triosephosphate isomerase
                IPI00118569 Tax_Id=10090 Gene_Symbol=Gna13 Guanine nucleotide-binding protein subunit alpha-13
                IPI00462072 Tax_Id=10090 Gene_Symbol=Eno1;Gm5506 Alpha-enolase
                IPI00120793 Tax_Id=10090 Gene_Symbol=Prnp Major prion protein
                IPI00113410 Tax_Id=10090 Gene_Symbol=Hapln2 Hyaluronan and proteoglycan link protein 2
                IPI00622240 Tax_Id=10090 Gene_Symbol=Krt2 Keratin, type II cytoskeletal 2 epidermal
```

	IPI_mouse	Decoy	False discovery rate
Peptide matches above identity threshold	164	0	0.00 %
Peptide matches above homology or identity threshold	182	0	0.00 %

Probability Based Mowse Score

Ions score is -10*Log(P), where P is the probability that the observed match is a random event.
Individual ions scores > 37 indicate identity or extensive homology (p<0.01).
Protein scores are derived from ions scores as a non-probabilistic basis for ranking protein hits.

Score Distribution

Peptide Summary Report

Format As | Peptide Summary | Help

Significance threshold p< 0.01 | Max. number of hits AUTO

Standard scoring | MudPIT scoring | Ions score or expect cut-off 0 | Show sub-sets 0

Show pop-ups | Suppress pop-ups | Sort unassigned Decreasing Score | Require bold red

Select All | Select None | Search Selected | **Error tolerant** | Archive Report

```
1.   IPI00420725   Mass: 55882   Score: 1121   Queries matched: 76   emPAI: 0.77
     Tax_Id=10090 Gene_Symbol=Camk2a Uncharacterized protein
     Check to include this hit in error tolerant search or archive report
```

	Query	Observed	Mr(expt)	Mr(calc)	ppm	Miss	Score	Expect	Rank	Peptide
☑	27	588.3868	587.3795	587.3642	26.0	0	22	0.71	1	K.IINTK.K
☑	69	432.7315	863.4484	863.4501	-1.94	0	23	0.56	1	R.SGAPSVLPH.-
☑	90	452.2513	902.4880	902.4895	-1.63	0	21	0.9	1	K.MLTINPSK.R
☑	139	525.2845	1048.5544	1048.5553	-0.80	0	(45)	0.0029	1	K.VLAGQEYAAK.I
☑	140	525.2850	1048.5554	1048.5553	0.16	0	(33)	0.044	1	K.VLAGQEYAAK.I
☑	141	525.2850	1048.5554	1048.5553	0.16	0	(39)	0.01	1	K.VLAGQEYAAK.I
☑	142	525.2855	1048.5564	1048.5553	1.11	0	54	0.00031	1	K.VLAGQEYAAK.I
☑	143	525.2856	1048.5566	1048.5553	1.30	0	(22)	0.49	1	K.VLAGQEYAAK.I
☑	144	525.2864	1048.5582	1048.5553	2.83	0	(47)	0.0015	1	K.VLAGQEYAAK.I
☑	201	574.3237	1146.6328	1146.6431	-8.91	0	(39)	0.0079	1	K.GAILTTMLATR.N
☑	202	574.3245	1146.6344	1146.6431	-7.52	0	(51)	0.00057	1	K.GAILTTMLATR.N
☑	204	574.3274	1146.6402	1146.6431	-2.46	0	(36)	0.014	1	K.GAILTTMLATR.N
☑	205	574.3279	1146.6412	1146.6431	-1.59	0	(49)	0.00074	1	K.GAILTTMLATR.N
☑	206	574.3290	1146.6434	1146.6431	0.33	0	(24)	0.21	1	K.GAILTTMLATR.N
☑	207	574.3298	1146.6450	1146.6431	1.73	0	63	2.6e-005	1	K.GAILTTMLATR.N
☑	216	582.3226	1162.6306	1162.6380	-6.31	0	(28)	0.097	1	K.GAILTTMLATR.N + Oxidation (M)
☑	256	631.3042	1260.5938	1260.5927	0.88	0	62	4.5e-005	1	R.FYFENLWSR.N
☑	257	631.3046	1260.5946	1260.5927	1.51	0	(49)	0.00083	1	R.FYFENLWSR.N
☑	258	631.3048	1260.5950	1260.5927	1.83	0	(54)	0.00028	1	R.FYFENLWSR.N
☑	259	631.3049	1260.5952	1260.5927	1.99	0	(58)	0.00011	1	R.FYFENLWSR.N
☑	260	631.3054	1260.5962	1260.5927	2.78	0	(58)	0.00011	1	R.FYFENLWSR.N
☑	261	631.3054	1260.5962	1260.5927	2.78	0	(37)	0.012	1	R.FYFENLWSR.N
☑	262	631.3058	1260.5970	1260.5927	3.42	0	(41)	0.0055	1	R.FYFENLWSR.N
☑	263	631.3059	1260.5972	1260.5927	3.57	0	(58)	0.00011	1	R.FYFENLWSR.N
☑	264	631.3059	1260.5972	1260.5927	3.57	0	(35)	0.022	1	R.FYFENLWSR.N
☑	265	631.3066	1260.5986	1260.5927	4.68	0	(29)	0.086	1	R.FYFENLWSR.N
☑	266	631.3085	1260.6024	1260.5927	7.70	0	(59)	9.9e-005	1	R.FYFENLWSR.N
	268	631.3294	1260.6442	1260.5927	40.9	0	(4)	35	3	R.FYFENLWSR.N
☑	294	652.3510	1302.6874	1302.6932	-4.41	0	(72)	5e-006	1	R.ITQYLDAGGIPR.T
☑	295	652.3511	1302.6876	1302.6932	-4.26	0	(72)	5.2e-006	1	R.ITQYLDAGGIPR.T
☑	296	652.3549	1302.6952	1302.6932	1.58	0	(60)	6.7e-005	1	R.ITQYLDAGGIPR.T
☑	297	652.3553	1302.6960	1302.6932	2.19	0	(69)	8.8e-006	1	R.ITQYLDAGGIPR.T
☑	298	652.3554	1302.6962	1302.6932	2.35	0	(77)	1.4e-006	1	R.ITQYLDAGGIPR.T
☑	299	652.3554	1302.6962	1302.6932	2.35	0	77	1.3e-006	1	R.ITQYLDAGGIPR.T
☑	300	652.3558	1302.6970	1302.6932	2.96	0	(77)	1.5e-006	1	R.ITQYLDAGGIPR.T
☑	301	652.3565	1302.6984	1302.6932	4.03	0	(69)	9.5e-006	1	R.ITQYLDAGGIPR.T
☑	491	816.8912	1631.7678	1631.7719	-2.47	0	(42)	0.0039	1	R.FTEEYQLFEELGK.G
☑	492	816.8919	1631.7692	1631.7719	-1.61	0	(33)	0.034	1	R.FTEEYQLFEELGK.G
☑	493	816.8925	1631.7704	1631.7719	-0.88	0	(18)	1	1	R.FTEEYQLFEELGK.G
☑	494	816.8930	1631.7714	1631.7719	-0.27	0	(64)	2.4e-005	1	R.FTEEYQLFEELGK.G
☑	495	816.8931	1631.7716	1631.7719	-0.14	0	(38)	0.011	1	R.FTEEYQLFEELGK.G
☑	496	816.8934	1631.7722	1631.7719	0.22	0	(5)	20	1	R.FTEEYQLFEELGK.G
☑	498	816.8940	1631.7734	1631.7719	0.96	0	(65)	1.8e-005	1	R.FTEEYQLFEELGK.G
☑	500	816.8945	1631.7744	1631.7719	1.57	0	(43)	0.003	1	R.FTEEYQLFEELGK.G
☑	501	816.8950	1631.7754	1631.7719	2.19	0	(48)	0.001	1	R.FTEEYQLFEELGK.G
☑	503	816.8951	1631.7756	1631.7719	2.31	0	70	5.7e-006	1	R.FTEEYQLFEELGK.G
☑	504	816.8952	1631.7758	1631.7719	2.43	0	(3)	29	1	R.FTEEYQLFEELGK.G
☑	505	816.8954	1631.7762	1631.7719	2.68	0	(64)	2.8e-005	1	R.FTEEYQLFEELGK.G
☑	506	816.8964	1631.7782	1631.7719	3.90	0	(23)	0.35	1	R.FTEEYQLFEELGK.G
☑	507	816.8967	1631.7788	1631.7719	4.27	0	(63)	2.9e-005	1	R.FTEEYQLFEELGK.G
☑	508	816.8970	1631.7794	1631.7719	4.64	0	(48)	0.001	1	R.FTEEYQLFEELGK.G
☑	509	816.8970	1631.7794	1631.7719	4.64	0	(37)	0.014	1	R.FTEEYQLFEELGK.G
☑	510	816.8972	1631.7798	1631.7719	4.88	0	(50)	0.00068	1	R.FTEEYQLFEELGK.G
☑	511	816.8972	1631.7798	1631.7719	4.88	0	(38)	0.0091	1	R.FTEEYQLFEELGK.G
☑	512	816.8990	1631.7834	1631.7719	7.09	0	(37)	0.012	1	R.FTEEYQLFEELGK.G
☑	513	816.8997	1631.7848	1631.7719	7.95	0	(2)	42	1	R.FTEEYQLFEELGK.G
☑	646	694.3165	2079.9277	2079.9426	-7.15	0	(51)	0.0003	1	K.AGAYDFPSPEWDTVTPEAK.D
☑	647	694.3185	2079.9337	2079.9426	-4.27	0	(60)	3.8e-005	1	K.AGAYDFPSPEWDTVTPEAK.D
☑	648	1040.9807	2079.9468	2079.9426	2.07	0	(60)	4.5e-005	1	K.AGAYDFPSPEWDTVTPEAK.D
☑	649	1040.9808	2079.9470	2079.9426	2.16	0	(43)	0.0021	1	K.AGAYDFPSPEWDTVTPEAK.D
☑	650	1040.9817	2079.9488	2079.9426	3.03	0	(53)	0.0002	1	K.AGAYDFPSPEWDTVTPEAK.D
☑	651	1040.9820	2079.9494	2079.9426	3.32	0	(56)	0.00011	1	K.AGAYDFPSPEWDTVTPEAK.D
☑	652	1040.9820	2079.9494	2079.9426	3.32	0	(50)	0.00046	1	K.AGAYDFPSPEWDTVTPEAK.D
☑	653	1040.9828	2079.9510	2079.9426	4.09	0	(45)	0.0013	1	K.AGAYDFPSPEWDTVTPEAK.D
☑	654	1040.9838	2079.9530	2079.9426	5.05	0	74	1.9e-006	1	K.AGAYDFPSPEWDTVTPEAK.D
☑	655	1040.9847	2079.9548	2079.9426	5.91	0	(34)	0.019	1	K.AGAYDFPSPEWDTVTPEAK.D
☑	662	715.3441	2143.0105	2143.0321	-10.08	0	61	4.1e-005	1	K.VTEQLIEAISNGDFESYTK.M
☑	663	1072.5222	2143.0298	2143.0321	-1.04	0	(38)	0.0075	1	K.VTEQLIEAISNGDFESYTK.M
☑	664	1072.5251	2143.0356	2143.0321	1.67	0	(54)	0.00019	1	K.VTEQLIEAISNGDFESYTK.M
☑	665	1072.5334	2143.0522	2143.0321	9.41	0	(11)	4.6	1	K.VTEQLIEAISNGDFESYTK.M
☑	696	888.7555	2663.2447	2663.2755	-11.57	1	(27)	0.084	1	K.AGAYDFPSPEWDTVTPEAKDLINK.M
☑	697	888.7577	2663.2513	2663.2755	-9.10	1	(8)	6.2	1	K.AGAYDFPSPEWDTVTPEAKDLINK.M
☑	698	888.7581	2663.2525	2663.2755	-8.65	1	(5)	14	1	K.AGAYDFPSPEWDTVTPEAKDLINK.M
☑	699	888.7607	2663.2603	2663.2755	-5.72	1	(22)	0.24	1	K.AGAYDFPSPEWDTVTPEAKDLINK.M
☑	701	888.7623	2663.2651	2663.2755	-3.91	1	(8)	7.1	1	K.AGAYDFPSPEWDTVTPEAKDLINK.M
☑	703	888.7635	2663.2687	2663.2755	-2.56	1	37	0.0079	1	K.AGAYDFPSPEWDTVTPEAKDLINK.M

Proteins matching the same set of peptides:

IPI00621806 **Mass:** 54651 **Score:** 1121 **Queries matched:** 76
Tax_Id=10090 Gene_Symbol=Camk2a Isoform Alpha CaMKII of Calcium/calmodulin-dependent protein kinase type II subunit alpha

---

2. IPI00474502 **Mass:** 61164 **Score:** 595 **Queries matched:** 38 **emPAI:** 0.52
Tax_Id=10090 Gene_Symbol=Camk2b Calcium/calmodulin-dependent protein kinase type II subunit beta
☐ Check to include this hit in error tolerant search or archive report

	Query	Observed	Mr(expt)	Mr(calc)	ppm	Miss	Score	Expect	Rank	Peptide
	27	588.3868	587.3795	587.3642	26.0	0	22	0.71	1	K.IINTK.K
☑	193	572.8034	1143.5922	1143.5964	-3.65	0	(13)	4.4	1	R.FYFENLLAK.N
☑	194	572.8046	1143.5946	1143.5964	-1.55	0	(19)	1	1	R.FYFENLLAK.N
☑	196	572.8075	1143.6004	1143.5964	3.52	0	22	0.51	1	R.FYFENLLAK.N
☑	197	572.8082	1143.6018	1143.5964	4.75	0	(12)	4.9	1	R.FYFENLLAK.N
	198	572.8111	1143.6076	1143.5964	9.82	0	(4)	32	2	R.FYFENLLAK.N
	201	574.3237	1146.6328	1146.6431	-8.91	0	(39)	0.0079	1	K.GAILTTMLATR.N
	202	574.3245	1146.6344	1146.6431	-7.52	0	(51)	0.00057	1	K.GAILTTMLATR.N
	204	574.3274	1146.6402	1146.6431	-2.46	0	(36)	0.014	1	K.GAILTTMLATR.N
	205	574.3279	1146.6412	1146.6431	-1.59	0	(49)	0.00074	1	K.GAILTTMLATR.N
	206	574.3290	1146.6434	1146.6431	0.33	0	(24)	0.21	1	K.GAILTTMLATR.N
	207	574.3298	1146.6450	1146.6431	1.73	0	63	2.6e-005	1	K.GAILTTMLATR.N
	216	582.3226	1162.6306	1162.6380	-6.31	0	(28)	0.097	1	K.GAILTTMLATR.N + Oxidation (M)
☑	372	735.4076	1468.8006	1468.8071	-4.42	0	(18)	0.75	1	K.NLINQMLTINPAK.R
☑	373	735.4100	1468.8054	1468.8071	-1.15	0	(63)	2.7e-005	1	K.NLINQMLTINPAK.R
☑	374	735.4109	1468.8072	1468.8071	0.07	0	75	1.7e-006	1	K.NLINQMLTINPAK.R
☑	375	735.4146	1468.8146	1468.8071	5.11	0	(62)	3.1e-005	1	K.NLINQMLTINPAK.R
☑	376	735.4150	1468.8154	1468.8071	5.65	0	(71)	3.6e-006	1	K.NLINQMLTINPAK.R
☑	480	810.8856	1619.7566	1619.7355	13.1	0	(4)	26	1	R.FTDEYQLYEDIGK.G
☑	481	810.8877	1619.7608	1619.7355	15.6	0	8	9.3	1	R.FTDEYQLYEDIGK.G
☑	632	656.9538	1967.8396	1967.8556	-8.12	1	52	0.00014	1	K.ESSDSTNTTIEDEDAKAR.K
	646	694.3165	2079.9277	2079.9426	-7.15	0	(51)	0.0003	1	K.AGAYDFPSPEWDTVTPEAK.N
	647	694.3185	2079.9337	2079.9426	-4.27	0	(60)	3.8e-005	1	K.AGAYDFPSPEWDTVTPEAK.N
	648	1040.9807	2079.9468	2079.9426	2.07	0	(60)	4.5e-005	1	K.AGAYDFPSPEWDTVTPEAK.N
	649	1040.9808	2079.9470	2079.9426	2.16	0	(43)	0.0021	1	K.AGAYDFPSPEWDTVTPEAK.N
	650	1040.9817	2079.9488	2079.9426	3.03	0	(53)	0.0002	1	K.AGAYDFPSPEWDTVTPEAK.N
	651	1040.9820	2079.9494	2079.9426	3.32	0	(56)	0.00011	1	K.AGAYDFPSPEWDTVTPEAK.N
	652	1040.9820	2079.9494	2079.9426	3.32	0	(50)	0.00046	1	K.AGAYDFPSPEWDTVTPEAK.N
	653	1040.9828	2079.9510	2079.9426	4.09	0	(45)	0.0013	1	K.AGAYDFPSPEWDTVTPEAK.N
	654	1040.9838	2079.9530	2079.9426	5.05	0	74	1.9e-006	1	K.AGAYDFPSPEWDTVTPEAK.N
	655	1040.9847	2079.9548	2079.9426	5.91	0	(34)	0.019	1	K.AGAYDFPSPEWDTVTPEAK.N
☑	676	785.0440	2352.1102	2352.1301	-8.48	0	(91)	3.4e-008	1	R.QTTAPATMSTAASGTTMGLVEQAK.S
☑	677	785.0472	2352.1198	2352.1301	-4.40	0	(90)	3.9e-008	1	R.QTTAPATMSTAASGTTMGLVEQAK.S
☑	678	1177.0771	2352.1396	2352.1301	4.05	0	(32)	0.025	1	R.QTTAPATMSTAASGTTMGLVEQAK.S
☑	679	1177.0814	2352.1482	2352.1301	7.71	0	91	3.4e-008	1	R.QTTAPATMSTAASGTTMGLVEQAK.S
☑	681	785.4148	2353.2226	2353.2642	-17.67	0	(13)	1.9	1	K.GSLPPAALEPQTTVIHNPVDGIK.E
☑	682	785.4241	2353.2505	2353.2642	-5.82	0	14	1.5	1	K.GSLPPAALEPQTTVIHNPVDGIK.E
☑	685	790.3805	2368.1197	2368.1250	-2.26	0	(42)	0.0024	1	R.QTTAPATMSTAASGTTMGLVEQAK.S + Oxidation (M)

Proteins matching the same set of peptides:
IPI00875723 **Mass:** 68686 **Score:** 595 **Queries matched:** 38
Tax_Id=10090 Gene_Symbol=Camk2b 68 kDa protein

---

3. IPI00109073 **Mass:** 50010 **Score:** 415 **Queries matched:** 22 **emPAI:** 0.38
Tax_Id=10090 Gene_Symbol=Tubb4 Tubulin beta-4 chain
☐ Check to include this hit in error tolerant search or archive report

	Query	Observed	Mr(expt)	Mr(calc)	ppm	Miss	Score	Expect	Rank	Peptide
☑	191	572.3217	1142.6288	1142.6270	1.59	0	69	1.1e-005	1	K.LAVNMVPFPR.L
☑	192	572.3229	1142.6312	1142.6270	3.69	0	(38)	0.013	1	K.LAVNMVPFPR.L
☑	308	660.3503	1318.6860	1318.6955	-7.18	0	(63)	4.2e-005	1	R.IMNTFSVVPSPK.V
☑	309	660.3552	1318.6958	1318.6955	0.26	0	(32)	0.048	1	R.IMNTFSVVPSPK.V
☑	310	660.3563	1318.6980	1318.6955	1.92	0	(71)	6.3e-006	1	R.IMNTFSVVPSPK.V
☑	311	660.3572	1318.6998	1318.6955	3.29	0	104	2.8e-009	1	R.IMNTFSVVPSPK.V
☑	454	801.4125	1600.8104	1600.8131	-1.63	0	(65)	2.2e-005	1	R.AVLVDLEPGTMDSVR.S
☑	455	801.4150	1600.8154	1600.8131	1.49	0	79	8.4e-007	1	R.AVLVDLEPGTMDSVR.S
☑	456	801.4152	1600.8158	1600.8131	1.74	0	(59)	8.6e-005	1	R.AVLVDLEPGTMDSVR.S
☑	457	801.4156	1600.8166	1600.8131	2.24	0	(60)	6.8e-005	1	R.AVLVDLEPGTMDSVR.S
☑	458	801.4175	1600.8204	1600.8131	4.61	0	(65)	1.8e-005	1	R.AVLVDLEPGTMDSVR.S
☑	475	809.4103	1616.8060	1616.8080	-1.20	0	(36)	0.017	1	R.AVLVDLEPGTMDSVR.S + Oxidation (M)
	521	846.4363	1690.8580	1690.8600	-1.16	0	(13)	3.3	2	R.ALTVPELTQQMFDAK.N
☑	522	846.4375	1690.8604	1690.8600	0.26	0	(34)	0.027	1	R.ALTVPELTQQMFDAK.N
☑	523	846.4381	1690.8616	1690.8600	0.97	0	(28)	0.099	1	R.ALTVPELTQQMFDAK.N
☑	524	846.4382	1690.8618	1690.8600	1.09	0	53	0.00034	1	R.ALTVPELTQQMFDAK.N
☑	526	848.9185	1695.8224	1695.8257	-1.89	0	12	3.8	1	K.NSSYFVEWIPNNVK.T
	558	854.4335	1706.8524	1706.8549	-1.45	0	(28)	0.11	2	R.ALTVPELTQQMFDAK.N + Oxidation (M)
	560	854.4359	1706.8572	1706.8549	1.36	0	(16)	1.5	2	R.ALTVPELTQQMFDAK.N + Oxidation (M)
☑	601	915.4471	1828.8796	1828.8744	2.87	0	65	2e-005	1	R.INVYYNEATGGNYVPR.A
☑	602	915.4512	1828.8878	1828.8744	7.36	0	(41)	0.0042	1	R.INVYYNEATGGNYVPR.A
	603	915.4561	1828.8976	1828.8744	12.7	0	(5)	18	4	R.INVYYNEATGGNYVPR.A

---

4. IPI00169463 **Mass:** 50255 **Score:** 404 **Queries matched:** 20 **emPAI:** 0.37
Tax_Id=10090 Gene_Symbol=Tubb2c Tubulin beta-2C chain
☐ Check to include this hit in error tolerant search or archive report

Query	Observed	Mr(expt)	Mr(calc)	ppm	Miss	Score	Expect	Rank	Peptide

Query	Observed	Mr(expt)	Mr(calc)	ppm	Miss	Score	Expect	Rank	Peptide
191	572.3217	1142.6288	1142.6270	1.59	0	69	1.1e-005	1	K.LAVNMVPFPR.L
192	572.3229	1142.6312	1142.6270	3.69	0	(38)	0.013	1	K.LAVNMVPFPR.L
308	660.3503	1318.6860	1318.6955	-7.18	0	(63)	4.2e-005	1	R.IMNTFSVVPSPK.V
309	660.3552	1318.6958	1318.6955	0.26	0	(32)	0.048	1	R.IMNTFSVVPSPK.V
310	660.3563	1318.6980	1318.6955	1.92	0	(71)	6.3e-006	1	R.IMNTFSVVPSPK.V
311	660.3572	1318.6998	1318.6955	3.29	0	104	2.8e-009	1	R.IMNTFSVVPSPK.V
**363**	**723.8521**	**1445.6896**	**1445.6820**	**5.28**	**0**	**61**	**5.4e-005**	**1**	**K.EVDEQMLNVQNK.N**
454	801.4125	1600.8104	1600.8131	-1.63	0	(65)	2.2e-005	1	R.AVLVDLEPGTMDSVR.S
455	801.4150	1600.8154	1600.8131	1.49	0	79	8.4e-007	1	R.AVLVDLEPGTMDSVR.S
456	801.4152	1600.8158	1600.8131	1.74	0	(59)	8.6e-005	1	R.AVLVDLEPGTMDSVR.S
457	801.4156	1600.8166	1600.8131	2.24	0	(60)	6.8e-005	1	R.AVLVDLEPGTMDSVR.S
458	801.4175	1600.8204	1600.8131	4.61	0	(65)	1.8e-005	1	R.AVLVDLEPGTMDSVR.S
475	809.4103	1616.8060	1616.8080	-1.20	0	(36)	0.017	1	R.AVLVDLEPGTMDSVR.S + Oxidation (M)
521	846.4363	1690.8580	1690.8600	-1.16	0	(13)	3.3	2	R.ALTVPELTQQMFDAK.N
522	846.4375	1690.8604	1690.8600	0.26	0	(34)	0.027	1	R.ALTVPELTQQMFDAK.N
523	846.4381	1690.8616	1690.8600	0.97	0	(28)	0.099	1	R.ALTVPELTQQMFDAK.N
524	846.4382	1690.8618	1690.8600	1.09	0	53	0.00034	1	R.ALTVPELTQQMFDAK.N
526	848.9185	1695.8224	1695.8257	-1.89	0	12	3.8	1	K.NSSYFVEWIPNNVK.T
558	854.4335	1706.8524	1706.8549	-1.45	0	(28)	0.11	2	R.ALTVPELTQQMFDAK.N + Oxidation (M)
560	854.4359	1706.8572	1706.8549	1.36	0	(16)	1.5	2	R.ALTVPELTQQMFDAK.N + Oxidation (M)

5. IPI00124695 **Mass:** 60254 **Score:** 386 **Queries matched:** 19 **emPAI:** 0.24
Tax_Id=10090 Gene_Symbol=Camk2g Isoform 1 of Calcium/calmodulin-dependent protein kinase type II subunit gamma
Check to include this hit in error tolerant search or archive report

Query	Observed	Mr(expt)	Mr(calc)	ppm	Miss	Score	Expect	Rank	Peptide
27	588.3868	587.3795	587.3642	26.0	0	22	0.71	1	K.IINTK.K
372	735.4076	1468.8006	1468.8071	-4.42	0	(18)	0.75	1	K.NLINQMLTINPAK.R
373	735.4100	1468.8054	1468.8071	-1.15	0	(63)	2.7e-005	1	K.NLINQMLTINPAK.R
374	735.4109	1468.8072	1468.8071	0.07	0	75	1.7e-006	1	K.NLINQMLTINPAK.R
375	735.4146	1468.8146	1468.8071	5.11	0	(62)	3.1e-005	1	K.NLINQMLTINPAK.R
376	735.4150	1468.8154	1468.8071	5.65	0	(71)	3.6e-006	1	K.NLINQMLTINPAK.R
**463**	**802.8795**	**1603.7444**	**1603.7406**	**2.40**	**0**	**51**	**0.00037**	**1**	**R.FTDDYQLFEELGK.G**
**464**	**802.8842**	**1603.7538**	**1603.7406**	**8.26**	**0**	**(29)**	**0.074**	**1**	**R.FTDDYQLFEELGK.G**
**465**	**802.8851**	**1603.7556**	**1603.7406**	**9.38**	**0**	**(37)**	**0.011**	**1**	**R.FTDDYQLFEELGK.G**
646	694.3165	2079.9277	2079.9426	-7.15	0	(51)	0.0003	1	K.AGAYDFPSPEWDTVTPEAK.N
647	694.3185	2079.9337	2079.9426	-4.27	0	(60)	3.8e-005	1	K.AGAYDFPSPEWDTVTPEAK.N
648	1040.9807	2079.9468	2079.9426	2.07	0	(60)	4.5e-005	1	K.AGAYDFPSPEWDTVTPEAK.N
649	1040.9808	2079.9470	2079.9426	2.16	0	(43)	0.0021	1	K.AGAYDFPSPEWDTVTPEAK.N
650	1040.9817	2079.9488	2079.9426	3.03	0	(53)	0.0002	1	K.AGAYDFPSPEWDTVTPEAK.N
651	1040.9820	2079.9494	2079.9426	3.32	0	(56)	0.00011	1	K.AGAYDFPSPEWDTVTPEAK.N
652	1040.9820	2079.9494	2079.9426	3.32	0	(50)	0.00046	1	K.AGAYDFPSPEWDTVTPEAK.N
653	1040.9828	2079.9510	2079.9426	4.09	0	(45)	0.0013	1	K.AGAYDFPSPEWDTVTPEAK.N
654	1040.9838	2079.9530	2079.9426	5.05	0	74	1.9e-006	1	K.AGAYDFPSPEWDTVTPEAK.N
655	1040.9847	2079.9548	2079.9426	5.91	0	(34)	0.019	1	K.AGAYDFPSPEWDTVTPEAK.N

Proteins matching the same set of peptides:
IPI00228044 **Mass:** 59013 **Score:** 386 **Queries matched:** 19
Tax_Id=10090 Gene_Symbol=Camk2g Isoform 2 of Calcium/calmodulin-dependent protein kinase type II subunit gamma
IPI00228045 **Mass:** 56610 **Score:** 386 **Queries matched:** 19
Tax_Id=10090 Gene_Symbol=Camk2g Isoform 3 of Calcium/calmodulin-dependent protein kinase type II subunit gamma

6. IPI00117352 **Mass:** 50095 **Score:** 381 **Queries matched:** 16 **emPAI:** 0.38
Tax_Id=10090 Gene_Symbol=Tubb5 Tubulin beta-5 chain
Check to include this hit in error tolerant search or archive report

Query	Observed	Mr(expt)	Mr(calc)	ppm	Miss	Score	Expect	Rank	Peptide
191	572.3217	1142.6288	1142.6270	1.59	0	69	1.1e-005	1	K.LAVNMVPFPR.L
192	572.3229	1142.6312	1142.6270	3.69	0	(38)	0.013	1	K.LAVNMVPFPR.L
308	660.3503	1318.6860	1318.6955	-7.18	0	(63)	4.2e-005	1	R.IMNTFSVVPSPK.V
309	660.3552	1318.6958	1318.6955	0.26	0	(32)	0.048	1	R.IMNTFSVVPSPK.V
310	660.3563	1318.6980	1318.6955	1.92	0	(71)	6.3e-006	1	R.IMNTFSVVPSPK.V
311	660.3572	1318.6998	1318.6955	3.29	0	104	2.8e-009	1	R.IMNTFSVVPSPK.V
363	723.8521	1445.6896	1445.6820	5.28	0	61	5.4e-005	1	K.EVDEQMLNVQNK.N
**468**	**808.4218**	**1614.8290**	**1614.8287**	**0.21**	**0**	**(36)**	**0.02**	**1**	**R.AILVDLEPGTMDSVR.S**
**469**	**808.4241**	**1614.8336**	**1614.8287**	**3.06**	**0**	**(70)**	**7.9e-006**	**1**	**R.AILVDLEPGTMDSVR.S**
**470**	**808.4243**	**1614.8340**	**1614.8287**	**3.31**	**0**	**(60)**	**7.8e-005**	**1**	**R.AILVDLEPGTMDSVR.S**
**471**	**808.4246**	**1614.8346**	**1614.8287**	**3.68**	**0**	**70**	**7.2e-006**	**1**	**R.AILVDLEPGTMDSVR.S**
**489**	**816.4179**	**1630.8212**	**1630.8236**	**-1.46**	**0**	**(48)**	**0.0011**	**1**	**R.AILVDLEPGTMDSVR.S + Oxidation (M)**
**490**	**816.4210**	**1630.8274**	**1630.8236**	**2.34**	**0**	**(54)**	**0.00032**	**1**	**R.AILVDLEPGTMDSVR.S + Oxidation (M)**
**518**	**830.4456**	**1658.8766**	**1658.8879**	**-6.81**	**0**	**(13)**	**2.7**	**1**	**R.ALTVPELTQQVFDAK.N**
**519**	**830.4520**	**1658.8894**	**1658.8879**	**0.91**	**0**	**28**	**0.085**	**1**	**R.ALTVPELTQQVFDAK.N**
526	848.9185	1695.8224	1695.8257	-1.89	0	12	3.8	1	K.NSSYFVEWIPNNVK.T

7. IPI00109061 **Mass:** 50377 **Score:** 375 **Queries matched:** 16 **emPAI:** 0.46
Tax_Id=10090 Gene_Symbol=Tubb2b Tubulin beta-2B chain
Check to include this hit in error tolerant search or archive report

Query	Observed	Mr(expt)	Mr(calc)	ppm	Miss	Score	Expect	Rank	Peptide
191	572.3217	1142.6288	1142.6270	1.59	0	69	1.1e-005	1	K.LAVNMVPFPR.L
192	572.3229	1142.6312	1142.6270	3.69	0	(38)	0.013	1	K.LAVNMVPFPR.L
**330**	**676.3404**	**1350.6662**	**1350.6676**	**-0.98**	**0**	**(65)**	**2.1e-005**	**1**	**R.IMNTFSVMPSPK.V**
**331**	**676.3443**	**1350.6740**	**1350.6676**	**4.79**	**0**	**82**	**5.1e-007**	**1**	**R.IMNTFSVMPSPK.V**
**332**	**676.3445**	**1350.6744**	**1350.6676**	**5.09**	**0**	**(64)**	**3.2e-005**	**1**	**R.IMNTFSVMPSPK.V**
363	723.8521	1445.6896	1445.6820	5.28	0	61	5.4e-005	1	K.EVDEQMLNVQNK.N

	468	808.4218	1614.8290	1614.8287	0.21	0	(36)	0.02	1	R.AILVDLEPGTMDSVR.S
	469	808.4241	1614.8336	1614.8287	3.06	0	(70)	7.9e-006	1	R.AILVDLEPGTMDSVR.S
	470	808.4243	1614.8340	1614.8287	3.31	0	(60)	7.8e-005	1	R.AILVDLEPGTMDSVR.S
	471	808.4246	1614.8346	1614.8287	3.68	0	70	7.2e-006	1	R.AILVDLEPGTMDSVR.S
	489	816.4179	1630.8212	1630.8236	-1.46	0	(48)	0.0011	1	R.AILVDLEPGTMDSVR.S + Oxidation (M)
	490	816.4210	1630.8274	1630.8236	2.34	0	(54)	0.00032	1	R.AILVDLEPGTMDSVR.S + Oxidation (M)
	526	848.9185	1695.8224	1695.8257	-1.89	0	12	3.8	1	K.NSSYFVEWIPNNVK.T
☑	558	854.4335	1706.8524	1706.8549	-1.45	0	40	0.0068	1	R.ALTVPELTQQMFDSK.N
☑	**559**	**854.4358**	**1706.8570**	**1706.8549**	**1.25**	**0**	**(4)**	**27**	**1**	**R.ALTVPELTQQMFDSK.N**
☑	560	854.4359	1706.8572	1706.8549	1.36	0	(24)	0.24	1	R.ALTVPELTQQMFDSK.N

Proteins matching the same set of peptides:
IPI00338039 **Mass:** 50274 **Score:** 375 **Queries matched:** 16
Tax_Id=10090 Gene_Symbol=Tubb2a Tubulin beta-2A chain

---

8. IPI00112251 **Mass:** 50842 **Score:** 372 **Queries matched:** 19 **emPAI:** 0.37
Tax_Id=10090 Gene_Symbol=Tubb3 Tubulin beta-3 chain
☐ Check to include this hit in error tolerant search or archive report

Query	Observed	Mr(expt)	Mr(calc)	ppm	Miss	Score	Expect	Rank	Peptide
191	572.3217	1142.6288	1142.6270	1.59	0	69	1.1e-005	1	K.LAVNMVPFPR.L
192	572.3229	1142.6312	1142.6270	3.69	0	(38)	0.013	1	K.LAVNMVPFPR.L
308	660.3503	1318.6860	1318.6955	-7.18	0	(63)	4.2e-005	1	R.IMNTFSVVPSPK.V
309	660.3552	1318.6958	1318.6955	0.26	0	(32)	0.048	1	R.IMNTFSVVPSPK.V
310	660.3563	1318.6980	1318.6955	1.92	0	(71)	6.3e-006	1	R.IMNTFSVVPSPK.V
311	660.3572	1318.6998	1318.6955	3.29	0	104	2.8e-009	1	R.IMNTFSVVPSPK.V
468	808.4218	1614.8290	1614.8287	0.21	0	(36)	0.02	1	R.AILVDLEPGTMDSVR.S
469	808.4241	1614.8336	1614.8287	3.06	0	(70)	7.9e-006	1	R.AILVDLEPGTMDSVR.S
470	808.4243	1614.8340	1614.8287	3.31	0	(60)	7.8e-005	1	R.AILVDLEPGTMDSVR.S
471	808.4246	1614.8346	1614.8287	3.68	0	70	7.2e-006	1	R.AILVDLEPGTMDSVR.S
489	816.4179	1630.8212	1630.8236	-1.46	0	(48)	0.0011	1	R.AILVDLEPGTMDSVR.S + Oxidation (M)
490	816.4210	1630.8274	1630.8236	2.34	0	(54)	0.00032	1	R.AILVDLEPGTMDSVR.S + Oxidation (M)
521	846.4363	1690.8580	1690.8600	-1.16	0	(13)	3.3	2	R.ALTVPELTQQMFDAK.N
522	846.4375	1690.8604	1690.8600	0.26	0	(34)	0.027	1	R.ALTVPELTQQMFDAK.N
523	846.4381	1690.8616	1690.8600	0.97	0	(28)	0.099	1	R.ALTVPELTQQMFDAK.N
524	846.4382	1690.8618	1690.8600	1.09	0	53	0.00034	1	R.ALTVPELTQQMFDAK.N
526	848.9185	1695.8224	1695.8257	-1.89	0	12	3.8	1	K.NSSYFVEWIPNNVK.V
558	854.4335	1706.8524	1706.8549	-1.45	0	(28)	0.11	2	R.ALTVPELTQQMFDAK.N + Oxidation (M)
560	854.4359	1706.8572	1706.8549	1.36	0	(16)	1.5	2	R.ALTVPELTQQMFDAK.N + Oxidation (M)

---

9. IPI00755181 **Mass:** 57178 **Score:** 296 **Queries matched:** 11 **emPAI:** 0.32
Tax_Id=10090 Gene_Symbol=Krt10 keratin, type I cytoskeletal 10
☐ Check to include this hit in error tolerant search or archive report

	Query	Observed	Mr(expt)	Mr(calc)	ppm	Miss	Score	Expect	Rank	Peptide
☑	170	545.7681	1089.5216	1089.5237	-1.87	0	(65)	2.8e-005	1	K.VTMQNLNDR.L
☑	171	545.7692	1089.5238	1089.5237	0.15	0	83	4.1e-007	1	K.VTMQNLNDR.L
☑	175	553.7703	1105.5260	1105.5186	6.74	0	(35)	0.029	1	K.VTMQNLNDR.L + Oxidation (M)
☑	176	553.7716	1105.5286	1105.5186	9.09	0	(50)	0.00085	1	K.VTMQNLNDR.L + Oxidation (M)
☑	217	583.2936	1164.5726	1164.5775	-4.14	0	(36)	0.019	1	R.LENEIQTYR.S
☑	218	583.2956	1164.5766	1164.5775	-0.70	0	41	0.0064	1	R.LENEIQTYR.S
☑	340	691.3282	1380.6418	1380.6408	0.73	0	(52)	0.00037	1	R.ALEESNYELEGK.I
☑	341	691.3283	1380.6420	1380.6408	0.88	0	(74)	2.5e-006	1	R.ALEESNYELEGK.I
☑	342	691.3298	1380.6450	1380.6408	3.05	0	87	1.1e-007	1	R.ALEESNYELEGK.I
☑	343	691.3315	1380.6484	1380.6408	5.51	0	(55)	0.00017	1	R.ALEESNYELEGK.I
☑	353	695.8544	1389.6942	1389.6736	14.9	0	55	0.00024	1	K.QSLEASLAETEGR.Y

Proteins matching the same set of peptides:
IPI00798492 **Mass:** 57906 **Score:** 296 **Queries matched:** 11
Tax_Id=10090 Gene_Symbol=Krt10 Isoform 1 of Keratin, type I cytoskeletal 10
IPI00828744 **Mass:** 57197 **Score:** 296 **Queries matched:** 11
Tax_Id=10090 Gene_Symbol=Krt10 Isoform 2 of Keratin, type I cytoskeletal 10

---

10. IPI00110753 **Mass:** 50788 **Score:** 288 **Queries matched:** 21 **emPAI:** 0.06
Tax_Id=10090 Gene_Symbol=Tuba1a Tubulin alpha-1A chain
☐ Check to include this hit in error tolerant search or archive report

	Query	Observed	Mr(expt)	Mr(calc)	ppm	Miss	Score	Expect	Rank	Peptide
☑	529	851.4498	1700.8850	1700.8985	-7.92	0	(1)	45	1	R.AVFVDLEPTVIDEVR.T
☑	530	851.4507	1700.8868	1700.8985	-6.86	0	(58)	8.7e-005	1	R.AVFVDLEPTVIDEVR.T
☑	532	851.4516	1700.8886	1700.8985	-5.80	0	(60)	6.1e-005	1	R.AVFVDLEPTVIDEVR.T
☑	533	851.4528	1700.8910	1700.8985	-4.39	0	(70)	5.8e-006	1	R.AVFVDLEPTVIDEVR.T
☑	534	851.4536	1700.8926	1700.8985	-3.45	0	(57)	0.00013	1	R.AVFVDLEPTVIDEVR.T
	535	851.4545	1700.8944	1700.8985	-2.39	0	(1)	47	2	R.AVFVDLEPTVIDEVR.T
☑	536	851.4548	1700.8950	1700.8985	-2.04	0	(40)	0.0052	1	R.AVFVDLEPTVIDEVR.T
☑	537	851.4554	1700.8962	1700.8985	-1.33	0	(20)	0.6	1	R.AVFVDLEPTVIDEVR.T
☑	539	851.4562	1700.8978	1700.8985	-0.39	0	(38)	0.0081	1	R.AVFVDLEPTVIDEVR.T
☑	540	851.4565	1700.8984	1700.8985	-0.04	0	(21)	0.49	1	R.AVFVDLEPTVIDEVR.T
☑	541	851.4575	1700.9004	1700.8985	1.14	0	(44)	0.0021	1	R.AVFVDLEPTVIDEVR.T
☑	542	851.4576	1700.9006	1700.8985	1.25	0	(23)	0.3	1	R.AVFVDLEPTVIDEVR.T
☑	543	851.4583	1700.9020	1700.8985	2.08	0	(49)	0.00072	1	R.AVFVDLEPTVIDEVR.T
☑	544	851.4584	1700.9022	1700.8985	2.20	0	(5)	17	1	R.AVFVDLEPTVIDEVR.T
☑	545	851.4591	1700.9036	1700.8985	3.02	0	77	1.1e-006	1	R.AVFVDLEPTVIDEVR.T
☑	546	851.4612	1700.9078	1700.8985	5.49	0	(22)	0.33	1	R.AVFVDLEPTVIDEVR.T

	Query	Observed	Mr(expt)	Mr(calc)	ppm	Miss	Score	Expect	Rank	Peptide
☑	547	851.4636	1700.9126	1700.8985	8.31	0	(56)	0.00013	1	R.AVFVDLEPTVIDEVR.T
☑	548	851.4642	1700.9138	1700.8985	9.02	0	(46)	0.0012	1	R.AVFVDLEPTVIDEVR.T
☑	597	912.9963	1823.9780	1823.9782	-0.06	0	(12)	2.8	1	K.VGINYQPPTVVPGGDLAK.V
☑	598	912.9998	1823.9850	1823.9782	3.77	0	22	0.25	1	K.VGINYQPPTVVPGGDLAK.V
☑	635	1004.4624	2006.9102	2006.8858	12.2	0	22	0.25	1	K.TIGGGDDSFNTFFSETGAGK.H

Proteins matching the same set of peptides:
IPI00117348 **Mass:** 50804 **Score:** 288 **Queries matched:** 21
Tax_Id=10090 Gene_Symbol=Tuba1b Tubulin alpha-1B chain
IPI00403810 **Mass:** 50562 **Score:** 288 **Queries matched:** 21
Tax_Id=10090 Gene_Symbol=Tuba1c Tubulin alpha-1C chain

---

11. IPI00112584 **Mass:** 56961 **Score:** 284 **Queries matched:** 34 **emPAI:** 0.48
Tax_Id=10090 Gene_Symbol=Camk2d Isoform 1 of Calcium/calmodulin-dependent protein kinase type II subunit delta
☐ Check to include this hit in error tolerant search or archive report

	Query	Observed	Mr(expt)	Mr(calc)	ppm	Miss	Score	Expect	Rank	Peptide
	27	588.3868	587.3795	587.3642	26.0	0	22	0.71	1	K.IINTK.K
	162	539.2801	1076.5456	1076.5502	-4.23	0	(1)	72	5	K.IPTGQEYAAK.I
☑	163	539.2836	1076.5526	1076.5502	2.27	0	24	0.38	1	K.IPTGQEYAAK.I
	164	539.2836	1076.5526	1076.5502	2.27	0	(6)	21	2	K.IPTGQEYAAK.I
☑	183	559.7768	1117.5390	1117.5444	-4.78	0	35	0.025	1	R.FYFENALSK.S
☑	184	559.7831	1117.5516	1117.5444	6.50	0	(11)	6.4	1	R.FYFENALSK.S
	201	574.3237	1146.6328	1146.6431	-8.91	0	(39)	0.0079	1	K.GAILTTMLATR.N
	202	574.3245	1146.6344	1146.6431	-7.52	0	(51)	0.00057	1	K.GAILTTMLATR.N
	204	574.3274	1146.6402	1146.6431	-2.46	0	(36)	0.014	1	K.GAILTTMLATR.N
	205	574.3279	1146.6412	1146.6431	-1.59	0	(49)	0.00074	1	K.GAILTTMLATR.N
	206	574.3290	1146.6434	1146.6431	0.33	0	(24)	0.21	1	K.GAILTTMLATR.N
	207	574.3298	1146.6450	1146.6431	1.73	0	63	2.6e-005	1	K.GAILTTMLATR.N
	216	582.3226	1162.6306	1162.6380	-6.31	0	(28)	0.097	1	K.GAILTTMLATR.N + Oxidation (M)
☑	312	664.3106	1326.6066	1326.5948	8.93	0	38	0.0084	1	R.LTQYMDGSGMPK.T
☑	313	664.3110	1326.6074	1326.5948	9.53	0	(29)	0.071	1	R.LTQYMDGSGMPK.T
☑	476	809.8831	1617.7516	1617.7562	-2.84	0	(7)	11	1	R.FTDEYQLFEELGK.G
☑	477	809.8839	1617.7532	1617.7562	-1.85	0	(36)	0.012	1	R.FTDEYQLFEELGK.G
☑	478	809.8858	1617.7570	1617.7562	0.50	0	48	0.0009	1	R.FTDEYQLFEELGK.G
	646	694.3165	2079.9277	2079.9426	-7.15	0	(51)	0.0003	1	K.AGAYDFPSPEWDTVTPEAK.D
	647	694.3185	2079.9337	2079.9426	-4.27	0	(60)	3.8e-005	1	K.AGAYDFPSPEWDTVTPEAK.D
	648	1040.9807	2079.9468	2079.9426	2.07	0	(60)	4.5e-005	1	K.AGAYDFPSPEWDTVTPEAK.D
	649	1040.9808	2079.9470	2079.9426	2.16	0	(43)	0.0021	1	K.AGAYDFPSPEWDTVTPEAK.D
	650	1040.9817	2079.9488	2079.9426	3.03	0	(53)	0.0002	1	K.AGAYDFPSPEWDTVTPEAK.D
	651	1040.9820	2079.9494	2079.9426	3.32	0	(56)	0.00011	1	K.AGAYDFPSPEWDTVTPEAK.D
	652	1040.9820	2079.9494	2079.9426	3.32	0	(50)	0.00046	1	K.AGAYDFPSPEWDTVTPEAK.D
	653	1040.9828	2079.9510	2079.9426	4.09	0	(45)	0.0013	1	K.AGAYDFPSPEWDTVTPEAK.D
	654	1040.9838	2079.9530	2079.9426	5.05	0	74	1.9e-006	1	K.AGAYDFPSPEWDTVTPEAK.D
	655	1040.9847	2079.9548	2079.9426	5.91	0	(34)	0.019	1	K.AGAYDFPSPEWDTVTPEAK.D
	696	888.7555	2663.2447	2663.2755	-11.57	1	(27)	0.084	1	K.AGAYDFPSPEWDTVTPEAKDLINK.M
	697	888.7577	2663.2513	2663.2755	-9.10	1	(8)	6.2	1	K.AGAYDFPSPEWDTVTPEAKDLINK.M
	698	888.7581	2663.2525	2663.2755	-8.65	1	(5)	14	1	K.AGAYDFPSPEWDTVTPEAKDLINK.M
	699	888.7607	2663.2603	2663.2755	-5.72	1	(22)	0.24	1	K.AGAYDFPSPEWDTVTPEAKDLINK.M
	701	888.7623	2663.2651	2663.2755	-3.91	1	(8)	7.1	1	K.AGAYDFPSPEWDTVTPEAKDLINK.M
	703	888.7635	2663.2687	2663.2755	-2.56	1	37	0.0079	1	K.AGAYDFPSPEWDTVTPEAKDLINK.M

Proteins matching the same set of peptides:
IPI00406790 **Mass:** 60593 **Score:** 284 **Queries matched:** 34
Tax_Id=10090 Gene_Symbol=Camk2d Isoform 4 of Calcium/calmodulin-dependent protein kinase type II subunit delta
IPI00475044 **Mass:** 55899 **Score:** 284 **Queries matched:** 34
Tax_Id=10090 Gene_Symbol=Camk2d Isoform 2 of Calcium/calmodulin-dependent protein kinase type II subunit delta
IPI00828919 **Mass:** 58492 **Score:** 284 **Queries matched:** 34
Tax_Id=10090 Gene_Symbol=Camk2d Isoform 3 of Calcium/calmodulin-dependent protein kinase type II subunit delta
IPI00857865 **Mass:** 54664 **Score:** 284 **Queries matched:** 34
Tax_Id=10090 Gene_Symbol=Camk2d Putative uncharacterized protein
IPI00858128 **Mass:** 58283 **Score:** 284 **Queries matched:** 34
Tax_Id=10090 Gene_Symbol=Camk2d calcium/calmodulin-dependent protein kinase type II subunit delta isoform 1
IPI00858144 **Mass:** 55828 **Score:** 284 **Queries matched:** 34
Tax_Id=10090 Gene_Symbol=Camk2d 55 kDa protein

---

12. IPI00608020 **Mass:** 20817 **Score:** 247 **Queries matched:** 6 **emPAI:** 0.35
Tax_Id=10090 Gene_Symbol=Ftl1 ferritin light chain 1
☐ Check to include this hit in error tolerant search or archive report

	Query	Observed	Mr(expt)	Mr(calc)	ppm	Miss	Score	Expect	Rank	Peptide
☑	125	517.7652	1033.5158	1033.5192	-3.28	0	(39)	0.012	1	R.LLEFQNDR.G
☑	126	517.7660	1033.5174	1033.5192	-1.73	0	(35)	0.033	1	R.LLEFQNDR.G
☑	128	517.7683	1033.5220	1033.5192	2.72	0	46	0.0026	1	R.LLEFQNDR.G
☑	686	791.7253	2372.1541	2372.1761	-9.27	0	(73)	2.1e-006	1	R.VAGPQPAQTGAPQGSLGEYLFER.L
☑	687	791.7254	2372.1544	2372.1761	-9.15	0	110	4.8e-010	1	R.VAGPQPAQTGAPQGSLGEYLFER.L
☑	688	791.7254	2372.1544	2372.1761	-9.15	0	(92)	2.8e-008	1	R.VAGPQPAQTGAPQGSLGEYLFER.L

Proteins matching the same set of peptides:
IPI00625129 **Mass:** 20783 **Score:** 247 **Queries matched:** 6
Tax_Id=10090 Gene_Symbol=Ftl2 ferritin light chain 2
IPI00762203 **Mass:** 20847 **Score:** 247 **Queries matched:** 6
Tax_Id=10090 Gene_Symbol=Ftl1 Ferritin light chain 1

---

13. IPI00122928 **Mass:** 50514 **Score:** 219 **Queries matched:** 12 **emPAI:** 0.21

Tax_Id=10090 Gene_Symbol=Tubb6 Tubulin beta-6 chain

☐ Check to include this hit in error tolerant search or archive report

Query	Observed	Mr(expt)	Mr(calc)	ppm	Miss	Score	Expect	Rank	Peptide
191	572.3217	1142.6288	1142.6270	1.59	0	69	1.1e-005	1	K.LAVNMVPFPR.L
192	572.3229	1142.6312	1142.6270	3.69	0	(38)	0.013	1	K.LAVNMVPFPR.L
330	676.3404	1350.6662	1350.6676	-0.98	0	(65)	2.1e-005	1	R.IMNTFSVMPSPK.V
331	676.3443	1350.6740	1350.6676	4.79	0	82	5.1e-007	1	R.IMNTFSVMPSPK.V
332	676.3445	1350.6744	1350.6676	5.09	0	(64)	3.2e-005	1	R.IMNTFSVMPSPK.V
521	846.4363	1690.8580	1690.8600	-1.16	0	(13)	3.3	2	R.ALTVPELTQQMFDAK.N
522	846.4375	1690.8604	1690.8600	0.26	0	(34)	0.027	1	R.ALTVPELTQQMFDAK.N
523	846.4381	1690.8616	1690.8600	0.97	0	(28)	0.099	1	R.ALTVPELTQQMFDAK.N
524	846.4382	1690.8618	1690.8600	1.09	0	53	0.00034	1	R.ALTVPELTQQMFDAK.N
526	848.9185	1695.8224	1695.8257	-1.89	0	12	3.8	1	K.NSSYFVEWIPNNVK.V
558	854.4335	1706.8524	1706.8549	-1.45	0	(28)	0.11	2	R.ALTVPELTQQMFDAK.N + Oxidation (M)
560	854.4359	1706.8572	1706.8549	1.36	0	(16)	1.5	2	R.ALTVPELTQQMFDAK.N + Oxidation (M)

---

14. IPI00347110 **Mass:** 59502 **Score:** 208 **Queries matched:** 9 **emPAI:** 0.11

Tax_Id=10090 Gene_Symbol=Krt73 Keratin, type II cytoskeletal 73

☐ Check to include this hit in error tolerant search or archive report

Query	Observed	Mr(expt)	Mr(calc)	ppm	Miss	Score	Expect	Rank	Peptide
279	639.3514	1276.6882	1276.7027	-11.29	0	(9)	9.3	1	K.LALDIEIATYR.K
280	639.3543	1276.6940	1276.7027	-6.75	0	(46)	0.0019	1	K.LALDIEIATYR.K
281	639.3561	1276.6976	1276.7027	-3.93	0	(47)	0.0012	1	K.LALDIEIATYR.K
282	639.3570	1276.6994	1276.7027	-2.52	0	54	0.00024	1	K.LALDIEIATYR.K
283	639.3654	1276.7162	1276.7027	10.6	0	(28)	0.071	1	K.LALDIEIATYR.K
395	738.3946	1474.7746	1474.7780	-2.26	0	(68)	9.4e-006	1	R.FLEQQNQVLQTK.W
396	738.3973	1474.7800	1474.7780	1.40	0	83	2.9e-007	1	R.FLEQQNQVLQTK.W
397	738.3979	1474.7812	1474.7780	2.21	0	(66)	1.6e-005	1	R.FLEQQNQVLQTK.W
398	738.3981	1474.7816	1474.7780	2.48	0	(43)	0.0036	1	R.FLEQQNQVLQTK.W

---

15. IPI00122048 **Mass:** 113045 **Score:** 198 **Queries matched:** 8 **emPAI:** 0.12

Tax_Id=10090 Gene_Symbol=Atp1a3 Sodium/potassium-transporting ATPase subunit alpha-3

☐ Check to include this hit in error tolerant search or archive report

Query	Observed	Mr(expt)	Mr(calc)	ppm	Miss	Score	Expect	Rank	Peptide
246	618.8546	1235.6946	1235.6986	-3.20	0	(54)	0.00019	1	R.LNIPVSQVNPR.D
247	618.8594	1235.7042	1235.6986	4.56	0	66	9.5e-006	1	R.LNIPVSQVNPR.D
277	634.8717	1267.7288	1267.7071	17.2	0	33	0.021	1	K.NMVPQQALVIR.E
595	906.4749	1810.9352	1810.9425	-4.01	0	60	6.4e-005	1	R.QGAIVAVTGDGVNDSPALK.K
596	906.4772	1810.9398	1810.9425	-1.47	0	(56)	0.00013	1	R.QGAIVAVTGDGVNDSPALK.K
600	915.4284	1828.8422	1828.9167	-40.68	0	(6)	12	7	K.GVGIISEGNETVEDIAAR.L
603	915.4561	1828.8976	1828.9167	-10.39	0	(52)	0.00038	1	K.GVGIISEGNETVEDIAAR.L
604	915.4638	1828.9130	1828.9167	-1.97	0	70	6.2e-006	1	K.GVGIISEGNETVEDIAAR.L

Proteins matching the same set of peptides:

IPI00420569 **Mass:** 113457 **Score:** 198 **Queries matched:** 8
Tax_Id=10090 Gene_Symbol=Atp1a2 Sodium/potassium-transporting ATPase subunit alpha-2

IPI00752412 **Mass:** 117548 **Score:** 198 **Queries matched:** 8
Tax_Id=10090 Gene_Symbol=Atp1a3 ATPase, Na+/K+ transporting, alpha 3 polypeptide, isoform CRA_c

IPI00762871 **Mass:** 104717 **Score:** 198 **Queries matched:** 8
Tax_Id=10090 Gene_Symbol=Atp1a2 Uncharacterized protein

---

16. IPI00138892 **Mass:** 15004 **Score:** 187 **Queries matched:** 5 **emPAI:** 0.23

Tax_Id=10090 Gene_Symbol=Uba52 Ubiquitin-60S ribosomal protein L40

☐ Check to include this hit in error tolerant search or archive report

Query	Observed	Mr(expt)	Mr(calc)	ppm	Miss	Score	Expect	Rank	Peptide
578	894.4645	1786.9144	1786.9200	-3.11	0	(9)	8.9	1	K.TITLEVEPSDTIENVK.A
579	894.4648	1786.9150	1786.9200	-2.78	0	(62)	4.5e-005	1	K.TITLEVEPSDTIENVK.A
580	894.4680	1786.9214	1786.9200	0.80	0	(67)	1.2e-005	1	K.TITLEVEPSDTIENVK.A
581	894.4692	1786.9238	1786.9200	2.15	0	(77)	1.4e-006	1	K.TITLEVEPSDTIENVK.A
582	894.4696	1786.9246	1786.9200	2.59	0	80	6e-007	1	K.TITLEVEPSDTIENVK.A

Proteins matching the same set of peptides:

IPI00470152 **Mass:** 18282 **Score:** 187 **Queries matched:** 5
Tax_Id=10090 Gene_Symbol=Rps27a Ubiquitin-40S ribosomal protein S27a

IPI00895319 **Mass:** 22277 **Score:** 187 **Queries matched:** 5
Tax_Id=10090 Gene_Symbol=2810422J05Rik 22 kDa protein

IPI00895479 **Mass:** 27181 **Score:** 187 **Queries matched:** 5
Tax_Id=10090 Gene_Symbol=2810422J05Rik 27 kDa protein

IPI00918763 **Mass:** 11021 **Score:** 187 **Queries matched:** 5
Tax_Id=10090 Gene_Symbol=Uba52 Ubiqutin subunit 1

IPI00750889 **Mass:** 22822 **Score:** 187 **Queries matched:** 5
Tax_Id=10090 Gene_Symbol=Ubc Ubc protein

IPI00923013 **Mass:** 26603 **Score:** 187 **Queries matched:** 5
Tax_Id=10090 Gene_Symbol=Ubb Ubiquitin B

IPI00923037 **Mass:** 22578 **Score:** 187 **Queries matched:** 5
Tax_Id=10090 Gene_Symbol=Ubc Putative uncharacterized protein

IPI00139518 **Mass:** 34348 **Score:** 187 **Queries matched:** 5
Tax_Id=10090 Gene_Symbol=Ubb Polyubiquitin-B

IPI00755916 **Mass:** 82614 **Score:** 187 **Queries matched:** 5

Tax_Id=10090 Gene_Symbol=Ubc ubiquitin
IPI00969323 **Mass:** 82603 **Score:** 187 **Queries matched:** 5
Tax_Id=10090 Gene_Symbol=Ubc Polyubiquitin-C

---

17. IPI00625729 **Mass:** 66079 **Score:** 175 **Queries matched:** 5 **emPAI:** 0.10
Tax_Id=10090 Gene_Symbol=Krt1 Keratin, type II cytoskeletal 1
☐ Check to include this hit in error tolerant search or archive report

	Query	Observed	Mr(expt)	Mr(calc)	ppm	Miss	Score	Expect	Rank	Peptide
☑	273	633.3264	1264.6382	1264.6299	6.59	0	44	0.003	1	R.TNAENEFVTIK.K
	395	738.3946	1474.7746	1474.7780	-2.26	0	(68)	9.4e-006	1	R.FLEQQNQVLQTK.W
	396	738.3973	1474.7800	1474.7780	1.40	0	83	2.9e-007	1	R.FLEQQNQVLQTK.W
	397	738.3979	1474.7812	1474.7780	2.21	0	(66)	1.6e-005	1	R.FLEQQNQVLQTK.W
	398	738.3981	1474.7816	1474.7780	2.48	0	(43)	0.0036	1	R.FLEQQNQVLQTK.W

---

18. IPI00136703 **Mass:** 42971 **Score:** 160 **Queries matched:** 5 **emPAI:** 0.08
Tax_Id=10090 Gene_Symbol=Ckb Creatine kinase B-type
☐ Check to include this hit in error tolerant search or archive report

	Query	Observed	Mr(expt)	Mr(calc)	ppm	Miss	Score	Expect	Rank	Peptide
☑	302	652.3596	1302.7046	1302.7183	-10.50	0	(72)	4.6e-006	1	K.VLTPELYAELR.A
☑	303	652.3610	1302.7074	1302.7183	-8.35	0	(58)	0.00011	1	K.VLTPELYAELR.A
☑	304	652.3613	1302.7080	1302.7183	-7.89	0	90	6e-008	1	K.VLTPELYAELR.A
☑	462	801.9241	1601.8336	1601.8260	4.74	0	20	0.73	1	K.LAVEALSSLDGDLSGR.Y
☑	517	828.9160	1655.8174	1655.8188	-0.85	0	3	29	1	R.LEQGQAIDDLMPAQK.-

---

19. IPI00110850 **Mass:** 42052 **Score:** 157 **Queries matched:** 11 **emPAI:** 0.35
Tax_Id=10090 Gene_Symbol=Actb Actin, cytoplasmic 1
☐ Check to include this hit in error tolerant search or archive report

	Query	Observed	Mr(expt)	Mr(calc)	ppm	Miss	Score	Expect	Rank	Peptide
☑	213	581.3192	1160.6238	1160.6111	11.0	0	43	0.004	1	K.EITALAPSTMK.I
☑	214	581.3192	1160.6238	1160.6111	11.0	0	(22)	0.51	1	K.EITALAPSTMK.I
☑	227	599.7656	1197.5166	1197.5150	1.41	0	46	0.00079	1	K.DSYVGDEAQSK.R
☑	583	895.9476	1789.8806	1789.8846	-2.22	0	(40)	0.0058	1	K.SYELPDGQVITIGNER.F
☑	584	895.9503	1789.8860	1789.8846	0.79	0	(55)	0.00017	1	K.SYELPDGQVITIGNER.F
☑	585	895.9514	1789.8882	1789.8846	2.02	0	93	2.7e-008	1	K.SYELPDGQVITIGNER.F
	613	652.0187	1953.0343	1953.0571	-11.69	0	(15)	1.3	2	R.VAPEEHPVLLTEAPLNPK.A
	614	652.0188	1953.0346	1953.0571	-11.53	0	31	0.034	2	R.VAPEEHPVLLTEAPLNPK.A
☑	615	652.0193	1953.0361	1953.0571	-10.77	0	(21)	0.39	1	R.VAPEEHPVLLTEAPLNPK.A
☑	616	652.0218	1953.0436	1953.0571	-6.93	0	(7)	8.3	1	R.VAPEEHPVLLTEAPLNPK.A
☑	618	652.0227	1953.0463	1953.0571	-5.54	0	(19)	0.5	1	R.VAPEEHPVLLTEAPLNPK.A

Proteins matching the same set of peptides:
IPI00136929 **Mass:** 44029 **Score:** 157 **Queries matched:** 11
Tax_Id=10090 Gene_Symbol=Actg1 Gamma actin-like protein
IPI00473320 **Mass:** 42115 **Score:** 157 **Queries matched:** 11
Tax_Id=10090 Gene_Symbol=Actb Putative uncharacterized protein
IPI00652436 **Mass:** 42053 **Score:** 157 **Queries matched:** 11
Tax_Id=10090 Gene_Symbol=Actg1 Putative uncharacterized protein
IPI00874482 **Mass:** 42108 **Score:** 157 **Queries matched:** 11
Tax_Id=10090 Gene_Symbol=Actg1 Actin, cytoplasmic 2

---

20. IPI00221797 **Mass:** 59932 **Score:** 155 **Queries matched:** 7 **emPAI:** 0.05
Tax_Id=10090 Gene_Symbol=Krt75 Keratin, type II cytoskeletal 75
☐ Check to include this hit in error tolerant search or archive report

	Query	Observed	Mr(expt)	Mr(calc)	ppm	Miss	Score	Expect	Rank	Peptide
☑	288	651.8494	1301.6842	1301.7078	-18.11	0	(7)	15	1	R.SLDLDSIIAEVK.A
☑	289	651.8604	1301.7062	1301.7078	-1.21	0	(30)	0.062	1	R.SLDLDSIIAEVK.A
☑	290	651.8617	1301.7088	1301.7078	0.79	0	(61)	4.8e-005	1	R.SLDLDSIIAEVK.A
☑	291	651.8624	1301.7102	1301.7078	1.86	0	(59)	7.4e-005	1	R.SLDLDSIIAEVK.A
☑	292	651.8624	1301.7102	1301.7078	1.86	0	(63)	3.1e-005	1	R.SLDLDSIIAEVK.A
☑	293	651.8641	1301.7136	1301.7078	4.47	0	63	2.9e-005	1	R.SLDLDSIIAEVK.A
☑	399	738.8942	1475.7738	1475.7984	-16.62	1	4	26	1	R.FLEQQNKVLETK.W

---

21. IPI00348328 **Mass:** 114018 **Score:** 142 **Queries matched:** 6 **emPAI:** 0.03
Tax_Id=10090 Gene_Symbol=Krt78 keratin Kb40
☐ Check to include this hit in error tolerant search or archive report

	Query	Observed	Mr(expt)	Mr(calc)	ppm	Miss	Score	Expect	Rank	Peptide
☑	114	504.7170	1007.4194	1007.4528	-33.13	0	5	7.8	1	K.QDMAQMLR.D + Oxidation (M)
☑	345	692.3474	1382.6802	1382.6830	-2.00	0	(60)	6.9e-005	1	R.SLNNQFASFIDK.V
☑	346	692.3569	1382.6992	1382.6830	11.7	0	(49)	0.00093	1	R.SLNNQFASFIDK.V
☑	347	692.3570	1382.6994	1382.6830	11.9	0	61	6.2e-005	1	R.SLNNQFASFIDK.V
☑	348	692.3643	1382.7140	1382.6830	22.4	0	(49)	0.00089	1	R.SLNNQFASFIDK.V
	399	738.8942	1475.7738	1475.7984	-16.62	1	4	26	1	R.FLEQQNKVLETK.W

---

22. IPI00322209 **Mass:** 54531 **Score:** 142 **Queries matched:** 4 **emPAI:** 0.06
Tax_Id=10090 Gene_Symbol=Krt8 Keratin, type II cytoskeletal 8
☐ Check to include this hit in error tolerant search or archive report

Query	Observed	Mr(expt)	Mr(calc)	ppm	Miss	Score	Expect	Rank	Peptide
345	692.3474	1382.6802	1382.7194	-28.31	1	(60)	6.9e-005	1	K.SLNNKFASFIDK.V
346	692.3569	1382.6992	1382.7194	-14.57	1	(49)	0.00093	1	K.SLNNKFASFIDK.V
347	692.3570	1382.6994	1382.7194	-14.42	1	61	6.2e-005	1	K.SLNNKFASFIDK.V
348	692.3643	1382.7140	1382.7194	-3.86	1	(49)	0.00089	1	K.SLNNKFASFIDK.V

---

23. IPI00221528 **Mass:** 42319 **Score:** 126 **Queries matched:** 8 **emPAI:** 0.16
Tax_Id=10090 Gene_Symbol=Actbl2 Beta-actin-like protein 2
☐ Check to include this hit in error tolerant search or archive report

	Query	Observed	Mr(expt)	Mr(calc)	ppm	Miss	Score	Expect	Rank	Peptide
	583	895.9476	1789.8806	1789.8846	-2.22	0	(40)	0.0058	1	R.SYELPDGQVITIGNER.F
	584	895.9503	1789.8860	1789.8846	0.79	0	(55)	0.00017	1	R.SYELPDGQVITIGNER.F
	585	895.9514	1789.8882	1789.8846	2.02	0	93	2.7e-008	1	R.SYELPDGQVITIGNER.F
☑	613	652.0187	1953.0343	1953.0571	-11.69	0	(16)	1.2	1	R.VAPDEHPILLTEAPLNPK.I
☑	614	652.0188	1953.0346	1953.0571	-11.53	0	32	0.03	1	R.VAPDEHPILLTEAPLNPK.I
	615	652.0193	1953.0361	1953.0571	-10.77	0	(13)	2.3	2	R.VAPDEHPILLTEAPLNPK.I
	616	652.0218	1953.0436	1953.0571	-6.93	0	(5)	15	3	R.VAPDEHPILLTEAPLNPK.I
	618	652.0227	1953.0463	1953.0571	-5.54	0	(16)	1.1	2	R.VAPDEHPILLTEAPLNPK.I

---

24. IPI00124499 **Mass:** 57802 **Score:** 105 **Queries matched:** 5 **emPAI:** 0.06
Tax_Id=10090 Gene_Symbol=Krt79 Keratin, type II cytoskeletal 79
☐ Check to include this hit in error tolerant search or archive report

	Query	Observed	Mr(expt)	Mr(calc)	ppm	Miss	Score	Expect	Rank	Peptide
☑	314	665.3632	1328.7118	1328.7187	-5.17	0	(34)	0.025	1	R.NLDLDSIIAEVK.A
☑	315	665.3666	1328.7186	1328.7187	-0.05	0	73	2.9e-006	1	R.NLDLDSIIAEVK.A
☑	316	665.3679	1328.7212	1328.7187	1.90	0	(41)	0.0055	1	R.NLDLDSIIAEVK.A
☑	317	665.3680	1328.7214	1328.7187	2.05	0	(42)	0.0038	1	R.NLDLDSIIAEVK.A
	399	738.8942	1475.7738	1475.7984	-16.62	1	4	26	1	R.FLEQQNKVLETK.W

---

25. IPI00230145 **Mass:** 21224 **Score:** 88 **Queries matched:** 3 **emPAI:** 0.16
Tax_Id=10090 Gene_Symbol=Fth1 Ferritin heavy chain
☐ Check to include this hit in error tolerant search or archive report

	Query	Observed	Mr(expt)	Mr(calc)	ppm	Miss	Score	Expect	Rank	Peptide
	79	438.7601	875.5056	875.5116	-6.85	0	7	12	5	R.IFLQDIK.K
☑	487	815.3727	1628.7308	1628.7214	5.78	0	(45)	0.0012	1	K.MGAPEAGMAEYLFDK.H
☑	488	815.3737	1628.7328	1628.7214	7.01	0	75	1.3e-006	1	K.MGAPEAGMAEYLFDK.H

---

26. IPI00406377 **Mass:** 50678 **Score:** 82 **Queries matched:** 6 **emPAI:** 0.06
Tax_Id=10090 Gene_Symbol=Krt7 Keratin, type II cytoskeletal 7
☐ Check to include this hit in error tolerant search or archive report

Query	Observed	Mr(expt)	Mr(calc)	ppm	Miss	Score	Expect	Rank	Peptide
104	487.2706	972.5266	972.5352	-8.81	1	10	9.1	8	K.SAKSSQLPR.I
279	639.3514	1276.6882	1276.7027	-11.29	0	(9)	9.3	1	K.LALDIEIATYR.K
280	639.3543	1276.6940	1276.7027	-6.75	0	(46)	0.0019	1	K.LALDIEIATYR.K
281	639.3561	1276.6976	1276.7027	-3.93	0	(47)	0.0012	1	K.LALDIEIATYR.K
282	639.3570	1276.6994	1276.7027	-2.52	0	54	0.00024	1	K.LALDIEIATYR.K
283	639.3654	1276.7162	1276.7027	10.6	0	(28)	0.071	1	K.LALDIEIATYR.K

---

27. IPI00222228 **Mass:** 58587 **Score:** 82 **Queries matched:** 6 **emPAI:** 0.06
Tax_Id=10090 Gene_Symbol=4732456N10Rik hypothetical protein LOC239673
☐ Check to include this hit in error tolerant search or archive report

Query	Observed	Mr(expt)	Mr(calc)	ppm	Miss	Score	Expect	Rank	Peptide
279	639.3514	1276.6882	1276.7027	-11.29	0	(9)	9.3	1	K.LALDIEIATYR.K
280	639.3543	1276.6940	1276.7027	-6.75	0	(46)	0.0019	1	K.LALDIEIATYR.K
281	639.3561	1276.6976	1276.7027	-3.93	0	(47)	0.0012	1	K.LALDIEIATYR.K
282	639.3570	1276.6994	1276.7027	-2.52	0	54	0.00024	1	K.LALDIEIATYR.K
283	639.3654	1276.7162	1276.7027	10.6	0	(28)	0.071	1	K.LALDIEIATYR.K
399	738.8942	1475.7738	1475.7984	-16.62	1	4	26	1	R.FLEQQNKVLETK.W

---

28. IPI00135284 **Mass:** 36092 **Score:** 78 **Queries matched:** 7 **emPAI:** 0.19
Tax_Id=10090 Gene_Symbol=Gm16374 Glyceraldehyde-3-phosphate dehydrogenase
☐ Check to include this hit in error tolerant search or archive report

	Query	Observed	Mr(expt)	Mr(calc)	ppm	Miss	Score	Expect	Rank	Peptide
☑	333	678.8232	1355.6318	1355.6465	-10.80	1	(21)	0.41	1	R.VVDLMAYMASKE.-
☑	334	678.8466	1355.6786	1355.6465	23.7	1	43	0.0032	1	R.VVDLMAYMASKE.-
☑	335	685.3756	1368.7366	1368.7361	0.40	0	(50)	0.00057	1	R.GAAQNIIPASTGAAK.A
☑	336	685.3767	1368.7388	1368.7361	2.01	0	(39)	0.0079	1	R.GAAQNIIPASTGAAK.A
☑	337	685.3770	1368.7394	1368.7361	2.45	0	(20)	0.53	1	R.GAAQNIIPASTGAAK.A
☑	338	685.3795	1368.7444	1368.7361	6.10	0	58	9.3e-005	1	R.GAAQNIIPASTGAAK.A
☑	573	890.4178	1778.8210	1778.7900	17.5	0	25	0.17	1	K.LISWYDNEYGYSNR.V

Proteins matching the same set of peptides:
IPI00271869 **Mass:** 36293 **Score:** 78 **Queries matched:** 7
Tax_Id=10090 Gene_Symbol=- 36 kDa protein

IPI00273646 **Mass:** 36072 **Score:** 78 **Queries matched:** 7
Tax_Id=10090 Gene_Symbol=Gapdh;LOC100042025 Glyceraldehyde-3-phosphate dehydrogenase
IPI00622795 **Mass:** 36074 **Score:** 78 **Queries matched:** 7
Tax_Id=10090 Gene_Symbol=- 36 kDa protein
IPI00752289 **Mass:** 36308 **Score:** 78 **Queries matched:** 7
Tax_Id=10090 Gene_Symbol=Gm10290 Glyceraldehyde-3-phosphate dehydrogenase
IPI00848801 **Mass:** 36100 **Score:** 78 **Queries matched:** 7
Tax_Id=10090 Gene_Symbol=- 36 kDa protein
IPI00849045 **Mass:** 36072 **Score:** 78 **Queries matched:** 7
Tax_Id=10090 Gene_Symbol=- 36 kDa protein
IPI00850337 **Mass:** 44633 **Score:** 78 **Queries matched:** 7
Tax_Id=10090 Gene_Symbol=LOC100048117 similar to Glyceraldehyde-3-phosphate dehydrogenase (GAPDH) isoform 1
IPI00850377 **Mass:** 35975 **Score:** 78 **Queries matched:** 7
Tax_Id=10090 Gene_Symbol=- 36 kDa protein
IPI00850779 **Mass:** 36076 **Score:** 78 **Queries matched:** 7
Tax_Id=10090 Gene_Symbol=- 36 kDa protein
IPI00874682 **Mass:** 36038 **Score:** 78 **Queries matched:** 7
Tax_Id=10090 Gene_Symbol=- 36 kDa protein
IPI00874964 **Mass:** 36512 **Score:** 78 **Queries matched:** 7
Tax_Id=10090 Gene_Symbol=- 36 kDa protein

---

29. IPI00134997 **Mass:** 106777 **Score:** 78 **Queries matched:** 1 **emPAI:** 0.03
Tax_Id=10090 Gene_Symbol=Dlgap3 Disks large-associated protein 3
☐ Check to include this hit in error tolerant search or archive report

	Query	Observed	Mr(expt)	Mr(calc)	ppm	Miss	Score	Expect	Rank	Peptide
☑	405	**751.8646**	**1501.7146**	**1501.7274**	**-8.46**	**0**	**78**	**1e-006**	**1**	**R.GPAGPGPGPGSGAAPEAR.S**

Proteins matching the same set of peptides:
IPI00648401 **Mass:** 105576 **Score:** 78 **Queries matched:** 1
Tax_Id=10090 Gene_Symbol=Dlgap3 Discs, large (Drosophila) homolog-associated protein 3

---

30. IPI00348094 **Mass:** 51093 **Score:** 75 **Queries matched:** 6 **emPAI:** 0.06
Tax_Id=10090 Gene_Symbol=Tubb1 Tubulin, beta 1
☐ Check to include this hit in error tolerant search or archive report

Query	Observed	Mr(expt)	Mr(calc)	ppm	Miss	Score	Expect	Rank	Peptide
191	572.3217	1142.6288	1142.6270	1.59	0	69	1.1e-005	1	K.LAVNMVPFPR.L
192	572.3229	1142.6312	1142.6270	3.69	0	(38)	0.013	1	K.LAVNMVPFPR.L
308	660.3503	1318.6860	1318.6955	-7.16	0	(11)	5.8	2	R.ILNSFSVMPSPK.V
309	660.3552	1318.6958	1318.6955	0.27	0	(13)	4.1	2	R.ILNSFSVMPSPK.V
310	660.3563	1318.6980	1318.6955	1.94	0	21	0.63	2	R.ILNSFSVMPSPK.V
311	660.3572	1318.6998	1318.6955	3.30	0	(16)	1.9	2	R.ILNSFSVMPSPK.V

---

31. IPI00115546 **Mass:** 40524 **Score:** 70 **Queries matched:** 5 **emPAI:** 0.17
Tax_Id=10090 Gene_Symbol=Gnao1 Isoform Alpha-2 of Guanine nucleotide-binding protein G(o) subunit alpha
☐ Check to include this hit in error tolerant search or archive report

	Query	Observed	Mr(expt)	Mr(calc)	ppm	Miss	Score	Expect	Rank	Peptide
☑	150	**529.3204**	**1056.6262**	**1056.6179**	**7.91**	**0**	**45**	**0.0019**	**1**	**K.LLLLGAGESGK.S**
☑	520	**838.4207**	**1674.8268**	**1674.8213**	**3.31**	**0**	**55**	**0.00023**	**1**	**R.IGAGDYQPTEQDILR.T**
☑	621	**979.4400**	**1956.8654**	**1956.8631**	**1.21**	**0**	**(7)**	**5.7**	**1**	**R.MEDTEPFSAELLSAMMR.L**
☑	622	**979.4463**	**1956.8780**	**1956.8631**	**7.65**	**0**	**(5)**	**11**	**1**	**R.MEDTEPFSAELLSAMMR.L**
☑	623	**979.4526**	**1956.8906**	**1956.8631**	**14.1**	**0**	**10**	**4.2**	**1**	**R.MEDTEPFSAELLSAMMR.L**

Proteins matching the same set of peptides:
IPI00230192 **Mass:** 40629 **Score:** 70 **Queries matched:** 5
Tax_Id=10090 Gene_Symbol=Gnao1 Isoform Alpha-1 of Guanine nucleotide-binding protein G(o) subunit alpha

---

32. IPI00122094 **Mass:** 85656 **Score:** 65 **Queries matched:** 3 **emPAI:** 0.08
Tax_Id=10090 Gene_Symbol=Dlg4 Isoform 2 of Disks large homolog 4
☐ Check to include this hit in error tolerant search or archive report

	Query	Observed	Mr(expt)	Mr(calc)	ppm	Miss	Score	Expect	Rank	Peptide
☑	185	**563.2996**	**1124.5846**	**1124.5938**	**-8.15**	**0**	**46**	**0.0019**	**1**	**K.IIPGGAAAQDGR.L**
☑	561	**857.9614**	**1713.9082**	**1713.9090**	**-0.44**	**0**	**42**	**0.0032**	**1**	**R.VIEDLSGPYIWVPAR.E**
☑	562	**857.9666**	**1713.9186**	**1713.9090**	**5.63**	**0**	**(26)**	**0.13**	**1**	**R.VIEDLSGPYIWVPAR.E**

Proteins matching the same set of peptides:
IPI00622720 **Mass:** 80765 **Score:** 65 **Queries matched:** 3
Tax_Id=10090 Gene_Symbol=Dlg4 Isoform 1 of Disks large homolog 4
IPI00626797 **Mass:** 80395 **Score:** 65 **Queries matched:** 3
Tax_Id=10090 Gene_Symbol=Dlg4 Isoform 3 of Disks large homolog 4

---

33. IPI00119667 **Mass:** 50764 **Score:** 53 **Queries matched:** 1 **emPAI:** 0.06
Tax_Id=10090 Gene_Symbol=Eef1a2 Elongation factor 1-alpha 2
☐ Check to include this hit in error tolerant search or archive report

	Query	Observed	Mr(expt)	Mr(calc)	ppm	Miss	Score	Expect	Rank	Peptide
☑	116	**513.3045**	**1024.5944**	**1024.6030**	**-8.30**	**0**	**53**	**0.00015**	**1**	**K.IGGIGTVPVGR.V**

Proteins matching the same set of peptides:
IPI00307837 **Mass:** 50424 **Score:** 53 **Queries matched:** 1

Tax_Id=10090 Gene_Symbol=Eef1a1 Elongation factor 1-alpha 1

---

34. IPI00135197 **Score:** 53 **Queries matched:** 2
Tax_Id=10090 Gene_Symbol=Krt20 Keratin, type I cytoskeletal 20
☐ Check to include this hit in error tolerant search or archive report

Query	Observed	Mr(expt)	Mr(calc)	ppm	Miss	Score	Expect	Rank	Peptide
170	545.7681	1089.5216	1089.5237	-1.85	0	47	0.0016	2	K.LAMQNLNDR.L + Oxidation (M)
171	545.7692	1089.5238	1089.5237	0.17	0	(45)	0.0024	2	K.LAMQNLNDR.L + Oxidation (M)

---

35. IPI00467833 **Mass:** 32684 **Score:** 45 **Queries matched:** 1 **emPAI:** 0.10
Tax_Id=10090 Gene_Symbol=Tpi1 triosephosphate isomerase
☐ Check to include this hit in error tolerant search or archive report

Query	Observed	Mr(expt)	Mr(calc)	ppm	Miss	Score	Expect	Rank	Peptide
249	**624.2955**	**1246.5764**	**1246.5902**	**-11.03**	**0**	**45**	**0.0017**	**1**	**K.SNVNDGVAQSTR.I**

---

36. IPI00118569 **Mass:** 44369 **Score:** 45 **Queries matched:** 1 **emPAI:** 0.07
Tax_Id=10090 Gene_Symbol=Gna13 Guanine nucleotide-binding protein subunit alpha-13
☐ Check to include this hit in error tolerant search or archive report

Query	Observed	Mr(expt)	Mr(calc)	ppm	Miss	Score	Expect	Rank	Peptide
150	529.3204	1056.6262	1056.6179	7.91	0	45	0.0019	1	K.ILLLGAGESGK.S

Proteins matching the same set of peptides:
IPI00230191 **Mass:** 44238 **Score:** 45 **Queries matched:** 1
Tax_Id=10090 Gene_Symbol=Gna12 Guanine nucleotide-binding protein subunit alpha-12
IPI00649388 **Mass:** 20095 **Score:** 45 **Queries matched:** 1
Tax_Id=10090 Gene_Symbol=Gna13 Putative uncharacterized protein

---

37. IPI00462072 **Mass:** 47453 **Score:** 44 **Queries matched:** 1 **emPAI:** 0.07
Tax_Id=10090 Gene_Symbol=Eno1;Gm5506 Alpha-enolase
☐ Check to include this hit in error tolerant search or archive report

Query	Observed	Mr(expt)	Mr(calc)	ppm	Miss	Score	Expect	Rank	Peptide
362	**720.3760**	**1438.7374**	**1438.7344**	**2.14**	**0**	**44**	**0.0025**	**1**	**R.YITPDQLADLYK.S**

Proteins matching the same set of peptides:
IPI00648821 **Mass:** 40099 **Score:** 44 **Queries matched:** 1
Tax_Id=10090 Gene_Symbol=Eno1 Enolase

---

38. IPI00120793 **Mass:** 28131 **Score:** 41 **Queries matched:** 2 **emPAI:** 0.12
Tax_Id=10090 Gene_Symbol=Prnp Major prion protein
☐ Check to include this hit in error tolerant search or archive report

Query	Observed	Mr(expt)	Mr(calc)	ppm	Miss	Score	Expect	Rank	Peptide
166	**544.7459**	**1087.4772**	**1087.4570**	**18.6**	**0**	**8**	**5.6**	**1**	**K.ESQAYYDGR.R**
189	**570.2626**	**1138.5106**	**1138.5142**	**-3.15**	**0**	**41**	**0.0039**	**1**	**K.GENFTETDVK.M**

---

39. IPI00113410 **Mass:** 38586 **Score:** 40 **Queries matched:** 2 **emPAI:** 0.09
Tax_Id=10090 Gene_Symbol=Hapln2 Hyaluronan and proteoglycan link protein 2
☐ Check to include this hit in error tolerant search or archive report

Query	Observed	Mr(expt)	Mr(calc)	ppm	Miss	Score	Expect	Rank	Peptide
354	**696.3665**	**1390.7184**	**1390.7245**	**-4.35**	**0**	**40**	**0.0065**	**1**	**R.LEGVVFPYQPSR.G**
355	**696.3699**	**1390.7252**	**1390.7245**	**0.54**	**0**	**(29)**	**0.092**	**1**	**R.LEGVVFPYQPSR.G**

---

40. IPI00622240 **Mass:** 71336 **Score:** 39 **Queries matched:** 5 **emPAI:** 0.05
Tax_Id=10090 Gene_Symbol=Krt2 Keratin, type II cytoskeletal 2 epidermal
☐ Check to include this hit in error tolerant search or archive report

Query	Observed	Mr(expt)	Mr(calc)	ppm	Miss	Score	Expect	Rank	Peptide
182	**556.7426**	**1111.4706**	**1111.4643**	**5.72**	**0**	**0**	**24**	**9**	**R.GGSGGGYGSGGGSR.G**
222	**590.3031**	**1178.5916**	**1178.6183**	**-22.60**	**0**	**(23)**	**0.42**	**1**	**K.YEELQVTAVK.H**
223	**590.3059**	**1178.5972**	**1178.6183**	**-17.85**	**0**	**(32)**	**0.054**	**1**	**K.YEELQVTAVK.H**
224	**590.3073**	**1178.6000**	**1178.6183**	**-15.47**	**0**	**35**	**0.025**	**1**	**K.YEELQVTAVK.H**
251	**627.8073**	**1253.6000**	**1253.6001**	**-0.00**	**0**	**39**	**0.01**	**1**	**R.GFSSGSAVVSGGSR.R**

Proteins matching the same set of peptides:
IPI00875096 **Mass:** 71035 **Score:** 39 **Queries matched:** 5
Tax_Id=10090 Gene_Symbol=Krt2 71 kDa protein

Peptide matches not assigned to protein hits: (no details means no match)

Query	Observed	Mr(expt)	Mr(calc)	ppm	Miss	Score	Expect	Rank	Peptide
250	**626.3681**	**1250.7216**	**1250.7347**	**-10.42**	**0**	**36**	**0.0076**	**1**	**GVAGAPPTVTLLR**
305	**656.3151**	**1310.6156**	**1310.5999**	**12.0**	**0**	**35**	**0.018**	**1**	**QMYMSLPQGEK**
132	**520.2753**	**1038.5360**	**1038.5458**	**-9.37**	**0**	**34**	**0.028**	**1**	**AQASAPAQAPK**
229	**614.8425**	**1227.6704**	**1227.6711**	**-0.50**	**0**	**33**	**0.033**	**1**	**TSGPPVSELITK**
136	**523.2849**	**1044.5552**	**1044.5564**	**-1.06**	**0**	**30**	**0.094**	**1**	**INDITLNSR**
208	**578.8143**	**1155.6140**	**1155.6135**	**0.45**	**0**	**30**	**0.073**	**1**	**SLPNEEIVQK**

104	487.2706	972.5266	972.5240	2.74	0	29	0.12	1	EIISEVQR
159	533.2659	1064.5172	1064.5138	3.23	0	28	0.11	1	AQYEDIAQK
133	520.2761	1038.5376	1038.5458	-7.83	0	28	0.11	1	AQASAPAQAPK
230	614.8445	1227.6744	1227.6711	2.75	0	25	0.23	1	TSGPPVSELITK
252	628.8171	1255.6196	1255.6157	3.16	0	25	0.23	1	GGSQGEAVGELPR
71	435.7725	869.5304	869.4971	38.4	0	25	0.14	1	AVVESLPR
231	614.8458	1227.6770	1227.6711	4.87	0	25	0.24	1	TSGPPVSELITK
160	533.2675	1064.5204	1064.5138	6.24	0	21	0.6	1	AQYEDIAQK
134	523.2794	1044.5442	1044.5564	-11.59	0	20	0.87	1	INDITLNSR
237	616.7883	1231.5620	1231.5680	-4.86	0	20	0.57	1	AVIESDEEQGR
105	487.2718	972.5290	972.5240	5.20	0	19	1.1	1	EIISEVQR
84	449.2162	896.4178	896.4214	-4.02	0	19	0.62	1	MWIEFR + Oxidation (M)
66	428.7654	855.5162	855.5542	-44.32	0	19	0.62	1	TLAVLALR
358	698.8622	1395.7098	1395.6630	33.6	0	19	0.92	1	ALGTSDPEGAEPPR
165	543.3147	1084.6148	1084.5811	31.1	1	18	0.99	1	EAIRLAPCR
112	496.2298	990.4450	990.4883	-43.65	0	17	0.94	1	AFSSHSSLR
357	698.8580	1395.7014	1395.6630	27.5	0	17	1.2	1	ALGTSDPEGAEPPR
437	785.8483	1569.6820	1569.7531	-45.25	0	17	0.57	1	SLMQCAGESISLFK
479	810.3789	1618.7432	1618.7331	6.26	0	17	1	1	SSSMVLGSFGTDLMR + 2 Oxidation (M)
56	412.7523	823.4900	823.4817	10.2	0	17	0.53	1	RPWLPR
360	472.9068	1415.6986	1415.7521	-37.80	1	17	1.6	1	ELARWIGVTESR
13	475.2986	474.2913	474.2802	23.5	0	17	2.5	1	LGGTK
684	786.0964	2355.2674	2355.2012	28.1	0	17	0.76	1	QALQFAFYVIEQVGTQPFNR
89	450.7500	899.4854	899.5076	-24.63	1	16	1.9	1	GAPKESALK
212	581.2999	1160.5852	1160.5543	26.7	1	16	2.6	1	AAPGRMCSVGR
624	656.6460	1966.9162	1966.9669	-25.81	1	16	1.3	1	ALVAMNESLKSQEQEFK + Oxidation (M)
370	730.9049	1459.7952	1459.7453	34.2	1	15	1.6	1	VDILENRVMDTR
210	579.3053	1156.5960	1156.6353	-33.92	0	15	2.6	1	VVSWNINGIR
158	533.2621	1064.5096	1064.5138	-3.91	0	15	2.5	1	AQYEDIAQK
41	327.7021	653.3896	653.3609	44.0	0	14	0.98	1	ALGTHR
113	499.2547	996.4948	996.5352	-40.53	0	14	2.5	1	IGTQPPAASR
172	546.7492	1091.4838	1091.5281	-40.52	0	14	1.8	1	TLLMENAER + Oxidation (M)
94	460.2562	918.4978	918.4705	29.8	1	14	4.1	1	EVTGAMRR
82	443.7305	885.4464	885.4304	18.1	1	14	2.4	1	KDPNAEGR
25	574.3228	573.3155	573.3122	5.78	0	13	6.1	1	DIQAK
521	846.4363	1690.8580	1690.8084	29.4	1	13	3	1	MTSTTPPGEDIKSSPK + Oxidation (M)
369	730.9024	1459.7902	1459.7453	30.8	1	13	2.7	1	VDILENRVMDTR
122	517.2615	1032.5084	1032.4724	35.0	0	13	4	1	VLDEEGSER
219	585.3231	1168.6316	1168.6492	-15.03	1	13	2.9	1	KVSSFDVFLK
236	616.3319	1230.6492	1230.6245	20.1	0	13	4.1	1	AWVEQEGVSVK
763	1019.4843	4073.9081	4074.0473	-34.18	1	13	0.98	1	SSLSARLISMTDLLSSSAAPLSSALVHSSAQLSCRPDSR + Oxidation (M)
79	438.7601	875.5056	875.4786	30.9	1	13	2.9	1	KMDELLK
123	517.2618	1032.5090	1032.4724	35.5	0	13	4.4	1	VLDEEGSER
55	412.7522	823.4898	823.4817	9.93	0	13	1.4	1	RPWLPR
177	554.2685	1106.5224	1106.5251	-2.38	1	13	3.7	1	SGCGLTRSNR
286	647.3292	1292.6438	1292.6336	7.96	0	13	4.2	1	TFNAPWMALAR + Oxidation (M)
67	428.7656	855.5166	855.5178	-1.34	0	12	2.9	1	LLSTPGLR
325	672.3442	1342.6738	1342.6729	0.73	0	11	5.3	1	QDVTLEPSPTTR
77	437.7505	873.4864	873.4807	6.55	0	11	7.8	1	VAVEELSK
74	435.7740	869.5334	869.5123	24.3	0	11	2.9	1	VSLWLPR
73	435.7737	869.5328	869.5123	23.6	0	11	3	1	VSLWLPR
269	631.7998	1261.5850	1261.5826	1.92	0	11	4.7	1	EALEVDWSGEK
146	529.2684	1056.5222	1056.5464	-22.90	1	11	5.3	1	HELFREAR
65	428.7642	855.5138	855.5112	3.04	1	11	4.3	1	ALRMLPR
127	517.7664	1033.5182	1033.5338	-15.09	1	11	8.4	1	CISRTEIR
97	461.2222	920.4298	920.4716	-45.31	0	10	7	1	ISAAGYPSR
431	785.8430	1569.6714	1569.7254	-34.40	1	10	2.5	1	MSVFPRDWMVMR + Oxidation (M)
62	421.7567	841.4988	841.4810	21.2	0	10	5.1	1	AVLTWPR
380	738.3709	1474.7272	1474.6787	32.9	1	10	6.8	1	VDTEPKAEEASGDK
274	634.3513	1266.6880	1266.7084	-16.09	1	10	5.1	1	YLYGIRVEVR
162	539.2801	1076.5456	1076.5648	-17.79	1	10	8.8	1	MIGERNITK + Oxidation (M)
392	738.3838	1474.7530	1474.8065	-36.24	1	10	7.1	1	VESLLENMGIKVK + Oxidation (M)
245	618.2814	1234.5482	1234.5426	4.60	0	10	4.5	1	ETAGSSGSTPEGR
169	545.2915	1088.5684	1088.5397	26.4	1	10	9.1	1	MGSADRPALR + Oxidation (M)
466	804.3981	1606.7816	1606.8454	-39.66	0	10	6.9	1	VEVELLSYETQGLK
640	1040.0016	2077.9886	2077.8876	48.6	0	10	5.1	1	LMCSLCHCPGATIGCDVK
391	738.3832	1474.7518	1474.7813	-19.99	1	10	7.9	1	VKALEESAVNMLR + Oxidation (M)
691	837.3803	2509.1191	2509.1301	-4.39	0	10	3.2	1	LTLQSHRPTCCCYLDFHSSK
323	671.3476	1340.6806	1340.6282	39.1	0	10	7.4	1	AVSPENMVTYSK + Oxidation (M)
173	546.7579	1091.5012	1091.5393	-34.88	1	10	6.2	1	KEMASGVNTR
57	412.7530	823.4914	823.4817	11.9	0	10	3.1	1	RPWLPR
270	631.8015	1261.5884	1261.5826	4.61	0	10	7	1	EALEVDWSGEK
706	894.7648	2681.2726	2681.2755	-1.11	0	9	4.5	1	VVSAMVNSLDDNGVLIGNWTGDYSR
49	379.7106	757.4066	757.3719	45.9	0	9	11	1	DGPLNSR
102	485.7497	969.4848	969.5131	-29.16	0	9	7	1	VVEGPGTPSK
368	730.9019	1459.7892	1459.7453	30.1	1	9	7.2	1	VDILENRVMDTR
705	894.4244	2680.2514	2680.3420	-33.81	1	9	4.1	1	NLTAGMAMITCREPLLMSISTNLK + Oxidation (M)
168	545.2596	1088.5046	1088.5462	-38.16	0	9	7.3	1	SSVLDANLDR
324	671.3478	1340.6810	1340.7048	-17.73	0	9	9.2	1	NPQSTSLLPTGAR
154	532.2648	1062.5150	1062.4764	36.4	0	9	11	1	DASISSCPAR
80	438.7608	875.5070	875.4786	32.5	1	9	7.9	1	KLEMDLK

☑	149	529.2750	1056.5354	1056.5312	4.02	0	9	10	1	QADGQAGAALR
☑	564	863.3917	1724.7688	1724.7458	13.4	0	9	5.5	1	GVSEGSAAMAAGESMAQR + Oxidation (M)
☑	384	738.3768	1474.7390	1474.8065	-45.73	1	9	10	1	VESLLENMGIKVK + Oxidation (M)
☑	271	631.8031	1261.5916	1261.5826	7.15	0	8	9.1	1	EALEVDWSGEK
☑	58	412.7545	823.4944	823.4817	15.5	0	8	4	1	RPWLPR
☑	417	774.9011	1547.7876	1547.7865	0.76	1	8	10	1	MEEQGLADASLLKK + Oxidation (M)
☑	552	852.4298	1702.8450	1702.8825	-21.99	1	8	9.9	1	TQVGVKGCYAHVLGSK
☑	388	738.3810	1474.7474	1474.6787	46.6	1	8	12	1	VDTEPKAEEASGDK
☑	361	711.3162	1420.6178	1420.6681	-35.39	1	8	5.1	1	SLSSQEAKEAEDK
☑	268	631.3294	1260.6442	1260.6649	-16.36	0	8	13	1	LTDPFRPMLR + Oxidation (M)
☑	61	419.2105	836.4064	836.3810	30.4	1	8	12	1	QMKGDSR + Oxidation (M)
☑	390	738.3816	1474.7486	1474.6787	47.4	1	8	12	1	VDTEPKAEEASGDK
☑	631	656.6597	1966.9573	1966.9670	-4.96	0	8	9.3	1	VSVPTHPDDVGDASLTMVK
☑	63	421.7579	841.5012	841.4810	24.0	0	8	9.1	1	AVLTWPR
☑	275	634.3516	1266.6886	1266.7230	-27.14	1	8	9.1	1	MEPRIKPRPK + Oxidation (M)
☑	711	916.4344	2746.2814	2746.2593	8.03	0	8	5.8	1	LGYCYVEEPQEKPMPCWLYLR + Oxidation (M)
☑	103	485.7501	969.4856	969.4477	39.1	0	8	9.6	1	TMLEYADK
☑	152	530.7871	1059.5596	1059.5284	29.5	1	8	18	1	VWHTMSKR + Oxidation (M)
☑	731	958.4579	2872.3519	2872.3337	6.31	1	8	6.4	1	TQQETFLEVIANASGYMYETSYRR + Oxidation (M)
☑	70	435.2076	868.4006	868.4290	-32.70	1	8	7.8	1	GKFSTDSK
☑	18	531.2912	530.2839	530.2813	5.04	0	8	26	1	AVADR
☑	329	672.8435	1343.6724	1343.6906	-13.47	1	8	12	1	LASRQSTPSQNR
☑	199	573.7952	1145.5758	1145.5751	0.69	0	8	15	1	QMGLGEPSVTK
☑	385	738.3773	1474.7400	1474.6787	41.6	1	8	13	1	VDTEPKAEEASGDK
☑	130	519.2655	1036.5164	1036.4681	46.6	0	8	13	1	DPATMLMSR + Oxidation (M)
☑	131	519.2684	1036.5222	1036.5739	-49.84	0	8	13	1	IYCLLSLR
☑	241	618.2693	1234.5240	1234.5677	-35.37	0	8	5.3	1	STDSEVSQSPAK
☑	26	581.3032	580.2959	580.2890	11.9	0	8	11	1	SSMLK + Oxidation (M)
☑	382	738.3743	1474.7340	1474.8065	-49.12	1	8	13	1	VESLLENMGIKVK + Oxidation (M)
☑	36	644.3062	643.2989	643.2748	37.5	0	8	5.1	1	GCALSH
☑	209	579.2987	1156.5828	1156.5585	21.1	1	7	14	1	AGSEPAGERQR
☑	72	435.7737	869.5328	869.5698	-42.50	1	7	7.2	1	VLKILER
☑	164	539.2836	1076.5526	1076.5437	8.32	1	7	16	1	QGAVMKNWK + Oxidation (M)
☑	155	532.2737	1062.5328	1062.5128	18.9	1	7	17	1	QEIDRMQK + Oxidation (M)
☑	211	580.7757	1159.5368	1159.5581	-18.36	1	7	11	1	ELQDEGRSAR
☑	356	697.3420	1392.6694	1392.6919	-16.10	0	7	14	1	MTLTAASEVIGER + Oxidation (M)
☑	182	556.7426	1111.4706	1111.4638	6.20	1	7	5.5	1	EGMERESMK + Oxidation (M)
☑	422	785.3591	1568.7036	1568.7551	-32.82	1	7	8.8	1	FKYYSFFDLDPK
☑	242	618.2741	1234.5336	1234.5910	-46.49	0	7	8.1	1	MSTARPCAGLR + Oxidation (M)
☑	254	629.8081	1257.6016	1257.6248	-18.41	1	7	15	1	QRMSPGVAGSPR + Oxidation (M)
☑	153	530.7873	1059.5600	1059.5560	3.79	0	7	23	1	DTSSILATPR
☑	387	738.3804	1474.7462	1474.6908	37.6	0	7	16	1	HNLLCLENMTSK + Oxidation (M)
☑	751	833.1402	3328.5317	3328.5380	-1.89	0	7	5.3	1	AMNDIGDYVGSNLEISWLPNLDGLMEGYAR + Oxidation (M)
☑	120	516.3018	1030.5890	1030.5546	33.4	0	7	15	1	VDILDIDTK
☑	167	544.7650	1087.5154	1087.5370	-19.83	1	6	16	1	SADIGARADGR
☑	319	670.8413	1339.6680	1339.7037	-26.63	0	6	16	1	QQVSPPWVPFR
☑	320	670.8761	1339.7376	1339.7459	-6.19	1	6	13	1	KAELEGVAVTAPR
☑	600	915.4284	1828.8422	1828.9068	-35.29	0	6	11	1	ASGGSGGVSVPALWSEVNR
☑	383	738.3758	1474.7370	1474.6908	31.4	0	6	17	1	HNLLCLENMTSK + Oxidation (M)
☑	710	916.4273	2746.2601	2746.2625	-0.89	1	6	7.5	1	SSDMTCAPHRPELEMRSYLDVPR
☑	118	515.7795	1029.5444	1029.5753	-30.00	1	6	22	1	QACGVVLRK
☑	59	414.2218	826.4290	826.3933	43.2	0	6	13	1	NEQPPSR
☑	611	973.5194	1945.0242	1944.9364	45.2	1	6	11	1	VIEAYCTSASFQQGTRK
☑	119	516.3002	1030.5858	1030.6321	-44.87	1	6	16	1	MLLLTLRR + Oxidation (M)
☑	407	756.8483	1511.6820	1511.6939	-7.86	1	6	12	1	YYGTSMHERVNR
☑	244	618.2772	1234.5398	1234.5677	-22.55	1	6	10	1	SELEEKDTER
☑	157	533.2596	1064.5046	1064.5251	-19.18	0	6	17	1	VYGQGLSEGR
☑	19	532.2899	531.2826	531.2653	32.7	0	6	37	1	SSNPK
☑	377	736.3522	1470.6898	1470.6517	26.0	0	6	13	1	SMSLCNIAMTPSK + 2 Oxidation (M)
☑	178	554.2697	1106.5248	1106.5754	-45.66	1	6	18	1	KAVTDAIMSR + Oxidation (M)
☑	60	414.2234	826.4322	826.4661	-40.94	0	6	14	1	VAAPNLSR
☑	608	954.8972	1907.7798	1907.7561	12.5	1	6	3.6	1	KMGGDDVMACVHDDNGR + 2 Oxidation (M)
☑	393	738.3840	1474.7534	1474.6908	42.5	0	6	19	1	HNLLCLENMTSK + Oxidation (M)
☑	86	450.2203	898.4260	898.4621	-40.09	1	6	12	1	REPGEVGR
☑	135	523.2825	1044.5504	1044.5101	38.6	1	6	26	1	KGAAGWEGGGR
☑	359	466.8943	1397.6611	1397.6364	17.6	1	6	17	1	DSPKSWFFDNR
☑	180	555.2522	1108.4898	1108.5183	-25.62	1	6	12	1	ENTSKVEMR + Oxidation (M)
☑	156	533.2565	1064.4984	1064.5073	-8.31	0	6	18	1	NFNICLER
☑	124	517.2717	1032.5288	1032.5200	8.58	0	6	24	1	GEVVTAGASSR
☑	322	671.3471	1340.6796	1340.6248	40.9	0	6	19	1	YGYTEDPLEVR
☑	692	837.3954	2509.1644	2509.1667	-0.94	1	5	12	1	TIETEEASPSTESLKSTSSDPGTR
☑	111	496.2291	990.4436	990.4077	36.3	0	5	15	1	VCSDEPER
☑	672	761.7168	2282.1286	2282.0572	31.3	1	5	15	1	HCVSAGEPINPEVMEQWRK + Oxidation (M)
☑	235	615.3110	1228.6074	1228.6663	-47.92	0	5	22	1	TIVAVEVQDQK
☑	147	529.2686	1056.5226	1056.5716	-46.35	0	5	21	1	LPTALWSGGR
☑	318	670.3047	1338.5948	1338.5809	10.4	0	5	14	1	GGFGEVCACQVR
☑	328	672.8430	1343.6714	1343.6681	2.51	1	5	22	1	AQGDEQEAQLKK
☑	106	487.7730	973.5314	973.4950	37.5	1	5	32	1	ICPICRR
☑	412	766.3771	1530.7396	1530.7474	-5.04	1	5	23	1	AADTKHHHSMIQR
☑	743	1026.4631	3076.3675	3076.4223	-17.83	0	5	6.7	1	PDPISVSEFVAETLEDYMAPTASSFTTR + Oxidation (M)
☑	617	652.0220	1953.0442	1953.0758	-16.17	0	5	15	1	QGFCIPVEPGKPGLLLTK

☑	137	523.7651	1045.5156	1045.5127	2.78	1	5	34	1	FPPVDCRR
☑	30	601.3463	600.3390	600.3595	-34.10	0	5	26	1	QVINK
☑	643	1040.0198	2078.0250	2078.0895	-31.01	1	5	19	1	LEVAKLPLTEHELESENK
☑	198	572.8111	1143.6076	1143.6546	-41.07	1	5	31	1	QAMLRQLLR + Oxidation (M)
☑	633	656.9688	1967.8846	1967.8417	21.8	1	4	13	1	ASETQTSGRGSSASGSGAGDQA
☑	406	756.8435	1511.6724	1511.7144	-27.75	0	4	15	1	EVVESPQPPFPGET
☑	87	450.2206	898.4266	898.4192	8.34	1	4	17	1	GAHMDRGR
☑	28	590.3152	589.3079	589.3184	-17.71	0	4	57	1	TLNSR
☑	441	785.8559	1569.6972	1569.7569	-38.00	1	4	14	1	EMHEEEVLNIRR + Oxidation (M)
☑	626	656.6524	1966.9354	1966.9758	-20.53	0	4	20	1	AAFLGTFGTVLQVPCACR
☑	577	595.3222	1782.9448	1782.8862	32.9	0	4	19	1	MIQGDYIEKPDFALK + Oxidation (M)
☑	240	616.8068	1231.5990	1231.6197	-16.75	1	4	30	1	DLEKNYPTPR
☑	588	897.9125	1793.8104	1793.8366	-14.60	1	4	18	1	QTWTDEGSVSERMLR
☑	628	656.6563	1966.9471	1966.9670	-10.14	0	4	23	1	VSVPTHPDDVGDASLTMVK
☑	117	514.2878	1026.5610	1026.5763	-14.88	0	4	25	1	WVFLIGHR
☑	121	516.3024	1030.5902	1030.6209	-29.74	0	4	28	1	VLVIVMTTR
☑	78	438.7511	875.4876	875.5076	-22.79	1	4	35	1	SLSSKLNK
☑	371	730.9055	1459.7964	1459.7592	25.5	1	4	24	1	DVLEKMELLDQK
☑	429	785.8380	1569.6614	1569.6837	-14.19	0	4	9.6	1	VTDVMLCAGEMSGGK + Oxidation (M)
☑	435	785.8458	1569.6770	1569.7232	-29.41	0	4	13	1	LAGMEEEELAFSTK + Oxidation (M)
☑	110	496.2278	990.4410	990.4804	-39.74	0	4	21	1	LEDMQALR + Oxidation (M)
☑	34	630.3195	629.3122	629.3133	-1.70	0	3	18	1	SDGVPR
☑	109	496.2252	990.4358	990.4744	-38.89	0	3	20	1	LGGNHSNHR
☑	527	849.4228	1696.8310	1696.8818	-29.91	1	3	29	1	AFLGELKTQMTSTVR + Oxidation (M)
☑	93	458.7181	915.4216	915.3974	26.5	0	3	21	1	SSSWSYSL
☑	215	581.7887	1161.5628	1161.5448	15.5	0	3	41	1	MEEQTQQIR
☑	550	852.4276	1702.8406	1702.8825	-24.57	1	3	30	1	TQVGVKGCYAHVLGSK
☑	630	656.6592	1966.9558	1966.9418	7.11	1	3	27	1	ALQEFSELNERMSELR + Oxidation (M)
☑	669	737.6966	2210.0680	2210.0776	-4.37	1	3	24	1	TNSMSFLPVVKEEAEESSVK
☑	745	801.6301	3202.4913	3202.5964	-32.82	1	3	13	1	TVCYSPEGDMVAIGMKNGEFIILLVSSLK + 2 Oxidation (M)
☑	459	801.4326	1600.8506	1600.8243	16.5	1	3	27	1	CHLKLSLDNLSSSK
☑	327	672.8421	1343.6696	1343.7045	-25.91	1	3	33	1	KEASSGSTPQKPK
☑	181	556.2957	1110.5768	1110.6033	-23.83	0	3	32	1	TVISPDPNLR
☑	629	656.6565	1966.9477	1966.9385	4.67	0	3	28	1	ASLVSSSDGSFLADTHFAR
☑	267	631.3170	1260.6194	1260.5775	33.3	0	3	39	1	FYETVGQDFR
☑	276	634.3563	1266.6980	1266.6720	20.5	1	3	25	1	RNIIGYFEQK
☑	238	616.8051	1231.5956	1231.6561	-49.04	1	3	38	1	AKALESQFPNK
☑	673	762.0465	2283.1177	2283.0550	27.5	1	3	27	1	AVDSACPELTGSSGGPGSREPPR
☑	591	897.9169	1793.8192	1793.7699	27.5	1	3	28	1	MSYLKTTMEDEESSK + Oxidation (M)
☑	68	428.7694	855.5242	855.5178	7.55	0	2	25	1	VTIASLPR
☑	670	737.6982	2210.0728	2209.9984	33.7	1	2	28	1	RMLSDSGMITPHYEDSDLK + Oxidation (M)
☑	228	601.7988	1201.5830	1201.5952	-10.14	1	2	41	1	TPRSFSTHGGR
☑	239	616.8063	1231.5980	1231.5768	17.3	1	2	44	1	HNGYDPRVMK + Oxidation (M)
☑	574	890.4332	1778.8518	1778.8010	28.6	1	2	36	1	MVMTVFACLMGRGMK + 3 Oxidation (M)
☑	195	572.8048	1143.5950	1143.5924	2.32	0	2	51	1	NILYHLDEK
☑	693	837.3973	2509.1701	2509.2531	-33.11	0	2	24	1	MVQSLAWAGVMTLLMVQWGSAAK + 2 Oxidation (M)
☑	188	565.3242	1128.6338	1128.6502	-14.52	1	2	43	1	KLELEATVAR
☑	570	885.9603	1769.9060	1769.9635	-32.47	1	2	38	1	ENQRLQEQILSLTAK
☑	99	475.7538	949.4930	949.4869	6.48	0	2	52	1	IGDTIEFR
☑	620	652.3461	1954.0165	1954.0023	7.27	1	2	33	1	AVVVGFDPHFSYMKLTK + Oxidation (M)
☑	339	458.9060	1373.6962	1373.6688	20.0	1	2	50	1	QWRNISDVDNK
☑	528	851.4495	1700.8844	1700.8920	-4.41	1	2	38	1	DKDTLSIHYLMLPR
☑	639	1039.9996	2077.9846	2077.9132	34.4	0	2	32	1	CPNGHIYVITECGGAMQR + Oxidation (M)
☑	148	529.2691	1056.5236	1056.5426	-17.95	0	2	42	1	LMTELWHK
☑	704	894.0798	2679.2176	2679.3439	-47.13	1	2	20	1	EMLLKPHSRVQVFEGAEDNLPDR
☑	451	795.8800	1589.7454	1589.7508	-3.35	0	2	36	1	QASDSSMFLPPSPAR
☑	365	728.3507	1454.6868	1454.7365	-34.13	0	2	37	1	EQISQHIADLSSK
☑	638	688.3139	2061.9199	2061.9426	-11.01	1	2	24	1	SKGSQGTSPFPMSPPSPDSR + Oxidation (M)
☑	590	897.9169	1793.8192	1793.7699	27.5	1	2	34	1	MSYLKTTMEDEESSK + Oxidation (M)
☑	555	854.3911	1706.7676	1706.7794	-6.91	1	2	30	1	MNSFRANHSSLDNSK
☑	190	572.3210	1142.6274	1142.6448	-15.16	0	2	54	1	AALLDPFAAVR
☑	625	656.6497	1966.9273	1966.9418	-7.41	0	2	34	1	SCSVTDTVAEQAHLPPGAK
☑	667	1073.0267	2144.0388	2144.0911	-24.36	1	2	37	1	SLSAFAPICNPVLCPWGKK
☑	179	554.2782	1106.5418	1106.5581	-14.68	1	2	56	1	QSFSRQAQR
☑	439	785.8525	1569.6904	1569.6671	14.9	0	1	26	1	EDTGWYWCGIQR
☑	516	824.3876	1646.7606	1646.8055	-27.22	1	1	41	1	GLRTLCCCLPIER
☑	535	851.4545	1700.8944	1700.8920	1.46	1	1	42	1	NPKILVECLTPDFR
☑	715	917.0835	2748.2287	2748.3371	-39.44	1	1	19	1	RPTLTGCSLPGASCGPGRCPAGPVPAR
☑	732	958.4640	2872.3702	2872.2763	32.7	0	1	29	1	ASQSVDFDGDSFMNWYQQKPGQPPK + Oxidation (M)
☑	556	854.3937	1706.7728	1706.7794	-3.86	1	1	36	1	MNSFRANHSSLDNSK
☑	607	624.3091	1869.9055	1869.8349	37.7	0	1	48	1	FPCNGNSQAGLTTQTMK + Oxidation (M)
☑	627	656.6545	1966.9417	1967.0323	-46.10	1	1	41	1	LGSSSLTSIPEEVREHVK
☑	138	525.2727	1048.5308	1048.5263	4.33	0	1	76	1	GIDMIEFPK
☑	40	325.1874	648.3602	648.3595	1.16	0	1	49	1	AFNAVK
☑	423	785.8161	1569.6176	1569.6518	-21.76	0	1	7.1	1	DVTCDVHYENYR
☑	321	671.3409	1340.6672	1340.7201	-39.40	1	1	57	1	SLSRTPSPPPFR
☑	378	736.4106	1470.8066	1470.7943	8.40	0	1	43	1	QVLSQQSLYLHR
☑	100	476.7474	951.4802	951.5178	-39.46	1	1	65	1	RLPIGSYF
☑	656	1048.9799	2095.9452	2095.9844	-18.70	0	1	34	1	TPEVSQSILMSSFGNQDTR
☑	467	805.8967	1609.7788	1609.7592	12.2	1	1	53	1	TIDSWMNTMVPKR + 2 Oxidation (M)
☑	729	958.1341	2871.3805	2871.4628	-28.66	1	1	31	1	GFSQSGAPLLTLQSVRLQFQETDGHR

39	325.1860	648.3574	648.3629	-8.36	1	1	67	1	GLGMKK + Oxidation (M)
680	785.4049	2353.1929	2353.2715	-33.42	1	0	40	1	IVKYLALQDLMLLSQYSPSR + Oxidation (M)
37	325.1857	648.3568	648.3629	-9.29	1	0	71	1	GLGMKK + Oxidation (M)
85	449.2170	896.4194	896.4538	-38.32	1	0	46	1	GYTMAAKR
734	963.4579	2887.3519	2887.4327	-27.99	0	0	33	1	FLTNMTITNDYQHLLVNSIANFFR + Oxidation (M)
306	657.3536	1312.6926	1312.7099	-13.15	1	0	69	1	ADVKTPGGTALQR
1	366.9022	365.8949							
2	396.9575	395.9502							
3	416.2356	415.2283							
4	416.2481	415.2408							
5	423.2329	422.2256							
6	431.2560	430.2487							
7	435.2127	434.2054							
8	435.2151	434.2078							
9	445.1303	444.1230							
10	445.2416	444.2343							
11	446.2505	445.2432							
12	446.2646	445.2573							
14	476.2701	475.2628							
15	487.1823	486.1750							
16	490.2626	489.2553							
17	503.1149	502.1076							
20	545.2539	544.2466							
21	545.2953	544.2880							
22	554.3151	553.3078							
23	558.3015	557.2942							
24	573.3213	572.3140							
29	600.3272	599.3199							
31	615.3143	614.3070							
32	624.3177	623.3104							
33	624.8013	623.7940							
35	632.3370	631.3297							
38	325.1857	648.3568							
42	677.1953	676.1880							
43	691.3529	690.3456							
44	698.3786	697.3713							
45	717.7251	716.7178							
46	719.3228	718.3155							
47	736.4116	735.4043							
48	753.3764	752.3691							
50	768.3764	767.3691							
51	786.3768	785.3695							
52	396.7395	791.4644							
53	805.4037	804.3964							
54	808.4230	807.4157							
64	846.4410	845.4337							
75	435.7748	869.5350							
76	872.4955	871.4882							
81	885.8868	884.8795							
83	892.4476	891.4403							
88	450.7097	899.4048							
91	452.7321	903.4496							
92	452.7397	903.4648							
95	920.4788	919.4715							
96	920.5129	919.5056							
98	929.5637	928.5564							
101	482.2186	962.4226							
107	978.5477	977.5404							
108	491.7354	981.4562							
115	505.7493	1009.4840							
129	519.1622	1036.3098							
145	529.2667	1056.5188							
151	530.2933	1058.5720							
161	539.2780	1076.5414							
174	552.2900	1102.5654							
186	563.3021	1124.5896							
187	565.2789	1128.5432							
200	574.3109	1146.6072							
203	574.3262	1146.6378							
220	587.8072	1173.5998							
221	588.3449	1174.6752							
225	595.3173	1188.6200							
226	1190.5615	1189.5542							
232	615.3006	1228.5866							
233	615.3041	1228.5936							
234	615.3063	1228.5980							
243	618.2758	1234.5370							
248	622.8178	1243.6210							
253	629.3243	1256.6340							
255	631.2940	1260.5734							
272	632.3372	1262.6598							

☑	278	638.8154	1275.6162
☑	284	643.7996	1285.5846
☑	285	646.8150	1291.6154
☑	287	648.3736	1294.7326
☑	307	660.3164	1318.6182
☑	326	672.8339	1343.6532
☑	344	692.3312	1382.6478
☑	349	692.8626	1383.7106
☑	350	692.8760	1383.7374
☑	351	692.8798	1383.7450
☑	352	693.8698	1385.7250
☑	364	726.3497	1450.6848
☑	366	728.8524	1455.6902
☑	367	730.8618	1459.7090
☑	379	736.8534	1471.6922
☑	381	738.3735	1474.7324
☑	386	738.3793	1474.7440
☑	389	738.3812	1474.7478
☑	394	738.3854	1474.7562
☑	400	740.3684	1478.7222
☑	401	740.3709	1478.7272
☑	402	745.8734	1489.7322
☑	403	746.8355	1491.6564
☑	404	748.8627	1495.7108
☑	408	756.8687	1511.7228
☑	409	757.8530	1513.6914
☑	410	757.8616	1513.7086
☑	411	760.8881	1519.7616
☑	413	766.8620	1531.7094
☑	414	766.8657	1531.7168
☑	415	768.3747	1534.7348
☑	416	770.3876	1538.7606
☑	418	775.8670	1549.7194
☑	419	775.8695	1549.7244
☑	420	776.3786	1550.7426
☑	421	784.3751	1566.7356
☑	424	785.8351	1569.6556
☑	425	785.8369	1569.6592
☑	426	785.8369	1569.6592
☑	427	785.8372	1569.6598
☑	428	785.8378	1569.6610
☑	430	785.8421	1569.6696
☑	432	785.8441	1569.6736
☑	433	785.8449	1569.6752
☑	434	785.8456	1569.6766
☑	436	785.8458	1569.6770
☑	438	785.8497	1569.6848
☑	440	785.8525	1569.6904
☑	442	785.8585	1569.7024
☑	443	785.8590	1569.7034
☑	444	786.8485	1571.6824
☑	445	786.8568	1571.6990
☑	446	793.4191	1584.8236
☑	447	794.3834	1586.7522
☑	448	794.8438	1587.6730
☑	449	794.8445	1587.6744
☑	450	794.8522	1587.6898
☑	452	795.8813	1589.7480
☑	453	795.8821	1589.7496
☑	460	801.9117	1601.8088
☑	461	801.9121	1601.8096
☑	472	808.9174	1615.8202
☑	473	808.9254	1615.8362
☑	474	808.9262	1615.8378
☑	482	810.8880	1619.7614
☑	483	810.8907	1619.7668
☑	484	810.8946	1619.7746
☑	485	811.3986	1620.7826
☑	486	814.3635	1626.7124
☑	497	816.8936	1631.7726
☑	499	816.8942	1631.7738
☑	502	816.8950	1631.7754
☑	514	816.9001	1631.7856
☑	515	816.9039	1631.7932
☑	525	846.9399	1691.8652
☑	531	851.4514	1700.8882
☑	538	851.4555	1700.8964
☑	549	852.4240	1702.8334
☑	551	852.4293	1702.8440
☑	553	853.9323	1705.8500
☑	554	854.3732	1706.7318

☑	557	854.4129	1706.8112
☑	563	858.4524	1714.8902
☑	565	863.4109	1724.8072
☑	566	870.8540	1739.6934
☑	567	870.8572	1739.6998
☑	568	870.8726	1739.7306
☑	569	883.4468	1764.8790
☑	571	886.9477	1771.8808
☑	572	890.4144	1778.8142
☑	575	595.3120	1782.9142
☑	576	595.3168	1782.9286
☑	586	896.3635	1790.7124
☑	587	896.3699	1790.7252
☑	589	897.9144	1793.8142
☑	592	904.9169	1807.8192
☑	593	906.4688	1810.9230
☑	594	906.4699	1810.9252
☑	599	913.0020	1823.9894
☑	605	920.9665	1839.9184
☑	606	930.0057	1857.9968
☑	609	954.9168	1907.8190
☑	610	973.5134	1945.0122
☑	612	650.9512	1949.8318
☑	619	652.0237	1953.0493
☑	634	1003.5219	2005.0292
☑	636	1004.9890	2007.9634
☑	637	1024.5085	2047.0024
☑	641	1040.0059	2077.9972
☑	642	1040.0195	2078.0244
☑	644	1040.5087	2079.0028
☑	645	1040.5095	2079.0044
☑	657	701.6409	2101.9009
☑	658	1057.0076	2112.0006
☑	659	707.3395	2118.9967
☑	660	707.6843	2120.0311
☑	661	1062.4869	2122.9592
☑	666	1073.0200	2144.0254
☑	668	1098.0914	2194.1682
☑	671	737.7042	2210.0908
☑	674	779.0428	2334.1066
☑	675	1168.5682	2335.1218
☑	683	785.4280	2353.2622
☑	689	837.3531	2509.0375
☑	690	837.3764	2509.1074
☑	694	651.8444	2603.3485
☑	695	888.4140	2662.2202
☑	700	888.7613	2663.2621
☑	702	888.7630	2663.2672
☑	707	911.4393	2731.2961
☑	708	912.4248	2734.2526
☑	709	916.4249	2746.2529
☑	712	917.0737	2748.1993
☑	713	917.0783	2748.2131
☑	714	917.0785	2748.2137
☑	716	917.0837	2748.2293
☑	717	917.0848	2748.2326
☑	718	922.4142	2764.2208
☑	719	922.4227	2764.2463
☑	720	922.4397	2764.2973
☑	721	927.7492	2780.2258
☑	722	932.4215	2794.2427
☑	723	702.3979	2805.5625
☑	724	938.3890	2812.1452
☑	725	1415.1267	2828.2388
☑	726	958.1252	2871.3538
☑	727	958.1253	2871.3541
☑	728	958.1334	2871.3784
☑	730	958.1431	2871.4075
☑	733	958.4709	2872.3909
☑	735	963.4701	2887.3885
☑	736	963.7979	2888.3719
☑	737	968.7911	2903.3515
☑	738	969.1218	2904.3436
☑	739	969.1257	2904.3553
☑	740	973.4473	2917.3201
☑	741	753.3869	3009.5185
☑	742	767.4480	3065.7629
☑	744	1066.1575	3195.4507
☑	746	1082.2153	3243.6241
☑	747	816.6345	3262.5089
☑	748	1088.8136	3263.4190

☑	749	*1088.8392*	*3263.4958*
☑	750	*1106.5133*	*3316.5181*
☑	752	*841.9054*	*3363.5925*
☑	753	*854.3892*	*3413.5277*
☑	754	*1188.5891*	*3562.7455*
☑	755	*892.1990*	*3564.7669*
☑	756	*894.1323*	*3572.5001*
☑	757	*898.2284*	*3588.8845*
☑	758	*905.8012*	*3619.1757*
☑	759	*920.1791*	*3676.6873*
☑	760	*953.6735*	*3810.6649*
☑	761	*984.5250*	*3934.0709*
☑	762	*1004.2122*	*4012.8197*
☑	764	*1024.5935*	*4094.3449*
☑	765	*1088.9841*	*4351.9073*
☑	766	*1094.2787*	*4373.0857*
☑	767	*1111.0824*	*4440.3005*
☑	768	*1130.5969*	*4518.3585*

Search Parameters

```
Type of search         : MS/MS Ion Search
Enzyme                 : Trypsin
Fixed modifications    : Carbamidomethyl (C)
Variable modifications : Oxidation (M)
Mass values            : Monoisotopic
Protein Mass           : Unrestricted
Peptide Mass Tolerance : ± 50 ppm
Fragment Mass Tolerance: ± 0.2 Da
Max Missed Cleavages   : 1
Instrument type        : ESI-QUAD-TOF
Number of queries      : 768
```

Mascot: http://www.matrixscience.com/

Mascot Search Results

```
User          : Dominic
Email         : dominic.kurian@bbsrc.ac.uk
Search title  : IPI Mouse_15-02-2010
MS data file  : D:\Peaklist\Dominic Kurian\QTofDK20010805 GATC P 0010883.pkl
Database      : IPI_mouse MOUSE_v3_79 (54943 sequences; 24917557 residues)
Timestamp     : 15 Feb 2011 at 15:25:08 GMT
Protein hits  : IPI00621806 Tax_Id=10090 Gene_Symbol=Camk2a Isoform Alpha CaMKII of Calcium/calmodulin-dependent protein kinase type
                IPI00474502 Tax_Id=10090 Gene_Symbol=Camk2b Calcium/calmodulin-dependent protein kinase type II subunit beta
                IPI00406790 Tax_Id=10090 Gene_Symbol=Camk2d Isoform 4 of Calcium/calmodulin-dependent protein kinase type II subunit
                IPI00124695 Tax_Id=10090 Gene_Symbol=Camk2g Isoform 1 of Calcium/calmodulin-dependent protein kinase type II subunit
                IPI00169463 Tax_Id=10090 Gene_Symbol=Tubb2c Tubulin beta-2C chain
                IPI00110850 Tax_Id=10090 Gene_Symbol=Actb Actin, cytoplasmic 1
                IPI00115546 Tax_Id=10090 Gene_Symbol=Gnao1 Isoform Alpha-2 of Guanine nucleotide-binding protein G(o) subunit alpha
                IPI00221528 Tax_Id=10090 Gene_Symbol=Actbl2 Beta-actin-like protein 2
                IPI00112251 Tax_Id=10090 Gene_Symbol=Tubb3 Tubulin beta-3 chain
                IPI00323571 Tax_Id=10090 Gene_Symbol=Apoe Apolipoprotein E
                IPI00117352 Tax_Id=10090 Gene_Symbol=Tubb5 Tubulin beta-5 chain
                IPI00138892 Tax_Id=10090 Gene_Symbol=Uba52 Ubiquitin-60S ribosomal protein L40
                IPI00755181 Tax_Id=10090 Gene_Symbol=Krt10 keratin, type I cytoskeletal 10
                IPI00122094 Tax_Id=10090 Gene_Symbol=Dlg4 Isoform 2 of Disks large homolog 4
                IPI00131366 Tax_Id=10090 Gene_Symbol=Krt6b Keratin, type II cytoskeletal 6B
                IPI00134997 Tax_Id=10090 Gene_Symbol=Dlgap3 Disks large-associated protein 3
                IPI00122928 Tax_Id=10090 Gene_Symbol=Tubb6 Tubulin beta-6 chain
                IPI00109061 Tax_Id=10090 Gene_Symbol=Tubb2b Tubulin beta-2B chain
                IPI00462140 Tax_Id=10090 Gene_Symbol=Krt77 Keratin, type II cytoskeletal 1b
                IPI00420970 Tax_Id=10090 Gene_Symbol=Krt74 Keratin, type II cytoskeletal 74
                IPI00131209 Tax_Id=10090 Gene_Symbol=Krt16 Keratin intermediate filament 16a
                IPI00348328 Tax_Id=10090 Gene_Symbol=Krt78 keratin Kb40
                IPI00322209 Tax_Id=10090 Gene_Symbol=Krt8 Keratin, type II cytoskeletal 8
                IPI00119063 Tax_Id=10090 Gene_Symbol=Lrp1 Prolow-density lipoprotein receptor-related protein 1
                IPI00121038 Tax_Id=10090 Gene_Symbol=Vcan Isoform V0 of Versican core protein
                IPI00122048 Tax_Id=10090 Gene_Symbol=Atp1a3 Sodium/potassium-transporting ATPase subunit alpha-3
                IPI00120793 Tax_Id=10090 Gene_Symbol=Prnp Major prion protein
                IPI00136402 Tax_Id=10090 Gene_Symbol=Dlgap1 Isoform 1 of Disks large-associated protein 1
                IPI00222548 Tax_Id=10090 Gene_Symbol=Rpl29;Gm13841;Gm8210 60S ribosomal protein L29
                IPI00124499 Tax_Id=10090 Gene_Symbol=Krt79 Keratin, type II cytoskeletal 79
                IPI00113410 Tax_Id=10090 Gene_Symbol=Hapln2 Hyaluronan and proteoglycan link protein 2
                IPI00223714 Tax_Id=10090 Gene_Symbol=Hist1h1e Histone H1.4
                IPI00136703 Tax_Id=10090 Gene_Symbol=Ckb Creatine kinase B-type
                IPI00229598 Tax_Id=10090 Gene_Symbol=Cnp Isoform CNPI of 2',3'-cyclic-nucleotide 3'-phosphodiesterase
                IPI00123176 Tax_Id=10090 Gene_Symbol=Gm6316 Glyceraldehyde-3-phosphate dehydrogenase
```

	IPI_mouse	Decoy	False discovery rate
Peptide matches above identity threshold	229	0	0.00 %
Peptide matches above homology or identity threshold	245	2	0.82 %

Probability Based Mowse Score

Ions score is -10*Log(P), where P is the probability that the observed match is a random event.
Individual ions scores > 37 indicate identity or extensive homology (p<0.01).
Protein scores are derived from ions scores as a non-probabilistic basis for ranking protein hits.

Score Distribution

Peptide Summary Report

Format As | Peptide Summary | Help

Significance threshold p< 0.01 | Max. number of hits AUTO

Standard scoring ○ MudPIT scoring ● Ions score or expect cut-off 0 | Show sub-sets 0

Show pop-ups ● Suppress pop-ups ○ Sort unassigned Decreasing Score | Require bold red ☐

Select All | Select None | Search Selected | ☐ **Error tolerant** | Archive Report

```
1.   IPI00621806     Mass: 54651     Score: 3316     Queries matched: 156     emPAI: 1.55
     Tax_Id=10090 Gene_Symbol=Camk2a Isoform Alpha CaMKII of Calcium/calmodulin-dependent protein kinase type II subunit alpha
   ☐ Check to include this hit in error tolerant search or archive report
```

	Query	Observed	Mr(expt)	Mr(calc)	ppm	Miss	Score	Expect	Rank	Peptide
☑	5	445.2779	444.2706	444.2696	2.25	0	(7)	29	1	K.GAAVK.L
☑	6	445.2791	444.2718	444.2696	4.95	0	8	27	1	K.GAAVK.L
☑	11	602.3611	601.3538	601.3435	17.2	0	12	12	1	K.DLINK.M

49	432.7308	863.4470	863.4501	-3.56	0	(24)	0.43	1	R.SGAPSVLPH.-
50	432.7333	863.4520	863.4501	2.23	0	(14)	3.8	1	R.SGAPSVLPH.-
51	432.7336	863.4526	863.4501	2.93	0	26	0.24	1	R.SGAPSVLPH.-
69	452.2506	902.4866	902.4895	-3.18	0	33	0.049	1	K.MLTINPSK.R
70	452.2533	902.4920	902.4895	2.80	0	(10)	10	1	K.MLTINPSK.R
72	461.2145	920.4144	920.4199	-5.95	0	23	0.31	1	R.TAQSEETR.V
116	525.2833	1048.5520	1048.5553	-3.09	0	(49)	0.0012	1	K.VLAGQEYAAK.I
117	525.2841	1048.5536	1048.5553	-1.56	0	(33)	0.044	1	K.VLAGQEYAAK.I
118	525.2841	1048.5536	1048.5553	-1.56	0	(45)	0.0028	1	K.VLAGQEYAAK.I
119	525.2849	1048.5552	1048.5553	-0.03	0	52	0.00048	1	K.VLAGQEYAAK.I
120	525.2850	1048.5554	1048.5553	0.16	0	(52)	0.00049	1	K.VLAGQEYAAK.I
121	525.2855	1048.5564	1048.5553	1.11	0	(45)	0.0028	1	K.VLAGQEYAAK.I
125	530.2988	1058.5830	1058.5906	-7.15	1	(38)	0.012	1	K.MLTINPSKR.I
126	530.2988	1058.5830	1058.5906	-7.15	1	(14)	3.6	1	K.MLTINPSKR.I
127	530.2996	1058.5846	1058.5906	-5.64	1	(48)	0.0014	1	K.MLTINPSKR.I
128	530.2996	1058.5846	1058.5906	-5.64	1	(49)	0.0012	1	K.MLTINPSKR.I
129	530.3013	1058.5880	1058.5906	-2.43	1	(46)	0.0019	1	K.MLTINPSKR.I
130	530.3015	1058.5884	1058.5906	-2.05	1	(38)	0.013	1	K.MLTINPSKR.I
131	530.3017	1058.5888	1058.5906	-1.68	1	52	0.00042	1	K.MLTINPSKR.I
132	530.3019	1058.5892	1058.5906	-1.30	1	(43)	0.0033	1	K.MLTINPSKR.I
133	530.3021	1058.5896	1058.5906	-0.92	1	(33)	0.041	1	K.MLTINPSKR.I
134	530.3024	1058.5902	1058.5906	-0.35	1	(40)	0.0077	1	K.MLTINPSKR.I
135	530.3027	1058.5908	1058.5906	0.21	1	(49)	0.001	1	K.MLTINPSKR.I
136	530.3054	1058.5962	1058.5906	5.31	1	(41)	0.006	1	K.MLTINPSKR.I
144	538.2992	1074.5838	1074.5855	-1.58	1	(21)	0.68	2	K.MLTINPSKR.I + Oxidation (M)
145	538.2996	1074.5846	1074.5855	-0.83	1	(7)	16	10	K.MLTINPSKR.I + Oxidation (M)
146	538.3000	1074.5854	1074.5855	-0.09	1	(47)	0.0018	1	K.MLTINPSKR.I + Oxidation (M)
147	538.3003	1074.5860	1074.5855	0.47	1	(23)	0.42	1	K.MLTINPSKR.I + Oxidation (M)
197	574.3211	1146.6276	1146.6431	-13.45	0	(62)	4.1e-005	1	K.GAILTTMLATR.N
198	574.3234	1146.6322	1146.6431	-9.43	0	(38)	0.011	1	K.GAILTTMLATR.N
199	574.3245	1146.6344	1146.6431	-7.52	0	(45)	0.002	1	K.GAILTTMLATR.N
200	574.3247	1146.6348	1146.6431	-7.17	0	(54)	0.00025	1	K.GAILTTMLATR.N
201	574.3251	1146.6356	1146.6431	-6.47	0	(52)	0.00036	1	K.GAILTTMLATR.N
202	574.3251	1146.6356	1146.6431	-6.47	0	(38)	0.011	1	K.GAILTTMLATR.N
203	574.3258	1146.6370	1146.6431	-5.25	0	(64)	2.4e-005	1	K.GAILTTMLATR.N
204	574.3258	1146.6370	1146.6431	-5.25	0	(59)	8.2e-005	1	K.GAILTTMLATR.N
205	574.3262	1146.6378	1146.6431	-4.55	0	(59)	7.9e-005	1	K.GAILTTMLATR.N
206	574.3262	1146.6378	1146.6431	-4.55	0	(43)	0.0032	1	K.GAILTTMLATR.N
207	574.3263	1146.6380	1146.6431	-4.38	0	(57)	0.00014	1	K.GAILTTMLATR.N
208	574.3271	1146.6396	1146.6431	-2.98	0	(48)	0.00088	1	K.GAILTTMLATR.N
209	574.3272	1146.6398	1146.6431	-2.81	0	(46)	0.0013	1	K.GAILTTMLATR.N
210	574.3275	1146.6404	1146.6431	-2.28	0	(41)	0.0049	1	K.GAILTTMLATR.N
211	574.3278	1146.6410	1146.6431	-1.76	0	(58)	8.4e-005	1	K.GAILTTMLATR.N
212	574.3279	1146.6412	1146.6431	-1.59	0	(59)	7.2e-005	1	K.GAILTTMLATR.N
213	574.3279	1146.6412	1146.6431	-1.59	0	(59)	7.1e-005	1	K.GAILTTMLATR.N
214	574.3279	1146.6412	1146.6431	-1.59	0	(59)	7.1e-005	1	K.GAILTTMLATR.N
215	574.3281	1146.6416	1146.6431	-1.24	0	(63)	3e-005	1	K.GAILTTMLATR.N
216	574.3281	1146.6416	1146.6431	-1.24	0	78	9.7e-007	1	K.GAILTTMLATR.N
217	574.3283	1146.6420	1146.6431	-0.89	0	(50)	0.00053	1	K.GAILTTMLATR.N
218	574.3287	1146.6428	1146.6431	-0.19	0	(49)	0.00065	1	K.GAILTTMLATR.N
219	574.3294	1146.6442	1146.6431	1.03	0	(56)	0.00015	1	K.GAILTTMLATR.N
220	574.3297	1146.6448	1146.6431	1.55	0	(31)	0.048	1	K.GAILTTMLATR.N
221	574.3318	1146.6490	1146.6431	5.22	0	(42)	0.0029	1	K.GAILTTMLATR.N
261	631.3007	1260.5868	1260.5927	-4.68	0	(35)	0.02	1	R.FYFENLWSR.N
262	631.3032	1260.5918	1260.5927	-0.71	0	(37)	0.012	1	R.FYFENLWSR.N
263	631.3038	1260.5930	1260.5927	0.24	0	(48)	0.001	1	R.FYFENLWSR.N
264	631.3044	1260.5942	1260.5927	1.19	0	(35)	0.021	1	R.FYFENLWSR.N
265	631.3046	1260.5946	1260.5927	1.51	0	(51)	0.00059	1	R.FYFENLWSR.N
266	631.3048	1260.5950	1260.5927	1.83	0	(48)	0.0011	1	R.FYFENLWSR.N
267	631.3051	1260.5956	1260.5927	2.30	0	(38)	0.0099	1	R.FYFENLWSR.N
268	631.3055	1260.5964	1260.5927	2.94	0	(48)	0.0011	1	R.FYFENLWSR.N
269	631.3056	1260.5966	1260.5927	3.10	0	(51)	0.0006	1	R.FYFENLWSR.N
270	631.3056	1260.5966	1260.5927	3.10	0	(36)	0.016	1	R.FYFENLWSR.N
271	631.3060	1260.5974	1260.5927	3.73	0	(51)	0.0006	1	R.FYFENLWSR.N
272	631.3065	1260.5984	1260.5927	4.53	0	51	0.00061	1	R.FYFENLWSR.N
292	652.3373	1302.6600	1302.6932	-25.44	0	(54)	0.00031	1	R.ITQYLDAGGIPR.T
293	652.3430	1302.6714	1302.6932	-16.69	0	(75)	2.6e-006	1	R.ITQYLDAGGIPR.T
294	652.3467	1302.6788	1302.6932	-11.01	0	(73)	4e-006	1	R.ITQYLDAGGIPR.T
295	652.3514	1302.6882	1302.6932	-3.80	0	(59)	0.0001	1	R.ITQYLDAGGIPR.T
296	652.3551	1302.6956	1302.6932	1.89	0	(77)	1.4e-006	1	R.ITQYLDAGGIPR.T
297	652.3551	1302.6956	1302.6932	1.89	0	79	9.7e-007	1	R.ITQYLDAGGIPR.T
298	652.3563	1302.6980	1302.6932	3.73	0	(52)	0.0004	1	R.ITQYLDAGGIPR.T
308	670.8843	1339.7540	1339.7711	-12.71	0	(4)	18	1	R.DLKPENLLLASK.L
309	670.8865	1339.7584	1339.7711	-9.42	0	(20)	0.47	1	R.DLKPENLLLASK.L
310	670.8892	1339.7638	1339.7711	-5.39	0	(27)	0.088	1	R.DLKPENLLLASK.L
311	670.8903	1339.7660	1339.7711	-3.75	0	(52)	0.00028	1	R.DLKPENLLLASK.L
312	670.8908	1339.7670	1339.7711	-3.00	0	(33)	0.018	1	R.DLKPENLLLASK.L
313	670.8910	1339.7674	1339.7711	-2.70	0	(35)	0.012	1	R.DLKPENLLLASK.L
314	670.8913	1339.7680	1339.7711	-2.26	0	(53)	0.00019	1	R.DLKPENLLLASK.L
315	670.8915	1339.7684	1339.7711	-1.96	0	(60)	3.6e-005	1	R.DLKPENLLLASK.L
316	670.8926	1339.7706	1339.7711	-0.32	0	(58)	6.1e-005	1	R.DLKPENLLLASK.L

317	670.8928	1339.7710	1339.7711	-0.02	0	(23)	0.17	1	R.DLKPENLLLASK.L
318	670.8931	1339.7716	1339.7711	0.43	0	(37)	0.0077	1	R.DLKPENLLLASK.L
319	670.8934	1339.7722	1339.7711	0.88	0	(61)	3e-005	1	R.DLKPENLLLASK.L
320	670.8937	1339.7728	1339.7711	1.33	0	(66)	8.4e-006	1	R.DLKPENLLLASK.L
321	670.8940	1339.7734	1339.7711	1.77	0	82	2.1e-007	1	R.DLKPENLLLASK.L
322	670.8940	1339.7734	1339.7711	1.77	0	(67)	6.9e-006	1	R.DLKPENLLLASK.L
323	670.8940	1339.7734	1339.7711	1.77	0	(63)	1.7e-005	1	R.DLKPENLLLASK.L
324	670.8945	1339.7744	1339.7711	2.52	0	(51)	0.00028	1	R.DLKPENLLLASK.L
325	670.8946	1339.7746	1339.7711	2.67	0	(70)	3.2e-006	1	R.DLKPENLLLASK.L
326	670.8951	1339.7756	1339.7711	3.42	0	(63)	1.6e-005	1	R.DLKPENLLLASK.L
327	670.8954	1339.7762	1339.7711	3.86	0	(63)	1.6e-005	1	R.DLKPENLLLASK.L
328	670.8955	1339.7764	1339.7711	4.01	0	(59)	4.4e-005	1	R.DLKPENLLLASK.L
398	816.8909	1631.7672	1631.7719	-2.84	0	(62)	4.1e-005	1	R.FTEEYQLFEELGK.G
399	816.8932	1631.7718	1631.7719	-0.02	0	(54)	0.00023	1	R.FTEEYQLFEELGK.G
400	816.8936	1631.7726	1631.7719	0.47	0	(60)	6.7e-005	1	R.FTEEYQLFEELGK.G
401	816.8937	1631.7728	1631.7719	0.59	0	(59)	8.6e-005	1	R.FTEEYQLFEELGK.G
402	816.8939	1631.7732	1631.7719	0.84	0	(54)	0.00024	1	R.FTEEYQLFEELGK.G
403	816.8940	1631.7734	1631.7719	0.96	0	(64)	2.3e-005	1	R.FTEEYQLFEELGK.G
404	816.8947	1631.7748	1631.7719	1.82	0	(64)	2.6e-005	1	R.FTEEYQLFEELGK.G
405	816.8948	1631.7750	1631.7719	1.94	0	(64)	2.4e-005	1	R.FTEEYQLFEELGK.G
406	816.8949	1631.7752	1631.7719	2.06	0	70	6.7e-006	1	R.FTEEYQLFEELGK.G
407	816.8950	1631.7754	1631.7719	2.19	0	(58)	0.00011	1	R.FTEEYQLFEELGK.G
408	816.8951	1631.7756	1631.7719	2.31	0	(66)	1.7e-005	1	R.FTEEYQLFEELGK.G
409	816.8952	1631.7758	1631.7719	2.43	0	(63)	3e-005	1	R.FTEEYQLFEELGK.G
410	816.8953	1631.7760	1631.7719	2.55	0	(59)	7.2e-005	1	R.FTEEYQLFEELGK.G
411	816.8957	1631.7768	1631.7719	3.04	0	(64)	2.6e-005	1	R.FTEEYQLFEELGK.G
412	816.8958	1631.7770	1631.7719	3.17	0	(61)	4.7e-005	1	R.FTEEYQLFEELGK.G
413	816.8960	1631.7774	1631.7719	3.41	0	(61)	4.9e-005	1	R.FTEEYQLFEELGK.G
414	816.8961	1631.7776	1631.7719	3.53	0	(53)	0.00032	1	R.FTEEYQLFEELGK.G
415	816.8961	1631.7776	1631.7719	3.53	0	(56)	0.00017	1	R.FTEEYQLFEELGK.G
416	816.8962	1631.7778	1631.7719	3.66	0	(67)	1.4e-005	1	R.FTEEYQLFEELGK.G
417	816.8962	1631.7778	1631.7719	3.66	0	(60)	6.2e-005	1	R.FTEEYQLFEELGK.G
418	816.8970	1631.7794	1631.7719	4.64	0	(57)	0.00013	1	R.FTEEYQLFEELGK.G
419	816.8971	1631.7796	1631.7719	4.76	0	(26)	0.15	1	R.FTEEYQLFEELGK.G
420	816.8973	1631.7800	1631.7719	5.00	0	(51)	0.0005	1	R.FTEEYQLFEELGK.G
421	816.8973	1631.7800	1631.7719	5.00	0	(68)	1.1e-005	1	R.FTEEYQLFEELGK.G
422	816.8975	1631.7804	1631.7719	5.25	0	(62)	4e-005	1	R.FTEEYQLFEELGK.G
423	816.8977	1631.7808	1631.7719	5.50	0	(61)	5.2e-005	1	R.FTEEYQLFEELGK.G
424	816.8977	1631.7808	1631.7719	5.50	0	(55)	0.0002	1	R.FTEEYQLFEELGK.G
425	816.8979	1631.7812	1631.7719	5.74	0	(57)	0.00013	1	R.FTEEYQLFEELGK.G
426	816.8990	1631.7834	1631.7719	7.09	0	(54)	0.00024	1	R.FTEEYQLFEELGK.G
477	680.9728	2039.8966	2039.9131	-8.10	1	103	1.4e-009	1	K.ESSESTNTTIEDEDTKVR.K
478	680.9773	2039.9101	2039.9131	-1.48	1	(102)	2.3e-009	1	K.ESSESTNTTIEDEDTKVR.K
483	1040.9476	2079.8806	2079.9426	-29.76	0	(74)	8.8e-007	1	K.AGAYDFPSPEWDTVTPEAK.D
484	1040.9493	2079.8840	2079.9426	-28.13	0	(58)	3.7e-005	1	K.AGAYDFPSPEWDTVTPEAK.D
485	1040.9618	2079.9090	2079.9426	-16.11	0	(57)	5.8e-005	1	K.AGAYDFPSPEWDTVTPEAK.D
486	694.3159	2079.9259	2079.9426	-8.02	0	(70)	3.7e-006	1	K.AGAYDFPSPEWDTVTPEAK.D
487	694.3180	2079.9322	2079.9426	-4.99	0	90	3.7e-008	1	K.AGAYDFPSPEWDTVTPEAK.D
488	1040.9735	2079.9324	2079.9426	-4.86	0	(42)	0.0023	1	K.AGAYDFPSPEWDTVTPEAK.D
489	1040.9767	2079.9388	2079.9426	-1.78	0	(64)	1.7e-005	1	K.AGAYDFPSPEWDTVTPEAK.D
490	1040.9814	2079.9482	2079.9426	2.74	0	(62)	2.8e-005	1	K.AGAYDFPSPEWDTVTPEAK.D
491	1040.9825	2079.9504	2079.9426	3.80	0	(75)	1.4e-006	1	K.AGAYDFPSPEWDTVTPEAK.D
498	715.3460	2143.0162	2143.0321	-7.42	0	(71)	4.1e-006	1	K.VTEQLIEAISNGDFESYTK.M
499	715.3465	2143.0177	2143.0321	-6.72	0	102	3.4e-009	1	K.VTEQLIEAISNGDFESYTK.M
500	1072.5264	2143.0382	2143.0321	2.88	0	(93)	2.6e-008	1	K.VTEQLIEAISNGDFESYTK.M
501	1072.5287	2143.0428	2143.0321	5.03	0	(77)	1e-006	1	K.VTEQLIEAISNGDFESYTK.M
558	888.7586	2663.2540	2663.2755	-8.08	1	(47)	0.00081	1	K.AGAYDFPSPEWDTVTPEAKDLINK.M
559	888.7591	2663.2555	2663.2755	-7.52	1	(71)	3.1e-006	1	K.AGAYDFPSPEWDTVTPEAKDLINK.M
560	888.7594	2663.2564	2663.2755	-7.18	1	(90)	4e-008	1	K.AGAYDFPSPEWDTVTPEAKDLINK.M
561	888.7601	2663.2585	2663.2755	-6.39	1	(59)	4.9e-005	1	K.AGAYDFPSPEWDTVTPEAKDLINK.M
562	888.7601	2663.2585	2663.2755	-6.39	1	97	8.5e-009	1	K.AGAYDFPSPEWDTVTPEAKDLINK.M
563	888.7603	2663.2591	2663.2755	-6.17	1	(78)	7e-007	1	K.AGAYDFPSPEWDTVTPEAKDLINK.M
564	888.7603	2663.2591	2663.2755	-6.17	1	(79)	5.1e-007	1	K.AGAYDFPSPEWDTVTPEAKDLINK.M
565	888.7603	2663.2591	2663.2755	-6.17	1	(78)	7e-007	1	K.AGAYDFPSPEWDTVTPEAKDLINK.M
566	888.7608	2663.2606	2663.2755	-5.60	1	(74)	1.5e-006	1	K.AGAYDFPSPEWDTVTPEAKDLINK.M
567	888.7608	2663.2606	2663.2755	-5.60	1	(52)	0.00027	1	K.AGAYDFPSPEWDTVTPEAKDLINK.M
568	888.7616	2663.2630	2663.2755	-4.70	1	(32)	0.03	1	K.AGAYDFPSPEWDTVTPEAKDLINK.M
569	888.7617	2663.2633	2663.2755	-4.59	1	(26)	0.11	1	K.AGAYDFPSPEWDTVTPEAKDLINK.M
570	888.7621	2663.2645	2663.2755	-4.14	1	(79)	4.8e-007	1	K.AGAYDFPSPEWDTVTPEAKDLINK.M
571	888.7621	2663.2645	2663.2755	-4.14	1	(67)	7.6e-006	1	K.AGAYDFPSPEWDTVTPEAKDLINK.M
572	888.7629	2663.2669	2663.2755	-3.24	1	(78)	6e-007	1	K.AGAYDFPSPEWDTVTPEAKDLINK.M
573	888.7650	2663.2732	2663.2755	-0.87	1	(12)	2.6	1	K.AGAYDFPSPEWDTVTPEAKDLINK.M

2. IPI00474502 **Mass:** 61164 **Score:** 2503 **Queries matched:** 106 **emPAI:** 1.85
Tax_Id=10090 Gene_Symbol=Camk2b Calcium/calmodulin-dependent protein kinase type II subunit beta
Check to include this hit in error tolerant search or archive report

Query	Observed	Mr(expt)	Mr(calc)	ppm	Miss	Score	Expect	Rank	Peptide
5	445.2779	444.2706	444.2696	2.25	0	(7)	29	1	K.GAAVK.L
6	445.2791	444.2718	444.2696	4.95	0	8	27	1	K.GAAVK.L
71	452.7382	903.4618	903.4661	-4.75	0	51	0.0009	1	K.NSSAITSPK.G

☑										
☑	188	**572.8056**	**1143.5966**	**1143.5964**	**0.20**	**0**	**21**	**0.77**	**1**	**R.FYFENLLAK.N**
☑	189	**572.8057**	**1143.5968**	**1143.5964**	**0.37**	**0**	**(19)**	**1.1**	**1**	**R.FYFENLLAK.N**
☑	190	**572.8067**	**1143.5988**	**1143.5964**	**2.12**	**0**	**(11)**	**6.9**	**1**	**R.FYFENLLAK.N**
☑	191	**572.8068**	**1143.5990**	**1143.5964**	**2.30**	**0**	**(19)**	**1.1**	**1**	**R.FYFENLLAK.N**
☑	193	**572.8070**	**1143.5994**	**1143.5964**	**2.65**	**0**	**(7)**	**18**	**1**	**R.FYFENLLAK.N**
☑	194	**572.8076**	**1143.6006**	**1143.5964**	**3.70**	**0**	**(15)**	**2.5**	**1**	**R.FYFENLLAK.N**
	195	**572.8080**	**1143.6014**	**1143.5964**	**4.40**	**0**	**(4)**	**32**	**3**	**R.FYFENLLAK.N**
	197	574.3211	1146.6276	1146.6431	-13.45	0	(62)	4.1e-005	1	K.GAILTTMLATR.N
	198	574.3234	1146.6322	1146.6431	-9.43	0	(38)	0.011	1	K.GAILTTMLATR.N
	199	574.3245	1146.6344	1146.6431	-7.52	0	(45)	0.002	1	K.GAILTTMLATR.N
	200	574.3247	1146.6348	1146.6431	-7.17	0	(54)	0.00025	1	K.GAILTTMLATR.N
	201	574.3251	1146.6356	1146.6431	-6.47	0	(52)	0.00036	1	K.GAILTTMLATR.N
	202	574.3251	1146.6356	1146.6431	-6.47	0	(38)	0.011	1	K.GAILTTMLATR.N
	203	574.3258	1146.6370	1146.6431	-5.25	0	(64)	2.4e-005	1	K.GAILTTMLATR.N
	204	574.3258	1146.6370	1146.6431	-5.25	0	(59)	8.2e-005	1	K.GAILTTMLATR.N
	205	574.3262	1146.6378	1146.6431	-4.55	0	(59)	7.9e-005	1	K.GAILTTMLATR.N
	206	574.3262	1146.6378	1146.6431	-4.55	0	(43)	0.0032	1	K.GAILTTMLATR.N
	207	574.3263	1146.6380	1146.6431	-4.38	0	(57)	0.00014	1	K.GAILTTMLATR.N
	208	574.3271	1146.6396	1146.6431	-2.98	0	(48)	0.00088	1	K.GAILTTMLATR.N
	209	574.3272	1146.6398	1146.6431	-2.81	0	(46)	0.0013	1	K.GAILTTMLATR.N
	210	574.3275	1146.6404	1146.6431	-2.28	0	(41)	0.0049	1	K.GAILTTMLATR.N
	211	574.3278	1146.6410	1146.6431	-1.76	0	(58)	8.4e-005	1	K.GAILTTMLATR.N
	212	574.3279	1146.6412	1146.6431	-1.59	0	(59)	7.2e-005	1	K.GAILTTMLATR.N
	213	574.3279	1146.6412	1146.6431	-1.59	0	(59)	7.1e-005	1	K.GAILTTMLATR.N
	214	574.3279	1146.6412	1146.6431	-1.59	0	(59)	7.1e-005	1	K.GAILTTMLATR.N
	215	574.3281	1146.6416	1146.6431	-1.24	0	(63)	3e-005	1	K.GAILTTMLATR.N
	216	574.3281	1146.6416	1146.6431	-1.24	0	78	9.7e-007	1	K.GAILTTMLATR.N
	217	574.3283	1146.6420	1146.6431	-0.89	0	(50)	0.00053	1	K.GAILTTMLATR.N
	218	574.3287	1146.6428	1146.6431	-0.19	0	(49)	0.00065	1	K.GAILTTMLATR.N
	219	574.3294	1146.6442	1146.6431	1.03	0	(56)	0.00015	1	K.GAILTTMLATR.N
	220	574.3297	1146.6448	1146.6431	1.55	0	(31)	0.048	1	K.GAILTTMLATR.N
	221	574.3318	1146.6490	1146.6431	5.22	0	(42)	0.0029	1	K.GAILTTMLATR.N
	308	670.8843	1339.7540	1339.7711	-12.71	0	(4)	18	1	R.DLKPENLLLASK.C
	309	670.8865	1339.7584	1339.7711	-9.42	0	(20)	0.47	1	R.DLKPENLLLASK.C
	310	670.8892	1339.7638	1339.7711	-5.39	0	(27)	0.088	1	R.DLKPENLLLASK.C
	311	670.8903	1339.7660	1339.7711	-3.75	0	(52)	0.00028	1	R.DLKPENLLLASK.C
	312	670.8908	1339.7670	1339.7711	-3.00	0	(33)	0.018	1	R.DLKPENLLLASK.C
	313	670.8910	1339.7674	1339.7711	-2.70	0	(35)	0.012	1	R.DLKPENLLLASK.C
	314	670.8913	1339.7680	1339.7711	-2.26	0	(53)	0.00019	1	R.DLKPENLLLASK.C
	315	670.8915	1339.7684	1339.7711	-1.96	0	(60)	3.6e-005	1	R.DLKPENLLLASK.C
	316	670.8926	1339.7706	1339.7711	-0.32	0	(58)	6.1e-005	1	R.DLKPENLLLASK.C
	317	670.8928	1339.7710	1339.7711	-0.02	0	(23)	0.17	1	R.DLKPENLLLASK.C
	318	670.8931	1339.7716	1339.7711	0.43	0	(37)	0.0077	1	R.DLKPENLLLASK.C
	319	670.8934	1339.7722	1339.7711	0.88	0	(61)	3e-005	1	R.DLKPENLLLASK.C
	320	670.8937	1339.7728	1339.7711	1.33	0	(66)	8.4e-006	1	R.DLKPENLLLASK.C
	321	670.8940	1339.7734	1339.7711	1.77	0	82	2.1e-007	1	R.DLKPENLLLASK.C
	322	670.8940	1339.7734	1339.7711	1.77	0	(67)	6.9e-006	1	R.DLKPENLLLASK.C
	323	670.8940	1339.7734	1339.7711	1.77	0	(63)	1.7e-005	1	R.DLKPENLLLASK.C
	324	670.8945	1339.7744	1339.7711	2.52	0	(51)	0.00028	1	R.DLKPENLLLASK.C
	325	670.8946	1339.7746	1339.7711	2.67	0	(70)	3.2e-006	1	R.DLKPENLLLASK.C
	326	670.8951	1339.7756	1339.7711	3.42	0	(63)	1.6e-005	1	R.DLKPENLLLASK.C
	327	670.8954	1339.7762	1339.7711	3.86	0	(63)	1.6e-005	1	R.DLKPENLLLASK.C
	328	670.8955	1339.7764	1339.7711	4.01	0	(59)	4.4e-005	1	R.DLKPENLLLASK.C
☑	346	**468.5799**	**1402.7179**	**1402.7317**	**-9.86**	**0**	**(50)**	**0.00069**	**1**	**R.LTQYIDGQGRPR.T**
☑	347	**468.5800**	**1402.7182**	**1402.7317**	**-9.65**	**0**	**(51)**	**0.00053**	**1**	**R.LTQYIDGQGRPR.T**
☑	348	**468.5803**	**1402.7191**	**1402.7317**	**-9.01**	**0**	**(52)**	**0.00046**	**1**	**R.LTQYIDGQGRPR.T**
☑	349	**468.5805**	**1402.7197**	**1402.7317**	**-8.58**	**0**	**(38)**	**0.011**	**1**	**R.LTQYIDGQGRPR.T**
☑	350	**468.5811**	**1402.7215**	**1402.7317**	**-7.30**	**0**	**59**	**8.4e-005**	**1**	**R.LTQYIDGQGRPR.T**
☑	351	**702.3745**	**1402.7344**	**1402.7317**	**1.96**	**0**	**(53)**	**0.00035**	**1**	**R.LTQYIDGQGRPR.T**
☑	352	**702.3751**	**1402.7356**	**1402.7317**	**2.81**	**0**	**(39)**	**0.0085**	**1**	**R.LTQYIDGQGRPR.T**
☑	353	**702.3760**	**1402.7374**	**1402.7317**	**4.09**	**0**	**(43)**	**0.0035**	**1**	**R.LTQYIDGQGRPR.T**
☑	354	**702.3771**	**1402.7396**	**1402.7317**	**5.66**	**0**	**(18)**	**0.99**	**1**	**R.LTQYIDGQGRPR.T**
☑	355	**702.3782**	**1402.7418**	**1402.7317**	**7.23**	**0**	**(26)**	**0.17**	**1**	**R.LTQYIDGQGRPR.T**
☑	360	**735.4061**	**1468.7976**	**1468.8071**	**-6.47**	**0**	**75**	**1.8e-006**	**1**	**K.NLINQMLTINPAK.R**
☑	361	**735.4121**	**1468.8096**	**1468.8071**	**1.70**	**0**	**(71)**	**4.1e-006**	**1**	**K.NLINQMLTINPAK.R**
☑	362	**735.4124**	**1468.8102**	**1468.8071**	**2.11**	**0**	**(41)**	**0.0041**	**1**	**K.NLINQMLTINPAK.R**
☑	363	**735.4127**	**1468.8108**	**1468.8071**	**2.52**	**0**	**(70)**	**4.3e-006**	**1**	**K.NLINQMLTINPAK.R**
☑	364	**735.4138**	**1468.8130**	**1468.8071**	**4.02**	**0**	**(71)**	**3.5e-006**	**1**	**K.NLINQMLTINPAK.R**
☑	394	**810.8770**	**1619.7394**	**1619.7355**	**2.43**	**0**	**70**	**5.6e-006**	**1**	**R.FTDEYQLYEDIGK.G**
☑	395	**810.8795**	**1619.7444**	**1619.7355**	**5.52**	**0**	**(65)**	**1.8e-005**	**1**	**R.FTDEYQLYEDIGK.G**
☑	396	**810.8807**	**1619.7468**	**1619.7355**	**7.00**	**0**	**(51)**	**0.00047**	**1**	**R.FTDEYQLYEDIGK.G**
☑	397	**542.6334**	**1624.8784**	**1624.9082**	**-18.39**	**1**	**45**	**0.0014**	**1**	**K.NLINQMLTINPAKR.I**
☑	443	**871.3691**	**1740.7236**	**1740.7173**	**3.62**	**0**	**90**	**2.1e-008**	**1**	**K.ESSDSTNTTIEDEDAK.A**
☑	475	**656.9542**	**1967.8408**	**1967.8556**	**-7.51**	**1**	**94**	**8.8e-009**	**1**	**K.ESSDSTNTTIEDEDAKAR.K**
	483	1040.9476	2079.8806	2079.9426	-29.76	0	(74)	8.8e-007	1	K.AGAYDFPSPEWDTVTPEAK.N
	484	1040.9493	2079.8840	2079.9426	-28.13	0	(58)	3.7e-005	1	K.AGAYDFPSPEWDTVTPEAK.N
	485	1040.9618	2079.9090	2079.9426	-16.11	0	(57)	5.8e-005	1	K.AGAYDFPSPEWDTVTPEAK.N
	486	694.3159	2079.9259	2079.9426	-8.02	0	(70)	3.7e-006	1	K.AGAYDFPSPEWDTVTPEAK.N
	487	694.3180	2079.9322	2079.9426	-4.99	0	90	3.7e-008	1	K.AGAYDFPSPEWDTVTPEAK.N
	488	1040.9735	2079.9324	2079.9426	-4.86	0	(42)	0.0023	1	K.AGAYDFPSPEWDTVTPEAK.N
	489	1040.9767	2079.9388	2079.9426	-1.78	0	(64)	1.7e-005	1	K.AGAYDFPSPEWDTVTPEAK.N
	490	1040.9814	2079.9482	2079.9426	2.74	0	(62)	2.8e-005	1	K.AGAYDFPSPEWDTVTPEAK.N
	491	1040.9825	2079.9504	2079.9426	3.80	0	(75)	1.4e-006	1	K.AGAYDFPSPEWDTVTPEAK.N
☑	494	**705.0025**	**2111.9857**	**2112.0011**	**-7.30**	**0**	**(66)**	**1.3e-005**	**1**	**K.TTEQLIEAVNNGDFEAYAK.I**
☑	495	**705.0052**	**2111.9938**	**2112.0011**	**-3.47**	**0**	**(65)**	**1.5e-005**	**1**	**K.TTEQLIEAVNNGDFEAYAK.I**
☑	496	**1057.0114**	**2112.0082**	**2112.0011**	**3.38**	**0**	**94**	**1.8e-008**	**1**	**K.TTEQLIEAVNNGDFEAYAK.I**

☑	540	779.7122	2336.1148	2336.1325	-7.58	1	(13)	2.4	1	R.FTDEYQLYEDIGKGAFSVVR.R
☑	541	779.7162	2336.1268	2336.1325	-2.44	1	73	2.3e-006	1	R.FTDEYQLYEDIGKGAFSVVR.R
☑	542	785.0447	2352.1123	2352.1301	-7.58	0	(87)	8.1e-008	1	R.QTTAPATMSTAASGTTMGLVEQAK.S
☑	543	785.0452	2352.1138	2352.1301	-6.95	0	(108)	6.7e-010	1	R.QTTAPATMSTAASGTTMGLVEQAK.S
☑	544	1177.0774	2352.1402	2352.1301	4.31	0	(60)	4.2e-005	1	R.QTTAPATMSTAASGTTMGLVEQAK.S
☑	545	1177.0776	2352.1406	2352.1301	4.48	0	(71)	3.7e-006	1	R.QTTAPATMSTAASGTTMGLVEQAK.S
☑	546	1177.0782	2352.1418	2352.1301	4.99	0	130	4.7e-012	1	R.QTTAPATMSTAASGTTMGLVEQAK.S
☑	547	785.4210	2353.2412	2353.2642	-9.77	0	(34)	0.015	1	K.GSLPPAALEPQTTVIHNPVDGIK.E
☑	548	785.4226	2353.2460	2353.2642	-7.73	0	89	4.7e-008	1	K.GSLPPAALEPQTTVIHNPVDGIK.E
☑	549	785.4229	2353.2469	2353.2642	-7.35	0	(70)	3.4e-006	1	K.GSLPPAALEPQTTVIHNPVDGIK.E
☑	550	785.4230	2353.2472	2353.2642	-7.22	0	(82)	2.1e-007	1	K.GSLPPAALEPQTTVIHNPVDGIK.E
☑	551	785.4232	2353.2478	2353.2642	-6.96	0	(80)	3.1e-007	1	K.GSLPPAALEPQTTVIHNPVDGIK.E
☑	552	785.4244	2353.2514	2353.2642	-5.43	0	(71)	2.8e-006	1	K.GSLPPAALEPQTTVIHNPVDGIK.E
☑	553	790.3778	2368.1116	2368.1250	-5.68	0	(88)	6.5e-008	1	R.QTTAPATMSTAASGTTMGLVEQAK.S + Oxidation (M)
☑	554	790.3788	2368.1146	2368.1250	-4.42	0	(111)	3.2e-010	1	R.QTTAPATMSTAASGTTMGLVEQAK.S + Oxidation (M)
☑	555	1185.0740	2368.1334	2368.1250	3.55	0	(88)	6.7e-008	1	R.QTTAPATMSTAASGTTMGLVEQAK.S + Oxidation (M)
☑	556	1185.0802	2368.1458	2368.1250	8.79	0	(72)	2.9e-006	1	R.QTTAPATMSTAASGTTMGLVEQAK.S + Oxidation (M)

Proteins matching the same set of peptides:
IPI00875723 **Mass:** 68686 **Score:** 2503 **Queries matched:** 106
Tax_Id=10090 Gene_Symbol=Camk2b 68 kDa protein

3. IPI00406790 **Mass:** 60593 **Score:** 1774 **Queries matched:** 91 **emPAI:** 0.70
Tax_Id=10090 Gene_Symbol=Camk2d Isoform 4 of Calcium/calmodulin-dependent protein kinase type II subunit delta
☐ Check to include this hit in error tolerant search or archive report

	Query	Observed	Mr(expt)	Mr(calc)	ppm	Miss	Score	Expect	Rank	Peptide
	5	445.2779	444.2706	444.2696	2.25	0	(7)	29	1	K.GAAVK.L
	6	445.2791	444.2718	444.2696	4.95	0	8	27	1	K.GAAVK.L
	11	602.3611	601.3538	601.3435	17.2	0	12	12	1	K.DLINK.M
☑	149	539.2814	1076.5482	1076.5502	-1.81	0	44	0.0034	1	K.IPTGQEYAAK.I
	161	556.8326	1111.6506	1111.6349	14.1	1	(3)	16	6	K.KPDGVKINNK.A
	163	556.8347	1111.6548	1111.6349	17.9	1	(1)	24	7	K.KPDGVKINNK.A
	166	556.8363	1111.6580	1111.6349	20.8	1	(2)	21	3	K.KPDGVKINNK.A
☑	167	556.8364	1111.6582	1111.6349	21.0	1	5	9.5	1	K.KPDGVKINNK.A
	168	556.8366	1111.6586	1111.6349	21.3	1	(3)	16	2	K.KPDGVKINNK.A
☑	174	559.7805	1117.5464	1117.5444	1.84	0	(22)	0.52	1	R.FYFENALSK.S
☑	175	559.7811	1117.5476	1117.5444	2.92	0	27	0.16	1	R.FYFENALSK.S
☑	176	559.7817	1117.5488	1117.5444	3.99	0	(26)	0.2	1	R.FYFENALSK.S
☑	177	559.7820	1117.5494	1117.5444	4.53	0	(20)	0.76	1	R.FYFENALSK.S
	197	574.3211	1146.6276	1146.6431	-13.45	0	(62)	4.1e-005	1	K.GAILTTMLATR.N
	198	574.3234	1146.6322	1146.6431	-9.43	0	(38)	0.011	1	K.GAILTTMLATR.N
	199	574.3245	1146.6344	1146.6431	-7.52	0	(45)	0.002	1	K.GAILTTMLATR.N
	200	574.3247	1146.6348	1146.6431	-7.17	0	(54)	0.00025	1	K.GAILTTMLATR.N
	201	574.3251	1146.6356	1146.6431	-6.47	0	(52)	0.00036	1	K.GAILTTMLATR.N
	202	574.3251	1146.6356	1146.6431	-6.47	0	(38)	0.011	1	K.GAILTTMLATR.N
	203	574.3258	1146.6370	1146.6431	-5.25	0	(64)	2.4e-005	1	K.GAILTTMLATR.N
	204	574.3258	1146.6370	1146.6431	-5.25	0	(59)	8.2e-005	1	K.GAILTTMLATR.N
	205	574.3262	1146.6378	1146.6431	-4.55	0	(59)	7.9e-005	1	K.GAILTTMLATR.N
	206	574.3262	1146.6378	1146.6431	-4.55	0	(43)	0.0032	1	K.GAILTTMLATR.N
	207	574.3263	1146.6380	1146.6431	-4.38	0	(57)	0.00014	1	K.GAILTTMLATR.N
	208	574.3271	1146.6396	1146.6431	-2.98	0	(48)	0.00088	1	K.GAILTTMLATR.N
	209	574.3272	1146.6398	1146.6431	-2.81	0	(46)	0.0013	1	K.GAILTTMLATR.N
	210	574.3275	1146.6404	1146.6431	-2.28	0	(41)	0.0049	1	K.GAILTTMLATR.N
	211	574.3278	1146.6410	1146.6431	-1.76	0	(58)	8.4e-005	1	K.GAILTTMLATR.N
	212	574.3279	1146.6412	1146.6431	-1.59	0	(59)	7.2e-005	1	K.GAILTTMLATR.N
	213	574.3279	1146.6412	1146.6431	-1.59	0	(59)	7.1e-005	1	K.GAILTTMLATR.N
	214	574.3279	1146.6412	1146.6431	-1.59	0	(59)	7.1e-005	1	K.GAILTTMLATR.N
	215	574.3281	1146.6416	1146.6431	-1.24	0	(63)	3e-005	1	K.GAILTTMLATR.N
	216	574.3281	1146.6416	1146.6431	-1.24	0	78	9.7e-007	1	K.GAILTTMLATR.N
	217	574.3283	1146.6420	1146.6431	-0.89	0	(50)	0.00053	1	K.GAILTTMLATR.N
	218	574.3287	1146.6428	1146.6431	-0.19	0	(49)	0.00065	1	K.GAILTTMLATR.N
	219	574.3294	1146.6442	1146.6431	1.03	0	(56)	0.00015	1	K.GAILTTMLATR.N
	220	574.3297	1146.6448	1146.6431	1.55	0	(31)	0.048	1	K.GAILTTMLATR.N
	221	574.3318	1146.6490	1146.6431	5.22	0	(42)	0.0029	1	K.GAILTTMLATR.N
☑	303	664.3068	1326.5990	1326.5948	3.20	0	(52)	0.0003	1	R.LTQYMDGSGMPK.T
☑	304	664.3070	1326.5994	1326.5948	3.50	0	(60)	4.5e-005	1	R.LTQYMDGSGMPK.T
☑	305	664.3091	1326.6036	1326.5948	6.67	0	71	3.9e-006	1	R.LTQYMDGSGMPK.T
	308	670.8843	1339.7540	1339.7711	-12.71	0	(4)	18	1	R.DLKPENLLLASK.S
	309	670.8865	1339.7584	1339.7711	-9.42	0	(20)	0.47	1	R.DLKPENLLLASK.S
	310	670.8892	1339.7638	1339.7711	-5.39	0	(27)	0.088	1	R.DLKPENLLLASK.S
	311	670.8903	1339.7660	1339.7711	-3.75	0	(52)	0.00028	1	R.DLKPENLLLASK.S
	312	670.8908	1339.7670	1339.7711	-3.00	0	(33)	0.018	1	R.DLKPENLLLASK.S
	313	670.8910	1339.7674	1339.7711	-2.70	0	(35)	0.012	1	R.DLKPENLLLASK.S
	314	670.8913	1339.7680	1339.7711	-2.26	0	(53)	0.00019	1	R.DLKPENLLLASK.S
	315	670.8915	1339.7684	1339.7711	-1.96	0	(60)	3.6e-005	1	R.DLKPENLLLASK.S
	316	670.8926	1339.7706	1339.7711	-0.32	0	(58)	6.1e-005	1	R.DLKPENLLLASK.S
	317	670.8928	1339.7710	1339.7711	-0.02	0	(23)	0.17	1	R.DLKPENLLLASK.S
	318	670.8931	1339.7716	1339.7711	0.43	0	(37)	0.0077	1	R.DLKPENLLLASK.S
	319	670.8934	1339.7722	1339.7711	0.88	0	(61)	3e-005	1	R.DLKPENLLLASK.S
	320	670.8937	1339.7728	1339.7711	1.33	0	(66)	8.4e-006	1	R.DLKPENLLLASK.S
	321	670.8940	1339.7734	1339.7711	1.77	0	82	2.1e-007	1	R.DLKPENLLLASK.S
	322	670.8940	1339.7734	1339.7711	1.77	0	(67)	6.9e-006	1	R.DLKPENLLLASK.S
	323	670.8940	1339.7734	1339.7711	1.77	0	(63)	1.7e-005	1	R.DLKPENLLLASK.S
	324	670.8945	1339.7744	1339.7711	2.52	0	(51)	0.00028	1	R.DLKPENLLLASK.S
	325	670.8946	1339.7746	1339.7711	2.67	0	(70)	3.2e-006	1	R.DLKPENLLLASK.S

	326	670.8951	1339.7756	1339.7711	3.42	0	(63)	1.6e-005	1	R.DLKPENLLLASK.S
	327	670.8954	1339.7762	1339.7711	3.86	0	(63)	1.6e-005	1	R.DLKPENLLLASK.S
	328	670.8955	1339.7764	1339.7711	4.01	0	(59)	4.4e-005	1	R.DLKPENLLLASK.S
☑	329	**672.3059**	**1342.5972**	**1342.5897**	**5.61**	**0**	**(54)**	**0.00014**	**1**	**R.LTQYMDGSGMPK.T + Oxidation (M)**
☑	390	**809.8870**	**1617.7594**	**1617.7562**	**1.98**	**0**	**(55)**	**0.00018**	**1**	**R.FTDEYQLFEELGK.G**
☑	391	**809.8891**	**1617.7636**	**1617.7562**	**4.58**	**0**	**66**	**1.5e-005**	**1**	**R.FTDEYQLFEELGK.G**
☑	392	**809.8947**	**1617.7748**	**1617.7562**	**11.5**	**0**	**(60)**	**6.7e-005**	**1**	**R.FTDEYQLFEELGK.G**
	483	1040.9476	2079.8806	2079.9426	-29.76	0	(74)	8.8e-007	1	K.AGAYDFPSPEWDTVTPEAK.D
	484	1040.9493	2079.8840	2079.9426	-28.13	0	(58)	3.7e-005	1	K.AGAYDFPSPEWDTVTPEAK.D
	485	1040.9618	2079.9090	2079.9426	-16.11	0	(57)	5.8e-005	1	K.AGAYDFPSPEWDTVTPEAK.D
	486	694.3159	2079.9259	2079.9426	-8.02	0	(70)	3.7e-006	1	K.AGAYDFPSPEWDTVTPEAK.D
	487	694.3180	2079.9322	2079.9426	-4.99	0	90	3.7e-008	1	K.AGAYDFPSPEWDTVTPEAK.D
	488	1040.9735	2079.9324	2079.9426	-4.86	0	(42)	0.0023	1	K.AGAYDFPSPEWDTVTPEAK.D
	489	1040.9767	2079.9388	2079.9426	-1.78	0	(64)	1.7e-005	1	K.AGAYDFPSPEWDTVTPEAK.D
	490	1040.9814	2079.9482	2079.9426	2.74	0	(62)	2.8e-005	1	K.AGAYDFPSPEWDTVTPEAK.D
	491	1040.9825	2079.9504	2079.9426	3.80	0	(75)	1.4e-006	1	K.AGAYDFPSPEWDTVTPEAK.D
	558	888.7586	2663.2540	2663.2755	-8.08	1	(47)	0.00081	1	K.AGAYDFPSPEWDTVTPEAKDLINK.M
	559	888.7591	2663.2555	2663.2755	-7.52	1	(71)	3.1e-006	1	K.AGAYDFPSPEWDTVTPEAKDLINK.M
	560	888.7594	2663.2564	2663.2755	-7.18	1	(90)	4e-008	1	K.AGAYDFPSPEWDTVTPEAKDLINK.M
	561	888.7601	2663.2585	2663.2755	-6.39	1	(59)	4.9e-005	1	K.AGAYDFPSPEWDTVTPEAKDLINK.M
	562	888.7601	2663.2585	2663.2755	-6.39	1	97	8.5e-009	1	K.AGAYDFPSPEWDTVTPEAKDLINK.M
	563	888.7603	2663.2591	2663.2755	-6.17	1	(78)	7e-007	1	K.AGAYDFPSPEWDTVTPEAKDLINK.M
	564	888.7603	2663.2591	2663.2755	-6.17	1	(79)	5.1e-007	1	K.AGAYDFPSPEWDTVTPEAKDLINK.M
	565	888.7603	2663.2591	2663.2755	-6.17	1	(78)	7e-007	1	K.AGAYDFPSPEWDTVTPEAKDLINK.M
	566	888.7608	2663.2606	2663.2755	-5.60	1	(74)	1.5e-006	1	K.AGAYDFPSPEWDTVTPEAKDLINK.M
	567	888.7608	2663.2606	2663.2755	-5.60	1	(52)	0.00027	1	K.AGAYDFPSPEWDTVTPEAKDLINK.M
	568	888.7616	2663.2630	2663.2755	-4.70	1	(32)	0.03	1	K.AGAYDFPSPEWDTVTPEAKDLINK.M
	569	888.7617	2663.2633	2663.2755	-4.59	1	(26)	0.11	1	K.AGAYDFPSPEWDTVTPEAKDLINK.M
	570	888.7621	2663.2645	2663.2755	-4.14	1	(79)	4.8e-007	1	K.AGAYDFPSPEWDTVTPEAKDLINK.M
	571	888.7621	2663.2645	2663.2755	-4.14	1	(67)	7.6e-006	1	K.AGAYDFPSPEWDTVTPEAKDLINK.M
	572	888.7629	2663.2669	2663.2755	-3.24	1	(78)	6e-007	1	K.AGAYDFPSPEWDTVTPEAKDLINK.M
	573	888.7650	2663.2732	2663.2755	-0.87	1	(12)	2.6	1	K.AGAYDFPSPEWDTVTPEAKDLINK.M

Proteins matching the same set of peptides:
IPI00858128 **Mass:** 58283 **Score:** 1774 **Queries matched:** 91
Tax_Id=10090 Gene_Symbol=Camk2d calcium/calmodulin-dependent protein kinase type II subunit delta isoform 1

---

4. IPI00124695 **Mass:** 60254 **Score:** 1126 **Queries matched:** 53 **emPAI:** 0.61
Tax_Id=10090 Gene_Symbol=Camk2g Isoform 1 of Calcium/calmodulin-dependent protein kinase type II subunit gamma
☐ Check to include this hit in error tolerant search or archive report

	Query	Observed	Mr(expt)	Mr(calc)	ppm	Miss	Score	Expect	Rank	Peptide
	5	445.2779	444.2706	444.2696	2.25	0	(7)	29	1	K.GAAVK.L
	6	445.2791	444.2718	444.2696	4.95	0	8	27	1	K.GAAVK.L
☑	230	**580.8067**	**1159.5988**	**1159.5913**	**6.48**	**0**	**22**	**0.52**	**1**	**K.FYFENLLSK.N**
☑	231	**580.8068**	**1159.5990**	**1159.5913**	**6.65**	**0**	**(22)**	**0.52**	**1**	**K.FYFENLLSK.N**
	308	670.8843	1339.7540	1339.7711	-12.71	0	(4)	18	1	R.DLKPENLLLASK.C
	309	670.8865	1339.7584	1339.7711	-9.42	0	(20)	0.47	1	R.DLKPENLLLASK.C
	310	670.8892	1339.7638	1339.7711	-5.39	0	(27)	0.088	1	R.DLKPENLLLASK.C
	311	670.8903	1339.7660	1339.7711	-3.75	0	(52)	0.00028	1	R.DLKPENLLLASK.C
	312	670.8908	1339.7670	1339.7711	-3.00	0	(33)	0.018	1	R.DLKPENLLLASK.C
	313	670.8910	1339.7674	1339.7711	-2.70	0	(35)	0.012	1	R.DLKPENLLLASK.C
	314	670.8913	1339.7680	1339.7711	-2.26	0	(53)	0.00019	1	R.DLKPENLLLASK.C
	315	670.8915	1339.7684	1339.7711	-1.96	0	(60)	3.6e-005	1	R.DLKPENLLLASK.C
	316	670.8926	1339.7706	1339.7711	-0.32	0	(58)	6.1e-005	1	R.DLKPENLLLASK.C
	317	670.8928	1339.7710	1339.7711	-0.02	0	(23)	0.17	1	R.DLKPENLLLASK.C
	318	670.8931	1339.7716	1339.7711	0.43	0	(37)	0.0077	1	R.DLKPENLLLASK.C
	319	670.8934	1339.7722	1339.7711	0.88	0	(61)	3e-005	1	R.DLKPENLLLASK.C
	320	670.8937	1339.7728	1339.7711	1.33	0	(66)	8.4e-006	1	R.DLKPENLLLASK.C
	321	670.8940	1339.7734	1339.7711	1.77	0	82	2.1e-007	1	R.DLKPENLLLASK.C
	322	670.8940	1339.7734	1339.7711	1.77	0	(67)	6.9e-006	1	R.DLKPENLLLASK.C
	323	670.8940	1339.7734	1339.7711	1.77	0	(63)	1.7e-005	1	R.DLKPENLLLASK.C
	324	670.8945	1339.7744	1339.7711	2.52	0	(51)	0.00028	1	R.DLKPENLLLASK.C
	325	670.8946	1339.7746	1339.7711	2.67	0	(70)	3.2e-006	1	R.DLKPENLLLASK.C
	326	670.8951	1339.7756	1339.7711	3.42	0	(63)	1.6e-005	1	R.DLKPENLLLASK.C
	327	670.8954	1339.7762	1339.7711	3.86	0	(63)	1.6e-005	1	R.DLKPENLLLASK.C
	328	670.8955	1339.7764	1339.7711	4.01	0	(59)	4.4e-005	1	R.DLKPENLLLASK.C
	346	468.5799	1402.7179	1402.7317	-9.86	0	(50)	0.00069	1	R.LTQYIDGQGRPR.T
	347	468.5800	1402.7182	1402.7317	-9.65	0	(51)	0.00053	1	R.LTQYIDGQGRPR.T
	348	468.5803	1402.7191	1402.7317	-9.01	0	(52)	0.00046	1	R.LTQYIDGQGRPR.T
	349	468.5805	1402.7197	1402.7317	-8.58	0	(38)	0.011	1	R.LTQYIDGQGRPR.T
	350	468.5811	1402.7215	1402.7317	-7.30	0	59	8.4e-005	1	R.LTQYIDGQGRPR.T
	351	702.3745	1402.7344	1402.7317	1.96	0	(53)	0.00035	1	R.LTQYIDGQGRPR.T
	352	702.3751	1402.7356	1402.7317	2.81	0	(39)	0.0085	1	R.LTQYIDGQGRPR.T
	353	702.3760	1402.7374	1402.7317	4.09	0	(43)	0.0035	1	R.LTQYIDGQGRPR.T
	354	702.3771	1402.7396	1402.7317	5.66	0	(18)	0.99	1	R.LTQYIDGQGRPR.T
	355	702.3782	1402.7418	1402.7317	7.23	0	(26)	0.17	1	R.LTQYIDGQGRPR.T
	360	735.4061	1468.7976	1468.8071	-6.47	0	75	1.8e-006	1	K.NLINQMLTINPAK.R
	361	735.4121	1468.8096	1468.8071	1.70	0	(71)	4.1e-006	1	K.NLINQMLTINPAK.R
	362	735.4124	1468.8102	1468.8071	2.11	0	(41)	0.0041	1	K.NLINQMLTINPAK.R
	363	735.4127	1468.8108	1468.8071	2.52	0	(70)	4.3e-006	1	K.NLINQMLTINPAK.R
	364	735.4138	1468.8130	1468.8071	4.02	0	(71)	3.5e-006	1	K.NLINQMLTINPAK.R
☑	385	**802.8809**	**1603.7472**	**1603.7406**	**4.14**	**0**	**70**	**5.2e-006**	**1**	**R.FTDDYQLFEELGK.G**
☑	386	**802.8814**	**1603.7482**	**1603.7406**	**4.77**	**0**	**(60)**	**5.5e-005**	**1**	**R.FTDDYQLFEELGK.G**
☑	387	**802.8885**	**1603.7624**	**1603.7406**	**13.6**	**0**	**(59)**	**7.2e-005**	**1**	**R.FTDDYQLFEELGK.G**
	397	542.6334	1624.8784	1624.9082	-18.39	1	45	0.0014	1	K.NLINQMLTINPAKR.I
	483	1040.9476	2079.8806	2079.9426	-29.76	0	(74)	8.8e-007	1	K.AGAYDFPSPEWDTVTPEAK.N
	484	1040.9493	2079.8840	2079.9426	-28.13	0	(58)	3.7e-005	1	K.AGAYDFPSPEWDTVTPEAK.N
	485	1040.9618	2079.9090	2079.9426	-16.11	0	(57)	5.8e-005	1	K.AGAYDFPSPEWDTVTPEAK.N

486	694.3159	2079.9259	2079.9426	-8.02	0	(70)	3.7e-006	1	K.AGAYDFPSPEWDTVTPEAK.N
487	694.3180	2079.9322	2079.9426	-4.99	0	90	3.7e-008	1	K.AGAYDFPSPEWDTVTPEAK.N
488	1040.9735	2079.9324	2079.9426	-4.86	0	(42)	0.0023	1	K.AGAYDFPSPEWDTVTPEAK.N
489	1040.9767	2079.9388	2079.9426	-1.78	0	(64)	1.7e-005	1	K.AGAYDFPSPEWDTVTPEAK.N
490	1040.9814	2079.9482	2079.9426	2.74	0	(62)	2.8e-005	1	K.AGAYDFPSPEWDTVTPEAK.N
491	1040.9825	2079.9504	2079.9426	3.80	0	(75)	1.4e-006	1	K.AGAYDFPSPEWDTVTPEAK.N

Proteins matching the same set of peptides:
IPI00228044 **Mass:** 59013 **Score:** 1126 **Queries matched:** 53
Tax_Id=10090 Gene_Symbol=Camk2g Isoform 2 of Calcium/calmodulin-dependent protein kinase type II subunit gamma
IPI00228045 **Mass:** 56610 **Score:** 1126 **Queries matched:** 53
Tax_Id=10090 Gene_Symbol=Camk2g Isoform 3 of Calcium/calmodulin-dependent protein kinase type II subunit gamma

---

5. IPI00169463 **Mass:** 50255 **Score:** 246 **Queries matched:** 13 **emPAI:** 0.21
Tax_Id=10090 Gene_Symbol=Tubb2c Tubulin beta-2C chain
☐ Check to include this hit in error tolerant search or archive report

	Query	Observed	Mr(expt)	Mr(calc)	ppm	Miss	Score	Expect	Rank	Peptide
	148	539.2706	1076.5266	1076.5250	1.49	1	13	4.6	2	K.IREEYPDR.I
☑	301	660.3567	1318.6988	1318.6955	2.53	0	80	7.9e-007	1	R.IMNTFSVVPSPK.V
☑	302	660.3600	1318.7054	1318.6955	7.54	0	(80)	7.5e-007	1	R.IMNTFSVVPSPK.V
☑	382	801.4147	1600.8148	1600.8131	1.11	0	(76)	1.4e-006	1	R.AVLVDLEPGTMDSVR.S
☑	383	801.4150	1600.8154	1600.8131	1.49	0	87	1.2e-007	1	R.AVLVDLEPGTMDSVR.S
☑	384	801.4154	1600.8162	1600.8131	1.99	0	(43)	0.0028	1	R.AVLVDLEPGTMDSVR.S
☑	432	846.4415	1690.8684	1690.8600	5.00	0	46	0.0016	1	R.ALTVPELTQQMFDAK.N
☑	434	848.9237	1695.8328	1695.8257	4.24	0	(20)	0.63	1	K.NSSYFVEWIPNNVK.T
☑	435	848.9244	1695.8342	1695.8257	5.07	0	26	0.15	1	K.NSSYFVEWIPNNVK.T
	439	854.4377	1706.8608	1706.8549	3.47	0	(13)	3.4	2	R.ALTVPELTQQMFDAK.N + Oxidation (M)
☑	584	933.4465	2797.3177	2797.3361	-6.60	0	(22)	0.23	1	R.SGPFGQIFRPDNFVFGQSGAGNNWAK.G
☑	585	933.4496	2797.3270	2797.3361	-3.27	0	(18)	0.54	1	R.SGPFGQIFRPDNFVFGQSGAGNNWAK.G
☑	586	933.4506	2797.3300	2797.3361	-2.20	0	27	0.073	1	R.SGPFGQIFRPDNFVFGQSGAGNNWAK.G

---

6. IPI00110850 **Mass:** 42052 **Score:** 234 **Queries matched:** 7 **emPAI:** 0.25
Tax_Id=10090 Gene_Symbol=Actb Actin, cytoplasmic 1
☐ Check to include this hit in error tolerant search or archive report

	Query	Observed	Mr(expt)	Mr(calc)	ppm	Miss	Score	Expect	Rank	Peptide
☑	233	581.3168	1160.6190	1160.6111	6.86	0	35	0.025	1	K.EITALAPSTMK.I
☑	452	895.9521	1789.8896	1789.8846	2.80	0	96	1.4e-008	1	K.SYELPDGQVITIGNER.F
☑	453	895.9536	1789.8926	1789.8846	4.48	0	(87)	1.1e-007	1	K.SYELPDGQVITIGNER.F
☑	468	652.0190	1953.0352	1953.0571	-11.23	0	(56)	0.00011	1	R.VAPEEHPVLLTEAPLNPK.A
	469	652.0204	1953.0394	1953.0571	-9.08	0	(30)	0.044	2	R.VAPEEHPVLLTEAPLNPK.A
☑	470	652.0221	1953.0445	1953.0571	-6.46	0	63	2.1e-005	1	R.VAPEEHPVLLTEAPLNPK.A
☑	471	652.0244	1953.0514	1953.0571	-2.93	0	(60)	4.2e-005	1	R.VAPEEHPVLLTEAPLNPK.A

Proteins matching the same set of peptides:
IPI00136929 **Mass:** 44029 **Score:** 234 **Queries matched:** 7
Tax_Id=10090 Gene_Symbol=Actg1 Gamma actin-like protein
IPI00473320 **Mass:** 42115 **Score:** 234 **Queries matched:** 7
Tax_Id=10090 Gene_Symbol=Actb Putative uncharacterized protein
IPI00652436 **Mass:** 42053 **Score:** 234 **Queries matched:** 7
Tax_Id=10090 Gene_Symbol=Actg1 Putative uncharacterized protein
IPI00874482 **Mass:** 42108 **Score:** 234 **Queries matched:** 7
Tax_Id=10090 Gene_Symbol=Actg1 Actin, cytoplasmic 2

---

7. IPI00115546 **Mass:** 40524 **Score:** 208 **Queries matched:** 3 **emPAI:** 0.17
Tax_Id=10090 Gene_Symbol=Gnao1 Isoform Alpha-2 of Guanine nucleotide-binding protein G(o) subunit alpha
☐ Check to include this hit in error tolerant search or archive report

	Query	Observed	Mr(expt)	Mr(calc)	ppm	Miss	Score	Expect	Rank	Peptide
☑	431	838.4230	1674.8314	1674.8213	6.06	0	99	8e-009	1	R.IGAGDYQPTEQDILR.T
☑	472	979.4432	1956.8718	1956.8631	4.48	0	(86)	9.3e-008	1	R.MEDTEPFSAELLSAMMR.L
☑	473	979.4452	1956.8758	1956.8631	6.52	0	92	2.4e-008	1	R.MEDTEPFSAELLSAMMR.L

Proteins matching the same set of peptides:
IPI00230192 **Mass:** 40629 **Score:** 208 **Queries matched:** 3
Tax_Id=10090 Gene_Symbol=Gnao1 Isoform Alpha-1 of Guanine nucleotide-binding protein G(o) subunit alpha
IPI00969970 **Mass:** 20777 **Score:** 208 **Queries matched:** 3
Tax_Id=10090 Gene_Symbol=Gnao1 Uncharacterized protein

---

8. IPI00221528 **Mass:** 42319 **Score:** 172 **Queries matched:** 6 **emPAI:** 0.16
Tax_Id=10090 Gene_Symbol=Actbl2 Beta-actin-like protein 2
☐ Check to include this hit in error tolerant search or archive report

	Query	Observed	Mr(expt)	Mr(calc)	ppm	Miss	Score	Expect	Rank	Peptide
	452	895.9521	1789.8896	1789.8846	2.80	0	96	1.4e-008	1	R.SYELPDGQVITIGNER.F
	453	895.9536	1789.8926	1789.8846	4.48	0	(87)	1.1e-007	1	R.SYELPDGQVITIGNER.F
	468	652.0190	1953.0352	1953.0571	-11.23	0	48	0.00078	2	R.VAPDEHPILLTEAPLNPK.I
☑	469	652.0204	1953.0394	1953.0571	-9.08	0	(31)	0.035	1	R.VAPDEHPILLTEAPLNPK.I
	470	652.0221	1953.0445	1953.0571	-6.46	0	(43)	0.0023	2	R.VAPDEHPILLTEAPLNPK.I
	471	652.0244	1953.0514	1953.0571	-2.93	0	(32)	0.028	2	R.VAPDEHPILLTEAPLNPK.I

---

9. IPI00112251 **Mass:** 50842 **Score:** 162 **Queries matched:** 8 **emPAI:** 0.21
Tax_Id=10090 Gene_Symbol=Tubb3 Tubulin beta-3 chain
☐ Check to include this hit in error tolerant search or archive report

	Query	Observed	Mr(expt)	Mr(calc)	ppm	Miss	Score	Expect	Rank	Peptide
	301	660.3567	1318.6988	1318.6955	2.53	0	80	7.9e-007	1	R.IMNTFSVVPSPK.V
	302	660.3600	1318.7054	1318.6955	7.54	0	(80)	7.5e-007	1	R.IMNTFSVVPSPK.V
☑	388	**808.4205**	**1614.8264**	**1614.8287**	**-1.40**	**0**	**(38)**	**0.011**	**1**	**R.AILVDLEPGTMDSVR.S**
☑	389	**808.4229**	**1614.8312**	**1614.8287**	**1.57**	**0**	**52**	**0.00045**	**1**	**R.AILVDLEPGTMDSVR.S**
	432	846.4415	1690.8684	1690.8600	5.00	0	46	0.0016	1	R.ALTVPELTQQMFDAK.N
	434	848.9237	1695.8328	1695.8257	4.24	0	(20)	0.63	1	K.NSSYFVEWIPNNVK.V
	435	848.9244	1695.8342	1695.8257	5.07	0	26	0.15	1	K.NSSYFVEWIPNNVK.V
	439	854.4377	1706.8608	1706.8549	3.47	0	(13)	3.4	2	R.ALTVPELTQQMFDAK.N + Oxidation (M)

10. IPI00323571 **Mass:** 35901 **Score:** 147 **Queries matched:** 8 **emPAI:** 0.42
Tax_Id=10090 Gene_Symbol=Apoe Apolipoprotein E
☐ Check to include this hit in error tolerant search or archive report

	Query	Observed	Mr(expt)	Mr(calc)	ppm	Miss	Score	Expect	Rank	Peptide
☑	92	**484.7768**	**967.5390**	**967.5451**	**-6.23**	**0**	**(56)**	**0.00011**	**1**	**R.LGPLVEQGR.Q**
☑	93	**484.7803**	**967.5460**	**967.5451**	**1.01**	**0**	**57**	**8.5e-005**	**1**	**R.LGPLVEQGR.Q**
☑	144	538.2992	1074.5838	1074.5822	1.56	0	46	0.0024	1	R.LQAEIFQAR.L
☑	145	538.2996	1074.5846	1074.5822	2.31	0	(43)	0.0043	1	R.LQAEIFQAR.L
	147	538.3003	1074.5860	1074.5822	3.61	0	(14)	3	2	R.LQAEIFQAR.L
☑	234	**581.7838**	**1161.5530**	**1161.5448**	**7.10**	**0**	**42**	**0.0054**	**1**	**K.MEEQTQQIR.L**
☑	381	**800.4018**	**1598.7890**	**1598.7787**	**6.44**	**0**	**74**	**2.7e-006**	**1**	**K.ELEEQLGPVAEETR.A**
☑	476	**997.5348**	**1993.0550**	**1993.0480**	**3.53**	**0**	**13**	**2.7**	**1**	**K.IQASVATNPIITPVAQENQ.-**

11. IPI00117352 **Mass:** 50095 **Score:** 141 **Queries matched:** 11 **emPAI:** 0.14
Tax_Id=10090 Gene_Symbol=Tubb5 Tubulin beta-5 chain
☐ Check to include this hit in error tolerant search or archive report

	Query	Observed	Mr(expt)	Mr(calc)	ppm	Miss	Score	Expect	Rank	Peptide
	148	539.2706	1076.5266	1076.5250	1.49	1	13	4.6	2	K.IREEYPDR.I
	301	660.3567	1318.6988	1318.6955	2.53	0	80	7.9e-007	1	R.IMNTFSVVPSPK.V
	302	660.3600	1318.7054	1318.6955	7.54	0	(80)	7.5e-007	1	R.IMNTFSVVPSPK.V
	388	808.4205	1614.8264	1614.8287	-1.40	0	(38)	0.011	1	R.AILVDLEPGTMDSVR.S
	389	808.4229	1614.8312	1614.8287	1.57	0	52	0.00045	1	R.AILVDLEPGTMDSVR.S
☑	429	**830.4437**	**1658.8728**	**1658.8879**	**-9.10**	**0**	**20**	**0.54**	**1**	**R.ALTVPELTQQVFDAK.N**
	434	848.9237	1695.8328	1695.8257	4.24	0	(20)	0.63	1	K.NSSYFVEWIPNNVK.T
	435	848.9244	1695.8342	1695.8257	5.07	0	26	0.15	1	K.NSSYFVEWIPNNVK.T
	584	933.4465	2797.3177	2797.3361	-6.60	0	(22)	0.23	1	R.SGPFGQIFRPDNFVFGQSGAGNNWAK.G
	585	933.4496	2797.3270	2797.3361	-3.27	0	(18)	0.54	1	R.SGPFGQIFRPDNFVFGQSGAGNNWAK.G
	586	933.4506	2797.3300	2797.3361	-2.20	0	27	0.073	1	R.SGPFGQIFRPDNFVFGQSGAGNNWAK.G

12. IPI00138892 **Mass:** 15004 **Score:** 133 **Queries matched:** 4 **emPAI:** 0.23
Tax_Id=10090 Gene_Symbol=Uba52 Ubiquitin-60S ribosomal protein L40
☐ Check to include this hit in error tolerant search or archive report

	Query	Observed	Mr(expt)	Mr(calc)	ppm	Miss	Score	Expect	Rank	Peptide
☑	448	**894.4662**	**1786.9178**	**1786.9200**	**-1.21**	**0**	**(47)**	**0.0012**	**1**	**K.TITLEVEPSDTIENVK.A**
☑	449	**894.4691**	**1786.9236**	**1786.9200**	**2.03**	**0**	**(65)**	**2.1e-005**	**1**	**K.TITLEVEPSDTIENVK.A**
☑	450	**894.4700**	**1786.9254**	**1786.9200**	**3.04**	**0**	**71**	**4.7e-006**	**1**	**K.TITLEVEPSDTIENVK.A**
☑	451	**894.4707**	**1786.9268**	**1786.9200**	**3.83**	**0**	**(53)**	**0.00033**	**1**	**K.TITLEVEPSDTIENVK.A**

Proteins matching the same set of peptides:
IPI00470152 **Mass:** 18282 **Score:** 133 **Queries matched:** 4
Tax_Id=10090 Gene_Symbol=Rps27a Ubiquitin-40S ribosomal protein S27a
IPI00895319 **Mass:** 22277 **Score:** 133 **Queries matched:** 4
Tax_Id=10090 Gene_Symbol=2810422J05Rik 22 kDa protein
IPI00895479 **Mass:** 27181 **Score:** 133 **Queries matched:** 4
Tax_Id=10090 Gene_Symbol=2810422J05Rik 27 kDa protein
IPI00918763 **Mass:** 11021 **Score:** 133 **Queries matched:** 4
Tax_Id=10090 Gene_Symbol=Uba52 Ubiqutin subunit 1
IPI00750889 **Mass:** 22822 **Score:** 133 **Queries matched:** 4
Tax_Id=10090 Gene_Symbol=Ubc Ubc protein
IPI00923013 **Mass:** 26603 **Score:** 133 **Queries matched:** 4
Tax_Id=10090 Gene_Symbol=Ubb Ubiquitin B
IPI00923037 **Mass:** 22578 **Score:** 133 **Queries matched:** 4
Tax_Id=10090 Gene_Symbol=Ubc Putative uncharacterized protein
IPI00139518 **Mass:** 34348 **Score:** 133 **Queries matched:** 4
Tax_Id=10090 Gene_Symbol=Ubb Polyubiquitin-B
IPI00755916 **Mass:** 82614 **Score:** 133 **Queries matched:** 4
Tax_Id=10090 Gene_Symbol=Ubc ubiquitin
IPI00969323 **Mass:** 82603 **Score:** 133 **Queries matched:** 4
Tax_Id=10090 Gene_Symbol=Ubc Polyubiquitin-C

13. IPI00755181 **Mass:** 57178 **Score:** 132 **Queries matched:** 5 **emPAI:** 0.12
Tax_Id=10090 Gene_Symbol=Krt10 keratin, type I cytoskeletal 10
☐ Check to include this hit in error tolerant search or archive report

	Query	Observed	Mr(expt)	Mr(calc)	ppm	Miss	Score	Expect	Rank	Peptide
☑	154	**545.7697**	**1089.5248**	**1089.5237**	**1.07**	**0**	**(46)**	**0.002**	**1**	**K.VTMQNLNDR.L**

	Query	Observed	Mr(expt)	Mr(calc)	ppm	Miss	Score	Expect	Rank	Peptide
☑	155	545.7701	1089.5256	1089.5237	1.80	0	64	3.4e-005	1	K.VTMQNLNDR.L
☑	156	545.7711	1089.5276	1089.5237	3.64	0	(62)	5.4e-005	1	K.VTMQNLNDR.L
☑	334	691.3312	1380.6478	1380.6408	5.08	0	(52)	0.00036	1	R.ALEESNYELEGK.I
☑	335	691.3317	1380.6488	1380.6408	5.80	0	61	4.3e-005	1	R.ALEESNYELEGK.I

Proteins matching the same set of peptides:
IPI00798492 **Mass:** 57906 **Score:** 132 **Queries matched:** 5
Tax_Id=10090 Gene_Symbol=Krt10 Isoform 1 of Keratin, type I cytoskeletal 10
IPI00828744 **Mass:** 57197 **Score:** 132 **Queries matched:** 5
Tax_Id=10090 Gene_Symbol=Krt10 Isoform 2 of Keratin, type I cytoskeletal 10

---

14. IPI00122094 **Mass:** 85656 **Score:** 123 **Queries matched:** 2 **emPAI:** 0.04
Tax_Id=10090 Gene_Symbol=Dlg4 Isoform 2 of Disks large homolog 4
☐ Check to include this hit in error tolerant search or archive report

	Query	Observed	Mr(expt)	Mr(calc)	ppm	Miss	Score	Expect	Rank	Peptide
☑	441	857.9645	1713.9144	1713.9090	3.18	0	83	2.7e-007	1	R.VIEDLSGPYIWVPAR.E
☑	442	857.9650	1713.9154	1713.9090	3.76	0	(68)	8e-006	1	R.VIEDLSGPYIWVPAR.E

Proteins matching the same set of peptides:
IPI00622720 **Mass:** 80765 **Score:** 123 **Queries matched:** 2
Tax_Id=10090 Gene_Symbol=Dlg4 Isoform 1 of Disks large homolog 4
IPI00626797 **Mass:** 80395 **Score:** 123 **Queries matched:** 2
Tax_Id=10090 Gene_Symbol=Dlg4 Isoform 3 of Disks large homolog 4

---

15. IPI00131366 **Mass:** 60627 **Score:** 114 **Queries matched:** 4 **emPAI:** 0.05
Tax_Id=10090 Gene_Symbol=Krt6b Keratin, type II cytoskeletal 6B
☐ Check to include this hit in error tolerant search or archive report

	Query	Observed	Mr(expt)	Mr(calc)	ppm	Miss	Score	Expect	Rank	Peptide
☑	288	651.8555	1301.6964	1301.7078	-8.74	0	(30)	0.072	1	R.SLDLDSIIAEVK.A
☑	289	651.8582	1301.7018	1301.7078	-4.59	0	(45)	0.0022	1	R.SLDLDSIIAEVK.A
☑	290	651.8605	1301.7064	1301.7078	-1.06	0	(60)	6.3e-005	1	R.SLDLDSIIAEVK.A
☑	291	651.8612	1301.7078	1301.7078	0.02	0	67	1.3e-005	1	R.SLDLDSIIAEVK.A

Proteins matching the same set of peptides:
IPI00131368 **Mass:** 59641 **Score:** 114 **Queries matched:** 4
Tax_Id=10090 Gene_Symbol=Krt6a Keratin, type II cytoskeletal 6A
IPI00139301 **Mass:** 61957 **Score:** 114 **Queries matched:** 4
Tax_Id=10090 Gene_Symbol=Krt5 Keratin, type II cytoskeletal 5
IPI00221797 **Mass:** 59932 **Score:** 114 **Queries matched:** 4
Tax_Id=10090 Gene_Symbol=Krt75 Keratin, type II cytoskeletal 75
IPI00330480 **Mass:** 35315 **Score:** 114 **Queries matched:** 4
Tax_Id=10090 Gene_Symbol=- 35 kDa protein
IPI00470126 **Mass:** 60553 **Score:** 114 **Queries matched:** 4
Tax_Id=10090 Gene_Symbol=Krt5 Uncharacterized protein
IPI00762364 **Mass:** 50641 **Score:** 114 **Queries matched:** 4
Tax_Id=10090 Gene_Symbol=Krt6a Uncharacterized protein
IPI00785403 **Mass:** 59832 **Score:** 114 **Queries matched:** 4
Tax_Id=10090 Gene_Symbol=Krt6b keratin, type II cytoskeletal 6B

---

16. IPI00134997 **Mass:** 106777 **Score:** 112 **Queries matched:** 1 **emPAI:** 0.03
Tax_Id=10090 Gene_Symbol=Dlgap3 Disks large-associated protein 3
☐ Check to include this hit in error tolerant search or archive report

	Query	Observed	Mr(expt)	Mr(calc)	ppm	Miss	Score	Expect	Rank	Peptide
☑	369	751.8735	1501.7324	1501.7274	3.39	0	112	4.7e-010	1	R.GPAGPGPGPGSGAAPEAR.S

Proteins matching the same set of peptides:
IPI00648401 **Mass:** 105576 **Score:** 112 **Queries matched:** 1
Tax_Id=10090 Gene_Symbol=Dlgap3 Discs, large (Drosophila) homolog-associated protein 3

---

17. IPI00122928 **Mass:** 50514 **Score:** 97 **Queries matched:** 6 **emPAI:** 0.13
Tax_Id=10090 Gene_Symbol=Tubb6 Tubulin beta-6 chain
☐ Check to include this hit in error tolerant search or archive report

	Query	Observed	Mr(expt)	Mr(calc)	ppm	Miss	Score	Expect	Rank	Peptide
	148	539.2706	1076.5266	1076.5250	1.49	1	13	4.6	2	K.IREEYPDR.I
☑	331	676.3433	1350.6720	1350.6676	3.31	0	78	1.2e-006	1	R.IMNTFSVMPSPK.V
	432	846.4415	1690.8684	1690.8600	5.00	0	46	0.0016	1	R.ALTVPELTQQMFDAK.N
	434	848.9237	1695.8328	1695.8257	4.24	0	(20)	0.63	1	K.NSSYFVEWIPNNVK.V
	435	848.9244	1695.8342	1695.8257	5.07	0	26	0.15	1	K.NSSYFVEWIPNNVK.V
	439	854.4377	1706.8608	1706.8549	3.47	0	(13)	3.4	2	R.ALTVPELTQQMFDAK.N + Oxidation (M)

---

18. IPI00109061 **Mass:** 50377 **Score:** 96 **Queries matched:** 10 **emPAI:** 0.14
Tax_Id=10090 Gene_Symbol=Tubb2b Tubulin beta-2B chain
☐ Check to include this hit in error tolerant search or archive report

	Query	Observed	Mr(expt)	Mr(calc)	ppm	Miss	Score	Expect	Rank	Peptide
	148	539.2706	1076.5266	1076.5250	1.49	1	13	4.6	2	K.IREEYPDR.I
	331	676.3433	1350.6720	1350.6676	3.31	0	78	1.2e-006	1	R.IMNTFSVMPSPK.V
	388	808.4205	1614.8264	1614.8287	-1.40	0	(38)	0.011	1	R.AILVDLEPGTMDSVR.S
	389	808.4229	1614.8312	1614.8287	1.57	0	52	0.00045	1	R.AILVDLEPGTMDSVR.S

	434	848.9237	1695.8328	1695.8257	4.24	0	(20)	0.63	1	K.NSSYFVEWIPNNVK.T
	435	848.9244	1695.8342	1695.8257	5.07	0	26	0.15	1	K.NSSYFVEWIPNNVK.T
☑	439	854.4377	1706.8608	1706.8549	3.47	0	20	0.61	1	R.ALTVPELTQQMFDSK.N
	584	933.4465	2797.3177	2797.3361	-6.60	0	(22)	0.23	1	R.SGPFGQIFRPDNFVFGQSGAGNNWAK.G
	585	933.4496	2797.3270	2797.3361	-3.27	0	(18)	0.54	1	R.SGPFGQIFRPDNFVFGQSGAGNNWAK.G
	586	933.4506	2797.3300	2797.3361	-2.20	0	27	0.073	1	R.SGPFGQIFRPDNFVFGQSGAGNNWAK.G

Proteins matching the same set of peptides:
IPI00338039 **Mass:** 50274 **Score:** 96 **Queries matched:** 10
Tax_Id=10090 Gene_Symbol=Tubb2a Tubulin beta-2A chain

---

19. IPI00462140 **Mass:** 61379 **Score:** 94 **Queries matched:** 3 **emPAI:** 0.05
Tax_Id=10090 Gene_Symbol=Krt77 Keratin, type II cytoskeletal 1b
☐ Check to include this hit in error tolerant search or archive report

	Query	Observed	Mr(expt)	Mr(calc)	ppm	Miss	Score	Expect	Rank	Peptide
☑	141	**532.2756**	**1062.5366**	**1062.5709**	**-32.26**	**0**	**6**	**21**	**1**	**R.AQYEIIAQK.S**
☑	367	**738.3973**	**1474.7800**	**1474.7780**	**1.40**	**0**	**(61)**	**5e-005**	**1**	**R.FLEQQNQVLQTK.W**
☑	368	**738.3973**	**1474.7800**	**1474.7780**	**1.40**	**0**	**63**	**3e-005**	**1**	**R.FLEQQNQVLQTK.W**

---

20. IPI00420970 **Mass:** 55340 **Score:** 94 **Queries matched:** 3 **emPAI:** 0.06
Tax_Id=10090 Gene_Symbol=Krt74 Keratin, type II cytoskeletal 74
☐ Check to include this hit in error tolerant search or archive report

	Query	Observed	Mr(expt)	Mr(calc)	ppm	Miss	Score	Expect	Rank	Peptide
	111	**519.2660**	**1036.5174**	**1036.5189**	**-1.41**	**0**	**5**	**24**	**5**	**R.YEVEITQR.T**
	367	738.3973	1474.7800	1474.7780	1.40	0	(61)	5e-005	1	R.FLEQQNQVLQTK.W
	368	738.3973	1474.7800	1474.7780	1.40	0	63	3e-005	1	R.FLEQQNQVLQTK.W

---

21. IPI00131209 **Mass:** 52420 **Score:** 94 **Queries matched:** 4 **emPAI:** 0.06
Tax_Id=10090 Gene_Symbol=Krt16 Keratin intermediate filament 16a
☐ Check to include this hit in error tolerant search or archive report

	Query	Observed	Mr(expt)	Mr(calc)	ppm	Miss	Score	Expect	Rank	Peptide
	154	545.7697	1089.5248	1089.5237	1.07	0	(46)	0.002	1	K.VTMQNLNDR.L
	155	545.7701	1089.5256	1089.5237	1.80	0	64	3.4e-005	1	K.VTMQNLNDR.L
	156	545.7711	1089.5276	1089.5237	3.64	0	(62)	5.4e-005	1	K.VTMQNLNDR.L
	227	**577.7924**	**1153.5702**	**1153.5220**	**41.9**	**0**	**7**	**13**	**2**	**R.MSSILAGGSCR.A + Oxidation (M)**

Proteins matching the same set of peptides:
IPI00227140 **Mass:** 53176 **Score:** 94 **Queries matched:** 4
Tax_Id=10090 Gene_Symbol=Krt14 Keratin, type I cytoskeletal 14
IPI00874585 **Mass:** 51973 **Score:** 94 **Queries matched:** 4
Tax_Id=10090 Gene_Symbol=Krt16 Keratin, type I cytoskeletal 16

---

22. IPI00348328 **Score:** 90 **Queries matched:** 3
Tax_Id=10090 Gene_Symbol=Krt78 keratin Kb40
☐ Check to include this hit in error tolerant search or archive report

	Query	Observed	Mr(expt)	Mr(calc)	ppm	Miss	Score	Expect	Rank	Peptide
	222	**577.2903**	**1152.5660**	**1152.5234**	**37.0**	**0**	**2**	**44**	**4**	**R.EYGGQVTMPR.G + Oxidation (M)**
☑	336	**692.3635**	**1382.7124**	**1382.6830**	**21.3**	**0**	**63**	**3.1e-005**	**1**	**R.SLNNQFASFIDK.V**
☑	337	**692.3693**	**1382.7240**	**1382.6830**	**29.7**	**0**	**(58)**	**0.00011**	**1**	**R.SLNNQFASFIDK.V**

---

23. IPI00322209 **Mass:** 54531 **Score:** 90 **Queries matched:** 2 **emPAI:** 0.06
Tax_Id=10090 Gene_Symbol=Krt8 Keratin, type II cytoskeletal 8
☐ Check to include this hit in error tolerant search or archive report

	Query	Observed	Mr(expt)	Mr(calc)	ppm	Miss	Score	Expect	Rank	Peptide
	336	692.3635	1382.7124	1382.7194	-5.02	1	63	3.1e-005	1	K.SLNNKFASFIDK.V
	337	692.3693	1382.7240	1382.7194	3.37	1	(58)	0.00011	1	K.SLNNKFASFIDK.V

---

24. IPI00119063 **Mass:** 523342 **Score:** 88 **Queries matched:** 4 **emPAI:** 0.01
Tax_Id=10090 Gene_Symbol=Lrp1 Prolow-density lipoprotein receptor-related protein 1
☐ Check to include this hit in error tolerant search or archive report

	Query	Observed	Mr(expt)	Mr(calc)	ppm	Miss	Score	Expect	Rank	Peptide
☑	91	**479.2393**	**956.4640**	**956.4676**	**-3.67**	**0**	**8**	**9.8**	**1**	**R.AGTSPGTPNR.I**
	128	530.2996	1058.5846	1058.5356	46.3	0	2	55	8	R.EDVVTNGIGR.V
	258	**626.3500**	**1250.6854**	**1250.7347**	**-39.36**	**0**	**0**	**51**	**2**	**R.GVAGAPPTVTLLR.S**
☑	306	**665.3570**	**1328.6994**	**1328.6976**	**1.38**	**0**	**88**	**1.2e-007**	**1**	**K.GPVGLAIDFPESK.L**

---

25. IPI00121038 **Mass:** 368613 **Score:** 78 **Queries matched:** 1 **emPAI:** 0.01
Tax_Id=10090 Gene_Symbol=Vcan Isoform V0 of Versican core protein
☐ Check to include this hit in error tolerant search or archive report

	Query	Observed	Mr(expt)	Mr(calc)	ppm	Miss	Score	Expect	Rank	Peptide
☑	474	**656.6594**	**1966.9564**	**1966.9670**	**-5.42**	**0**	**78**	**9.3e-007**	**1**	**R.VSVPTHPDDVGDASLTMVK.L**

Proteins matching the same set of peptides:

IPI00230441 **Mass:** 264594 **Score:** 78 **Queries matched:** 1
Tax_Id=10090 Gene_Symbol=Vcan Isoform V1 of Versican core protein
IPI00469172 **Mass:** 76209 **Score:** 78 **Queries matched:** 1
Tax_Id=10090 Gene_Symbol=Vcan versican core protein isoform 4
IPI00469565 **Mass:** 180325 **Score:** 78 **Queries matched:** 1
Tax_Id=10090 Gene_Symbol=Vcan versican core protein isoform 3
IPI00473932 **Mass:** 41850 **Score:** 78 **Queries matched:** 1
Tax_Id=10090 Gene_Symbol=Vcan versican core protein isoform 5
IPI00656277 **Mass:** 41758 **Score:** 78 **Queries matched:** 1
Tax_Id=10090 Gene_Symbol=Vcan Versican
IPI00875672 **Mass:** 368720 **Score:** 78 **Queries matched:** 1
Tax_Id=10090 Gene_Symbol=Vcan versican core protein isoform 1

---

26. IPI00122048 **Mass:** 113045 **Score:** 76 **Queries matched:** 2 **emPAI:** 0.03
Tax_Id=10090 Gene_Symbol=Atp1a3 Sodium/potassium-transporting ATPase subunit alpha-3
☐ Check to include this hit in error tolerant search or archive report

	Query	Observed	Mr(expt)	Mr(calc)	ppm	Miss	Score	Expect	Rank	Peptide
☑	253	618.8558	1235.6970	1235.6986	-1.26	0	(43)	0.0019	1	R.LNIPVSQVNPR.D
☑	254	618.8574	1235.7002	1235.6986	1.33	0	64	1.7e-005	1	R.LNIPVSQVNPR.D

Proteins matching the same set of peptides:
IPI00420569 **Mass:** 113457 **Score:** 76 **Queries matched:** 2
Tax_Id=10090 Gene_Symbol=Atp1a2 Sodium/potassium-transporting ATPase subunit alpha-2
IPI00752412 **Mass:** 117548 **Score:** 76 **Queries matched:** 2
Tax_Id=10090 Gene_Symbol=Atp1a3 ATPase, Na+/K+ transporting, alpha 3 polypeptide, isoform CRA_c
IPI00762871 **Mass:** 104717 **Score:** 76 **Queries matched:** 2
Tax_Id=10090 Gene_Symbol=Atp1a2 Uncharacterized protein

---

27. IPI00120793 **Mass:** 28131 **Score:** 70 **Queries matched:** 6 **emPAI:** 0.25
Tax_Id=10090 Gene_Symbol=Prnp Major prion protein
☐ Check to include this hit in error tolerant search or archive report

	Query	Observed	Mr(expt)	Mr(calc)	ppm	Miss	Score	Expect	Rank	Peptide
☑	150	544.7386	1087.4626	1087.4570	5.17	0	40	0.0024	1	K.ESQAYYDGR.R
☑	186	570.2647	1138.5148	1138.5142	0.54	0	56	0.00012	1	K.GENFTETDVK.M
☑	187	570.2665	1138.5184	1138.5142	3.70	0	(32)	0.032	1	K.GENFTETDVK.M
☑	255	622.7899	1243.5652	1243.5581	5.72	1	(13)	2.6	1	K.ESQAYYDGRR.S
☑	256	622.7908	1243.5670	1243.5581	7.17	1	24	0.2	1	K.ESQAYYDGRR.S
☑	257	622.7933	1243.5720	1243.5581	11.2	1	(7)	12	1	K.ESQAYYDGRR.S

---

28. IPI00136402 **Mass:** 111559 **Score:** 55 **Queries matched:** 1 **emPAI:** 0.03
Tax_Id=10090 Gene_Symbol=Dlgap1 Isoform 1 of Disks large-associated protein 1
☐ Check to include this hit in error tolerant search or archive report

	Query	Observed	Mr(expt)	Mr(calc)	ppm	Miss	Score	Expect	Rank	Peptide
☑	370	757.4081	1512.8016	1512.8035	-1.24	0	55	0.00018	1	R.SLDSLDPAGLLTSPK.F

Proteins matching the same set of peptides:
IPI00473783 **Mass:** 106407 **Score:** 55 **Queries matched:** 1
Tax_Id=10090 Gene_Symbol=Dlgap1 Uncharacterized protein
IPI00648461 **Mass:** 60639 **Score:** 55 **Queries matched:** 1
Tax_Id=10090 Gene_Symbol=Dlgap1 Isoform 5 of Disks large-associated protein 1
IPI00649710 **Mass:** 110576 **Score:** 55 **Queries matched:** 1
Tax_Id=10090 Gene_Symbol=Dlgap1 Isoform 2 of Disks large-associated protein 1
IPI00650032 **Mass:** 108461 **Score:** 55 **Queries matched:** 1
Tax_Id=10090 Gene_Symbol=Dlgap1 Isoform 3 of Disks large-associated protein 1
IPI00653701 **Mass:** 71554 **Score:** 55 **Queries matched:** 1
Tax_Id=10090 Gene_Symbol=Dlgap1 disks large-associated protein 1 isoform 4
IPI00918030 **Mass:** 76735 **Score:** 55 **Queries matched:** 1
Tax_Id=10090 Gene_Symbol=Dlgap1 disks large-associated protein 1 isoform 3
IPI00918186 **Mass:** 74905 **Score:** 55 **Queries matched:** 1
Tax_Id=10090 Gene_Symbol=Dlgap1 Uncharacterized protein
IPI00918416 **Mass:** 77718 **Score:** 55 **Queries matched:** 1
Tax_Id=10090 Gene_Symbol=Dlgap1 Uncharacterized protein

---

29. IPI00222548 **Mass:** 17633 **Score:** 55 **Queries matched:** 1 **emPAI:** 0.19
Tax_Id=10090 Gene_Symbol=Rpl29;Gm13841;Gm8210 60S ribosomal protein L29
☐ Check to include this hit in error tolerant search or archive report

	Query	Observed	Mr(expt)	Mr(calc)	ppm	Miss	Score	Expect	Rank	Peptide
☑	112	520.2804	1038.5462	1038.5458	0.45	0	55	0.00021	1	K.AQASAPAQAPK.G

Proteins matching the same set of peptides:
IPI00461500 **Mass:** 17603 **Score:** 55 **Queries matched:** 1
Tax_Id=10090 Gene_Symbol=Rpl29-ps2 Uncharacterized protein
IPI00662684 **Mass:** 17663 **Score:** 55 **Queries matched:** 1
Tax_Id=10090 Gene_Symbol=Gm6344 Uncharacterized protein
IPI00762791 **Mass:** 17693 **Score:** 55 **Queries matched:** 1
Tax_Id=10090 Gene_Symbol=Gm10709 Uncharacterized protein
IPI00849210 **Mass:** 17678 **Score:** 55 **Queries matched:** 1
Tax_Id=10090 Gene_Symbol=Gm5561 similar to ribosomal protein
IPI00849550 **Mass:** 28730 **Score:** 55 **Queries matched:** 1

Tax_Id=10090 Gene_Symbol=Gm12508 similar to ribosomal protein
IPI00850168 **Mass:** 22613 **Score:** 55 **Queries matched:** 1
Tax_Id=10090 Gene_Symbol=Gm5218 similar to ribosomal protein
IPI00874437 **Mass:** 17127 **Score:** 55 **Queries matched:** 1
Tax_Id=10090 Gene_Symbol=- Uncharacterized protein

---

30. IPI00124499 **Mass:** 57802 **Score:** 50 **Queries matched:** 2 **emPAI:** 0.06
Tax_Id=10090 Gene_Symbol=Krt79 Keratin, type II cytoskeletal 79
☐ Check to include this hit in error tolerant search or archive report

	Query	Observed	Mr(expt)	Mr(calc)	ppm	Miss	Score	Expect	Rank	Peptide
	141	532.2756	1062.5366	1062.5709	-32.26	0	6	21	1	K.AQYELIAQK.S
☑	307	**665.3589**	**1328.7032**	**1328.7187**	**-11.64**	**0**	**50**	**0.00068**	**1**	**R.NLDLDSIIAEVK.A**

---

31. IPI00113410 **Mass:** 38586 **Score:** 50 **Queries matched:** 1 **emPAI:** 0.09
Tax_Id=10090 Gene_Symbol=Hapln2 Hyaluronan and proteoglycan link protein 2
☐ Check to include this hit in error tolerant search or archive report

	Query	Observed	Mr(expt)	Mr(calc)	ppm	Miss	Score	Expect	Rank	Peptide
☑	342	**696.3721**	**1390.7296**	**1390.7245**	**3.70**	**0**	**50**	**0.00072**	**1**	**R.LEGVVFPYQPSR.G**

---

32. IPI00223714 **Mass:** 21964 **Score:** 47 **Queries matched:** 1 **emPAI:** 0.15
Tax_Id=10090 Gene_Symbol=Hist1h1e Histone H1.4
☐ Check to include this hit in error tolerant search or archive report

	Query	Observed	Mr(expt)	Mr(calc)	ppm	Miss	Score	Expect	Rank	Peptide
☑	250	**614.8409**	**1227.6672**	**1227.6711**	**-3.11**	**0**	**47**	**0.0013**	**1**	**K.TSGPPVSELITK.A**

---

33. IPI00136703 **Mass:** 42971 **Score:** 46 **Queries matched:** 2 **emPAI:** 0.08
Tax_Id=10090 Gene_Symbol=Ckb Creatine kinase B-type
☐ Check to include this hit in error tolerant search or archive report

	Query	Observed	Mr(expt)	Mr(calc)	ppm	Miss	Score	Expect	Rank	Peptide
☑	299	**652.3648**	**1302.7150**	**1302.7183**	**-2.51**	**0**	**(36)**	**0.016**	**1**	**K.VLTPELYAELR.A**
☑	300	**652.3654**	**1302.7162**	**1302.7183**	**-1.59**	**0**	**46**	**0.0016**	**1**	**K.VLTPELYAELR.A**

---

34. IPI00229598 **Mass:** 45025 **Score:** 40 **Queries matched:** 1 **emPAI:** 0.07
Tax_Id=10090 Gene_Symbol=Cnp Isoform CNPI of 2',3'-cyclic-nucleotide 3'-phosphodiesterase
☐ Check to include this hit in error tolerant search or archive report

	Query	Observed	Mr(expt)	Mr(calc)	ppm	Miss	Score	Expect	Rank	Peptide
☑	260	**628.8149**	**1255.6152**	**1255.6157**	**-0.34**	**0**	**40**	**0.0062**	**1**	**K.GGSQGEAVGELPR.G**

Proteins matching the same set of peptides:
IPI00319602 **Mass:** 47493 **Score:** 40 **Queries matched:** 1
Tax_Id=10090 Gene_Symbol=Cnp Isoform CNPII of 2',3'-cyclic-nucleotide 3'-phosphodiesterase

---

35. IPI00123176 **Mass:** 36804 **Score:** 39 **Queries matched:** 2 **emPAI:** 0.09
Tax_Id=10090 Gene_Symbol=Gm6316 Glyceraldehyde-3-phosphate dehydrogenase
☐ Check to include this hit in error tolerant search or archive report

	Query	Observed	Mr(expt)	Mr(calc)	ppm	Miss	Score	Expect	Rank	Peptide
☑	332	**685.3768**	**1368.7390**	**1368.7361**	**2.15**	**0**	**39**	**0.0067**	**1**	**R.GAAQNIIPASTGAAK.A**
☑	333	**685.3788**	**1368.7430**	**1368.7361**	**5.08**	**0**	**(33)**	**0.031**	**1**	**R.GAAQNIIPASTGAAK.A**

Proteins matching the same set of peptides:
IPI00135284 **Mass:** 36092 **Score:** 39 **Queries matched:** 2
Tax_Id=10090 Gene_Symbol=Gm16374 Glyceraldehyde-3-phosphate dehydrogenase
IPI00229201 **Mass:** 16676 **Score:** 39 **Queries matched:** 2
Tax_Id=10090 Gene_Symbol=3000002C10Rik Putative uncharacterized protein
IPI00271869 **Mass:** 36293 **Score:** 39 **Queries matched:** 2
Tax_Id=10090 Gene_Symbol=- 36 kDa protein
IPI00273646 **Mass:** 36072 **Score:** 39 **Queries matched:** 2
Tax_Id=10090 Gene_Symbol=Gapdh;LOC100042025 Glyceraldehyde-3-phosphate dehydrogenase
IPI00622795 **Mass:** 36074 **Score:** 39 **Queries matched:** 2
Tax_Id=10090 Gene_Symbol=- 36 kDa protein
IPI00625893 **Mass:** 36205 **Score:** 39 **Queries matched:** 2
Tax_Id=10090 Gene_Symbol=Gm6981 Glyceraldehyde-3-phosphate dehydrogenase
IPI00752289 **Mass:** 36308 **Score:** 39 **Queries matched:** 2
Tax_Id=10090 Gene_Symbol=Gm10290 Glyceraldehyde-3-phosphate dehydrogenase
IPI00848801 **Mass:** 36100 **Score:** 39 **Queries matched:** 2
Tax_Id=10090 Gene_Symbol=- 36 kDa protein
IPI00849045 **Mass:** 36072 **Score:** 39 **Queries matched:** 2
Tax_Id=10090 Gene_Symbol=- 36 kDa protein
IPI00850337 **Mass:** 44633 **Score:** 39 **Queries matched:** 2
Tax_Id=10090 Gene_Symbol=LOC100048117 similar to Glyceraldehyde-3-phosphate dehydrogenase (GAPDH) isoform 1
IPI00850377 **Mass:** 35975 **Score:** 39 **Queries matched:** 2
Tax_Id=10090 Gene_Symbol=- 36 kDa protein
IPI00850779 **Mass:** 36076 **Score:** 39 **Queries matched:** 2
Tax_Id=10090 Gene_Symbol=- 36 kDa protein
IPI00874682 **Mass:** 36038 **Score:** 39 **Queries matched:** 2
Tax_Id=10090 Gene_Symbol=- 36 kDa protein

IPI00874964 **Mass:** 36512 **Score:** 39 **Queries matched:** 2
Tax_Id=10090 Gene_Symbol=- 36 kDa protein

Peptide matches not assigned to protein hits: (no details means no match)

Query	Observed	Mr(expt)	Mr(calc)	ppm	Miss	Score	Expect	Rank	Peptide
235	582.7992	1163.5838	1163.5757	6.99	0	34	0.036	1	QMIGEFLGNR
157	551.8046	1101.5946	1101.5778	15.3	1	28	0.13	1	EEEGRITIR
48	428.7658	855.5170	855.4814	41.7	0	26	0.14	1	LGAETLPR
30	408.2383	814.4620	814.4661	-4.97	1	24	0.43	1	GTVAPSKR
34	408.2388	814.4630	814.4661	-3.74	1	24	0.45	1	GTVAPSKR
461	912.9987	1823.9828	1823.9782	2.57	0	23	0.19	1	VGINYQPPTVVPGGDLAK
53	435.7717	869.5288	869.5446	-18.17	1	20	0.39	1	ERLLAIR
344	699.8972	1397.7798	1397.8493	-49.71	1	20	0.5	1	LNKLEDTIVLIK
274	634.3542	1266.6938	1266.7084	-11.52	1	19	0.6	1	YLYGIRVEVR
47	428.7636	855.5126	855.5542	-48.53	0	19	0.62	1	TLAVLALR
276	634.3552	1266.6958	1266.7084	-9.94	1	19	0.68	1	YLYGIRVEVR
437	851.4508	1700.8870	1700.8985	-6.74	0	18	0.89	1	AVFVDLEPTVIDEVR
38	408.2404	814.4662	814.4661	0.19	1	17	1.9	1	GTVAPSKR
275	634.3550	1266.6954	1266.7084	-10.25	1	17	0.95	1	YLYGIRVEVR
172	557.7983	1113.5820	1113.5891	-6.30	1	17	1.6	1	SSPLRGPGTSR
32	408.2387	814.4628	814.4661	-3.99	1	17	2.1	1	GTVAPSKR
246	595.8132	1189.6118	1189.6489	-31.12	1	17	2	1	KTNIIDSMLR
345	699.8976	1397.7806	1397.8493	-49.14	1	16	1.2	1	LNKLEDTIVLIK
35	408.2389	814.4632	814.4661	-3.50	1	16	2.6	1	GTVAPSKR
170	557.7947	1113.5748	1113.5891	-12.77	1	16	2.1	1	SSPLRGPGTSR
171	557.7957	1113.5768	1113.5891	-10.97	1	16	2.2	1	SSPLRGPGTSR
228	578.8077	1155.6008	1155.5897	9.62	1	16	1.9	1	SSWTGHGLRR
41	413.6995	825.3844	825.4093	-30.13	0	15	1.3	1	HQDVATR
140	531.2813	1060.5480	1060.5665	-17.41	0	15	2.6	1	YLLASNAPGR
36	408.2390	814.4634	814.4661	-3.23	1	15	3.1	1	KISPNTR
31	408.2386	814.4626	814.4661	-4.23	1	15	3.3	1	GTVAPSKR
29	408.2381	814.4616	814.4661	-5.44	1	15	3	1	KISPNTR
12	602.3666	601.3593	601.3435	26.3	0	15	5.6	1	SSPALK
19	343.7292	685.4438	685.4235	29.7	1	15	1.8	1	KGAAALR
37	408.2393	814.4640	814.4661	-2.49	1	15	3.3	1	KISPNTR
45	423.2270	844.4394	844.4515	-14.24	1	15	3.9	1	AIRENSR
180	565.3195	1128.6244	1128.6754	-45.16	0	14	2.7	1	QLITDLVISK
225	577.2920	1152.5694	1152.6251	-48.29	1	14	2.6	1	EGVHAGTINKK
227	577.7924	1153.5702	1153.5840	-11.89	1	14	2.7	1	KLHDSNDGLR
40	412.7514	823.4882	823.4817	7.98	0	14	1.1	1	RPWLPR
138	531.2780	1060.5414	1060.5665	-23.63	0	14	3.6	1	YLLASNAPGR
39	412.7500	823.4854	823.5028	-21.08	1	14	1	1	VPSRLPR
223	577.2915	1152.5684	1152.6251	-49.16	1	14	3	1	EGVHAGTINKK
33	408.2388	814.4630	814.4661	-3.74	1	14	4.6	1	GTVAPSKR
148	539.2706	1076.5266	1076.5098	15.6	0	13	3.7	1	SSLDGESGGIR
143	533.2622	1064.5098	1064.5138	-3.72	0	13	3.3	1	AQYEDIAQK
61	450.7198	899.4250	899.4461	-23.38	1	13	2.3	1	REPEDVR
111	519.2660	1036.5174	1036.5011	15.7	0	13	4.2	1	VLPYMENR + Oxidation (M)
28	408.2372	814.4598	814.4661	-7.67	1	12	5.7	1	GTVAPSKR
195	572.8080	1143.6014	1143.6546	-46.49	1	12	5.2	1	QAMLRQLLR + Oxidation (M)
104	508.2908	1014.5670	1014.5709	-3.83	0	12	5.1	1	DVNAAIATIK
229	578.8155	1155.6164	1155.6611	-38.68	1	12	4.4	1	DAVSSLPLAKR
94	487.3005	972.5864	972.5869	-0.46	0	12	2.7	1	VPGHLPVVR
52	435.7706	869.5266	869.4858	46.9	0	12	2.8	1	VSGVEVPLA
285	645.8337	1289.6528	1289.6054	36.8	1	11	5.8	1	GGDYWRFHPR
159	556.8300	1111.6454	1111.6713	-23.25	1	11	2.6	1	LRAELLELR
139	531.2795	1060.5444	1060.5665	-20.80	0	11	6.9	1	YLLASNAPGR
95	491.7284	981.4422	981.4879	-46.56	0	11	3.3	1	EPAPQGQAGK
239	595.3087	1188.6028	1188.6285	-21.58	1	11	7.2	1	SPTIRDMVVR + Oxidation (M)
248	606.8034	1211.5922	1211.6371	-37.01	1	10	5.8	1	GSRGDLGLQGPR
114	520.7663	1039.5180	1039.5062	11.4	1	10	5.8	1	KFPFCWR
173	558.2787	1114.5428	1114.5731	-27.11	1	10	7	1	KSASSHIETR
179	565.3195	1128.6244	1128.6502	-22.85	1	10	7.4	1	KLELEATVAR
73	464.7799	927.5452	927.5501	-5.27	1	10	6.1	1	LNLTSPKR
82	464.7838	927.5530	927.5138	42.3	1	10	5.6	1	DVPVQSKR
358	728.8514	1455.6882	1455.7027	-9.96	0	10	6	1	DAMELKPNGGAEPK
57	444.2293	886.4440	886.4695	-28.67	1	10	10	1	KVMGPSPR + Oxidation (M)
108	517.2664	1032.5182	1032.4724	44.5	0	10	9.5	1	VLDEEGSER
27	399.7258	797.4370	797.4759	-48.75	1	9	6	1	IPVKGER
152	545.2901	1088.5656	1088.5397	23.9	0	9	11	1	MGSADRPALR + Oxidation (M)
280	643.3559	1284.6972	1284.7190	-16.91	1	9	7.9	1	NQKWLEILNK
153	545.2922	1088.5698	1088.5978	-25.71	1	9	11	1	KDFTVNLPR
240	595.3089	1188.6032	1188.5887	12.2	0	9	10	1	FVPPQTSSGNR
142	532.2774	1062.5402	1062.5128	25.9	1	9	11	1	KMEEQQVR + Oxidation (M)
81	464.7831	927.5516	927.5250	28.7	1	9	6.7	1	QSPLATRR
273	631.8077	1261.6008	1261.5826	14.4	0	9	9	1	EALEVDWSGEK
446	885.9580	1769.9014	1769.9271	-14.51	1	9	8.1	1	ELEAERQIVASQLER
243	595.3099	1188.6052	1188.6285	-19.56	1	9	11	1	SPTIRDMVVR + Oxidation (M)
76	464.7814	927.5482	927.5501	-2.02	1	9	7.7	1	LKAESKPR
105	514.8010	1027.5874	1027.5597	27.0	1	9	8.2	1	MLSKAVSHR
85	470.7675	939.5204	939.5515	-33.03	1	9	7.2	1	LRPGWRR

☑	137	530.7860	1059.5574	1059.5924	-32.98	0	9	15	1	TASLAELISR
☑	241	595.3092	1188.6038	1188.5887	12.7	0	9	11	1	FVPPQTSSGNR
☑	279	643.3487	1284.6828	1284.6357	36.7	1	8	9.9	1	GRAGVCDPALNR
☑	113	520.7655	1039.5164	1039.5121	4.23	0	8	8.9	1	SGMLSYGLGR
☑	79	464.7817	927.5488	927.5501	-1.38	1	8	8.5	1	LKAESKPR
☑	169	557.7910	1113.5674	1113.6005	-29.66	1	8	11	1	MTAFFKTLR
☑	465	948.9885	1895.9624	1895.9516	5.71	0	8	9.3	1	TITFEQFQEALEELAK
☑	182	565.3201	1128.6256	1128.6754	-44.09	0	8	11	1	QLITDLVISK
☑	226	577.2932	1152.5718	1152.6251	-46.19	1	8	12	1	EGKLEHTLAR
☑	58	449.7471	897.4796	897.4556	26.8	0	8	11	1	DAETPLPR
☑	181	565.3198	1128.6250	1128.6503	-22.33	0	8	12	1	TKPVLSSLER
☑	43	415.2015	828.3884	828.4276	-47.26	0	8	6.5	1	IHGSMLR + Oxidation (M)
☑	97	496.2269	990.4392	990.4883	-49.50	0	8	8.1	1	AFSSHSSLR
☑	445	885.9568	1769.8990	1769.8995	-0.27	1	8	11	1	LQFRDVNGEMVAVHR
☑	80	464.7817	927.5488	927.5138	37.8	1	8	10	1	DVPVQSKR
☑	77	464.7814	927.5482	927.5138	37.2	1	8	10	1	DVPVQSKR
☑	330	672.8422	1343.6698	1343.6681	1.32	1	7	12	1	AQGDEQEAQLKK
☑	222	577.2903	1152.5660	1152.5420	20.9	0	7	13	1	AGGLAAMPYMR + Oxidation (M)
☑	100	506.2394	1010.4642	1010.4781	-13.71	1	7	10	1	DYDRSLSR
☑	74	464.7807	927.5468	927.5138	35.7	1	7	12	1	DVPVQSKR
☑	338	692.3942	1382.7738	1382.7630	7.86	1	7	10	1	QSASPLRLQLDR
☑	99	500.2558	998.4970	998.4491	48.0	0	7	14	1	TMSNFLDR + Oxidation (M)
☑	283	645.8325	1289.6504	1289.6054	34.9	1	7	17	1	GGDYWRFHPR
☑	42	415.2015	828.3884	828.4276	-47.26	0	7	8.7	1	IHGSMLR + Oxidation (M)
☑	64	450.7218	899.4290	899.4324	-3.68	0	7	11	1	FMFNGLR + Oxidation (M)
☑	244	595.3101	1188.6056	1188.5887	14.2	0	7	18	1	FVPPQTSSGNR
☑	103	506.2436	1010.4726	1010.5145	-41.43	0	7	12	1	EGTQAPGVPR
☑	96	496.2260	990.4374	990.4487	-11.39	1	7	10	1	ASMSRHMR + Oxidation (M)
☑	438	852.4293	1702.8440	1702.8989	-32.20	0	6	15	1	VLSTSTDLEAAVADALK
☑	286	645.8344	1289.6542	1289.6054	37.9	1	6	18	1	GGDYWRFHPR
☑	59	450.2231	898.4316	898.4695	-42.09	0	6	11	1	MSVVPPNR
☑	196	572.8097	1143.6048	1143.6546	-43.52	1	6	20	1	QAMLRQLLR + Oxidation (M)
☑	192	572.8068	1143.5990	1143.6546	-48.59	1	6	20	1	QAMLRQLLR + Oxidation (M)
☑	161	556.8326	1111.6506	1111.5985	46.9	1	6	8.1	1	SKSHPASLASK
☑	151	545.2896	1088.5646	1088.5978	-30.49	1	6	22	1	KDFTVNLPR
☑	98	499.2703	996.5260	996.5141	12.0	1	6	16	1	EGYFRAVR
☑	184	565.3227	1128.6308	1128.6754	-39.49	0	6	17	1	QLITDLVISK
☑	75	464.7812	927.5478	927.5138	36.7	1	6	14	1	DVPVQSKR
☑	343	699.8953	1397.7760	1397.7990	-16.45	1	6	12	1	TGNDVLKALALGAR
☑	78	464.7816	927.5486	927.5501	-1.59	1	6	14	1	LKAESKPR
☑	242	595.3098	1188.6050	1188.6285	-19.71	1	6	22	1	AALKDVVACSR
☑	124	529.2703	1056.5260	1056.5087	16.4	0	6	18	1	KPETGEDPGK
☑	359	730.9042	1459.7938	1459.7453	33.3	1	6	15	1	VDILENRVMDTR
☑	278	643.3486	1284.6826	1284.6357	36.5	1	6	19	1	GRAGVCDPALNR
☑	232	581.3160	1160.6174	1160.6587	-35.53	1	6	25	1	TELSILRAMK
☑	224	577.2915	1152.5684	1152.5420	23.0	0	5	21	1	MTLNTMTWR
☑	282	645.8323	1289.6500	1289.6054	34.6	1	5	25	1	GGDYWRFHPR
☑	277	636.8104	1271.6062	1271.6357	-23.17	0	5	17	1	LLDAESEDRPK
☑	88	470.7690	939.5234	939.5515	-29.84	1	5	16	1	LRPGWRR
☑	185	565.3278	1128.6410	1128.6251	14.1	1	5	21	1	AGLSDLELRR
☑	55	441.2665	880.5184	880.5355	-19.36	1	5	9	1	SPRLRPR
☑	168	556.8366	1111.6586	1111.6462	11.2	1	5	9.8	1	SPRISRPTAK
☑	249	612.3348	1222.6550	1222.6346	16.7	0	5	22	1	LDGNAIFFQAK
☑	284	645.8327	1289.6508	1289.6054	35.2	1	5	27	1	GGDYWRFHPR
☑	462	612.9324	1835.7754	1835.7858	-5.70	1	5	6.7	1	DESLVSRAMEYMFGW + Oxidation (M)
☑	247	597.3036	1192.5926	1192.5724	17.0	1	5	28	1	KEGTEWSQTK
☑	183	565.3204	1128.6262	1128.6503	-21.27	0	5	26	1	TKPVLSSLER
☑	56	441.2668	880.5190	880.4879	35.4	1	5	9.6	1	GLDGRHVK
☑	115	523.7672	1045.5198	1045.5152	4.45	1	5	35	1	QEQESARAK
☑	160	556.8324	1111.6502	1111.5985	46.5	1	4	12	1	SKSHPASLASK
☑	502	1073.0221	2144.0296	2144.0498	-9.39	1	4	18	1	KLQNSIQESTQNFDDHLK
☑	84	470.7674	939.5202	939.5614	-43.77	1	4	20	1	NRLPIATR
☑	90	475.7499	949.4852	949.5089	-24.87	1	4	32	1	LCMSLKAK
☑	520	747.3844	2239.1314	2239.2259	-42.22	1	4	18	1	GAFTGLHSLKVLMLQNNQLR
☑	54	435.7731	869.5316	869.5123	22.2	0	4	15	1	VSLWLPR
☑	163	556.8347	1111.6548	1111.6462	7.80	1	4	12	1	KPQLDTRVR
☑	83	470.7668	939.5190	939.5515	-34.52	1	4	21	1	LRPGWRR
☑	236	586.7966	1171.5786	1171.6271	-41.33	0	4	26	1	MNLPSPVISSK
☑	67	450.7224	899.4302	899.4032	30.1	0	4	19	1	HSGCAVNR
☑	365	735.9140	1469.8134	1469.7435	47.6	1	4	18	1	TTLLQEEYAKMK + Oxidation (M)
☑	522	747.3883	2239.1431	2239.1346	3.80	1	4	18	1	VSQSGGTGHANPTFKLQTPQGK
☑	281	645.8319	1289.6492	1289.6292	15.5	1	4	32	1	TKEFWSTYTK
☑	109	517.2719	1032.5292	1032.5564	-26.28	0	4	35	1	VSQGSTVISR
☑	467	650.9503	1949.8291	1949.9001	-36.40	0	4	9.3	1	EGGGTELVGTVMTSNTPER + Oxidation (M)
☑	238	595.3069	1188.5992	1188.6285	-24.60	1	4	33	1	SPTIRDMVVR + Oxidation (M)
☑	164	556.8352	1111.6558	1111.6462	8.70	1	4	14	1	KPQLDTRVR
☑	44	422.2433	842.4720	842.4610	13.1	0	4	37	1	VAVEGNVR
☑	178	561.2647	1120.5148	1120.5625	-42.53	1	3	32	1	GAFSNNERVK
☑	251	616.8057	1231.5968	1231.5680	23.4	1	3	33	1	EENEAEKVER
☑	258	626.3500	1250.6854	1250.6329	42.0	0	3	25	1	YLPTCLDTLR
☑	123	529.2694	1056.5242	1056.5352	-10.40	0	3	30	1	NGFVLHENK

427	552.5915	1654.7527	1654.7708	-10.97	1	3	20	1	MRGPCQLFDFTGAR
87	470.7683	939.5220	939.5250	-3.15	1	3	23	1	PRAVPSGTR
158	556.7419	1111.4692	1111.5121	-38.51	1	3	12	1	WKMEFDTR
503	1073.0231	2144.0316	2144.0233	3.89	0	3	25	1	KPDLENVSSEGGGGTLDNLDK
24	370.6898	739.3650	739.3357	39.7	0	3	23	1	MTMGLR + 2 Oxidation (M)
245	595.3107	1188.6068	1188.5631	36.8	0	3	42	1	LDMPQGMIPR + 2 Oxidation (M)
20	349.2049	696.3952	696.3629	46.5	0	3	18	1	MVIYR + Oxidation (M)
66	450.7223	899.4300	899.4726	-47.29	1	3	25	1	HFPSTRR
10	577.2819	576.2746	576.2867	-21.00	0	3	46	1	SSAQGK
455	897.9139	1793.8132	1793.7699	24.2	1	3	26	1	MSYLKTTMEDEESSK + Oxidation (M)
576	916.4275	2746.2607	2746.2996	-14.16	1	3	17	1	QAPPQYTFIGELNSMGIWYRMGR + 2 Oxidation (M)
62	450.7201	899.4256	899.4647	-43.42	0	3	24	1	SLQHMLR + Oxidation (M)
13	323.1536	644.2926	644.3242	-48.93	0	3	16	1	GAASPSR
86	470.7677	939.5208	939.5548	-36.18	1	3	28	1	MRKPRPR
89	470.7696	939.5246	939.5515	-28.56	1	3	25	1	LRPGWRR
166	556.8363	1111.6580	1111.6423	14.1	1	3	18	1	MKPKASLPPK + Oxidation (M)
17	325.1857	648.3568	648.3377	29.5	1	3	42	1	MAAGKR + Oxidation (M)
63	450.7214	899.4282	899.4647	-40.53	0	2	28	1	SLQHMLR + Oxidation (M)
357	728.3519	1454.6892	1454.7617	-49.78	1	2	34	1	VVSSTSLFESSGKK
68	450.7227	899.4308	899.4686	-41.92	1	2	30	1	GAAAGGRGGAR
60	450.7193	899.4240	899.4647	-45.20	0	2	26	1	LNTHLMR + Oxidation (M)
165	556.8359	1111.6572	1111.6713	-12.66	1	2	19	1	GVKALIQDIR
454	897.9121	1793.8096	1793.7699	22.2	1	2	28	1	MSYLKTTMEDEESSK + Oxidation (M)
101	506.2419	1010.4692	1010.4525	16.6	1	2	32	1	RMDMDTVK + Oxidation (M)
162	556.8341	1111.6536	1111.5986	49.6	1	2	21	1	QPLSGTPKER
532	762.0566	2283.1480	2283.2620	-49.94	1	2	29	1	EKSLVAQLAAAQLELQMTALR
65	450.7219	899.4292	899.4647	-39.42	0	2	31	1	SLQHMLR + Oxidation (M)
107	516.3027	1030.5908	1030.5593	30.6	0	2	42	1	LMTGAGNVLR
521	747.3854	2239.1344	2239.0625	32.1	1	2	32	1	VACSGGAPGAGGRGGAGGAAAAGAGAAGR
106	516.2996	1030.5846	1030.5407	42.7	1	2	47	1	ELDTLRER
9	575.3143	574.3070	574.2962	18.8	1	2	1.1e+002	1	KDLEA
252	618.2717	1234.5288	1234.5724	-35.29	1	2	24	1	ADTSQPSARMR + Oxidation (M)
430	838.3983	1674.7820	1674.8246	-25.43	1	2	41	1	ALDGLQREAMEELGK + Oxidation (M)
525	762.0536	2283.1390	2283.1894	-22.10	0	1	33	1	EEVIDFSKPFMSLGISIMIK
526	762.0540	2283.1402	2283.1190	9.26	1	1	33	1	KGTTTNATATSTSTASTAVADAQK
480	688.3133	2061.9181	2061.9426	-11.89	1	1	26	1	SKGSQGTSPFPMSPPSPDSR + Oxidation (M)
380	795.3489	1588.6832	1588.7515	-42.96	1	1	19	1	TPEEVICERSQNK
479	688.3128	2061.9166	2061.9426	-12.61	1	1	25	1	SKGSQGTSPFPMSPPSPDSR + Oxidation (M)
259	627.2626	1252.5106	1252.5587	-38.34	0	1	12	1	VWNYFSYMK + Oxidation (M)
8	562.3096	561.3023	561.3122	-17.64	0	1	84	1	TLGSGK
14	647.3585	646.3512	646.3438	11.4	0	1	93	1	LYSHK
530	762.0560	2283.1462	2283.1205	11.2	0	1	39	1	DWDPGLPIIDNLMQSINQSK
444	882.9548	1763.8950	1763.8889	3.46	1	1	54	1	NIPWRCVVIDEAHR
537	767.0458	2298.1156	2298.1525	-16.07	1	1	39	1	MSLSLEVATYRTLLEAENSR + Oxidation (M)
587	1414.1199	2826.2252	2826.3139	-31.38	1	1	16	1	HGRAGVPMEVMGLMLGEFVDDYTVR + 3 Oxidation (M)
102	506.2426	1010.4706	1010.4855	-14.70	0	1	45	1	SCLEFSLR
26	396.7331	791.4516	791.4177	42.8	0	0	55	1	EFIGAQK
18	325.1862	648.3578	648.3629	-7.74	1	0	73	1	GLGMKK + Oxidation (M)
504	546.7143	2182.8281	2182.8329	-2.22	0	0	4.1	1	CFPCPDQGCPQMGHYADK + Oxidation (M)
339	692.3972	1382.7798	1382.7154	46.6	1	0	45	1	KDPSQTSPLGTPR
1	369.8990	368.8917							
2	412.8516	411.8443							
3	429.1010	428.0937							
4	445.1302	444.1229							
7	460.2643	459.2570							
15	325.1771	648.3396							
16	325.1830	648.3514							
21	350.1520	698.2894							
22	360.7044	719.3942							
23	360.7065	719.3984							
25	396.7322	791.4498							
46	423.7246	845.4346							
110	519.1532	1036.2918							
122	528.3091	1054.6036							
237	588.3435	1174.6724							
287	648.3742	1294.7338							
340	692.8701	1383.7256							
341	692.8714	1383.7282							
356	720.3672	1438.7198							
366	736.4177	1470.8208							
371	757.8600	1513.7054							
372	766.3796	1530.7446							
373	766.3809	1530.7472							
374	775.8672	1549.7198							
375	785.8416	1569.6686							
376	785.8436	1569.6726							
377	785.8450	1569.6754							
378	785.8463	1569.6780							
379	785.8502	1569.6858							
393	810.3764	1618.7382							
428	828.4725	1654.9304							

☑	433	847.4376	1692.8606
☑	436	849.4251	1696.8356
☑	440	855.4716	1708.9286
☑	447	444.8585	1775.4049
☑	456	897.9154	1793.8162
☑	457	897.9534	1793.8922
☑	458	906.4670	1810.9194
☑	459	906.4675	1810.9204
☑	460	906.4682	1810.9218
☑	463	924.9924	1847.9702
☑	464	617.9799	1850.9179
☑	466	948.9913	1895.9680
☑	481	1036.4299	2070.8452
☑	482	1038.5869	2075.1592
☑	492	701.6434	2101.9084
☑	493	701.6444	2101.9114
☑	497	1062.4862	2122.9578
☑	505	1093.5850	2185.1554
☑	506	737.6982	2210.0728
☑	507	737.6986	2210.0740
☑	508	737.6988	2210.0746
☑	509	737.6998	2210.0776
☑	510	737.7000	2210.0782
☑	511	737.7000	2210.0782
☑	512	737.7003	2210.0791
☑	513	737.7007	2210.0803
☑	514	737.7010	2210.0812
☑	515	737.7013	2210.0821
☑	516	737.7016	2210.0830
☑	517	737.7018	2210.0836
☑	518	737.7018	2210.0836
☑	519	737.7036	2210.0890
☑	523	761.7198	2282.1376
☑	524	761.7235	2282.1487
☑	527	762.0549	2283.1429
☑	528	762.0551	2283.1435
☑	529	762.0555	2283.1447
☑	531	762.0562	2283.1468
☑	533	762.0594	2283.1564
☑	534	1144.1368	2286.2590
☑	535	767.0423	2298.1051
☑	536	767.0435	2298.1087
☑	538	779.0403	2334.0991
☑	539	1168.5659	2335.1172
☑	557	1259.6788	2517.3430
☑	574	916.4235	2746.2487
☑	575	916.4275	2746.2607
☑	577	917.0772	2748.2098
☑	578	917.0789	2748.2149
☑	579	917.0808	2748.2206
☑	580	917.0855	2748.2347
☑	581	922.4243	2764.2511
☑	582	927.0934	2778.2584
☑	583	927.7480	2780.2222
☑	588	1414.1230	2826.2314
☑	589	1415.1056	2828.1966
☑	590	1415.1093	2828.2040
☑	591	975.8646	2924.5720
☑	592	1015.5191	3043.5355
☑	593	1179.7867	4715.1177
☑	594	1572.7246	4715.1520

Search Parameters

```
Type of search         : MS/MS Ion Search
Enzyme                 : Trypsin
Fixed modifications    : Carbamidomethyl (C)
Variable modifications : Oxidation (M)
Mass values            : Monoisotopic
Protein Mass           : Unrestricted
Peptide Mass Tolerance : ± 50 ppm
Fragment Mass Tolerance: ± 0.2 Da
Max Missed Cleavages   : 1
Instrument type        : ESI-QUAD-TOF
Number of queries      : 594
```

Mascot: http://www.matrixscience.com/

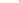

Supplement: Supplementary Mascot Searches S1 — Composite file of results from Mascot searches on LC-MS/MS data. The file is composed of Mascot single searches of SAF derived from uninfected mice and PrP−/− as well as SAF derived from 2 replicate preparations each of wild type mice infected with ME7, 22F and 79A strains of mouse passaged scrapie. (PDF) [file pone.0026813.s004.pdf]
